# Mitigating environmental pollution by methyl orange via effective adsorption using modified layered double hydroxide

**DOI:** 10.1038/s41598-026-52336-7

**Published:** 2026-08-20

**Authors:** I. M. Ahmed, AbdElAziz A. Nayl, Weal A.A. Arafa, Ahmed Hamad Alanazi, Ahmed I. Abd-Elhamid, Stefan Bräse

**Affiliations:** 1 Department of Chemistry, College of Science, Jouf University, Sakaka, 72341 Aljouf Saudi Arabia; 2 Composites and Nanostructured Materials Research Department, Advanced Technology and New Materials Research Institute, City of Scientific Research and Technological Applications (SRTA-City), NewBorgAl-Arab, 21934 Egypt; 3 Institute of Biological and Chemical Systems – Functional Molecular Systems (IBCS-FMS), Kaiserstrasse 12, 76131 Karlsruhe, Germany

**Keywords:** Environmental Pollution, Methyl orange, Activated carbon, Layered double hydroxide, Kinetics and equilibrium studies, Chemistry, Environmental sciences

## Abstract

In alignment with global trends toward environmental and economic sustainability, this study focused on the preparation of layered double hydroxide (LDH) and its modification with activated carbon, as a sustainable material, to produce LDH–AC aimed at enhancing the sorption efficiency of methyl orange (MO) from aqueous solutions. Both materials were characterized via SEM, FTIR, XRD, and BET analysis confirming successful modification. Batch experiments revealed pseudo-second-order kinetics (R² > 0.99) with intra-particle diffusion as a rate-limiting step for LDH–AC. Langmuir isotherm fitting yielded maximum capacities of 71.43 mg/g (LDH) and 393.70 mg/g (LDH–AC) at 298 K and pH 5.5. Thermodynamic parameters (ΔH° = +18.54 and 35.77 kJ/mol for LDH and LDH–AC, respectively, and ΔG° = -0.069 and − 3.78 kJ/mol for LDH and LDH–AC, respectively, ) indicated an endothermic and spontaneous process. The greater efficiency of the LDH–AC composite was demonstrated by comparison with other sorbents reported in the literature. As a result, this sorbent is suggested as a practical way to eliminate MO while providing environmental benefits.

**Supplementary Information:**

The online version contains supplementary material available at 10.1038/s41598-026-52336-7.

## Introduction

Water pollution from organic dyes is a major environmental concern owing to their widespread use in sectors such as textiles, leather, paper, plastics, and cosmetics^1^. These dyes are frequently discharged into aquatic systems through industrial effluents, resulting in significant water pollution. Organic dyes pose a significant problem because they are difficult to break down, toxic, cancer-causing, and highly harmful to both humans and aquatic life. Synthetic azo dyes are prominent organic pollutants, valued for their ease of synthesis, low cost, and capacity to generate a diverse array of colouring agents^2^.

Methyl orange is one of these azo dyes, which have one or more azo groups. The colour of the dye comes from these groups and some chromophores. It is widely used in textiles, printing, medicines, cosmetics, and food^3^. Various industries release millions of tons of methyl orange into wastewater, which then mixes with water bodies and affects microbial communities^4^. Also, methyl orange is a mutagen and highly toxic because of its aromatic structure, which makes it a xenobiotic. They don’t break down in the environment and accumulate in water sources, which causes significant difficulties for the ecosystem^3^. Consequently, the presence of organic dyes, especially methyl orange, in water is a serious environmental problem that requires effective remediation.

Different techniques, such as biological treatments, chemical procedures, and physical methods, have all shown promise in breaking down organic contaminants^5^^7^. It is important to assess the benefits and drawbacks of each method. The physical mechanisms work well only with small amounts and often produce by-products that can’t be digested^8^. Chemical techniques can be costly and have undesirable consequences. Some biological approaches, such as bioremediation, often require microorganisms that have already been grown and specific environmental conditions^9^. Therefore, subsequent studies must enhance existing methodologies, investigate novel materials and technologies, and integrate various approaches to achieve sustainable and effective elimination of organic dyes from water.

Adsorption is a well-known method that is easy to use, effective, environmentally friendly, and cost-effective [10-13]^10^^13^. Choosing the right adsorbent materials is necessary for using adsorption techniques. This can be done through effective synthesis or modification processes. Adding supporting materials to adsorbents to form composites can improve their adsorption capacity, selectivity, stability, and reusability, thereby increasing their potential^14^^-17^.

Layered double hydroxides (LDH) are a type of two-dimensional inorganic layered substance made up of brucite-like sheets and anions that sit between the sheets. There is a positively charged metal layer at the surface and water, and a negatively charged anion-exchange layer in the interlayer area. LDH is usually written as [M^2 +^ _1−x_ M^3 +^ _x_ (OH)_2_]^x+^(A^n−^)_x/n_‧yH_2_O. It is composed of MO6 octahedra that share edges and contain M^2+^ and M^3+^ metal cations. The symbol x is the molar ratio of M^3+^ to (M^2+^+M^3+^), and A^n−^ is the interlayer anion that balances off the extra positive charges on the sheets^14^^18^. Recently, LDH-based catalysts have shown outstanding performance in Fenton-like reactions because they feature multivalent metal cations, well-distributed active sites, efficient separation, and simple, inexpensive manufacturing techniques^19^^21^. Also, the LDH structure has many hydroxyl groups, which confer basic properties and pH-buffering capacity due to its high pHzpc^22^. Several multivalent iron-containing LDH, including CoFe, NiFe, and MnMgFe, were produced and applied as Fenton catalysts to treat organic wastewater^23^^24^.

Further, the affinity of the LDH material was enhanced by a suitable combination with activated carbon (AC), as the LDH–AC composite offers a range of advantages, including low cost, eco-friendliness, and a large surface area for the LDH substrate.

However, a major problem with LDHs is that they aren’t very stable over long periods and are difficult to separate or regenerate because they tend to exfoliate. To improve LDH effectiveness, it is suggested to add support material particles^25^^26^. Several investigations have reported alterations in LDHs via the integration of diverse supporting materials, such as charcoal^27^, metal oxides^28^, oxalate^29^, chitosan^30^, graphite^31^, and humic acid^32^.

The main aim of this study is to develop and evaluate a sustainable, low-cost composite sorbent, termed LDH–AC, by modifying layered double hydroxide (LDH) with activated carbon (AC) for the efficient removal of methyl orange (MO), which is considered as a prevalent refractory azo dye, from aqueous solutions. This addresses the pressing global challenge of textile wastewater pollution, where conventional treatments often fall short in efficiency, cost, and environmental impact. The physicochemical features of LDH–AC were investigated. Also, some factors affecting the sorption efficiency of methyl orange by LDH–AC from the liquid phase were established.

## Experimental

### Chemicals

The activated carbon (AC) and nitric acid (67%) were purchased from Merck. Methyl orange (MO), magnesium nitrate hexahydrate, Mg(NO_3_)_2_.6H_2_O, and ferric nitrate, Fe(NO_3_)_3_.9H_2_O, were supplied from Fluka Chemika, Switzerland. All chemicals were of analytical grade.

### Modification of activated carbon (AC)

The activated carbon used in this study was modified by oxidizing its surface with concentrated nitric acid (diluted 1:1 from 67% HNO3). This is how the modification process went: boiled water was used to wash the commercial AC for two hours. For 24 h, the wet AC was dried at 90 °C. Five grams of dried AC were mixed with 50 mL (1:1) HNO_3_ and left to sit overnight. The mixture was then filtered and rinsed with deionized water under vacuum filtration until the permeate pH was about 7. Finally, the cleaned AC was put in a vacuum oven at 90 °C overnight to dry. After that, the dried AC was sent through mesh sizes 50–100 for more studies.

### Synthesis of LDH-AC composite

0.2 g of the modified AC, 0.15 mol/L of Mg(NO_3_)_2_.6H_2_O [6.06 g], and 0.05 mol/L of Fe(NO_3_)_3_.9H_2_O [1.28 g] were mixed in a 300 mL flask with 100 mL of deionized water and sonicated for 1 h. The reaction mixture was continuously agitated, and a solution of sodium hydroxide (2 mol/L) was added slowly until the pH reached 10. The pH was adjusted for four hours, and then the reaction mixture was maintained at 80 °C overnight. The resultant composite, layered double hydroxide–AC (LDH–AC), was filtered, washed several times with water, and dried at 90 °C. For comparison, LDH was prepared without AC.

### Characterization of LDH–AC composite

Surface morphology of both LDH–AC composite and LDH powders was carried out using Scanning Electron Microscopy (SEM), Thermofisher-Quanta FEG 250. The composite samples were washed, dried, mounted on a support, and then made conductive with sputtered gold. The FT-IR spectrum was analyzed at 500–4000 cm-1 on a spectrometer (FTIR), IR–Tracer 100, Shimadzu, Japan, of the sample. The crystallinity of LDH before and after the modification was examined by X-ray Diffraction using PANalytical (Netherlands) Model: X’PertPRO (MPD). Additionally, BET analysis of both LDH and LDH–AC was carried out. The surface area was measured using the Brunauer-Emmett-Teller (BET) method. This method involves nitrogen adsorption at liquid nitrogen temperatures (77 K) and provides a value known as the BET surface area.

### Sorption experiments

#### Effect of pH

The batch technique was employed to explore how methyl orange adsorbs from water onto LDH and LDH–AC. A quantity of 0.03 g of LDH–AC and/or LDH was mixed with 10 mL of a methyl orange solution at a concentration of 100 mg/L (*C*_*o*_) at different pH values (3.0–11.0) at room temperature for 3.0 h. The solutions were centrifuged at 6000 rpm once equilibrium was reached. The filtrate was then utilized to find the dye’s equilibrium concentration (*C*_*e*_) using a spectrophotometer. All measurements were validated in duplicates. The distribution coefficient (Kd, mL/g) and the percent absorption (*R%*) of methyl orange were calculated as follows.

1 $${K_d}(mL/g)=\frac{{{C_o}-{C_e}}}{{{C_e}}}x\frac{V}{m}$$

2 $$R\% =\frac{{{C_o}-{C_e}}}{{{C_e}}}\times 100$$

where *V* (mL) is the volume of methyl orange, and *m* (g) is the mass of the adsorbent.

The concentration of MO was measured by a UV-vis spectrometer at 465 nm.

#### Effect of sorbent mass

A collection of bottles containing varying amounts (0.01–0.03 g) of LDH and/or LDH–AC, along with 10 mL of 100 mg/L MO, were agitated at 250 rpm and ambient temperature for 24 h to ensure equilibrium was achieved. The sorbent and sorbate were centrifuged at 6000 rpm, and the equilibrium concentration of MO in the sorbate was measured spectrophotometrically. The percentage absorption (*R%*) of MO was calculated using Eq. (2).

#### Kinetic studies

To determine how long it would take to reach equilibrium, a series of 50 mL glass bottles was used. Each bottle contained 0.03 g of LDH and/or LDH–AC and 10 mL of a 100 mg/L MO solution at the optimal pH. For varying times (2 to 120 min), the mixtures were stirred at 200 rpm and room temperature. The liquid and solid phases were separated, and the concentration of the ions, *C*_*t*_, at time *t*, was determined in the filtrate. Equation (2) was used to determine the percentage of MO taken up after replacing *C*_*e*_ with *C*_*t*_.

#### Equilibrium isotherm investigations

Batch equilibrium experiments were conducted at a liquid-to-solid ratio (*V/m*) of 0.03 L/g, utilizing various dye concentrations ranging from 100 to 600 mg/L at ambient temperature. The mixes were stirred to achieve equilibrium. The two phases were separated, and the equilibrium concentration of *C*_*e*_ (mg/L) in the filtrate was quantified. The amount of dye adsorbed onto both LDH and LDH–AC at equilibrium, *q*_*e*_ (mg/g), was calculated as follows.

3 $${q_e}(mg/g)=\left( {{C_o} - {C_e}} \right)x\frac{V}{m}$$

## Results and discussion

### Characterization of the prepared materials

**Fig. 1:**
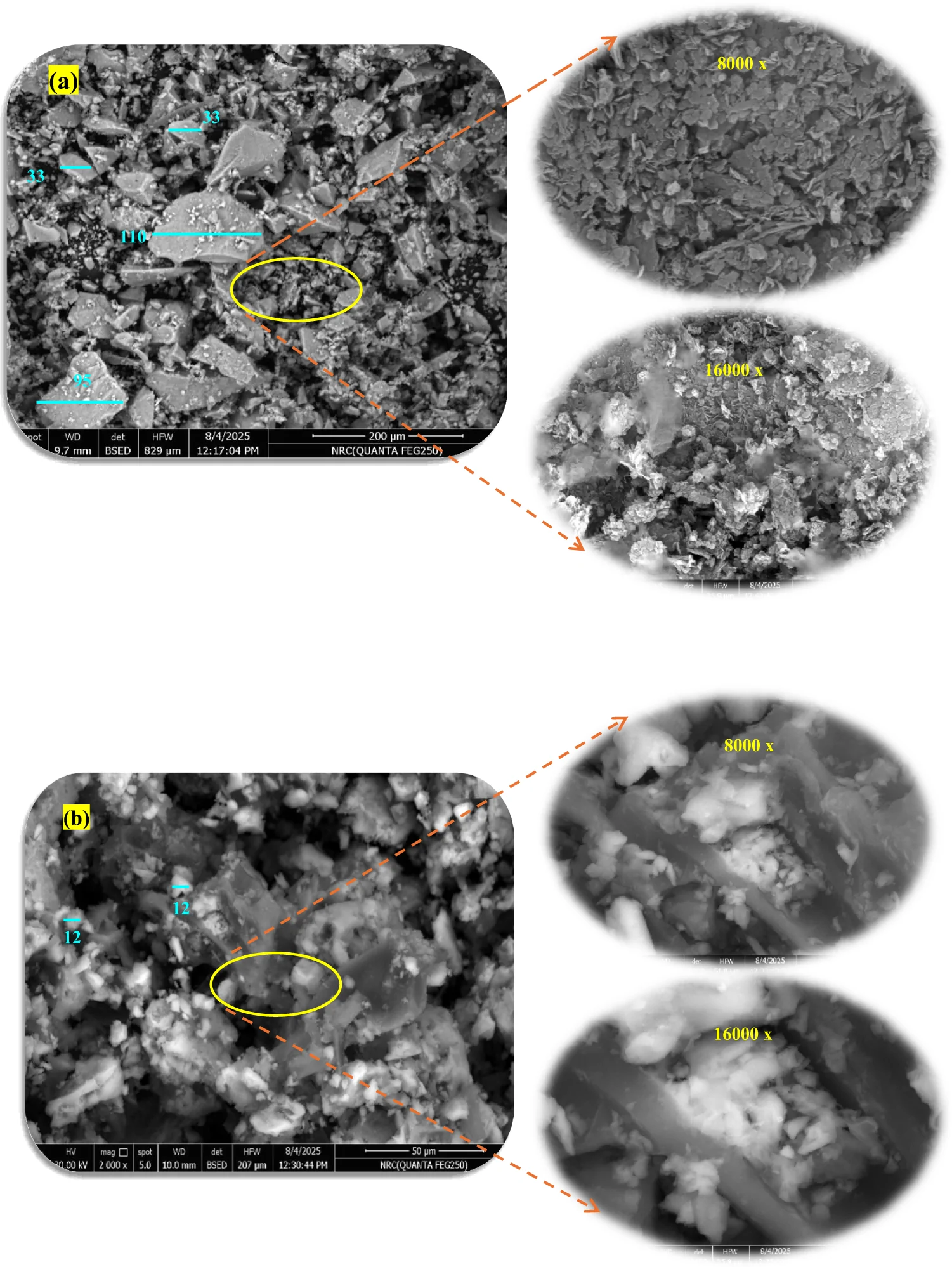
SEM images for the synthesized (**a**) LDH and (**b**) LDH–AC.

The morphological structure of LDH and the LDH–AC composite was examined, as illustrated in Fig. 1. The figure shows that LDH has a micro-sized structure averaging approximately 33 μm, with fine particles aggregating to form larger particles of about 110 μm. Figure 1(a) shows that LDH and its composite have semi-hexagonal sheets. LDH particles were prepared with AC layers, yielding a well-designed porous material (Fig. 1(b)). The LDH–AC has a lower particle size of about 12 μm because LDH and AC are mixed. This is consistent with the findings reported in the literature^33^.

**Fig. 2:**
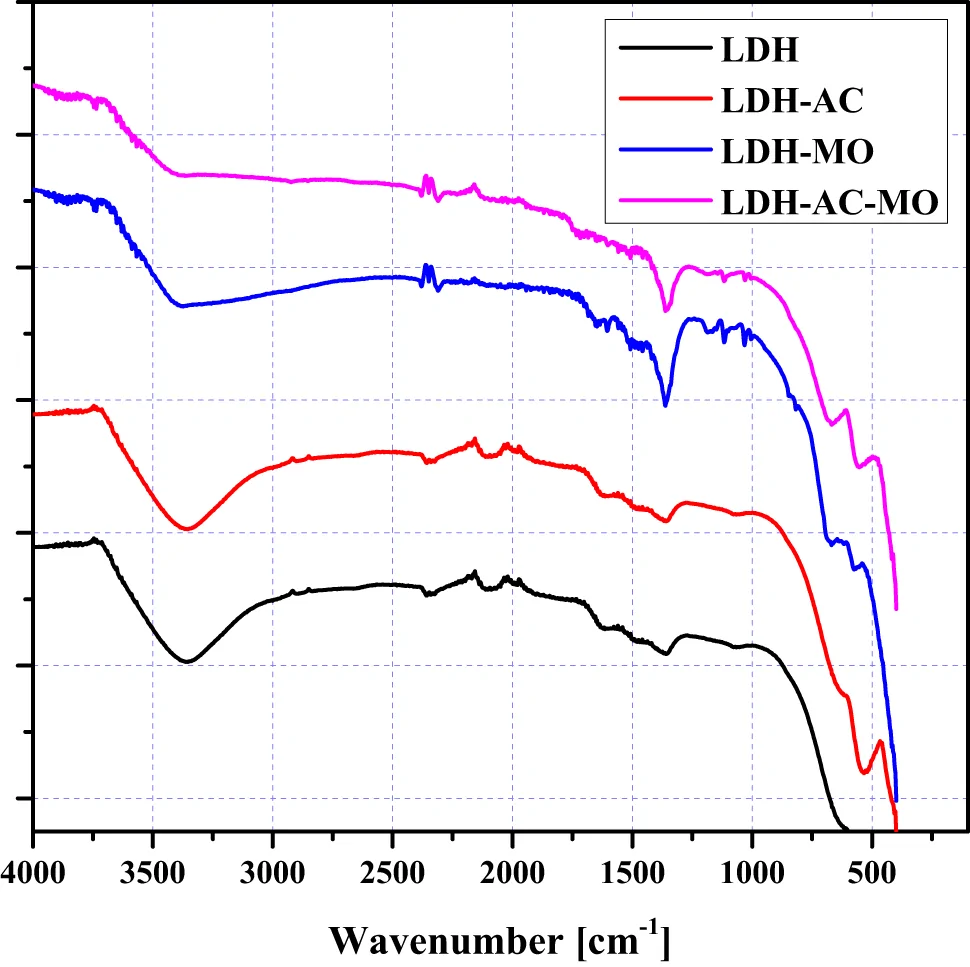
FT–IR spectra for LDH, LDH–AC, LDH–MO, and LDH–AC–MO.

FT–IR analysis was employed to ascertain the molecular structure of the synthesized material (LDH) and to validate the interaction between LDH and AC. We found the functional groups in LDH, LDH-MO, LDH-AC, and LDH-AC-MO. Figure 2 shows the spectrum that came out of this. In the high-frequency range, two separate absorption bands show up about 3380 cm⁻¹. These bands are due to the stretching vibrations of interlayer water hydroxyl groups (O–H) and magnesium–iron–bound hydroxyl groups of hydrotalcite for all samples. The O–H bending vibration characteristic peaks of water molecules trapped between the layers and surfaces of LDHs may be observed at 1646 cm^− 1^. The C–O band of AC was seen at 1002 cm^− 1^. This showed that the C–O of AC and the LDH were interacting at the molecular level^32^. The COOH group of AC transformed into a carboxylate, and the free OH absorption band of LDH became hydrogen-bound, indicating the intercalation of AC into LDH layers ^34,35^, as seen in Fig. 2. There is a sharp peak at 1380 cm^− 1^ because MO is chemisorbed on LDH and LDH–AC. This is shown by functional groups such as –COO that sorb MO via collateral interactions, electrostatic interactions, and ion exchange^36^.

**Fig. 3:**
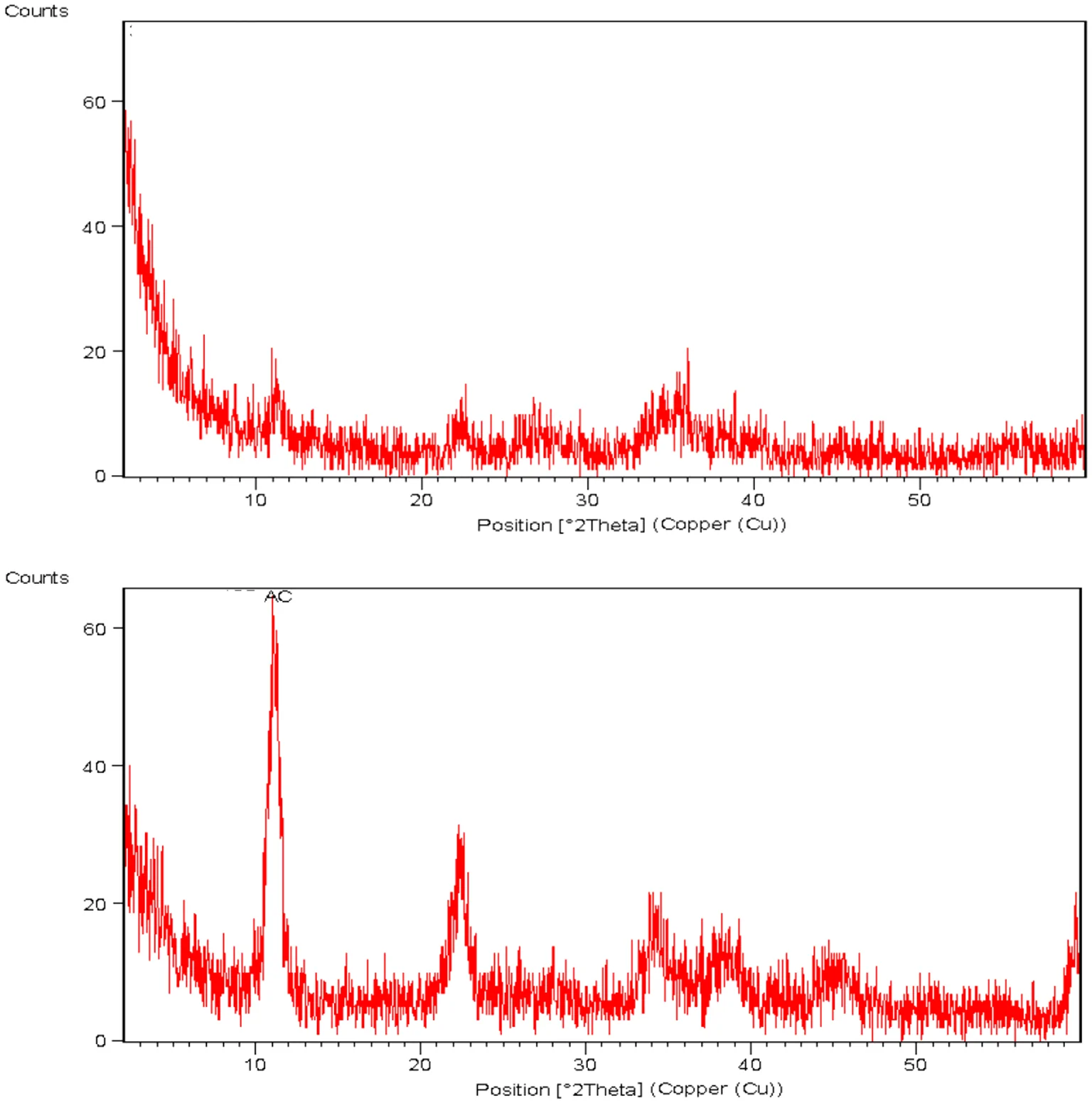
XRD pattern of (**a**) LDH and (**b**) LDH–AC.

The X-Ray spectra of the synthesized LDH and LDH-AC are shown in Fig. 3. The peak positions (*2θ*) at 11.75°, 23.44°, and 34.43° corresponded to the (0 0 3), (0 0 6), and (0 0 9) planes of LDHs, with d values of 7.52, 3.79, and 2.60, respectively. These were characteristic peaks of crystalline LDHs^37^, as shown in Fig. 3(a). Figure 3(b) shows the X-ray spectra of the LDH-AC composite. The typical reflections of LDH were amplified, indicating the production of a mixture of pure LDH and intercalated LDH. This shows that the AC-intercalated LDH structure has formed.

BET analysis revealed specific surface areas of 23.49 m²/g for LDH and 19.81 m²/g for LDH–AC. The decrease in the surface area is attributed to the impregnation of activated carbon particles into the interlayer galleries and onto the external surfaces of LDH, causing partial pore blockage and aggregation that reduces accessible microporosity. This confirms the results obtained above from SEM. Such behavior is consistent with LDH composite formation, where high surface area of AC integrates but does not fully dominate due to intimate LDH-AC interactions, including hydrogen bonding and van der Waals forces that smoothen the composite texture. Despite the lower BET area of LDH–AC, it exhibited markedly superior methyl orange sorption capacity, suggesting compensatory mechanisms: enhanced active sites from functional groups of AC (such as –OH, –COOH), improved hydrophobicity aiding dye diffusion.

**Fig. 4:**
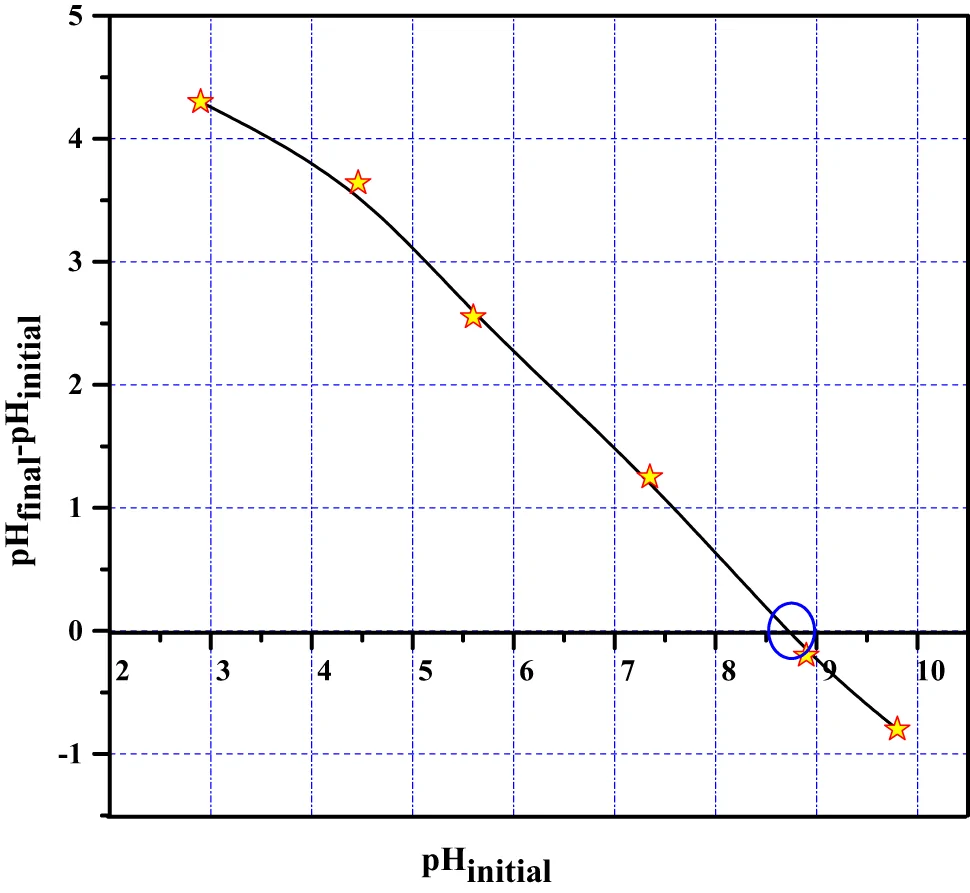
Studying the point of zero charge of LDH–AC.

The point of zero charge (pH_PZC_) is the pH at which the LDH–AC surface charge is neutral; -ve charges exist above this point, while +ve charges occur beyond. A set of 50 mL bottles with 0.1 g of LDH–AC and 10 mL of an electrolyte (KNO_3_) at 0.1 mol/L were used for the computation. The original pH of the bottles ranged from 2 to 10, and they were agitated for a full day. The final pH (pH_final_) was measured in each case after the centrifugation. Plotting initial pH (pH_initial_) against ∆pH (pH_final_-pH_initial_), as shown in Fig. 4. The pH_PZC_ was found to be 8.75. As a result, the LDH–AC surface becomes positive charged at pH values lower than 8.75, which increases its capacity to adsorb anions (MO) throughout a wide pH range.

### Sorption experiments

#### Effect of pH

**Fig. 5:**
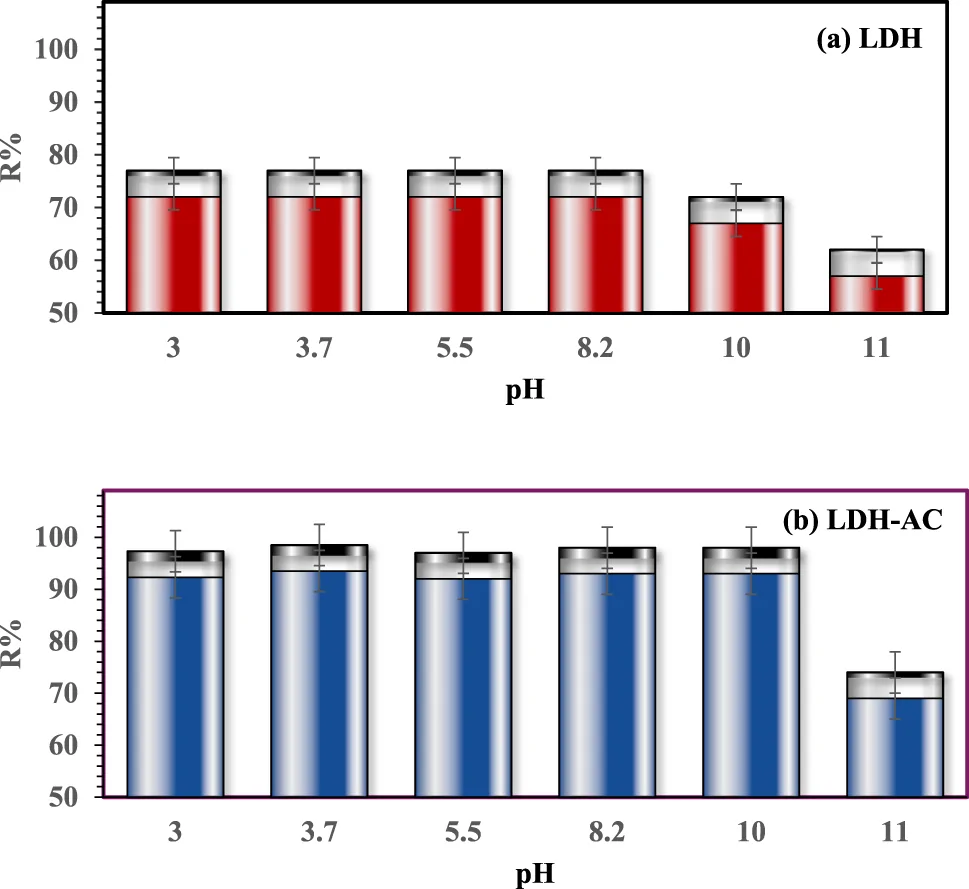
Effect of pH on sorption of 100 mg/L of MO by 0.03 g of (**a**) LDH and (**b**) LDH–AC, at 298 K and equilibrium time of 60 min.

Figure 5 shows that both LDH and LDH–AC sorb methyl orange less as pH increases. This could be because of the surface charge. The protonation process on the sorbent materials’ surfaces has left many positively charged binding sites on both materials^38^. Sorption occurs due to electrostatic attraction between the anion and the positive charge on the surfaces of both materials^39^. As the pH increased, the negative charge density on the sorbet surface increased, reducing the electrostatic attraction force. This made it harder for the negatively charged MO ions to stick to the sorbet. The optimal pH for MO sorption is 5.5, yielding a high R% and a moderate pH. The *R%* value for LDH–AC (92%) is also higher than that for LDH (72%), indicating that the modification method improved sorption efficiency.

#### Effect of sorbent mass

**Fig. 6:**
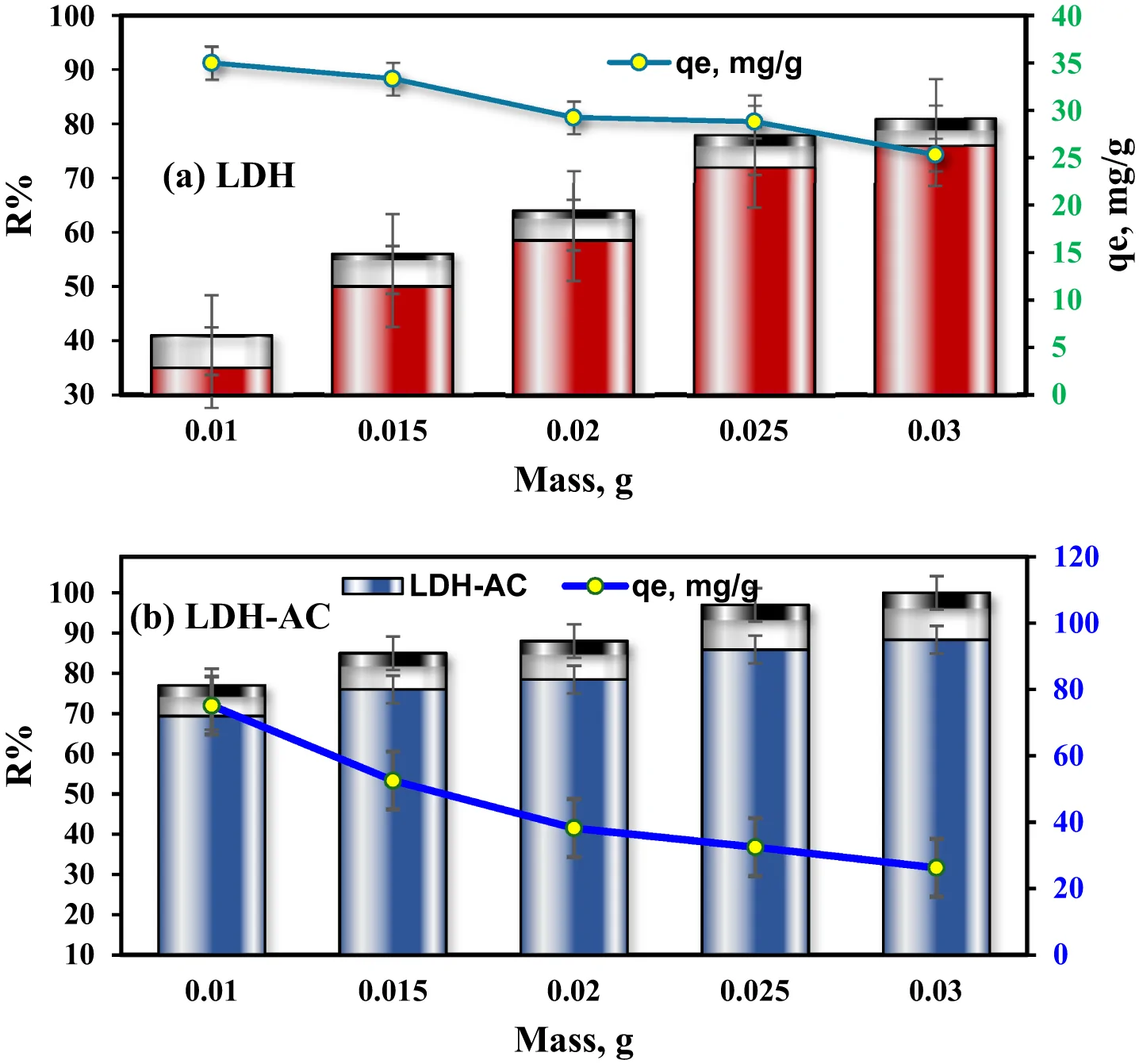
Effect of sorbent mass on sorption of 100 mg/L MO by (**a**) LDH and (**b**) LDH–AC at 298 K, pH 5.5 and 60 min equilibrium time.

The sorbent dose is an important parameter in sorption studies because it indicates how much of a given initial ion concentration the sorbent can retain. It is also important to look into the effect of sorbent mass to find the best sorbent mass by balancing the sorbent capacity and the *R%* of MO^**40**^. Figure 6 shows how the sorbent mass, ranging from 0.01 to 0.03 g, affects the results. As the LDH and LDH–AC mass went up from 0.01 g to 0.03 g, the *R%* of MO went up from 35% to 76% for LDH and from 72% to 95% for LDH–AC. When the amount of sorbent was raised from 0.01 to 0.03 g, the amount of sorbed (*q*_*e*_) dropped from 35 to 25.3 mg/g for LDH and from 72 to 31.7 mg/g for LDH–AC. This is because there are more places and surfaces for the sorption to happen when the mass is greater. The fact that the sites remain unsaturated during sorption is what causes the amount sorbed to decrease ^40,41^. To determine the optimal amount of sorbent, you need to perform a trade-off analysis between sorption capacity and *R%*. The best amount of ion removal and sorbed mass is 0.03 g for both types of sorbents.

#### Effect of shaking time

**Fig. 7:**
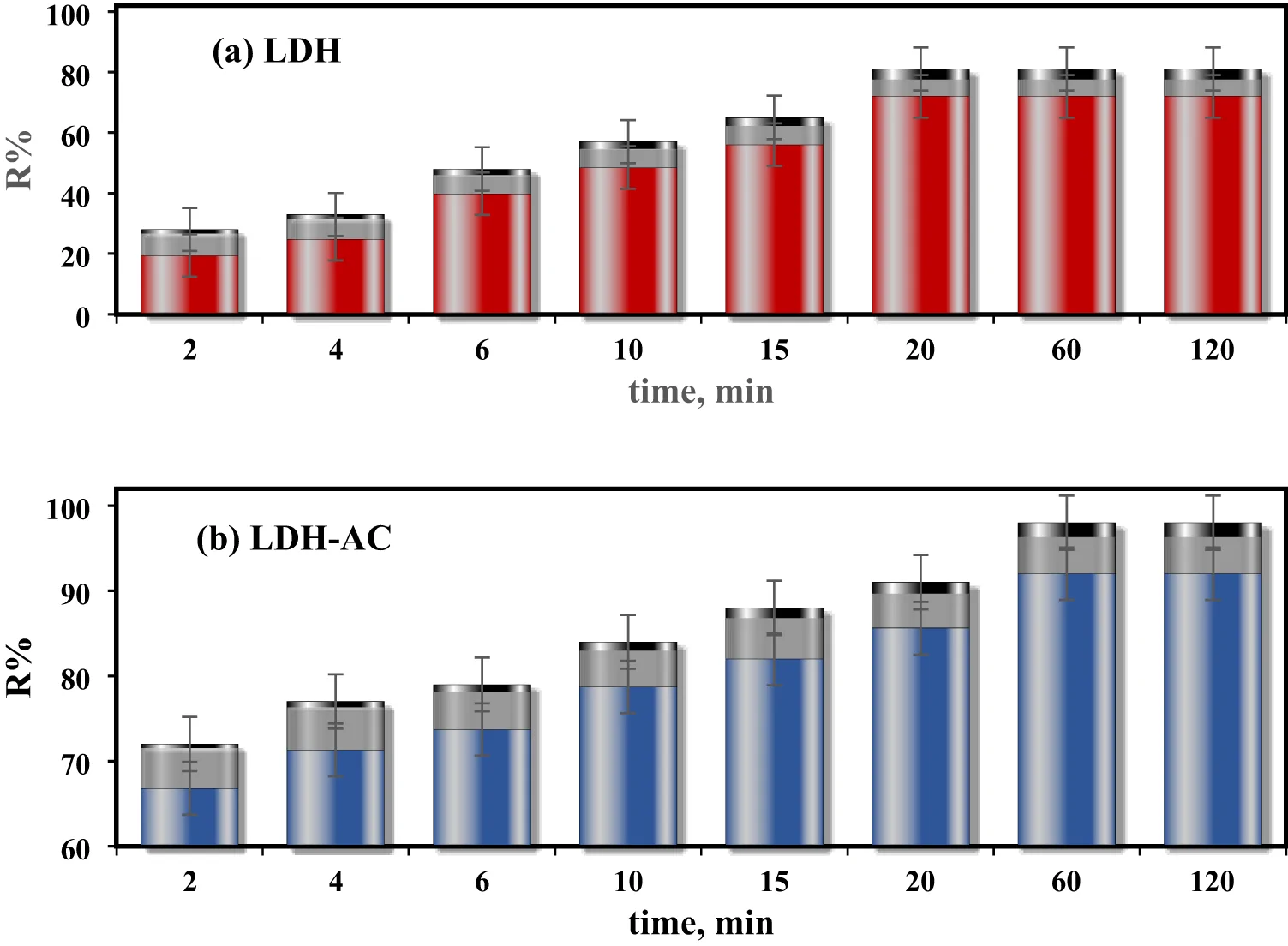
Effect of shaking time on sorption of 100 mg/L MO by 0.03 g of (**a**) LDH and (**b**) LDH–AC at 298 K and pH 5.5.

The shaking duration was analyzed for the sorption of methyl orange by both LDH and LDH–AC at pH 5.5 over 2-120 min. Figure 7 shows that the clearing percentage increased with increasing shaking period. It reached its maximum at the equilibrium time, and after that, there was almost no further sorption. Figure 7(a) shows that the equilibrium time for MO is reached after 20 min for LDH. Figure 7(b) shows that the equilibrium time for LDH–AC is increased to 60 min. However, since the *R%* values for LDH–AC are higher than those for unmodified LDH, the modification method improves sorption.

##### Pseudo–first–order model

**Fig. 8:**
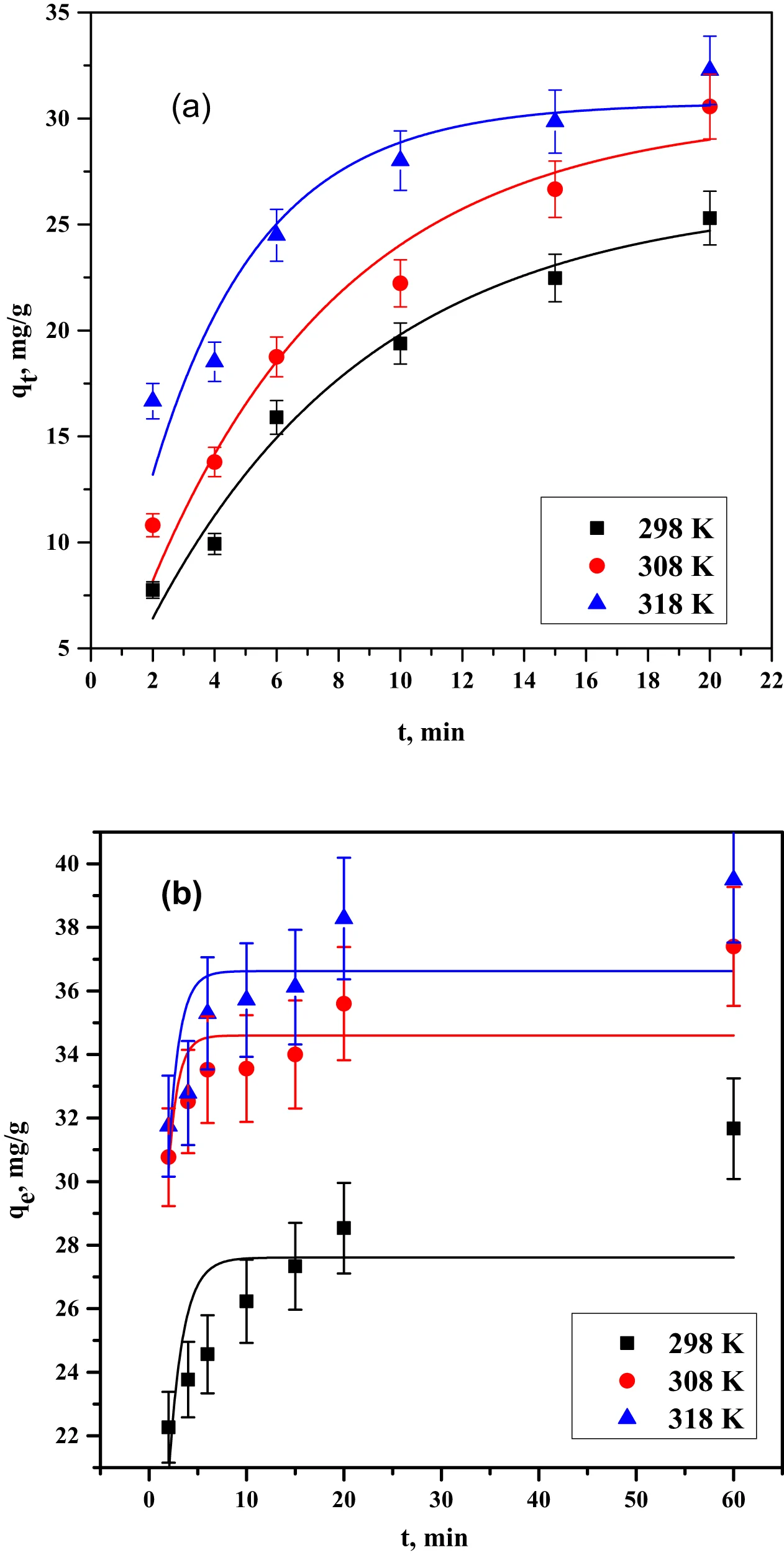
Pseudo-first-order for sorption of 100 mg/L MO by 0.03 g of (**a**) LDH and (**b**) LDH–AC at different temperatures and pH 5.5.

Kinetic models can provide information on how sorption occurs and which mechanisms may be involved. They are also very important for designing the sorption system and developing the process. Lagergren^42^ put forward the pseudo-first-order kinetic model (PFO). The Lagergren rate equation describes the sorption rate from aqueous solution and can be expressed as follows.

4 $$\frac{{d{q_t}}}{{dt}}={k_n}{\left( {{q_e} - {q_t}} \right)^n}$$

where *q*_*e*_ and *q*_*t*_ are the amounts of sorbed material at equilibrium and time *t* (mg/g), respectively. The rate constant is identified by k_n_ and n is the sorption reaction order. If n equals 1, integrated form of Eq. (1) for the boundary conditions q_t_ = 0 when t = 0, will give the PFO formula as follows.

5 $${q_t}={q_e} \left( {1 - e^{ - {k_1}t}} \right)$$

k_1_ (min^− 1)^ is the rate constant for PFO theory. It is usually noted that kinetics follows Lagergren equation when the sorption performs over diffusion in the interface^43,44^. Applying the nonlinear fitting by plotting of *q*_*t*_ vs. *t* is explored in Fig. 8. Table 1 reported the computed values of *k*_*1*_ and *q*_*e*_ along with the regression coefficient, *R*^*2*^, of the PFO. Despite the computed *q*_*e*_ values are near to the experimental values, the *R*^*2*^ values are very low for both materials; hence, this model does not capture how MO was adsorbed by either LDH or the LDH–AC composite.

**Table 1:** Calculated parameters of PFO, PSO, and intra-particle diffusion models for sorption of MO onto LDH and LDH–AC.

Sorbent	Temp, K	PFO				PSO			Intra-particle		
		q_e, exp_, mg/g	q_e, calc_, mg/g	k_1_, min^− 1^	R^2^	k_2_, g/mg.min	q_e, calc_. mg/g	R^2^	k_ad_, mg/g.min^− 1/2^	C, mg/g	R^2^
LDH	298	25.3	26.31	0.139	0.771	0.065	25.58	0.967	6.338	1.305	0.960
	308	30.56	27.99	0.157	0.741	0.0051	30.84	0.980	6.656	1.940	0.990
	318	32.27	22.02	0.280	0.867	0.0102	33.21	0.990	5.743	8.656	0.933
LDH–AC	298	31.70	27.61	0.692	0.346	0.0573	30.71	0.995	2.458	23.440	0.992
	308	37.01	34.59	1.05	0.384	0.254	37.00	0.935	1.127	29.929	0.779
	318	38.28	36.62	0.913	0.397	0.670	39.98	0.930	2.636	27.996	0.902

#### Pseudo–second–order model

The pseudo–second–order (PSO) model posits that the rate-determining phase of the process may involve chemisorption^44^, necessitating an electron valency force or electron exchange between the sorbent and the sorbate and probably the formation of a new compound^41^. From Eq. (1), PSO can be obtained if *n* equals 2,. The differential equation of the model is formulated as:

6 $$\frac{{d{q_t}}}{{dt}}={k_2}{\left( {{q_e} - {q_t}} \right)^2}$$

*k*_*2*_ (g/mg min^− 1^) denotes the PSO rate constant for the sorption process. Integrating and applying boundary conditions *q*_*t*_=0 when *t* = 0 and *q*_*t*_=*q*_*t*_ when *t* = *t*, Eq. (6) is changed to be as follows

7 $${q_t}=\frac{{{k_2}q_{e}^{2}t}}{{1+{k_2}{q_e}t}}$$

**Fig. 9:**
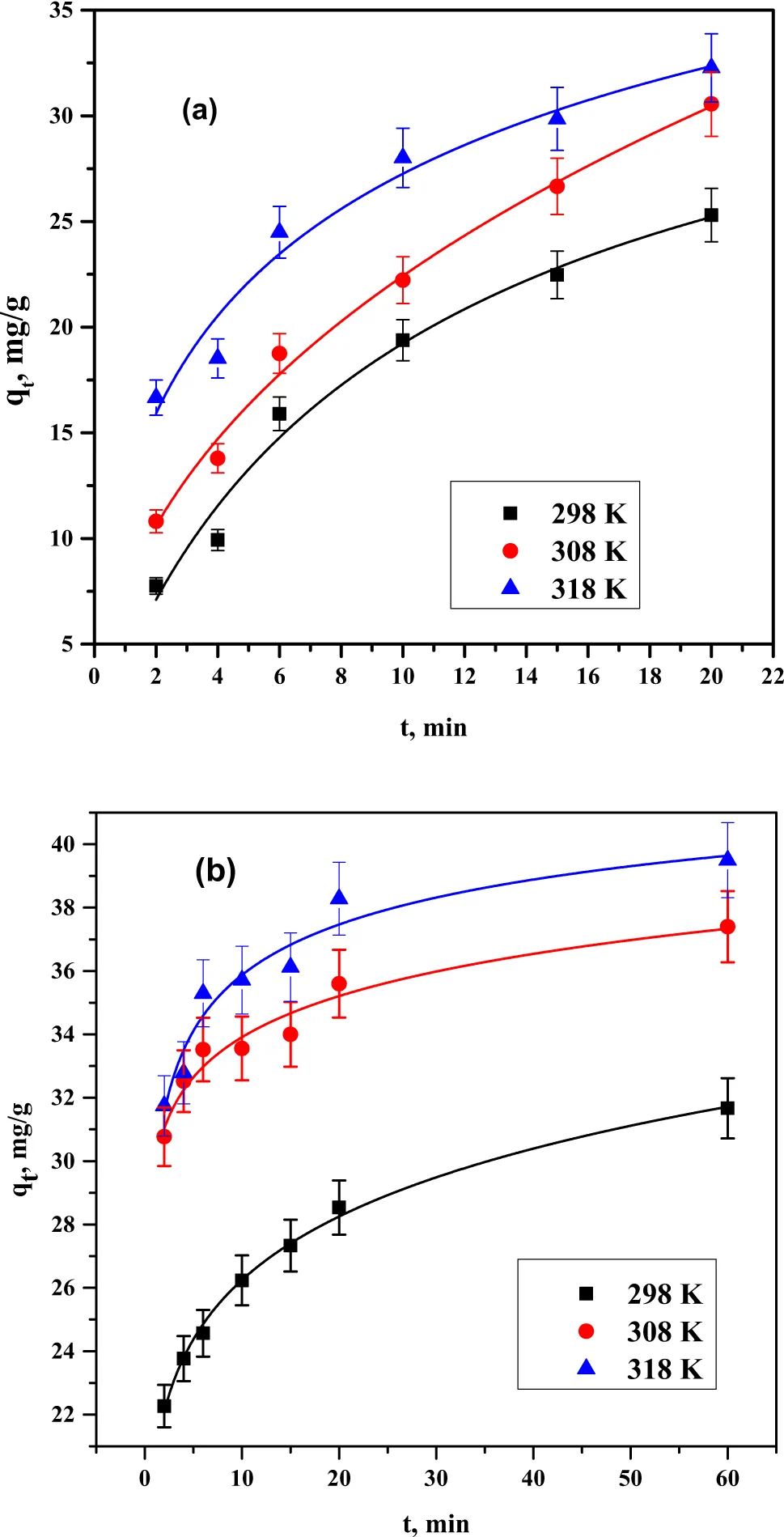
Pseudo–second–order plot for sorption of 100 mg/L MO by 0.03 g of (**a**) LDH and (**b**) LDH–AC at different temperatures and pH 5.5.

*k*_*2*_ and the calculated amount sorbed, *q*_*e, calc*_, can be gained from the nonlinear plot of *q*_*t*_ vs. *t*, Fig. 9. The data demonstrated that *R*^*2*^ values of PSO are higher than that of PFO, Table 1. Moreover, the computed values of *q*_*e, calc*_ from the PSO are more close to the *q*_*e, exp*_. Hence, the PSO is more likely to determine the sorption over the whole range of time; it also indicates the chemisorption process. So, the PSO is better fit the data than the PFO on the sorption of MO by the prepared material.

##### Intra-particle diffusion model

The examination of Mores and Weber’s theory revealed that film diffusion exerts negligible influence during sorption; hence, intra-particle diffusion governs the process’s rate^45^. Mores and Weber developed an equation based on their own ideas.

8 $$q_t = k_{ad} t^{1/2} + C$$

**Fig. 10:**
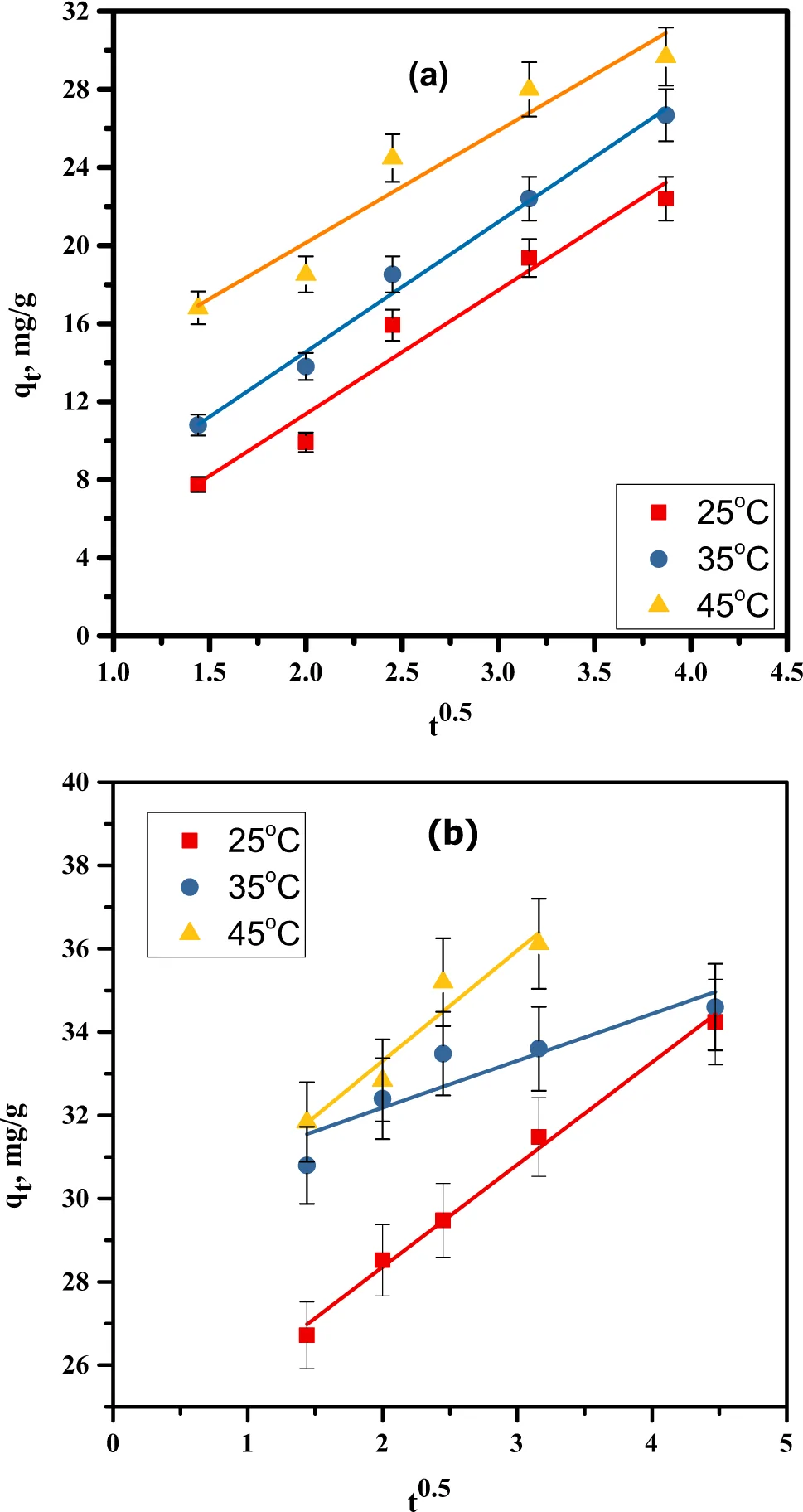
Plotting of Intra-particle diffusion model for sorption of 100 mg/L MO by 0.03 g of (**a**) LDH and (**b**) LDH–AC at different temperatures and pH 5.5.

The rate constant for intra-particle diffusion is *k*_*ad*_ (mg g^− 1^ min^− 1/2^), and the constant *C* is directly related to boundary films. An elevated *C* value signifies enhanced boundary films. We examined how the amount of sorbed material changed over time (*q*_*t*_ vs. *t½*) to understand better the distinct stages of mass transfer in Fig. 10. The boundary-film diffusion stage occurred quickly because it didn’t affect the reaction rate; therefore, the researchers omitted it from the figure. The next phase, on the other hand, was the slowest in the gradual sorption process.

The sorption of MO onto LDH and LDH–AC transpired via film and intra-particle diffusion, with intra-particle diffusion emerging as the predominant mechanism governing MO sorption behavior. The sorbate moves by going from the bulk solution into the pores of the sorbent. Table 1 shows the values of *C*. Intra-particle diffusion is a major rate-influencing step (multi-linear Weber-Morris plots (high values of R^2^), but non-zero intercepts indicate concomitant film/boundary layer diffusion. This hybrid diffusion control aligns with the enhanced capacity of LDH–AC despite reduced BET area, as surface chemistry accelerates boundary layer penetration. The film diffusion is a sharp increase (fast step) while the intra-particle diffusion is the slowest step so, the intra-particle diffusion was selected as the rate determining step.

#### Equilibrium isotherm studies

Different theoretical models, such as Langmuir, Freundlich, and D-R, can be used to examine both sorption capacity and the processes involved in MO sorption by LDH and LDH–AC.

##### Langmuir isotherm

**Fig. 11:**
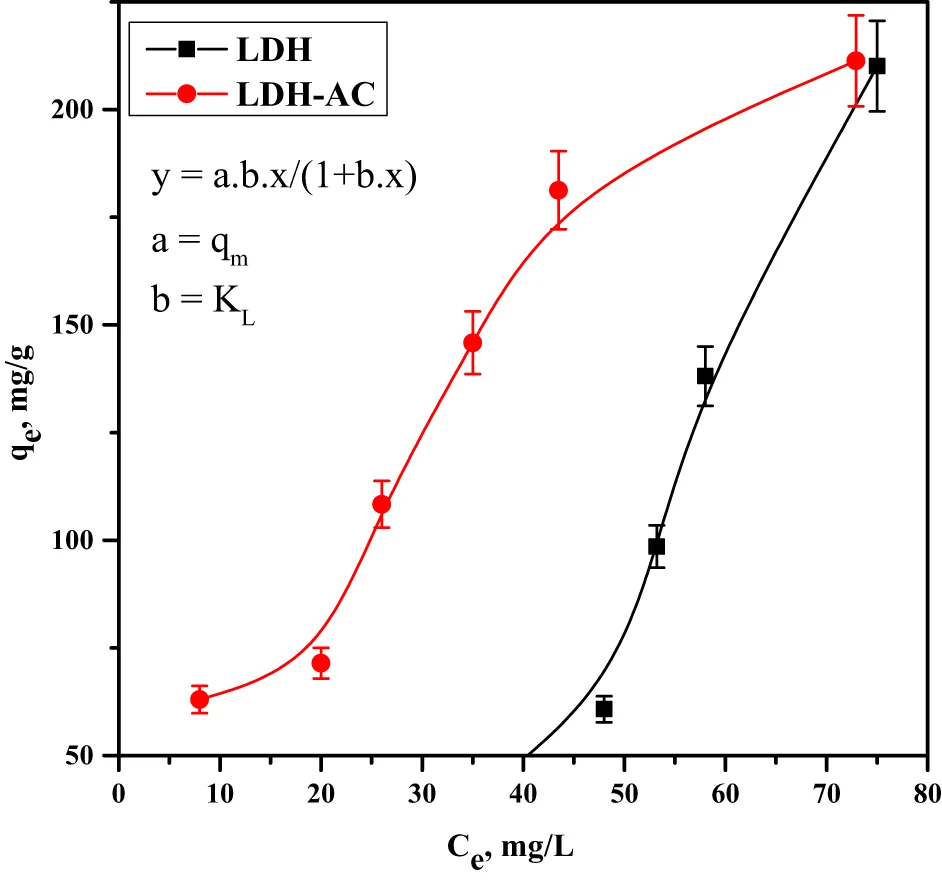
Langmuir plot for sorption of MO by 0.03 g of LDH and LDH–AC at 298 K and pH 5.5.

Different sorbents use the Langmuir model because it was first used for systems that adsorb gases and solids, it was modified to fit the adsorption isotherm of liquid on solid surfaces^46^. The model demonstrated that both adsorption and desorption terminate at the surface, as kinetic principles govern equilibrium^47^. To assess sorption capacity, different concentrations of MO solutions were agitated with LDH and/or LDH–AC for a specified equilibrium duration. The nonlinear form of Langmuir theory is identified as follows.

9 $$q_e = q_m \frac{K_L C_e}{1+ K_L C_e}$$

where *q*_*m*_ gives the max sorption capacity, mg/g, and *K*_*L*_ in L/mg is the sorption affinity constant. Figure 11 illustrates fitting of the experimental results to the sorption equation by nonlinear regression fitting (*q*_*e*_ vs. *C*_*e*_). Table 2 reports values of Langmuir constants; the *q*_*m*_ of MO ions sorbed by LDH and LDH–AC are 71.43 and 393.70 mg/g at room temperature, respectively. This suggests that modifying LDH improved its sorption capacity. Equation (10) was used to figure out the *R*_*L*_ values for both sorbents. They are between 0 and 1, indicating good sorption. In particular, the *R*_*L*_ values for LDH (0.493) and LDH–AC (0.383) suggest that the sorption process worked well at the examined doses.

10 $$R_L =\frac{1}{1+bC_o}$$

**Table 2:** Parameters of different equilibrium isotherm models for sorption of MO by LDH and LDH–AC.

Model	Model parameters	Sorbent	
		LDH	LDH–AC
Langmuir	*q*_*m*,_ *(mg/g)* *b (L/g)* *R*_*L*_ *R* ^*2*^	71.43 0.01 0.493 0.872	393.70 0.02 0.383 0.650
Freundlich	*1/n* *K*_*f*_ *(mg/g)(L/mg)*^*1/n*^ *R* ^*2*^	2.13 0.02 0.927	0.83 6.67 0.963
D-R	*q*_*m*_ *(mmol/g)* *β (mol* ^*2*^ */kJ* ^*2*^ *)* *E* *(kJ/ mol)* *R* ^*2*^	0.193 7.1 × 10^− 2^ 2.65 0.937	0.651 2.9 × 10^− 2^ 4.16 0.936

##### Freundlich isotherm theory

The sorption was also described using the Freundlich isotherm, which accounts for sorption on surfaces that are not uniform. This theory is often used in places where multilayer sorption and surfaces with varying affinities happen. Freundlich theory is non-linearly formulated by Eq. (6).

11 $${q_e}={K_F}C_{e}^{{{\raise0.7ex\hbox{$1$} \!\mathord{\left/ {\vphantom {1 n}}\right.\kern-0pt}\!\lower0.7ex\hbox{$n$}}}}$$

**Fig. 12:**
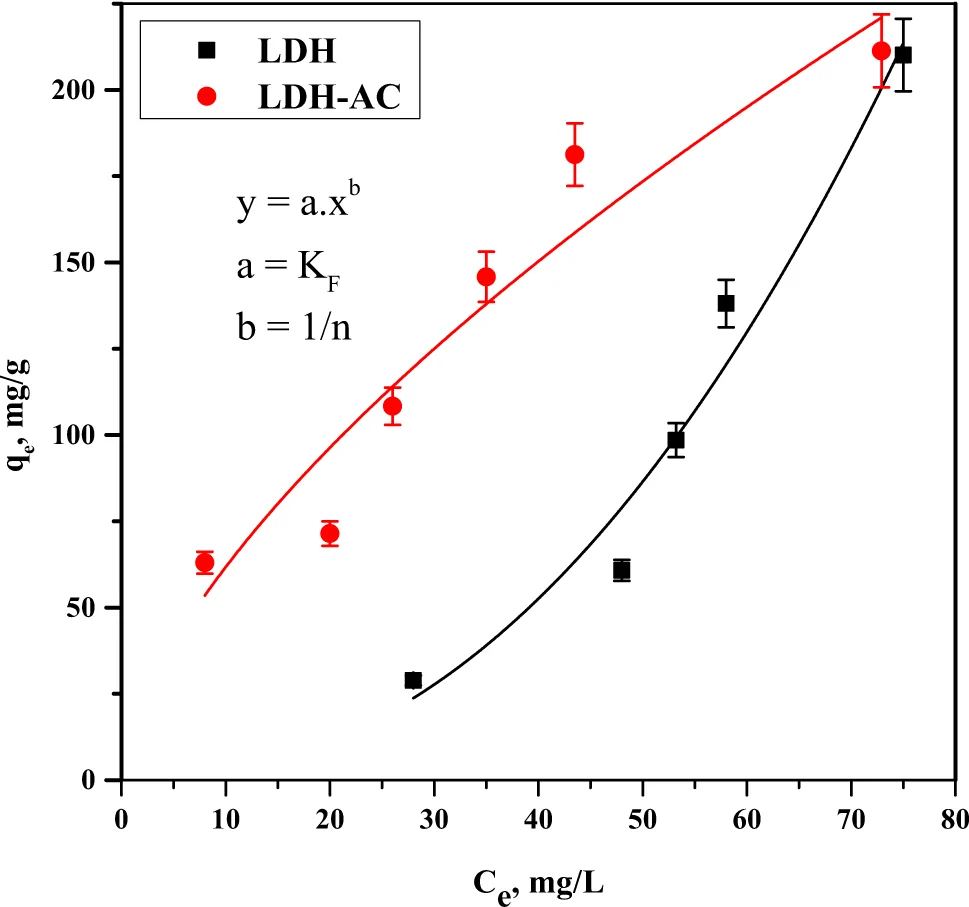
Freundlich non-linear plot for sorption of MO by 0.03 g of LDH and LDH–AC at 298 K and pH 5.5.

Identifying Freundlich constants using *n* and *k*. The constant *k* shows how much the sorbent can hold, whereas the value of *1/n* gives how strong and sufficient the sorbent/sorbate system is. Figure 12 explores the non-linear plot of *q*_*e*_ against *C*_*e*_ and the values of Freundlich parameters are reported in Table 2. Both LDH and LDH–AC exhibit concentration-dependent MO sorption, as indicated by 1/n values less than 1. The table shows that the *R*^*2*^ values are higher than those predicted by Langmuir theory (*R*^*2*^ > 0.9). So, the Freundlich isotherm is better for MO sorption by LDH and LDH–AC.

##### Dubin–Radushkevich (D–R) isotherm

The Dubinin–Radushkevich theory posits that the porous structure of the sorbent influences the shape of the sorption curve. The sorption data were analyzed using the D–R theory to distinguish between chemical and physical adsorption^48^. It also specifies the sorption type as ion-exchange or physical adsorption. It is relevant at low concentrations and can be used to characterize sorption on both homogeneous and heterogeneous surfaces. Sorption is characterized by a singular type of uniform pore. The D–R isotherm is analogous to the Langmuir type but is more general, as it does not presume a homogeneous surface or a constant sorption potential. The non-linear form of D-R formula is expressed by the subsequent equation.

12 $${q_e}={q_m}{e^{ - \beta {{\left( {RT\ln \left( {1+\frac{1}{{{C_e}}}} \right)} \right)}^2}}}$$

where *q*_m_ (mol/g) is the maximum quantity of MO that can be sorbed onto unit weight of the sorbent, and *β* is the activity coefficient related to mean sorption energy. Polanyi potential, *ε*, is expressed in (kJ/mol)^2^, is specified by the following equation.

13 $$\epsilon = RT In \left( 1+\frac{1}{C_e}\right )$$

where *R* is the general gas constant (0.008314 kJ/mol K.), and *T is* the absolute temperature in kelvin (K).

**Fig. 13:**
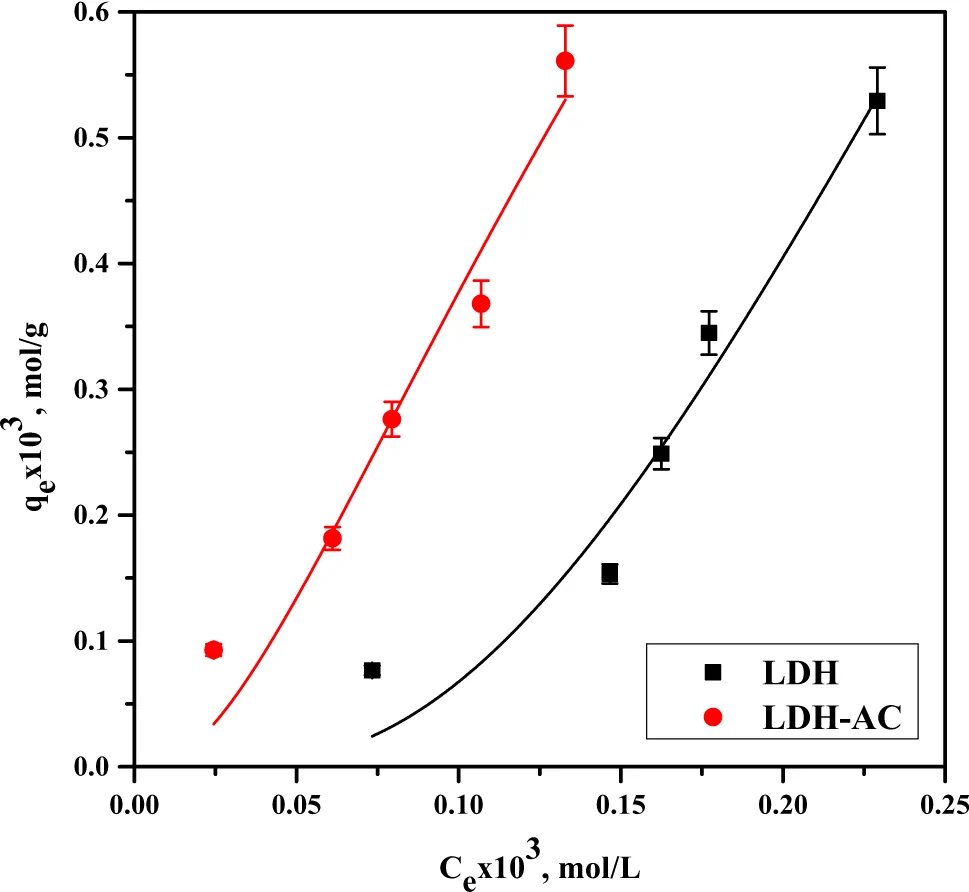
D–R isotherm non-linear plot for sorption of MO by 0.03 g of LDH and/or LDH–AC at 298 K and pH 5.5.

Plotting the non-linear form of D–R is seen in Fig. 13. Table 2 shows how to find the values of *β* (mol^2^/kJ^2^) and *q*_*m*_ (mmol/g). The value of *β* is connected to the sorption mean free energy, *E* (kJ/mol), which you can find using Eq. 14 and identified as the change in free energy required to move one mole of sorbate from the solution’s infinity to the sorbent surface.

14 $$E=\frac{1}{{\sqrt {2\beta } }}$$

The sorption mechanism, such as ion-exchange or physical adsorption, can be inferred from the mean free energy of sorption, *E*. Ion-exchange occurs during the sorption process if the *E* value is between 8 and 16 kJ/mol, whereas physical sorption occurs if the *E* value is less than 8 kJ/mol^**48**^. The findings demonstrated the physical character of the sorption process.

#### Thermodynamic studies

The thermodynamic parameters (∆G^o^, ∆H^o^, and ∆S^o^) of sorption are important for determining whether a process is endothermic or exothermic, as well as the spontaneity of MO sorption. The thermodynamic coefficients are calculated using the Vant Hoff equation.

15 $$\Delta {G^o} = - RT \ln {K_d}$$

where *K*_*d*_ denotes the distribution coefficient, L/g, and *∆G*^*o*^ is the Gibbs free energy change. This is one way to depict the shift in free energy.

16 $$\Delta {G^o} = - \Delta H^o \cdot T \Delta S^o$$

The thermodynamic formula is used to compute *ln K*_*d*_ by rearranging and substituting the value of *∆G*_*o*_ from Eq. (15) in Eq. (14) as follows.

17 $$In K_d = \frac{\Delta S^o }{R}- \frac{\Delta H^o}{R} \frac{1}{T}$$

**Fig. 14:**
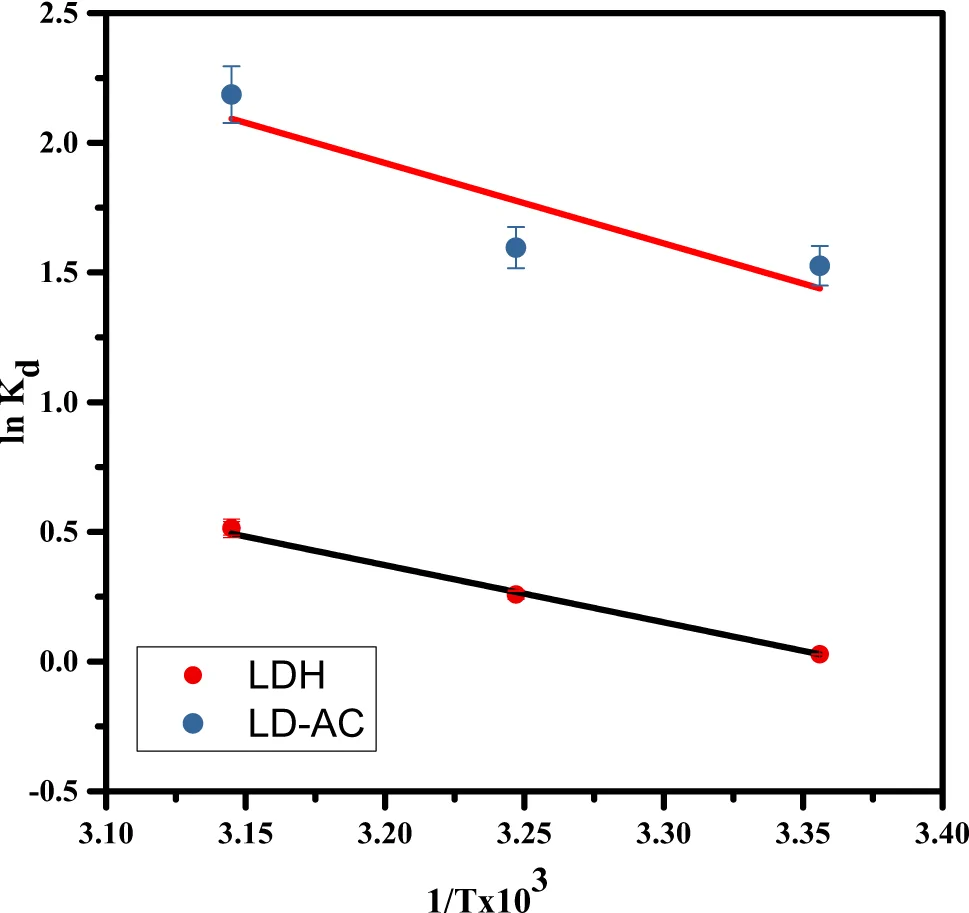
Thermodynamics for sorption of MO by 0.03 g of (a) LDH and (b) LDH–AC at pH 5.5.

Straight lines were obtained from the *ln Kd* & *1/T* plot, as explored in Fig. 14. Entropy (*ΔS*^*o*^) and enthalpy changes (*ΔH*^*o*^) can be calculated using the slope and intercept. Because both LDH and LDH–AC have positive *ΔH*^*o*^ values, Table 3 indicates that sorption is endothermic. Furthermore, the sorption mechanism is chemisorption when *ΔH*^*o*^ values for LDH–AC are higher than 20 kJ/mol. Because of structural changes in both the sorbent and the sorbate, as well as the sorbents’ affinity for MO, as seen by the positive values of *ΔS*^*o*^, the solid/solution interface provides increased unpredictability. The obtained negative values of *∆G*^*o*^ verify the spontaneous nature and viability of the sorption processes.

**Table 3:** Thermodynamic parameters for sorption of MO dye onto LDH and LDH–AC.

T (K)	ΔG^o^(kJ/mol)		ΔH^o^ (kJ/mol)		ΔS^o^ (J/mole/K)	
	LDH	LDH–AC	LDH	LDH–AC	LDH	LDHAC
298	-0.069	-3.78	18.54	35.77	64.38	98.44
308	-0.661	-4.09				
318	-1.359	-5.77				

### Proposed sorption mechanism

**Scheme 1:**
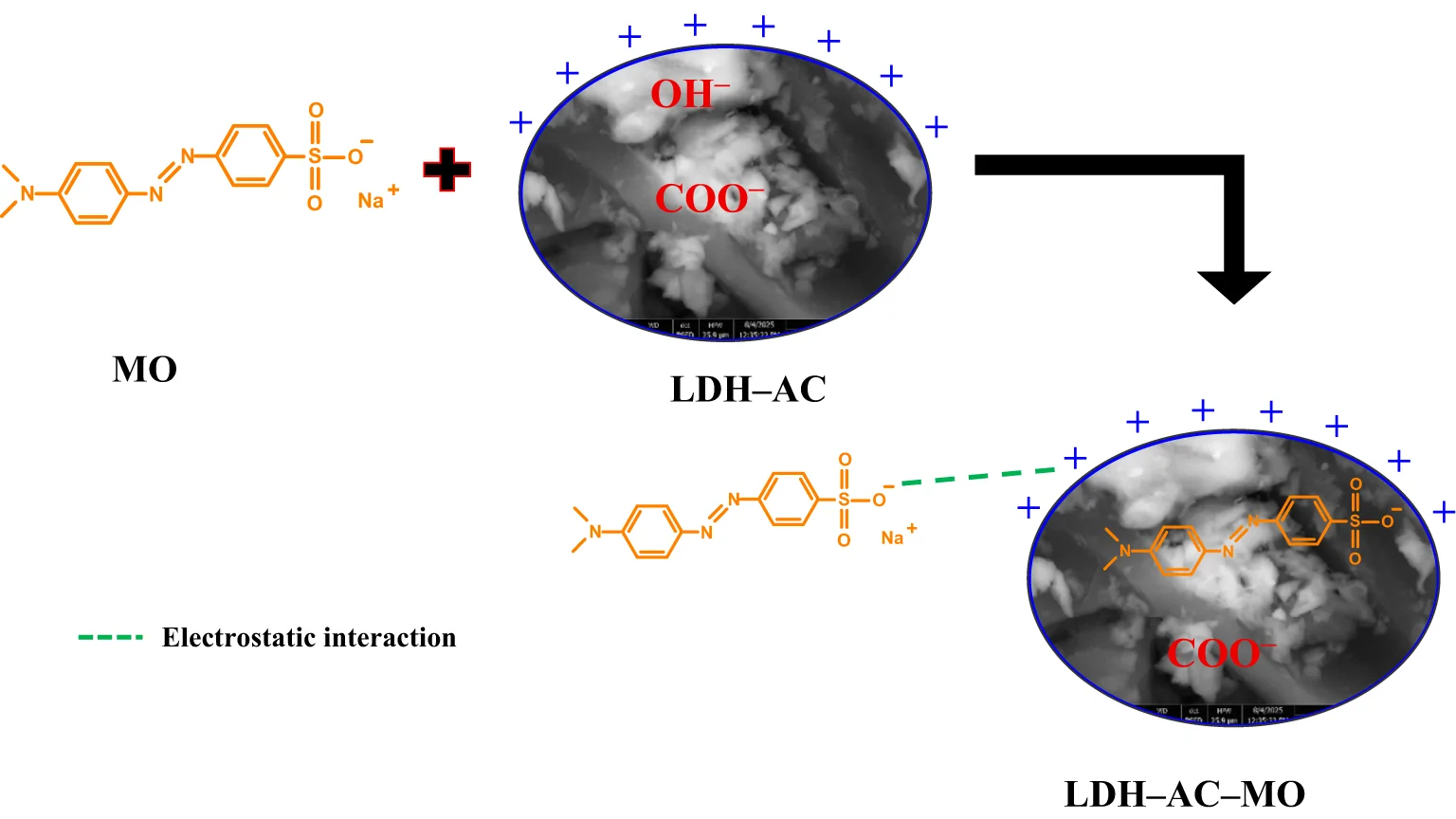
Proposed sorption mechanism of MO by LDH–AC at 298 K and pH 5.5

The molecular structure of the dye, the sorbent’s surface properties, and the conditions of the sorption medium all have impacts on the sorption mechanism of organic dye. Dye sorption can be controlled by a different mechanism, including hydrophobic interaction, hydrogen bonding, and electrostatic attraction, depending on these factors. It is known that the LDH is characterized by the presence of positive charge on the surface, and a negatively charged anion-exchange layer (OH^–^) in the interlayer area, as mentioned above in the FTIR section. This simplifies the adsorption of anions by ion exchange process such as methyl orange which has a negative charge due to the presence of sulfonate group as illustrated by Scheme 1. In addition, the activated carbon contains negatively charged carboxylate groups (–COO^–^) which increase the sorption capacity. Based on the experimental findings and considering the molecular structures of MO as well as the surface properties of the LDH–AC, the sorption mechanism maybe also proceed via electrostatic attraction between the negative charge of the dye and the positively charged surface of LDH–AC, as illustrated in Scheme 1. This confirms the suggested mechanism gained from D–R isotherm model which told us that the sorption mechanism may be physical process according to the value of mean free energy. Therefore, MO removal by LDH–AC involves a multi-mechanism process.

### Desorption study

The desorption of methyl orange ions from LDH–AC sorbent was carried out employing both ethyl alcohol and different molarities of sodium hydroxide, ranged from 0.1 to 1.0 mol/L at 298 K for 3 h. The desorption percentages, *D%*, of MO were computed by measuring the values of MO concentration in the aqueous phase, *C*_*a*_, and in the solid, *C*_*s*_, as illustrated in Eq. (17). The results showed that the value of *D%* was found to be more than 95% using 0.5 mol/L NaOH. This indicates that LDH–AC is capable of regeneration and reuse.

18 $$D\% =\frac{{{C_a}}}{{{C_s}}} \times 100$$

### Comparison of adsorbents

The results of a comparison of MO’s sorption capacity with modified LDH and other sorbents used in the literature are reported in Table 4. LDH-AC’s strong sorption capacity is demonstrated by its highest sorption capacity compared to previous materials.

**Table 4:** Comparison of the prepared LDH and LDH–AC with the literature-reported sorbents to remove MO-dye[49-56].

Sorbent	pH	T, K	Q_max_, mg/g	Reference
Activated carbon	2.0	298	79.7	[49]
Cellulose fiber	2.0	298	50.76	[50]
Fe_3_O_4_/chitosan	3.0	298	1104.1	[51]
Chitosan/PVA/zeolite nanofiber	3.0	298	153.0	[52]
Amino-ethyl carboxymethyl cellulose	7.0	298	2.03	[53]
Amino-ethyl Chitosan crosslinked hydrogel	7.0	298	10.53	[54]
Ni(II) doped magnetic Fe_3_O_4_@mesoporous SiO_2_/UiO-66	4.0	303	182.8	[55]
chitosan–GA–cellulose composite	4.4	298	324.62	[56]
LDH LDH–AC	5.5 5.5	298 298	71.43 393.70	This work This work

## Conclusion

Layered double hydroxide was modified with activated carbon to produce LDH–AC, a composite sorbent evaluated for methyl orange removal from aqueous solutions under controlled laboratory conditions. Comprehensive characterization (FTIR, SEM, XRD, and BET) confirmed structural integrity and modification success. The modification increased sorption capacity from 71.43 mg/g (LDH) to 393.73 mg/g (LDH–AC) at 298 K and pH 5.5, as determined by Langmuir isotherm modeling. Sorption followed pseudo-second-order kinetics, with thermodynamic parameters indicating an endothermic and spontaneous process under tested conditions. Compared with other materials reported in the literature, the modified LDH showed a higher sorption capacity. To remove MO from aqueous solutions, it is therefore recommended to use modified LDH as a low-cost and environmentally friendly sorbent.

Limitations of the work: Despite the promising results, the limitations must be acknowledged to contextualize the findings and guide future research. Sample sizes and experimental scales were relatively small (lab scale), focusing on batch kinetic and isotherm studies rather than continuous-flow column experiments, which are more representative of pilot scale applications. Consequently, parameters like bed depth, flow rate, and breakthrough curves are critical for scale-up. Future work should address these through pilot-scale trials and multi-pollutant matrices.

## Supplementary Information

Below is the link to the electronic supplementary material.

Supplementary Material 1

## Data Availability

All data generated or analysed during this study are included in this published article [and its supplementary information files].

## Declarations

### Data Availabity

All data generated or analysed during this study are included in this published article [and its supplementary information files].

### Statement on the Use of Generative AI

Grammarly and ChatGPT were employed exclusively as language assistance tools to paraphrase and improve the clarity of the manuscript, without affecting the originality, scientific content, or data interpretation.
