# Supplementary material for: Mitigating environmental pollution by methyl orange via effective adsorption using modified layered double hydroxide
Source: Sci Rep. 2026 Aug 20;16:26065. doi: 10.1038/s41598-026-52336-7 (PMC13490572; doi:10.1038/s41598-026-52336-7)
Supplement: Supplementary file 1 [file 41598_2026_52336_MOESM1_ESM.pdf]

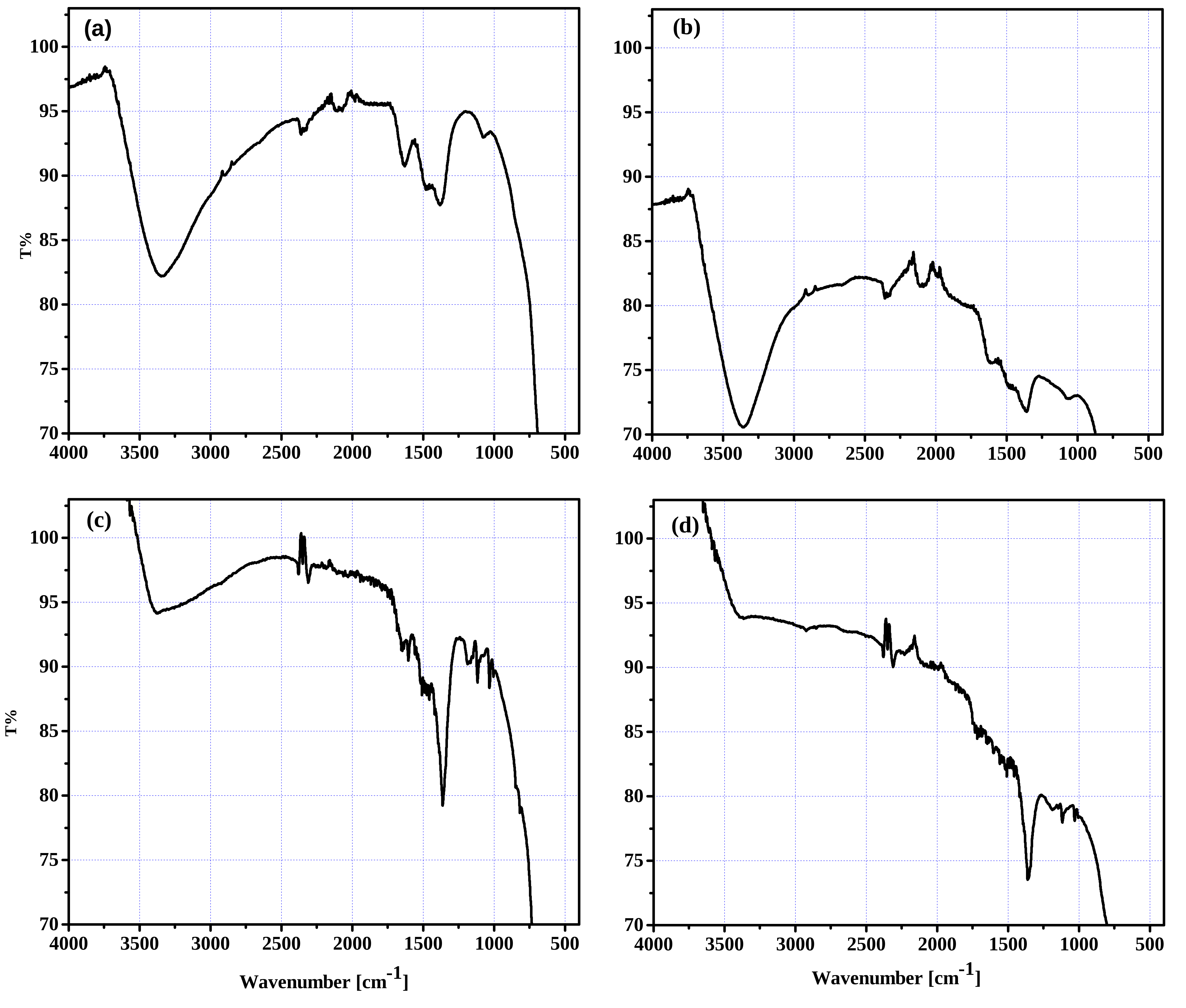

(a)
(b)
(c)
(d)
T%
100
95
90
85
80
75
70
4000
3500
3000
2500
2000
1500
1000
500
Wavenumber [cm-1]

جامعة الجوف
وكالة الجامعة للدراسات العليا والبحث العلمي
المختبرات المركزية

SHIMADZU

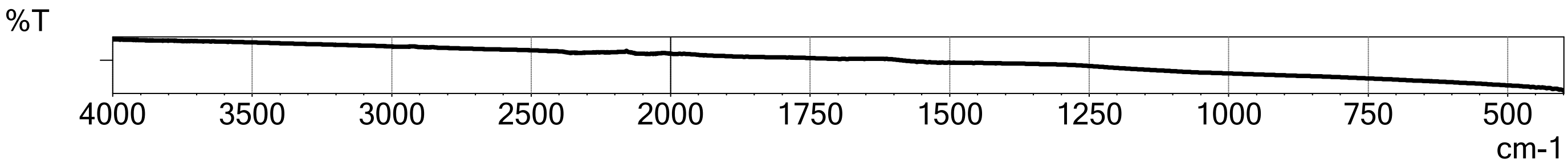

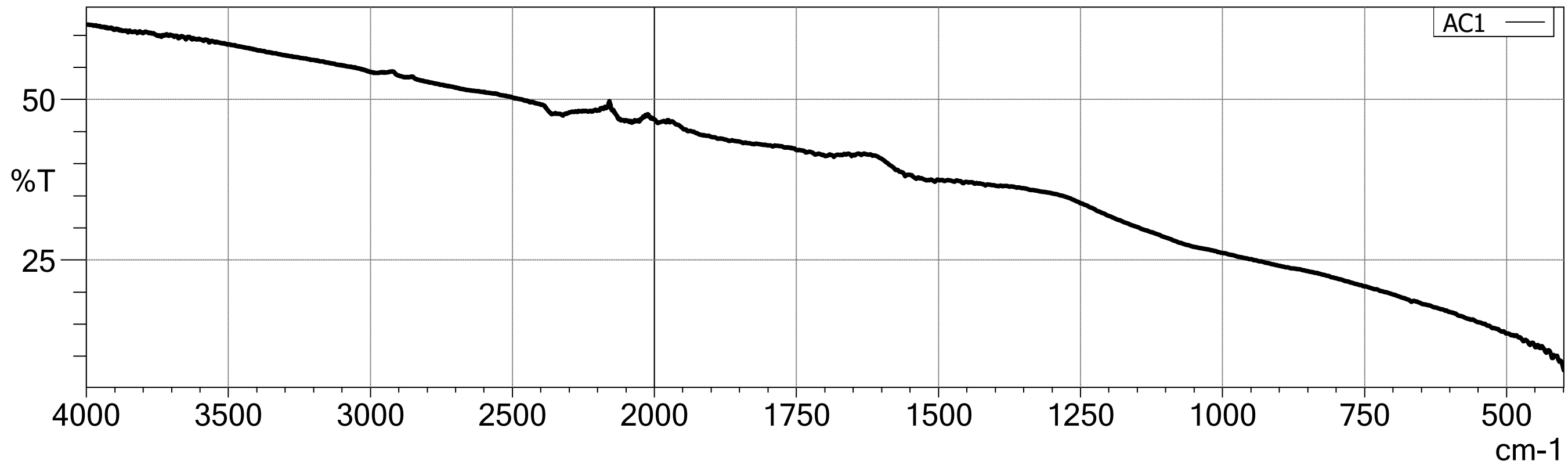

	Peak	Intensity	Corr. Intensity	Base (H)	Base (L)	Area	Corr. Area	Comment
1								

##TITLE=No Description

##DATA TYPE=INFRARED SPECTRUM

##XUNITS=1/CM

##YUNITS=%T

399.264912	7.855422
401.193728	8.467894
403.122544	9.173106
405.051360	9.359271
406.980176	9.162660
408.908992	9.485757
410.837808	10.104084
412.766624	10.033709
414.695440	10.029060
416.624256	10.197703
418.553072	9.745975
420.481888	9.761313
422.410704	10.345065
424.339520	10.810037
426.268336	10.980808
428.197152	10.888208
430.125968	10.520033
432.054784	10.709375
433.983600	11.138407
435.912416	11.379726
437.841232	11.582792
439.770048	11.323078

441.698864	11.295341
443.627680	11.728680
445.556496	11.762324
447.485312	11.411057
449.414128	11.422421
451.342944	11.799772
453.271760	12.106595
455.200576	12.090068
457.129392	11.929399
459.058208	11.788167
460.987024	11.952946
462.915840	12.265980
464.844656	12.477410
466.773472	12.601742
468.702288	12.386697
470.631104	12.366119
472.559920	12.569056
474.488736	12.824380
476.417552	12.952801
478.346368	12.937331
480.275184	13.012427
482.204000	13.263945
484.132816	13.272209
486.061632	13.167650
487.990448	13.310421
489.919264	13.290211

491.848080	13.271996
493.776896	13.435509
495.705712	13.496414
497.634528	13.546792
499.563344	13.570515
501.492160	13.562578
503.420976	13.714788
505.349792	13.903546
507.278608	13.872423
509.207424	13.856152
511.136240	13.952878
513.065056	14.082568
514.993872	14.230667
516.922688	14.273192
518.851504	14.329206
520.780320	14.373473
522.709136	14.412323
524.637952	14.393392
526.566768	14.492794
528.495584	14.684956
530.424400	14.768659
532.353216	14.778853
534.282032	14.776049
536.210848	14.884927
538.139664	15.046298
540.068480	15.043214

541.997296	15.070779
543.926112	15.178555
545.854928	15.254079
547.783744	15.290101
549.712560	15.304344
551.641376	15.347757
553.570192	15.423897
555.499008	15.523643
557.427824	15.630913
559.356640	15.766080
561.285456	15.720008
563.214272	15.707102
565.143088	15.808821
567.071904	15.831130
569.000720	15.857742
570.929536	15.905134
572.858352	15.968768
574.787168	16.058376
576.715984	16.176183
578.644800	16.236878
580.573616	16.272193
582.502432	16.301820
584.431248	16.337982
586.360064	16.412051
588.288880	16.545377
590.217696	16.667337

592.146512	16.689094
594.075328	16.773967
596.004144	16.801053
597.932960	16.843217
599.861776	16.931248
601.790592	16.927031
603.719408	17.039825
605.648224	17.124776
607.577040	17.087695
609.505856	17.174789
611.434672	17.244923
613.363488	17.291358
615.292304	17.324349
617.221120	17.368303
619.149936	17.458306
621.078752	17.508931
623.007568	17.557410
624.936384	17.582162
626.865200	17.607563
628.794016	17.669580
630.722832	17.730637
632.651648	17.820653
634.580464	17.917795
636.509280	17.962646
638.438096	17.959494
640.366912	18.032242

642.295728	18.087171
644.224544	18.097989
646.153360	18.148896
648.082176	18.158418
650.010992	18.201331
651.939808	18.284928
653.868624	18.365555
655.797440	18.431221
657.726256	18.512425
659.655072	18.546200
661.583888	18.608693
663.512704	18.658972
665.441520	18.606770
667.370336	18.450650
669.299152	18.574197
671.227968	18.769325
673.156784	18.840133
675.085600	18.906276
677.014416	18.960796
678.943232	19.027918
680.872048	19.068636
682.800864	19.121327
684.729680	19.197448
686.658496	19.217900
688.587312	19.261456
690.516128	19.365047

692.444944	19.404298
694.373760	19.466172
696.302576	19.567212
698.231392	19.594470
700.160208	19.635007
702.089024	19.711394
704.017840	19.767870
705.946656	19.832417
707.875472	19.885082
709.804288	19.915720
711.733104	19.957198
713.661920	19.987405
715.590736	20.064053
717.519552	20.118947
719.448368	20.134872
721.377184	20.177211
723.306000	20.230175
725.234816	20.313736
727.163632	20.369403
729.092448	20.419968
731.021264	20.438752
732.950080	20.463964
734.878896	20.506113
736.807712	20.560190
738.736528	20.628800
740.665344	20.657610

742.594160	20.712932
744.522976	20.785043
746.451792	20.839807
748.380608	20.890481
750.309424	20.919978
752.238240	20.932646
754.167056	21.000207
756.095872	21.068990
758.024688	21.101053
759.953504	21.143892
761.882320	21.206996
763.811136	21.246351
765.739952	21.258413
767.668768	21.315566
769.597584	21.379996
771.526400	21.432146
773.455216	21.484627
775.384032	21.515123
777.312848	21.585668
779.241664	21.649043
781.170480	21.685685
783.099296	21.714752
785.028112	21.739490
786.956928	21.809240
788.885744	21.875032
790.814560	21.956874

792.743376	21.987835
794.672192	22.006598
796.601008	22.090962
798.529824	22.136969
800.458640	22.167047
802.387456	22.201002
804.316272	22.252104
806.245088	22.283838
808.173904	22.315011
810.102720	22.375673
812.031536	22.467000
813.960352	22.506799
815.889168	22.513773
817.817984	22.566631
819.746800	22.622090
821.675616	22.676948
823.604432	22.726889
825.533248	22.772504
827.462064	22.831086
829.390880	22.870415
831.319696	22.904625
833.248512	22.970961
835.177328	23.003516
837.106144	23.015950
839.034960	23.054562
840.963776	23.098732

842.892592	23.129050
844.821408	23.160164
846.750224	23.190874
848.679040	23.223297
850.607856	23.266320
852.536672	23.297334
854.465488	23.331464
856.394304	23.384625
858.323120	23.414764
860.251936	23.451593
862.180752	23.517099
864.109568	23.564403
866.038384	23.573715
867.967200	23.573216
869.896016	23.615009
871.824832	23.650647
873.753648	23.666261
875.682464	23.671275
877.611280	23.666921
879.540096	23.699176
881.468912	23.766585
883.397728	23.822760
885.326544	23.847030
887.255360	23.844294
889.184176	23.871568
891.112992	23.924767

893.041808	23.959323
894.970624	23.992653
896.899440	24.033050
898.828256	24.085120
900.757072	24.115145
902.685888	24.148000
904.614704	24.188715
906.543520	24.224415
908.472336	24.286197
910.401152	24.339463
912.329968	24.342588
914.258784	24.354198
916.187600	24.420769
918.116416	24.477281
920.045232	24.509215
921.974048	24.535606
923.902864	24.574574
925.831680	24.630423
927.760496	24.675500
929.689312	24.719661
931.618128	24.764983
933.546944	24.790154
935.475760	24.809978
937.404576	24.853436
939.333392	24.912007
941.262208	24.947044

943.191024	24.938781
945.119840	24.979419
947.048656	25.056149
948.977472	25.106490
950.906288	25.126408
952.835104	25.140605
954.763920	25.182135
956.692736	25.227000
958.621552	25.277190
960.550368	25.295909
962.479184	25.312017
964.408000	25.356884
966.336816	25.406610
968.265632	25.430281
970.194448	25.463412
972.123264	25.500438
974.052080	25.557389
975.980896	25.612280
977.909712	25.656998
979.838528	25.702084
981.767344	25.741174
983.696160	25.783531
985.624976	25.813140
987.553792	25.834248
989.482608	25.863396
991.411424	25.931477

993.340240	26.009533
995.269056	26.045459
997.197872	26.041750
999.126688	26.060547
1001.055504	26.108578
1002.984320	26.136820
1004.913136	26.168063
1006.841952	26.212815
1008.770768	26.258590
1010.699584	26.323379
1012.628400	26.382172
1014.557216	26.432640
1016.486032	26.459318
1018.414848	26.494044
1020.343664	26.539459
1022.272480	26.583377
1024.201296	26.624999
1026.130112	26.651508
1028.058928	26.676155
1029.987744	26.721474
1031.916560	26.749488
1033.845376	26.753980
1035.774192	26.790305
1037.703008	26.826091
1039.631824	26.846908
1041.560640	26.894887

1043.489456	26.932055
1045.418272	26.962465
1047.347088	26.999860
1049.275904	27.029638
1051.204720	27.065056
1053.133536	27.098373
1055.062352	27.166967
1056.991168	27.216431
1058.919984	27.265066
1060.848800	27.327994
1062.777616	27.351175
1064.706432	27.389468
1066.635248	27.436135
1068.564064	27.517550
1070.492880	27.587196
1072.421696	27.607362
1074.350512	27.655455
1076.279328	27.690348
1078.208144	27.761400
1080.136960	27.835658
1082.065776	27.889996
1083.994592	27.967883
1085.923408	28.038803
1087.852224	28.093571
1089.781040	28.173883
1091.709856	28.249944

1093.638672	28.303768
1095.567488	28.364360
1097.496304	28.434754
1099.425120	28.501698
1101.353936	28.563304
1103.282752	28.604309
1105.211568	28.674433
1107.140384	28.747635
1109.069200	28.817728
1110.998016	28.893097
1112.926832	28.951294
1114.855648	29.005913
1116.784464	29.047361
1118.713280	29.118407
1120.642096	29.204092
1122.570912	29.276244
1124.499728	29.308933
1126.428544	29.345251
1128.357360	29.402919
1130.286176	29.491453
1132.214992	29.564998
1134.143808	29.610011
1136.072624	29.664803
1138.001440	29.722699
1139.930256	29.787285
1141.859072	29.833325

1143.787888	29.882760
1145.716704	29.973617
1147.645520	30.052200
1149.574336	30.115090
1151.503152	30.178188
1153.431968	30.231164
1155.360784	30.291461
1157.289600	30.346494
1159.218416	30.396469
1161.147232	30.459380
1163.076048	30.540483
1165.004864	30.622708
1166.933680	30.678929
1168.862496	30.747891
1170.791312	30.817976
1172.720128	30.872481
1174.648944	30.945356
1176.577760	31.017005
1178.506576	31.105006
1180.435392	31.191607
1182.364208	31.224510
1184.293024	31.277328
1186.221840	31.334578
1188.150656	31.396795
1190.079472	31.474661
1192.008288	31.548995

1193.937104	31.631055
1195.865920	31.710030
1197.794736	31.790067
1199.723552	31.853429
1201.652368	31.911206
1203.581184	31.986057
1205.510000	32.088270
1207.438816	32.182184
1209.367632	32.245894
1211.296448	32.286745
1213.225264	32.378118
1215.154080	32.472646
1217.082896	32.550646
1219.011712	32.608522
1220.940528	32.677083
1222.869344	32.765390
1224.798160	32.865550
1226.726976	32.971953
1228.655792	33.056053
1230.584608	33.123722
1232.513424	33.174467
1234.442240	33.243835
1236.371056	33.339093
1238.299872	33.443437
1240.228688	33.506229
1242.157504	33.558842

1244.086320	33.651401
1246.015136	33.748202
1247.943952	33.801408
1249.872768	33.842040
1251.801584	33.937655
1253.730400	34.036156
1255.659216	34.095805
1257.588032	34.178951
1259.516848	34.274340
1261.445664	34.335898
1263.374480	34.397155
1265.303296	34.474581
1267.232112	34.550009
1269.160928	34.613042
1271.089744	34.661631
1273.018560	34.722035
1274.947376	34.790313
1276.876192	34.848020
1278.805008	34.899132
1280.733824	34.950531
1282.662640	35.010193
1284.591456	35.057568
1286.520272	35.100084
1288.449088	35.143386
1290.377904	35.191543
1292.306720	35.235422

1294.235536	35.269938
1296.164352	35.294046
1298.093168	35.330784
1300.021984	35.383629
1301.950800	35.420371
1303.879616	35.447180
1305.808432	35.467545
1307.737248	35.497793
1309.666064	35.540498
1311.594880	35.577115
1313.523696	35.607433
1315.452512	35.609952
1317.381328	35.617209
1319.310144	35.657962
1321.238960	35.695687
1323.167776	35.739980
1325.096592	35.781317
1327.025408	35.806546
1328.954224	35.842489
1330.883040	35.862023
1332.811856	35.880973
1334.740672	35.891348
1336.669488	35.887044
1338.598304	35.886778
1340.527120	35.942025
1342.455936	36.036022

1344.384752	36.091827
1346.313568	36.120720
1348.242384	36.134424
1350.171200	36.161553
1352.100016	36.192555
1354.028832	36.212205
1355.957648	36.249915
1357.886464	36.289130
1359.815280	36.291215
1361.744096	36.240603
1363.672912	36.283545
1365.601728	36.388506
1367.530544	36.410696
1369.459360	36.441602
1371.388176	36.456238
1373.316992	36.392465
1375.245808	36.414313
1377.174624	36.485507
1379.103440	36.525854
1381.032256	36.541396
1382.961072	36.534914
1384.889888	36.511225
1386.818704	36.480669
1388.747520	36.524711
1390.676336	36.585415
1392.605152	36.544955

1394.533968	36.485214
1396.462784	36.524745
1398.391600	36.531348
1400.320416	36.611531
1402.249232	36.673181
1404.178048	36.636449
1406.106864	36.669094
1408.035680	36.714623
1409.964496	36.715957
1411.893312	36.748137
1413.822128	36.771070
1415.750944	36.732384
1417.679760	36.615242
1419.608576	36.683626
1421.537392	36.831544
1423.466208	36.816937
1425.395024	36.887192
1427.323840	36.924452
1429.252656	36.894396
1431.181472	36.932711
1433.110288	36.973354
1435.039104	36.889749
1436.967920	36.900770
1438.896736	37.045686
1440.825552	37.086612
1442.754368	37.110819

1444.683184	37.124864
1446.612000	37.081923
1448.540816	37.090914
1450.469632	37.146167
1452.398448	37.162490
1454.327264	37.081328
1456.256080	36.935142
1458.184896	37.013652
1460.113712	37.208391
1462.042528	37.300617
1463.971344	37.277364
1465.900160	37.318686
1467.828976	37.386057
1469.757792	37.334623
1471.686608	37.187973
1473.615424	37.219837
1475.544240	37.281874
1477.473056	37.350291
1479.401872	37.417347
1481.330688	37.408570
1483.259504	37.434942
1485.188320	37.420274
1487.117136	37.357495
1489.045952	37.274012
1490.974768	37.356211
1492.903584	37.476560

1494.832400	37.435469
1496.761216	37.382311
1498.690032	37.427547
1500.618848	37.491681
1502.547664	37.497061
1504.476480	37.355522
1506.405296	37.144344
1508.334112	37.263580
1510.262928	37.413154
1512.191744	37.497529
1514.120560	37.504617
1516.049376	37.415339
1517.978192	37.446303
1519.907008	37.394702
1521.835824	37.389317
1523.764640	37.538367
1525.693456	37.562976
1527.622272	37.639372
1529.551088	37.766963
1531.479904	37.734652
1533.408720	37.688179
1535.337536	37.869160
1537.266352	37.828182
1539.195168	37.657955
1541.123984	37.761089
1543.052800	37.879965

1544.981616	38.011638
1546.910432	38.212277
1548.839248	38.221399
1550.768064	38.251680
1552.696880	38.286011
1554.625696	38.291545
1556.554512	38.239441
1558.483328	38.046808
1560.412144	38.400104
1562.340960	38.634604
1564.269776	38.680906
1566.198592	38.763794
1568.127408	38.748402
1570.056224	38.827027
1571.985040	39.023107
1573.913856	39.026731
1575.842672	39.031163
1577.771488	39.245263
1579.700304	39.409026
1581.629120	39.549008
1583.557936	39.679144
1585.486752	39.792469
1587.415568	39.920915
1589.344384	40.045996
1591.273200	40.180154
1593.202016	40.310407

1595.130832	40.450784
1597.059648	40.603085
1598.988464	40.727128
1600.917280	40.821248
1602.846096	40.905103
1604.774912	41.018556
1606.703728	41.093123
1608.632544	41.145975
1610.561360	41.221293
1612.490176	41.307712
1614.418992	41.280797
1616.347808	41.233290
1618.276624	41.369395
1620.205440	41.432011
1622.134256	41.384653
1624.063072	41.423760
1625.991888	41.480528
1627.920704	41.480103
1629.849520	41.561070
1631.778336	41.576948
1633.707152	41.435977
1635.635968	41.326362
1637.564784	41.457817
1639.493600	41.549653
1641.422416	41.575751
1643.351232	41.529265

1645.280048	41.382393
1647.208864	41.298933
1649.137680	41.420626
1651.066496	41.338655
1652.995312	41.154346
1654.924128	41.378595
1656.852944	41.506311
1658.781760	41.543541
1660.710576	41.487476
1662.639392	41.426917
1664.568208	41.510169
1666.497024	41.490319
1668.425840	41.343476
1670.354656	41.318534
1672.283472	41.375756
1674.212288	41.337454
1676.141104	41.387121
1678.069920	41.410084
1679.998736	41.347908
1681.927552	41.221697
1683.856368	41.068355
1685.785184	41.249705
1687.714000	41.333862
1689.642816	41.357487
1691.571632	41.439738
1693.500448	41.367804

1695.429264	41.222042
1697.358080	41.245824
1699.286896	41.153530
1701.215712	41.282030
1703.144528	41.397898
1705.073344	41.342847
1707.002160	41.430655
1708.930976	41.527523
1710.859792	41.568315
1712.788608	41.530441
1714.717424	41.461663
1716.646240	41.372818
1718.575056	41.491108
1720.503872	41.702700
1722.432688	41.751515
1724.361504	41.786030
1726.290320	41.849374
1728.219136	41.858782
1730.147952	41.820953
1732.076768	41.750357
1734.005584	41.731727
1735.934400	41.975580
1737.863216	42.015252
1739.792032	42.016816
1741.720848	42.079231
1743.649664	42.084314

1745.578480	42.128998
1747.507296	42.117430
1749.436112	42.109440
1751.364928	42.222422
1753.293744	42.380844
1755.222560	42.392000
1757.151376	42.407478
1759.080192	42.471094
1761.009008	42.437691
1762.937824	42.491987
1764.866640	42.560115
1766.795456	42.514944
1768.724272	42.528771
1770.653088	42.519437
1772.581904	42.537880
1774.510720	42.649447
1776.439536	42.715499
1778.368352	42.743913
1780.297168	42.755314
1782.225984	42.801233
1784.154800	42.773133
1786.083616	42.795026
1788.012432	42.831093
1789.941248	42.742977
1791.870064	42.695325
1793.798880	42.794835

1795.727696	42.834259
1797.656512	42.855543
1799.585328	42.846340
1801.514144	42.823829
1803.442960	42.909250
1805.371776	42.936778
1807.300592	42.904052
1809.229408	42.934686
1811.158224	42.962657
1813.087040	42.992670
1815.015856	43.029328
1816.944672	43.058894
1818.873488	43.099420
1820.802304	43.116262
1822.731120	43.069946
1824.659936	43.010554
1826.588752	43.056210
1828.517568	43.065541
1830.446384	43.123016
1832.375200	43.210776
1834.304016	43.193256
1836.232832	43.199830
1838.161648	43.234909
1840.090464	43.305774
1842.019280	43.247183
1843.948096	43.198799

1845.876912	43.287711
1847.805728	43.341090
1849.734544	43.435292
1851.663360	43.489598
1853.592176	43.488134
1855.520992	43.495544
1857.449808	43.528831
1859.378624	43.533453
1861.307440	43.581651
1863.236256	43.629240
1865.165072	43.590379
1867.093888	43.526851
1869.022704	43.539338
1870.951520	43.642825
1872.880336	43.713499
1874.809152	43.753985
1876.737968	43.796263
1878.666784	43.840236
1880.595600	43.871676
1882.524416	43.905525
1884.453232	43.927163
1886.382048	43.908814
1888.310864	43.912499
1890.239680	43.964674
1892.168496	44.041710
1894.097312	44.103401

1896.026128	44.114539
1897.954944	44.105797
1899.883760	44.152195
1901.812576	44.244034
1903.741392	44.325282
1905.670208	44.360475
1907.599024	44.352515
1909.527840	44.362419
1911.456656	44.415096
1913.385472	44.437922
1915.314288	44.423111
1917.243104	44.470521
1919.171920	44.532236
1921.100736	44.534108
1923.029552	44.621196
1924.958368	44.734327
1926.887184	44.782563
1928.816000	44.894393
1930.744816	45.003191
1932.673632	45.008202
1934.602448	45.044366
1936.531264	45.094277
1938.460080	45.086496
1940.388896	45.031424
1942.317712	45.131309
1944.246528	45.311717

1946.175344	45.311605
1948.104160	45.307117
1950.032976	45.481785
1951.961792	45.693476
1953.890608	45.742965
1955.819424	45.888664
1957.748240	46.041929
1959.677056	46.073672
1961.605872	46.098717
1963.534688	46.176165
1965.463504	46.356237
1967.392320	46.527688
1969.321136	46.608385
1971.249952	46.493947
1973.178768	46.446801
1975.107584	46.788260
1977.036400	46.756856
1978.965216	46.417932
1980.894032	46.470976
1982.822848	46.643202
1984.751664	46.615041
1986.680480	46.531453
1988.609296	46.467180
1990.538112	46.459616
1992.466928	46.404602
1994.395744	46.334151

1996.324560	46.510817
1998.253376	46.776749
2000.182192	46.915084
2002.111008	47.008829
2004.039824	47.066649
2005.968640	46.969402
2007.897456	47.003683
2009.826272	47.165900
2011.755088	47.013940
2013.683904	47.201713
2015.612720	47.236438
2017.541536	47.267536
2019.470352	47.468057
2021.399168	47.631014
2023.327984	47.700348
2025.256800	47.391729
2027.185616	47.418075
2029.114432	47.597864
2031.043248	47.578876
2032.972064	47.368861
2034.900880	47.246509
2036.829696	47.310731
2038.758512	47.331920
2040.687328	47.191227
2042.616144	47.058873
2044.544960	47.033583

2046.473776	47.016822
2048.402592	46.851925
2050.331408	46.711032
2052.260224	46.683320
2054.189040	46.548354
2056.117856	46.662639
2058.046672	46.767536
2059.975488	46.710701
2061.904304	46.601639
2063.833120	46.609215
2065.761936	46.677370
2067.690752	46.649831
2069.619568	46.753308
2071.548384	46.682934
2073.477200	46.496042
2075.406016	46.477291
2077.334832	46.444913
2079.263648	46.375546
2081.192464	46.552959
2083.121280	46.674425
2085.050096	46.637624
2086.978912	46.567990
2088.907728	46.494456
2090.836544	46.526671
2092.765360	46.652976
2094.694176	46.735659

2096.622992	46.730411
2098.551808	46.743431
2100.480624	46.753400
2102.409440	46.693147
2104.338256	46.628564
2106.267072	46.684184
2108.195888	46.760284
2110.124704	46.788156
2112.053520	46.795318
2113.982336	46.806500
2115.911152	46.742773
2117.839968	46.725369
2119.768784	46.873497
2121.697600	46.992630
2123.626416	46.931964
2125.555232	46.907813
2127.484048	47.018859
2129.412864	47.136572
2131.341680	47.391537
2133.270496	47.555426
2135.199312	47.615534
2137.128128	47.842698
2139.056944	47.925580
2140.985760	47.935227
2142.914576	48.203976
2144.843392	48.400524

2146.772208	48.285576
2148.701024	48.301305
2150.629840	48.411354
2152.558656	48.649920
2154.487472	49.164819
2156.416288	49.510246
2158.345104	49.715943
2160.273920	49.421704
2162.202736	48.917093
2164.131552	48.818165
2166.060368	48.875698
2167.989184	48.766893
2169.918000	48.661705
2171.846816	48.787843
2173.775632	48.853859
2175.704448	48.767816
2177.633264	48.605655
2179.562080	48.546274
2181.490896	48.644153
2183.419712	48.717616
2185.348528	48.617224
2187.277344	48.586160
2189.206160	48.609923
2191.134976	48.376952
2193.063792	48.266316
2194.992608	48.302570

2196.921424	48.267261
2198.850240	48.337788
2200.779056	48.285617
2202.707872	48.218818
2204.636688	48.298672
2206.565504	48.392416
2208.494320	48.400704
2210.423136	48.298370
2212.351952	48.271769
2214.280768	48.271692
2216.209584	48.154176
2218.138400	48.131951
2220.067216	48.112999
2221.996032	48.135422
2223.924848	48.219196
2225.853664	48.236914
2227.782480	48.206571
2229.711296	48.151600
2231.640112	48.124745
2233.568928	48.090598
2235.497744	48.120566
2237.426560	48.190982
2239.355376	48.202186
2241.284192	48.213606
2243.213008	48.199296
2245.141824	48.171657

2247.070640	48.218140
2248.999456	48.198820
2250.928272	48.213749
2252.857088	48.202500
2254.785904	48.136389
2256.714720	48.154266
2258.643536	48.175594
2260.572352	48.188889
2262.501168	48.180995
2264.429984	48.186143
2266.358800	48.227298
2268.287616	48.121422
2270.216432	48.061614
2272.145248	48.079271
2274.074064	48.073512
2276.002880	48.132996
2277.931696	48.151590
2279.860512	48.099037
2281.789328	48.050112
2283.718144	48.040413
2285.646960	48.080107
2287.575776	48.126365
2289.504592	48.092970
2291.433408	48.052036
2293.362224	48.047361
2295.291040	48.008404

2297.219856	47.993434
2299.148672	48.010548
2301.077488	47.956734
2303.006304	47.903889
2304.935120	47.895245
2306.863936	47.825028
2308.792752	47.758643
2310.721568	47.782239
2312.650384	47.815511
2314.579200	47.763696
2316.508016	47.720123
2318.436832	47.692831
2320.365648	47.600508
2322.294464	47.500335
2324.223280	47.520488
2326.152096	47.627748
2328.080912	47.655912
2330.009728	47.703468
2331.938544	47.743437
2333.867360	47.754281
2335.796176	47.772761
2337.724992	47.754787
2339.653808	47.750976
2341.582624	47.764569
2343.511440	47.746672
2345.440256	47.776084

2347.369072	47.883332
2349.297888	47.884953
2351.226704	47.813622
2353.155520	47.784669
2355.084336	47.749856
2357.013152	47.743259
2358.941968	47.773567
2360.870784	47.718301
2362.799600	47.715237
2364.728416	47.818838
2366.657232	47.923623
2368.586048	47.972013
2370.514864	48.046416
2372.443680	48.180817
2374.372496	48.305147
2376.301312	48.401174
2378.230128	48.478837
2380.158944	48.609185
2382.087760	48.727195
2384.016576	48.830710
2385.945392	48.963963
2387.874208	49.061300
2389.803024	49.095394
2391.731840	49.120792
2393.660656	49.144212
2395.589472	49.187026

2397.518288	49.199700
2399.447104	49.175484
2401.375920	49.198915
2403.304736	49.249972
2405.233552	49.323421
2407.162368	49.330008
2409.091184	49.323199
2411.020000	49.336181
2412.948816	49.361609
2414.877632	49.384519
2416.806448	49.397249
2418.735264	49.423618
2420.664080	49.461500
2422.592896	49.496266
2424.521712	49.494801
2426.450528	49.555243
2428.379344	49.600646
2430.308160	49.596349
2432.236976	49.587872
2434.165792	49.575360
2436.094608	49.622078
2438.023424	49.604131
2439.952240	49.567256
2441.881056	49.660895
2443.809872	49.734388
2445.738688	49.755589

2447.667504	49.792942
2449.596320	49.821440
2451.525136	49.808486
2453.453952	49.804098
2455.382768	49.817212
2457.311584	49.835410
2459.240400	49.864218
2461.169216	49.896934
2463.098032	49.938291
2465.026848	49.978513
2466.955664	50.025221
2468.884480	50.046743
2470.813296	50.034004
2472.742112	50.035757
2474.670928	50.078198
2476.599744	50.100420
2478.528560	50.093837
2480.457376	50.097066
2482.386192	50.130680
2484.315008	50.167154
2486.243824	50.179165
2488.172640	50.187704
2490.101456	50.160031
2492.030272	50.166549
2493.959088	50.227348
2495.887904	50.282833

2497.816720	50.290484
2499.745536	50.289679
2501.674352	50.322898
2503.603168	50.377714
2505.531984	50.414212
2507.460800	50.413701
2509.389616	50.445101
2511.318432	50.489123
2513.247248	50.490094
2515.176064	50.465358
2517.104880	50.486028
2519.033696	50.501922
2520.962512	50.527447
2522.891328	50.567013
2524.820144	50.582150
2526.748960	50.598906
2528.677776	50.593467
2530.606592	50.597608
2532.535408	50.613380
2534.464224	50.635813
2536.393040	50.665392
2538.321856	50.668941
2540.250672	50.663545
2542.179488	50.680179
2544.108304	50.702463
2546.037120	50.738859

2547.965936	50.749801
2549.894752	50.770555
2551.823568	50.841353
2553.752384	50.894008
2555.681200	50.875217
2557.610016	50.876265
2559.538832	50.932181
2561.467648	50.881492
2563.396464	50.899170
2565.325280	50.943742
2567.254096	50.921301
2569.182912	50.940564
2571.111728	50.971187
2573.040544	50.970295
2574.969360	50.969946
2576.898176	51.012378
2578.826992	51.032477
2580.755808	51.034521
2582.684624	51.033519
2584.613440	51.050482
2586.542256	51.064434
2588.471072	51.082954
2590.399888	51.091132
2592.328704	51.090460
2594.257520	51.088844
2596.186336	51.123034

2598.115152	51.153950
2600.043968	51.138195
2601.972784	51.156346
2603.901600	51.191729
2605.830416	51.190989
2607.759232	51.175387
2609.688048	51.181830
2611.616864	51.198703
2613.545680	51.211854
2615.474496	51.233053
2617.403312	51.256162
2619.332128	51.270038
2621.260944	51.271658
2623.189760	51.270178
2625.118576	51.300036
2627.047392	51.298027
2628.976208	51.281896
2630.905024	51.315241
2632.833840	51.328846
2634.762656	51.336937
2636.691472	51.357135
2638.620288	51.364088
2640.549104	51.368112
2642.477920	51.376054
2644.406736	51.395435
2646.335552	51.420280

2648.264368	51.429754
2650.193184	51.432207
2652.122000	51.439422
2654.050816	51.446030
2655.979632	51.468691
2657.908448	51.471904
2659.837264	51.460958
2661.766080	51.492906
2663.694896	51.525679
2665.623712	51.538307
2667.552528	51.545274
2669.481344	51.561726
2671.410160	51.581165
2673.338976	51.598759
2675.267792	51.631797
2677.196608	51.646080
2679.125424	51.658503
2681.054240	51.671290
2682.983056	51.670041
2684.911872	51.693164
2686.840688	51.729840
2688.769504	51.758190
2690.698320	51.766682
2692.627136	51.776823
2694.555952	51.790411
2696.484768	51.791056

2698.413584	51.807419
2700.342400	51.855322
2702.271216	51.879218
2704.200032	51.880008
2706.128848	51.905365
2708.057664	51.934724
2709.986480	51.963692
2711.915296	51.965215
2713.844112	51.970802
2715.772928	52.006303
2717.701744	52.025567
2719.630560	52.041763
2721.559376	52.049798
2723.488192	52.067705
2725.417008	52.079044
2727.345824	52.073463
2729.274640	52.105061
2731.203456	52.134077
2733.132272	52.138136
2735.061088	52.135884
2736.989904	52.149039
2738.918720	52.177218
2740.847536	52.212366
2742.776352	52.242637
2744.705168	52.253003
2746.633984	52.273691

2748.562800	52.287502
2750.491616	52.285455
2752.420432	52.301482
2754.349248	52.306097
2756.278064	52.324119
2758.206880	52.370017
2760.135696	52.399968
2762.064512	52.401694
2763.993328	52.423982
2765.922144	52.453881
2767.850960	52.467655
2769.779776	52.460587
2771.708592	52.464866
2773.637408	52.497182
2775.566224	52.507734
2777.495040	52.536462
2779.423856	52.547517
2781.352672	52.565786
2783.281488	52.600508
2785.210304	52.623436
2787.139120	52.627957
2789.067936	52.628946
2790.996752	52.652135
2792.925568	52.676995
2794.854384	52.704155
2796.783200	52.727994

2798.712016	52.730101
2800.640832	52.745655
2802.569648	52.795385
2804.498464	52.814485
2806.427280	52.815449
2808.356096	52.810062
2810.284912	52.815365
2812.213728	52.855911
2814.142544	52.909849
2816.071360	52.927924
2818.000176	52.916479
2819.928992	52.934660
2821.857808	52.967438
2823.786624	52.970673
2825.715440	52.981063
2827.644256	53.008431
2829.573072	53.028635
2831.501888	53.068236
2833.430704	53.085763
2835.359520	53.101129
2837.288336	53.139389
2839.217152	53.167088
2841.145968	53.194920
2843.074784	53.226603
2845.003600	53.305128
2846.932416	53.406292

2848.861232	53.501744
2850.790048	53.549762
2852.718864	53.527389
2854.647680	53.517123
2856.576496	53.528662
2858.505312	53.493588
2860.434128	53.479381
2862.362944	53.495816
2864.291760	53.468737
2866.220576	53.466337
2868.149392	53.475419
2870.078208	53.485208
2872.007024	53.482167
2873.935840	53.462462
2875.864656	53.491740
2877.793472	53.486078
2879.722288	53.458147
2881.651104	53.484533
2883.579920	53.502672
2885.508736	53.521581
2887.437552	53.561737
2889.366368	53.592453
2891.295184	53.612658
2893.224000	53.625963
2895.152816	53.638410
2897.081632	53.682928

2899.010448	53.703481
2900.939264	53.713072
2902.868080	53.744083
2904.796896	53.782646
2906.725712	53.827796
2908.654528	53.873307
2910.583344	53.928819
2912.512160	54.023195
2914.440976	54.160116
2916.369792	54.254591
2918.298608	54.301411
2920.227424	54.335620
2922.156240	54.363106
2924.085056	54.372608
2926.013872	54.364341
2927.942688	54.343037
2929.871504	54.322334
2931.800320	54.332468
2933.729136	54.286337
2935.657952	54.253814
2937.586768	54.267838
2939.515584	54.228031
2941.444400	54.190586
2943.373216	54.177209
2945.302032	54.191431
2947.230848	54.209823

2949.159664	54.226375
2951.088480	54.205647
2953.017296	54.205733
2954.946112	54.237272
2956.874928	54.214428
2958.803744	54.219244
2960.732560	54.244121
2962.661376	54.233229
2964.590192	54.212120
2966.519008	54.195150
2968.447824	54.182042
2970.376640	54.158066
2972.305456	54.146693
2974.234272	54.160344
2976.163088	54.152518
2978.091904	54.137980
2980.020720	54.138776
2981.949536	54.149418
2983.878352	54.158484
2985.807168	54.163660
2987.735984	54.141684
2989.664800	54.143177
2991.593616	54.194004
2993.522432	54.239773
2995.451248	54.256777
2997.380064	54.244839

2999.308880	54.256979
3001.237696	54.300200
3003.166512	54.330957
3005.095328	54.348297
3007.024144	54.375389
3008.952960	54.414833
3010.881776	54.432655
3012.810592	54.455145
3014.739408	54.493455
3016.668224	54.518179
3018.597040	54.549959
3020.525856	54.619793
3022.454672	54.651103
3024.383488	54.628813
3026.312304	54.663349
3028.241120	54.690137
3030.169936	54.677538
3032.098752	54.686032
3034.027568	54.718724
3035.956384	54.761272
3037.885200	54.809259
3039.814016	54.838285
3041.742832	54.837290
3043.671648	54.855236
3045.600464	54.884753
3047.529280	54.888438

3049.458096	54.906707
3051.386912	54.954796
3053.315728	54.974825
3055.244544	54.958995
3057.173360	54.978778
3059.102176	55.021134
3061.030992	55.026296
3062.959808	55.023919
3064.888624	55.036779
3066.817440	55.050641
3068.746256	55.086378
3070.675072	55.098722
3072.603888	55.113521
3074.532704	55.130420
3076.461520	55.105096
3078.390336	55.090626
3080.319152	55.129212
3082.247968	55.168352
3084.176784	55.171847
3086.105600	55.193009
3088.034416	55.215596
3089.963232	55.218278
3091.892048	55.239639
3093.820864	55.291074
3095.749680	55.304768
3097.678496	55.304083

3099.607312	55.326973
3101.536128	55.330701
3103.464944	55.313414
3105.393760	55.319497
3107.322576	55.355224
3109.251392	55.372089
3111.180208	55.387988
3113.109024	55.426700
3115.037840	55.410688
3116.966656	55.398551
3118.895472	55.434237
3120.824288	55.463911
3122.753104	55.488109
3124.681920	55.513925
3126.610736	55.571977
3128.539552	55.595040
3130.468368	55.570521
3132.397184	55.554814
3134.326000	55.574494
3136.254816	55.623356
3138.183632	55.631540
3140.112448	55.631990
3142.041264	55.663726
3143.970080	55.702224
3145.898896	55.710527
3147.827712	55.714282

3149.756528	55.734429
3151.685344	55.735423
3153.614160	55.751222
3155.542976	55.772382
3157.471792	55.778962
3159.400608	55.785579
3161.329424	55.824915
3163.258240	55.870060
3165.187056	55.900412
3167.115872	55.908617
3169.044688	55.906380
3170.973504	55.925583
3172.902320	55.928759
3174.831136	55.920451
3176.759952	55.939755
3178.688768	55.942075
3180.617584	55.966794
3182.546400	56.011991
3184.475216	56.016107
3186.404032	56.049939
3188.332848	56.073319
3190.261664	56.064560
3192.190480	56.060616
3194.119296	56.077212
3196.048112	56.117685
3197.976928	56.137764

3199.905744	56.160221
3201.834560	56.181147
3203.763376	56.158478
3205.692192	56.138488
3207.621008	56.145488
3209.549824	56.178806
3211.478640	56.223545
3213.407456	56.244581
3215.336272	56.256684
3217.265088	56.282189
3219.193904	56.274822
3221.122720	56.283465
3223.051536	56.329198
3224.980352	56.359491
3226.909168	56.364633
3228.837984	56.361928
3230.766800	56.366966
3232.695616	56.364475
3234.624432	56.380344
3236.553248	56.408997
3238.482064	56.430466
3240.410880	56.422630
3242.339696	56.420611
3244.268512	56.448245
3246.197328	56.468693
3248.126144	56.468684

3250.054960	56.516626
3251.983776	56.572122
3253.912592	56.565420
3255.841408	56.571132
3257.770224	56.566562
3259.699040	56.570320
3261.627856	56.603141
3263.556672	56.627804
3265.485488	56.624516
3267.414304	56.612618
3269.343120	56.632767
3271.271936	56.648627
3273.200752	56.655253
3275.129568	56.673740
3277.058384	56.716291
3278.987200	56.716923
3280.916016	56.716556
3282.844832	56.747256
3284.773648	56.751702
3286.702464	56.771110
3288.631280	56.778746
3290.560096	56.774930
3292.488912	56.805397
3294.417728	56.826821
3296.346544	56.824586
3298.275360	56.852784

3300.204176	56.874832
3302.132992	56.882792
3304.061808	56.907803
3305.990624	56.907515
3307.919440	56.899446
3309.848256	56.929353
3311.777072	56.961711
3313.705888	56.984532
3315.634704	57.017209
3317.563520	57.011504
3319.492336	57.017129
3321.421152	57.054926
3323.349968	57.089514
3325.278784	57.106376
3327.207600	57.108874
3329.136416	57.144618
3331.065232	57.165701
3332.994048	57.171802
3334.922864	57.186232
3336.851680	57.173039
3338.780496	57.181579
3340.709312	57.196101
3342.638128	57.196990
3344.566944	57.212693
3346.495760	57.241710
3348.424576	57.277935

3350.353392	57.299034
3352.282208	57.341299
3354.211024	57.340604
3356.139840	57.337859
3358.068656	57.374088
3359.997472	57.364536
3361.926288	57.373727
3363.855104	57.398369
3365.783920	57.398702
3367.712736	57.399927
3369.641552	57.434580
3371.570368	57.484159
3373.499184	57.495180
3375.428000	57.519832
3377.356816	57.528080
3379.285632	57.521164
3381.214448	57.543153
3383.143264	57.571342
3385.072080	57.595881
3387.000896	57.578208
3388.929712	57.603805
3390.858528	57.638424
3392.787344	57.609418
3394.716160	57.622182
3396.644976	57.670932
3398.573792	57.685220

3400.502608	57.676161
3402.431424	57.716716
3404.360240	57.755883
3406.289056	57.757111
3408.217872	57.766154
3410.146688	57.789780
3412.075504	57.822469
3414.004320	57.841015
3415.933136	57.874714
3417.861952	57.902785
3419.790768	57.878178
3421.719584	57.900075
3423.648400	57.928787
3425.577216	57.941771
3427.506032	58.000333
3429.434848	58.008501
3431.363664	58.000330
3433.292480	57.990621
3435.221296	58.008710
3437.150112	58.058464
3439.078928	58.077514
3441.007744	58.075832
3442.936560	58.096021
3444.865376	58.117648
3446.794192	58.102801
3448.723008	58.150104

3450.651824	58.176641
3452.580640	58.170673
3454.509456	58.195032
3456.438272	58.215424
3458.367088	58.219237
3460.295904	58.222911
3462.224720	58.248812
3464.153536	58.287048
3466.082352	58.293811
3468.011168	58.283168
3469.939984	58.282389
3471.868800	58.312607
3473.797616	58.375595
3475.726432	58.416142
3477.655248	58.445487
3479.584064	58.437413
3481.512880	58.424279
3483.441696	58.457770
3485.370512	58.475624
3487.299328	58.472283
3489.228144	58.486129
3491.156960	58.497298
3493.085776	58.522463
3495.014592	58.577475
3496.943408	58.599222
3498.872224	58.600449

3500.801040	58.573351
3502.729856	58.543076
3504.658672	58.587541
3506.587488	58.642379
3508.516304	58.637472
3510.445120	58.657144
3512.373936	58.722132
3514.302752	58.751361
3516.231568	58.776587
3518.160384	58.750122
3520.089200	58.727040
3522.018016	58.748878
3523.946832	58.764515
3525.875648	58.762117
3527.804464	58.747823
3529.733280	58.824755
3531.662096	58.875869
3533.590912	58.905168
3535.519728	58.905722
3537.448544	58.895007
3539.377360	58.955198
3541.306176	58.982010
3543.234992	58.947919
3545.163808	58.862204
3547.092624	58.861208
3549.021440	58.966957

3550.950256	59.004893
3552.879072	59.006163
3554.807888	59.040881
3556.736704	59.085805
3558.665520	59.104911
3560.594336	59.093846
3562.523152	59.086996
3564.451968	59.035268
3566.380784	58.889702
3568.309600	58.889475
3570.238416	59.078362
3572.167232	59.215661
3574.096048	59.271862
3576.024864	59.295512
3577.953680	59.307308
3579.882496	59.282389
3581.811312	59.321230
3583.740128	59.309118
3585.668944	59.186636
3587.597760	59.085379
3589.526576	59.151363
3591.455392	59.272214
3593.384208	59.286450
3595.313024	59.284265
3597.241840	59.327222
3599.170656	59.331420

3601.099472	59.346286
3603.028288	59.438768
3604.957104	59.471296
3606.885920	59.404745
3608.814736	59.359144
3610.743552	59.355456
3612.672368	59.316465
3614.601184	59.383802
3616.530000	59.451027
3618.458816	59.396057
3620.387632	59.407593
3622.316448	59.529465
3624.245264	59.585937
3626.174080	59.524762
3628.102896	59.368283
3630.031712	59.383351
3631.960528	59.524374
3633.889344	59.560136
3635.818160	59.684806
3637.746976	59.750954
3639.675792	59.740457
3641.604608	59.755678
3643.533424	59.759532
3645.462240	59.650397
3647.391056	59.442654
3649.319872	59.361209

3651.248688	59.454957
3653.177504	59.649773
3655.106320	59.710707
3657.035136	59.697691
3658.963952	59.770585
3660.892768	59.830976
3662.821584	59.860309
3664.750400	59.886926
3666.679216	59.840205
3668.608032	59.719412
3670.536848	59.648412
3672.465664	59.747748
3674.394480	59.669218
3676.323296	59.539225
3678.252112	59.716228
3680.180928	59.844261
3682.109744	59.928566
3684.038560	59.937848
3685.967376	59.904537
3687.896192	59.841110
3689.825008	59.724719
3691.753824	59.751753
3693.682640	59.905085
3695.611456	59.966211
3697.540272	59.977415
3699.469088	59.991920

3701.397904	59.954980
3703.326720	60.010247
3705.255536	60.083670
3707.184352	60.037950
3709.113168	59.932899
3711.041984	59.901499
3712.970800	59.945584
3714.899616	60.060294
3716.828432	60.161048
3718.757248	60.130022
3720.686064	60.049780
3722.614880	59.992293
3724.543696	59.972388
3726.472512	59.931159
3728.401328	60.007841
3730.330144	60.057330
3732.258960	59.947890
3734.187776	59.787347
3736.116592	59.727072
3738.045408	59.893929
3739.974224	59.993653
3741.903040	59.990236
3743.831856	59.922854
3745.760672	59.871819
3747.689488	59.956313
3749.618304	59.954416

3751.547120	59.905928
3753.475936	60.020439
3755.404752	60.104007
3757.333568	60.121791
3759.262384	60.137203
3761.191200	60.220050
3763.120016	60.310314
3765.048832	60.276079
3766.977648	60.254101
3768.906464	60.251684
3770.835280	60.270833
3772.764096	60.366353
3774.692912	60.398353
3776.621728	60.391395
3778.550544	60.328898
3780.479360	60.337569
3782.408176	60.434970
3784.336992	60.415337
3786.265808	60.436513
3788.194624	60.506700
3790.123440	60.534507
3792.052256	60.573366
3793.981072	60.534335
3795.909888	60.421026
3797.838704	60.378879
3799.767520	60.400548

3801.696336	60.332602
3803.625152	60.367611
3805.553968	60.417716
3807.482784	60.438830
3809.411600	60.536699
3811.340416	60.613834
3813.269232	60.589192
3815.198048	60.494585
3817.126864	60.457453
3819.055680	60.418744
3820.984496	60.316721
3822.913312	60.415441
3824.842128	60.573290
3826.770944	60.579305
3828.699760	60.613218
3830.628576	60.602686
3832.557392	60.538268
3834.486208	60.519544
3836.415024	60.483516
3838.343840	60.472716
3840.272656	60.470953
3842.201472	60.543682
3844.130288	60.597212
3846.059104	60.684565
3847.987920	60.734693
3849.916736	60.663631

3851.845552	60.516604
3853.774368	60.416646
3855.703184	60.554683
3857.632000	60.691081
3859.560816	60.749093
3861.489632	60.715821
3863.418448	60.682319
3865.347264	60.669998
3867.276080	60.752794
3869.204896	60.696776
3871.133712	60.660901
3873.062528	60.712735
3874.991344	60.691718
3876.920160	60.810002
3878.848976	60.818232
3880.777792	60.702579
3882.706608	60.781201
3884.635424	60.805999
3886.564240	60.798620
3888.493056	60.959154
3890.421872	60.945981
3892.350688	60.908888
3894.279504	61.008792
3896.208320	61.014672
3898.137136	60.913783
3900.065952	60.822460

3901.994768	60.823126
3903.923584	60.819587
3905.852400	60.922384
3907.781216	61.068189
3909.710032	61.129125
3911.638848	61.160192
3913.567664	61.176448
3915.496480	61.101892
3917.425296	61.066421
3919.354112	61.105054
3921.282928	61.157068
3923.211744	61.151897
3925.140560	61.101748
3927.069376	61.203687
3928.998192	61.242864
3930.927008	61.181922
3932.855824	61.164842
3934.784640	61.237449
3936.713456	61.316488
3938.642272	61.297128
3940.571088	61.289898
3942.499904	61.272607
3944.428720	61.279411
3946.357536	61.320875
3948.286352	61.295735
3950.215168	61.321104

3952.143984	61.396794
3954.072800	61.393512
3956.001616	61.400999
3957.930432	61.430735
3959.859248	61.428716
3961.788064	61.426290
3963.716880	61.452353
3965.645696	61.524103
3967.574512	61.557430
3969.503328	61.528692
3971.432144	61.521840
3973.360960	61.508009
3975.289776	61.489412
3977.218592	61.553872
3979.147408	61.588850
3981.076224	61.570932
3983.005040	61.605115
3984.933856	61.623231
3986.862672	61.591745
3988.791488	61.606108
3990.720304	61.650294
3992.649120	61.686063
3994.577936	61.682432
3996.506752	61.684017
3998.435568	61.677698
4000.364384	61.679981

SHIMADZU

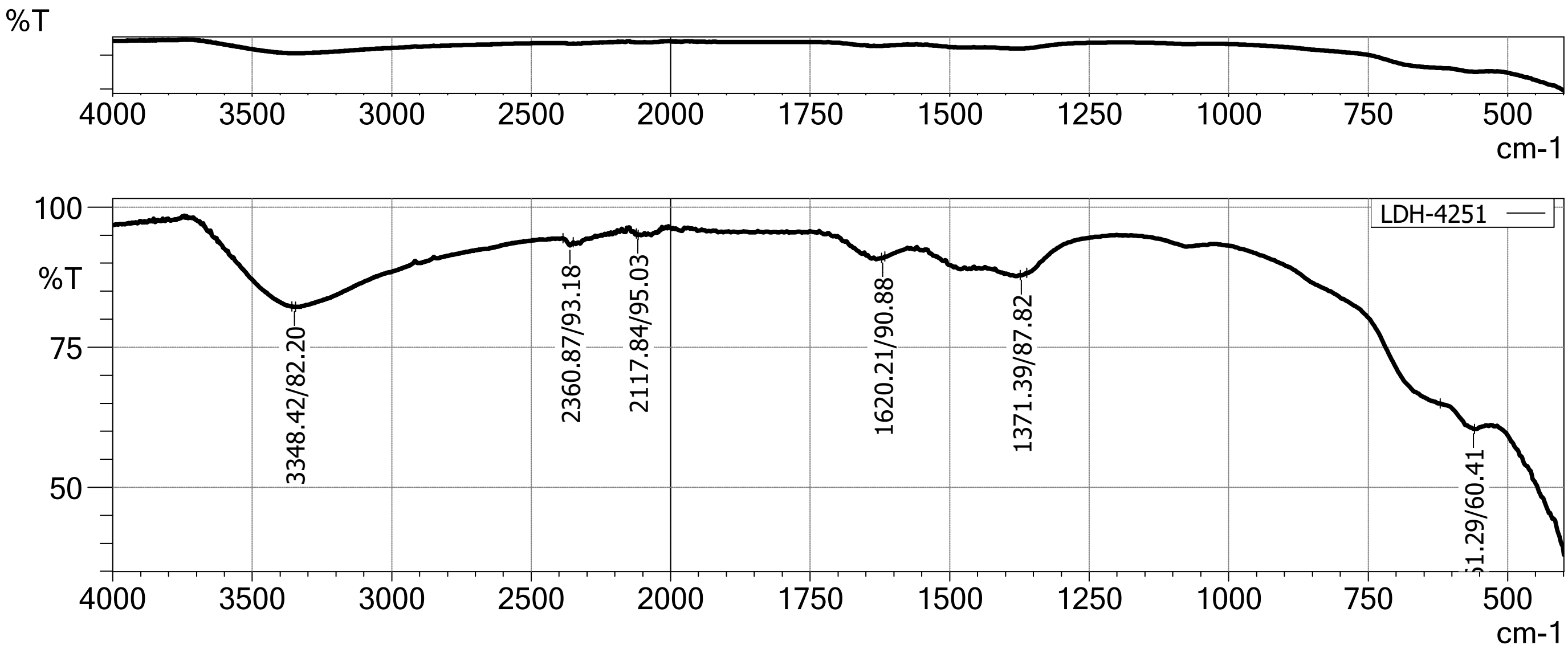

	Peak	Intensity	Corr. Intensity	Base (H)	Base (L)	Area	Corr. Area	Comment
1	561.29	60.41	0.15	621.08	559.36	2293.237	-8.477	
2	1371.39	87.82	0.14	1373.32	1361.74	139.084	1.131	
3	1620.21	90.88	0.15	1622.13	1616.35	52.099	0.459	
4	2117.84	95.03	0.09	2123.63	2115.91	37.683	0.553	
5	2360.87	93.18	0.77	2385.95	2349.30	229.594	12.732	
6	3348.42	82.20	0.06	3358.07	3344.57	239.949	0.456	

##TITLE=No Description

##DATA TYPE=INFRARED SPECTRUM

##XUNITS=1/CM

##YUNITS=%T

399.264912 37.990439

401.193728 39.430222

403.122544 39.805861

405.051360 40.340437

406.980176 41.214276

408.908992 41.717846

410.837808 42.348637

412.766624 43.168206

414.695440 44.065150

416.624256 44.315317

418.553072 44.466662

420.481888 44.385571

422.410704 44.945367

424.339520 45.428119

426.268336 45.402345

428.197152 45.876694

430.125968 46.322164

432.054784 46.896286

433.983600 47.363456

435.912416 47.967925

437.841232 48.266085

439.770048 48.280904

441.698864	48.847307
443.627680	49.170137
445.556496	49.725309
447.485312	50.332070
449.414128	50.591068
451.342944	51.009158
453.271760	51.223779
455.200576	51.475973
457.129392	52.187141
459.058208	52.916926
460.987024	53.071055
462.915840	53.410083
464.844656	53.742506
466.773472	53.823269
468.702288	53.982469
470.631104	54.230151
472.559920	54.754564
474.488736	55.369571
476.417552	55.581552
478.346368	55.694225
480.275184	56.315404
482.204000	56.706290
484.132816	56.838224
486.061632	57.112715
487.990448	57.360049
489.919264	57.830243

491.848080	58.089214
493.776896	58.209075
495.705712	58.531150
497.634528	58.869443
499.563344	59.199275
501.492160	59.449524
503.420976	59.742492
505.349792	59.922374
507.278608	60.106982
509.207424	60.252761
511.136240	60.368195
513.065056	60.536666
514.993872	60.582411
516.922688	60.816182
518.851504	60.988410
520.780320	60.943299
522.709136	60.839381
524.637952	60.882029
526.566768	61.060989
528.495584	61.088932
530.424400	61.119306
532.353216	61.022138
534.282032	60.923070
536.210848	60.969712
538.139664	61.049315
540.068480	61.075338

541.997296	60.938720
543.926112	60.875645
545.854928	60.892973
547.783744	60.766631
549.712560	60.745234
551.641376	60.679162
553.570192	60.499944
555.499008	60.420253
557.427824	60.396541
559.356640	60.420952
561.285456	60.413771
563.214272	60.530404
565.143088	60.673275
567.071904	60.691425
569.000720	60.776138
570.929536	60.920683
572.858352	61.078756
574.787168	61.090777
576.715984	61.156869
578.644800	61.546940
580.573616	61.866627
582.502432	62.070689
584.431248	62.288456
586.360064	62.499266
588.288880	62.764665
590.217696	63.027493

592.146512	63.275535
594.075328	63.521436
596.004144	63.705884
597.932960	63.952145
599.861776	64.150847
601.790592	64.300858
603.719408	64.461206
605.648224	64.528799
607.577040	64.577728
609.505856	64.664176
611.434672	64.695478
613.363488	64.734994
615.292304	64.746644
617.221120	64.750252
619.149936	64.894337
621.078752	64.995932
623.007568	64.984117
624.936384	64.972639
626.865200	65.066675
628.794016	65.214586
630.722832	65.292036
632.651648	65.325327
634.580464	65.405845
636.509280	65.409728
638.438096	65.467262
640.366912	65.568929

642.295728	65.683425
644.224544	65.783580
646.153360	65.854159
648.082176	66.005223
650.010992	66.066559
651.939808	66.139068
653.868624	66.261780
655.797440	66.356325
657.726256	66.547902
659.655072	66.703293
661.583888	66.803761
663.512704	66.941497
665.441520	67.052081
667.370336	67.066926
669.299152	67.189443
671.227968	67.462084
673.156784	67.650722
675.085600	67.895481
677.014416	68.103266
678.943232	68.268044
680.872048	68.513752
682.800864	68.660894
684.729680	68.847717
686.658496	69.105610
688.587312	69.383280
690.516128	69.663936

692.444944	69.997863
694.373760	70.295895
696.302576	70.594880
698.231392	70.983381
700.160208	71.353409
702.089024	71.696739
704.017840	72.061997
705.946656	72.422832
707.875472	72.778912
709.804288	73.165632
711.733104	73.558771
713.661920	73.983049
715.590736	74.376959
717.519552	74.750188
719.448368	75.153234
721.377184	75.524628
723.306000	75.939288
725.234816	76.330154
727.163632	76.705938
729.092448	77.089645
731.021264	77.438687
732.950080	77.799720
734.878896	78.088551
736.807712	78.392771
738.736528	78.736904
740.665344	79.037876

742.594160	79.308003
744.522976	79.619998
746.451792	79.916796
748.380608	80.134482
750.309424	80.318372
752.238240	80.553904
754.167056	80.778032
756.095872	80.913335
758.024688	81.082162
759.953504	81.280386
761.882320	81.483540
763.811136	81.665659
765.739952	81.801164
767.668768	81.933991
769.597584	82.066338
771.526400	82.203682
773.455216	82.343670
775.384032	82.436142
777.312848	82.564626
779.241664	82.686903
781.170480	82.809378
783.099296	82.935542
785.028112	83.062622
786.956928	83.220137
788.885744	83.290741
790.814560	83.371037

792.743376	83.516721
794.672192	83.619756
796.601008	83.672959
798.529824	83.725270
800.458640	83.880579
802.387456	84.069676
804.316272	84.168478
806.245088	84.276607
808.173904	84.399738
810.102720	84.501688
812.031536	84.617453
813.960352	84.747771
815.889168	84.885259
817.817984	84.991559
819.746800	85.007344
821.675616	85.106543
823.604432	85.261559
825.533248	85.337118
827.462064	85.413677
829.390880	85.526160
831.319696	85.626410
833.248512	85.677142
835.177328	85.773547
837.106144	85.887574
839.034960	85.972365
840.963776	86.049987

842.892592	86.128871
844.821408	86.199695
846.750224	86.316141
848.679040	86.458980
850.607856	86.538940
852.536672	86.615338
854.465488	86.736645
856.394304	86.897769
858.323120	87.059509
860.251936	87.171172
862.180752	87.279178
864.109568	87.430794
866.038384	87.619508
867.967200	87.769591
869.896016	87.915515
871.824832	88.086033
873.753648	88.200919
875.682464	88.311860
877.611280	88.433117
879.540096	88.573831
881.468912	88.722626
883.397728	88.818212
885.326544	88.927373
887.255360	89.046901
889.184176	89.182478
891.112992	89.285516

893.041808	89.311427
894.970624	89.413260
896.899440	89.520090
898.828256	89.602814
900.757072	89.712783
902.685888	89.801229
904.614704	89.870679
906.543520	89.963129
908.472336	90.056394
910.401152	90.140422
912.329968	90.223704
914.258784	90.298379
916.187600	90.404304
918.116416	90.526047
920.045232	90.569701
921.974048	90.613230
923.902864	90.696127
925.831680	90.767827
927.760496	90.846476
929.689312	90.921698
931.618128	91.009622
933.546944	91.098757
935.475760	91.193554
937.404576	91.234126
939.333392	91.312265
941.262208	91.401661

943.191024	91.425678
945.119840	91.481145
947.048656	91.564476
948.977472	91.656887
950.906288	91.765793
952.835104	91.803682
954.763920	91.850051
956.692736	91.927611
958.621552	91.980726
960.550368	92.063660
962.479184	92.125781
964.408000	92.148373
966.336816	92.211731
968.265632	92.309490
970.194448	92.376624
972.123264	92.425741
974.052080	92.478299
975.980896	92.530801
977.909712	92.548991
979.838528	92.570875
981.767344	92.658875
983.696160	92.767186
985.624976	92.828609
987.553792	92.846374
989.482608	92.908233
991.411424	93.000484

993.340240	93.038444
995.269056	93.075618
997.197872	93.068672
999.126688	93.060247
1001.055504	93.145969
1002.984320	93.172036
1004.913136	93.159556
1006.841952	93.230024
1008.770768	93.215195
1010.699584	93.212267
1012.628400	93.278063
1014.557216	93.315685
1016.486032	93.357954
1018.414848	93.369160
1020.343664	93.390916
1022.272480	93.408877
1024.201296	93.422343
1026.130112	93.445107
1028.058928	93.431919
1029.987744	93.386026
1031.916560	93.381756
1033.845376	93.392133
1035.774192	93.354781
1037.703008	93.295986
1039.631824	93.243415
1041.560640	93.256320

1043.489456	93.274678
1045.418272	93.300881
1047.347088	93.273757
1049.275904	93.192973
1051.204720	93.194670
1053.133536	93.183384
1055.062352	93.184866
1056.991168	93.180321
1058.919984	93.133446
1060.848800	93.119785
1062.777616	93.088449
1064.706432	93.023159
1066.635248	93.001222
1068.564064	93.017832
1070.492880	93.025662
1072.421696	93.020503
1074.350512	92.985484
1076.279328	92.971646
1078.208144	92.965783
1080.136960	93.006674
1082.065776	93.119314
1083.994592	93.158631
1085.923408	93.201527
1087.852224	93.282469
1089.781040	93.344247
1091.709856	93.400503

1093.638672	93.446344
1095.567488	93.491591
1097.496304	93.525777
1099.425120	93.606896
1101.353936	93.693325
1103.282752	93.730099
1105.211568	93.786236
1107.140384	93.887103
1109.069200	93.978228
1110.998016	94.012711
1112.926832	94.038779
1114.855648	94.078892
1116.784464	94.131133
1118.713280	94.196643
1120.642096	94.278539
1122.570912	94.326483
1124.499728	94.343781
1126.428544	94.389823
1128.357360	94.405981
1130.286176	94.420227
1132.214992	94.459438
1134.143808	94.508681
1136.072624	94.559696
1138.001440	94.613187
1139.930256	94.638552
1141.859072	94.646504

1143.787888	94.657537
1145.716704	94.694306
1147.645520	94.717663
1149.574336	94.711553
1151.503152	94.719417
1153.431968	94.761891
1155.360784	94.841695
1157.289600	94.859027
1159.218416	94.845998
1161.147232	94.853871
1163.076048	94.868474
1165.004864	94.884757
1166.933680	94.888041
1168.862496	94.898179
1170.791312	94.933708
1172.720128	94.963426
1174.648944	94.959273
1176.577760	94.944105
1178.506576	94.929814
1180.435392	94.937375
1182.364208	94.952187
1184.293024	94.942520
1186.221840	94.944469
1188.150656	94.950185
1190.079472	94.927965
1192.008288	94.948997

1193.937104	94.985623
1195.865920	94.974149
1197.794736	94.989144
1199.723552	95.020866
1201.652368	95.012592
1203.581184	94.976403
1205.510000	94.969213
1207.438816	94.950419
1209.367632	94.945140
1211.296448	94.984621
1213.225264	94.949525
1215.154080	94.915185
1217.082896	94.897778
1219.011712	94.873742
1220.940528	94.863674
1222.869344	94.853616
1224.798160	94.842053
1226.726976	94.825100
1228.655792	94.800318
1230.584608	94.745514
1232.513424	94.726332
1234.442240	94.729394
1236.371056	94.720315
1238.299872	94.722672
1240.228688	94.696874
1242.157504	94.648537

1244.086320	94.599927
1246.015136	94.574329
1247.943952	94.569547
1249.872768	94.535760
1251.801584	94.516467
1253.730400	94.484781
1255.659216	94.448046
1257.588032	94.406282
1259.516848	94.374490
1261.445664	94.367187
1263.374480	94.330789
1265.303296	94.298751
1267.232112	94.247261
1269.160928	94.209185
1271.089744	94.167284
1273.018560	94.112973
1274.947376	94.075707
1276.876192	94.037307
1278.805008	93.979387
1280.733824	93.898868
1282.662640	93.823591
1284.591456	93.764473
1286.520272	93.729415
1288.449088	93.672467
1290.377904	93.559230
1292.306720	93.468186

1294.235536	93.407385
1296.164352	93.324237
1298.093168	93.210375
1300.021984	93.108416
1301.950800	93.002556
1303.879616	92.879378
1305.808432	92.779311
1307.737248	92.669544
1309.666064	92.514392
1311.594880	92.399185
1313.523696	92.299792
1315.452512	92.134344
1317.381328	91.993648
1319.310144	91.830921
1321.238960	91.615233
1323.167776	91.429832
1325.096592	91.254747
1327.025408	91.085819
1328.954224	90.919998
1330.883040	90.716566
1332.811856	90.510413
1334.740672	90.348707
1336.669488	90.195771
1338.598304	90.083137
1340.527120	89.867733
1342.455936	89.575386

1344.384752	89.359645
1346.313568	89.171406
1348.242384	89.000786
1350.171200	88.842623
1352.100016	88.673346
1354.028832	88.552431
1355.957648	88.453762
1357.886464	88.307860
1359.815280	88.204342
1361.744096	88.254141
1363.672912	88.188638
1365.601728	87.979754
1367.530544	87.932165
1369.459360	87.887923
1371.388176	87.823428
1373.316992	87.905182
1375.245808	87.862016
1377.174624	87.740143
1379.103440	87.716931
1381.032256	87.717233
1382.961072	87.714283
1384.889888	87.754726
1386.818704	87.873253
1388.747520	87.858653
1390.676336	87.802863
1392.605152	87.886303

1394.533968	88.040324
1396.462784	88.094306
1398.391600	88.161789
1400.320416	88.124784
1402.249232	88.101264
1404.178048	88.261055
1406.106864	88.309188
1408.035680	88.317453
1409.964496	88.405093
1411.893312	88.486161
1413.822128	88.548876
1415.750944	88.687423
1417.679760	88.947424
1419.608576	89.019473
1421.537392	88.886727
1423.466208	88.952771
1425.395024	88.951377
1427.323840	88.956579
1429.252656	89.062350
1431.181472	89.081718
1433.110288	89.076932
1435.039104	89.223452
1436.967920	89.330531
1438.896736	89.117789
1440.825552	89.035617
1442.754368	89.049306

1444.683184	89.047313
1446.612000	89.123673
1448.540816	89.110686
1450.469632	89.033406
1452.398448	89.013845
1454.327264	89.093895
1456.256080	89.367165
1458.184896	89.309218
1460.113712	89.022802
1462.042528	88.934121
1463.971344	89.024989
1465.900160	89.035056
1467.828976	88.930508
1469.757792	88.965850
1471.686608	89.187147
1473.615424	89.226299
1475.544240	89.129263
1477.473056	89.006935
1479.401872	88.890192
1481.330688	88.953419
1483.259504	88.961730
1485.188320	89.027025
1487.117136	89.190653
1489.045952	89.378815
1490.974768	89.400915
1492.903584	89.255057

1494.832400	89.373880
1496.761216	89.600196
1498.690032	89.634872
1500.618848	89.636723
1502.547664	89.689149
1504.476480	89.929117
1506.405296	90.461748
1508.334112	90.524895
1510.262928	90.376486
1512.191744	90.396361
1514.120560	90.458712
1516.049376	90.712970
1517.978192	90.842609
1519.907008	90.971649
1521.835824	91.178463
1523.764640	91.128191
1525.693456	91.247877
1527.622272	91.347700
1529.551088	91.351569
1531.479904	91.498168
1533.408720	91.743775
1535.337536	91.734363
1537.266352	91.880146
1539.195168	92.340696
1541.123984	92.424501
1543.052800	92.415427

1544.981616	92.425601
1546.910432	92.219558
1548.839248	92.292991
1550.768064	92.372848
1552.696880	92.335650
1554.625696	92.453738
1556.554512	92.553731
1558.483328	92.909997
1560.412144	92.754968
1562.340960	92.462308
1564.269776	92.491717
1566.198592	92.509900
1568.127408	92.615154
1570.056224	92.717068
1571.985040	92.577877
1573.913856	92.612097
1575.842672	92.775853
1577.771488	92.643489
1579.700304	92.502860
1581.629120	92.461752
1583.557936	92.370832
1585.486752	92.293448
1587.415568	92.252182
1589.344384	92.202712
1591.273200	92.120557
1593.202016	92.033569

1595.130832	91.950788
1597.059648	91.859601
1598.988464	91.771560
1600.917280	91.704427
1602.846096	91.656525
1604.774912	91.553387
1606.703728	91.474457
1608.632544	91.405157
1610.561360	91.297346
1612.490176	91.189526
1614.418992	91.165662
1616.347808	91.209185
1618.276624	91.035467
1620.205440	90.877955
1622.134256	90.942092
1624.063072	90.916244
1625.991888	90.796353
1627.920704	90.811232
1629.849520	90.756686
1631.778336	90.709305
1633.707152	90.823718
1635.635968	91.024517
1637.564784	90.946973
1639.493600	90.839131
1641.422416	90.875727
1643.351232	90.986839

1645.280048	91.233891
1647.208864	91.455308
1649.137680	91.340804
1651.066496	91.457505
1652.995312	91.939009
1654.924128	91.775519
1656.852944	91.639793
1658.781760	91.731686
1660.710576	91.882105
1662.639392	92.092454
1664.568208	92.187129
1666.497024	92.282256
1668.425840	92.509007
1670.354656	92.710346
1672.283472	92.723741
1674.212288	92.912557
1676.141104	93.092085
1678.069920	93.111088
1679.998736	93.308732
1681.927552	93.529820
1683.856368	93.887472
1685.785184	93.845474
1687.714000	93.808194
1689.642816	93.961015
1691.571632	93.960961
1693.500448	94.089863

1695.429264	94.440877
1697.358080	94.520158
1699.286896	94.697677
1701.215712	94.845616
1703.144528	94.701415
1705.073344	94.850781
1707.002160	94.825817
1708.930976	94.810020
1710.859792	94.858522
1712.788608	94.979282
1714.717424	95.189269
1716.646240	95.350028
1718.575056	95.352291
1720.503872	95.169837
1722.432688	95.182556
1724.361504	95.229910
1726.290320	95.193548
1728.219136	95.299941
1730.147952	95.430881
1732.076768	95.527831
1734.005584	95.731790
1735.934400	95.521865
1737.863216	95.450030
1739.792032	95.512161
1741.720848	95.491370
1743.649664	95.545180

1745.578480	95.540824
1747.507296	95.633246
1749.436112	95.695549
1751.364928	95.668456
1753.293744	95.495823
1755.222560	95.470207
1757.151376	95.555835
1759.080192	95.503167
1761.009008	95.537689
1762.937824	95.531482
1764.866640	95.428844
1766.795456	95.534806
1768.724272	95.597569
1770.653088	95.619309
1772.581904	95.653382
1774.510720	95.549558
1776.439536	95.506956
1778.368352	95.463533
1780.297168	95.485880
1782.225984	95.464021
1784.154800	95.494710
1786.083616	95.495295
1788.012432	95.461858
1789.941248	95.602745
1791.870064	95.701025
1793.798880	95.572943

1795.727696	95.502485
1797.656512	95.519899
1799.585328	95.598578
1801.514144	95.655791
1803.442960	95.568323
1805.371776	95.488652
1807.300592	95.483776
1809.229408	95.515076
1811.158224	95.570225
1813.087040	95.561426
1815.015856	95.569125
1816.944672	95.587459
1818.873488	95.492827
1820.802304	95.469446
1822.731120	95.524793
1824.659936	95.615542
1826.588752	95.612514
1828.517568	95.654481
1830.446384	95.657238
1832.375200	95.491155
1834.304016	95.504183
1836.232832	95.559812
1838.161648	95.555030
1840.090464	95.534645
1842.019280	95.595759
1843.948096	95.745396

1845.876912	95.626441
1847.805728	95.496253
1849.734544	95.502411
1851.663360	95.495033
1853.592176	95.472845
1855.520992	95.491352
1857.449808	95.498011
1859.378624	95.508994
1861.307440	95.554625
1863.236256	95.623346
1865.165072	95.622774
1867.093888	95.627209
1869.022704	95.693209
1870.951520	95.634825
1872.880336	95.569087
1874.809152	95.571091
1876.737968	95.549594
1878.666784	95.470477
1880.595600	95.493357
1882.524416	95.547229
1884.453232	95.546257
1886.382048	95.609023
1888.310864	95.640529
1890.239680	95.565950
1892.168496	95.500595
1894.097312	95.592738

1896.026128	95.683618
1897.954944	95.642020
1899.883760	95.640952
1901.812576	95.608689
1903.741392	95.558501
1905.670208	95.526950
1907.599024	95.602218
1909.527840	95.631624
1911.456656	95.565426
1913.385472	95.590026
1915.314288	95.527139
1917.243104	95.608398
1919.171920	95.791599
1921.100736	95.781313
1923.029552	95.797794
1924.958368	95.816063
1926.887184	95.686892
1928.816000	95.698068
1930.744816	95.767908
1932.673632	95.728775
1934.602448	95.693833
1936.531264	95.681719
1938.460080	95.877365
1940.388896	95.916222
1942.317712	95.901428
1944.246528	95.991467

1946.175344	95.888248
1948.104160	95.802233
1950.032976	95.804130
1951.961792	95.743708
1953.890608	95.913207
1955.819424	96.143180
1957.748240	96.068982
1959.677056	96.065246
1961.605872	96.215765
1963.534688	96.218222
1965.463504	96.155582
1967.392320	96.255273
1969.321136	96.348161
1971.249952	96.196925
1973.178768	96.203519
1975.107584	96.341203
1977.036400	96.164364
1978.965216	95.762649
1980.894032	95.746856
1982.822848	95.834684
1984.751664	95.869178
1986.680480	95.932686
1988.609296	96.088687
1990.538112	96.241857
1992.466928	96.050562
1994.395744	96.140773

1996.324560	96.250519
1998.253376	96.050960
2000.182192	96.232159
2002.111008	96.409246
2004.039824	96.425545
2005.968640	96.538051
2007.897456	96.619330
2009.826272	96.500930
2011.755088	96.206539
2013.683904	96.257037
2015.612720	96.455515
2017.541536	96.504061
2019.470352	96.317681
2021.399168	96.229514
2023.327984	96.307762
2025.256800	96.329613
2027.185616	96.157164
2029.114432	96.311227
2031.043248	96.466727
2032.972064	96.193988
2034.900880	95.899125
2036.829696	95.868560
2038.758512	96.000969
2040.687328	95.895374
2042.616144	95.586984
2044.544960	95.505791

2046.473776	95.600818
2048.402592	95.533557
2050.331408	95.411626
2052.260224	95.444727
2054.189040	95.439974
2056.117856	95.508563
2058.046672	95.457089
2059.975488	95.358026
2061.904304	95.354082
2063.833120	95.245334
2065.761936	95.240937
2067.690752	95.120380
2069.619568	94.988478
2071.548384	95.028220
2073.477200	95.103062
2075.406016	95.139884
2077.334832	95.150488
2079.263648	95.207145
2081.192464	95.278064
2083.121280	95.188986
2085.050096	95.100534
2086.978912	95.085584
2088.907728	95.068454
2090.836544	95.177770
2092.765360	95.348477
2094.694176	95.378091

2096.622992	95.199850
2098.551808	95.095330
2100.480624	95.067208
2102.409440	95.018889
2104.338256	95.012575
2106.267072	95.006172
2108.195888	95.020397
2110.124704	95.108761
2112.053520	95.101808
2113.982336	95.036728
2115.911152	95.055057
2117.839968	95.028012
2119.768784	95.056181
2121.697600	95.191690
2123.626416	95.319862
2125.555232	95.212115
2127.484048	95.114638
2129.412864	95.307837
2131.341680	95.535854
2133.270496	95.661081
2135.199312	95.664762
2137.128128	95.618740
2139.056944	95.577181
2140.985760	95.596873
2142.914576	95.710266
2144.843392	95.954883

2146.772208	96.312282
2148.701024	96.415517
2150.629840	96.245955
2152.558656	96.278319
2154.487472	96.303975
2156.416288	96.364424
2158.345104	96.074593
2160.273920	95.528423
2162.202736	95.628955
2164.131552	95.713296
2166.060368	95.598902
2167.989184	95.579155
2169.918000	95.761651
2171.846816	95.911654
2173.775632	96.086301
2175.704448	96.136084
2177.633264	95.938858
2179.562080	95.870202
2181.490896	95.646937
2183.419712	95.561078
2185.348528	95.796197
2187.277344	95.759545
2189.206160	95.713220
2191.134976	95.853871
2193.063792	95.694891
2194.992608	95.434465

2196.921424	95.331790
2198.850240	95.481181
2200.779056	95.531057
2202.707872	95.417816
2204.636688	95.506945
2206.565504	95.389229
2208.494320	95.187386
2210.423136	95.191346
2212.351952	95.343320
2214.280768	95.489795
2216.209584	95.447215
2218.138400	95.349084
2220.067216	95.133582
2221.996032	95.130843
2223.924848	95.376333
2225.853664	95.242799
2227.782480	95.172275
2229.711296	95.167470
2231.640112	95.051007
2233.568928	95.138158
2235.497744	95.259035
2237.426560	95.249036
2239.355376	95.089129
2241.284192	95.044060
2243.213008	94.975841
2245.141824	94.887459

2247.070640	94.937749
2248.999456	95.013580
2250.928272	94.949169
2252.857088	94.913366
2254.785904	94.940577
2256.714720	94.868195
2258.643536	94.813003
2260.572352	94.774840
2262.501168	94.836583
2264.429984	94.808146
2266.358800	94.697062
2268.287616	94.750618
2270.216432	94.883264
2272.145248	94.801067
2274.074064	94.666099
2276.002880	94.654263
2277.931696	94.645785
2279.860512	94.611153
2281.789328	94.508960
2283.718144	94.399116
2285.646960	94.358349
2287.575776	94.398310
2289.504592	94.455327
2291.433408	94.419389
2293.362224	94.353582
2295.291040	94.366233

2297.219856	94.402617
2299.148672	94.390808
2301.077488	94.353509
2303.006304	94.374775
2304.935120	94.340452
2306.863936	94.221225
2308.792752	94.177661
2310.721568	94.148607
2312.650384	94.064446
2314.579200	94.037345
2316.508016	94.061632
2318.436832	93.961725
2320.365648	93.769337
2322.294464	93.599112
2324.223280	93.510318
2326.152096	93.594879
2328.080912	93.657034
2330.009728	93.684262
2331.938544	93.702681
2333.867360	93.660201
2335.796176	93.590134
2337.724992	93.491174
2339.653808	93.403320
2341.582624	93.435153
2343.511440	93.511613
2345.440256	93.555899

2347.369072	93.687623
2349.297888	93.730534
2351.226704	93.587320
2353.155520	93.430521
2355.084336	93.400862
2357.013152	93.327240
2358.941968	93.224529
2360.870784	93.184011
2362.799600	93.218975
2364.728416	93.309152
2366.657232	93.424169
2368.586048	93.583297
2370.514864	93.726797
2372.443680	93.898086
2374.372496	94.062481
2376.301312	94.183260
2378.230128	94.263915
2380.158944	94.288017
2382.087760	94.351273
2384.016576	94.420107
2385.945392	94.434468
2387.874208	94.429616
2389.803024	94.437963
2391.731840	94.412073
2393.660656	94.408867
2395.589472	94.372751

2397.518288	94.323564
2399.447104	94.349440
2401.375920	94.372377
2403.304736	94.378596
2405.233552	94.377289
2407.162368	94.382662
2409.091184	94.366014
2411.020000	94.362749
2412.948816	94.346694
2414.877632	94.347193
2416.806448	94.378195
2418.735264	94.389592
2420.664080	94.398498
2422.592896	94.365507
2424.521712	94.361822
2426.450528	94.362633
2428.379344	94.345952
2430.308160	94.319466
2432.236976	94.283769
2434.165792	94.287227
2436.094608	94.302337
2438.023424	94.320418
2439.952240	94.295320
2441.881056	94.241431
2443.809872	94.198528
2445.738688	94.199474

2447.667504	94.205844
2449.596320	94.201549
2451.525136	94.223315
2453.453952	94.214330
2455.382768	94.177296
2457.311584	94.191971
2459.240400	94.239479
2461.169216	94.232366
2463.098032	94.190902
2465.026848	94.162755
2466.955664	94.198804
2468.884480	94.239645
2470.813296	94.232844
2472.742112	94.206508
2474.670928	94.188314
2476.599744	94.169313
2478.528560	94.129320
2480.457376	94.111326
2482.386192	94.118744
2484.315008	94.121207
2486.243824	94.132973
2488.172640	94.091868
2490.101456	94.065556
2492.030272	94.116039
2493.959088	94.100896
2495.887904	94.081001

2497.816720	94.061135
2499.745536	94.030877
2501.674352	93.957694
2503.603168	93.970892
2505.531984	94.019272
2507.460800	93.982216
2509.389616	93.967216
2511.318432	93.946944
2513.247248	93.950848
2515.176064	93.930495
2517.104880	93.916117
2519.033696	93.897032
2520.962512	93.839450
2522.891328	93.840145
2524.820144	93.916203
2526.748960	93.946076
2528.677776	93.878040
2530.606592	93.833157
2532.535408	93.821490
2534.464224	93.828512
2536.393040	93.822989
2538.321856	93.808140
2540.250672	93.795971
2542.179488	93.749425
2544.108304	93.759106
2546.037120	93.749933

2547.965936	93.729782
2549.894752	93.698981
2551.823568	93.664864
2553.752384	93.690701
2555.681200	93.664355
2557.610016	93.621986
2559.538832	93.629752
2561.467648	93.607906
2563.396464	93.561667
2565.325280	93.578176
2567.254096	93.596652
2569.182912	93.583936
2571.111728	93.543254
2573.040544	93.496693
2574.969360	93.497134
2576.898176	93.466213
2578.826992	93.459729
2580.755808	93.458701
2582.684624	93.411108
2584.613440	93.371647
2586.542256	93.372648
2588.471072	93.398005
2590.399888	93.371380
2592.328704	93.336909
2594.257520	93.313246
2596.186336	93.302993

2598.115152	93.288040
2600.043968	93.263115
2601.972784	93.241072
2603.901600	93.211052
2605.830416	93.155188
2607.759232	93.141143
2609.688048	93.149189
2611.616864	93.116221
2613.545680	93.085375
2615.474496	93.036802
2617.403312	92.989329
2619.332128	92.971901
2621.260944	92.957310
2623.189760	92.927057
2625.118576	92.914902
2627.047392	92.918033
2628.976208	92.900516
2630.905024	92.858184
2632.833840	92.814334
2634.762656	92.783870
2636.691472	92.790834
2638.620288	92.795736
2640.549104	92.746084
2642.477920	92.722823
2644.406736	92.713551
2646.335552	92.686745

2648.264368	92.673341
2650.193184	92.642700
2652.122000	92.598114
2654.050816	92.555674
2655.979632	92.538378
2657.908448	92.540808
2659.837264	92.556583
2661.766080	92.559831
2663.694896	92.548534
2665.623712	92.543684
2667.552528	92.544947
2669.481344	92.548347
2671.410160	92.531343
2673.338976	92.516515
2675.267792	92.492842
2677.196608	92.473916
2679.125424	92.451925
2681.054240	92.423487
2682.983056	92.411000
2684.911872	92.406940
2686.840688	92.414572
2688.769504	92.403826
2690.698320	92.386868
2692.627136	92.385307
2694.555952	92.372678
2696.484768	92.335583

2698.413584	92.306537
2700.342400	92.298998
2702.271216	92.287565
2704.200032	92.266695
2706.128848	92.216564
2708.057664	92.194620
2709.986480	92.211259
2711.915296	92.208975
2713.844112	92.187198
2715.772928	92.178956
2717.701744	92.163252
2719.630560	92.117699
2721.559376	92.083029
2723.488192	92.066772
2725.417008	92.065814
2727.345824	92.053657
2729.274640	92.010148
2731.203456	91.990159
2733.132272	92.017615
2735.061088	92.005102
2736.989904	91.955877
2738.918720	91.933266
2740.847536	91.927225
2742.776352	91.915593
2744.705168	91.893827
2746.633984	91.871979

2748.562800	91.862293
2750.491616	91.836636
2752.420432	91.810480
2754.349248	91.765668
2756.278064	91.723789
2758.206880	91.700090
2760.135696	91.695060
2762.064512	91.698213
2763.993328	91.676644
2765.922144	91.659383
2767.850960	91.641331
2769.779776	91.626582
2771.708592	91.611295
2773.637408	91.594745
2775.566224	91.552669
2777.495040	91.527732
2779.423856	91.540138
2781.352672	91.506261
2783.281488	91.473951
2785.210304	91.493774
2787.139120	91.480100
2789.067936	91.432577
2790.996752	91.407867
2792.925568	91.378205
2794.854384	91.342687
2796.783200	91.327185

2798.712016	91.293007
2800.640832	91.289007
2802.569648	91.302151
2804.498464	91.276661
2806.427280	91.242241
2808.356096	91.211003
2810.284912	91.211716
2812.213728	91.196803
2814.142544	91.150206
2816.071360	91.116988
2818.000176	91.105963
2819.928992	91.096454
2821.857808	91.060983
2823.786624	91.030490
2825.715440	91.012933
2827.644256	90.993165
2829.573072	90.945439
2831.501888	90.901308
2833.430704	90.897978
2835.359520	90.893488
2837.288336	90.868227
2839.217152	90.874963
2841.145968	90.897973
2843.074784	90.914108
2845.003600	90.930884
2846.932416	91.003743

2848.861232	91.121829
2850.790048	91.082721
2852.718864	90.926584
2854.647680	90.815052
2856.576496	90.741364
2858.505312	90.678294
2860.434128	90.605601
2862.362944	90.553694
2864.291760	90.531371
2866.220576	90.487425
2868.149392	90.440785
2870.078208	90.418817
2872.007024	90.401394
2873.935840	90.374467
2875.864656	90.342851
2877.793472	90.321146
2879.722288	90.310815
2881.651104	90.281971
2883.579920	90.239407
2885.508736	90.208584
2887.437552	90.183540
2889.366368	90.154664
2891.295184	90.124814
2893.224000	90.066915
2895.152816	90.030515
2897.081632	90.035345

2899.010448	90.026424
2900.939264	90.031221
2902.868080	90.047033
2904.796896	90.037467
2906.725712	90.031694
2908.654528	90.054405
2910.583344	90.112109
2912.512160	90.242005
2914.440976	90.362986
2916.369792	90.398982
2918.298608	90.339093
2920.227424	90.199484
2922.156240	90.055642
2924.085056	89.952441
2926.013872	89.867521
2927.942688	89.812703
2929.871504	89.755371
2931.800320	89.683388
2933.729136	89.643527
2935.657952	89.598649
2937.586768	89.577217
2939.515584	89.535441
2941.444400	89.491940
2943.373216	89.462861
2945.302032	89.410497
2947.230848	89.383424

2949.159664	89.332307
2951.088480	89.316321
2953.017296	89.308060
2954.946112	89.277843
2956.874928	89.241213
2958.803744	89.182326
2960.732560	89.146755
2962.661376	89.133272
2964.590192	89.079957
2966.519008	89.032412
2968.447824	88.996858
2970.376640	88.944701
2972.305456	88.911880
2974.234272	88.894985
2976.163088	88.867806
2978.091904	88.800581
2980.020720	88.789770
2981.949536	88.755866
2983.878352	88.697713
2985.807168	88.676925
2987.735984	88.654934
2989.664800	88.640674
2991.593616	88.624657
2993.522432	88.600377
2995.451248	88.576306
2997.380064	88.530620

2999.308880	88.466942
3001.237696	88.427225
3003.166512	88.430693
3005.095328	88.425857
3007.024144	88.384703
3008.952960	88.347837
3010.881776	88.357560
3012.810592	88.351689
3014.739408	88.306192
3016.668224	88.296811
3018.597040	88.254866
3020.525856	88.180375
3022.454672	88.162638
3024.383488	88.137465
3026.312304	88.125020
3028.241120	88.108918
3030.169936	88.055894
3032.098752	88.026958
3034.027568	87.992400
3035.956384	87.996461
3037.885200	87.946655
3039.814016	87.868089
3041.742832	87.862202
3043.671648	87.832119
3045.600464	87.804317
3047.529280	87.766342

3049.458096	87.710084
3051.386912	87.649193
3053.315728	87.630418
3055.244544	87.654733
3057.173360	87.591146
3059.102176	87.519245
3061.030992	87.481595
3062.959808	87.458250
3064.888624	87.467701
3066.817440	87.452278
3068.746256	87.381946
3070.675072	87.300132
3072.603888	87.270131
3074.532704	87.243815
3076.461520	87.171256
3078.390336	87.125858
3080.319152	87.123012
3082.247968	87.057934
3084.176784	86.986910
3086.105600	86.964071
3088.034416	86.915560
3089.963232	86.887220
3091.892048	86.864063
3093.820864	86.810858
3095.749680	86.761813
3097.678496	86.735369

3099.607312	86.685610
3101.536128	86.631737
3103.464944	86.576438
3105.393760	86.524839
3107.322576	86.500258
3109.251392	86.434242
3111.180208	86.375004
3113.109024	86.362649
3115.037840	86.356175
3116.966656	86.298053
3118.895472	86.243511
3120.824288	86.214042
3122.753104	86.183952
3124.681920	86.130899
3126.610736	86.066878
3128.539552	86.054138
3130.468368	86.008236
3132.397184	85.919713
3134.326000	85.869091
3136.254816	85.854253
3138.183632	85.823768
3140.112448	85.750333
3142.041264	85.666949
3143.970080	85.632970
3145.898896	85.624242
3147.827712	85.565035

3149.756528	85.484606
3151.685344	85.477540
3153.614160	85.435751
3155.542976	85.344991
3157.471792	85.305338
3159.400608	85.278535
3161.329424	85.226781
3163.258240	85.158249
3165.187056	85.106017
3167.115872	85.068338
3169.044688	85.053858
3170.973504	84.995878
3172.902320	84.932407
3174.831136	84.903568
3176.759952	84.883388
3178.688768	84.819355
3180.617584	84.722528
3182.546400	84.701965
3184.475216	84.699802
3186.404032	84.632113
3188.332848	84.560800
3190.261664	84.516950
3192.190480	84.479397
3194.119296	84.443794
3196.048112	84.381766
3197.976928	84.337222

3199.905744	84.275783
3201.834560	84.259312
3203.763376	84.251509
3205.692192	84.195096
3207.621008	84.161113
3209.549824	84.091564
3211.478640	84.033491
3213.407456	84.028438
3215.336272	83.991182
3217.265088	83.939286
3219.193904	83.921237
3221.122720	83.860398
3223.051536	83.795450
3224.980352	83.768340
3226.909168	83.703682
3228.837984	83.695468
3230.766800	83.687915
3232.695616	83.637494
3234.624432	83.639828
3236.553248	83.587562
3238.482064	83.530783
3240.410880	83.520365
3242.339696	83.508851
3244.268512	83.484131
3246.197328	83.456487
3248.126144	83.410673

3250.054960	83.354051
3251.983776	83.331160
3253.912592	83.300825
3255.841408	83.273603
3257.770224	83.254550
3259.699040	83.207872
3261.627856	83.166255
3263.556672	83.131645
3265.485488	83.116661
3267.414304	83.112422
3269.343120	83.065503
3271.271936	83.013748
3273.200752	82.999420
3275.129568	82.968307
3277.058384	82.883702
3278.987200	82.855933
3280.916016	82.897397
3282.844832	82.875353
3284.773648	82.804340
3286.702464	82.776720
3288.631280	82.763845
3290.560096	82.734237
3292.488912	82.693973
3294.417728	82.648216
3296.346544	82.631341
3298.275360	82.591659

3300.204176	82.531198
3302.132992	82.535543
3304.061808	82.553632
3305.990624	82.547638
3307.919440	82.521527
3309.848256	82.463673
3311.777072	82.423070
3313.705888	82.431660
3315.634704	82.387217
3317.563520	82.327225
3319.492336	82.338659
3321.421152	82.316351
3323.349968	82.272483
3325.278784	82.252300
3327.207600	82.225924
3329.136416	82.222411
3331.065232	82.231201
3332.994048	82.214546
3334.922864	82.205341
3336.851680	82.197794
3338.780496	82.224240
3340.709312	82.232643
3342.638128	82.228445
3344.566944	82.242564
3346.495760	82.212044
3348.424576	82.195332

3350.353392	82.203929
3352.282208	82.212862
3354.211024	82.236098
3356.139840	82.275516
3358.068656	82.281446
3359.997472	82.281305
3361.926288	82.284099
3363.855104	82.280918
3365.783920	82.331612
3367.712736	82.378869
3369.641552	82.414006
3371.570368	82.409601
3373.499184	82.420620
3375.428000	82.474801
3377.356816	82.483739
3379.285632	82.503122
3381.214448	82.573334
3383.143264	82.618859
3385.072080	82.615157
3387.000896	82.641607
3388.929712	82.708356
3390.858528	82.799179
3392.787344	82.845364
3394.716160	82.865894
3396.644976	82.952137
3398.573792	83.027403

3400.502608	83.050223
3402.431424	83.096829
3404.360240	83.137587
3406.289056	83.190842
3408.217872	83.242726
3410.146688	83.287610
3412.075504	83.363824
3414.004320	83.450400
3415.933136	83.504640
3417.861952	83.524030
3419.790768	83.581140
3421.719584	83.664519
3423.648400	83.718794
3425.577216	83.775147
3427.506032	83.822292
3429.434848	83.900259
3431.363664	84.030753
3433.292480	84.097809
3435.221296	84.170386
3437.150112	84.233602
3439.078928	84.282024
3441.007744	84.366541
3442.936560	84.430006
3444.865376	84.521974
3446.794192	84.679554
3448.723008	84.728984

3450.651824	84.730082
3452.580640	84.799211
3454.509456	84.885274
3456.438272	84.985778
3458.367088	85.065786
3460.295904	85.141298
3462.224720	85.182186
3464.153536	85.283362
3466.082352	85.423961
3468.011168	85.485775
3469.939984	85.540251
3471.868800	85.633274
3473.797616	85.704041
3475.726432	85.776899
3477.655248	85.890071
3479.584064	86.010568
3481.512880	86.113755
3483.441696	86.176724
3485.370512	86.246754
3487.299328	86.376616
3489.228144	86.465248
3491.156960	86.585652
3493.085776	86.706050
3495.014592	86.797240
3496.943408	86.880598
3498.872224	86.971594

3500.801040	87.126548
3502.729856	87.221500
3504.658672	87.304847
3506.587488	87.350126
3508.516304	87.431054
3510.445120	87.559385
3512.373936	87.632013
3514.302752	87.756561
3516.231568	87.888295
3518.160384	87.990854
3520.089200	88.097365
3522.018016	88.245633
3523.946832	88.403986
3525.875648	88.494436
3527.804464	88.627332
3529.733280	88.713543
3531.662096	88.780885
3533.590912	88.895458
3535.519728	89.010205
3537.448544	89.122498
3539.377360	89.206988
3541.306176	89.317535
3543.234992	89.470297
3545.163808	89.640449
3547.092624	89.748122
3549.021440	89.774257

3550.950256	89.867462
3552.879072	89.977358
3554.807888	90.025710
3556.736704	90.151503
3558.665520	90.288273
3560.594336	90.373630
3562.523152	90.466649
3564.451968	90.650653
3566.380784	90.960181
3568.309600	91.088707
3570.238416	90.916386
3572.167232	90.929443
3574.096048	91.099914
3576.024864	91.209027
3577.953680	91.308673
3579.882496	91.403336
3581.811312	91.468096
3583.740128	91.611506
3585.668944	91.891973
3587.597760	92.151624
3589.526576	92.157551
3591.455392	92.106566
3593.384208	92.251909
3595.313024	92.403028
3597.241840	92.412039
3599.170656	92.475655

3601.099472	92.617762
3603.028288	92.680730
3604.957104	92.779424
3606.885920	93.031742
3608.814736	93.211711
3610.743552	93.266285
3612.672368	93.430099
3614.601184	93.518741
3616.530000	93.552002
3618.458816	93.805914
3620.387632	93.908558
3622.316448	93.779121
3624.245264	93.831159
3626.174080	94.040091
3628.102896	94.437109
3630.031712	94.532139
3631.960528	94.357601
3633.889344	94.499416
3635.818160	94.557639
3637.746976	94.560884
3639.675792	94.647778
3641.604608	94.802505
3643.533424	94.855781
3645.462240	95.022695
3647.391056	95.475376
3649.319872	95.829605

3651.248688	95.742898
3653.177504	95.487108
3655.106320	95.644775
3657.035136	95.799615
3658.963952	95.704728
3660.892768	95.781159
3662.821584	95.902873
3664.750400	96.008883
3666.679216	96.141205
3668.608032	96.397259
3670.536848	96.612371
3672.465664	96.554060
3674.394480	96.883042
3676.323296	97.094864
3678.252112	96.909052
3680.180928	96.947260
3682.109744	96.949224
3684.038560	96.997427
3685.967376	97.146964
3687.896192	97.451822
3689.825008	97.533765
3691.753824	97.464293
3693.682640	97.463077
3695.611456	97.542656
3697.540272	97.577963
3699.469088	97.611507

3701.397904	97.759665
3703.326720	97.765270
3705.255536	97.763471
3707.184352	97.872044
3709.113168	98.026726
3711.041984	98.184635
3712.970800	98.173743
3714.899616	98.035757
3716.828432	98.032943
3718.757248	98.095393
3720.686064	98.117703
3722.614880	98.119831
3724.543696	98.119347
3726.472512	98.143264
3728.401328	98.023803
3730.330144	98.036075
3732.258960	98.184163
3734.187776	98.341367
3736.116592	98.416017
3738.045408	98.146756
3739.974224	98.097993
3741.903040	98.157092
3743.831856	98.522250
3745.760672	98.421628
3747.689488	98.153855
3749.618304	98.321967

3751.547120	98.389912
3753.475936	98.206631
3755.404752	98.040204
3757.333568	98.027837
3759.262384	98.041926
3761.191200	97.986634
3763.120016	97.891733
3765.048832	97.960555
3766.977648	97.883639
3768.906464	97.863389
3770.835280	97.840888
3772.764096	97.720495
3774.692912	97.712602
3776.621728	97.732513
3778.550544	97.829878
3780.479360	97.789608
3782.408176	97.691593
3784.336992	97.731361
3786.265808	97.683991
3788.194624	97.620107
3790.123440	97.613868
3792.052256	97.617847
3793.981072	97.589991
3795.909888	97.681205
3797.838704	97.715568
3799.767520	97.790129

3801.696336	97.966836
3803.625152	97.702266
3805.553968	97.734819
3807.482784	97.875460
3809.411600	97.617606
3811.340416	97.496151
3813.269232	97.489529
3815.198048	97.660305
3817.126864	97.678878
3819.055680	97.662016
3820.984496	97.949941
3822.913312	97.699442
3824.842128	97.538879
3826.770944	97.626895
3828.699760	97.520176
3830.628576	97.512988
3832.557392	97.584547
3834.486208	97.591741
3836.415024	97.653315
3838.343840	97.728867
3840.272656	97.697322
3842.201472	97.597377
3844.130288	97.542124
3846.059104	97.394018
3847.987920	97.333920
3849.916736	97.457168

3851.845552	97.737967
3853.774368	97.950369
3855.703184	97.626039
3857.632000	97.458232
3859.560816	97.462251
3861.489632	97.536716
3863.418448	97.594071
3865.347264	97.576077
3867.276080	97.423514
3869.204896	97.622649
3871.133712	97.625007
3873.062528	97.438777
3874.991344	97.422593
3876.920160	97.232914
3878.848976	97.316158
3880.777792	97.465372
3882.706608	97.312103
3884.635424	97.453829
3886.564240	97.501063
3888.493056	97.225775
3890.421872	97.339945
3892.350688	97.396946
3894.279504	97.260409
3896.208320	97.225822
3898.137136	97.379204
3900.065952	97.489444

3901.994768	97.472184
3903.923584	97.527752
3905.852400	97.413705
3907.781216	97.224358
3909.710032	97.147973
3911.638848	97.166723
3913.567664	97.115321
3915.496480	97.193469
3917.425296	97.312116
3919.354112	97.237978
3921.282928	97.179103
3923.211744	97.213981
3925.140560	97.235612
3927.069376	97.158820
3928.998192	97.142133
3930.927008	97.183341
3932.855824	97.244415
3934.784640	97.117917
3936.713456	97.041264
3938.642272	97.059894
3940.571088	97.054413
3942.499904	97.162160
3944.428720	97.107727
3946.357536	97.035399
3948.286352	97.092131
3950.215168	97.056776

3952.143984	96.998489
3954.072800	96.964756
3956.001616	96.942309
3957.930432	96.959603
3959.859248	96.995470
3961.788064	97.001233
3963.716880	96.964858
3965.645696	96.933003
3967.574512	96.913470
3969.503328	96.939553
3971.432144	96.980908
3973.360960	96.977292
3975.289776	96.978392
3977.218592	96.971606
3979.147408	96.904012
3981.076224	96.861970
3983.005040	96.902094
3984.933856	96.923177
3986.862672	96.916136
3988.791488	96.931622
3990.720304	96.928112
3992.649120	96.868644
3994.577936	96.828701
3996.506752	96.792456
3998.435568	96.824239
4000.364384	96.853309

جـامـعـة الـجـوف
وكالة الجامعة للدراسات العليا والبحث العلمي
المختبرات المركزية

SHIMADZU

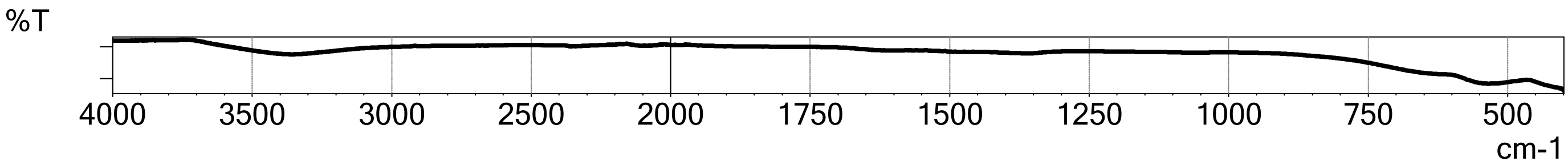

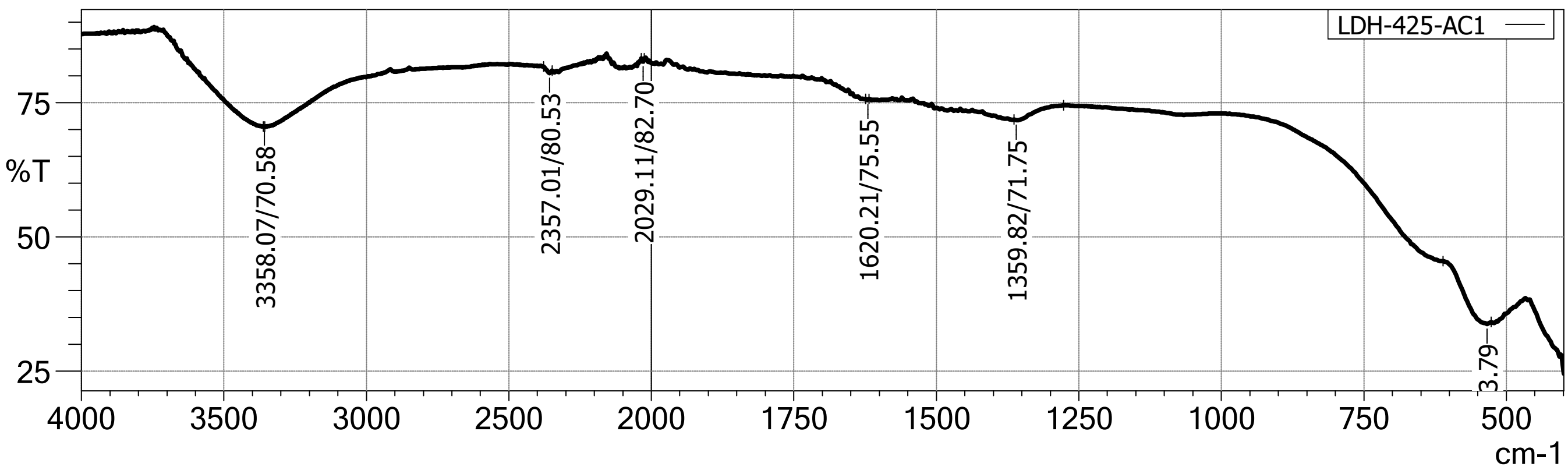

	Peak	Intensity	Corr. Intensity	Base (H)	Base (L)	Area	Corr. Area	Comment
1	534.28	33.79	1.41	611.43	526.57	5187.128	82.175	
2	1359.82	71.75	0.30	1363.67	1276.88	2311.698	-12.198	
3	1620.21	75.55	0.19	1624.06	1618.28	141.058	0.664	
4	2029.11	82.70	0.59	2034.90	2025.26	164.176	2.980	
5	2357.01	80.53	0.73	2378.23	2347.37	586.178	12.138	
6	3358.07	70.58	0.00	3361.93	3356.14	170.240	0.018	

##TITLE=No Description

##DATA TYPE=INFRARED SPECTRUM

##XUNITS=1/CM

##YUNITS=%T

399.264912	24.573653
401.193728	26.158464
403.122544	27.965086
405.051360	28.115965
406.980176	27.731758
408.908992	28.454157
410.837808	28.954071
412.766624	29.128938
414.695440	29.354059
416.624256	29.485421
418.553072	29.635668
420.481888	29.941928
422.410704	30.483435
424.339520	30.770943
426.268336	31.156871
428.197152	31.532997
430.125968	31.684057
432.054784	31.977294
433.983600	32.383789
435.912416	32.797174
437.841232	33.191721
439.770048	33.671238

441.698864	34.154768
443.627680	34.616872
445.556496	35.254485
447.485312	35.762231
449.414128	36.122047
451.342944	36.619253
453.271760	37.101117
455.200576	37.437325
457.129392	37.842829
459.058208	38.349348
460.987024	38.358298
462.915840	38.237161
464.844656	38.406021
466.773472	38.621211
468.702288	38.499755
470.631104	38.266414
472.559920	38.086529
474.488736	38.068559
476.417552	37.891389
478.346368	37.622949
480.275184	37.446126
482.204000	37.165342
484.132816	36.944738
486.061632	36.973559
487.990448	36.920498
489.919264	36.711836

491.848080	36.547525
493.776896	36.397800
495.705712	36.136482
497.634528	35.922688
499.563344	35.772106
501.492160	35.726191
503.420976	35.692891
505.349792	35.319118
507.278608	34.961304
509.207424	34.820369
511.136240	34.779739
513.065056	34.549637
514.993872	34.280747
516.922688	34.331408
518.851504	34.181694
520.780320	33.951547
522.709136	34.007036
524.637952	34.083306
526.566768	34.178738
528.495584	34.065935
530.424400	33.958108
532.353216	33.853420
534.282032	33.794765
536.210848	33.894630
538.139664	34.010952
540.068480	34.012996

541.997296	34.041642
543.926112	34.115087
545.854928	34.275595
547.783744	34.504220
549.712560	34.593931
551.641376	34.742651
553.570192	35.111247
555.499008	35.412955
557.427824	35.595440
559.356640	35.934677
561.285456	36.409911
563.214272	36.820323
565.143088	37.177160
567.071904	37.667375
569.000720	38.134277
570.929536	38.500102
572.858352	38.908610
574.787168	39.388583
576.715984	39.935443
578.644800	40.471132
580.573616	40.892842
582.502432	41.350706
584.431248	41.852233
586.360064	42.410002
588.288880	42.925137
590.217696	43.384559

592.146512	43.800980
594.075328	44.131038
596.004144	44.461017
597.932960	44.729720
599.861776	44.919178
601.790592	45.115581
603.719408	45.291555
605.648224	45.361065
607.577040	45.407176
609.505856	45.507120
611.434672	45.517772
613.363488	45.499151
615.292304	45.528264
617.221120	45.520566
619.149936	45.599656
621.078752	45.752334
623.007568	45.819820
624.936384	45.814118
626.865200	45.867682
628.794016	45.981589
630.722832	46.114355
632.651648	46.173898
634.580464	46.253921
636.509280	46.330316
638.438096	46.415813
640.366912	46.568705

642.295728	46.647485
644.224544	46.807874
646.153360	47.021858
648.082176	47.112093
650.010992	47.209381
651.939808	47.339022
653.868624	47.429063
655.797440	47.662579
657.726256	47.913735
659.655072	48.120132
661.583888	48.308918
663.512704	48.546027
665.441520	48.650079
667.370336	48.674709
669.299152	49.206744
671.227968	49.426808
673.156784	49.554055
675.085600	49.773782
677.014416	49.991998
678.943232	50.203062
680.872048	50.399993
682.800864	50.666728
684.729680	50.927589
686.658496	51.187727
688.587312	51.478606
690.516128	51.746585

692.444944	51.996947
694.373760	52.272287
696.302576	52.619272
698.231392	52.858521
700.160208	53.087471
702.089024	53.348731
704.017840	53.608401
705.946656	53.868435
707.875472	54.142350
709.804288	54.398879
711.733104	54.712948
713.661920	55.053395
715.590736	55.308350
717.519552	55.553488
719.448368	55.877241
721.377184	56.145104
723.306000	56.386095
725.234816	56.665531
727.163632	56.968638
729.092448	57.286948
731.021264	57.546884
732.950080	57.781796
734.878896	58.015862
736.807712	58.271311
738.736528	58.540594
740.665344	58.820923

742.594160	59.076929
744.522976	59.351028
746.451792	59.644554
748.380608	59.882203
750.309424	60.070010
752.238240	60.320590
754.167056	60.599172
756.095872	60.822598
758.024688	61.019111
759.953504	61.263015
761.882320	61.477030
763.811136	61.731843
765.739952	62.012761
767.668768	62.180483
769.597584	62.415813
771.526400	62.674736
773.455216	62.852178
775.384032	63.013062
777.312848	63.230790
779.241664	63.446423
781.170480	63.644961
783.099296	63.847866
785.028112	64.037690
786.956928	64.202094
788.885744	64.361175
790.814560	64.552075

792.743376	64.708009
794.672192	64.860036
796.601008	65.041618
798.529824	65.207681
800.458640	65.380162
802.387456	65.555650
804.316272	65.713848
806.245088	65.853099
808.173904	65.954733
810.102720	66.108475
812.031536	66.257726
813.960352	66.394091
815.889168	66.603873
817.817984	66.752207
819.746800	66.832153
821.675616	66.963172
823.604432	67.089817
825.533248	67.204707
827.462064	67.334388
829.390880	67.462489
831.319696	67.553911
833.248512	67.648481
835.177328	67.808927
837.106144	67.899027
839.034960	67.999170
840.963776	68.135466

842.892592	68.205481
844.821408	68.296512
846.750224	68.420089
848.679040	68.528298
850.607856	68.645449
852.536672	68.759532
854.465488	68.862676
856.394304	68.960951
858.323120	69.066441
860.251936	69.183336
862.180752	69.332747
864.109568	69.477427
866.038384	69.596045
867.967200	69.705374
869.896016	69.820784
871.824832	69.925850
873.753648	70.042377
875.682464	70.183587
877.611280	70.291637
879.540096	70.361842
881.468912	70.475077
883.397728	70.586491
885.326544	70.678692
887.255360	70.763797
889.184176	70.844921
891.112992	70.942529

893.041808	70.993958
894.970624	71.056107
896.899440	71.140018
898.828256	71.244311
900.757072	71.344171
902.685888	71.410757
904.614704	71.454062
906.543520	71.510597
908.472336	71.574374
910.401152	71.630577
912.329968	71.660763
914.258784	71.708106
916.187600	71.791250
918.116416	71.875837
920.045232	71.930629
921.974048	71.942809
923.902864	71.994965
925.831680	72.042940
927.760496	72.071144
929.689312	72.129469
931.618128	72.198348
933.546944	72.262367
935.475760	72.331341
937.404576	72.352366
939.333392	72.334648
941.262208	72.388175

943.191024	72.445755
945.119840	72.448027
947.048656	72.459301
948.977472	72.492753
950.906288	72.562706
952.835104	72.587452
954.763920	72.586747
956.692736	72.622803
958.621552	72.684296
960.550368	72.719497
962.479184	72.709134
964.408000	72.711054
966.336816	72.734845
968.265632	72.777214
970.194448	72.801845
972.123264	72.803648
974.052080	72.847777
975.980896	72.883322
977.909712	72.879040
979.838528	72.884037
981.767344	72.896858
983.696160	72.946343
985.624976	72.986311
987.553792	72.964240
989.482608	72.960922
991.411424	73.001646

993.340240	73.017923
995.269056	73.013630
997.197872	73.013769
999.126688	73.033372
1001.055504	73.032155
1002.984320	73.001631
1004.913136	73.005092
1006.841952	73.023181
1008.770768	73.009404
1010.699584	73.000976
1012.628400	73.017103
1014.557216	73.020565
1016.486032	72.999459
1018.414848	73.006439
1020.343664	73.031116
1022.272480	73.019411
1024.201296	72.975533
1026.130112	72.968090
1028.058928	72.980276
1029.987744	72.963184
1031.916560	72.947184
1033.845376	72.940889
1035.774192	72.896660
1037.703008	72.866654
1039.631824	72.883853
1041.560640	72.873488

1043.489456	72.846411
1045.418272	72.842124
1047.347088	72.852142
1049.275904	72.819509
1051.204720	72.795053
1053.133536	72.775962
1055.062352	72.784114
1056.991168	72.812467
1058.919984	72.802466
1060.848800	72.812239
1062.777616	72.798358
1064.706432	72.769008
1066.635248	72.760545
1068.564064	72.805037
1070.492880	72.834616
1072.421696	72.810021
1074.350512	72.821558
1076.279328	72.809048
1078.208144	72.782778
1080.136960	72.815017
1082.065776	72.857313
1083.994592	72.876830
1085.923408	72.923809
1087.852224	73.006650
1089.781040	73.042348
1091.709856	73.063094

1093.638672	73.092429
1095.567488	73.103110
1097.496304	73.154804
1099.425120	73.209616
1101.353936	73.249236
1103.282752	73.259836
1105.211568	73.247895
1107.140384	73.288964
1109.069200	73.335701
1110.998016	73.378646
1112.926832	73.378695
1114.855648	73.373665
1116.784464	73.411561
1118.713280	73.429101
1120.642096	73.453835
1122.570912	73.462365
1124.499728	73.502521
1126.428544	73.563625
1128.357360	73.554702
1130.286176	73.542107
1132.214992	73.550759
1134.143808	73.578578
1136.072624	73.623579
1138.001440	73.645744
1139.930256	73.643382
1141.859072	73.648344

1143.787888	73.651151
1145.716704	73.657438
1147.645520	73.679189
1149.574336	73.669820
1151.503152	73.676670
1153.431968	73.710204
1155.360784	73.769936
1157.289600	73.804453
1159.218416	73.745855
1161.147232	73.742348
1163.076048	73.783354
1165.004864	73.806074
1166.933680	73.824115
1168.862496	73.831829
1170.791312	73.860645
1172.720128	73.897610
1174.648944	73.922980
1176.577760	73.916342
1178.506576	73.918400
1180.435392	73.937674
1182.364208	73.933292
1184.293024	73.935485
1186.221840	73.958711
1188.150656	73.969757
1190.079472	73.973767
1192.008288	73.996006

1193.937104	74.060106
1195.865920	74.091674
1197.794736	74.100562
1199.723552	74.149474
1201.652368	74.164641
1203.581184	74.189732
1205.510000	74.200231
1207.438816	74.154314
1209.367632	74.155914
1211.296448	74.213320
1213.225264	74.234192
1215.154080	74.230567
1217.082896	74.232812
1219.011712	74.231404
1220.940528	74.249317
1222.869344	74.274516
1224.798160	74.298109
1226.726976	74.305303
1228.655792	74.300340
1230.584608	74.328735
1232.513424	74.349963
1234.442240	74.367200
1236.371056	74.384219
1238.299872	74.372101
1240.228688	74.366024
1242.157504	74.384417

1244.086320	74.411388
1246.015136	74.396846
1247.943952	74.389183
1249.872768	74.419538
1251.801584	74.431366
1253.730400	74.426144
1255.659216	74.436053
1257.588032	74.430544
1259.516848	74.447740
1261.445664	74.472430
1263.374480	74.478639
1265.303296	74.497094
1267.232112	74.515774
1269.160928	74.538275
1271.089744	74.549477
1273.018560	74.530543
1274.947376	74.523693
1276.876192	74.526603
1278.805008	74.520819
1280.733824	74.502136
1282.662640	74.481924
1284.591456	74.458987
1286.520272	74.438378
1288.449088	74.436892
1290.377904	74.407132
1292.306720	74.371056

1294.235536	74.363358
1296.164352	74.348911
1298.093168	74.303405
1300.021984	74.261816
1301.950800	74.207170
1303.879616	74.154266
1305.808432	74.117498
1307.737248	74.086662
1309.666064	74.027200
1311.594880	73.951619
1313.523696	73.899376
1315.452512	73.831199
1317.381328	73.782635
1319.310144	73.708983
1321.238960	73.571722
1323.167776	73.484186
1325.096592	73.404743
1327.025408	73.307447
1328.954224	73.195460
1330.883040	73.075803
1332.811856	72.959946
1334.740672	72.845592
1336.669488	72.773670
1338.598304	72.738005
1340.527120	72.619253
1342.455936	72.408623

1344.384752	72.258988
1346.313568	72.153208
1348.242384	72.036320
1350.171200	71.945482
1352.100016	71.871814
1354.028832	71.825078
1355.957648	71.784289
1357.886464	71.748833
1359.815280	71.745226
1361.744096	71.852651
1363.672912	71.925393
1365.601728	71.808650
1367.530544	71.805856
1369.459360	71.881659
1371.388176	71.900214
1373.316992	72.037885
1375.245808	72.120190
1377.174624	72.049142
1379.103440	72.051227
1381.032256	72.067462
1382.961072	72.103773
1384.889888	72.146259
1386.818704	72.263231
1388.747520	72.323034
1390.676336	72.280664
1392.605152	72.299134

1394.533968	72.471466
1396.462784	72.575385
1398.391600	72.605280
1400.320416	72.638225
1402.249232	72.557812
1404.178048	72.638132
1406.106864	72.733722
1408.035680	72.733984
1409.964496	72.808616
1411.893312	72.875389
1413.822128	72.925561
1415.750944	73.040235
1417.679760	73.202653
1419.608576	73.403942
1421.537392	73.298906
1423.466208	73.278300
1425.395024	73.327729
1427.323840	73.286416
1429.252656	73.377244
1431.181472	73.447315
1433.110288	73.434731
1435.039104	73.473990
1436.967920	73.684167
1438.896736	73.577852
1440.825552	73.434255
1442.754368	73.464516

1444.683184	73.458148
1446.612000	73.511159
1448.540816	73.568693
1450.469632	73.491996
1452.398448	73.487562
1454.327264	73.488993
1456.256080	73.697707
1458.184896	73.924727
1460.113712	73.683987
1462.042528	73.508568
1463.971344	73.566854
1465.900160	73.670745
1467.828976	73.592482
1469.757792	73.526625
1471.686608	73.693220
1473.615424	73.878032
1475.544240	73.790513
1477.473056	73.694809
1479.401872	73.574585
1481.330688	73.606341
1483.259504	73.625997
1485.188320	73.625223
1487.117136	73.755387
1489.045952	73.909886
1490.974768	74.003001
1492.903584	73.823430

1494.832400	73.779419
1496.761216	74.009275
1498.690032	74.063723
1500.618848	74.041167
1502.547664	74.013937
1504.476480	74.023392
1506.405296	74.528683
1508.334112	74.804903
1510.262928	74.529817
1512.191744	74.484247
1514.120560	74.466442
1516.049376	74.632384
1517.978192	74.772928
1519.907008	74.714453
1521.835824	74.979908
1523.764640	74.933678
1525.693456	74.904825
1527.622272	74.985757
1529.551088	74.961053
1531.479904	74.975375
1533.408720	75.203464
1535.337536	75.275896
1537.266352	75.180653
1539.195168	75.512050
1541.123984	75.775988
1543.052800	75.682012

1544.981616	75.675950
1546.910432	75.454923
1548.839248	75.416852
1550.768064	75.516044
1552.696880	75.416603
1554.625696	75.532427
1556.554512	75.496927
1558.483328	75.765721
1560.412144	76.027650
1562.340960	75.592141
1564.269776	75.522924
1566.198592	75.547858
1568.127408	75.542543
1570.056224	75.742604
1571.985040	75.693274
1573.913856	75.571992
1575.842672	75.784307
1577.771488	75.862892
1579.700304	75.678154
1581.629120	75.644094
1583.557936	75.613805
1585.486752	75.581371
1587.415568	75.601033
1589.344384	75.613005
1591.273200	75.605978
1593.202016	75.594936

1595.130832	75.591889
1597.059648	75.575865
1598.988464	75.547964
1600.917280	75.541063
1602.846096	75.578189
1604.774912	75.567416
1606.703728	75.560693
1608.632544	75.580057
1610.561360	75.570232
1612.490176	75.556218
1614.418992	75.500157
1616.347808	75.634169
1618.276624	75.743757
1620.205440	75.554392
1622.134256	75.576360
1624.063072	75.731158
1625.991888	75.678641
1627.920704	75.722587
1629.849520	75.777434
1631.778336	75.748026
1633.707152	75.776006
1635.635968	76.102247
1637.564784	76.244338
1639.493600	76.108459
1641.422416	76.166413
1643.351232	76.232115

1645.280048	76.404867
1647.208864	76.798429
1649.137680	76.801583
1651.066496	76.650774
1652.995312	77.223843
1654.924128	77.453781
1656.852944	77.156019
1658.781760	77.200784
1660.710576	77.297848
1662.639392	77.495763
1664.568208	77.662968
1666.497024	77.669703
1668.425840	77.815448
1670.354656	78.095582
1672.283472	78.090449
1674.212288	78.138957
1676.141104	78.402328
1678.069920	78.356525
1679.998736	78.464110
1681.927552	78.548645
1683.856368	78.840066
1685.785184	79.033132
1687.714000	78.848660
1689.642816	78.888239
1691.571632	78.897922
1693.500448	78.865380

1695.429264	79.168604
1697.358080	79.286382
1699.286896	79.242904
1701.215712	79.612926
1703.144528	79.361913
1705.073344	79.352735
1707.002160	79.410077
1708.930976	79.340321
1710.859792	79.372927
1712.788608	79.416252
1714.717424	79.548669
1716.646240	79.646385
1718.575056	79.770145
1720.503872	79.607231
1722.432688	79.536276
1724.361504	79.603433
1726.290320	79.548408
1728.219136	79.607426
1730.147952	79.742384
1732.076768	79.755486
1734.005584	80.052822
1735.934400	80.012330
1737.863216	79.750889
1739.792032	79.830031
1741.720848	79.845981
1743.649664	79.866224

1745.578480	79.859377
1747.507296	79.895399
1749.436112	79.966150
1751.364928	80.040249
1753.293744	79.918073
1755.222560	79.804221
1757.151376	79.938227
1759.080192	79.897493
1761.009008	79.891349
1762.937824	79.966664
1764.866640	79.859345
1766.795456	79.918804
1768.724272	80.035047
1770.653088	79.990363
1772.581904	80.083876
1774.510720	80.038260
1776.439536	79.927399
1778.368352	79.882953
1780.297168	79.946929
1782.225984	79.976045
1784.154800	79.970547
1786.083616	79.993902
1788.012432	79.940529
1789.941248	79.999594
1791.870064	80.158690
1793.798880	80.128727

1795.727696	80.030467
1797.656512	79.999242
1799.585328	80.048482
1801.514144	80.150451
1803.442960	80.084922
1805.371776	80.007796
1807.300592	80.048211
1809.229408	80.122244
1811.158224	80.172487
1813.087040	80.146112
1815.015856	80.116039
1816.944672	80.150227
1818.873488	80.123559
1820.802304	80.142875
1822.731120	80.148493
1824.659936	80.250462
1826.588752	80.286692
1828.517568	80.258149
1830.446384	80.366157
1832.375200	80.254385
1834.304016	80.220706
1836.232832	80.306381
1838.161648	80.326928
1840.090464	80.309331
1842.019280	80.287505
1843.948096	80.466915

1845.876912	80.499622
1847.805728	80.374501
1849.734544	80.406522
1851.663360	80.400448
1853.592176	80.421286
1855.520992	80.429083
1857.449808	80.426141
1859.378624	80.455993
1861.307440	80.423631
1863.236256	80.456026
1865.165072	80.509912
1867.093888	80.512900
1869.022704	80.661838
1870.951520	80.681656
1872.880336	80.564627
1874.809152	80.556408
1876.737968	80.598405
1878.666784	80.635285
1880.595600	80.604876
1882.524416	80.583844
1884.453232	80.574047
1886.382048	80.600998
1888.310864	80.660991
1890.239680	80.748578
1892.168496	80.783262
1894.097312	80.816126

1896.026128	80.857600
1897.954944	80.867687
1899.883760	80.802101
1901.812576	80.676568
1903.741392	80.697591
1905.670208	80.740907
1907.599024	80.824789
1909.527840	80.884560
1911.456656	80.864775
1913.385472	80.897973
1915.314288	80.895945
1917.243104	81.058437
1919.171920	81.161069
1921.100736	81.072946
1923.029552	81.235631
1924.958368	81.336931
1926.887184	81.231208
1928.816000	81.187517
1930.744816	81.368899
1932.673632	81.401163
1934.602448	81.231182
1936.531264	81.238549
1938.460080	81.299342
1940.388896	81.333147
1942.317712	81.615169
1944.246528	81.762372

1946.175344	81.700511
1948.104160	81.694051
1950.032976	81.561377
1951.961792	81.687962
1953.890608	82.003817
1955.819424	82.179508
1957.748240	82.185089
1959.677056	82.088004
1961.605872	82.080383
1963.534688	82.357511
1965.463504	82.619838
1967.392320	82.811116
1969.321136	82.920318
1971.249952	82.947927
1973.178768	83.034269
1975.107584	82.857694
1977.036400	82.396994
1978.965216	82.146307
1980.894032	82.240969
1982.822848	82.288399
1984.751664	82.223554
1986.680480	82.213754
1988.609296	82.254637
1990.538112	82.508144
1992.466928	82.538982
1994.395744	82.284102

1996.324560	82.345728
1998.253376	82.305280
2000.182192	82.360393
2002.111008	82.498570
2004.039824	82.643753
2005.968640	82.880642
2007.897456	82.650026
2009.826272	82.635029
2011.755088	82.839674
2013.683904	82.769506
2015.612720	82.874096
2017.541536	83.241184
2019.470352	83.424002
2021.399168	83.356808
2023.327984	83.255673
2025.256800	83.295819
2027.185616	83.040312
2029.114432	82.695865
2031.043248	82.741896
2032.972064	83.118821
2034.900880	83.275232
2036.829696	82.915514
2038.758512	82.773252
2040.687328	82.806607
2042.616144	82.567816
2044.544960	82.403234

2046.473776	82.520465
2048.402592	82.214828
2050.331408	81.866455
2052.260224	81.961540
2054.189040	82.031400
2056.117856	82.103602
2058.046672	81.973588
2059.975488	81.868518
2061.904304	81.835678
2063.833120	81.769817
2065.761936	81.726016
2067.690752	81.570864
2069.619568	81.574463
2071.548384	81.604361
2073.477200	81.730763
2075.406016	81.731461
2077.334832	81.582156
2079.263648	81.558824
2081.192464	81.550392
2083.121280	81.541641
2085.050096	81.520362
2086.978912	81.469682
2088.907728	81.439930
2090.836544	81.566458
2092.765360	81.726905
2094.694176	81.735883

2096.622992	81.559371
2098.551808	81.497587
2100.480624	81.531043
2102.409440	81.458135
2104.338256	81.480033
2106.267072	81.564094
2108.195888	81.554672
2110.124704	81.476551
2112.053520	81.444063
2113.982336	81.585440
2115.911152	81.681810
2117.839968	81.640465
2119.768784	81.652154
2121.697600	81.691042
2123.626416	81.733251
2125.555232	81.755402
2127.484048	81.841302
2129.412864	82.104953
2131.341680	82.424091
2133.270496	82.551233
2135.199312	82.544145
2137.128128	82.363741
2139.056944	82.272539
2140.985760	82.581575
2142.914576	82.866940
2144.843392	83.149489

2146.772208	83.284833
2148.701024	83.217282
2150.629840	83.289901
2152.558656	83.536388
2154.487472	83.806606
2156.416288	84.213712
2158.345104	84.100432
2160.273920	83.649826
2162.202736	83.792033
2164.131552	83.778579
2166.060368	83.653999
2167.989184	83.319694
2169.918000	83.124238
2171.846816	83.293104
2173.775632	83.438671
2175.704448	83.306393
2177.633264	83.220863
2179.562080	83.497493
2181.490896	83.304320
2183.419712	83.250087
2185.348528	83.532140
2187.277344	83.386857
2189.206160	83.141922
2191.134976	83.114561
2193.063792	83.165121
2194.992608	82.940863

2196.921424	82.723105
2198.850240	82.721548
2200.779056	82.857104
2202.707872	82.914971
2204.636688	82.737444
2206.565504	82.668072
2208.494320	82.646100
2210.423136	82.533189
2212.351952	82.616782
2214.280768	82.800913
2216.209584	82.742384
2218.138400	82.604704
2220.067216	82.535622
2221.996032	82.638343
2223.924848	82.740754
2225.853664	82.618155
2227.782480	82.608358
2229.711296	82.537087
2231.640112	82.361523
2233.568928	82.295922
2235.497744	82.385527
2237.426560	82.512975
2239.355376	82.374769
2241.284192	82.245654
2243.213008	82.296124
2245.141824	82.256636

2247.070640	82.217711
2248.999456	82.306839
2250.928272	82.217168
2252.857088	82.169747
2254.785904	82.196917
2256.714720	82.102733
2258.643536	82.039230
2260.572352	81.965011
2262.501168	82.052516
2264.429984	82.070470
2266.358800	81.965694
2268.287616	81.961009
2270.216432	81.955941
2272.145248	81.955200
2274.074064	81.923293
2276.002880	81.889778
2277.931696	81.849953
2279.860512	81.777429
2281.789328	81.748700
2283.718144	81.723420
2285.646960	81.664335
2287.575776	81.639342
2289.504592	81.621741
2291.433408	81.553946
2293.362224	81.526154
2295.291040	81.566744

2297.219856	81.563218
2299.148672	81.520495
2301.077488	81.477269
2303.006304	81.463151
2304.935120	81.448523
2306.863936	81.344617
2308.792752	81.333337
2310.721568	81.326487
2312.650384	81.259177
2314.579200	81.249460
2316.508016	81.197565
2318.436832	81.065501
2320.365648	80.910858
2322.294464	80.829876
2324.223280	80.737093
2326.152096	80.749675
2328.080912	80.848874
2330.009728	80.901047
2331.938544	80.919827
2333.867360	80.915054
2335.796176	80.864049
2337.724992	80.739956
2339.653808	80.669381
2341.582624	80.695967
2343.511440	80.755449
2345.440256	80.869509

2347.369072	81.033439
2349.297888	80.979178
2351.226704	80.777386
2353.155520	80.719210
2355.084336	80.655019
2357.013152	80.533410
2358.941968	80.554957
2360.870784	80.637454
2362.799600	80.761960
2364.728416	80.912155
2366.657232	81.079636
2368.586048	81.219713
2370.514864	81.225125
2372.443680	81.344244
2374.372496	81.585515
2376.301312	81.710038
2378.230128	81.764977
2380.158944	81.751911
2382.087760	81.739580
2384.016576	81.803968
2385.945392	81.867774
2387.874208	81.874708
2389.803024	81.881798
2391.731840	81.858904
2393.660656	81.859750
2395.589472	81.856031

2397.518288	81.846455
2399.447104	81.873008
2401.375920	81.862622
2403.304736	81.893358
2405.233552	81.916534
2407.162368	81.915651
2409.091184	81.882471
2411.020000	81.871142
2412.948816	81.901983
2414.877632	81.937369
2416.806448	81.968866
2418.735264	81.978316
2420.664080	82.003341
2422.592896	82.005763
2424.521712	82.008091
2426.450528	81.992645
2428.379344	81.977911
2430.308160	81.984555
2432.236976	82.003954
2434.165792	82.060783
2436.094608	82.051269
2438.023424	82.038796
2439.952240	82.022655
2441.881056	81.993209
2443.809872	81.995222
2445.738688	81.995111

2447.667504	82.018713
2449.596320	82.052519
2451.525136	82.078092
2453.453952	82.071402
2455.382768	82.036581
2457.311584	82.078895
2459.240400	82.138557
2461.169216	82.115001
2463.098032	82.074745
2465.026848	82.093514
2466.955664	82.118600
2468.884480	82.118504
2470.813296	82.170338
2472.742112	82.167775
2474.670928	82.136802
2476.599744	82.129903
2478.528560	82.136556
2480.457376	82.127835
2482.386192	82.085592
2484.315008	82.140750
2486.243824	82.235346
2488.172640	82.188298
2490.101456	82.154219
2492.030272	82.213368
2493.959088	82.222512
2495.887904	82.220467

2497.816720	82.198745
2499.745536	82.164450
2501.674352	82.122790
2503.603168	82.129931
2505.531984	82.154262
2507.460800	82.146518
2509.389616	82.133655
2511.318432	82.148605
2513.247248	82.166092
2515.176064	82.196943
2517.104880	82.215475
2519.033696	82.177344
2520.962512	82.174196
2522.891328	82.181092
2524.820144	82.184240
2526.748960	82.191424
2528.677776	82.190166
2530.606592	82.213896
2532.535408	82.193722
2534.464224	82.154609
2536.393040	82.159022
2538.321856	82.198902
2540.250672	82.233129
2542.179488	82.225839
2544.108304	82.215322
2546.037120	82.164969

2547.965936	82.166152
2549.894752	82.176470
2551.823568	82.156895
2553.752384	82.182640
2555.681200	82.176102
2557.610016	82.159981
2559.538832	82.215457
2561.467648	82.203134
2563.396464	82.166658
2565.325280	82.241178
2567.254096	82.244599
2569.182912	82.177062
2571.111728	82.156812
2573.040544	82.112085
2574.969360	82.120488
2576.898176	82.147615
2578.826992	82.134573
2580.755808	82.142274
2582.684624	82.108125
2584.613440	82.060674
2586.542256	82.097616
2588.471072	82.115485
2590.399888	82.071118
2592.328704	82.078855
2594.257520	82.082681
2596.186336	82.060848

2598.115152	82.020541
2600.043968	81.989648
2601.972784	82.001976
2603.901600	82.024835
2605.830416	82.004831
2607.759232	81.970781
2609.688048	81.952827
2611.616864	81.939281
2613.545680	81.938445
2615.474496	81.912837
2617.403312	81.882024
2619.332128	81.889652
2621.260944	81.883490
2623.189760	81.844515
2625.118576	81.809549
2627.047392	81.807808
2628.976208	81.822855
2630.905024	81.821941
2632.833840	81.801624
2634.762656	81.741646
2636.691472	81.726901
2638.620288	81.731929
2640.549104	81.687776
2642.477920	81.687981
2644.406736	81.692495
2646.335552	81.689068

2648.264368	81.700092
2650.193184	81.675379
2652.122000	81.650141
2654.050816	81.636441
2655.979632	81.609732
2657.908448	81.598822
2659.837264	81.631767
2661.766080	81.612144
2663.694896	81.580105
2665.623712	81.605294
2667.552528	81.615161
2669.481344	81.616848
2671.410160	81.636060
2673.338976	81.632440
2675.267792	81.636919
2677.196608	81.638079
2679.125424	81.621991
2681.054240	81.606263
2682.983056	81.598264
2684.911872	81.608384
2686.840688	81.609232
2688.769504	81.627459
2690.698320	81.642711
2692.627136	81.633942
2694.555952	81.647775
2696.484768	81.638640

2698.413584	81.605709
2700.342400	81.618494
2702.271216	81.615563
2704.200032	81.598466
2706.128848	81.589192
2708.057664	81.574842
2709.986480	81.568444
2711.915296	81.597538
2713.844112	81.610078
2715.772928	81.590889
2717.701744	81.584412
2719.630560	81.554976
2721.559376	81.543115
2723.488192	81.560651
2725.417008	81.569382
2727.345824	81.566922
2729.274640	81.535170
2731.203456	81.521193
2733.132272	81.544970
2735.061088	81.548958
2736.989904	81.545750
2738.918720	81.553999
2740.847536	81.548768
2742.776352	81.535825
2744.705168	81.529314
2746.633984	81.530714

2748.562800	81.529426
2750.491616	81.510672
2752.420432	81.502436
2754.349248	81.481562
2756.278064	81.463806
2758.206880	81.469142
2760.135696	81.487712
2762.064512	81.477754
2763.993328	81.463604
2765.922144	81.474418
2767.850960	81.462125
2769.779776	81.451012
2771.708592	81.436377
2773.637408	81.447731
2775.566224	81.431949
2777.495040	81.413604
2779.423856	81.423660
2781.352672	81.419978
2783.281488	81.421466
2785.210304	81.406128
2787.139120	81.400707
2789.067936	81.389170
2790.996752	81.369559
2792.925568	81.375283
2794.854384	81.370175
2796.783200	81.347386

2798.712016	81.342188
2800.640832	81.358005
2802.569648	81.340329
2804.498464	81.328886
2806.427280	81.349677
2808.356096	81.351322
2810.284912	81.348303
2812.213728	81.341271
2814.142544	81.309245
2816.071360	81.288641
2818.000176	81.298412
2819.928992	81.277115
2821.857808	81.255588
2823.786624	81.265771
2825.715440	81.276984
2827.644256	81.274867
2829.573072	81.242597
2831.501888	81.195357
2833.430704	81.193820
2835.359520	81.214901
2837.288336	81.210088
2839.217152	81.223750
2841.145968	81.264456
2843.074784	81.304927
2845.003600	81.341275
2846.932416	81.441179

2848.861232	81.542872
2850.790048	81.535967
2852.718864	81.430497
2854.647680	81.300468
2856.576496	81.252741
2858.505312	81.212852
2860.434128	81.147180
2862.362944	81.114869
2864.291760	81.093892
2866.220576	81.059938
2868.149392	81.030016
2870.078208	81.004907
2872.007024	80.981085
2873.935840	80.971561
2875.864656	80.969647
2877.793472	80.957982
2879.722288	80.932925
2881.651104	80.919581
2883.579920	80.905950
2885.508736	80.886585
2887.437552	80.886487
2889.366368	80.897676
2891.295184	80.882435
2893.224000	80.839915
2895.152816	80.805704
2897.081632	80.814929

2899.010448	80.819209
2900.939264	80.801335
2902.868080	80.831798
2904.796896	80.887199
2906.725712	80.892220
2908.654528	80.908941
2910.583344	80.972774
2912.512160	81.091037
2914.440976	81.222065
2916.369792	81.265141
2918.298608	81.235340
2920.227424	81.173291
2922.156240	81.074580
2924.085056	80.973408
2926.013872	80.901000
2927.942688	80.855574
2929.871504	80.778705
2931.800320	80.701934
2933.729136	80.660756
2935.657952	80.628083
2937.586768	80.626524
2939.515584	80.602234
2941.444400	80.550968
2943.373216	80.507749
2945.302032	80.488799
2947.230848	80.474978

2949.159664	80.435394
2951.088480	80.402606
2953.017296	80.372295
2954.946112	80.346223
2956.874928	80.347578
2958.803744	80.363190
2960.732560	80.347273
2962.661376	80.313431
2964.590192	80.260341
2966.519008	80.182665
2968.447824	80.143641
2970.376640	80.125774
2972.305456	80.104703
2974.234272	80.094793
2976.163088	80.094018
2978.091904	80.070429
2980.020720	80.056277
2981.949536	80.014622
2983.878352	79.962210
2985.807168	79.971705
2987.735984	79.976581
2989.664800	79.949887
2991.593616	79.919096
2993.522432	79.931933
2995.451248	79.930085
2997.380064	79.867580

2999.308880	79.799463
3001.237696	79.764314
3003.166512	79.802376
3005.095328	79.844061
3007.024144	79.827407
3008.952960	79.792593
3010.881776	79.768922
3012.810592	79.744371
3014.739408	79.747536
3016.668224	79.732539
3018.597040	79.702668
3020.525856	79.705577
3022.454672	79.709695
3024.383488	79.667823
3026.312304	79.650817
3028.241120	79.605154
3030.169936	79.520237
3032.098752	79.513523
3034.027568	79.515931
3035.956384	79.507425
3037.885200	79.460599
3039.814016	79.418906
3041.742832	79.410349
3043.671648	79.375638
3045.600464	79.337978
3047.529280	79.321057

3049.458096	79.304187
3051.386912	79.249742
3053.315728	79.233161
3055.244544	79.246509
3057.173360	79.203777
3059.102176	79.164907
3061.030992	79.123679
3062.959808	79.088838
3064.888624	79.082936
3066.817440	79.052019
3068.746256	78.985510
3070.675072	78.920643
3072.603888	78.897381
3074.532704	78.876320
3076.461520	78.816257
3078.390336	78.759581
3080.319152	78.731680
3082.247968	78.677162
3084.176784	78.627836
3086.105600	78.630703
3088.034416	78.575013
3089.963232	78.535934
3091.892048	78.529166
3093.820864	78.492586
3095.749680	78.459201
3097.678496	78.396291

3099.607312	78.301739
3101.536128	78.265997
3103.464944	78.256733
3105.393760	78.169799
3107.322576	78.066450
3109.251392	78.017075
3111.180208	77.997315
3113.109024	78.001305
3115.037840	77.978230
3116.966656	77.913436
3118.895472	77.860812
3120.824288	77.786788
3122.753104	77.727270
3124.681920	77.688195
3126.610736	77.605770
3128.539552	77.550053
3130.468368	77.496891
3132.397184	77.427643
3134.326000	77.378798
3136.254816	77.306591
3138.183632	77.251359
3140.112448	77.215528
3142.041264	77.154369
3143.970080	77.077656
3145.898896	77.037137
3147.827712	76.976166

3149.756528	76.886787
3151.685344	76.839423
3153.614160	76.759654
3155.542976	76.686393
3157.471792	76.644972
3159.400608	76.583772
3161.329424	76.515473
3163.258240	76.425264
3165.187056	76.359708
3167.115872	76.302306
3169.044688	76.218486
3170.973504	76.126589
3172.902320	76.078900
3174.831136	76.023008
3176.759952	75.957283
3178.688768	75.880149
3180.617584	75.794507
3182.546400	75.751907
3184.475216	75.671972
3186.404032	75.604538
3188.332848	75.543672
3190.261664	75.484011
3192.190480	75.425418
3194.119296	75.322101
3196.048112	75.226869
3197.976928	75.153465

3199.905744	75.061801
3201.834560	75.019658
3203.763376	75.003844
3205.692192	74.919913
3207.621008	74.810234
3209.549824	74.700983
3211.478640	74.644029
3213.407456	74.625430
3215.336272	74.568111
3217.265088	74.490905
3219.193904	74.438691
3221.122720	74.361255
3223.051536	74.276776
3224.980352	74.199503
3226.909168	74.106407
3228.837984	74.086433
3230.766800	74.044200
3232.695616	73.941020
3234.624432	73.881983
3236.553248	73.820832
3238.482064	73.753006
3240.410880	73.708888
3242.339696	73.629067
3244.268512	73.530507
3246.197328	73.459620
3248.126144	73.377593

3250.054960	73.339558
3251.983776	73.301073
3253.912592	73.217596
3255.841408	73.145953
3257.770224	73.068642
3259.699040	73.013445
3261.627856	72.979407
3263.556672	72.918864
3265.485488	72.840524
3267.414304	72.764519
3269.343120	72.700005
3271.271936	72.619083
3273.200752	72.547178
3275.129568	72.481239
3277.058384	72.399342
3278.987200	72.337835
3280.916016	72.282602
3282.844832	72.232987
3284.773648	72.171434
3286.702464	72.089184
3288.631280	72.032433
3290.560096	71.943306
3292.488912	71.867092
3294.417728	71.831670
3296.346544	71.781539
3298.275360	71.704273

3300.204176	71.600507
3302.132992	71.546439
3304.061808	71.516073
3305.990624	71.478622
3307.919440	71.417705
3309.848256	71.330737
3311.777072	71.293170
3313.705888	71.271922
3315.634704	71.192248
3317.563520	71.124914
3319.492336	71.099084
3321.421152	71.020217
3323.349968	70.958131
3325.278784	70.949541
3327.207600	70.897546
3329.136416	70.851633
3331.065232	70.801458
3332.994048	70.778187
3334.922864	70.784058
3336.851680	70.745393
3338.780496	70.709015
3340.709312	70.664577
3342.638128	70.656852
3344.566944	70.664207
3346.495760	70.647510
3348.424576	70.643399

3350.353392	70.614769
3352.282208	70.561543
3354.211024	70.550237
3356.139840	70.581551
3358.068656	70.577705
3359.997472	70.578381
3361.926288	70.583886
3363.855104	70.568498
3365.783920	70.580929
3367.712736	70.631789
3369.641552	70.670145
3371.570368	70.665594
3373.499184	70.688996
3375.428000	70.734713
3377.356816	70.718998
3379.285632	70.726772
3381.214448	70.800588
3383.143264	70.818384
3385.072080	70.808353
3387.000896	70.854834
3388.929712	70.923677
3390.858528	71.000046
3392.787344	71.061316
3394.716160	71.090649
3396.644976	71.129996
3398.573792	71.182490

3400.502608	71.210634
3402.431424	71.289804
3404.360240	71.330013
3406.289056	71.343484
3408.217872	71.447616
3410.146688	71.513455
3412.075504	71.553484
3414.004320	71.603859
3415.933136	71.668384
3417.861952	71.735733
3419.790768	71.804121
3421.719584	71.904146
3423.648400	71.978210
3425.577216	72.010694
3427.506032	72.051417
3429.434848	72.147419
3431.363664	72.256337
3433.292480	72.337494
3435.221296	72.410226
3437.150112	72.468664
3439.078928	72.505091
3441.007744	72.595699
3442.936560	72.707498
3444.865376	72.777347
3446.794192	72.937522
3448.723008	73.029223

3450.651824	73.041387
3452.580640	73.133723
3454.509456	73.230180
3456.438272	73.346362
3458.367088	73.469042
3460.295904	73.508738
3462.224720	73.536996
3464.153536	73.634482
3466.082352	73.755533
3468.011168	73.842860
3469.939984	73.893267
3471.868800	73.996385
3473.797616	74.132144
3475.726432	74.233320
3477.655248	74.315443
3479.584064	74.430503
3481.512880	74.532989
3483.441696	74.637332
3485.370512	74.713392
3487.299328	74.822752
3489.228144	74.943582
3491.156960	75.007770
3493.085776	75.090860
3495.014592	75.197122
3496.943408	75.319513
3498.872224	75.454755

3500.801040	75.583924
3502.729856	75.691887
3504.658672	75.808389
3506.587488	75.833899
3508.516304	75.892721
3510.445120	76.057429
3512.373936	76.160713
3514.302752	76.261303
3516.231568	76.364688
3518.160384	76.432878
3520.089200	76.530140
3522.018016	76.706312
3523.946832	76.862786
3525.875648	76.959191
3527.804464	77.095922
3529.733280	77.204330
3531.662096	77.269948
3533.590912	77.326223
3535.519728	77.426982
3537.448544	77.533470
3539.377360	77.648270
3541.306176	77.785199
3543.234992	77.858585
3545.163808	77.996479
3547.092624	78.182514
3549.021440	78.239857

3550.950256	78.322826
3552.879072	78.496182
3554.807888	78.576054
3556.736704	78.665668
3558.665520	78.785940
3560.594336	78.872652
3562.523152	79.000001
3564.451968	79.101423
3566.380784	79.357110
3568.309600	79.616718
3570.238416	79.509009
3572.167232	79.475485
3574.096048	79.577441
3576.024864	79.677294
3577.953680	79.805163
3579.882496	79.938791
3581.811312	80.031129
3583.740128	80.122886
3585.668944	80.280506
3587.597760	80.633436
3589.526576	80.766799
3591.455392	80.621823
3593.384208	80.744273
3595.313024	80.945159
3597.241840	81.015420
3599.170656	81.063020

3601.099472	81.202075
3603.028288	81.343996
3604.957104	81.393825
3606.885920	81.504098
3608.814736	81.775125
3610.743552	81.862438
3612.672368	81.951479
3614.601184	82.108598
3616.530000	82.112454
3618.458816	82.320875
3620.387632	82.540659
3622.316448	82.412139
3624.245264	82.426610
3626.174080	82.566314
3628.102896	82.850717
3630.031712	83.288209
3631.960528	83.103674
3633.889344	83.117175
3635.818160	83.198617
3637.746976	83.232750
3639.675792	83.363205
3641.604608	83.545951
3643.533424	83.669678
3645.462240	83.775651
3647.391056	84.195936
3649.319872	84.574906

3651.248688	84.769289
3653.177504	84.563790
3655.106320	84.513325
3657.035136	84.802702
3658.963952	84.810917
3660.892768	84.838604
3662.821584	84.993224
3664.750400	85.130147
3666.679216	85.287378
3668.608032	85.523964
3670.536848	85.944510
3672.465664	85.946997
3674.394480	86.017928
3676.323296	86.529931
3678.252112	86.469578
3680.180928	86.476963
3682.109744	86.546070
3684.038560	86.615663
3685.967376	86.705874
3687.896192	86.974385
3689.825008	87.288478
3691.753824	87.275374
3693.682640	87.272059
3695.611456	87.365496
3697.540272	87.505262
3699.469088	87.556582

3701.397904	87.770012
3703.326720	87.971281
3705.255536	88.015125
3707.184352	88.097109
3709.113168	88.251091
3711.041984	88.452000
3712.970800	88.623184
3714.899616	88.528425
3716.828432	88.400390
3718.757248	88.502375
3720.686064	88.547463
3722.614880	88.542663
3724.543696	88.547759
3726.472512	88.617922
3728.401328	88.594521
3730.330144	88.498263
3732.258960	88.678225
3734.187776	88.837186
3736.116592	88.968647
3738.045408	88.771412
3739.974224	88.684224
3741.903040	88.656160
3743.831856	88.929215
3745.760672	89.164047
3747.689488	88.723618
3749.618304	88.765999

3751.547120	88.962354
3753.475936	88.846235
3755.404752	88.638966
3757.333568	88.551171
3759.262384	88.612076
3761.191200	88.626062
3763.120016	88.458147
3765.048832	88.494959
3766.977648	88.505510
3768.906464	88.401140
3770.835280	88.459322
3772.764096	88.374261
3774.692912	88.316781
3776.621728	88.294911
3778.550544	88.357301
3780.479360	88.435622
3782.408176	88.330715
3784.336992	88.335722
3786.265808	88.333390
3788.194624	88.270329
3790.123440	88.220658
3792.052256	88.180837
3793.981072	88.135146
3795.909888	88.224762
3797.838704	88.337868
3799.767520	88.254840

3801.696336	88.476897
3803.625152	88.424940
3805.553968	88.272838
3807.482784	88.473687
3809.411600	88.305261
3811.340416	88.143711
3813.269232	88.088281
3815.198048	88.168776
3817.126864	88.385116
3819.055680	88.247332
3820.984496	88.447735
3822.913312	88.440623
3824.842128	88.136324
3826.770944	88.180803
3828.699760	88.140575
3830.628576	88.135813
3832.557392	88.281726
3834.486208	88.260171
3836.415024	88.236667
3838.343840	88.364024
3840.272656	88.390287
3842.201472	88.232591
3844.130288	88.171660
3846.059104	88.076796
3847.987920	88.029418
3849.916736	88.107562

3851.845552	88.184294
3853.774368	88.540209
3855.703184	88.509710
3857.632000	88.246751
3859.560816	88.127960
3861.489632	88.183209
3863.418448	88.230647
3865.347264	88.300145
3867.276080	88.147777
3869.204896	88.217134
3871.133712	88.395264
3873.062528	88.128373
3874.991344	88.161612
3876.920160	88.037187
3878.848976	87.964763
3880.777792	88.231685
3882.706608	88.128806
3884.635424	88.094419
3886.564240	88.287301
3888.493056	88.015687
3890.421872	87.973101
3892.350688	88.145711
3894.279504	88.026717
3896.208320	87.967554
3898.137136	88.077452
3900.065952	88.204635

3901.994768	88.212486
3903.923584	88.282254
3905.852400	88.216651
3907.781216	87.991003
3909.710032	87.890329
3911.638848	87.913955
3913.567664	87.888865
3915.496480	87.949132
3917.425296	88.088735
3919.354112	88.069036
3921.282928	88.031859
3923.211744	88.016743
3925.140560	88.031119
3927.069376	88.017758
3928.998192	87.983643
3930.927008	87.988318
3932.855824	88.059251
3934.784640	87.983320
3936.713456	87.902921
3938.642272	87.919491
3940.571088	87.885781
3942.499904	87.973554
3944.428720	87.985090
3946.357536	87.893675
3948.286352	87.932106
3950.215168	87.956615

3952.143984	87.905589
3954.072800	87.880435
3956.001616	87.895636
3957.930432	87.913276
3959.859248	87.910324
3961.788064	87.904772
3963.716880	87.901078
3965.645696	87.883162
3967.574512	87.847988
3969.503328	87.864819
3971.432144	87.893679
3973.360960	87.913443
3975.289776	87.913701
3977.218592	87.886834
3979.147408	87.868393
3981.076224	87.865282
3983.005040	87.886290
3984.933856	87.875326
3986.862672	87.848607
3988.791488	87.857196
3990.720304	87.884623
3992.649120	87.852938
3994.577936	87.827337
3996.506752	87.838505
3998.435568	87.818520
4000.364384	87.835385

##TITLE=No Description

##DATA TYPE=INFRARED SPECTRUM

##XUNITS=1/CM

##YUNITS=%T

399.264912 19.615214

401.193728 21.635516

403.122544 23.783060

405.051360 24.677342

406.980176 25.434637

408.908992 26.671663

410.837808 26.834045

412.766624 27.111297

414.695440 27.981015

416.624256 28.047631

418.553072 27.724919

420.481888 29.015719

422.410704 30.239078

424.339520 30.406452

426.268336 31.051982

428.197152 31.953018

430.125968 32.291251

432.054784 32.685073

433.983600 33.403324

435.912416 34.001013

437.841232 34.828547

439.770048 35.278654

441.698864	35.431245
443.627680	36.330984
445.556496	37.255332
447.485312	37.697531
449.414128	38.109938
451.342944	38.772054
453.271760	39.729779
455.200576	40.444506
457.129392	40.568962
459.058208	40.833214
460.987024	41.321414
462.915840	42.190295
464.844656	42.649522
466.773472	42.637393
468.702288	43.207947
470.631104	43.807257
472.559920	43.974837
474.488736	44.525412
476.417552	45.224409
478.346368	45.684717
480.275184	46.236739
482.204000	46.727203
484.132816	47.159370
486.061632	47.630726
487.990448	48.026064
489.919264	48.416484

491.848080	48.845582
493.776896	49.344732
495.705712	49.731745
497.634528	50.039081
499.563344	50.389000
501.492160	50.570173
503.420976	51.022993
505.349792	51.554649
507.278608	51.752003
509.207424	52.023336
511.136240	52.614712
513.065056	53.163448
514.993872	53.332448
516.922688	53.346838
518.851504	53.535268
520.780320	53.997800
522.709136	54.338408
524.637952	54.232930
526.566768	54.270405
528.495584	54.583412
530.424400	54.796278
532.353216	54.848351
534.282032	54.952320
536.210848	55.178332
538.139664	55.297998
540.068480	55.357446

541.997296	55.415985
543.926112	55.303151
545.854928	55.141937
547.783744	55.076114
549.712560	55.064681
551.641376	55.010437
553.570192	54.854786
555.499008	54.816854
557.427824	54.839259
559.356640	54.884990
561.285456	54.836371
563.214272	54.778276
565.143088	54.814387
567.071904	54.646352
569.000720	54.561378
570.929536	54.648300
572.858352	54.621528
574.787168	54.478086
576.715984	54.551792
578.644800	54.753999
580.573616	54.909006
582.502432	55.269557
584.431248	55.652373
586.360064	55.947248
588.288880	56.292660
590.217696	56.553173

592.146512	56.835517
594.075328	57.167502
596.004144	57.422834
597.932960	57.749149
599.861776	58.066742
601.790592	58.274252
603.719408	58.410562
605.648224	58.501006
607.577040	58.600238
609.505856	58.569076
611.434672	58.447799
613.363488	58.380550
615.292304	58.352393
617.221120	58.324324
619.149936	58.368209
621.078752	58.509655
623.007568	58.583005
624.936384	58.609838
626.865200	58.705447
628.794016	58.778922
630.722832	58.792412
632.651648	58.795612
634.580464	58.753707
636.509280	58.725547
638.438096	58.715513
640.366912	58.747524

642.295728	58.826889
644.224544	58.983540
646.153360	59.071474
648.082176	58.974801
650.010992	58.910046
651.939808	58.830982
653.868624	58.767112
655.797440	58.732953
657.726256	58.641166
659.655072	58.569989
661.583888	58.505745
663.512704	58.377809
665.441520	58.380928
667.370336	58.355832
669.299152	58.160689
671.227968	58.425595
673.156784	58.661716
675.085600	58.696126
677.014416	58.686948
678.943232	58.751861
680.872048	58.836615
682.800864	58.862967
684.729680	59.012876
686.658496	59.126291
688.587312	59.199610
690.516128	59.404373

692.444944	59.675480
694.373760	60.007821
696.302576	60.458808
698.231392	61.082085
700.160208	61.809359
702.089024	62.442030
704.017840	62.872812
705.946656	63.223873
707.875472	63.705623
709.804288	64.140666
711.733104	64.554825
713.661920	65.054962
715.590736	65.533028
717.519552	66.009194
719.448368	66.424754
721.377184	66.914768
723.306000	67.426953
725.234816	67.907968
727.163632	68.471525
729.092448	68.968653
731.021264	69.408896
732.950080	69.837602
734.878896	70.316865
736.807712	70.805819
738.736528	71.252876
740.665344	71.651302

742.594160	72.022011
744.522976	72.387534
746.451792	72.748981
748.380608	73.168758
750.309424	73.607140
752.238240	74.029677
754.167056	74.393768
756.095872	74.715425
758.024688	74.992387
759.953504	75.208602
761.882320	75.469361
763.811136	75.743603
765.739952	75.923763
767.668768	76.122915
769.597584	76.358118
771.526400	76.549910
773.455216	76.730703
775.384032	76.851301
777.312848	76.980519
779.241664	77.154857
781.170480	77.339562
783.099296	77.506212
785.028112	77.606432
786.956928	77.757937
788.885744	77.886659
790.814560	77.970187

792.743376	78.116061
794.672192	78.280182
796.601008	78.412484
798.529824	78.548717
800.458640	78.665691
802.387456	78.742959
804.316272	78.896414
806.245088	79.039762
808.173904	79.071966
810.102720	79.105711
812.031536	79.111746
813.960352	79.045135
815.889168	78.843813
817.817984	78.580010
819.746800	78.858479
821.675616	79.563053
823.604432	79.962921
825.533248	80.073930
827.462064	80.200513
829.390880	80.364130
831.319696	80.464292
833.248512	80.517550
835.177328	80.548812
837.106144	80.537436
839.034960	80.538269
840.963776	80.659691

842.892592	80.796182
844.821408	80.774087
846.750224	80.601232
848.679040	80.638899
850.607856	81.182313
852.536672	81.753803
854.465488	82.087431
856.394304	82.353790
858.323120	82.561869
860.251936	82.714695
862.180752	82.874096
864.109568	83.080649
866.038384	83.281954
867.967200	83.441637
869.896016	83.626248
871.824832	83.772155
873.753648	83.893583
875.682464	84.045174
877.611280	84.172945
879.540096	84.313717
881.468912	84.490766
883.397728	84.630884
885.326544	84.736529
887.255360	84.856436
889.184176	84.990372
891.112992	85.090388

893.041808	85.205929
894.970624	85.351517
896.899440	85.454719
898.828256	85.514147
900.757072	85.613883
902.685888	85.762564
904.614704	85.867559
906.543520	85.957697
908.472336	86.038102
910.401152	86.132152
912.329968	86.239938
914.258784	86.348235
916.187600	86.454112
918.116416	86.533074
920.045232	86.592301
921.974048	86.679563
923.902864	86.775705
925.831680	86.890097
927.760496	87.032893
929.689312	87.127431
931.618128	87.198795
933.546944	87.280392
935.475760	87.370786
937.404576	87.434774
939.333392	87.482839
941.262208	87.532795

943.191024	87.623268
945.119840	87.702974
947.048656	87.757997
948.977472	87.893375
950.906288	88.094184
952.835104	88.232910
954.763920	88.307591
956.692736	88.419416
958.621552	88.492208
960.550368	88.564307
962.479184	88.702358
964.408000	88.800887
966.336816	88.856374
968.265632	88.925830
970.194448	89.029596
972.123264	89.097129
974.052080	89.132675
975.980896	89.219208
977.909712	89.315244
979.838528	89.396492
981.767344	89.464415
983.696160	89.504240
985.624976	89.540502
987.553792	89.567550
989.482608	89.631609
991.411424	89.692430

993.340240	89.693576
995.269056	89.669910
997.197872	89.642510
999.126688	89.605822
1001.055504	89.471509
1002.984320	89.238639
1004.913136	89.186684
1006.841952	89.600683
1008.770768	90.129798
1010.699584	90.425529
1012.628400	90.552285
1014.557216	90.583526
1016.486032	90.521626
1018.414848	90.434822
1020.343664	90.378429
1022.272480	90.244501
1024.201296	90.010447
1026.130112	89.634022
1028.058928	89.088559
1029.987744	88.581353
1031.916560	88.291280
1033.845376	88.413408
1035.774192	89.033847
1037.703008	89.900773
1039.631824	90.661955
1041.560640	91.100724

1043.489456	91.292557
1045.418272	91.377164
1047.347088	91.412398
1049.275904	91.409410
1051.204720	91.405923
1053.133536	91.374087
1055.062352	91.320382
1056.991168	91.281649
1058.919984	91.255288
1060.848800	91.166388
1062.777616	91.077084
1064.706432	91.018201
1066.635248	90.902097
1068.564064	90.874882
1070.492880	90.900516
1072.421696	90.886422
1074.350512	90.846588
1076.279328	90.827519
1078.208144	90.870459
1080.136960	90.891490
1082.065776	90.886253
1083.994592	90.895423
1085.923408	90.886421
1087.852224	90.869342
1089.781040	90.870283
1091.709856	90.832253

1093.638672	90.757097
1095.567488	90.683756
1097.496304	90.538450
1099.425120	90.375737
1101.353936	90.390454
1103.282752	90.513964
1105.211568	90.543394
1107.140384	90.433568
1109.069200	90.177017
1110.998016	89.763266
1112.926832	89.290956
1114.855648	88.873932
1116.784464	88.715226
1118.713280	89.016831
1120.642096	89.658505
1122.570912	90.365885
1124.499728	90.974051
1126.428544	91.452963
1128.357360	91.779602
1130.286176	91.944157
1132.214992	92.017812
1134.143808	92.017793
1136.072624	91.938629
1138.001440	91.836070
1139.930256	91.684830
1141.859072	91.513494

1143.787888	91.303442
1145.716704	91.055769
1147.645520	90.799790
1149.574336	90.643614
1151.503152	90.696774
1153.431968	90.794971
1155.360784	90.851213
1157.289600	90.815965
1159.218416	90.713993
1161.147232	90.584273
1163.076048	90.440445
1165.004864	90.313360
1166.933680	90.269998
1168.862496	90.344871
1170.791312	90.419654
1172.720128	90.443289
1174.648944	90.385675
1176.577760	90.284977
1178.506576	90.257622
1180.435392	90.287096
1182.364208	90.321324
1184.293024	90.266085
1186.221840	90.199947
1188.150656	90.241029
1190.079472	90.325999
1192.008288	90.429237

1193.937104	90.615584
1195.865920	90.855439
1197.794736	91.018273
1199.723552	91.188867
1201.652368	91.365488
1203.581184	91.504978
1205.510000	91.656752
1207.438816	91.780538
1209.367632	91.887087
1211.296448	91.976877
1213.225264	92.016679
1215.154080	92.023171
1217.082896	92.042264
1219.011712	92.080510
1220.940528	92.091383
1222.869344	92.125668
1224.798160	92.154434
1226.726976	92.135337
1228.655792	92.089037
1230.584608	92.070212
1232.513424	92.081766
1234.442240	92.112879
1236.371056	92.183764
1238.299872	92.240412
1240.228688	92.287128
1242.157504	92.303371

1244.086320	92.267220
1246.015136	92.229705
1247.943952	92.216620
1249.872768	92.178734
1251.801584	92.127972
1253.730400	92.116653
1255.659216	92.125270
1257.588032	92.145692
1259.516848	92.170138
1261.445664	92.192313
1263.374480	92.226427
1265.303296	92.233283
1267.232112	92.169169
1269.160928	92.086649
1271.089744	91.986962
1273.018560	91.928268
1274.947376	91.904395
1276.876192	91.817566
1278.805008	91.693551
1280.733824	91.596303
1282.662640	91.536358
1284.591456	91.433684
1286.520272	91.245376
1288.449088	91.075896
1290.377904	90.967360
1292.306720	90.839764

1294.235536	90.686021
1296.164352	90.458445
1298.093168	90.252793
1300.021984	90.064421
1301.950800	89.838423
1303.879616	89.589158
1305.808432	89.347498
1307.737248	89.095593
1309.666064	88.811059
1311.594880	88.402010
1313.523696	87.968428
1315.452512	87.662584
1317.381328	87.381876
1319.310144	87.168646
1321.238960	86.998879
1323.167776	86.741422
1325.096592	86.380847
1327.025408	85.964288
1328.954224	85.533806
1330.883040	85.108601
1332.811856	84.664439
1334.740672	84.114030
1336.669488	83.421942
1338.598304	82.618817
1340.527120	82.220264
1342.455936	82.229266

1344.384752	81.925425
1346.313568	81.529373
1348.242384	81.156650
1350.171200	80.800323
1352.100016	80.525941
1354.028832	80.184819
1355.957648	79.940315
1357.886464	79.779045
1359.815280	79.529310
1361.744096	79.149182
1363.672912	79.264146
1365.601728	79.873211
1367.530544	80.196978
1369.459360	80.598132
1371.388176	81.111929
1373.316992	81.211642
1375.245808	81.649098
1377.174624	82.260724
1379.103440	82.599348
1381.032256	82.957115
1382.961072	83.321611
1384.889888	83.457765
1386.818704	83.246663
1388.747520	83.530225
1390.676336	84.025172
1392.605152	84.114172

1394.533968	83.970008
1396.462784	84.292380
1398.391600	84.622701
1400.320416	85.161734
1402.249232	85.748630
1404.178048	85.723428
1406.106864	85.978766
1408.035680	86.387678
1409.964496	86.450200
1411.893312	86.566751
1413.822128	86.786809
1415.750944	86.679451
1417.679760	86.223813
1419.608576	86.279863
1421.537392	86.969898
1423.466208	87.182767
1425.395024	87.714904
1427.323840	88.185713
1429.252656	88.158958
1431.181472	88.251503
1433.110288	88.438015
1435.039104	88.184770
1436.967920	87.874407
1438.896736	88.489707
1440.825552	88.736249
1442.754368	88.639863

1444.683184	88.609033
1446.612000	88.303782
1448.540816	88.183684
1450.469632	88.428000
1452.398448	88.414993
1454.327264	88.138503
1456.256080	87.420917
1458.184896	87.377064
1460.113712	88.240218
1462.042528	88.649891
1463.971344	88.484752
1465.900160	88.420617
1467.828976	88.637560
1469.757792	88.534887
1471.686608	87.911297
1473.615424	87.729897
1475.544240	87.935517
1477.473056	88.267170
1479.401872	88.659827
1481.330688	88.592713
1483.259504	88.724584
1485.188320	88.689722
1487.117136	88.357722
1489.045952	87.889524
1490.974768	88.119530
1492.903584	88.928391

1494.832400	88.958648
1496.761216	88.697508
1498.690032	88.839570
1500.618848	89.034843
1502.547664	89.212978
1504.476480	89.107778
1506.405296	87.963543
1508.334112	87.637075
1510.262928	88.444301
1512.191744	88.957779
1514.120560	89.087314
1516.049376	88.645054
1517.978192	88.723050
1519.907008	88.851569
1521.835824	88.927879
1523.764640	89.594059
1525.693456	89.696208
1527.622272	89.998042
1529.551088	90.598516
1531.479904	90.644877
1533.408720	90.360127
1535.337536	90.818566
1537.266352	91.088702
1539.195168	90.667639
1541.123984	90.563678
1543.052800	90.650653

1544.981616	90.764578
1546.910432	91.550754
1548.839248	91.478258
1550.768064	91.295450
1552.696880	91.637220
1554.625696	91.442712
1556.554512	91.443071
1558.483328	90.819484
1560.412144	91.146249
1562.340960	92.127496
1564.269776	92.244294
1566.198592	92.300685
1568.127408	92.216471
1570.056224	92.053233
1571.985040	92.391563
1573.913856	92.507968
1575.842672	92.278257
1577.771488	92.318688
1579.700304	92.515434
1581.629120	92.487888
1583.557936	92.442486
1585.486752	92.374539
1587.415568	92.256260
1589.344384	92.161362
1591.273200	92.064599
1593.202016	91.911928

1595.130832	91.709067
1597.059648	91.523126
1598.988464	91.258207
1600.917280	90.876119
1602.846096	90.514909
1604.774912	90.438144
1606.703728	90.639119
1608.632544	91.061778
1610.561360	91.548488
1612.490176	91.903334
1614.418992	92.048608
1616.347808	91.954374
1618.276624	92.001272
1620.205440	92.122471
1622.134256	91.945279
1624.063072	91.913749
1625.991888	92.021550
1627.920704	91.898796
1629.849520	91.956541
1631.778336	92.000592
1633.707152	91.774629
1635.635968	91.361644
1637.564784	91.462781
1639.493600	91.811338
1641.422416	91.908892
1643.351232	91.899132

1645.280048	91.581960
1647.208864	91.099268
1649.137680	91.492979
1651.066496	91.771036
1652.995312	91.174171
1654.924128	91.369818
1656.852944	92.077865
1658.781760	92.290452
1660.710576	92.233356
1662.639392	92.182532
1664.568208	92.471483
1666.497024	92.667806
1668.425840	92.501173
1670.354656	92.529962
1672.283472	92.999249
1674.212288	93.041249
1676.141104	93.036747
1678.069920	93.359092
1679.998736	93.142407
1681.927552	92.982676
1683.856368	92.670974
1685.785184	93.185543
1687.714000	93.673116
1689.642816	93.857805
1691.571632	94.367832
1693.500448	94.507863

1695.429264	94.075678
1697.358080	94.160887
1699.286896	94.219681
1701.215712	94.142029
1703.144528	94.799521
1705.073344	94.887569
1707.002160	95.127872
1708.930976	95.416204
1710.859792	95.468400
1712.788608	95.362402
1714.717424	95.017360
1716.646240	94.785729
1718.575056	94.945462
1720.503872	95.679360
1722.432688	95.928494
1724.361504	95.916518
1726.290320	96.058667
1728.219136	95.865552
1730.147952	95.568188
1732.076768	95.480117
1734.005584	95.161336
1735.934400	95.676640
1737.863216	96.008612
1739.792032	95.915143
1741.720848	95.903394
1743.649664	95.667422

1745.578480	95.724635
1747.507296	95.605702
1749.436112	95.387641
1751.364928	95.413745
1753.293744	96.015122
1755.222560	96.232454
1757.151376	95.959110
1759.080192	96.162008
1761.009008	96.039678
1762.937824	96.045378
1764.866640	96.441949
1766.795456	96.152572
1768.724272	95.896552
1770.653088	96.036646
1772.581904	95.977695
1774.510720	96.139096
1776.439536	96.303339
1778.368352	96.354262
1780.297168	96.217864
1782.225984	96.317682
1784.154800	96.271349
1786.083616	96.250808
1788.012432	96.483172
1789.941248	96.189769
1791.870064	95.838356
1793.798880	96.046500

1795.727696	96.236866
1797.656512	96.335661
1799.585328	96.275374
1801.514144	96.058442
1803.442960	96.309614
1805.371776	96.567363
1807.300592	96.547932
1809.229408	96.461928
1811.158224	96.400665
1813.087040	96.560771
1815.015856	96.708484
1816.944672	96.625996
1818.873488	96.672847
1820.802304	96.713580
1822.731120	96.559931
1824.659936	96.247960
1826.588752	96.325793
1828.517568	96.370151
1830.446384	96.245063
1832.375200	96.632060
1834.304016	96.794901
1836.232832	96.705532
1838.161648	96.705740
1840.090464	96.762217
1842.019280	96.509315
1843.948096	96.161620

1845.876912	96.356554
1847.805728	96.541968
1849.734544	96.671638
1851.663360	96.883606
1853.592176	96.907252
1855.520992	96.919284
1857.449808	96.929234
1859.378624	96.871479
1861.307440	96.840517
1863.236256	96.884392
1865.165072	96.796905
1867.093888	96.501887
1869.022704	96.288877
1870.951520	96.592669
1872.880336	96.866565
1874.809152	96.876897
1876.737968	96.960289
1878.666784	97.010017
1880.595600	96.951331
1882.524416	96.864362
1884.453232	96.831633
1886.382048	96.879927
1888.310864	96.744714
1890.239680	96.633299
1892.168496	96.842940
1894.097312	96.803138

1896.026128	96.799010
1897.954944	96.980241
1899.883760	96.927433
1901.812576	96.803736
1903.741392	96.784121
1905.670208	96.798966
1907.599024	96.797080
1909.527840	96.771649
1911.456656	96.844984
1913.385472	96.910739
1915.314288	96.958002
1917.243104	96.807488
1919.171920	96.720840
1921.100736	96.709366
1923.029552	96.567195
1924.958368	96.863481
1926.887184	96.990993
1928.816000	96.920234
1930.744816	97.029863
1932.673632	97.021614
1934.602448	96.973369
1936.531264	97.128331
1938.460080	97.153604
1940.388896	96.846857
1942.317712	96.515841
1944.246528	96.746956

1946.175344	97.072834
1948.104160	97.182234
1950.032976	97.190240
1951.961792	97.095930
1953.890608	97.103169
1955.819424	97.109947
1957.748240	97.396672
1959.677056	97.285847
1961.605872	97.097727
1963.534688	97.492243
1965.463504	97.436859
1967.392320	97.053928
1969.321136	97.266499
1971.249952	97.471167
1973.178768	97.195171
1975.107584	96.954132
1977.036400	96.914245
1978.965216	96.961489
1980.894032	97.166423
1982.822848	97.132331
1984.751664	97.190020
1986.680480	97.363832
1988.609296	97.307604
1990.538112	97.050216
1992.466928	97.059759
1994.395744	97.321216

1996.324560	97.271489
1998.253376	97.368112
2000.182192	97.381228
2002.111008	97.400413
2004.039824	97.354248
2005.968640	97.155891
2007.897456	97.064132
2009.826272	97.347040
2011.755088	97.465358
2013.683904	97.238802
2015.612720	97.425853
2017.541536	97.274737
2019.470352	97.013001
2021.399168	97.151279
2023.327984	97.154079
2025.256800	97.069381
2027.185616	97.148748
2029.114432	97.049105
2031.043248	96.894652
2032.972064	97.070043
2034.900880	97.150336
2036.829696	97.101909
2038.758512	97.059151
2040.687328	97.034884
2042.616144	97.075861
2044.544960	97.080164

2046.473776	97.055863
2048.402592	97.163805
2050.331408	97.488632
2052.260224	97.368814
2054.189040	97.148002
2056.117856	97.136250
2058.046672	97.110958
2059.975488	97.076647
2061.904304	96.994382
2063.833120	97.087972
2065.761936	97.177640
2067.690752	97.230877
2069.619568	97.370574
2071.548384	97.344688
2073.477200	97.272450
2075.406016	97.279281
2077.334832	97.295018
2079.263648	97.352908
2081.192464	97.278669
2083.121280	97.342695
2085.050096	97.421758
2086.978912	97.357978
2088.907728	97.349671
2090.836544	97.243335
2092.765360	97.354500
2094.694176	97.462051

2096.622992	97.352259
2098.551808	97.290775
2100.480624	97.211195
2102.409440	97.256389
2104.338256	97.359958
2106.267072	97.271239
2108.195888	97.219510
2110.124704	97.256063
2112.053520	97.384106
2113.982336	97.393158
2115.911152	97.347310
2117.839968	97.550520
2119.768784	97.531460
2121.697600	97.422094
2123.626416	97.498521
2125.555232	97.612665
2127.484048	97.669811
2129.412864	97.468160
2131.341680	97.487910
2133.270496	97.588471
2135.199312	97.454985
2137.128128	97.459411
2139.056944	97.575111
2140.985760	97.845414
2142.914576	97.961567
2144.843392	97.909439

2146.772208	97.796686
2148.701024	97.963243
2150.629840	98.102153
2152.558656	97.840222
2154.487472	97.777324
2156.416288	98.171586
2158.345104	98.363135
2160.273920	98.003044
2162.202736	97.899821
2164.131552	98.149776
2166.060368	98.237021
2167.989184	97.927532
2169.918000	97.797016
2171.846816	97.714679
2173.775632	97.701957
2175.704448	97.770614
2177.633264	97.718457
2179.562080	97.703027
2181.490896	97.713921
2183.419712	97.616526
2185.348528	97.579526
2187.277344	97.741698
2189.206160	97.836898
2191.134976	97.867369
2193.063792	97.828003
2194.992608	97.637193

2196.921424	97.644727
2198.850240	97.797711
2200.779056	97.715334
2202.707872	97.600205
2204.636688	97.864337
2206.565504	98.004805
2208.494320	97.895208
2210.423136	98.009313
2212.351952	97.977453
2214.280768	98.098951
2216.209584	98.127400
2218.138400	97.841996
2220.067216	97.864069
2221.996032	97.893590
2223.924848	97.827665
2225.853664	97.745804
2227.782480	97.714819
2229.711296	97.763376
2231.640112	97.777458
2233.568928	97.879301
2235.497744	97.743099
2237.426560	97.683303
2239.355376	97.929417
2241.284192	97.859393
2243.213008	97.864141
2245.141824	97.914539

2247.070640	97.777319
2248.999456	97.735238
2250.928272	97.717076
2252.857088	97.848282
2254.785904	97.893010
2256.714720	97.716729
2258.643536	97.690495
2260.572352	97.836520
2262.501168	97.900610
2264.429984	97.933976
2266.358800	97.884788
2268.287616	97.856752
2270.216432	97.941815
2272.145248	97.970320
2274.074064	97.947967
2276.002880	97.896502
2277.931696	97.863707
2279.860512	97.794450
2281.789328	97.798908
2283.718144	97.896175
2285.646960	97.909721
2287.575776	97.842797
2289.504592	97.740174
2291.433408	97.663336
2293.362224	97.551129
2295.291040	97.396926

2297.219856	97.225836
2299.148672	97.158447
2301.077488	97.081982
2303.006304	96.902620
2304.935120	96.786876
2306.863936	96.706781
2308.792752	96.558372
2310.721568	96.488961
2312.650384	96.600644
2314.579200	96.810017
2316.508016	97.029251
2318.436832	97.126386
2320.365648	97.160578
2322.294464	97.334946
2324.223280	97.752562
2326.152096	98.159847
2328.080912	98.441034
2330.009728	98.732906
2331.938544	99.040540
2333.867360	99.455518
2335.796176	99.829661
2337.724992	100.051074
2339.653808	100.113376
2341.582624	100.024291
2343.511440	99.664759
2345.440256	99.110752

2347.369072	98.287830
2349.297888	97.868232
2351.226704	98.359618
2353.155520	99.146336
2355.084336	99.646100
2357.013152	100.095006
2358.941968	100.329404
2360.870784	100.384865
2362.799600	100.374111
2364.728416	100.121000
2366.657232	99.675937
2368.586048	99.227445
2370.514864	98.748842
2372.443680	98.221110
2374.372496	97.754141
2376.301312	97.370383
2378.230128	97.169326
2380.158944	97.157831
2382.087760	97.307253
2384.016576	97.598724
2385.945392	97.902382
2387.874208	98.049379
2389.803024	98.077663
2391.731840	98.073981
2393.660656	98.123678
2395.589472	98.154598

2397.518288	98.164495
2399.447104	98.201652
2401.375920	98.196838
2403.304736	98.214621
2405.233552	98.279857
2407.162368	98.290139
2409.091184	98.281214
2411.020000	98.277428
2412.948816	98.274208
2414.877632	98.302421
2416.806448	98.315662
2418.735264	98.333689
2420.664080	98.374154
2422.592896	98.409509
2424.521712	98.378722
2426.450528	98.324167
2428.379344	98.314721
2430.308160	98.333224
2432.236976	98.348994
2434.165792	98.384932
2436.094608	98.415164
2438.023424	98.440650
2439.952240	98.423304
2441.881056	98.404026
2443.809872	98.429436
2445.738688	98.440308

2447.667504	98.479863
2449.596320	98.517221
2451.525136	98.500550
2453.453952	98.501227
2455.382768	98.478888
2457.311584	98.478278
2459.240400	98.515317
2461.169216	98.516733
2463.098032	98.478440
2465.026848	98.476075
2466.955664	98.525340
2468.884480	98.566361
2470.813296	98.602488
2472.742112	98.579873
2474.670928	98.480160
2476.599744	98.456866
2478.528560	98.472387
2480.457376	98.454683
2482.386192	98.463956
2484.315008	98.543296
2486.243824	98.596717
2488.172640	98.560539
2490.101456	98.527395
2492.030272	98.524955
2493.959088	98.571236
2495.887904	98.513730

2497.816720	98.461762
2499.745536	98.468959
2501.674352	98.425666
2503.603168	98.427219
2505.531984	98.466472
2507.460800	98.451302
2509.389616	98.458730
2511.318432	98.527551
2513.247248	98.526901
2515.176064	98.511219
2517.104880	98.479810
2519.033696	98.453994
2520.962512	98.462695
2522.891328	98.465310
2524.820144	98.469164
2526.748960	98.496499
2528.677776	98.521158
2530.606592	98.474806
2532.535408	98.463029
2534.464224	98.534946
2536.393040	98.530750
2538.321856	98.467011
2540.250672	98.477440
2542.179488	98.486124
2544.108304	98.423020
2546.037120	98.413896

2547.965936	98.445614
2549.894752	98.522808
2551.823568	98.527528
2553.752384	98.435115
2555.681200	98.488564
2557.610016	98.506235
2559.538832	98.464955
2561.467648	98.453576
2563.396464	98.475982
2565.325280	98.498054
2567.254096	98.443266
2569.182912	98.438815
2571.111728	98.441895
2573.040544	98.469861
2574.969360	98.522061
2576.898176	98.502652
2578.826992	98.475021
2580.755808	98.436912
2582.684624	98.392515
2584.613440	98.392596
2586.542256	98.396736
2588.471072	98.414967
2590.399888	98.428363
2592.328704	98.419757
2594.257520	98.416034
2596.186336	98.419268

2598.115152	98.432031
2600.043968	98.409634
2601.972784	98.363485
2603.901600	98.332467
2605.830416	98.353386
2607.759232	98.378284
2609.688048	98.364095
2611.616864	98.337535
2613.545680	98.305755
2615.474496	98.270076
2617.403312	98.254249
2619.332128	98.267173
2621.260944	98.279127
2623.189760	98.264522
2625.118576	98.269676
2627.047392	98.321633
2628.976208	98.327795
2630.905024	98.275430
2632.833840	98.249200
2634.762656	98.244143
2636.691472	98.237516
2638.620288	98.213589
2640.549104	98.197071
2642.477920	98.214199
2644.406736	98.221019
2646.335552	98.201148

2648.264368	98.192522
2650.193184	98.199653
2652.122000	98.193475
2654.050816	98.184140
2655.979632	98.171156
2657.908448	98.136325
2659.837264	98.108941
2661.766080	98.126260
2663.694896	98.141456
2665.623712	98.118664
2667.552528	98.101870
2669.481344	98.104232
2671.410160	98.108893
2673.338976	98.112825
2675.267792	98.082303
2677.196608	98.065708
2679.125424	98.084968
2681.054240	98.073897
2682.983056	98.070954
2684.911872	98.094788
2686.840688	98.097675
2688.769504	98.078240
2690.698320	98.076330
2692.627136	98.093714
2694.555952	98.082650
2696.484768	98.048179

2698.413584	98.040098
2700.342400	98.048676
2702.271216	98.058927
2704.200032	98.074599
2706.128848	98.068781
2708.057664	98.031000
2709.986480	97.994190
2711.915296	97.997699
2713.844112	98.025196
2715.772928	98.029307
2717.701744	98.021015
2719.630560	98.031414
2721.559376	98.026973
2723.488192	98.004056
2725.417008	97.998718
2727.345824	97.985374
2729.274640	97.942787
2731.203456	97.936675
2733.132272	97.949523
2735.061088	97.944148
2736.989904	97.945368
2738.918720	97.926624
2740.847536	97.922801
2742.776352	97.904331
2744.705168	97.869084
2746.633984	97.862061

2748.562800	97.854967
2750.491616	97.854121
2752.420432	97.850804
2754.349248	97.850681
2756.278064	97.838660
2758.206880	97.792871
2760.135696	97.762027
2762.064512	97.773064
2763.993328	97.785653
2765.922144	97.755732
2767.850960	97.721153
2769.779776	97.701895
2771.708592	97.685476
2773.637408	97.686638
2775.566224	97.691921
2777.495040	97.694500
2779.423856	97.676500
2781.352672	97.668481
2783.281488	97.646185
2785.210304	97.608920
2787.139120	97.616235
2789.067936	97.606216
2790.996752	97.573244
2792.925568	97.548531
2794.854384	97.533057
2796.783200	97.546349

2798.712016	97.533048
2800.640832	97.480126
2802.569648	97.467628
2804.498464	97.468813
2806.427280	97.449659
2808.356096	97.456052
2810.284912	97.425137
2812.213728	97.370802
2814.142544	97.357184
2816.071360	97.351167
2818.000176	97.344878
2819.928992	97.334058
2821.857808	97.305314
2823.786624	97.281994
2825.715440	97.262320
2827.644256	97.265031
2829.573072	97.278788
2831.501888	97.277538
2833.430704	97.278491
2835.359520	97.263398
2837.288336	97.237420
2839.217152	97.246290
2841.145968	97.245031
2843.074784	97.203435
2845.003600	97.174531
2846.932416	97.128436

2848.861232	97.093821
2850.790048	97.084459
2852.718864	97.062275
2854.647680	97.053046
2856.576496	97.042662
2858.505312	97.033636
2860.434128	97.036815
2862.362944	97.037478
2864.291760	97.036219
2866.220576	97.022096
2868.149392	97.019683
2870.078208	97.016494
2872.007024	96.988489
2873.935840	96.939510
2875.864656	96.930957
2877.793472	96.918314
2879.722288	96.870473
2881.651104	96.874677
2883.579920	96.880873
2885.508736	96.884805
2887.437552	96.851712
2889.366368	96.782392
2891.295184	96.744124
2893.224000	96.759612
2895.152816	96.757559
2897.081632	96.733123

2899.010448	96.728566
2900.939264	96.677747
2902.868080	96.642212
2904.796896	96.641851
2906.725712	96.634484
2908.654528	96.630512
2910.583344	96.605729
2912.512160	96.592817
2914.440976	96.578986
2916.369792	96.549077
2918.298608	96.579107
2920.227424	96.560654
2922.156240	96.460335
2924.085056	96.432755
2926.013872	96.464776
2927.942688	96.476315
2929.871504	96.482285
2931.800320	96.463800
2933.729136	96.439459
2935.657952	96.451553
2937.586768	96.449416
2939.515584	96.420549
2941.444400	96.435237
2943.373216	96.475877
2945.302032	96.463542
2947.230848	96.406232

2949.159664	96.380911
2951.088480	96.390291
2953.017296	96.375532
2954.946112	96.359296
2956.874928	96.362116
2958.803744	96.356421
2960.732560	96.344977
2962.661376	96.327070
2964.590192	96.285449
2966.519008	96.296944
2968.447824	96.344507
2970.376640	96.331539
2972.305456	96.344828
2974.234272	96.350431
2976.163088	96.286331
2978.091904	96.266220
2980.020720	96.289684
2981.949536	96.268789
2983.878352	96.244368
2985.807168	96.234821
2987.735984	96.183766
2989.664800	96.179614
2991.593616	96.210036
2993.522432	96.162790
2995.451248	96.175990
2997.380064	96.215031

2999.308880	96.170634
3001.237696	96.145600
3003.166512	96.128274
3005.095328	96.114161
3007.024144	96.109699
3008.952960	96.091392
3010.881776	96.054097
3012.810592	96.028664
3014.739408	96.013460
3016.668224	96.034510
3018.597040	96.064910
3020.525856	96.041729
3022.454672	96.021913
3024.383488	96.016873
3026.312304	96.043701
3028.241120	96.002527
3030.169936	95.918528
3032.098752	95.906079
3034.027568	95.898990
3035.956384	95.907180
3037.885200	95.887616
3039.814016	95.850631
3041.742832	95.866169
3043.671648	95.867390
3045.600464	95.806166
3047.529280	95.772344

3049.458096	95.798635
3051.386912	95.755831
3053.315728	95.709630
3055.244544	95.718550
3057.173360	95.730128
3059.102176	95.724641
3061.030992	95.708107
3062.959808	95.704703
3064.888624	95.685268
3066.817440	95.646366
3068.746256	95.634348
3070.675072	95.684330
3072.603888	95.669443
3074.532704	95.601007
3076.461520	95.586578
3078.390336	95.592931
3080.319152	95.601780
3082.247968	95.592038
3084.176784	95.575919
3086.105600	95.570033
3088.034416	95.540399
3089.963232	95.514789
3091.892048	95.481799
3093.820864	95.423745
3095.749680	95.416860
3097.678496	95.399205

3099.607312	95.367195
3101.536128	95.343627
3103.464944	95.340536
3105.393760	95.387348
3107.322576	95.396850
3109.251392	95.372630
3111.180208	95.333584
3113.109024	95.302583
3115.037840	95.334557
3116.966656	95.330050
3118.895472	95.297241
3120.824288	95.258211
3122.753104	95.223004
3124.681920	95.243419
3126.610736	95.232221
3128.539552	95.214959
3130.468368	95.212740
3132.397184	95.206700
3134.326000	95.211935
3136.254816	95.218825
3138.183632	95.214465
3140.112448	95.228361
3142.041264	95.204672
3143.970080	95.154307
3145.898896	95.139987
3147.827712	95.122115

3149.756528	95.113000
3151.685344	95.149208
3153.614160	95.147857
3155.542976	95.101982
3157.471792	95.084031
3159.400608	95.035636
3161.329424	95.031551
3163.258240	95.056008
3165.187056	95.027249
3167.115872	94.987367
3169.044688	94.981591
3170.973504	94.985089
3172.902320	94.924714
3174.831136	94.936912
3176.759952	94.985496
3178.688768	94.953228
3180.617584	94.941982
3182.546400	94.903530
3184.475216	94.887047
3186.404032	94.938082
3188.332848	94.940815
3190.261664	94.896345
3192.190480	94.870852
3194.119296	94.887103
3196.048112	94.851376
3197.976928	94.824195

3199.905744	94.833332
3201.834560	94.836033
3203.763376	94.845364
3205.692192	94.856591
3207.621008	94.884069
3209.549824	94.885306
3211.478640	94.849725
3213.407456	94.813243
3215.336272	94.757426
3217.265088	94.714344
3219.193904	94.715573
3221.122720	94.744198
3223.051536	94.726724
3224.980352	94.709055
3226.909168	94.715632
3228.837984	94.696515
3230.766800	94.680058
3232.695616	94.682169
3234.624432	94.677823
3236.553248	94.666320
3238.482064	94.693777
3240.410880	94.646183
3242.339696	94.632654
3244.268512	94.664680
3246.197328	94.687109
3248.126144	94.687488

3250.054960	94.624900
3251.983776	94.628015
3253.912592	94.593284
3255.841408	94.541995
3257.770224	94.567722
3259.699040	94.580660
3261.627856	94.605166
3263.556672	94.606150
3265.485488	94.547801
3267.414304	94.564822
3269.343120	94.607879
3271.271936	94.589967
3273.200752	94.551637
3275.129568	94.542350
3277.058384	94.543788
3278.987200	94.537668
3280.916016	94.531992
3282.844832	94.517340
3284.773648	94.497917
3286.702464	94.483660
3288.631280	94.491243
3290.560096	94.479571
3292.488912	94.465870
3294.417728	94.432467
3296.346544	94.409450
3298.275360	94.458287

3300.204176	94.479555
3302.132992	94.478413
3304.061808	94.463762
3305.990624	94.496413
3307.919440	94.509933
3309.848256	94.467718
3311.777072	94.440609
3313.705888	94.434053
3315.634704	94.425135
3317.563520	94.377137
3319.492336	94.406289
3321.421152	94.435921
3323.349968	94.410553
3325.278784	94.406087
3327.207600	94.410511
3329.136416	94.440279
3331.065232	94.398109
3332.994048	94.348282
3334.922864	94.395551
3336.851680	94.406119
3338.780496	94.367287
3340.709312	94.337076
3342.638128	94.347352
3344.566944	94.347465
3346.495760	94.319758
3348.424576	94.301954

3350.353392	94.306439
3352.282208	94.294159
3354.211024	94.290303
3356.139840	94.262619
3358.068656	94.209872
3359.997472	94.219674
3361.926288	94.227305
3363.855104	94.218552
3365.783920	94.215795
3367.712736	94.199460
3369.641552	94.214022
3371.570368	94.159895
3373.499184	94.111165
3375.428000	94.163086
3377.356816	94.190349
3379.285632	94.157197
3381.214448	94.134916
3383.143264	94.158695
3385.072080	94.231374
3387.000896	94.281323
3388.929712	94.280034
3390.858528	94.328831
3392.787344	94.311449
3394.716160	94.300361
3396.644976	94.339121
3398.573792	94.406100

3400.502608	94.530656
3402.431424	94.580865
3404.360240	94.565626
3406.289056	94.600353
3408.217872	94.616195
3410.146688	94.649048
3412.075504	94.761735
3414.004320	94.821842
3415.933136	94.892696
3417.861952	94.949166
3419.790768	94.920895
3421.719584	94.948530
3423.648400	95.050075
3425.577216	95.159630
3427.506032	95.259773
3429.434848	95.365952
3431.363664	95.468110
3433.292480	95.552565
3435.221296	95.654055
3437.150112	95.749625
3439.078928	95.833259
3441.007744	95.902935
3442.936560	95.943922
3444.865376	96.015116
3446.794192	96.040898
3448.723008	96.179703

3450.651824	96.428402
3452.580640	96.579516
3454.509456	96.658948
3456.438272	96.702733
3458.367088	96.744614
3460.295904	96.856431
3462.224720	96.941848
3464.153536	97.014216
3466.082352	97.166893
3468.011168	97.299075
3469.939984	97.389802
3471.868800	97.496250
3473.797616	97.644914
3475.726432	97.763382
3477.655248	97.889724
3479.584064	97.948292
3481.512880	97.944472
3483.441696	98.057280
3485.370512	98.220862
3487.299328	98.336047
3489.228144	98.478016
3491.156960	98.633718
3493.085776	98.722977
3495.014592	98.754840
3496.943408	98.788703
3498.872224	98.972688

3500.801040	99.036028
3502.729856	98.969886
3504.658672	99.120224
3506.587488	99.397173
3508.516304	99.475625
3510.445120	99.542495
3512.373936	99.836671
3514.302752	100.035634
3516.231568	100.115517
3518.160384	100.174113
3520.089200	100.280737
3522.018016	100.273484
3523.946832	100.156408
3525.875648	100.319392
3527.804464	100.510357
3529.733280	100.770382
3531.662096	101.068436
3533.590912	101.220969
3535.519728	101.210817
3537.448544	101.270567
3539.377360	101.522318
3541.306176	101.609379
3543.234992	101.533146
3545.163808	101.277002
3547.092624	101.267846
3549.021440	101.792638

3550.950256	102.046418
3552.879072	101.989736
3554.807888	102.207729
3556.736704	102.449055
3558.665520	102.475840
3560.594336	102.428804
3562.523152	102.453980
3564.451968	102.316920
3566.380784	101.741061
3568.309600	101.650300
3570.238416	102.534179
3572.167232	103.170887
3574.096048	103.326810
3576.024864	103.423624
3577.953680	103.481064
3579.882496	103.493880
3581.811312	103.548687
3583.740128	103.591690
3585.668944	103.309668
3587.597760	102.761022
3589.526576	103.147967
3591.455392	103.914904
3593.384208	103.905283
3595.313024	103.807593
3597.241840	104.177470
3599.170656	104.549172

3601.099472	104.615468
3603.028288	104.815527
3604.957104	104.952882
3606.885920	104.716266
3608.814736	104.419616
3610.743552	104.630436
3612.672368	104.758453
3614.601184	104.795269
3616.530000	105.256968
3618.458816	105.327427
3620.387632	105.253952
3622.316448	105.857189
3624.245264	106.273790
3626.174080	106.294934
3628.102896	106.065685
3630.031712	105.421254
3631.960528	106.208267
3633.889344	106.756630
3635.818160	106.987282
3637.746976	107.343732
3639.675792	107.368390
3641.604608	107.328882
3643.533424	107.356737
3645.462240	107.392735
3647.391056	106.893876
3649.319872	106.679490

3651.248688	106.723880
3653.177504	107.669059
3655.106320	108.353557
3657.035136	108.081004
3658.963952	108.455679
3660.892768	108.807455
3662.821584	108.877019
3664.750400	109.015057
3666.679216	108.986628
3668.608032	108.884876
3670.536848	108.275868
3672.465664	108.448687
3674.394480	109.008961
3676.323296	108.304573
3678.252112	108.759819
3680.180928	109.263303
3682.109744	109.496963
3684.038560	109.661295
3685.967376	109.649927
3687.896192	109.643463
3689.825008	109.110637
3691.753824	109.220560
3693.682640	109.646191
3695.611456	109.929855
3697.540272	109.830806
3699.469088	109.980116

3701.397904	109.915640
3703.326720	109.813203
3705.255536	110.101920
3707.184352	110.177632
3709.113168	110.012801
3711.041984	109.816309
3712.970800	109.504261
3714.899616	109.697797
3716.828432	110.207904
3718.757248	110.193557
3720.686064	110.146118
3722.614880	109.899914
3724.543696	109.661389
3726.472512	109.672044
3728.401328	109.796001
3730.330144	110.249745
3732.258960	109.917585
3734.187776	109.010064
3736.116592	108.485200
3738.045408	109.081484
3739.974224	109.541770
3741.903040	109.849800
3743.831856	109.935176
3745.760672	108.628323
3747.689488	109.495629
3749.618304	109.924233

3751.547120	109.467522
3753.475936	109.509361
3755.404752	109.937835
3757.333568	110.193768
3759.262384	110.096355
3761.191200	110.069163
3763.120016	110.350658
3765.048832	110.300279
3766.977648	110.069450
3768.906464	110.449410
3770.835280	110.115661
3772.764096	110.262918
3774.692912	110.580040
3776.621728	110.573520
3778.550544	110.575927
3780.479360	110.255066
3782.408176	110.428243
3784.336992	110.604421
3786.265808	110.486647
3788.194624	110.629170
3790.123440	110.686247
3792.052256	110.642705
3793.981072	110.740991
3795.909888	110.600088
3797.838704	110.133615
3799.767520	110.673564

3801.696336	110.388958
3803.625152	109.847880
3805.553968	110.728756
3807.482784	110.462405
3809.411600	110.416492
3811.340416	110.759543
3813.269232	110.730913
3815.198048	110.935190
3817.126864	110.090078
3819.055680	110.278701
3820.984496	110.353947
3822.913312	109.850287
3824.842128	110.576662
3826.770944	110.524010
3828.699760	110.431128
3830.628576	110.661417
3832.557392	110.401403
3834.486208	110.338964
3836.415024	110.472986
3838.343840	110.066501
3840.272656	109.705912
3842.201472	110.143695
3844.130288	110.329946
3846.059104	110.516203
3847.987920	110.692339
3849.916736	110.479652

3851.845552	110.606589
3853.774368	109.947735
3855.703184	109.511350
3857.632000	110.099884
3859.560816	110.595429
3861.489632	110.571765
3863.418448	110.369994
3865.347264	110.013139
3867.276080	110.265584
3869.204896	110.424548
3871.133712	109.718583
3873.062528	110.326040
3874.991344	110.321911
3876.920160	110.497496
3878.848976	110.754128
3880.777792	109.925568
3882.706608	110.067305
3884.635424	110.522405
3886.564240	110.067024
3888.493056	110.574757
3890.421872	110.894554
3892.350688	110.274075
3894.279504	110.510813
3896.208320	110.802964
3898.137136	110.592193
3900.065952	110.006516

3901.994768	109.879950
3903.923584	109.905241
3905.852400	110.073214
3907.781216	110.747096
3909.710032	111.208309
3911.638848	111.226565
3913.567664	111.190359
3915.496480	111.004935
3917.425296	110.596826
3919.354112	110.687144
3921.282928	110.880754
3923.211744	110.909933
3925.140560	110.719179
3927.069376	110.854270
3928.998192	111.076747
3930.927008	110.824891
3932.855824	110.677130
3934.784640	111.040547
3936.713456	111.352080
3938.642272	111.315913
3940.571088	111.327455
3942.499904	110.980283
3944.428720	110.915831
3946.357536	111.112081
3948.286352	110.928175
3950.215168	110.957311

3952.143984	111.250037
3954.072800	111.422475
3956.001616	111.441792
3957.930432	111.422335
3959.859248	111.432168
3961.788064	111.387652
3963.716880	111.404910
3965.645696	111.545500
3967.574512	111.574580
3969.503328	111.522405
3971.432144	111.551815
3973.360960	111.570556
3975.289776	111.518952
3977.218592	111.516419
3979.147408	111.594881
3981.076224	111.616099
3983.005040	111.599436
3984.933856	111.654154
3986.862672	111.686760
3988.791488	111.652543
3990.720304	111.582430
3992.649120	111.614044
3994.577936	111.684366
3996.506752	111.692012
3998.435568	111.747158
4000.364384	111.760964

##TITLE=No Description

##DATA TYPE=INFRARED SPECTRUM

##XUNITS=1/CM

##YUNITS=%T

399.264912 28.528057

401.193728 30.556315

403.122544 32.299617

405.051360 33.180901

406.980176 33.835731

408.908992 34.601773

410.837808 35.945365

412.766624 36.793403

414.695440 36.733880

416.624256 36.459878

418.553072 36.056752

420.481888 37.224457

422.410704 38.225578

424.339520 38.469373

426.268336 39.011489

428.197152 39.854358

430.125968 40.069313

432.054784 40.270317

433.983600 40.799752

435.912416 41.089838

437.841232 41.744603

439.770048 42.406165

441.698864	42.731444
443.627680	43.158188
445.556496	43.742922
447.485312	44.590651
449.414128	45.497461
451.342944	45.945538
453.271760	46.886421
455.200576	47.623255
457.129392	47.598548
459.058208	48.155230
460.987024	48.603576
462.915840	49.117810
464.844656	49.933853
466.773472	50.139909
468.702288	50.152801
470.631104	50.220345
472.559920	50.237181
474.488736	50.811923
476.417552	51.190145
478.346368	51.130828
480.275184	51.109487
482.204000	51.067692
484.132816	51.184267
486.061632	51.374738
487.990448	51.319782
489.919264	51.288379

491.848080	51.283124
493.776896	51.291497
495.705712	51.427889
497.634528	51.518047
499.563344	51.478284
501.492160	51.217997
503.420976	51.143950
505.349792	51.296940
507.278608	51.295629
509.207424	51.066333
511.136240	50.904747
513.065056	51.057206
514.993872	51.140525
516.922688	50.952786
518.851504	50.724611
520.780320	50.688274
522.709136	50.859633
524.637952	50.686009
526.566768	50.491241
528.495584	50.472833
530.424400	50.285667
532.353216	50.301943
534.282032	50.363885
536.210848	50.380168
538.139664	50.330145
540.068480	50.259864

541.997296	50.227474
543.926112	50.123496
545.854928	50.014635
547.783744	49.988498
549.712560	49.981185
551.641376	49.921533
553.570192	49.878333
555.499008	49.968427
557.427824	50.037842
559.356640	49.997064
561.285456	50.092104
563.214272	50.295055
565.143088	50.431251
567.071904	50.581791
569.000720	50.826513
570.929536	51.062097
572.858352	51.445514
574.787168	51.775157
576.715984	52.163441
578.644800	52.640973
580.573616	53.071496
582.502432	53.549423
584.431248	54.008277
586.360064	54.531576
588.288880	55.106440
590.217696	55.576664

592.146512	55.974182
594.075328	56.406457
596.004144	56.775272
597.932960	57.164462
599.861776	57.579154
601.790592	57.830409
603.719408	58.046981
605.648224	58.289440
607.577040	58.443652
609.505856	58.488763
611.434672	58.426313
613.363488	58.426926
615.292304	58.281047
617.221120	58.083810
619.149936	58.007580
621.078752	57.936941
623.007568	57.880034
624.936384	57.781922
626.865200	57.730447
628.794016	57.727468
630.722832	57.621572
632.651648	57.476371
634.580464	57.400832
636.509280	57.339566
638.438096	57.272854
640.366912	57.147358

642.295728	57.055850
644.224544	57.059956
646.153360	57.063533
648.082176	57.023491
650.010992	56.937573
651.939808	56.809815
653.868624	56.681946
655.797440	56.623906
657.726256	56.619499
659.655072	56.580511
661.583888	56.511815
663.512704	56.461164
665.441520	56.411396
667.370336	56.324980
669.299152	56.301125
671.227968	56.544858
673.156784	56.690833
675.085600	56.745071
677.014416	56.853560
678.943232	56.906841
680.872048	56.934121
682.800864	56.939803
684.729680	57.046411
686.658496	57.177808
688.587312	57.225313
690.516128	57.385329

692.444944	57.602515
694.373760	57.762975
696.302576	57.972112
698.231392	58.232511
700.160208	58.513626
702.089024	58.744745
704.017840	58.948912
705.946656	59.126715
707.875472	59.365295
709.804288	59.608022
711.733104	59.857072
713.661920	60.126590
715.590736	60.366555
717.519552	60.568877
719.448368	60.716034
721.377184	60.967511
723.306000	61.246039
725.234816	61.474728
727.163632	61.740657
729.092448	62.007011
731.021264	62.252924
732.950080	62.509152
734.878896	62.786552
736.807712	63.056176
738.736528	63.293188
740.665344	63.492291

742.594160	63.729585
744.522976	63.955908
746.451792	64.189311
748.380608	64.513517
750.309424	64.803350
752.238240	65.074699
754.167056	65.361408
756.095872	65.601446
758.024688	65.826188
759.953504	66.029025
761.882320	66.215775
763.811136	66.431527
765.739952	66.657720
767.668768	66.890922
769.597584	67.068477
771.526400	67.224103
773.455216	67.426164
775.384032	67.637186
777.312848	67.806579
779.241664	67.960248
781.170480	68.151042
783.099296	68.301388
785.028112	68.426600
786.956928	68.666692
788.885744	68.855108
790.814560	68.946224

792.743376	69.123618
794.672192	69.302883
796.601008	69.457607
798.529824	69.640476
800.458640	69.793678
802.387456	69.906397
804.316272	70.095964
806.245088	70.245086
808.173904	70.326853
810.102720	70.462285
812.031536	70.570650
813.960352	70.651259
815.889168	70.711078
817.817984	70.781007
819.746800	70.920342
821.675616	71.080931
823.604432	71.246949
825.533248	71.375617
827.462064	71.503800
829.390880	71.662635
831.319696	71.799147
833.248512	71.932965
835.177328	72.065913
837.106144	72.186396
839.034960	72.294804
840.963776	72.383065

842.892592	72.501103
844.821408	72.662500
846.750224	72.874538
848.679040	73.113669
850.607856	73.298839
852.536672	73.449583
854.465488	73.642444
856.394304	73.821505
858.323120	73.953998
860.251936	74.094437
862.180752	74.240343
864.109568	74.360829
866.038384	74.491163
867.967200	74.617900
869.896016	74.726851
871.824832	74.822649
873.753648	74.901410
875.682464	74.999818
877.611280	75.077793
879.540096	75.173353
881.468912	75.311707
883.397728	75.411500
885.326544	75.476670
887.255360	75.547630
889.184176	75.625341
891.112992	75.690169

893.041808	75.784735
894.970624	75.888642
896.899440	75.969222
898.828256	76.023954
900.757072	76.109998
902.685888	76.217781
904.614704	76.274250
906.543520	76.359160
908.472336	76.427824
910.401152	76.493734
912.329968	76.561037
914.258784	76.607777
916.187600	76.682636
918.116416	76.738678
920.045232	76.762635
921.974048	76.819789
923.902864	76.890674
925.831680	76.974950
927.760496	77.029027
929.689312	77.067977
931.618128	77.136994
933.546944	77.190842
935.475760	77.213356
937.404576	77.231910
939.333392	77.259970
941.262208	77.276734

943.191024	77.360384
945.119840	77.449478
947.048656	77.506767
948.977472	77.587855
950.906288	77.681897
952.835104	77.727873
954.763920	77.746833
956.692736	77.787265
958.621552	77.804390
960.550368	77.823641
962.479184	77.885349
964.408000	77.959159
966.336816	78.026329
968.265632	78.032909
970.194448	78.041274
972.123264	78.066512
974.052080	78.086790
975.980896	78.156117
977.909712	78.220744
979.838528	78.273552
981.767344	78.323156
983.696160	78.333184
985.624976	78.338167
987.553792	78.338841
989.482608	78.366451
991.411424	78.423856

993.340240	78.419195
995.269056	78.400878
997.197872	78.419826
999.126688	78.447231
1001.055504	78.425676
1002.984320	78.367445
1004.913136	78.306249
1006.841952	78.395242
1008.770768	78.628629
1010.699584	78.827572
1012.628400	78.964126
1014.557216	79.012064
1016.486032	78.976374
1018.414848	78.952142
1020.343664	78.922914
1022.272480	78.841458
1024.201296	78.716381
1026.130112	78.493643
1028.058928	78.213932
1029.987744	78.062748
1031.916560	78.183772
1033.845376	78.540514
1035.774192	78.924829
1037.703008	79.159260
1039.631824	79.263210
1041.560640	79.296516

1043.489456	79.303783
1045.418272	79.302400
1047.347088	79.292145
1049.275904	79.263117
1051.204720	79.252477
1053.133536	79.251811
1055.062352	79.232252
1056.991168	79.231090
1058.919984	79.241286
1060.848800	79.224200
1062.777616	79.201387
1064.706432	79.197230
1066.635248	79.142811
1068.564064	79.128374
1070.492880	79.141756
1072.421696	79.099356
1074.350512	79.053848
1076.279328	79.038182
1078.208144	79.040363
1080.136960	79.036341
1082.065776	79.036555
1083.994592	79.058141
1085.923408	79.054793
1087.852224	79.004699
1089.781040	78.977604
1091.709856	78.960520

1093.638672	78.917821
1095.567488	78.865273
1097.496304	78.815771
1099.425120	78.769598
1101.353936	78.759642
1103.282752	78.756110
1105.211568	78.708313
1107.140384	78.664481
1109.069200	78.608620
1110.998016	78.411765
1112.926832	78.185395
1114.855648	78.018024
1116.784464	77.936751
1118.713280	78.026023
1120.642096	78.255797
1122.570912	78.605687
1124.499728	78.935731
1126.428544	79.162766
1128.357360	79.313821
1130.286176	79.376189
1132.214992	79.418493
1134.143808	79.396603
1136.072624	79.323653
1138.001440	79.273919
1139.930256	79.206275
1141.859072	79.129335

1143.787888	79.067333
1145.716704	79.055795
1147.645520	79.088865
1149.574336	79.159872
1151.503152	79.255897
1153.431968	79.305232
1155.360784	79.328710
1157.289600	79.293475
1159.218416	79.247398
1161.147232	79.218132
1163.076048	79.149873
1165.004864	79.130006
1166.933680	79.127614
1168.862496	79.091536
1170.791312	79.082659
1172.720128	79.106339
1174.648944	79.057805
1176.577760	78.992003
1178.506576	78.991012
1180.435392	79.018547
1182.364208	79.044183
1184.293024	78.991605
1186.221840	78.954422
1188.150656	78.964212
1190.079472	78.982498
1192.008288	79.017356

1193.937104	79.032250
1195.865920	79.058415
1197.794736	79.090914
1199.723552	79.142809
1201.652368	79.189182
1203.581184	79.213050
1205.510000	79.264793
1207.438816	79.340030
1209.367632	79.395805
1211.296448	79.389753
1213.225264	79.386884
1215.154080	79.404520
1217.082896	79.414208
1219.011712	79.455021
1220.940528	79.503241
1222.869344	79.519681
1224.798160	79.520362
1226.726976	79.590019
1228.655792	79.621098
1230.584608	79.648507
1232.513424	79.727006
1234.442240	79.789923
1236.371056	79.851330
1238.299872	79.879460
1240.228688	79.915030
1242.157504	79.917156

1244.086320	79.924034
1246.015136	79.939096
1247.943952	79.971486
1249.872768	80.010346
1251.801584	80.003061
1253.730400	80.010763
1255.659216	80.025338
1257.588032	80.046635
1259.516848	80.048596
1261.445664	80.054568
1263.374480	80.117919
1265.303296	80.138188
1267.232112	80.101043
1269.160928	80.068961
1271.089744	80.016926
1273.018560	80.009359
1274.947376	80.063594
1276.876192	80.056271
1278.805008	79.985118
1280.733824	79.937110
1282.662640	79.917313
1284.591456	79.876042
1286.520272	79.786000
1288.449088	79.723680
1290.377904	79.707817
1292.306720	79.659950

1294.235536	79.578810
1296.164352	79.452936
1298.093168	79.348104
1300.021984	79.245639
1301.950800	79.134986
1303.879616	79.013490
1305.808432	78.892956
1307.737248	78.760387
1309.666064	78.633445
1311.594880	78.427042
1313.523696	78.204324
1315.452512	78.075157
1317.381328	77.908050
1319.310144	77.776053
1321.238960	77.688503
1323.167776	77.548385
1325.096592	77.335286
1327.025408	77.092068
1328.954224	76.837369
1330.883040	76.565425
1332.811856	76.285093
1334.740672	75.888180
1336.669488	75.357587
1338.598304	74.751855
1340.527120	74.503451
1342.455936	74.612955

1344.384752	74.473900
1346.313568	74.240098
1348.242384	74.025664
1350.171200	73.850275
1352.100016	73.758850
1354.028832	73.624591
1355.957648	73.599457
1357.886464	73.659343
1359.815280	73.617587
1361.744096	73.435400
1363.672912	73.721955
1365.601728	74.376954
1367.530544	74.644675
1369.459360	74.949103
1371.388176	75.345205
1373.316992	75.367736
1375.245808	75.750895
1377.174624	76.284080
1379.103440	76.564210
1381.032256	76.887856
1382.961072	77.232786
1384.889888	77.367069
1386.818704	77.181937
1388.747520	77.456152
1390.676336	77.914166
1392.605152	77.951270

1394.533968	77.785285
1396.462784	78.056191
1398.391600	78.311379
1400.320416	78.770358
1402.249232	79.247897
1404.178048	79.182712
1406.106864	79.415410
1408.035680	79.813897
1409.964496	79.910696
1411.893312	80.061297
1413.822128	80.292411
1415.750944	80.217574
1417.679760	79.849999
1419.608576	80.063053
1421.537392	80.754869
1423.466208	80.817641
1425.395024	81.131728
1427.323840	81.475892
1429.252656	81.436955
1431.181472	81.537906
1433.110288	81.725680
1435.039104	81.480885
1436.967920	81.274022
1438.896736	81.947347
1440.825552	82.235795
1442.754368	82.257325

1444.683184	82.344685
1446.612000	82.145633
1448.540816	82.121994
1450.469632	82.377380
1452.398448	82.351109
1454.327264	82.076695
1456.256080	81.449012
1458.184896	81.567051
1460.113712	82.407943
1462.042528	82.776305
1463.971344	82.655826
1465.900160	82.657677
1467.828976	82.887378
1469.757792	82.792592
1471.686608	82.235323
1473.615424	82.132760
1475.544240	82.327971
1477.473056	82.660525
1479.401872	83.015338
1481.330688	82.942250
1483.259504	83.045704
1485.188320	82.944639
1487.117136	82.580972
1489.045952	82.127290
1490.974768	82.331635
1492.903584	82.985836

1494.832400	82.922523
1496.761216	82.665778
1498.690032	82.763367
1500.618848	82.882278
1502.547664	82.934915
1504.476480	82.710754
1506.405296	81.674622
1508.334112	81.394527
1510.262928	82.018415
1512.191744	82.408415
1514.120560	82.481540
1516.049376	82.050933
1517.978192	82.086312
1519.907008	82.103584
1521.835824	82.072065
1523.764640	82.536187
1525.693456	82.460072
1527.622272	82.618679
1529.551088	83.076889
1531.479904	82.985096
1533.408720	82.660411
1535.337536	83.023559
1537.266352	83.122652
1539.195168	82.681020
1541.123984	82.634245
1543.052800	82.622819

1544.981616	82.637791
1546.910432	83.272852
1548.839248	83.125900
1550.768064	82.937297
1552.696880	83.197813
1554.625696	82.988524
1556.554512	82.924615
1558.483328	82.346317
1560.412144	82.773186
1562.340960	83.565909
1564.269776	83.601067
1566.198592	83.616859
1568.127408	83.501589
1570.056224	83.394964
1571.985040	83.689822
1573.913856	83.711427
1575.842672	83.538094
1577.771488	83.669703
1579.700304	83.831613
1581.629120	83.810517
1583.557936	83.816359
1585.486752	83.825267
1587.415568	83.801268
1589.344384	83.760038
1591.273200	83.722370
1593.202016	83.673192

1595.130832	83.579052
1597.059648	83.513888
1598.988464	83.453590
1600.917280	83.355614
1602.846096	83.333970
1604.774912	83.487805
1606.703728	83.645852
1608.632544	83.838590
1610.561360	84.088525
1612.490176	84.277213
1614.418992	84.309004
1616.347808	84.240104
1618.276624	84.357389
1620.205440	84.453360
1622.134256	84.299880
1624.063072	84.345198
1625.991888	84.489155
1627.920704	84.423689
1629.849520	84.534609
1631.778336	84.583138
1633.707152	84.348206
1635.635968	84.070986
1637.564784	84.274524
1639.493600	84.555156
1641.422416	84.645413
1643.351232	84.616215

1645.280048	84.314714
1647.208864	83.978430
1649.137680	84.344867
1651.066496	84.517579
1652.995312	84.060389
1654.924128	84.337812
1656.852944	84.908586
1658.781760	85.091123
1660.710576	85.029791
1662.639392	84.946150
1664.568208	85.137725
1666.497024	85.156320
1668.425840	84.867303
1670.354656	84.829512
1672.283472	85.116051
1674.212288	85.067643
1676.141104	85.070146
1678.069920	85.293108
1679.998736	85.023418
1681.927552	84.807336
1683.856368	84.494843
1685.785184	84.976690
1687.714000	85.281749
1689.642816	85.247692
1691.571632	85.504162
1693.500448	85.398265

1695.429264	84.883156
1697.358080	84.817644
1699.286896	84.684773
1701.215712	84.578224
1703.144528	85.044026
1705.073344	84.983960
1707.002160	85.127846
1708.930976	85.280663
1710.859792	85.231489
1712.788608	85.041215
1714.717424	84.675708
1716.646240	84.430136
1718.575056	84.588838
1720.503872	85.212751
1722.432688	85.404582
1724.361504	85.417391
1726.290320	85.547422
1728.219136	85.368434
1730.147952	85.122216
1732.076768	85.040148
1734.005584	84.845096
1735.934400	85.405380
1737.863216	85.688435
1739.792032	85.675705
1741.720848	85.732569
1743.649664	85.598989

1745.578480	85.745372
1747.507296	85.727915
1749.436112	85.610525
1751.364928	85.766706
1753.293744	86.427833
1755.222560	86.662032
1757.151376	86.529980
1759.080192	86.811528
1761.009008	86.786470
1762.937824	86.945436
1764.866640	87.373228
1766.795456	87.174181
1768.724272	87.059464
1770.653088	87.226873
1772.581904	87.301190
1774.510720	87.504476
1776.439536	87.644836
1778.368352	87.718624
1780.297168	87.650467
1782.225984	87.804619
1784.154800	87.770973
1786.083616	87.778200
1788.012432	87.977533
1789.941248	87.693350
1791.870064	87.431559
1793.798880	87.679660

1795.727696	87.810487
1797.656512	87.874150
1799.585328	87.832431
1801.514144	87.658199
1803.442960	87.919563
1805.371776	88.167386
1807.300592	88.147615
1809.229408	88.080306
1811.158224	88.024489
1813.087040	88.183806
1815.015856	88.270392
1816.944672	88.181967
1818.873488	88.258216
1820.802304	88.300969
1822.731120	88.217131
1824.659936	87.962721
1826.588752	88.081228
1828.517568	88.130199
1830.446384	88.025403
1832.375200	88.312598
1834.304016	88.412121
1836.232832	88.411942
1838.161648	88.424773
1840.090464	88.479033
1842.019280	88.285615
1843.948096	88.028920

1845.876912	88.171416
1847.805728	88.340786
1849.734544	88.506857
1851.663360	88.625993
1853.592176	88.637987
1855.520992	88.691682
1857.449808	88.695014
1859.378624	88.674139
1861.307440	88.675526
1863.236256	88.709021
1865.165072	88.638477
1867.093888	88.338931
1869.022704	88.197554
1870.951520	88.482227
1872.880336	88.736293
1874.809152	88.776404
1876.737968	88.774031
1878.666784	88.792462
1880.595600	88.824218
1882.524416	88.792517
1884.453232	88.724773
1886.382048	88.816585
1888.310864	88.774086
1890.239680	88.652032
1892.168496	88.824902
1894.097312	88.778423

1896.026128	88.723500
1897.954944	88.902707
1899.883760	88.905418
1901.812576	88.866438
1903.741392	88.885923
1905.670208	88.858873
1907.599024	88.889749
1909.527840	88.980552
1911.456656	89.038842
1913.385472	89.008712
1915.314288	88.949052
1917.243104	88.880330
1919.171920	88.904711
1921.100736	88.926691
1923.029552	88.892918
1924.958368	89.183636
1926.887184	89.268201
1928.816000	89.189223
1930.744816	89.316429
1932.673632	89.297652
1934.602448	89.329707
1936.531264	89.440451
1938.460080	89.522520
1940.388896	89.302857
1942.317712	89.058818
1944.246528	89.454519

1946.175344	89.718946
1948.104160	89.705979
1950.032976	89.676258
1951.961792	89.725798
1953.890608	89.731393
1955.819424	89.754956
1957.748240	90.137561
1959.677056	90.083335
1961.605872	89.896686
1963.534688	90.088494
1965.463504	90.043628
1967.392320	89.990837
1969.321136	90.184193
1971.249952	90.268322
1973.178768	90.338297
1975.107584	90.427964
1977.036400	90.203552
1978.965216	89.961075
1980.894032	90.020203
1982.822848	89.957356
1984.751664	90.020388
1986.680480	90.172809
1988.609296	90.024011
1990.538112	89.832752
1992.466928	89.764021
1994.395744	89.967748

1996.324560	90.092886
1998.253376	89.986257
2000.182192	89.906820
2002.111008	90.069989
2004.039824	90.063890
2005.968640	90.008362
2007.897456	90.059254
2009.826272	90.118118
2011.755088	90.125233
2013.683904	90.111401
2015.612720	90.245477
2017.541536	90.068574
2019.470352	89.741500
2021.399168	89.969410
2023.327984	90.282381
2025.256800	90.216246
2027.185616	90.372393
2029.114432	90.476232
2031.043248	90.244498
2032.972064	90.082927
2034.900880	90.050202
2036.829696	90.175281
2038.758512	90.138229
2040.687328	89.875201
2042.616144	90.119324
2044.544960	90.484860

2046.473776	90.413007
2048.402592	90.149964
2050.331408	90.119156
2052.260224	90.183182
2054.189040	90.090134
2056.117856	89.977737
2058.046672	90.023637
2059.975488	90.087046
2061.904304	90.020645
2063.833120	90.119074
2065.761936	90.128707
2067.690752	90.016469
2069.619568	90.037007
2071.548384	90.141542
2073.477200	90.277309
2075.406016	90.312219
2077.334832	90.238669
2079.263648	90.243088
2081.192464	90.229866
2083.121280	90.271591
2085.050096	90.321939
2086.978912	90.208733
2088.907728	90.125487
2090.836544	90.082906
2092.765360	90.120133
2094.694176	90.172242

2096.622992	90.187201
2098.551808	90.279946
2100.480624	90.337806
2102.409440	90.396258
2104.338256	90.471089
2106.267072	90.394406
2108.195888	90.382433
2110.124704	90.356055
2112.053520	90.381028
2113.982336	90.373278
2115.911152	90.299769
2117.839968	90.518804
2119.768784	90.581225
2121.697600	90.536093
2123.626416	90.645643
2125.555232	90.694314
2127.484048	90.727957
2129.412864	90.619895
2131.341680	90.641849
2133.270496	90.840337
2135.199312	90.837463
2137.128128	90.860960
2139.056944	91.066938
2140.985760	91.401985
2142.914576	91.604619
2144.843392	91.625035

2146.772208	91.463800
2148.701024	91.611483
2150.629840	91.886535
2152.558656	91.784901
2154.487472	91.918540
2156.416288	92.191268
2158.345104	92.482733
2160.273920	92.529862
2162.202736	92.247641
2164.131552	92.129117
2166.060368	92.194758
2167.989184	91.988912
2169.918000	91.824847
2171.846816	91.738945
2173.775632	91.603056
2175.704448	91.421827
2177.633264	91.489994
2179.562080	91.827387
2181.490896	91.667662
2183.419712	91.416190
2185.348528	91.536710
2187.277344	91.589531
2189.206160	91.577141
2191.134976	91.659918
2193.063792	91.607975
2194.992608	91.306963

2196.921424	91.208109
2198.850240	91.389612
2200.779056	91.509366
2202.707872	91.370624
2204.636688	91.265295
2206.565504	91.298729
2208.494320	91.212578
2210.423136	91.227824
2212.351952	91.147703
2214.280768	91.219362
2216.209584	91.375590
2218.138400	91.111470
2220.067216	91.087871
2221.996032	91.191408
2223.924848	91.152233
2225.853664	91.096591
2227.782480	91.019414
2229.711296	91.010466
2231.640112	90.981291
2233.568928	91.028439
2235.497744	91.119434
2237.426560	91.078418
2239.355376	91.161418
2241.284192	91.235201
2243.213008	91.220206
2245.141824	91.221167

2247.070640	91.179963
2248.999456	91.203893
2250.928272	91.181338
2252.857088	91.181721
2254.785904	91.099195
2256.714720	91.108017
2258.643536	91.230214
2260.572352	91.341195
2262.501168	91.301281
2264.429984	91.258986
2266.358800	91.310767
2268.287616	91.302244
2270.216432	91.332639
2272.145248	91.334010
2274.074064	91.291590
2276.002880	91.229529
2277.931696	91.197222
2279.860512	91.225241
2281.789328	91.253192
2283.718144	91.266426
2285.646960	91.258084
2287.575776	91.190914
2289.504592	91.129490
2291.433408	91.031893
2293.362224	90.915868
2295.291040	90.798588

2297.219856	90.709380
2299.148672	90.695435
2301.077488	90.544855
2303.006304	90.377598
2304.935120	90.298462
2306.863936	90.192276
2308.792752	90.050383
2310.721568	90.003316
2312.650384	90.082757
2314.579200	90.229330
2316.508016	90.437474
2318.436832	90.552025
2320.365648	90.604048
2322.294464	90.793114
2324.223280	91.164015
2326.152096	91.530329
2328.080912	91.805094
2330.009728	92.071251
2331.938544	92.418620
2333.867360	92.894541
2335.796176	93.198965
2337.724992	93.324853
2339.653808	93.389766
2341.582624	93.358363
2343.511440	93.011863
2345.440256	92.504979

2347.369072	91.766363
2349.297888	91.244752
2351.226704	91.654210
2353.155520	92.494677
2355.084336	92.872925
2357.013152	93.287653
2358.941968	93.773455
2360.870784	93.805176
2362.799600	93.721747
2364.728416	93.628286
2366.657232	93.216609
2368.586048	92.736239
2370.514864	92.267067
2372.443680	91.822940
2374.372496	91.435455
2376.301312	91.038199
2378.230128	90.819270
2380.158944	90.789022
2382.087760	90.927052
2384.016576	91.191317
2385.945392	91.457697
2387.874208	91.648956
2389.803024	91.719405
2391.731840	91.773956
2393.660656	91.810631
2395.589472	91.792663

2397.518288	91.770621
2399.447104	91.780354
2401.375920	91.795522
2403.304736	91.817297
2405.233552	91.859024
2407.162368	91.860273
2409.091184	91.879553
2411.020000	91.910647
2412.948816	91.931691
2414.877632	91.966516
2416.806448	91.971712
2418.735264	91.992065
2420.664080	92.003048
2422.592896	92.026207
2424.521712	92.042082
2426.450528	92.065590
2428.379344	92.098439
2430.308160	92.090713
2432.236976	92.126830
2434.165792	92.133525
2436.094608	92.132290
2438.023424	92.186089
2439.952240	92.184843
2441.881056	92.191440
2443.809872	92.240601
2445.738688	92.303332

2447.667504	92.308269
2449.596320	92.309061
2451.525136	92.339867
2453.453952	92.325924
2455.382768	92.290706
2457.311584	92.322292
2459.240400	92.381513
2461.169216	92.375057
2463.098032	92.371865
2465.026848	92.392366
2466.955664	92.428637
2468.884480	92.423757
2470.813296	92.441514
2472.742112	92.454999
2474.670928	92.382613
2476.599744	92.388497
2478.528560	92.448250
2480.457376	92.409809
2482.386192	92.350500
2484.315008	92.388950
2486.243824	92.468266
2488.172640	92.454042
2490.101456	92.438328
2492.030272	92.448273
2493.959088	92.454084
2495.887904	92.479281

2497.816720	92.467335
2499.745536	92.441349
2501.674352	92.412152
2503.603168	92.392563
2505.531984	92.449761
2507.460800	92.463352
2509.389616	92.484590
2511.318432	92.540589
2513.247248	92.519729
2515.176064	92.558948
2517.104880	92.606763
2519.033696	92.558645
2520.962512	92.556223
2522.891328	92.587644
2524.820144	92.581390
2526.748960	92.570835
2528.677776	92.566937
2530.606592	92.566957
2532.535408	92.568955
2534.464224	92.671362
2536.393040	92.693233
2538.321856	92.607498
2540.250672	92.633765
2542.179488	92.679888
2544.108304	92.661147
2546.037120	92.637691

2547.965936	92.646575
2549.894752	92.667465
2551.823568	92.693672
2553.752384	92.696706
2555.681200	92.706159
2557.610016	92.685153
2559.538832	92.676460
2561.467648	92.705824
2563.396464	92.755620
2565.325280	92.810697
2567.254096	92.773497
2569.182912	92.741407
2571.111728	92.748356
2573.040544	92.775702
2574.969360	92.775604
2576.898176	92.757357
2578.826992	92.771715
2580.755808	92.763151
2582.684624	92.750453
2584.613440	92.746745
2586.542256	92.733841
2588.471072	92.761832
2590.399888	92.782969
2592.328704	92.770280
2594.257520	92.787021
2596.186336	92.753346

2598.115152	92.720017
2600.043968	92.765252
2601.972784	92.785271
2603.901600	92.771273
2605.830416	92.750850
2607.759232	92.738429
2609.688048	92.756838
2611.616864	92.771987
2613.545680	92.783348
2615.474496	92.776127
2617.403312	92.779871
2619.332128	92.797344
2621.260944	92.765908
2623.189760	92.742559
2625.118576	92.770276
2627.047392	92.779289
2628.976208	92.782195
2630.905024	92.762728
2632.833840	92.737607
2634.762656	92.758352
2636.691472	92.779036
2638.620288	92.783433
2640.549104	92.789049
2642.477920	92.819924
2644.406736	92.821291
2646.335552	92.803913

2648.264368	92.796685
2650.193184	92.788659
2652.122000	92.822538
2654.050816	92.865786
2655.979632	92.855553
2657.908448	92.823479
2659.837264	92.796773
2661.766080	92.828199
2663.694896	92.887652
2665.623712	92.903706
2667.552528	92.890354
2669.481344	92.891816
2671.410160	92.920134
2673.338976	92.919280
2675.267792	92.901421
2677.196608	92.909396
2679.125424	92.936426
2681.054240	92.972741
2682.983056	92.982060
2684.911872	92.977124
2686.840688	92.984711
2688.769504	93.001027
2690.698320	93.043713
2692.627136	93.085595
2694.555952	93.067679
2696.484768	93.058567

2698.413584	93.086142
2700.342400	93.089341
2702.271216	93.103239
2704.200032	93.133876
2706.128848	93.151085
2708.057664	93.143708
2709.986480	93.129915
2711.915296	93.132606
2713.844112	93.164277
2715.772928	93.180442
2717.701744	93.183763
2719.630560	93.204173
2721.559376	93.198643
2723.488192	93.179110
2725.417008	93.181208
2727.345824	93.202528
2729.274640	93.192995
2731.203456	93.192526
2733.132272	93.210975
2735.061088	93.205742
2736.989904	93.206691
2738.918720	93.204845
2740.847536	93.223332
2742.776352	93.215628
2744.705168	93.198766
2746.633984	93.222806

2748.562800	93.212127
2750.491616	93.193005
2752.420432	93.224266
2754.349248	93.253764
2756.278064	93.249953
2758.206880	93.227410
2760.135696	93.217389
2762.064512	93.258754
2763.993328	93.255957
2765.922144	93.213933
2767.850960	93.220029
2769.779776	93.235097
2771.708592	93.232980
2773.637408	93.232504
2775.566224	93.239275
2777.495040	93.247788
2779.423856	93.242427
2781.352672	93.222278
2783.281488	93.210803
2785.210304	93.191231
2787.139120	93.203484
2789.067936	93.218290
2790.996752	93.212591
2792.925568	93.211414
2794.854384	93.227429
2796.783200	93.259589

2798.712016	93.246768
2800.640832	93.188408
2802.569648	93.178473
2804.498464	93.227868
2806.427280	93.217388
2808.356096	93.197697
2810.284912	93.217280
2812.213728	93.212450
2814.142544	93.221761
2816.071360	93.221546
2818.000176	93.199851
2819.928992	93.193340
2821.857808	93.213915
2823.786624	93.237439
2825.715440	93.219036
2827.644256	93.212051
2829.573072	93.208808
2831.501888	93.210121
2833.430704	93.213391
2835.359520	93.192204
2837.288336	93.187950
2839.217152	93.195487
2841.145968	93.199689
2843.074784	93.187848
2845.003600	93.138845
2846.932416	93.100727

2848.861232	93.071512
2850.790048	93.042538
2852.718864	93.028509
2854.647680	93.029806
2856.576496	93.056648
2858.505312	93.101910
2860.434128	93.153500
2862.362944	93.177133
2864.291760	93.165755
2866.220576	93.152662
2868.149392	93.183650
2870.078208	93.190120
2872.007024	93.115806
2873.935840	93.090847
2875.864656	93.145172
2877.793472	93.146613
2879.722288	93.092157
2881.651104	93.094217
2883.579920	93.101828
2885.508736	93.102208
2887.437552	93.113820
2889.366368	93.091251
2891.295184	93.067608
2893.224000	93.088560
2895.152816	93.072205
2897.081632	93.047126

2899.010448	93.075460
2900.939264	93.051207
2902.868080	93.005854
2904.796896	93.004209
2906.725712	93.013058
2908.654528	93.010510
2910.583344	92.994257
2912.512160	92.963042
2914.440976	92.945329
2916.369792	92.935369
2918.298608	92.924144
2920.227424	92.908035
2922.156240	92.859740
2924.085056	92.833394
2926.013872	92.875452
2927.942688	92.915475
2929.871504	92.947273
2931.800320	92.961465
2933.729136	92.975430
2935.657952	93.005062
2937.586768	93.024015
2939.515584	93.056694
2941.444400	93.088223
2943.373216	93.118968
2945.302032	93.120028
2947.230848	93.112604

2949.159664	93.153274
2951.088480	93.150100
2953.017296	93.117707
2954.946112	93.136679
2956.874928	93.155747
2958.803744	93.149908
2960.732560	93.114680
2962.661376	93.092985
2964.590192	93.106594
2966.519008	93.129341
2968.447824	93.190308
2970.376640	93.233173
2972.305456	93.216919
2974.234272	93.206992
2976.163088	93.199362
2978.091904	93.198462
2980.020720	93.213818
2981.949536	93.218624
2983.878352	93.245536
2985.807168	93.244881
2987.735984	93.209723
2989.664800	93.223239
2991.593616	93.268197
2993.522432	93.257047
2995.451248	93.262997
2997.380064	93.281175

2999.308880	93.273214
3001.237696	93.300509
3003.166512	93.308160
3005.095328	93.325307
3007.024144	93.343464
3008.952960	93.303297
3010.881776	93.302146
3012.810592	93.317695
3014.739408	93.331699
3016.668224	93.405267
3018.597040	93.465099
3020.525856	93.456698
3022.454672	93.437189
3024.383488	93.431196
3026.312304	93.434284
3028.241120	93.429003
3030.169936	93.406104
3032.098752	93.429453
3034.027568	93.445768
3035.956384	93.445247
3037.885200	93.439770
3039.814016	93.440336
3041.742832	93.486683
3043.671648	93.503782
3045.600464	93.471376
3047.529280	93.459898

3049.458096	93.456645
3051.386912	93.446582
3053.315728	93.479954
3055.244544	93.497211
3057.173360	93.487102
3059.102176	93.506563
3061.030992	93.531947
3062.959808	93.539113
3064.888624	93.514883
3066.817440	93.500555
3068.746256	93.515130
3070.675072	93.551881
3072.603888	93.539727
3074.532704	93.543658
3076.461520	93.570812
3078.390336	93.555613
3080.319152	93.580879
3082.247968	93.596290
3084.176784	93.622928
3086.105600	93.639772
3088.034416	93.597355
3089.963232	93.590490
3091.892048	93.631417
3093.820864	93.623395
3095.749680	93.599724
3097.678496	93.575279

3099.607312	93.562565
3101.536128	93.580446
3103.464944	93.613052
3105.393760	93.652126
3107.322576	93.656520
3109.251392	93.638554
3111.180208	93.597848
3113.109024	93.618957
3115.037840	93.659770
3116.966656	93.644043
3118.895472	93.623890
3120.824288	93.612558
3122.753104	93.621457
3124.681920	93.672639
3126.610736	93.683438
3128.539552	93.656678
3130.468368	93.687054
3132.397184	93.701585
3134.326000	93.706667
3136.254816	93.714778
3138.183632	93.679733
3140.112448	93.703206
3142.041264	93.725152
3143.970080	93.734735
3145.898896	93.724031
3147.827712	93.691405

3149.756528	93.691030
3151.685344	93.734536
3153.614160	93.785645
3155.542976	93.807407
3157.471792	93.825562
3159.400608	93.816119
3161.329424	93.809278
3163.258240	93.788051
3165.187056	93.768802
3167.115872	93.764333
3169.044688	93.788021
3170.973504	93.803983
3172.902320	93.769063
3174.831136	93.779510
3176.759952	93.812340
3178.688768	93.767338
3180.617584	93.791965
3182.546400	93.813127
3184.475216	93.794081
3186.404032	93.848587
3188.332848	93.873297
3190.261664	93.848196
3192.190480	93.840953
3194.119296	93.867293
3196.048112	93.848035
3197.976928	93.820251

3199.905744	93.850675
3201.834560	93.879532
3203.763376	93.844755
3205.692192	93.859009
3207.621008	93.900524
3209.549824	93.884163
3211.478640	93.865868
3213.407456	93.866359
3215.336272	93.849721
3217.265088	93.841412
3219.193904	93.837157
3221.122720	93.809619
3223.051536	93.810905
3224.980352	93.817343
3226.909168	93.824767
3228.837984	93.861252
3230.766800	93.892178
3232.695616	93.907224
3234.624432	93.900722
3236.553248	93.882739
3238.482064	93.905891
3240.410880	93.892203
3242.339696	93.906966
3244.268512	93.931077
3246.197328	93.937023
3248.126144	93.954816

3250.054960	93.926629
3251.983776	93.955650
3253.912592	93.937447
3255.841408	93.865609
3257.770224	93.906651
3259.699040	93.949048
3261.627856	93.923992
3263.556672	93.937346
3265.485488	93.917993
3267.414304	93.926857
3269.343120	93.948128
3271.271936	93.944875
3273.200752	93.955152
3275.129568	93.928211
3277.058384	93.937186
3278.987200	93.986670
3280.916016	94.001435
3282.844832	93.970431
3284.773648	93.928968
3286.702464	93.938264
3288.631280	93.971487
3290.560096	93.959783
3292.488912	93.943616
3294.417728	93.927506
3296.346544	93.931245
3298.275360	93.977942

3300.204176	93.985278
3302.132992	93.983355
3304.061808	93.956738
3305.990624	93.947948
3307.919440	93.969014
3309.848256	93.973675
3311.777072	94.002330
3313.705888	93.970434
3315.634704	93.928131
3317.563520	93.927133
3319.492336	93.929725
3321.421152	93.926619
3323.349968	93.914724
3325.278784	93.886377
3327.207600	93.906454
3329.136416	93.964141
3331.065232	93.953913
3332.994048	93.899219
3334.922864	93.882646
3336.851680	93.891652
3338.780496	93.904751
3340.709312	93.892139
3342.638128	93.870786
3344.566944	93.874221
3346.495760	93.867456
3348.424576	93.843918

3350.353392	93.855633
3352.282208	93.855265
3354.211024	93.840523
3356.139840	93.824952
3358.068656	93.817584
3359.997472	93.808762
3361.926288	93.803697
3363.855104	93.809579
3365.783920	93.777469
3367.712736	93.818982
3369.641552	93.895880
3371.570368	93.905288
3373.499184	93.898810
3375.428000	93.912772
3377.356816	93.899849
3379.285632	93.858859
3381.214448	93.850695
3383.143264	93.868755
3385.072080	93.877700
3387.000896	93.904871
3388.929712	93.910013
3390.858528	93.914814
3392.787344	93.896550
3394.716160	93.898262
3396.644976	93.937739
3398.573792	94.008847

3400.502608	94.096354
3402.431424	94.099409
3404.360240	94.100803
3406.289056	94.149469
3408.217872	94.126478
3410.146688	94.138885
3412.075504	94.215799
3414.004320	94.211090
3415.933136	94.220489
3417.861952	94.270887
3419.790768	94.258645
3421.719584	94.271654
3423.648400	94.376467
3425.577216	94.434531
3427.506032	94.501445
3429.434848	94.568584
3431.363664	94.621101
3433.292480	94.668047
3435.221296	94.694126
3437.150112	94.738385
3439.078928	94.795213
3441.007744	94.837725
3442.936560	94.867727
3444.865376	94.889349
3446.794192	94.830660
3448.723008	94.877401

3450.651824	95.054419
3452.580640	95.201711
3454.509456	95.274128
3456.438272	95.269017
3458.367088	95.256456
3460.295904	95.309718
3462.224720	95.373198
3464.153536	95.438879
3466.082352	95.556897
3468.011168	95.639704
3469.939984	95.697509
3471.868800	95.777477
3473.797616	95.849843
3475.726432	95.908283
3477.655248	95.995487
3479.584064	96.030411
3481.512880	95.997415
3483.441696	96.077494
3485.370512	96.239921
3487.299328	96.302932
3489.228144	96.398338
3491.156960	96.566507
3493.085776	96.626458
3495.014592	96.644021
3496.943408	96.696683
3498.872224	96.827058

3500.801040	96.812059
3502.729856	96.711136
3504.658672	96.886014
3506.587488	97.130629
3508.516304	97.141223
3510.445120	97.174396
3512.373936	97.435634
3514.302752	97.572086
3516.231568	97.548594
3518.160384	97.590736
3520.089200	97.698728
3522.018016	97.644388
3523.946832	97.494733
3525.875648	97.615798
3527.804464	97.746575
3529.733280	97.893484
3531.662096	98.074232
3533.590912	98.217064
3535.519728	98.231035
3537.448544	98.277883
3539.377360	98.478159
3541.306176	98.535313
3543.234992	98.425531
3545.163808	98.129518
3547.092624	98.106077
3549.021440	98.575652

3550.950256	98.835118
3552.879072	98.740341
3554.807888	98.893139
3556.736704	99.118304
3558.665520	99.072691
3560.594336	98.996327
3562.523152	99.013024
3564.451968	98.827017
3566.380784	98.244562
3568.309600	98.223772
3570.238416	99.112511
3572.167232	99.604123
3574.096048	99.629980
3576.024864	99.749497
3577.953680	99.829291
3579.882496	99.779121
3581.811312	99.804979
3583.740128	99.825441
3585.668944	99.550880
3587.597760	99.069322
3589.526576	99.457654
3591.455392	100.147424
3593.384208	100.067025
3595.313024	99.986221
3597.241840	100.296831
3599.170656	100.564693

3601.099472	100.645871
3603.028288	100.800167
3604.957104	100.885708
3606.885920	100.654486
3608.814736	100.415719
3610.743552	100.638666
3612.672368	100.668490
3614.601184	100.658543
3616.530000	101.003744
3618.458816	100.985391
3620.387632	100.964416
3622.316448	101.457519
3624.245264	101.767994
3626.174080	101.769585
3628.102896	101.572755
3630.031712	101.129830
3631.960528	101.865905
3633.889344	102.301729
3635.818160	102.424620
3637.746976	102.692473
3639.675792	102.733235
3641.604608	102.694930
3643.533424	102.704080
3645.462240	102.688781
3647.391056	102.164029
3649.319872	102.004841

3651.248688	102.141409
3653.177504	102.902909
3655.106320	103.446346
3657.035136	103.318939
3658.963952	103.676992
3660.892768	103.927651
3662.821584	103.966841
3664.750400	104.073525
3666.679216	104.012618
3668.608032	103.915559
3670.536848	103.529040
3672.465664	103.627012
3674.394480	104.032748
3676.323296	103.560308
3678.252112	103.983931
3680.180928	104.444990
3682.109744	104.688717
3684.038560	104.779896
3685.967376	104.701648
3687.896192	104.778336
3689.825008	104.448997
3691.753824	104.592947
3693.682640	104.972282
3695.611456	105.178281
3697.540272	105.081240
3699.469088	105.203936

3701.397904	105.184311
3703.326720	105.144557
3705.255536	105.349619
3707.184352	105.358166
3709.113168	105.199115
3711.041984	105.067279
3712.970800	104.845688
3714.899616	105.032901
3716.828432	105.402635
3718.757248	105.317116
3720.686064	105.273323
3722.614880	105.097410
3724.543696	104.970714
3726.472512	104.989474
3728.401328	105.096876
3730.330144	105.474364
3732.258960	105.165836
3734.187776	104.335065
3736.116592	103.887786
3738.045408	104.510116
3739.974224	104.845224
3741.903040	105.022688
3743.831856	105.172125
3745.760672	104.118983
3747.689488	104.801625
3749.618304	105.144285

3751.547120	104.810972
3753.475936	104.876443
3755.404752	105.208034
3757.333568	105.393870
3759.262384	105.382899
3761.191200	105.495101
3763.120016	105.682451
3765.048832	105.582977
3766.977648	105.410240
3768.906464	105.722864
3770.835280	105.451043
3772.764096	105.622961
3774.692912	105.880141
3776.621728	105.859204
3778.550544	105.894661
3780.479360	105.631692
3782.408176	105.785366
3784.336992	105.890239
3786.265808	105.793300
3788.194624	105.976664
3790.123440	106.015993
3792.052256	105.953265
3793.981072	106.010191
3795.909888	105.905531
3797.838704	105.523234
3799.767520	105.966905

3801.696336	105.769421
3803.625152	105.318083
3805.553968	106.062656
3807.482784	105.847587
3809.411600	105.792328
3811.340416	106.051576
3813.269232	106.029742
3815.198048	106.267718
3817.126864	105.553513
3819.055680	105.671270
3820.984496	105.762620
3822.913312	105.365019
3824.842128	105.984633
3826.770944	105.932249
3828.699760	105.849439
3830.628576	106.083229
3832.557392	105.887477
3834.486208	105.792238
3836.415024	105.882464
3838.343840	105.586882
3840.272656	105.256557
3842.201472	105.613725
3844.130288	105.829770
3846.059104	106.004534
3847.987920	106.118285
3849.916736	105.876188

3851.845552	105.964618
3853.774368	105.566498
3855.703184	105.251785
3857.632000	105.678001
3859.560816	106.078657
3861.489632	105.985084
3863.418448	105.841968
3865.347264	105.593029
3867.276080	105.743079
3869.204896	105.922476
3871.133712	105.345398
3873.062528	105.836518
3874.991344	105.837894
3876.920160	105.994584
3878.848976	106.197115
3880.777792	105.478499
3882.706608	105.627191
3884.635424	106.020076
3886.564240	105.665400
3888.493056	106.097164
3890.421872	106.313446
3892.350688	105.802550
3894.279504	106.068324
3896.208320	106.284784
3898.137136	106.024890
3900.065952	105.484931

3901.994768	105.385696
3903.923584	105.425275
3905.852400	105.658521
3907.781216	106.298332
3909.710032	106.685011
3911.638848	106.686764
3913.567664	106.676190
3915.496480	106.518235
3917.425296	106.136257
3919.354112	106.218897
3921.282928	106.430398
3923.211744	106.480670
3925.140560	106.324615
3927.069376	106.463249
3928.998192	106.627838
3930.927008	106.349928
3932.855824	106.199806
3934.784640	106.585871
3936.713456	106.901990
3938.642272	106.814721
3940.571088	106.824934
3942.499904	106.557912
3944.428720	106.507314
3946.357536	106.713401
3948.286352	106.557431
3950.215168	106.591838

3952.143984	106.870821
3954.072800	107.028657
3956.001616	107.045714
3957.930432	107.069731
3959.859248	107.092956
3961.788064	107.027418
3963.716880	107.074509
3965.645696	107.182041
3967.574512	107.161722
3969.503328	107.168692
3971.432144	107.243070
3973.360960	107.259406
3975.289776	107.192803
3977.218592	107.178759
3979.147408	107.278359
3981.076224	107.272283
3983.005040	107.286224
3984.933856	107.365172
3986.862672	107.392003
3988.791488	107.371789
3990.720304	107.270095
3992.649120	107.284599
3994.577936	107.379325
3996.506752	107.402192
3998.435568	107.429811
4000.364384	107.450370

جـامـعـة الـجـوف
وكالة الجامعة للدراسات العليا والبحث العلمي
المختبرات المركزية

SHIMADZU

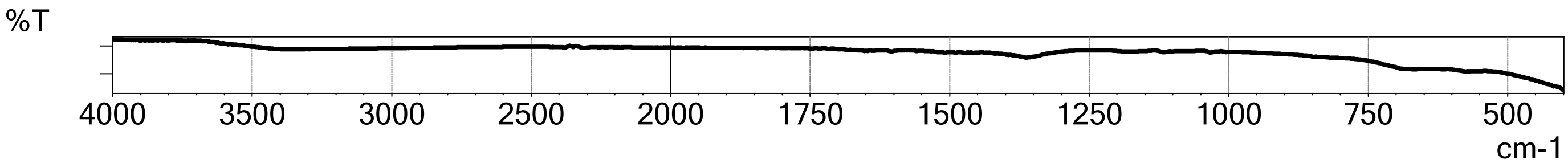

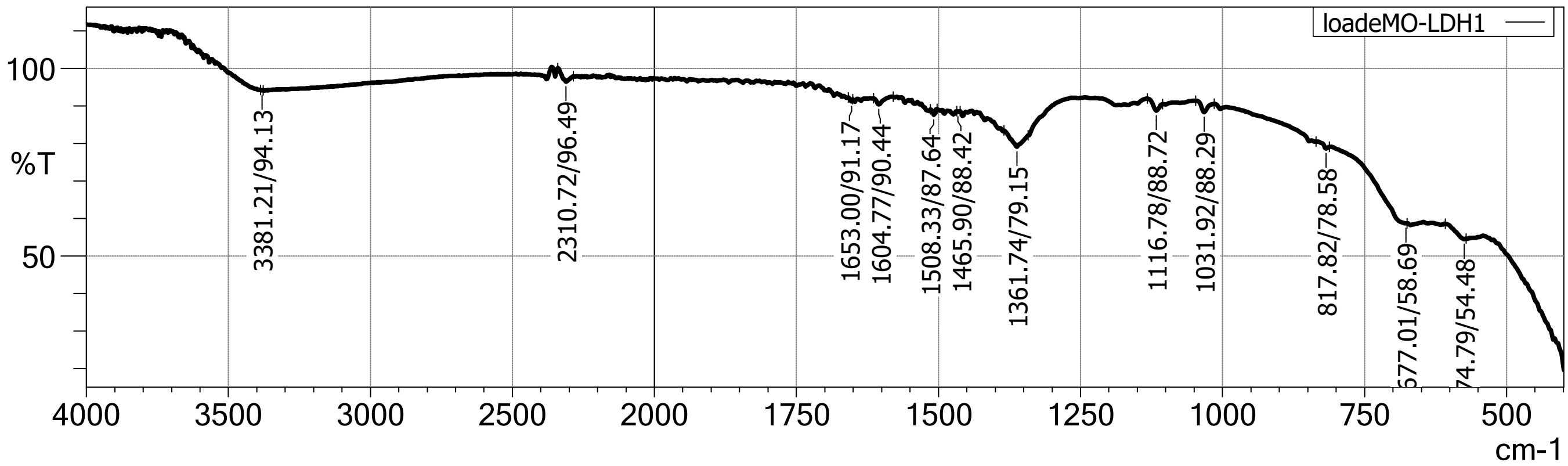

	Peak	Intensity	Corr. Intensity	Base (H)	Base (L)	Area	Corr. Area	Comment
1	574.79	54.48	0.59	607.58	570.93	1596.903	7.291	
2	677.01	58.69	0.30	812.03	675.09	4041.158	-217.321	
3	817.82	78.58	0.89	835.18	812.03	470.045	3.200	
4	1031.92	88.29	2.73	1047.35	1014.56	327.969	32.794	
5	1116.78	88.72	2.46	1132.21	1105.21	262.846	27.393	
6	1361.74	79.15	3.64	1384.89	1342.46	805.458	77.441	
7	1465.90	88.42	0.22	1467.83	1462.04	66.449	0.737	
8	1508.33	87.64	1.51	1514.12	1502.55	132.586	7.021	
9	1604.77	90.44	1.74	1614.42	1579.70	290.377	22.419	
10	1653.00	91.17	0.73	1658.78	1651.07	64.321	2.836	
11	2310.72	96.49	2.44	2339.65	2285.65	124.137	70.754	
12	3381.21	94.13	0.09	3387.00	3377.36	56.094	0.504	

SHIMADZU

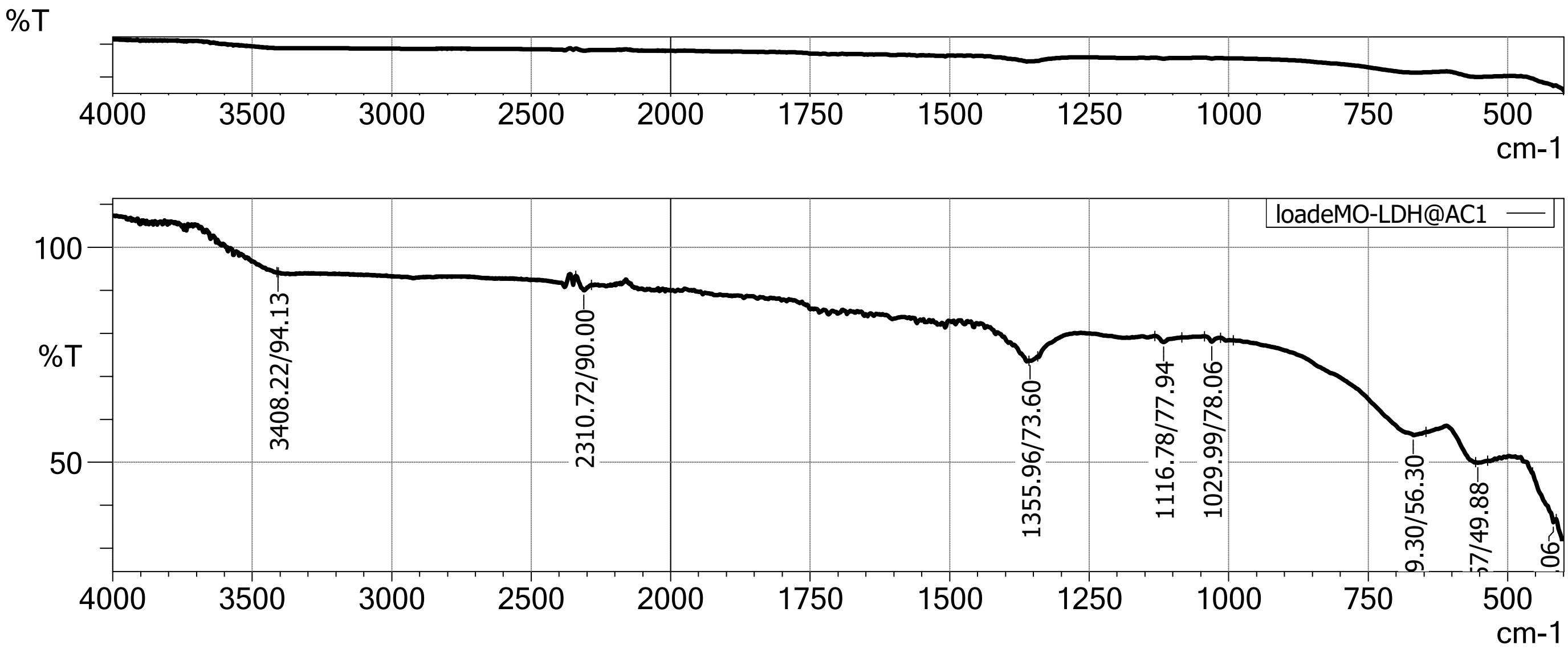

	Peak	Intensity	Corr. Intensity	Base (H)	Base (L)	Area	Corr. Area	Comment
1	418.55	36.06	2.21	455.20	412.77	2501.334	49.005	
2	553.57	49.88	0.22	557.43	536.21	1059.108	2.694	
3	669.30	56.30	2.19	991.41	646.15	10618.328	-518.421	
4	1029.99	78.06	1.10	1043.49	1014.56	613.931	10.923	
5	1116.78	77.94	1.37	1132.21	1083.99	1025.495	24.358	
6	1355.96	73.60	0.18	1357.89	1342.46	401.755	2.662	
7	2310.72	90.00	2.29	2339.65	2283.72	496.417	67.284	
8	3408.22	94.13	0.05	3412.08	3406.29	33.855	0.193	

LDH

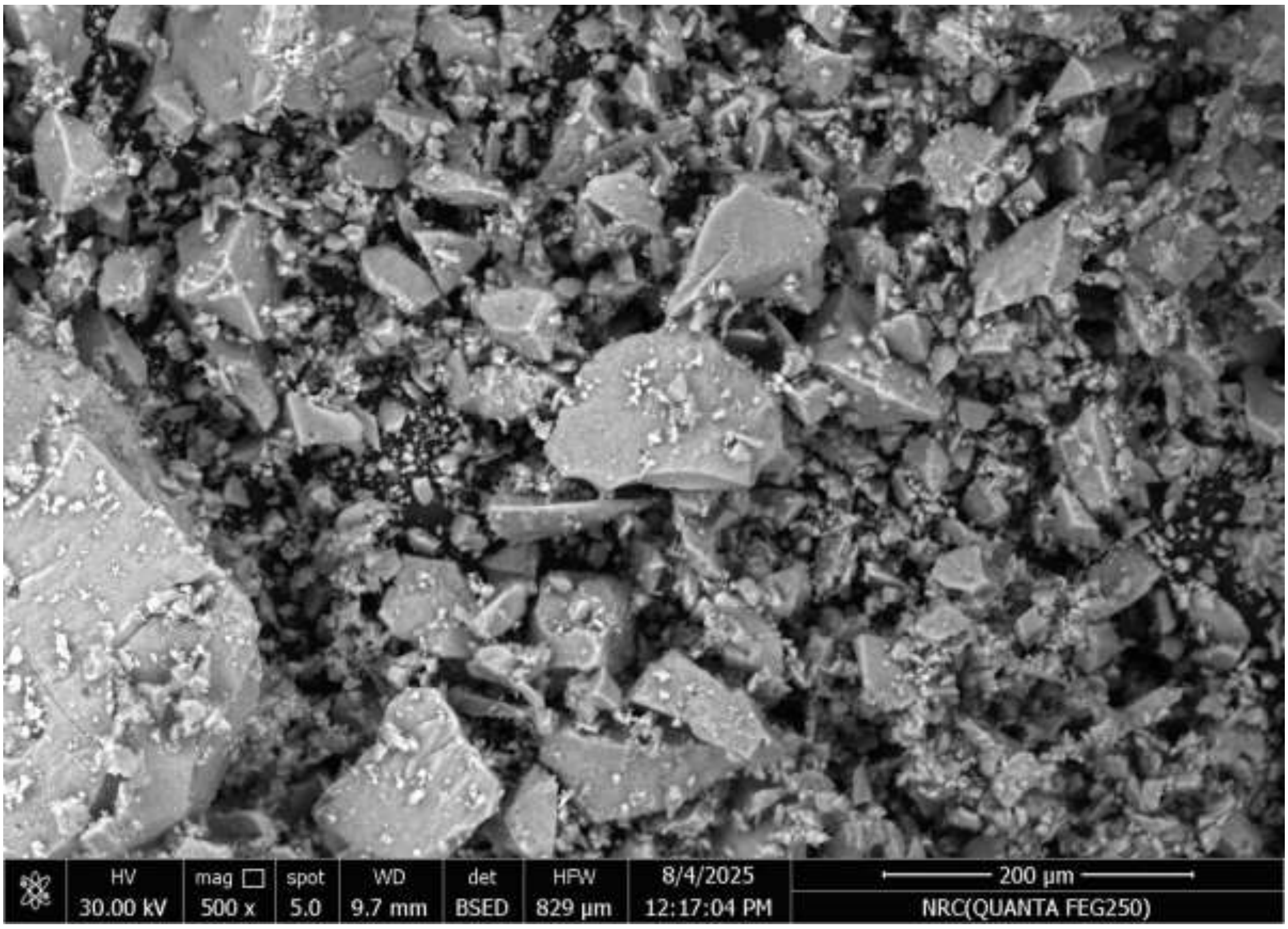
HV
30.00 kV
mag
500 x
spot
5.0
WD
9.7 mm
det
BSED
HFW
829 µm
8/4/2025
12:17:04 PM
200 µm
NRC(QUANTA FEG250)

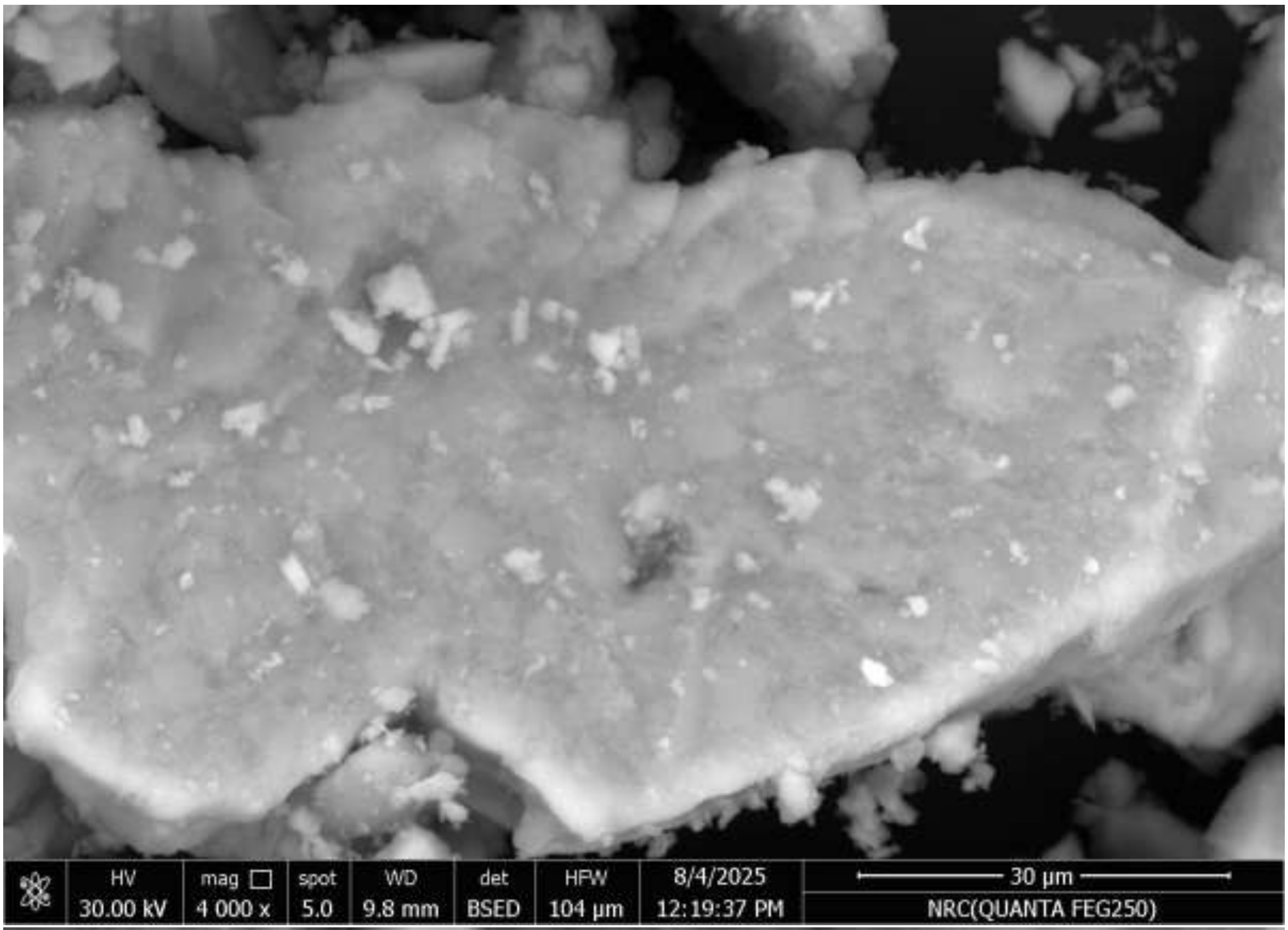
HV
30.00 kV
mag
4 000 x
spot
5.0
WD
9.8 mm
det
BSED
HFW
104 µm
8/4/2025
12:19:37 PM
30 µm
NRC(QUANTA FEG250)

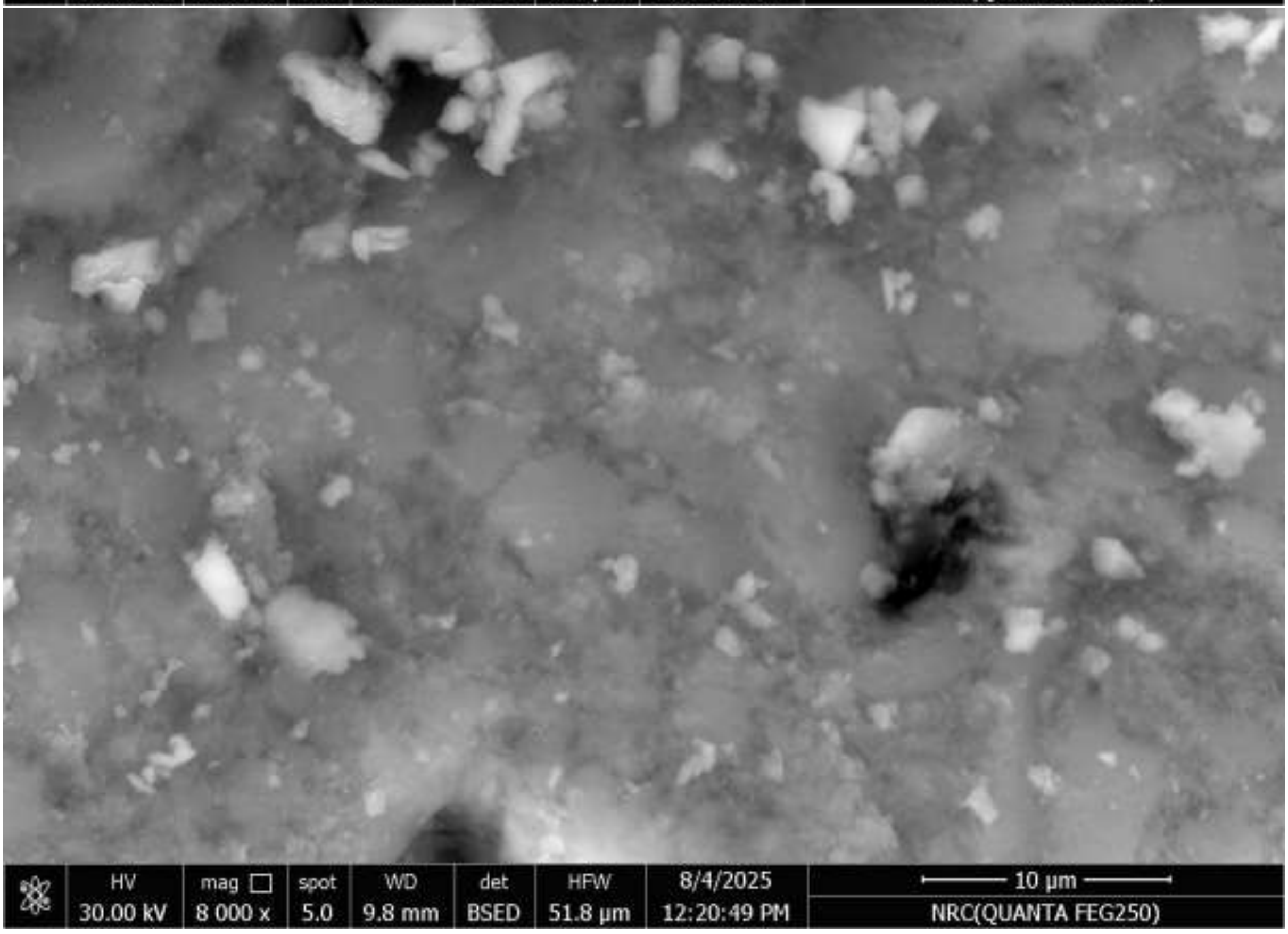
HV
30.00 kV
mag
8 000 x
spot
5.0
WD
9.8 mm
det
BSED
HFW
51.8 µm
8/4/2025
12:20:49 PM
10 µm
NRC(QUANTA FEG250)

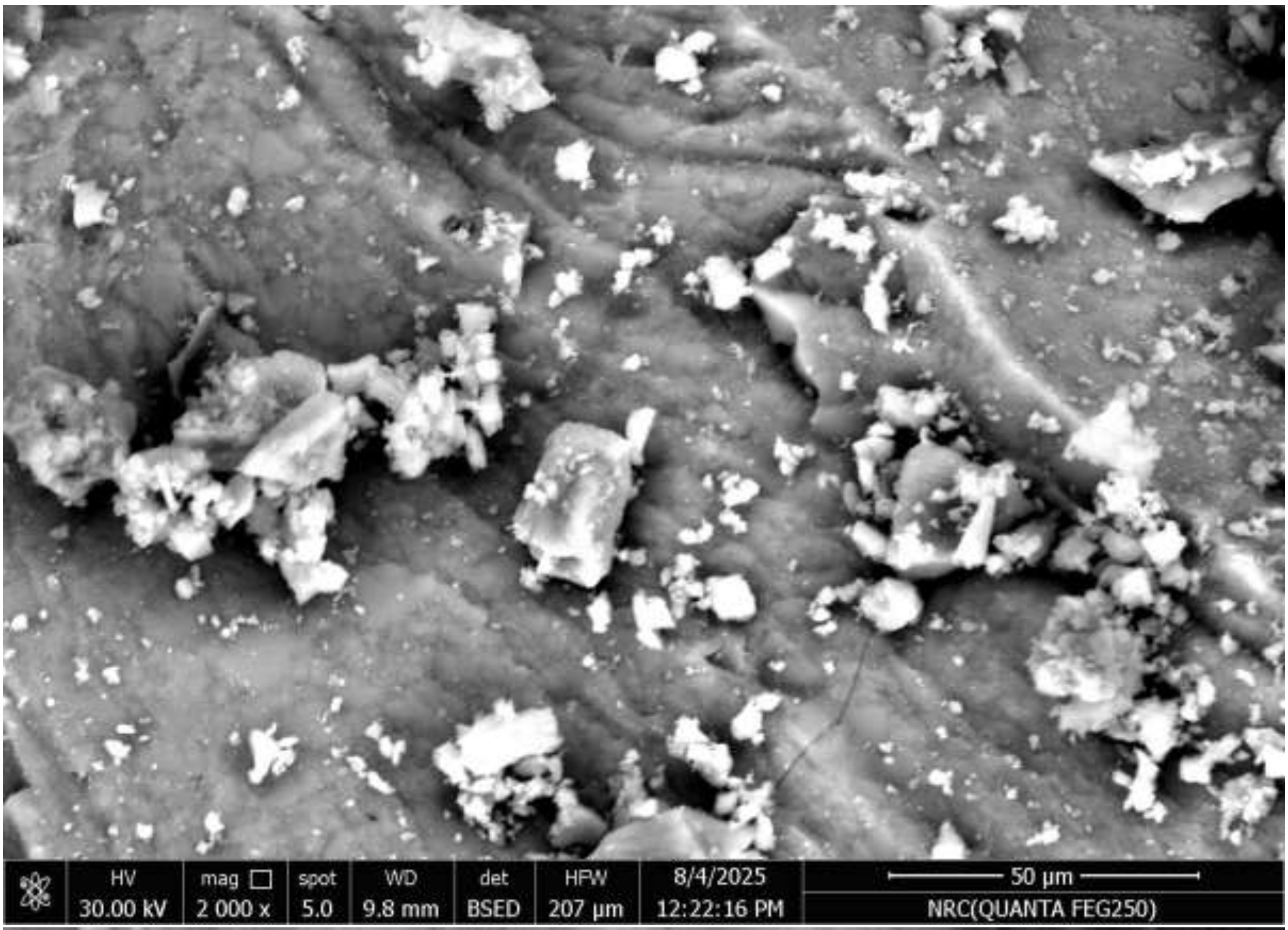
HV
30.00 kV
mag
2 000 x
spot
5.0
WD
9.8 mm
det
BSED
HFW
207 µm
8/4/2025
12:22:16 PM
50 µm
NRC(QUANTA FEG250)

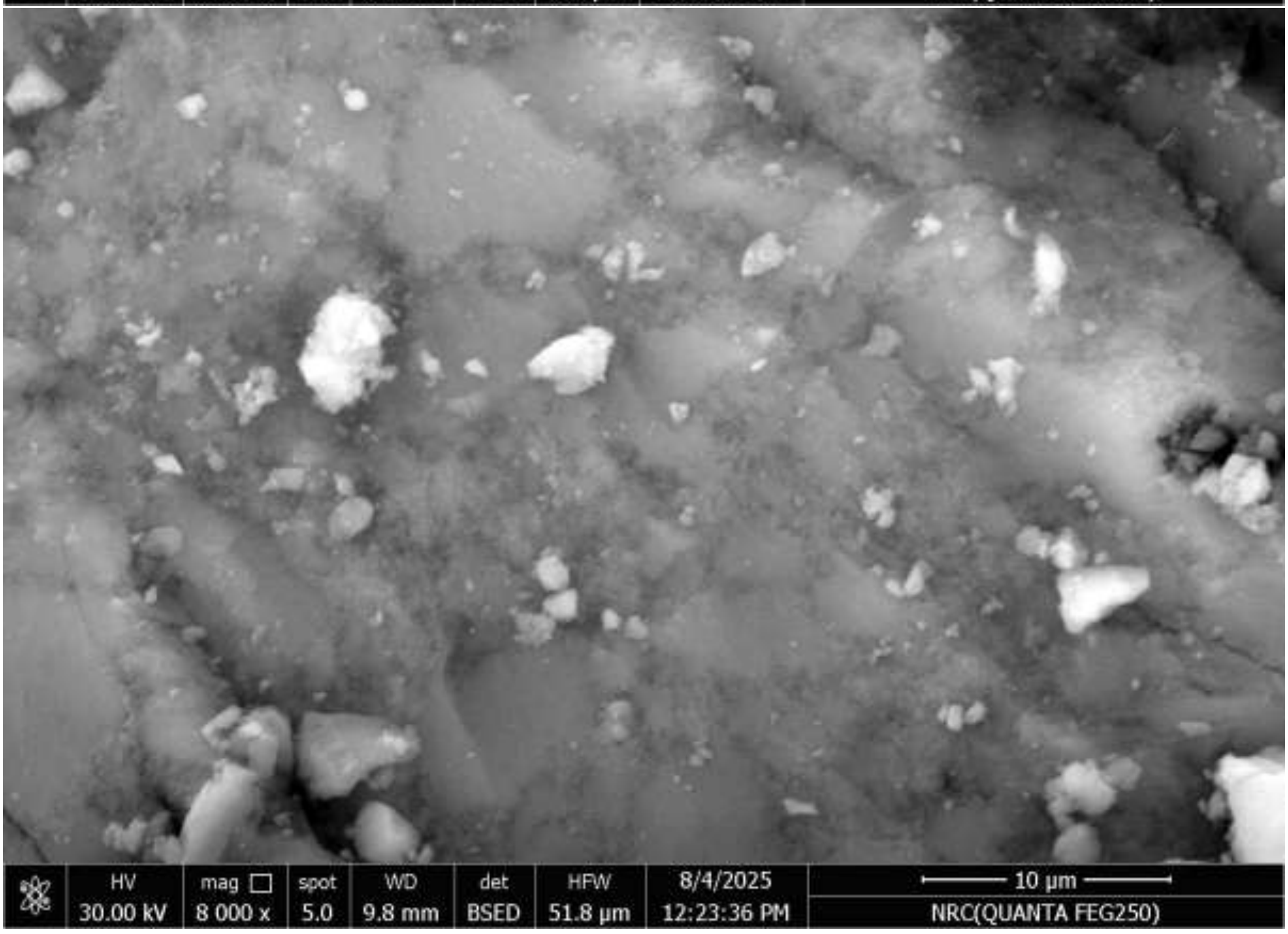
HV
30.00 kV
mag
8 000 x
spot
5.0
WD
9.8 mm
det
BSED
HFW
51.8 µm
8/4/2025
12:23:36 PM
10 µm
NRC(QUANTA FEG250)

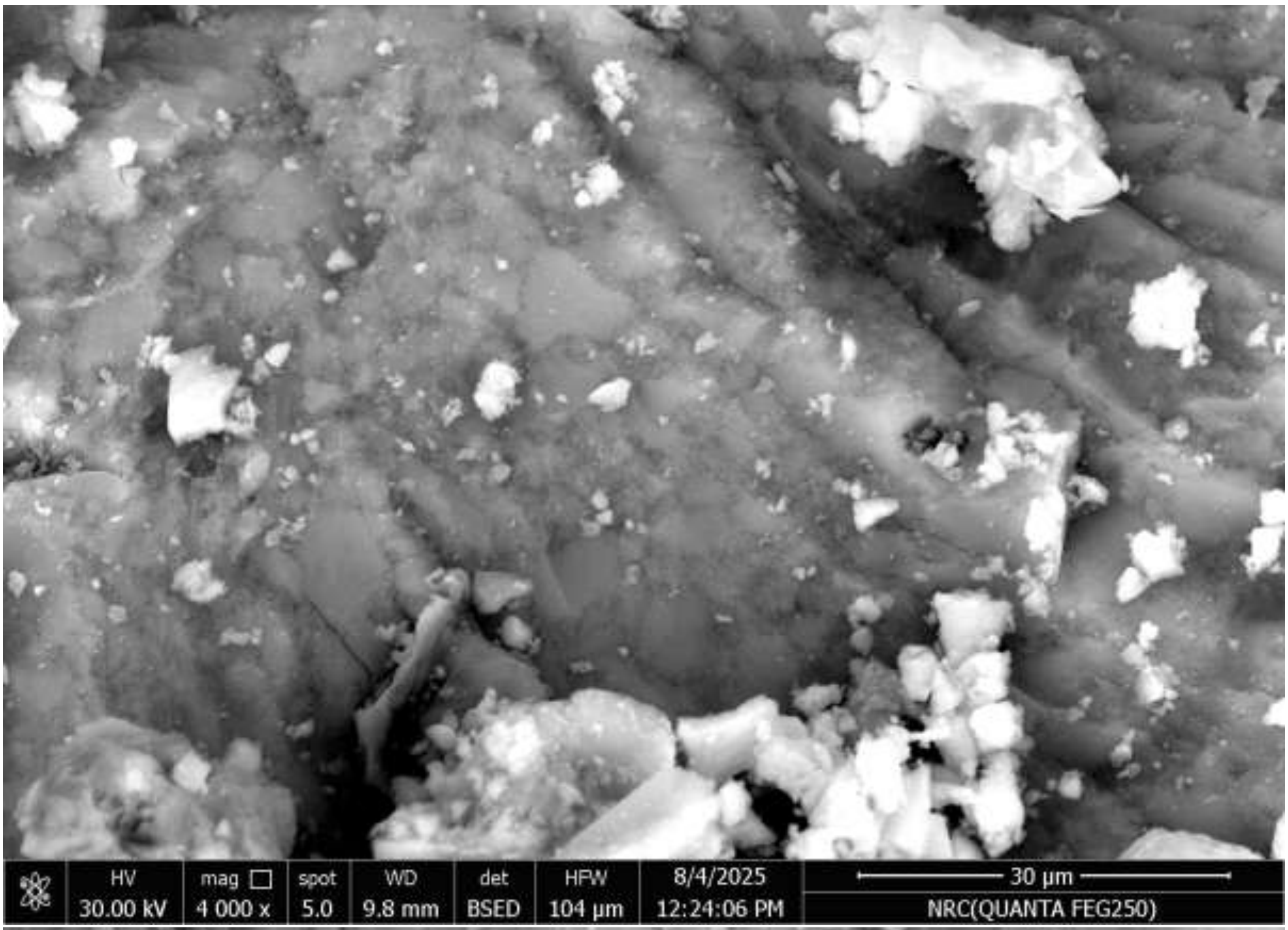
HV
30.00 kV
mag
4 000 x
spot
5.0
WD
9.8 mm
det
BSED
HFW
104 µm
8/4/2025
12:24:06 PM
30 µm
NRC(QUANTA FEG250)

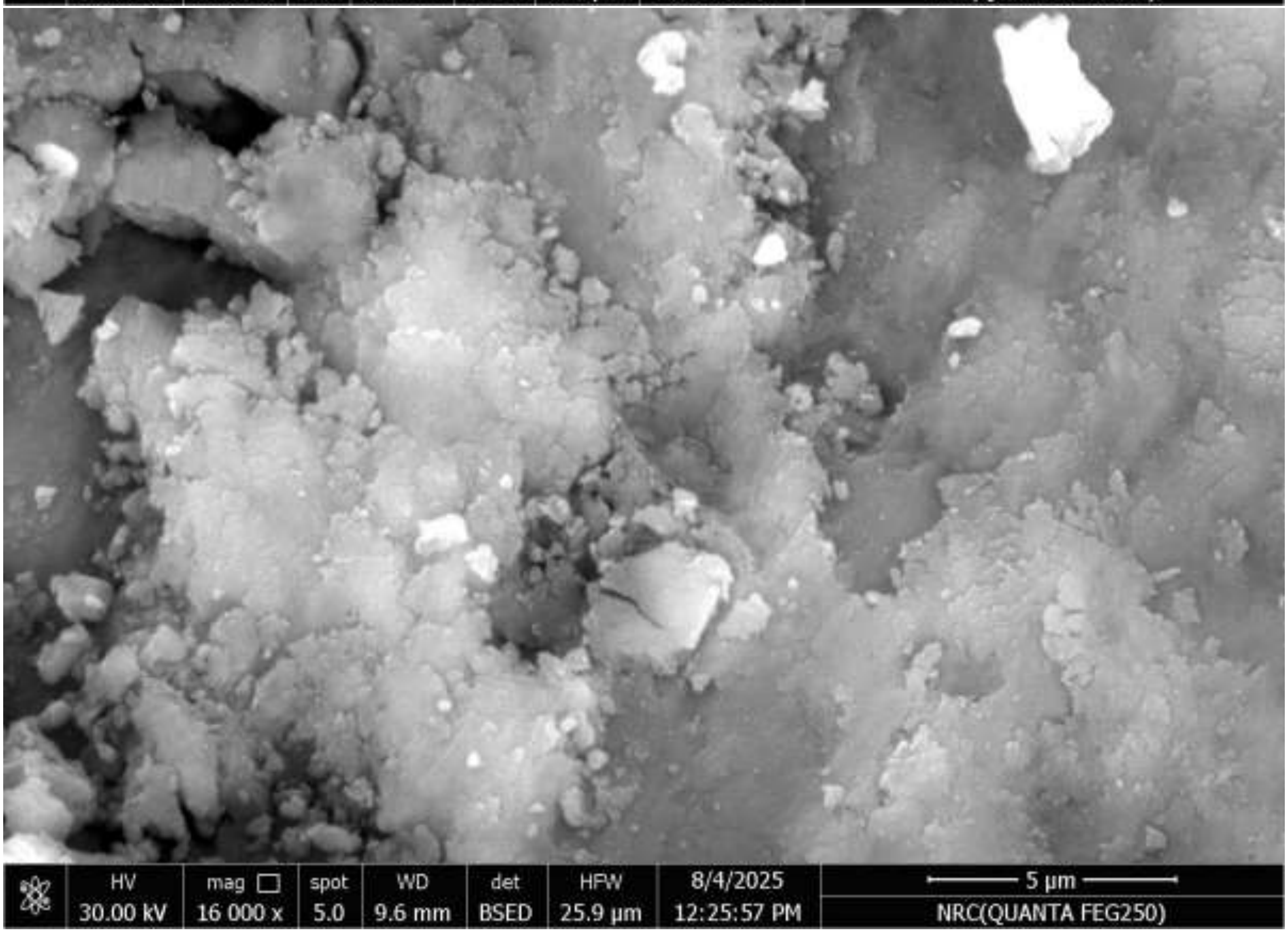
HV
30.00 kV
mag
16 000 x
spot
5.0
WD
9.6 mm
det
BSED
HFW
25.9 µm
8/4/2025
12:25:57 PM
5 µm
NRC(QUANTA FEG250)

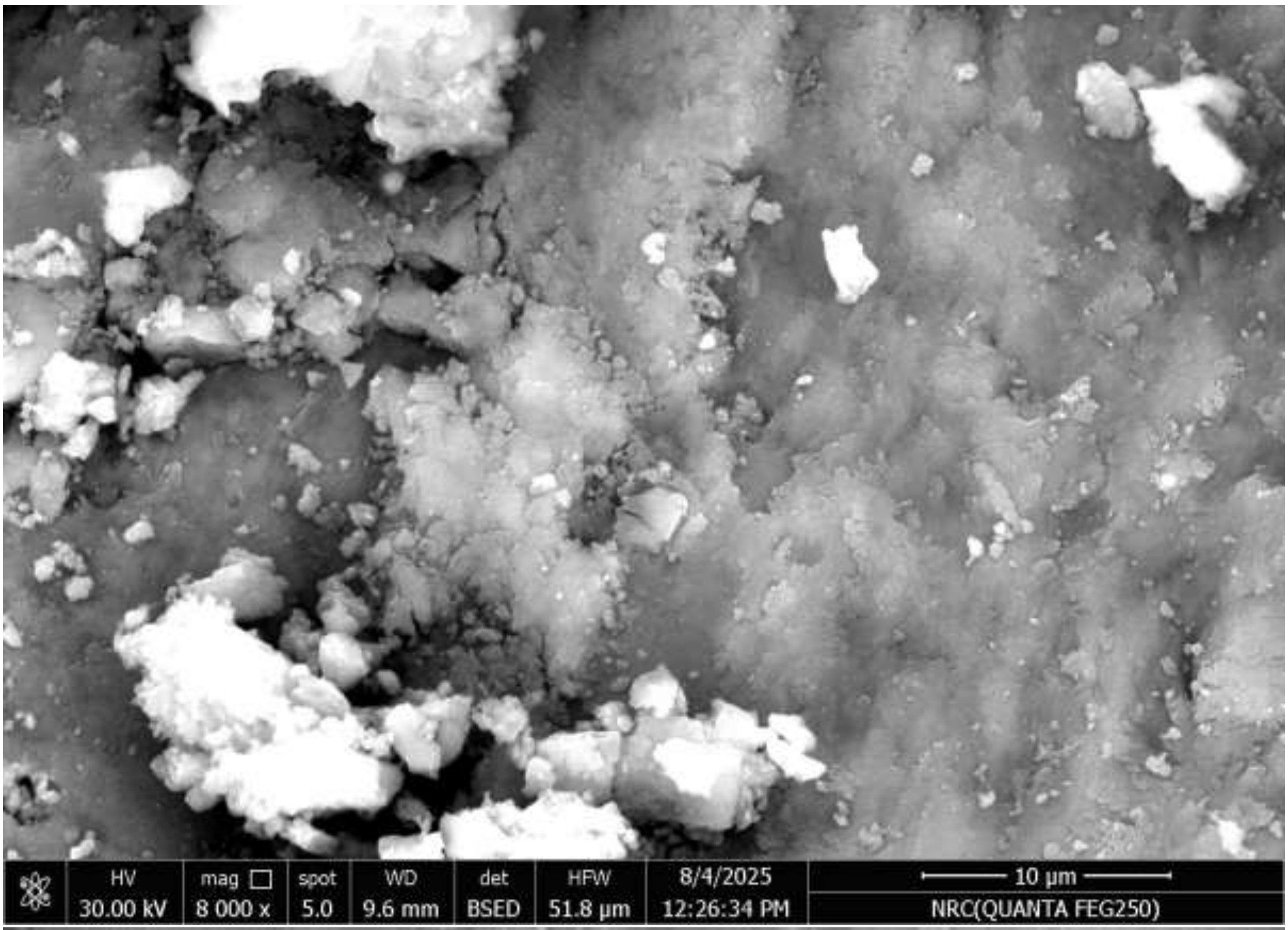
HV
30.00 kV
mag
8 000 x
spot
5.0
WD
9.6 mm
det
BSED
HFW
51.8 µm
8/4/2025
12:26:34 PM
10 µm
NRC(QUANTA FEG250)

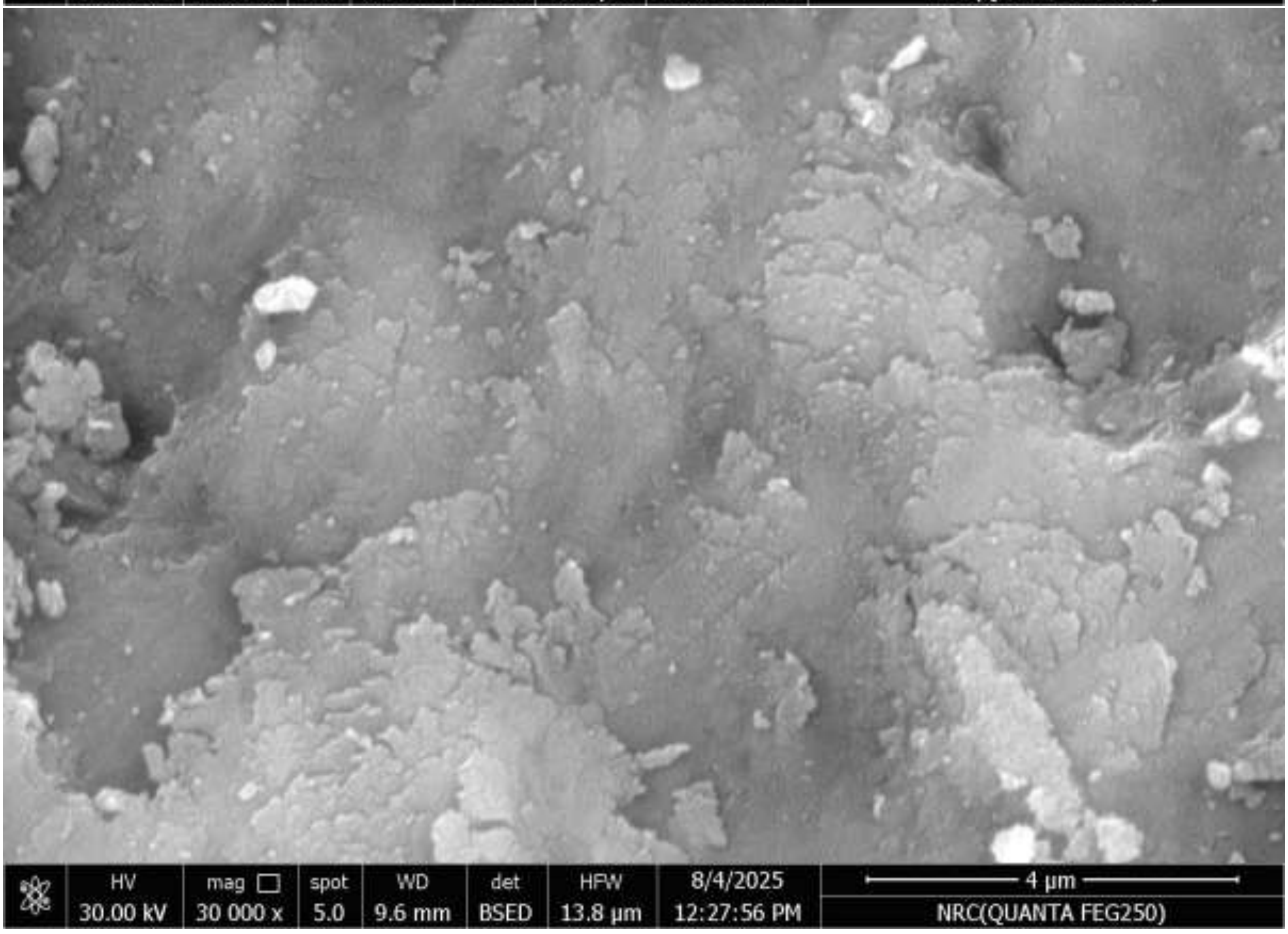
HV
30.00 kV
mag
30 000 x
spot
5.0
WD
9.6 mm
det
BSED
HFW
13.8 µm
8/4/2025
12:27:56 PM
4 µm
NRC(QUANTA FEG250)

LDH@AC

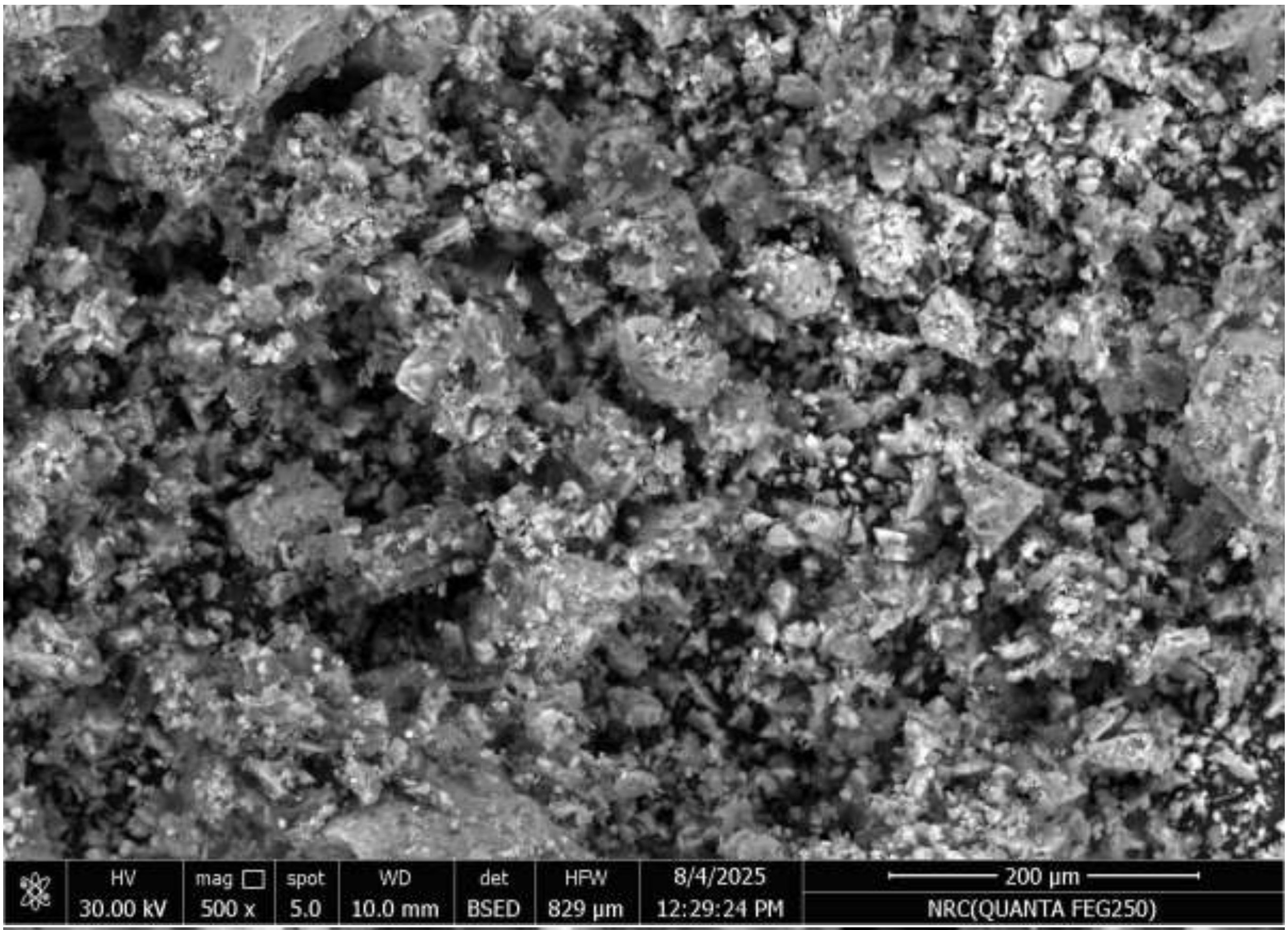
HV
30.00 kV
mag
500 x
spot
5.0
WD
10.0 mm
det
BSED
HFW
829 µm
8/4/2025
12:29:24 PM
200 µm
NRC(QUANTA FEG250)

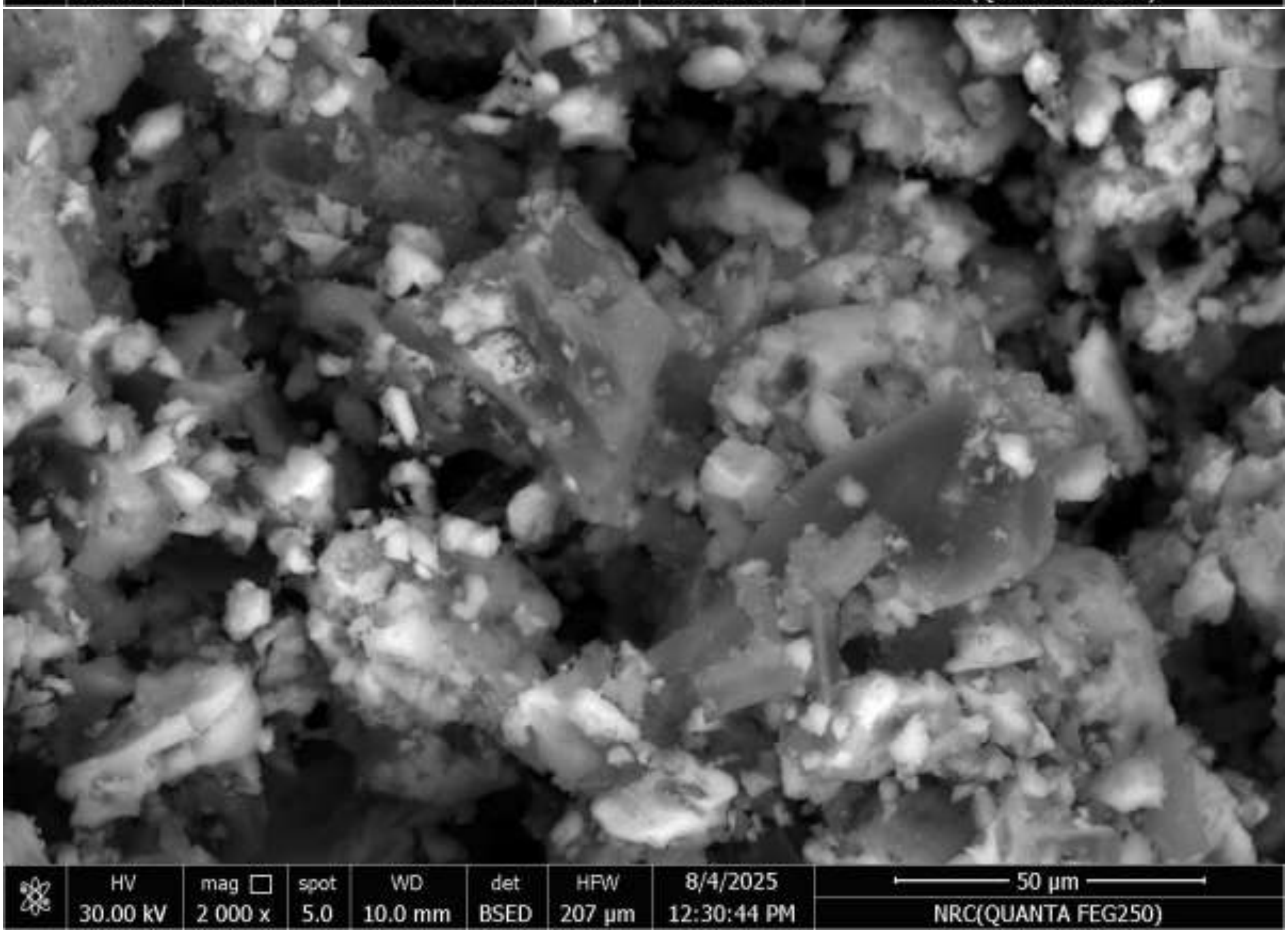
HV
30.00 kV
mag
2 000 x
spot
5.0
WD
10.0 mm
det
BSED
HFW
207 µm
8/4/2025
12:30:44 PM
50 µm
NRC(QUANTA FEG250)

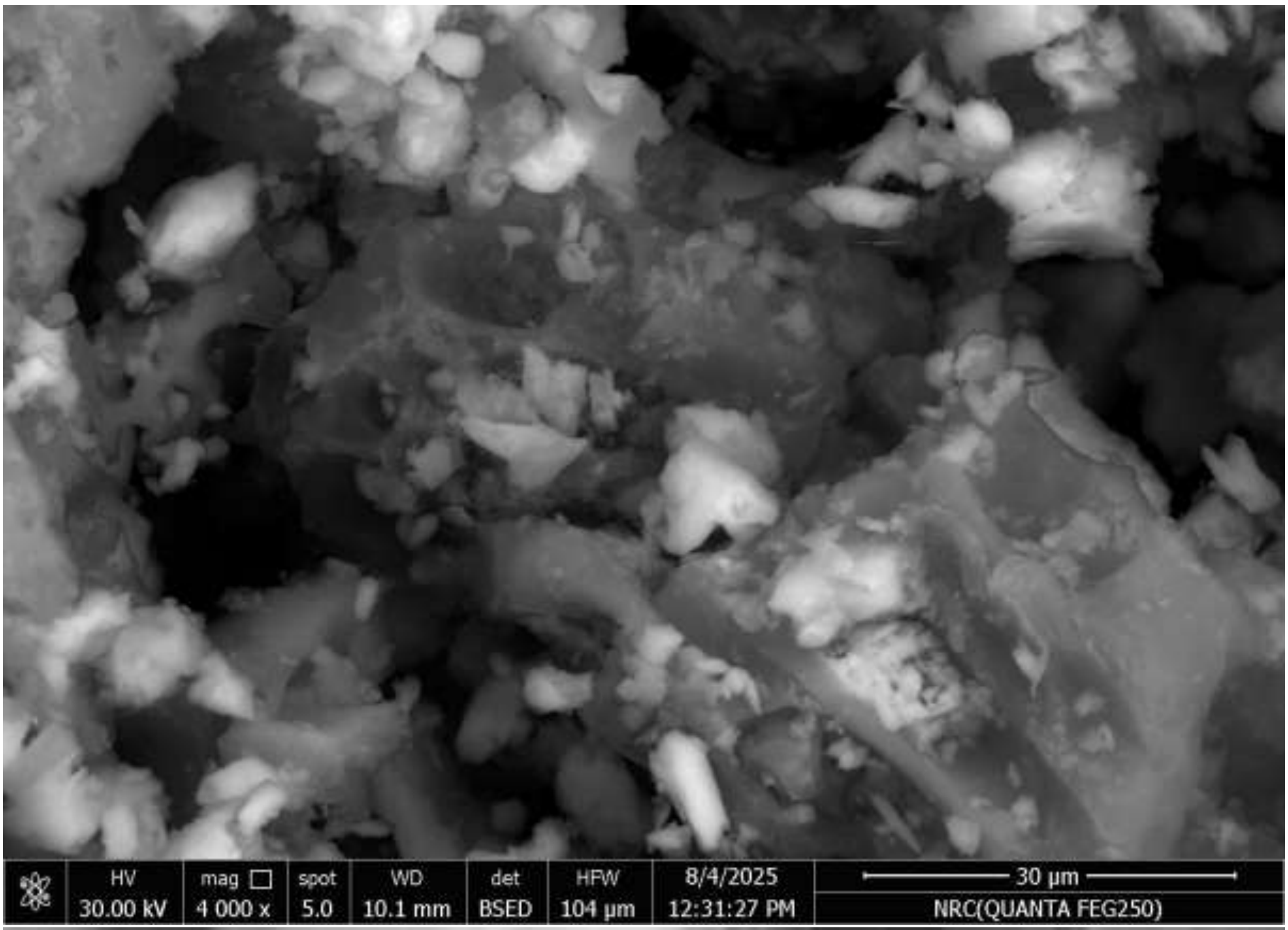
HV
30.00 kV
mag
4 000 x
spot
5.0
WD
10.1 mm
det
BSED
HFW
104 μm
8/4/2025
12:31:27 PM
30 μm
NRC(QUANTA FEG250)
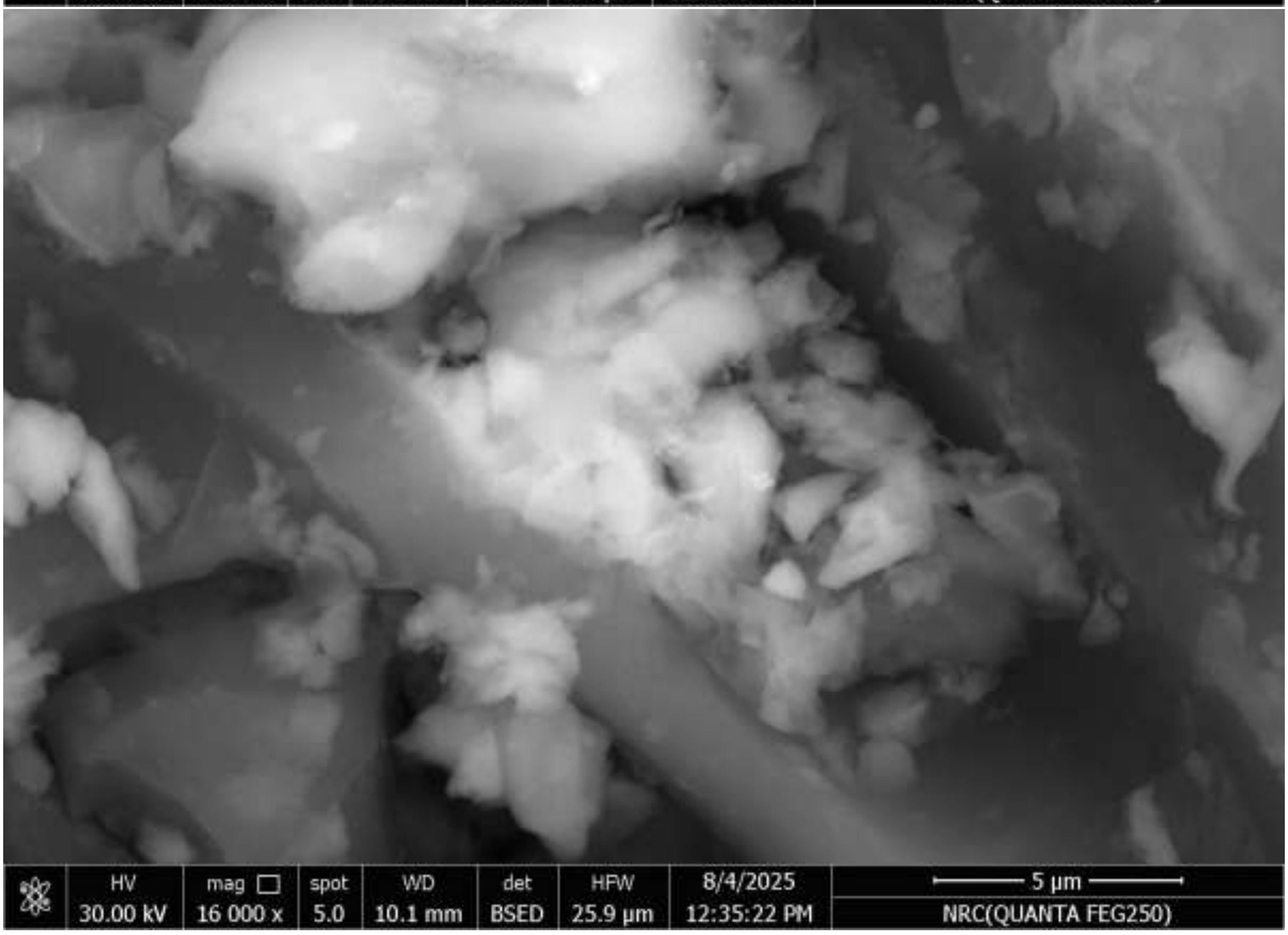
HV
30.00 kV
mag
16 000 x
spot
5.0
WD
10.1 mm
det
BSED
HFW
25.9 μm
8/4/2025
12:35:22 PM
5 μm
NRC(QUANTA FEG250)

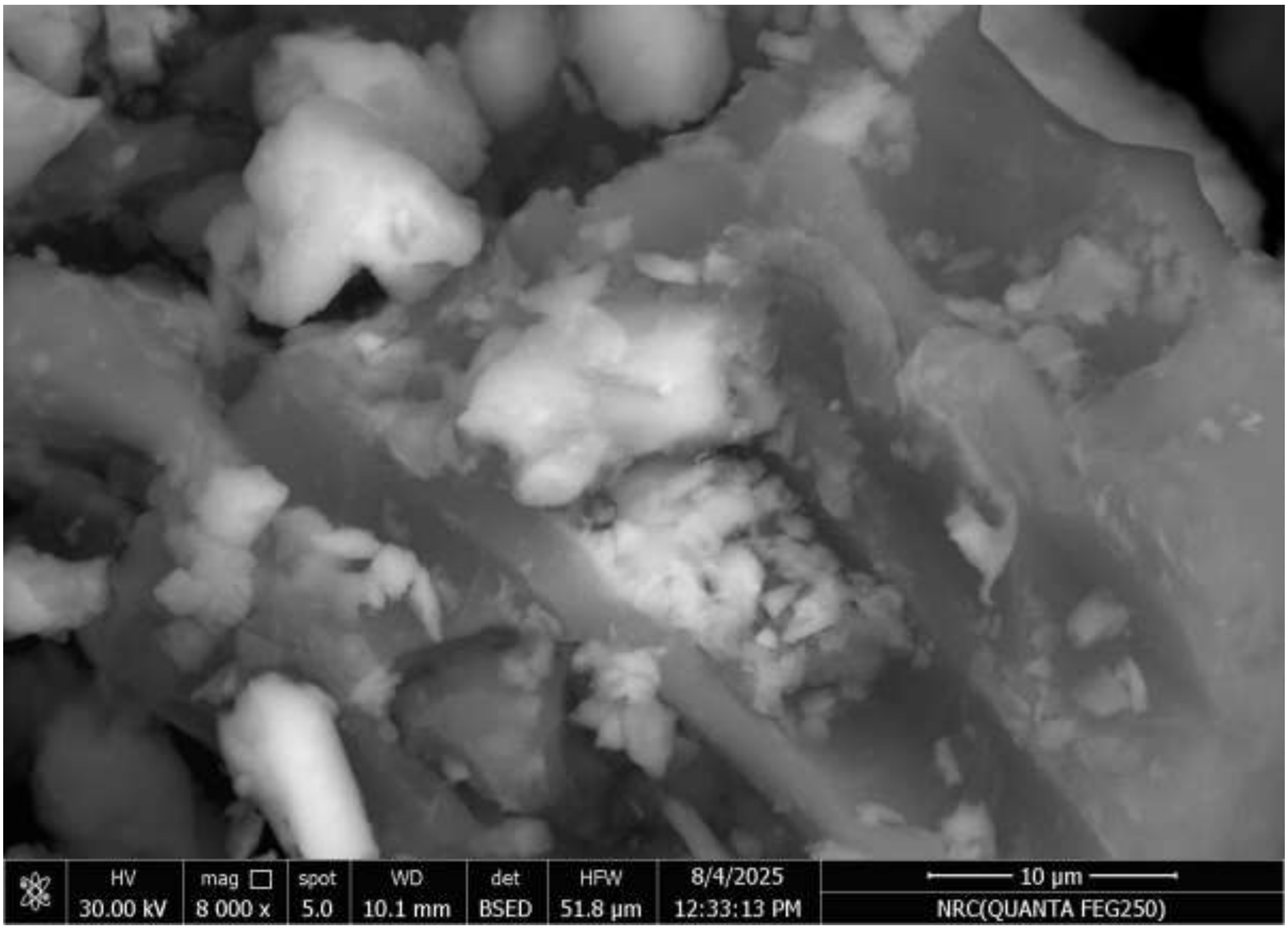
HV
30.00 kV
mag
8 000 x
spot
5.0
WD
10.1 mm
det
BSED
HFW
51.8 µm
8/4/2025
12:33:13 PM
10 µm
NRC(QUANTA FEG250)

[TA Ascii File]

TGA-51H

File Name : C:\TAData\TGA Data\ISMAILi\LDH-425--- der.txt

[File Information]

File Name: LDH-425---.tad

Sample Name: LDH-425---

Lot No: LDH-425---

Acquire Date: 25/05/27

Acquire Time: 10:03:23(-0800)

Detector Type: TGA-51H

Detector Serial No:

Operator:

Atmosphere: Air

Gas Flow: 0[ml/min]

Annotation:

Pan Name: Aluminum

Sample Weight: 5.545[mg]

[Measurements Program]

[Temperature Program]

Start Temp.

Temp. Rate Hold Temp. Hold Time

[C/min] [C] [min]

10.00	600.0	0

[Valve Program]

V1:	0

V2:	0

V1	V2	Direction		Hold Temp.	Hold Time
				[C]	[min]

- Repeat Program -

Init.	Final	Count

[PID Program]

P :	10

I :	10

D :	10

Temp.	Direction		P	I	D
[C]					

- Repeat Program -

Init.	Final	Count

[Data]

Time	Temp	TGA	DrTGA

sec	C	mg	mg/sec
0.000000	30.666916	5.547727	0.000427
1.000000	30.701530	5.545667	0.000759
2.000000	30.659225	5.550893	0.001129
3.000000	30.615374	5.549939	0.001469
4.000000	30.580761	5.551236	0.001175
5.000000	30.505379	5.552838	0.001263
6.000000	30.466146	5.555318	0.001061
7.000000	30.483068	5.554212	0.001396
8.000000	30.665377	5.555852	0.000702
9.000000	30.631531	5.559553	0.000877
10.000000	30.657684	5.556158	0.000599
11.000000	30.706148	5.558446	-0.000240
12.000000	30.710762	5.559400	0.000683
13.000000	30.626144	5.556730	-0.000042
14.000000	30.725378	5.560430	0.000259
15.000000	30.776146	5.557722	0.000923
16.000000	30.669991	5.560201	-0.000206
17.000000	30.722298	5.561460	0.000298
18.000000	30.730761	5.557531	-0.001015
19.000000	30.808453	5.560544	-0.000904
20.000000	30.836914	5.555585	0.000492
21.000000	30.668453	5.557912	0.000977
22.000000	30.833071	5.561307	0.001701
23.000000	30.810762	5.562566	0.000641
24.000000	30.796917	5.561765	-0.000458

25.000000	30.813070	5.560888	-0.000053
26.000000	30.797686	5.559858	-0.000389
27.000000	30.826147	5.563253	0.000229
28.000000	30.824608	5.558637	0.000084
29.000000	30.963070	5.562643	-0.000477
30.000000	31.052299	5.560583	-0.000389
31.000000	31.049225	5.559896	-0.001503
32.000000	31.034607	5.558065	0.000206
33.000000	31.075378	5.556386	0.001034
34.000000	31.209221	5.563367	0.001633
35.000000	31.153835	5.562414	0.001709
36.000000	31.156918	5.563215	0.000610
37.000000	31.129223	5.565008	0.000183
38.000000	31.273836	5.565122	-0.000317
39.000000	31.168449	5.562376	-0.001011
40.000000	31.212297	5.562948	-0.001217
41.000000	31.333069	5.561040	0.000225
42.000000	31.209221	5.559705	0.000271
43.000000	31.281532	5.565122	0.000874
44.000000	31.349993	5.562261	0.000877
45.000000	31.422298	5.564130	-0.000736
46.000000	31.542299	5.564588	-0.000385
47.000000	31.492300	5.560277	-0.000858
48.000000	31.572298	5.562261	0.000027
49.000000	31.554607	5.561002	0.000233
50.000000	31.544607	5.564359	-0.000473

51.000000	31.623068	5.560392	-0.000301
52.000000	31.633837	5.560201	-0.000340
53.000000	31.727684	5.561574	-0.000050
54.000000	31.768454	5.562070	0.000206
55.000000	31.906149	5.559209	-0.000225
56.000000	31.883837	5.562414	-0.001144
57.000000	31.889223	5.560277	0.000324
58.000000	31.937685	5.555814	0.000225
59.000000	31.938454	5.564130	0.000427
60.000000	31.993837	5.561613	0.000469
61.000000	32.160759	5.559515	-0.001442
62.000000	32.154606	5.560468	-0.001064
63.000000	32.135376	5.557493	0.000195
64.000000	32.156143	5.557302	0.000175
65.000000	32.296143	5.562070	0.000488
66.000000	32.242298	5.559057	0.000713
67.000000	32.267685	5.559057	-0.000050
68.000000	32.546143	5.562376	0.000790
69.000000	32.522293	5.560163	0.000847
70.000000	32.658451	5.562452	0.000492
71.000000	32.702301	5.563253	0.000031
72.000000	32.743065	5.563291	-0.000717
73.000000	32.871532	5.559896	-0.000458
74.000000	32.887680	5.560544	-0.000076
75.000000	32.953064	5.562337	0.000687
76.000000	33.038452	5.561689	0.000271

77.000000	33.008453	5.562757	0.000229
78.000000	33.123062	5.561689	-0.000172
79.000000	33.233833	5.563482	-0.000137
80.000000	33.266144	5.560468	0.000500
81.000000	33.399990	5.562681	0.000336
82.000000	33.471531	5.564588	0.000927
83.000000	33.446144	5.563100	-0.000198
84.000000	33.686146	5.564893	0.000164
85.000000	33.626144	5.561536	0.000252
86.000000	33.801529	5.566190	-0.000149
87.000000	33.740757	5.563711	-0.000580
88.000000	33.932297	5.563062	-0.001221
89.000000	34.046913	5.560201	-0.000290
90.000000	34.095375	5.561841	0.000435
91.000000	34.339989	5.562871	0.000626
92.000000	34.501526	5.563901	0.000500
93.000000	34.699223	5.562299	0.000416
94.000000	34.689220	5.564626	0.000931
95.000000	34.864609	5.564588	0.001057
96.000000	35.097683	5.567411	-0.000084
97.000000	35.258453	5.566190	0.000584
98.000000	35.346912	5.563406	0.000378
99.000000	35.457684	5.569509	0.000095
100.000000	35.626911	5.567640	-0.000671
101.000000	35.870758	5.564550	-0.002060
102.000000	35.994602	5.562528	-0.000568

103.000000	36.173065	5.561765	0.000587
104.000000	36.440762	5.566190	0.001198
105.000000	36.692295	5.565656	0.000908
106.000000	36.772297	5.566572	0.000046
107.000000	36.961529	5.566114	-0.000336
108.000000	36.983837	5.566190	-0.000546
109.000000	37.257687	5.564168	-0.000359
110.000000	37.508453	5.564817	-0.000443
111.000000	37.754604	5.565008	0.000118
112.000000	38.046143	5.563558	-0.000404
113.000000	38.222294	5.565389	0.000210
114.000000	38.413834	5.562604	0.000084
115.000000	38.727676	5.566534	0.000172
116.000000	38.735374	5.563406	-0.000656
117.000000	39.009991	5.565847	-0.001022
118.000000	39.276913	5.559667	-0.000172
119.000000	39.513836	5.563291	0.000118
120.000000	39.678452	5.563825	0.000282
121.000000	39.949074	5.564359	-0.000694
122.000000	40.048660	5.560544	-0.000824
123.000000	40.125076	5.561460	-0.000610
124.000000	40.420258	5.561155	0.000046
125.000000	40.671375	5.561002	-0.000618
126.000000	40.813698	5.561002	-0.000774
127.000000	41.086426	5.558446	0.000046
128.000000	41.377789	5.558561	0.000126

129.000000	41.546188	5.562452	0.000526
130.000000	41.843506	5.559629	-0.000244
131.000000	42.034267	5.560544	-0.001019
132.000000	42.221298	5.558294	-0.000771
133.000000	42.377033	5.558027	-0.000584
134.000000	42.569286	5.557035	-0.000221
135.000000	42.850952	5.558256	0.000019
136.000000	43.005203	5.557073	-0.000084
137.000000	43.359154	5.558103	-0.000595
138.000000	43.497757	5.556692	-0.000942
139.000000	43.786877	5.555470	-0.001217
140.000000	43.970924	5.553678	-0.000561
141.000000	44.055870	5.553525	-0.000523
142.000000	44.355427	5.554861	-0.000294
143.000000	44.460495	5.552266	-0.000805
144.000000	44.751854	5.552839	-0.001312
145.000000	44.944103	5.550511	-0.000259
146.000000	45.098347	5.549176	-0.000023
147.000000	45.310719	5.552800	0.000751
148.000000	45.491791	5.551579	0.000004
149.000000	45.750362	5.553067	-0.000824
150.000000	46.045441	5.549062	-0.001213
151.000000	46.228748	5.549939	-0.001472
152.000000	46.413548	5.547078	-0.000767
153.000000	46.694473	5.546697	-0.001030
154.000000	46.802525	5.546849	-0.000080

155.000000	47.054382	5.544904	-0.000263
156.000000	47.201183	5.547574	0.000652
157.000000	47.361389	5.545018	0.000874
158.000000	47.570034	5.550054	-0.000385
159.000000	47.739185	5.548032	-0.000980
160.000000	47.848724	5.544141	-0.002357
161.000000	48.052151	5.543073	-0.002140
162.000000	48.172863	5.540746	-0.000862
163.000000	48.386723	5.539029	-0.000515
164.000000	48.649765	5.541852	-0.000641
165.000000	48.914295	5.539945	-0.001263
166.000000	49.018616	5.537084	-0.001606
167.000000	49.203415	5.535100	-0.000267
168.000000	49.368095	5.536244	0.001171
169.000000	49.467949	5.539029	0.000702
170.000000	49.733963	5.540975	0.000103
171.000000	49.879250	5.536244	-0.000504
172.000000	50.073589	5.538152	-0.000259
173.000000	50.165329	5.537923	-0.000290
174.000000	50.342224	5.538838	-0.001095
175.000000	50.425262	5.534451	-0.001289
176.000000	50.709736	5.534413	-0.000763
177.000000	50.823090	5.533689	-0.000114
178.000000	51.011543	5.535405	-0.001060
179.000000	51.058472	5.533383	-0.000942
180.000000	51.273632	5.529263	-0.001667

181.000000	51.398540	5.532048	-0.001053
182.000000	51.584106	5.527738	-0.000126
183.000000	51.789154	5.528882	-0.000805
184.000000	51.911900	5.530217	0.000431
185.000000	52.177605	5.526784	0.000141
186.000000	52.177605	5.530942	-0.000687
187.000000	52.357391	5.529225	-0.000492
188.000000	52.441135	5.525563	-0.001091
189.000000	52.547997	5.527013	-0.000362
190.000000	52.766777	5.526593	-0.000217
191.000000	52.801426	5.526898	-0.000740
192.000000	52.922733	5.524533	-0.001377
193.000000	53.009369	5.524343	-0.000568
194.000000	53.176159	5.520986	-0.000080
195.000000	53.438976	5.525830	-0.000149
196.000000	53.480125	5.523389	-0.000443
197.000000	53.685905	5.522397	-0.001251
198.000000	53.680851	5.520490	-0.000809
199.000000	53.840416	5.521024	-0.000465
200.000000	54.049084	5.520032	0.000713
201.000000	54.190598	5.520299	0.001553
202.000000	54.406479	5.524419	0.001293
203.000000	54.507568	5.526593	0.000038
204.000000	54.663517	5.523351	-0.001884
205.000000	54.720566	5.521024	-0.001747
206.000000	54.854137	5.517781	0.000038

207.000000	55.023815	5.520642	-0.000275
208.000000	55.157383	5.523732	-0.000099
209.000000	55.259918	5.516675	-0.001102
210.000000	55.418762	5.519269	-0.001114
211.000000	55.569656	5.517362	-0.000351
212.000000	55.710453	5.517819	-0.000835
213.000000	55.896011	5.515645	-0.000782
214.000000	56.002151	5.515950	-0.000935
215.000000	56.258469	5.514386	0.000546
216.000000	56.312622	5.513776	0.000385
217.000000	56.449081	5.519460	0.000416
218.000000	56.580490	5.515340	0.000851
219.000000	56.773266	5.515683	0.000084
220.000000	56.871464	5.519917	-0.000031
221.000000	57.036804	5.517591	-0.000973
222.000000	57.296734	5.514234	-0.001148
223.000000	57.405762	5.513661	-0.000031
224.000000	57.527782	5.516141	0.000000
225.000000	57.696732	5.516484	0.000324
226.000000	57.867859	5.512822	-0.000389
227.000000	58.063522	5.516942	-0.000504
228.000000	58.173996	5.513967	-0.000095
229.000000	58.376877	5.513394	-0.000275
230.000000	58.509007	5.514119	0.000050
231.000000	58.800705	5.515492	-0.000462
232.000000	58.937889	5.513165	-0.001057

233.000000	59.035362	5.511563	-0.000580
234.000000	59.324894	5.510800	-0.000626
235.000000	59.475071	5.513776	0.000072
236.000000	59.508286	5.508931	0.000320
237.000000	59.828148	5.512860	-0.000347
238.000000	59.893845	5.512860	-0.000469
239.000000	60.115662	5.510076	-0.000854
240.000000	60.213505	5.507977	-0.000423
241.000000	60.471874	5.511029	-0.000397
242.000000	60.538780	5.510266	-0.000210
243.000000	60.708878	5.506948	-0.001060
244.000000	61.002831	5.508969	-0.000729
245.000000	61.109589	5.506375	0.000458
246.000000	61.251232	5.506909	0.000046
247.000000	61.367954	5.510266	0.000050
248.000000	61.525955	5.507253	-0.000244
249.000000	61.693932	5.506452	-0.000332
250.000000	61.990738	5.507596	-0.000191
251.000000	62.098911	5.508435	-0.000572
252.000000	62.288242	5.505307	-0.000774
253.000000	62.358704	5.504735	-0.001110
254.000000	62.483257	5.505574	0.000168
255.000000	62.679703	5.502751	0.000164
256.000000	62.933079	5.507138	0.000515
257.000000	63.146606	5.504773	0.000183
258.000000	63.320976	5.507138	-0.000965

259.000000	63.572227	5.503667	-0.001137
260.000000	63.862621	5.502866	-0.001198
261.000000	63.932365	5.501225	-0.000713
262.000000	64.113861	5.502370	-0.000050
263.000000	64.314575	5.500348	0.000710
264.000000	64.528091	5.503057	0.000202
265.000000	64.646957	5.504430	-0.000034
266.000000	64.853355	5.501340	-0.000721
267.000000	65.128807	5.501035	-0.000652
268.000000	65.250519	5.501149	-0.000122
269.000000	65.392860	5.501264	-0.000420
270.000000	65.636993	5.500615	-0.000862
271.000000	65.639122	5.499204	-0.000999
272.000000	65.978630	5.497869	-0.000393
273.000000	66.170082	5.497640	-0.000134
274.000000	66.404251	5.499433	-0.000439
275.000000	66.557983	5.497754	-0.000046
276.000000	66.748016	5.495618	-0.000423
277.000000	66.876854	5.499318	-0.000153
278.000000	67.102478	5.496533	-0.000340
279.000000	67.269730	5.496533	-0.001328
280.000000	67.562256	5.495313	-0.000919
281.000000	67.541626	5.493291	-0.000740
282.000000	67.716003	5.493558	-0.001286
283.000000	67.949448	5.493711	-0.001038
284.000000	68.082542	5.488675	-0.000729

285.000000	68.301758	5.490544	0.000046
286.000000	68.466888	5.491498	0.000259
287.000000	68.678978	5.492528	-0.000534
288.000000	68.726669	5.488980	-0.000774
289.000000	68.817062	5.489133	-0.000774
290.000000	69.064751	5.489324	-0.000034
291.000000	69.268311	5.488484	-0.000027
292.000000	69.496773	5.489133	-0.000420
293.000000	69.666893	5.489095	0.000206
294.000000	69.743759	5.486920	-0.000557
295.000000	69.969986	5.490621	0.000202
296.000000	70.261559	5.485585	0.000145
297.000000	70.385544	5.490773	-0.000359
298.000000	70.538811	5.487569	0.000034
299.000000	70.665504	5.487836	-0.000843
300.000000	70.837852	5.487226	-0.000553
301.000000	70.993851	5.486730	-0.000225
302.000000	71.241814	5.485356	-0.000565
303.000000	71.385536	5.487645	-0.000797
304.000000	71.641670	5.483945	-0.001457
305.000000	71.687988	5.483449	-0.001606
306.000000	71.940720	5.480168	0.000439
307.000000	72.119186	5.481503	0.000534
308.000000	72.160065	5.487111	0.000916
309.000000	72.330360	5.482648	-0.000568
310.000000	72.401886	5.484174	-0.000916

311.000000	72.573555	5.480130	-0.000259
312.000000	72.647118	5.483792	-0.000439
313.000000	72.927094	5.481542	0.000084
314.000000	73.087166	5.481275	-0.000401
315.000000	73.201614	5.481809	-0.000732
316.000000	73.375999	5.481656	-0.001831
317.000000	73.508148	5.477689	-0.001152
318.000000	73.696846	5.474179	-0.000469
319.000000	73.690712	5.479787	0.001011
320.000000	73.910049	5.478261	0.000214
321.000000	74.031998	5.480702	-0.000706
322.000000	74.232269	5.474790	-0.000843
323.000000	74.341270	5.477994	-0.001022
324.000000	74.582397	5.475400	-0.000431
325.000000	74.653244	5.475286	-0.000362
326.000000	74.836487	5.473989	-0.000015
327.000000	75.021774	5.476888	-0.000153
328.000000	75.131447	5.474523	-0.000340
329.000000	75.260887	5.474256	-0.000603
330.000000	75.497940	5.473607	-0.000824
331.000000	75.523140	5.474332	-0.000553
332.000000	75.697533	5.470365	-0.000328
333.000000	75.808563	5.473111	0.000603
334.000000	76.051064	5.472577	0.000778
335.000000	76.217278	5.476239	-0.000538
336.000000	76.380089	5.472692	-0.001068

337.000000	76.477501	5.470365	-0.001549
338.000000	76.671638	5.470173	-0.000900
339.000000	76.897110	5.469754	-0.000038
340.000000	77.047668	5.468495	-0.000248
341.000000	77.152565	5.471013	-0.000500
342.000000	77.299706	5.468304	0.000183
343.000000	77.446838	5.467351	-0.000156
344.000000	77.680496	5.471242	0.000156
345.000000	77.842621	5.468762	-0.000172
346.000000	78.023811	5.468381	-0.000610
347.000000	78.254066	5.467923	-0.000237
348.000000	78.341949	5.468609	0.000214
349.000000	78.563332	5.467465	-0.000488
350.000000	78.754066	5.469678	-0.001144
351.000000	78.880768	5.464947	-0.001774
352.000000	79.042206	5.464146	-0.001770
353.000000	79.331047	5.461361	-0.001061
354.000000	79.572868	5.462620	-0.000782
355.000000	79.655968	5.460407	0.000206
356.000000	79.957619	5.460712	0.000614
357.000000	80.052193	5.463345	0.001022
358.000000	80.195404	5.464222	0.000057
359.000000	80.374054	5.463764	-0.000641
360.000000	80.617172	5.460789	-0.000931
361.000000	80.802521	5.461857	-0.000629
362.000000	80.955658	5.460522	-0.000889

363.000000	81.059753	5.460751	-0.000530
364.000000	81.416367	5.456898	0.000256
365.000000	81.517105	5.461018	-0.000401
366.000000	81.666870	5.461666	-0.000496
367.000000	81.891174	5.456364	-0.001385
368.000000	81.990570	5.456745	-0.000759
369.000000	82.212204	5.456554	-0.000324
370.000000	82.331070	5.457775	-0.000240
371.000000	82.615822	5.454227	0.000130
372.000000	82.803192	5.456707	0.000511
373.000000	82.934837	5.457737	0.000866
374.000000	83.058411	5.458576	-0.000660
375.000000	83.279358	5.457623	-0.000587
376.000000	83.523148	5.453465	-0.001026
377.000000	83.570160	5.457355	-0.000652
378.000000	83.846855	5.453579	-0.000198
379.000000	84.019447	5.454304	-0.000614
380.000000	84.057739	5.453999	0.000587
381.000000	84.221603	5.454075	-0.000210
382.000000	84.337791	5.456631	-0.000374
383.000000	84.588966	5.451939	-0.000961
384.000000	84.789101	5.453197	-0.000919
385.000000	84.999977	5.450985	-0.000420
386.000000	85.315628	5.452511	-0.000759
387.000000	85.390167	5.450184	-0.000607
388.000000	85.546646	5.449802	-0.000843

389.000000	85.622543	5.449306	-0.000977
390.000000	85.836105	5.448734	-0.000317
391.000000	85.900589	5.445835	-0.000702
392.000000	86.218239	5.449955	-0.000301
393.000000	86.357933	5.445187	-0.000195
394.000000	86.647385	5.447552	-0.000034
395.000000	86.845505	5.446064	-0.000179
396.000000	86.991913	5.449345	-0.001511
397.000000	87.100037	5.443394	-0.001312
398.000000	87.285416	5.441334	-0.001713
399.000000	87.435844	5.443508	0.000145
400.000000	87.552025	5.440723	0.001289
401.000000	87.826706	5.444424	0.000866
402.000000	87.878418	5.447323	0.000568
403.000000	88.055046	5.444538	-0.000664
404.000000	88.175262	5.443508	-0.001289
405.000000	88.351227	5.443012	-0.000935
406.000000	88.503662	5.441639	-0.000587
407.000000	88.703133	5.440800	-0.000401
408.000000	88.901917	5.441677	-0.000668
409.000000	89.012070	5.440990	0.000088
410.000000	89.181976	5.438206	0.000214
411.000000	89.357262	5.442974	0.000309
412.000000	89.532547	5.441753	-0.000229
413.000000	89.644707	5.440762	-0.001228
414.000000	89.779022	5.438168	-0.001404

415.000000	89.860283	5.438625	-0.002274
416.000000	90.063545	5.435802	-0.000927
417.000000	90.264885	5.430576	-0.000168
418.000000	90.490044	5.437557	0.000381
419.000000	90.597321	5.436909	0.000561
420.000000	90.819176	5.434544	-0.000240
421.000000	90.990051	5.434887	-0.000244
422.000000	91.055618	5.437366	0.000160
423.000000	91.231110	5.434277	-0.000126
424.000000	91.378128	5.435650	-0.001060
425.000000	91.516541	5.435116	0.000149
426.000000	91.768845	5.431644	-0.000786
427.000000	91.905930	5.437023	-0.000881
428.000000	92.021828	5.430767	-0.000542
429.000000	92.311241	5.431149	-0.000439
430.000000	92.379448	5.431873	0.000656
431.000000	92.484085	5.434277	0.000038
432.000000	92.666855	5.432484	-0.000324
433.000000	92.808571	5.431034	-0.001198
434.000000	92.919838	5.431873	-0.001183
435.000000	93.127800	5.428593	-0.000370
436.000000	93.339714	5.427792	0.000019
437.000000	93.374153	5.431225	-0.000065
438.000000	93.695335	5.430653	0.000042
439.000000	93.895325	5.426838	-0.000389
440.000000	94.064880	5.430195	-0.000412

441.000000	94.286079	5.429508	-0.000340
442.000000	94.451630	5.427258	-0.000664
443.000000	94.717857	5.426609	-0.000450
444.000000	94.852951	5.428326	0.000469
445.000000	95.149658	5.426723	-0.000069
446.000000	95.177467	5.429546	-0.001190
447.000000	95.474152	5.425655	-0.000893
448.000000	95.544350	5.422909	-0.000763
449.000000	95.782082	5.425579	-0.000179
450.000000	96.005272	5.425770	0.000629
451.000000	96.116539	5.423328	-0.000492
452.000000	96.293365	5.427181	-0.000633
453.000000	96.479446	5.422413	-0.001068
454.000000	96.694008	5.423061	-0.001137
455.000000	96.767517	5.420048	0.000099
456.000000	97.025810	5.422680	-0.000740
457.000000	97.077469	5.423100	-0.000568
458.000000	97.357597	5.417835	-0.000805
459.000000	97.584076	5.419628	-0.000355
460.000000	97.715202	5.420391	0.000271
461.000000	97.860237	5.420048	0.000164
462.000000	97.967529	5.418980	-0.000004
463.000000	98.092697	5.421154	-0.000252
464.000000	98.409248	5.419819	-0.000614
465.000000	98.478119	5.418369	-0.001274
466.000000	98.691360	5.417301	-0.001232

467.000000	98.758911	5.416042	-0.000652
468.000000	99.041702	5.414822	-0.000389
469.000000	99.148331	5.416348	0.000114
470.000000	99.366203	5.415203	-0.000175
471.000000	99.437729	5.416424	-0.000954
472.000000	99.566200	5.413906	-0.001045
473.000000	99.705933	5.412228	-0.000488
474.000000	99.916527	5.412075	-0.000038
475.000000	100.095062	5.414898	0.000214
476.000000	100.313766	5.412380	-0.000397
477.000000	100.470985	5.413143	-0.000469
478.000000	100.562477	5.410969	0.000530
479.000000	100.854355	5.413258	-0.000202
480.000000	100.968407	5.414974	-0.000359
481.000000	101.017365	5.410130	-0.000744
482.000000	101.148178	5.410740	-0.000381
483.000000	101.335030	5.411655	-0.000011
484.000000	101.541855	5.412304	-0.000122
485.000000	101.726135	5.409290	-0.001060
486.000000	101.922005	5.411312	-0.000969
487.000000	102.090828	5.406849	-0.001064
488.000000	102.171371	5.408680	-0.001476
489.000000	102.351143	5.405285	-0.000359
490.000000	102.503181	5.404713	-0.000107
491.000000	102.662338	5.407040	0.001331
492.000000	102.846619	5.407269	0.001186

493.000000	103.034760	5.410664	0.000149
494.000000	103.123680	5.408833	-0.000366
495.000000	103.297668	5.407001	-0.001560
496.000000	103.523163	5.407269	-0.001389
497.000000	103.668137	5.403645	-0.001202
498.000000	103.808601	5.403568	-0.001392
499.000000	103.941345	5.402843	-0.000782
500.000000	104.037338	5.400707	0.000061
501.000000	104.200996	5.401165	0.000614
502.000000	104.334381	5.404713	0.001205
503.000000	104.537987	5.403912	0.000004
504.000000	104.771881	5.405361	-0.000793
505.000000	104.938766	5.400860	-0.000729
506.000000	105.029610	5.402271	-0.000980
507.000000	105.152046	5.401814	-0.000648
508.000000	105.328583	5.399982	-0.001190
509.000000	105.551521	5.398762	-0.000664
510.000000	105.762863	5.397846	-0.000484
511.000000	105.867882	5.399563	-0.000374
512.000000	106.012848	5.397160	0.000263
513.000000	106.255768	5.397236	0.000011
514.000000	106.490936	5.400326	-0.000103
515.000000	106.730003	5.398037	-0.000446
516.000000	106.891075	5.396244	-0.001228
517.000000	107.068916	5.397045	-0.000240
518.000000	107.126251	5.394680	-0.000050

519.000000	107.393013	5.397617	-0.000484
520.000000	107.517357	5.395710	-0.000290
521.000000	107.737717	5.394108	0.000179
522.000000	107.896225	5.394985	0.000019
523.000000	108.003830	5.398876	0.000065
524.000000	108.124336	5.393421	-0.000954
525.000000	108.288635	5.395214	-0.001602
526.000000	108.395576	5.392048	-0.000008
527.000000	108.677818	5.391552	0.000584
528.000000	108.737083	5.395214	0.000362
529.000000	108.887215	5.396549	-0.000828
530.000000	109.094688	5.391361	-0.001972
531.000000	109.246101	5.389339	-0.000843
532.000000	109.548950	5.388958	0.000710
533.000000	109.608215	5.393536	0.000782
534.000000	109.791855	5.392811	-0.000748
535.000000	109.974815	5.391323	-0.001297
536.000000	110.192047	5.386326	-0.000492
537.000000	110.314476	5.390293	0.000462
538.000000	110.475800	5.390865	0.000237
539.000000	110.746361	5.391361	-0.000687
540.000000	110.920250	5.386974	-0.001148
541.000000	111.028854	5.388805	-0.001446
542.000000	111.102928	5.386402	-0.000744
543.000000	111.246040	5.384418	-0.000614
544.000000	111.362183	5.385448	-0.000179

545.000000	111.713081	5.386211	-0.000122
546.000000	111.865631	5.384609	-0.000435
547.000000	111.996834	5.384228	-0.000324
548.000000	112.127403	5.384266	0.000031
549.000000	112.266769	5.384762	-0.000381
550.000000	112.535431	5.384495	0.000210
551.000000	112.637115	5.382206	-0.000256
552.000000	112.851830	5.386593	-0.000114
553.000000	112.988037	5.382435	0.000706
554.000000	113.171349	5.383808	-0.000362
555.000000	113.341461	5.387127	-0.000183
556.000000	113.571228	5.382435	-0.000881
557.000000	113.799713	5.382206	-0.001728
558.000000	113.935944	5.381863	-0.001228
559.000000	114.134918	5.378773	-0.001118
560.000000	114.297516	5.378010	-0.001320
561.000000	114.415535	5.378544	-0.000553
562.000000	114.572479	5.375378	0.000084
563.000000	114.830475	5.377323	0.000099
564.000000	114.985535	5.379040	0.000240
565.000000	115.081573	5.377209	0.000385
566.000000	115.256088	5.376637	0.000141
567.000000	115.440025	5.380451	-0.000149
568.000000	115.708687	5.378124	-0.000511
569.000000	115.796577	5.375721	-0.001022
570.000000	115.948486	5.376446	-0.001198

571.000000	116.175728	5.376179	-0.000813
572.000000	116.352753	5.371906	-0.000729
573.000000	116.434357	5.373928	-0.000309
574.000000	116.546738	5.373928	0.000458
575.000000	116.743225	5.373623	-0.000217
576.000000	116.914597	5.374348	-0.000717
577.000000	117.045166	5.372631	-0.000999
578.000000	117.251678	5.370838	-0.001019
579.000000	117.478943	5.370380	-0.000282
580.000000	117.633362	5.370380	-0.000183
581.000000	117.797836	5.371449	-0.000427
582.000000	117.888229	5.369389	-0.000736
583.000000	117.973602	5.368740	-0.000340
584.000000	118.163818	5.368053	-0.000229
585.000000	118.362183	5.370419	-0.000095
586.000000	118.533554	5.367405	-0.000336
587.000000	118.607628	5.368587	-0.000057
588.000000	118.839905	5.367290	0.000694
589.000000	119.081573	5.370190	0.000202
590.000000	119.133057	5.370075	-0.000237
591.000000	119.297516	5.368206	-0.000816
592.000000	119.496521	5.367100	-0.001099
593.000000	119.696136	5.367596	0.000015
594.000000	119.870026	5.364887	0.000088
595.000000	120.082909	5.369389	-0.000282
596.000000	120.172729	5.366642	-0.000034

597.000000	120.427216	5.365307	-0.000134
598.000000	120.603683	5.366756	-0.001045
599.000000	120.801842	5.368664	-0.000839
600.000000	120.918846	5.359737	-0.000626
601.000000	121.162811	5.364620	-0.000912
602.000000	121.299042	5.365650	-0.000645
603.000000	121.510803	5.361149	-0.001640
604.000000	121.522568	5.358250	-0.000523
605.000000	121.652596	5.360119	0.000317
606.000000	121.902130	5.363552	0.000469
607.000000	122.072411	5.360080	-0.001240
608.000000	122.209251	5.360615	-0.000698
609.000000	122.443306	5.355388	-0.000042
610.000000	122.606148	5.362408	-0.000084
611.000000	122.837128	5.358974	0.000645
612.000000	122.946724	5.358402	-0.000317
613.000000	123.134323	5.360615	0.000137
614.000000	123.380768	5.360004	-0.000999
615.000000	123.508324	5.358860	-0.001366
616.000000	123.651367	5.354282	-0.001152
617.000000	124.001816	5.356647	-0.000267
618.000000	124.050728	5.355350	0.000309
619.000000	124.367767	5.356991	-0.000259
620.000000	124.521957	5.355656	-0.000019
621.000000	124.670555	5.355198	-0.000134
622.000000	124.751045	5.356152	-0.000507

623.000000	124.953537	5.356075	-0.000851
624.000000	125.144859	5.352680	-0.000347
625.000000	125.282944	5.352680	-0.000580
626.000000	125.447639	5.356113	-0.000320
627.000000	125.615448	5.351459	-0.001221
628.000000	125.909569	5.351688	-0.000962
629.000000	126.128769	5.348787	0.000408
630.000000	126.250122	5.352642	0.000469
631.000000	126.448883	5.353023	0.000035
632.000000	126.610497	5.351917	-0.000515
633.000000	126.744232	5.349322	-0.000786
634.000000	126.949203	5.351916	-0.001305
635.000000	127.164063	5.349093	-0.001358
636.000000	127.237740	5.345507	-0.001625
637.000000	127.449493	5.345735	-0.000217
638.000000	127.554771	5.345469	0.000015
639.000000	127.709564	5.348025	0.000378
640.000000	127.852600	5.344439	0.000034
641.000000	128.051346	5.348138	-0.000336
642.000000	128.189423	5.345583	0.000374
643.000000	128.363434	5.345774	-0.000656
644.000000	128.378906	5.347491	-0.000477
645.000000	128.573334	5.343904	-0.000809
646.000000	128.794373	5.344133	-0.000881
647.000000	128.838974	5.343408	-0.000263
648.000000	129.040207	5.343332	-0.000866

649.000000	129.268692	5.342988	-0.001385
650.000000	129.402435	5.340013	-0.000080
651.000000	129.633392	5.338144	0.000366
652.000000	129.731842	5.345354	0.000347
653.000000	129.924347	5.342149	-0.000179
654.000000	130.075012	5.339746	-0.001362
655.000000	130.199234	5.340051	-0.000214
656.000000	130.382599	5.339593	-0.000050
657.000000	130.513412	5.341157	-0.001148
658.000000	130.803131	5.338945	-0.001385
659.000000	130.919281	5.334634	-0.001995
660.000000	131.017685	5.335931	-0.000561
661.000000	131.249359	5.332689	0.000225
662.000000	131.363037	5.337114	0.000370
663.000000	131.597748	5.335168	0.000698
664.000000	131.718781	5.336542	-0.000389
665.000000	131.878922	5.336465	0.000027
666.000000	132.129532	5.334520	-0.000160
667.000000	132.301926	5.336313	-0.000282
668.000000	132.440674	5.335817	-0.000572
669.000000	132.623428	5.334405	-0.001236
670.000000	132.712067	5.332613	-0.000549
671.000000	132.858765	5.331735	-0.000149
672.000000	133.185165	5.334405	-0.000072
673.000000	133.245071	5.332765	-0.000427
674.000000	133.433334	5.331735	-0.001289

675.000000	133.597153	5.330934	-0.000706
676.000000	133.848373	5.328874	-0.001217
677.000000	134.001175	5.330667	-0.000576
678.000000	134.149719	5.325784	0.000336
679.000000	134.295197	5.329599	0.000103
680.000000	134.479187	5.331087	0.000214
681.000000	134.661316	5.328531	-0.000050
682.000000	134.799469	5.327386	-0.000504
683.000000	134.926605	5.331201	-0.000298
684.000000	135.119141	5.327234	0.000263
685.000000	135.386261	5.327119	-0.000519
686.000000	135.518921	5.330743	-0.000790
687.000000	135.701050	5.326852	-0.001671
688.000000	135.806793	5.323419	-0.001007
689.000000	136.078186	5.322427	-0.000351
690.000000	136.166214	5.327920	0.000256
691.000000	136.382614	5.322847	-0.000214
692.000000	136.537247	5.324487	-0.000755
693.000000	136.661926	5.323076	-0.000252
694.000000	136.803146	5.324029	-0.000950
695.000000	136.957153	5.321817	-0.000793
696.000000	137.128952	5.320367	-0.000286
697.000000	137.338577	5.320940	-0.000015
698.000000	137.438232	5.323038	0.000328
699.000000	137.718185	5.320405	0.000160
700.000000	137.753006	5.322275	-0.000370

701.000000	137.968170	5.322122	-0.000446
702.000000	138.222458	5.320329	-0.001076
703.000000	138.408264	5.319147	-0.001369
704.000000	138.561707	5.318384	-0.001228
705.000000	138.721848	5.316247	0.000183
706.000000	138.845322	5.315637	-0.000465
707.000000	139.042740	5.321435	-0.000721
708.000000	139.264008	5.313463	-0.000900
709.000000	139.484070	5.313730	-0.001297
710.000000	139.589813	5.314989	0.000374
711.000000	139.826904	5.314188	0.000271
712.000000	140.020874	5.315103	0.000252
713.000000	140.158081	5.315027	-0.000095
714.000000	140.287827	5.315828	-0.000061
715.000000	140.574478	5.313348	-0.000057
716.000000	140.675278	5.315637	-0.000439
717.000000	140.824341	5.314836	-0.001541
718.000000	140.978821	5.312891	-0.001423
719.000000	141.071167	5.307016	0.000137
720.000000	141.331879	5.312433	0.000515
721.000000	141.447144	5.315752	0.000393
722.000000	141.756134	5.311098	-0.000786
723.000000	141.844849	5.309648	-0.000961
724.000000	141.969803	5.311555	-0.000641
725.000000	142.075394	5.310716	-0.000298
726.000000	142.212997	5.307359	-0.000458

727.000000	142.561218	5.310258	-0.000267
728.000000	142.626389	5.309495	-0.000828
729.000000	142.806244	5.308313	-0.000767
730.000000	142.932373	5.304193	-0.000763
731.000000	143.132126	5.309076	-0.000835
732.000000	143.235931	5.305299	0.000084
733.000000	143.398254	5.303583	-0.000389
734.000000	143.668030	5.307359	-0.000145
735.000000	143.882874	5.306100	-0.000656
736.000000	143.827957	5.303316	-0.001179
737.000000	144.102570	5.302324	-0.000164
738.000000	144.298080	5.303354	-0.000393
739.000000	144.455002	5.305261	-0.000164
740.000000	144.602234	5.299882	-0.000629
741.000000	144.706665	5.303239	-0.000492
742.000000	144.947464	5.301218	-0.000301
743.000000	145.112808	5.302133	-0.000267
744.000000	145.299301	5.298929	-0.000130
745.000000	145.369919	5.303049	-0.000668
746.000000	145.628204	5.300111	-0.000141
747.000000	145.803833	5.298204	-0.001106
748.000000	145.890121	5.300645	-0.000553
749.000000	146.153259	5.297250	-0.000244
750.000000	146.193680	5.297822	-0.000038
751.000000	146.365067	5.298395	0.000557
752.000000	146.508713	5.299882	-0.000542

753.000000	146.666214	5.299005	-0.001514
754.000000	146.846069	5.294809	-0.002014
755.000000	147.122452	5.293359	-0.001316
756.000000	147.228073	5.292634	0.000153
757.000000	147.436890	5.293512	0.000744
758.000000	147.649918	5.295496	0.001221
759.000000	147.858124	5.295648	0.000050
760.000000	147.961349	5.297670	-0.000977
761.000000	148.167725	5.292673	-0.001175
762.000000	148.249207	5.292100	-0.001415
763.000000	148.477325	5.292558	-0.000328
764.000000	148.701233	5.290651	-0.000744
765.000000	148.756165	5.291757	-0.000691
766.000000	148.882278	5.288781	-0.000454
767.000000	149.117035	5.290040	-0.000420
768.000000	149.278183	5.289239	0.000198
769.000000	149.479736	5.289430	-0.000527
770.000000	149.657776	5.290078	-0.000343
771.000000	149.785706	5.286988	-0.001148
772.000000	149.983444	5.288743	-0.000782
773.000000	150.170502	5.284356	0.000248
774.000000	150.310028	5.287484	-0.000404
775.000000	150.540222	5.288857	0.000637
776.000000	150.714325	5.284471	-0.000744
777.000000	150.864609	5.289049	-0.001133
778.000000	151.051849	5.283669	-0.000759

779.000000	151.184219	5.283593	-0.001049
780.000000	151.317200	5.283402	0.000217
781.000000	151.512787	5.283937	-0.000637
782.000000	151.630249	5.284585	-0.001167
783.000000	151.779922	5.279817	-0.001030
784.000000	151.962982	5.279626	-0.000961
785.000000	152.160950	5.281266	0.000301
786.000000	152.357727	5.279054	-0.000042
787.000000	152.495483	5.281610	-0.001324
788.000000	152.701797	5.279244	-0.000736
789.000000	152.813309	5.274552	-0.000614
790.000000	153.008911	5.278901	-0.000172
791.000000	153.163330	5.278710	0.000374
792.000000	153.278442	5.276307	-0.000336
793.000000	153.496094	5.277719	-0.000866
794.000000	153.648132	5.277719	-0.000801
795.000000	153.811523	5.273675	-0.000782
796.000000	153.944519	5.274323	-0.000366
797.000000	154.121017	5.275506	0.000019
798.000000	154.343430	5.274972	-0.000748
799.000000	154.424545	5.273446	-0.000130
800.000000	154.577179	5.271615	0.000591
801.000000	154.766190	5.276536	0.000835
802.000000	154.932571	5.276383	-0.000465
803.000000	155.188980	5.275239	-0.001804
804.000000	155.357727	5.269937	-0.002449

805.000000	155.543198	5.270738	-0.001682
806.000000	155.735794	5.266389	0.000046
807.000000	155.840149	5.268601	-0.000607
808.000000	156.055420	5.271234	-0.000011
809.000000	156.188995	5.265283	-0.001072
810.000000	156.344025	5.267991	-0.002224
811.000000	156.530060	5.264863	-0.000599
812.000000	156.711334	5.260324	-0.000599
813.000000	156.843704	5.266122	0.000443
814.000000	157.039307	5.264367	0.001030
815.000000	157.269470	5.265054	-0.001034
816.000000	157.407227	5.266007	-0.000523
817.000000	157.620712	5.260133	-0.000477
818.000000	157.708359	5.264215	-0.000313
819.000000	157.923630	5.263566	0.000164
820.000000	158.061981	5.262727	-0.000935
821.000000	158.293335	5.261697	-0.000912
822.000000	158.468643	5.260476	-0.001141
823.000000	158.630829	5.260133	-0.000740
824.000000	158.711945	5.257806	-0.001408
825.000000	158.870560	5.259332	-0.000320
826.000000	159.166321	5.253839	-0.000179
827.000000	159.277222	5.260514	-0.000881
828.000000	159.510986	5.256318	-0.000851
829.000000	159.723862	5.253686	-0.001614
830.000000	159.898590	5.252999	-0.000690

831.000000	159.964050	5.254106	-0.000530
832.000000	160.094727	5.252656	-0.000374
833.000000	160.331314	5.251206	-0.000694
834.000000	160.520859	5.252580	-0.000298
835.000000	160.630310	5.250672	-0.001251
836.000000	160.781006	5.251435	-0.000462
837.000000	160.878723	5.245522	-0.000225
838.000000	160.934036	5.252847	-0.000134
839.000000	161.161804	5.248841	0.000061
840.000000	161.294846	5.249108	-0.001678
841.000000	161.511429	5.247697	-0.001469
842.000000	161.714478	5.245027	-0.000965
843.000000	161.916382	5.243539	-0.000378
844.000000	162.024689	5.246362	-0.000244
845.000000	162.190674	5.245141	-0.000954
846.000000	162.307190	5.243005	-0.001766
847.000000	162.563812	5.240449	-0.000854
848.000000	162.689758	5.239877	-0.000061
849.000000	162.788071	5.242433	0.000267
850.000000	163.024689	5.241708	-0.000317
851.000000	163.158295	5.240869	-0.001011
852.000000	163.337219	5.239076	-0.001194
853.000000	163.510254	5.238694	-0.000530
854.000000	163.708618	5.236825	-0.000599
855.000000	163.877518	5.239343	-0.000572
856.000000	163.989944	5.235757	-0.000610

857.000000	164.199478	5.236367	-0.000904
858.000000	164.342514	5.235261	-0.000824
859.000000	164.454926	5.235070	-0.001793
860.000000	164.771591	5.232285	-0.001068
861.000000	164.829865	5.228889	-0.000843
862.000000	165.012894	5.233010	-0.000397
863.000000	165.278961	5.230492	-0.000359
864.000000	165.416092	5.229500	-0.001049
865.000000	165.629745	5.228851	-0.001015
866.000000	165.646225	5.228584	-0.000587
867.000000	165.884583	5.225876	-0.000793
868.000000	166.067062	5.228050	-0.000797
869.000000	166.227142	5.225151	-0.001110
870.000000	166.433731	5.224960	-0.000999
871.000000	166.647400	5.221870	0.000256
872.000000	166.792191	5.224693	0.000179
873.000000	166.897568	5.226563	0.000629
874.000000	167.112366	5.223511	-0.001171
875.000000	167.284836	5.225609	-0.002045
876.000000	167.486710	5.219315	-0.002022
877.000000	167.562637	5.218437	-0.000942
878.000000	167.811600	5.216988	-0.000202
879.000000	167.940506	5.222061	-0.000950
880.000000	168.107666	5.216492	-0.000660
881.000000	168.267776	5.213936	-0.000996
882.000000	168.438446	5.217751	-0.000336

883.000000	168.581482	5.216454	-0.000542
884.000000	168.679184	5.213554	-0.000496
885.000000	168.912842	5.213326	-0.000320
886.000000	169.065872	5.216835	-0.000263
887.000000	169.224182	5.213211	-0.001301
888.000000	169.455505	5.212296	-0.002159
889.000000	169.553207	5.209091	-0.001671
890.000000	169.729218	5.208099	-0.001419
891.000000	169.853989	5.206955	-0.000084
892.000000	170.121155	5.206268	-0.000011
893.000000	170.191772	5.209587	-0.000595
894.000000	170.413513	5.206726	-0.001232
895.000000	170.476685	5.203751	-0.002140
896.000000	170.705276	5.203026	-0.001194
897.000000	170.819595	5.200737	-0.000702
898.000000	170.972382	5.202263	-0.000446
899.000000	171.219940	5.200623	-0.000233
900.000000	171.399704	5.200851	-0.000904
901.000000	171.547333	5.200279	-0.001186
902.000000	171.725403	5.197914	-0.001854
903.000000	171.885071	5.196159	-0.001198
904.000000	172.081512	5.193642	-0.000645
905.000000	172.218796	5.196426	-0.000572
906.000000	172.296326	5.194557	-0.000175
907.000000	172.572037	5.192841	-0.001152
908.000000	172.724823	5.194557	-0.001041

909.000000	172.890244	5.190666	-0.000427
910.000000	173.056259	5.190437	-0.000645
911.000000	173.206741	5.192764	-0.001488
912.000000	173.424408	5.190285	-0.000908
913.000000	173.520340	5.183304	-0.001560
914.000000	173.858643	5.190628	-0.001049
915.000000	173.911499	5.184792	-0.001190
916.000000	174.016602	5.184296	-0.001999
917.000000	174.199280	5.180519	-0.001099
918.000000	174.308960	5.182770	-0.001106
919.000000	174.542725	5.180061	-0.000858
920.000000	174.627747	5.178993	-0.001095
921.000000	174.874161	5.178116	-0.000751
922.000000	175.070023	5.178268	-0.000603
923.000000	175.160767	5.176666	-0.000973
924.000000	175.371002	5.176704	-0.000668
925.000000	175.483017	5.174034	-0.000107
926.000000	175.655319	5.176247	-0.000797
927.000000	175.874161	5.176361	-0.000607
928.000000	176.052795	5.171555	-0.001461
929.000000	176.209595	5.173347	-0.000797
930.000000	176.392258	5.170448	-0.000858
931.000000	176.548492	5.172928	-0.001968
932.000000	176.709320	5.167473	-0.001587
933.000000	176.854630	5.164993	-0.002266
934.000000	177.072311	5.166481	-0.000427

935.000000	177.256134	5.162094	-0.000229
936.000000	177.348602	5.166786	-0.001678
937.000000	177.478424	5.163696	-0.001415
938.000000	177.785141	5.157288	-0.002010
939.000000	177.889099	5.159767	-0.000637
940.000000	178.151596	5.158699	0.000446
941.000000	178.354919	5.159805	-0.000187
942.000000	178.484741	5.159500	-0.001343
943.000000	178.632339	5.158432	-0.002201
944.000000	178.888519	5.152672	-0.001713
945.000000	179.069458	5.152214	-0.001312
946.000000	179.250381	5.154045	-0.000172
947.000000	179.369278	5.151184	-0.000751
948.000000	179.512878	5.152328	-0.001751
949.000000	179.604797	5.149315	-0.001160
950.000000	179.910934	5.146225	-0.000813
951.000000	180.026993	5.148438	-0.001003
952.000000	180.151108	5.148705	-0.000759
953.000000	180.359131	5.143059	-0.002945
954.000000	180.540176	5.145119	-0.002983
955.000000	180.708008	5.135506	-0.001457
956.000000	180.814880	5.137566	-0.001152
957.000000	181.016617	5.139549	0.000450
958.000000	181.209717	5.137337	-0.000412
959.000000	181.421204	5.137871	-0.001862
960.000000	181.587296	5.136345	-0.001232

961.000000	181.813736	5.130737	-0.001801
962.000000	181.801666	5.134743	-0.001190
963.000000	182.074097	5.129669	-0.000488
964.000000	182.221802	5.130928	-0.001205
965.000000	182.443634	5.130203	-0.001293
966.000000	182.560867	5.128448	-0.001835
967.000000	182.844788	5.124443	-0.002556
968.000000	182.853973	5.124634	-0.001747
969.000000	183.028687	5.119331	-0.001297
970.000000	183.200516	5.122268	-0.001812
971.000000	183.433289	5.119140	-0.000912
972.000000	183.560303	5.115668	-0.000862
973.000000	183.643616	5.118072	-0.000290
974.000000	183.838455	5.118491	-0.000386
975.000000	184.001099	5.116278	-0.001278
976.000000	184.162033	5.114637	-0.001564
977.000000	184.340744	5.113607	-0.001873
978.000000	184.524094	5.112005	-0.001621
979.000000	184.697632	5.108228	-0.001507
980.000000	184.857422	5.109220	-0.000938
981.000000	185.001099	5.107465	-0.000496
982.000000	185.217773	5.107694	-0.001156
983.000000	185.391907	5.106512	-0.001621
984.000000	185.483871	5.103918	-0.001595
985.000000	185.643051	5.101247	-0.001911
986.000000	185.830414	5.102354	-0.001602

987.000000	185.910294	5.097738	-0.001621
988.000000	186.176956	5.097661	-0.001629
989.000000	186.271225	5.095487	-0.000965
990.000000	186.468353	5.095335	-0.002003
991.000000	186.666046	5.094076	-0.002094
992.000000	186.760864	5.088354	-0.001610
993.000000	186.925232	5.088506	-0.001331
994.000000	187.159714	5.090070	-0.001072
995.000000	187.222366	5.086561	-0.001328
996.000000	187.464325	5.083967	-0.001858
997.000000	187.658569	5.084920	-0.001411
998.000000	187.834442	5.081602	-0.001949
999.000000	187.920059	5.080686	-0.001991
1000.000000	188.102234	5.076337	-0.000988
1001.000000	188.297073	5.077596	-0.001392
1002.000000	188.392487	5.078207	-0.001030
1003.000000	188.602264	5.072790	-0.001156
1004.000000	188.867767	5.073591	-0.002163
1005.000000	188.867188	5.074125	-0.001831
1006.000000	189.066605	5.066724	-0.003002
1007.000000	189.264297	5.067068	-0.003071
1008.000000	189.342499	5.062109	-0.001369
1009.000000	189.653397	5.061079	-0.000664
1010.000000	189.803406	5.062871	0.000015
1011.000000	189.924576	5.063367	-0.000717
1012.000000	190.154892	5.061040	-0.001740

1013.000000	190.364319	5.058408	-0.002354
1014.000000	190.513565	5.056653	-0.001980
1015.000000	190.640137	5.053792	-0.001671
1016.000000	190.853531	5.053449	-0.002037
1017.000000	191.045929	5.051656	-0.002377
1018.000000	191.207092	5.047536	-0.002083
1019.000000	191.313217	5.044866	-0.001850
1020.000000	191.519821	5.046430	-0.001305
1021.000000	191.617981	5.042958	-0.001133
1022.000000	191.943756	5.041966	-0.002090
1023.000000	192.079956	5.041432	-0.002064
1024.000000	192.202576	5.036740	-0.002541
1025.000000	192.356339	5.035253	-0.002895
1026.000000	192.426758	5.032353	-0.002083
1027.000000	192.612335	5.029149	-0.002045
1028.000000	192.773483	5.029378	-0.001431
1029.000000	192.930725	5.026517	-0.000767
1030.000000	193.108917	5.026517	-0.001907
1031.000000	193.253662	5.026746	-0.002724
1032.000000	193.480652	5.019727	-0.003090
1033.000000	193.616272	5.016294	-0.001816
1034.000000	193.839340	5.016294	-0.000576
1035.000000	193.965897	5.019383	-0.001038
1036.000000	194.158325	5.015302	-0.001625
1037.000000	194.301315	5.011601	-0.001820
1038.000000	194.417084	5.012059	-0.001572

1039.000000	194.634460	5.011907	-0.002064
1040.000000	194.783722	5.007291	-0.002228
1041.000000	194.869995	5.003667	-0.002178
1042.000000	195.031723	5.005040	-0.001503
1043.000000	195.257065	5.002141	-0.001518
1044.000000	195.442627	5.000539	-0.001900
1045.000000	195.579987	4.998325	-0.001965
1046.000000	195.767822	4.997448	-0.002522
1047.000000	196.044800	4.993862	-0.002728
1048.000000	196.072601	4.990162	-0.002888
1049.000000	196.288818	4.988331	-0.002682
1050.000000	196.441483	4.985775	-0.001942
1051.000000	196.588455	4.982647	-0.001911
1052.000000	196.799072	4.983295	-0.002064
1053.000000	197.006195	4.980015	-0.001911
1054.000000	197.173050	4.976772	-0.002182
1055.000000	197.369965	4.976353	-0.001286
1056.000000	197.499954	4.974216	-0.001110
1057.000000	197.708801	4.974865	-0.002056
1058.000000	197.761597	4.971966	-0.002338
1059.000000	197.936401	4.967197	-0.003162
1060.000000	198.048767	4.966358	-0.002594
1061.000000	198.388153	4.961857	-0.001659
1062.000000	198.544220	4.961666	-0.001938
1063.000000	198.709366	4.961246	-0.001888
1064.000000	198.913101	4.956974	-0.002602

1065.000000	199.011307	4.954762	-0.002506
1066.000000	199.256470	4.951900	-0.001915
1067.000000	199.407440	4.951252	-0.002701
1068.000000	199.537964	4.949154	-0.002571
1069.000000	199.708801	4.942631	-0.002907
1070.000000	199.934509	4.943356	-0.002026
1071.000000	200.073898	4.939617	-0.000389
1072.000000	200.265228	4.940533	-0.001209
1073.000000	200.449768	4.942097	-0.001095
1074.000000	200.583252	4.936069	-0.002403
1075.000000	200.791870	4.936375	-0.003719
1076.000000	200.978088	4.931377	-0.003494
1077.000000	201.141861	4.925846	-0.003094
1078.000000	201.250656	4.923862	-0.001690
1079.000000	201.400391	4.924664	-0.001156
1080.000000	201.608490	4.923519	-0.001495
1081.000000	201.769440	4.920238	-0.002415
1082.000000	201.890030	4.918598	-0.002304
1083.000000	202.195679	4.915051	-0.002163
1084.000000	202.320190	4.914593	-0.001865
1085.000000	202.468811	4.911427	-0.002396
1086.000000	202.647736	4.911083	-0.002529
1087.000000	202.781784	4.904827	-0.002628
1088.000000	203.014526	4.905247	-0.003246
1089.000000	203.187866	4.901203	-0.002094
1090.000000	203.306168	4.896664	-0.002232

1091.000000	203.426758	4.898647	-0.000854
1092.000000	203.555756	4.895367	-0.001511
1093.000000	203.809265	4.897579	-0.003113
1094.000000	203.910233	4.889645	-0.003430
1095.000000	204.122223	4.885944	-0.003789
1096.000000	204.212524	4.884035	-0.001877
1097.000000	204.522110	4.881441	-0.001252
1098.000000	204.632034	4.882510	-0.001259
1099.000000	204.777283	4.880449	-0.001808
1100.000000	204.961807	4.878236	-0.002316
1101.000000	205.073410	4.874536	-0.002022
1102.000000	205.311783	4.873888	-0.002430
1103.000000	205.452560	4.872514	-0.002914
1104.000000	205.562469	4.867097	-0.002075
1105.000000	205.775040	4.863359	-0.001526
1106.000000	205.915817	4.868089	-0.000546
1107.000000	206.059952	4.864389	-0.000568
1108.000000	206.218140	4.863855	-0.002769
1109.000000	206.450317	4.862634	-0.002316
1110.000000	206.569229	4.855119	-0.002590
1111.000000	206.707169	4.857179	-0.001873
1112.000000	206.842896	4.853631	-0.000324
1113.000000	206.984253	4.854013	-0.001022
1114.000000	207.213623	4.855081	-0.001762
1115.000000	207.323547	4.851343	-0.001801
1116.000000	207.492935	4.846155	-0.002224

1117.000000	207.633713	4.849473	-0.001492
1118.000000	207.758789	4.844896	-0.001053
1119.000000	208.118286	4.844514	-0.001968
1120.000000	208.135635	4.843370	-0.001663
1121.000000	208.343750	4.840394	-0.001778
1122.000000	208.464325	4.838640	-0.001110
1123.000000	208.679123	4.837991	-0.001350
1124.000000	208.847946	4.839021	-0.001583
1125.000000	209.017334	4.833452	-0.001167
1126.000000	209.146866	4.832994	-0.001705
1127.000000	209.290451	4.835168	-0.000710
1128.000000	209.469376	4.829637	-0.001213
1129.000000	209.630341	4.831583	-0.002022
1130.000000	209.796356	4.828722	-0.001194
1131.000000	209.865845	4.825517	-0.002331
1132.000000	210.090347	4.826700	-0.001480
1133.000000	210.301117	4.820939	-0.000729
1134.000000	210.404297	4.823610	-0.000797
1135.000000	210.695450	4.823419	0.000046
1136.000000	210.815811	4.821474	-0.001095
1137.000000	211.078140	4.822237	-0.002014
1138.000000	211.118057	4.818727	-0.001698
1139.000000	211.283371	4.814721	-0.001305
1140.000000	211.506882	4.816743	0.000427
1141.000000	211.591736	4.816705	0.000359
1142.000000	211.857422	4.819871	-0.000008

1143.000000	211.931168	4.814950	-0.001579
1144.000000	212.181290	4.817582	-0.002361
1145.000000	212.329926	4.809953	-0.000809
1146.000000	212.463058	4.810563	-0.000652
1147.000000	212.607269	4.814416	0.000824
1148.000000	212.783630	4.812089	0.000118
1149.000000	212.989395	4.813310	-0.000008
1150.000000	213.196259	4.811707	0.000477
1151.000000	213.309418	4.814569	-0.000908
1152.000000	213.485245	4.813844	-0.001770
1153.000000	213.629440	4.807702	-0.001694
1154.000000	213.791397	4.806291	-0.001751
1155.000000	214.022705	4.809876	-0.000282
1156.000000	214.252853	4.804002	-0.000153
1157.000000	214.389297	4.807435	-0.000778
1158.000000	214.573975	4.806748	0.000244
1159.000000	214.755905	4.804612	0.000042
1160.000000	214.854095	4.806634	-0.000233
1161.000000	215.047653	4.807702	-0.000732
1162.000000	215.248962	4.804040	-0.000889
1163.000000	215.309982	4.802247	-0.000187
1164.000000	215.577286	4.804917	0.000202
1165.000000	215.613907	4.806329	0.000114
1166.000000	215.896240	4.803010	-0.000523
1167.000000	216.086487	4.803773	-0.001492
1168.000000	216.286682	4.803582	-0.000854

1169.000000	216.398163	4.798585	-0.000057
1170.000000	216.610031	4.801332	-0.000404
1171.000000	216.735367	4.804612	-0.000599
1172.000000	216.856323	4.798547	-0.001015
1173.000000	217.161911	4.796983	0.000000
1174.000000	217.333267	4.800073	0.001328
1175.000000	217.463058	4.803849	0.001060
1176.000000	217.681580	4.801751	-0.000408
1177.000000	217.834106	4.801446	-0.001442
1178.000000	218.036560	4.799233	-0.001568
1179.000000	218.254517	4.797898	-0.000645
1180.000000	218.485809	4.795686	0.000294
1181.000000	218.533493	4.799996	0.000252
1182.000000	218.664398	4.799653	-0.000130
1183.000000	218.927826	4.797174	-0.001144
1184.000000	219.058731	4.796449	-0.000839
1185.000000	219.194626	4.795877	-0.000515
1186.000000	219.374313	4.796105	-0.001034
1187.000000	219.574554	4.794770	-0.000305
1188.000000	219.686584	4.791833	-0.000835
1189.000000	219.845184	4.796487	-0.000267
1190.000000	220.023849	4.791070	-0.000084
1191.000000	220.167618	4.793817	-0.000793
1192.000000	220.374191	4.792748	0.000252
1193.000000	220.540741	4.791680	-0.000080
1194.000000	220.688721	4.793397	-0.000065

1195.000000	220.759949	4.793092	-0.000320
1196.000000	220.931442	4.791718	-0.000782
1197.000000	221.128174	4.790917	-0.000542
1198.000000	221.308975	4.790574	-0.000042
1199.000000	221.456924	4.790956	-0.000328
1200.000000	221.679947	4.791490	-0.001396
1201.000000	221.802673	4.788819	-0.001575
1202.000000	221.924896	4.784661	-0.000774
1203.000000	222.039368	4.786492	0.000504
1204.000000	222.177490	4.788781	0.000835
1205.000000	222.374725	4.789277	-0.000233
1206.000000	222.561035	4.787446	-0.000870
1207.000000	222.738037	4.785996	-0.000549
1208.000000	222.860764	4.786073	-0.000069
1209.000000	223.058594	4.787217	0.000385
1210.000000	223.184052	4.786492	0.000233
1211.000000	223.343506	4.787713	-0.000034
1212.000000	223.564865	4.786988	-0.000130
1213.000000	223.738571	4.786798	-0.001198
1214.000000	223.902405	4.786302	-0.000465
1215.000000	224.058029	4.782067	-0.000431
1216.000000	224.290894	4.787026	-0.000435
1217.000000	224.544617	4.784280	-0.000408
1218.000000	224.590622	4.783021	-0.000996
1219.000000	224.836121	4.782029	-0.000549
1220.000000	225.067886	4.783174	-0.000813

1221.000000	225.207611	4.781457	-0.000469
1222.000000	225.365417	4.779244	-0.000416
1223.000000	225.521042	4.781648	0.000908
1224.000000	225.688171	4.780999	0.000698
1225.000000	225.855835	4.785119	-0.000008
1226.000000	226.019653	4.780999	-0.000481
1227.000000	226.142960	4.781610	-0.001484
1228.000000	226.371994	4.780351	-0.001076
1229.000000	226.493652	4.778024	-0.001171
1230.000000	226.684326	4.777413	-0.000118
1231.000000	226.810349	4.777223	0.000076
1232.000000	226.997772	4.780160	0.000050
1233.000000	227.188446	4.777032	0.000080
1234.000000	227.317749	4.777757	-0.000706
1235.000000	227.513367	4.778825	-0.000980
1236.000000	227.690323	4.775735	-0.000507
1237.000000	227.802673	4.773141	-0.000614
1238.000000	227.976364	4.778062	0.000313
1239.000000	228.164886	4.774590	0.000507
1240.000000	228.307343	4.776574	-0.000374
1241.000000	228.506775	4.776422	0.000397
1242.000000	228.663498	4.775277	-0.000675
1243.000000	228.838303	4.777223	-0.000614
1244.000000	228.958832	4.772798	-0.001064
1245.000000	229.121597	4.774590	-0.000839
1246.000000	229.340210	4.771272	-0.000278

1247.000000	229.468964	4.773789	-0.000969
1248.000000	229.660202	4.771806	-0.000248
1249.000000	229.802673	4.769478	-0.000252
1250.000000	229.969788	4.772187	0.000134
1251.000000	230.106293	4.772340	-0.000267
1252.000000	230.208267	4.771043	-0.000690
1253.000000	230.434692	4.768716	-0.000034
1254.000000	230.551453	4.770547	-0.000439
1255.000000	230.755981	4.772416	-0.000057
1256.000000	230.926483	4.766998	0.000458
1257.000000	231.059128	4.770204	-0.000458
1258.000000	231.159470	4.773942	-0.000401
1259.000000	231.352997	4.766655	-0.000916
1260.000000	231.537231	4.766769	-0.001080
1261.000000	231.687988	4.769211	0.000164
1262.000000	231.703339	4.767265	0.000069
1263.000000	232.025146	4.767228	-0.000450
1264.000000	232.124405	4.768105	-0.001564
1265.000000	232.297623	4.766541	-0.000992
1266.000000	232.475800	4.759789	0.000095
1267.000000	232.673187	4.766427	-0.000031
1268.000000	232.888107	4.768640	0.000317
1269.000000	233.014206	4.761963	-0.001801
1270.000000	233.110657	4.763603	-0.000797
1271.000000	233.373291	4.759941	0.000824
1272.000000	233.482361	4.765664	0.000450

1273.000000	233.649063	4.765053	0.000450
1274.000000	233.866180	4.763298	-0.000305
1275.000000	234.021332	4.763374	0.000015
1276.000000	234.229095	4.764977	-0.000771
1277.000000	234.400696	4.764290	-0.000805
1278.000000	234.577209	4.758987	-0.000530
1279.000000	234.681946	4.762344	-0.000309
1280.000000	234.877686	4.763298	0.000015
1281.000000	235.059143	4.760590	-0.000633
1282.000000	235.167160	4.759941	-0.000843
1283.000000	235.361786	4.760857	0.000282
1284.000000	235.442383	4.758949	0.000195
1285.000000	235.675919	4.762497	-0.000931
1286.000000	235.930313	4.760094	-0.001099
1287.000000	236.038864	4.755630	-0.000267
1288.000000	236.203903	4.756889	0.000149
1289.000000	236.343689	4.762764	0.000496
1290.000000	236.443481	4.757271	-0.000690
1291.000000	236.693481	4.757919	-0.001431
1292.000000	236.839294	4.755859	-0.000595
1293.000000	236.946747	4.756317	-0.000656
1294.000000	237.201706	4.755096	-0.000465
1295.000000	237.379883	4.755020	-0.000710
1296.000000	237.487885	4.754181	-0.000225
1297.000000	237.691284	4.753227	-0.000069
1298.000000	237.914963	4.754868	0.000233

1299.000000	238.058044	4.754333	-0.000641
1300.000000	238.317917	4.754791	-0.000992
1301.000000	238.452805	4.750061	-0.000793
1302.000000	238.615051	4.752045	-0.000771
1303.000000	238.781189	4.751740	0.000622
1304.000000	238.956665	4.750099	0.000134
1305.000000	239.251587	4.754143	-0.000317
1306.000000	239.371643	4.751511	-0.000690
1307.000000	239.525726	4.749451	-0.001236
1308.000000	239.702805	4.748993	0.000267
1309.000000	239.947800	4.749222	0.000671
1310.000000	240.105026	4.752960	0.000206
1311.000000	240.197113	4.750824	-0.000900
1312.000000	240.413818	4.749222	-0.000965
1313.000000	240.530273	4.746590	-0.000134
1314.000000	240.802216	4.750252	0.000313
1315.000000	241.021606	4.749641	0.000755
1316.000000	241.164063	4.749260	-0.000626
1317.000000	241.268082	4.750862	-0.001427
1318.000000	241.431152	4.746513	-0.001362
1319.000000	241.593109	4.743881	-0.000996
1320.000000	241.690613	4.745941	0.000740
1321.000000	241.999390	4.746170	0.000763
1322.000000	242.128311	4.749069	-0.000263
1323.000000	242.299484	4.746132	-0.000587
1324.000000	242.384003	4.744644	-0.000778

1325.000000	242.555756	4.745445	-0.000439
1326.000000	242.809250	4.745522	-0.000996
1327.000000	242.880219	4.743500	-0.000488
1328.000000	243.074158	4.740639	-0.000423
1329.000000	243.269165	4.745445	-0.000492
1330.000000	243.346085	4.742432	-0.000538
1331.000000	243.577408	4.740143	-0.001732
1332.000000	243.742081	4.740601	0.000015
1333.000000	243.892700	4.737701	0.000458
1334.000000	244.092575	4.743729	0.000675
1335.000000	244.295700	4.740868	-0.000019
1336.000000	244.414322	4.742393	-0.001068
1337.000000	244.490204	4.738274	-0.001339
1338.000000	244.655411	4.739685	-0.000916
1339.000000	244.709564	4.735527	-0.000492
1340.000000	244.926285	4.739189	-0.001015
1341.000000	245.053558	4.736061	-0.000446
1342.000000	245.348770	4.734344	-0.000698
1343.000000	245.482056	4.735718	-0.000084
1344.000000	245.557343	4.735870	0.001091
1345.000000	245.738831	4.734879	0.000755
1346.000000	245.970154	4.740219	0.000740
1347.000000	246.136978	4.737320	-0.000256
1348.000000	246.378067	4.738350	-0.000965
1349.000000	246.503189	4.734535	-0.000355
1350.000000	246.649429	4.736786	-0.000713

1351.000000	246.844482	4.736328	-0.000145
1352.000000	246.999405	4.733887	-0.000961
1353.000000	247.157028	4.735260	-0.000870
1354.000000	247.354218	4.732513	0.000061
1355.000000	247.511856	4.732666	-0.000275
1356.000000	247.765915	4.735489	-0.000172
1357.000000	247.866638	4.732399	-0.000805
1358.000000	247.952805	4.731789	-0.000221
1359.000000	248.140793	4.730492	0.000538
1360.000000	248.365601	4.735336	-0.000175
1361.000000	248.447922	4.733315	-0.000011
1362.000000	248.604507	4.729500	-0.000599
1363.000000	248.847702	4.733353	-0.000450
1364.000000	248.967422	4.732323	0.000042
1365.000000	249.139694	4.729652	-0.001137
1366.000000	249.260513	4.731560	-0.001274
1367.000000	249.419205	4.728050	-0.001488
1368.000000	249.583389	4.726753	-0.001411
1369.000000	249.686295	4.724617	-0.000336
1370.000000	249.864502	4.726219	0.000141
1371.000000	250.036911	4.726639	0.001007
1372.000000	250.120529	4.726448	0.000740
1373.000000	250.333801	4.729538	-0.000301
1374.000000	250.575500	4.728470	-0.001236
1375.000000	250.750183	4.724121	-0.001938
1376.000000	250.845062	4.722977	-0.000595

1377.000000	251.099091	4.722595	0.000504
1378.000000	251.189102	4.726257	-0.000034
1379.000000	251.435638	4.724998	-0.000946
1380.000000	251.589966	4.721603	-0.001144
1381.000000	251.780197	4.720192	-0.000828
1382.000000	251.991882	4.722939	0.000549
1383.000000	252.114059	4.720192	0.001156
1384.000000	252.362198	4.724350	0.000340
1385.000000	252.454926	4.725266	0.000416
1386.000000	252.643570	4.722099	-0.001141
1387.000000	252.859512	4.723396	-0.001556
1388.000000	252.972061	4.719582	-0.001198
1389.000000	253.179489	4.718742	-0.000954
1390.000000	253.279678	4.718437	-0.000511
1391.000000	253.502579	4.719200	-0.000263
1392.000000	253.640869	4.716797	-0.000263
1393.000000	253.899216	4.718246	-0.000256
1394.000000	254.038544	4.717598	-0.000080
1395.000000	254.208420	4.717522	-0.000481
1396.000000	254.414185	4.716759	-0.000340
1397.000000	254.586227	4.716263	-0.000099
1398.000000	254.855759	4.716530	-0.000614
1399.000000	254.978485	4.717140	-0.000534
1400.000000	255.155334	4.713249	-0.000526
1401.000000	255.354706	4.715233	-0.000443
1402.000000	255.424362	4.714851	0.000515

1403.000000	255.622681	4.714127	-0.000885
1404.000000	255.746964	4.716377	-0.000599
1405.000000	255.991379	4.710045	-0.001301
1406.000000	256.229309	4.713898	-0.001232
1407.000000	256.304321	4.708862	-0.000393
1408.000000	256.550812	4.710808	-0.001549
1409.000000	256.731415	4.709625	-0.000290
1410.000000	256.894318	4.705772	-0.000450
1411.000000	257.058868	4.709930	0.000324
1412.000000	257.264130	4.708405	0.000526
1413.000000	257.473633	4.709930	-0.000763
1414.000000	257.617828	4.708405	0.000313
1415.000000	257.795197	4.706116	-0.000668
1416.000000	257.910400	4.711876	0.000103
1417.000000	258.058868	4.704857	0.000240
1418.000000	258.223938	4.709549	-0.000980
1419.000000	258.304321	4.708481	-0.000401
1420.000000	258.472046	4.705162	-0.000587
1421.000000	258.650513	4.705048	-0.000851
1422.000000	258.854126	4.708328	-0.000004
1423.000000	258.970978	4.702644	-0.000610
1424.000000	259.208374	4.706345	-0.001858
1425.000000	259.333801	4.702988	-0.000999
1426.000000	259.475220	4.698868	-0.000809
1427.000000	259.576019	4.701385	-0.000401
1428.000000	259.764130	4.703102	-0.000122

1429.000000	259.846130	4.698868	0.000061
1430.000000	260.022400	4.699516	-0.000607
1431.000000	260.243073	4.703484	-0.000244
1432.000000	260.293457	4.697762	-0.001297
1433.000000	260.465363	4.698524	-0.001011
1434.000000	260.578400	4.695511	0.000195
1435.000000	260.703705	4.699554	0.001247
1436.000000	260.915283	4.698219	0.001347
1437.000000	261.019745	4.703407	-0.000103
1438.000000	261.193268	4.700317	-0.000763
1439.000000	261.381805	4.697990	-0.002197
1440.000000	261.538208	4.697113	-0.001472
1441.000000	261.616425	4.694023	-0.000118
1442.000000	261.890106	4.694939	-0.000301
1443.000000	262.019745	4.698486	-0.000500
1444.000000	262.190063	4.693375	-0.001854
1445.000000	262.398407	4.692307	-0.001263
1446.000000	262.482513	4.688759	0.000530
1447.000000	262.714447	4.694481	-0.000259
1448.000000	262.890137	4.694939	0.000305
1449.000000	263.062592	4.687920	-0.000648
1450.000000	263.286469	4.693565	-0.000805
1451.000000	263.506104	4.691925	-0.000076
1452.000000	263.604645	4.688911	-0.001068
1453.000000	263.781921	4.689865	-0.000416
1454.000000	263.939941	4.689255	-0.000683

1455.000000	264.115601	4.689674	-0.000740
1456.000000	264.334686	4.685593	-0.001015
1457.000000	264.548401	4.687996	-0.001644
1458.000000	264.741241	4.685020	-0.000793
1459.000000	264.854767	4.681740	-0.001041
1460.000000	265.066864	4.684753	0.000347
1461.000000	265.281647	4.682922	0.001278
1462.000000	265.436432	4.686165	0.000374
1463.000000	265.608917	4.687424	0.000118
1464.000000	265.713379	4.684372	-0.001644
1465.000000	265.949036	4.684410	-0.001812
1466.000000	266.088318	4.679451	-0.000687
1467.000000	266.307892	4.680824	-0.000038
1468.000000	266.416626	4.682732	0.000526
1469.000000	266.563385	4.682579	-0.000427
1470.000000	266.713898	4.681206	-0.001114
1471.000000	266.877808	4.679451	-0.000568
1472.000000	267.049194	4.678726	-0.000931
1473.000000	267.162231	4.680977	-0.000126
1474.000000	267.376465	4.675789	-0.000313
1475.000000	267.481445	4.680290	-0.001850
1476.000000	267.626038	4.677505	-0.000103
1477.000000	267.782990	4.670868	-0.000568
1478.000000	267.905640	4.679985	0.000092
1479.000000	268.048126	4.676208	0.000828
1480.000000	268.285400	4.675293	-0.001228

1481.000000	268.417175	4.677353	-0.000744
1482.000000	268.585876	4.673271	-0.001331
1483.000000	268.724091	4.673500	-0.001614
1484.000000	268.956543	4.670563	-0.001789
1485.000000	269.126343	4.670639	-0.000916
1486.000000	269.303619	4.665756	0.000336
1487.000000	269.415009	4.671326	0.000324
1488.000000	269.638916	4.671898	0.001053
1489.000000	269.783539	4.669189	-0.000011
1490.000000	269.842987	4.672089	-0.000488
1491.000000	270.026855	4.671173	-0.000309
1492.000000	270.261719	4.668465	-0.001415
1493.000000	270.414307	4.669456	-0.001282
1494.000000	270.554199	4.665871	-0.001080
1495.000000	270.782104	4.666061	-0.001690
1496.000000	270.946899	4.664764	-0.000610
1497.000000	271.041260	4.661560	-0.000801
1498.000000	271.297729	4.665070	-0.000942
1499.000000	271.498596	4.661903	0.000084
1500.000000	271.656525	4.659882	-0.000732
1501.000000	271.801178	4.664574	-0.000362
1502.000000	271.985596	4.660072	0.000122
1503.000000	272.182251	4.659996	-0.000523
1504.000000	272.280762	4.662781	0.000610
1505.000000	272.473694	4.660606	-0.000050
1506.000000	272.612549	4.662819	-0.000629

1507.000000	272.772064	4.659729	-0.000427
1508.000000	272.951691	4.660072	-0.000836
1509.000000	273.142487	4.659843	-0.000935
1510.000000	273.258514	4.658584	-0.001160
1511.000000	273.393677	4.655799	-0.001442
1512.000000	273.600372	4.656296	-0.000523
1513.000000	273.708466	4.653777	-0.001145
1514.000000	273.918823	4.656982	-0.001236
1515.000000	274.171631	4.649734	-0.001202
1516.000000	274.250061	4.652136	-0.000717
1517.000000	274.465210	4.650190	0.000958
1518.000000	274.651733	4.653167	-0.000389
1519.000000	274.799072	4.654006	-0.000908
1520.000000	274.991394	4.648283	-0.001339
1521.000000	275.170532	4.648092	-0.001152
1522.000000	275.324768	4.649428	-0.000530
1523.000000	275.462524	4.647672	0.000199
1524.000000	275.692566	4.645842	-0.000175
1525.000000	275.808075	4.650878	-0.000935
1526.000000	275.969208	4.646948	-0.000805
1527.000000	276.094238	4.642446	-0.002121
1528.000000	276.274933	4.646032	-0.000416
1529.000000	276.548431	4.640729	0.000000
1530.000000	276.670837	4.645727	-0.000912
1531.000000	276.765137	4.642599	-0.000778
1532.000000	277.019531	4.640539	-0.001728

1533.000000	277.269653	4.639432	-0.001354
1534.000000	277.385162	4.638669	-0.001495
1535.000000	277.528290	4.636762	-0.001099
1536.000000	277.723267	4.634397	-0.001053
1537.000000	277.933655	4.636075	-0.000134
1538.000000	278.011597	4.633749	-0.000301
1539.000000	278.225128	4.636419	-0.000591
1540.000000	278.389435	4.632719	0.000256
1541.000000	278.555817	4.633634	0.000477
1542.000000	278.830322	4.636419	-0.000225
1543.000000	278.971313	4.636953	-0.001472
1544.000000	279.103790	4.629934	-0.002483
1545.000000	279.310455	4.629514	-0.001289
1546.000000	279.472626	4.627721	0.000065
1547.000000	279.569092	4.631612	-0.000332
1548.000000	279.748169	4.629209	-0.000473
1549.000000	280.028320	4.627111	-0.001190
1550.000000	280.185974	4.627607	-0.001530
1551.000000	280.279266	4.626462	-0.001411
1552.000000	280.460602	4.621885	-0.001705
1553.000000	280.597412	4.622915	-0.000874
1554.000000	280.844269	4.620855	-0.000481
1555.000000	281.035034	4.622610	0.000435
1556.000000	281.240479	4.619634	-0.000088
1557.000000	281.318573	4.625700	-0.001122
1558.000000	281.565430	4.618871	-0.001190

1559.000000	281.637238	4.617383	-0.001751
1560.000000	281.852661	4.617841	-0.000084
1561.000000	281.999908	4.617460	-0.000416
1562.000000	282.152954	4.618413	-0.002083
1563.000000	282.348969	4.615018	-0.001228
1564.000000	282.526123	4.608648	-0.000912
1565.000000	282.691742	4.616201	0.000042
1566.000000	282.822266	4.613264	0.000038
1567.000000	282.985779	4.612920	-0.001011
1568.000000	283.190704	4.610479	-0.000786
1569.000000	283.318085	4.612539	-0.000912
1570.000000	283.485229	4.609525	-0.000629
1571.000000	283.636719	4.608839	-0.000919
1572.000000	283.699615	4.609182	-0.001049
1573.000000	283.894043	4.608114	-0.001301
1574.000000	284.051788	4.604642	-0.001503
1575.000000	284.137238	4.604604	-0.001625
1576.000000	284.369965	4.603422	-0.000065
1577.000000	284.564392	4.600599	0.000053
1578.000000	284.688080	4.606321	0.000008
1579.000000	284.857361	4.603422	-0.000534
1580.000000	285.005157	4.602048	-0.001839
1581.000000	285.166595	4.600065	-0.001301
1582.000000	285.240479	4.598806	-0.000999
1583.000000	285.460602	4.598539	-0.001305
1584.000000	285.622559	4.597814	-0.001816

1585.000000	285.817505	4.594038	-0.001286
1586.000000	285.996246	4.591977	-0.000694
1587.000000	286.145081	4.595029	-0.000050
1588.000000	286.275085	4.593847	-0.000355
1589.000000	286.543427	4.592854	-0.001179
1590.000000	286.567535	4.591290	-0.001541
1591.000000	286.841095	4.590413	-0.001209
1592.000000	287.004120	4.587361	-0.001186
1593.000000	287.086914	4.588773	-0.000736
1594.000000	287.235779	4.586179	-0.000221
1595.000000	287.447510	4.587323	-0.000843
1596.000000	287.547119	4.586980	-0.000401
1597.000000	287.729980	4.584157	-0.001022
1598.000000	287.935944	4.585759	-0.001286
1599.000000	288.051270	4.582822	-0.001125
1600.000000	288.253571	4.581220	-0.001957
1601.000000	288.444336	4.580800	-0.001041
1602.000000	288.624634	4.576985	-0.000732
1603.000000	288.809174	4.579732	-0.001087
1604.000000	288.989441	4.578092	-0.001057
1605.000000	289.044983	4.574811	-0.001347
1606.000000	289.259399	4.574162	-0.001785
1607.000000	289.419220	4.574964	-0.001194
1608.000000	289.535553	4.569089	-0.000343
1609.000000	289.830109	4.571378	-0.000675
1610.000000	289.990997	4.574239	0.000183

1611.000000	290.178040	4.569013	-0.000252
1612.000000	290.319977	4.571187	-0.000591
1613.000000	290.487061	4.571645	-0.000481
1614.000000	290.678314	4.569967	-0.001671
1615.000000	290.799805	4.567220	-0.001926
1616.000000	290.983154	4.565046	-0.001389
1617.000000	291.170166	4.564474	-0.001057
1618.000000	291.292236	4.564397	-0.000641
1619.000000	291.560944	4.562261	-0.002365
1620.000000	291.672485	4.562948	-0.001972
1621.000000	291.800812	4.553373	-0.001167
1622.000000	291.945435	4.558980	0.000000
1623.000000	292.187469	4.558408	0.000511
1624.000000	292.406372	4.560430	-0.001160
1625.000000	292.552002	4.555204	-0.000854
1626.000000	292.655212	4.554784	-0.002075
1627.000000	292.870514	4.556959	-0.001141
1628.000000	293.120392	4.549177	-0.001152
1629.000000	293.249756	4.552305	-0.001438
1630.000000	293.365601	4.551351	-0.000759
1631.000000	293.553589	4.548681	-0.002087
1632.000000	293.739044	4.547193	-0.001595
1633.000000	293.895172	4.543951	-0.000771
1634.000000	294.182739	4.545743	-0.000443
1635.000000	294.265503	4.545553	-0.000446
1636.000000	294.512756	4.544179	-0.001648

1637.000000	294.656311	4.542501	-0.001133
1638.000000	294.744263	4.539029	-0.000950
1639.000000	295.058075	4.542463	-0.000576
1640.000000	295.186890	4.539449	-0.000900
1641.000000	295.300079	4.539411	-0.002255
1642.000000	295.566681	4.536054	-0.001209
1643.000000	295.636353	4.532887	-0.000435
1644.000000	295.798248	4.536664	-0.000069
1645.000000	296.008301	4.536931	-0.000351
1646.000000	296.116211	4.533688	-0.001858
1647.000000	296.313690	4.532620	-0.001614
1648.000000	296.452484	4.529530	-0.001755
1649.000000	296.611206	4.530941	-0.001270
1650.000000	296.807129	4.525753	-0.000622
1651.000000	296.943848	4.528156	-0.000618
1652.000000	297.166504	4.527813	0.000111
1653.000000	297.286438	4.526821	-0.001427
1654.000000	297.501740	4.526974	-0.001888
1655.000000	297.651062	4.521442	-0.001644
1656.000000	297.770477	4.521061	-0.001873
1657.000000	297.953827	4.521557	-0.000885
1658.000000	298.095276	4.517551	-0.001095
1659.000000	298.322052	4.518772	-0.001354
1660.000000	298.541565	4.516979	-0.000439
1661.000000	298.678253	4.515072	-0.001259
1662.000000	298.855865	4.517208	-0.001850

1663.000000	298.930237	4.512363	-0.001148
1664.000000	299.166992	4.509083	-0.001637
1665.000000	299.354553	4.513393	0.000126
1666.000000	299.466125	4.508511	0.000172
1667.000000	299.657837	4.513279	-0.000645
1668.000000	299.837524	4.509998	-0.000317
1669.000000	299.905609	4.509426	-0.000916
1670.000000	300.108917	4.508854	-0.000557
1671.000000	300.235748	4.509274	-0.000385
1672.000000	300.404022	4.507290	-0.001701
1673.000000	300.546021	4.508282	-0.002361
1674.000000	300.711121	4.500843	-0.001766
1675.000000	300.814423	4.500690	-0.001598
1676.000000	301.058624	4.502254	-0.000095
1677.000000	301.203278	4.499584	-0.000881
1678.000000	301.445953	4.500919	-0.002338
1679.000000	301.530304	4.496952	-0.002300
1680.000000	301.671295	4.491879	-0.001892
1681.000000	301.807007	4.492603	-0.001053
1682.000000	302.007263	4.493633	-0.000187
1683.000000	302.210083	4.490810	-0.000515
1684.000000	302.343201	4.491840	-0.000408
1685.000000	302.489410	4.490925	-0.000351
1686.000000	302.685974	4.491535	-0.001362
1687.000000	302.785553	4.489208	-0.001831
1688.000000	302.951691	4.485889	-0.001644

1689.000000	303.143555	4.484592	-0.000942
1690.000000	303.401398	4.485622	-0.001003
1691.000000	303.623596	4.484631	-0.000538
1692.000000	303.731567	4.480854	-0.000660
1693.000000	303.845825	4.484287	-0.000729
1694.000000	304.051819	4.482494	-0.001022
1695.000000	304.225800	4.480167	-0.001087
1696.000000	304.372559	4.477802	-0.000088
1697.000000	304.587952	4.481197	-0.000443
1698.000000	304.716370	4.481541	-0.000664
1699.000000	304.757263	4.476086	-0.001839
1700.000000	304.956909	4.477039	-0.002182
1701.000000	305.180725	4.474255	-0.001518
1702.000000	305.269836	4.471546	-0.001110
1703.000000	305.477386	4.471241	-0.000420
1704.000000	305.740479	4.472996	0.000286
1705.000000	305.913452	4.471432	-0.000965
1706.000000	306.149811	4.472881	-0.001991
1707.000000	306.185944	4.466473	-0.001812
1708.000000	306.482635	4.465519	-0.001461
1709.000000	306.563324	4.466053	-0.000065
1710.000000	306.737854	4.465786	-0.000637
1711.000000	306.878326	4.466015	-0.001320
1712.000000	307.083771	4.462353	-0.001568
1713.000000	307.203796	4.461170	-0.001221
1714.000000	307.454346	4.460369	-0.000645

1715.000000	307.547119	4.460903	-0.000980
1716.000000	307.806519	4.459263	-0.001408
1717.000000	307.895111	4.456821	-0.000931
1718.000000	308.106323	4.455372	-0.000053
1719.000000	308.320160	4.458195	0.000057
1720.000000	308.528748	4.458309	-0.000103
1721.000000	308.702209	4.455639	-0.001568
1722.000000	308.836395	4.456135	-0.001862
1723.000000	309.036102	4.451443	-0.001976
1724.000000	309.178619	4.451099	-0.001469
1725.000000	309.301758	4.448277	-0.000866
1726.000000	309.535553	4.450375	-0.001026
1727.000000	309.687500	4.447475	-0.001026
1728.000000	309.939575	4.446369	-0.001118
1729.000000	310.101990	4.445148	0.000278
1730.000000	310.240845	4.445950	0.000134
1731.000000	310.433594	4.449078	-0.000355
1732.000000	310.604095	4.445072	-0.001801
1733.000000	310.705078	4.443813	-0.001907
1734.000000	310.881775	4.439579	-0.001480
1735.000000	310.981781	4.442287	-0.000580
1736.000000	311.189026	4.438435	-0.001232
1737.000000	311.363129	4.441486	-0.001347
1738.000000	311.467773	4.433819	-0.001194
1739.000000	311.608215	4.437862	-0.001503
1740.000000	311.834595	4.434277	-0.000084

1741.000000	311.937225	4.433743	-0.001553
1742.000000	312.160522	4.435459	-0.001026
1743.000000	312.153290	4.429508	-0.001442
1744.000000	312.344482	4.431263	-0.001835
1745.000000	312.510803	4.428631	-0.001331
1746.000000	312.553253	4.426723	-0.001244
1747.000000	312.717010	4.425121	-0.000259
1748.000000	312.916504	4.426800	0.000202
1749.000000	313.017548	4.427296	0.000191
1750.000000	313.174500	4.426647	-0.000961
1751.000000	313.447021	4.426151	-0.000790
1752.000000	313.612335	4.422565	-0.000893
1753.000000	313.707153	4.425388	-0.001450
1754.000000	313.883301	4.422565	-0.000748
1755.000000	314.023743	4.418902	-0.001667
1756.000000	314.207703	4.422069	-0.000839
1757.000000	314.346039	4.417299	-0.001038
1758.000000	314.578156	4.419169	-0.001595
1759.000000	314.713898	4.415163	-0.000340
1760.000000	314.923737	4.415163	-0.001229
1761.000000	315.101990	4.417604	-0.000381
1762.000000	315.285919	4.411806	-0.000923
1763.000000	315.506134	4.414934	-0.002029
1764.000000	315.665192	4.411882	-0.001606
1765.000000	315.746552	4.407419	-0.001831
1766.000000	315.968323	4.407534	-0.001366

1767.000000	316.118073	4.407953	-0.000946
1768.000000	316.299927	4.404787	-0.000687
1769.000000	316.435638	4.404062	-0.000446
1770.000000	316.588501	4.406046	0.000027
1771.000000	316.859985	4.405092	-0.000080
1772.000000	317.002014	4.404406	-0.000854
1773.000000	317.260529	4.404482	-0.001469
1774.000000	317.458466	4.402079	-0.001694
1775.000000	317.542389	4.398912	-0.001320
1776.000000	317.742920	4.398722	-0.000660
1777.000000	317.961578	4.399561	-0.000179
1778.000000	318.199951	4.398455	-0.000378
1779.000000	318.351746	4.398149	-0.000328
1780.000000	318.602509	4.397539	0.000275
1781.000000	318.684875	4.398378	-0.000385
1782.000000	318.901978	4.399714	-0.000454
1783.000000	319.072998	4.395136	-0.001389
1784.000000	319.243439	4.396891	-0.001762
1785.000000	319.435181	4.392847	-0.001171
1786.000000	319.605621	4.392046	-0.001953
1787.000000	319.842407	4.391703	-0.001335
1788.000000	319.987793	4.387697	-0.001076
1789.000000	320.153809	4.388346	-0.000858
1790.000000	320.275818	4.388346	-0.000645
1791.000000	320.481964	4.387087	-0.000431
1792.000000	320.656311	4.385103	-0.000408

1793.000000	320.797333	4.387812	-0.000717
1794.000000	320.897308	4.385942	-0.000725
1795.000000	321.083008	4.383081	-0.001438
1796.000000	321.254791	4.383844	-0.001713
1797.000000	321.397858	4.381670	-0.001568
1798.000000	321.576324	4.378084	-0.001965
1799.000000	321.691162	4.378122	-0.000694
1800.000000	321.858337	4.375795	-0.000916
1801.000000	321.898315	4.379343	-0.000198
1802.000000	322.064056	4.372896	0.000282
1803.000000	322.196350	4.378580	-0.001049
1804.000000	322.419403	4.377588	-0.000965
1805.000000	322.539368	4.371752	-0.002419
1806.000000	322.693237	4.371485	-0.001160
1807.000000	322.849121	4.369539	0.000042
1808.000000	322.971741	4.372896	-0.000553
1809.000000	323.183533	4.371256	-0.000420
1810.000000	323.316315	4.367861	-0.002106
1811.000000	323.507599	4.369959	-0.001804
1812.000000	323.670166	4.363016	-0.000782
1813.000000	323.818848	4.364656	-0.001038
1814.000000	323.998413	4.366602	0.000046
1815.000000	324.108673	4.362978	0.000023
1816.000000	324.231720	4.364084	-0.000862
1817.000000	324.331696	4.366030	-0.000332
1818.000000	324.500916	4.360765	-0.000404

1819.000000	324.676300	4.362978	-0.001679
1820.000000	324.829620	4.363588	-0.001400
1821.000000	324.985596	4.356225	-0.001629
1822.000000	325.160980	4.357141	-0.000519
1823.000000	325.322510	4.358057	0.000881
1824.000000	325.492737	4.360078	0.000187
1825.000000	325.570679	4.359163	-0.000309
1826.000000	325.691681	4.357522	-0.002335
1827.000000	325.888092	4.357790	-0.001637
1828.000000	326.135345	4.349092	-0.001060
1829.000000	326.207123	4.355196	-0.001007
1830.000000	326.411713	4.353517	-0.000298
1831.000000	326.569672	4.350542	-0.002480
1832.000000	326.772705	4.349931	-0.001526
1833.000000	326.879395	4.344591	-0.000687
1834.000000	327.105591	4.348863	-0.000351
1835.000000	327.223511	4.347642	-0.000168
1836.000000	327.361969	4.346651	-0.000904
1837.000000	327.587616	4.344858	-0.000961
1838.000000	327.739899	4.345735	-0.001133
1839.000000	327.899872	4.343294	-0.001480
1840.000000	328.096893	4.341768	-0.001385
1841.000000	328.273254	4.339441	-0.000278
1842.000000	328.466583	4.340738	-0.000458
1843.000000	328.672729	4.342416	-0.000656
1844.000000	328.831177	4.337991	-0.001480

1845.000000	329.023560	4.337533	-0.001534
1846.000000	329.238403	4.335779	-0.000343
1847.000000	329.429657	4.335855	-0.000515
1848.000000	329.518341	4.337114	-0.000095
1849.000000	329.692230	4.334291	-0.001202
1850.000000	329.869629	4.336084	-0.001614
1851.000000	330.115845	4.330362	-0.000889
1852.000000	330.287598	4.331010	-0.000935
1853.000000	330.427094	4.332384	-0.000267
1854.000000	330.656830	4.330400	-0.000206
1855.000000	330.795776	4.329332	-0.001118
1856.000000	331.001465	4.331506	-0.001125
1857.000000	331.187622	4.326242	-0.000984
1858.000000	331.433228	4.326318	-0.001503
1859.000000	331.591187	4.327005	-0.000790
1860.000000	331.853210	4.323610	-0.000771
1861.000000	331.978882	4.323648	-0.000999
1862.000000	332.168152	4.324144	-0.000481
1863.000000	332.380951	4.321741	-0.000702
1864.000000	332.535309	4.322160	-0.001083
1865.000000	332.644531	4.321130	-0.001198
1866.000000	332.853210	4.319032	-0.001518
1867.000000	333.035858	4.317316	-0.001163
1868.000000	333.206085	4.316476	-0.000500
1869.000000	333.329163	4.316591	-0.000713
1870.000000	333.423492	4.316896	-0.001060

1871.000000	333.676300	4.313539	-0.001320
1872.000000	333.854736	4.312700	-0.001122
1873.000000	333.984039	4.312089	-0.000137
1874.000000	334.156860	4.312013	-0.000065
1875.000000	334.369141	4.313196	-0.001057
1876.000000	334.526062	4.311822	-0.001431
1877.000000	334.680420	4.306901	-0.002694
1878.000000	334.865021	4.308007	-0.001740
1879.000000	335.108673	4.301636	-0.000500
1880.000000	335.232239	4.305757	-0.001122
1881.000000	335.343506	4.305528	-0.000454
1882.000000	335.491180	4.300454	-0.000855
1883.000000	335.715820	4.302018	-0.000378
1884.000000	335.923462	4.303239	0.000137
1885.000000	336.036865	4.302247	0.000175
1886.000000	336.133789	4.301025	-0.001290
1887.000000	336.222504	4.304002	-0.001457
1888.000000	336.315308	4.295914	-0.000809
1889.000000	336.486572	4.297516	-0.000706
1890.000000	336.698364	4.300224	0.000370
1891.000000	336.864990	4.298317	-0.000217
1892.000000	337.022522	4.297363	-0.001358
1893.000000	337.153809	4.297859	-0.001774
1894.000000	337.389160	4.293663	-0.002060
1895.000000	337.489136	4.291298	-0.001591
1896.000000	337.702972	4.290344	-0.000706

1897.000000	337.911133	4.291565	0.000118
1898.000000	338.031250	4.290001	0.000607
1899.000000	338.137390	4.292061	0.000244
1900.000000	338.409668	4.293129	0.000053
1901.000000	338.522491	4.291222	-0.001465
1902.000000	338.608124	4.290688	-0.002914
1903.000000	338.871185	4.285957	-0.001568
1904.000000	339.043549	4.281189	-0.001141
1905.000000	339.221985	4.288132	-0.000553
1906.000000	339.318878	4.283897	0.000484
1907.000000	339.472198	4.281837	-0.001133
1908.000000	339.543518	4.286758	-0.001591
1909.000000	339.773712	4.281036	-0.001316
1910.000000	339.883972	4.276344	-0.001659
1911.000000	340.019531	4.280464	0.000462
1912.000000	340.241302	4.278748	0.000359
1913.000000	340.358429	4.282143	-0.000046
1914.000000	340.567322	4.277298	-0.000416
1915.000000	340.644928	4.280960	-0.002071
1916.000000	340.774780	4.277260	-0.002335
1917.000000	340.912720	4.271805	-0.002346
1918.000000	341.217987	4.270203	-0.000645
1919.000000	341.320374	4.272758	0.000084
1920.000000	341.449707	4.273560	0.000847
1921.000000	341.678894	4.270546	0.000099
1922.000000	341.818359	4.275543	-0.001331

1923.000000	342.096283	4.272263	-0.002087
1924.000000	342.181427	4.266045	-0.001968
1925.000000	342.408142	4.264862	-0.000931
1926.000000	342.579010	4.269402	-0.000542
1927.000000	342.768646	4.265930	-0.000935
1928.000000	342.951202	4.262802	-0.001236
1929.000000	343.119598	4.263489	-0.000774
1930.000000	343.264618	4.264442	-0.000683
1931.000000	343.479126	4.261238	-0.000652
1932.000000	343.639343	4.260513	-0.001896
1933.000000	343.888824	4.262192	-0.000999
1934.000000	344.055664	4.254486	-0.000660
1935.000000	344.259521	4.259254	-0.001015
1936.000000	344.419312	4.258682	0.000576
1937.000000	344.594727	4.255020	-0.000416
1938.000000	344.756470	4.259483	-0.000954
1939.000000	344.828979	4.256775	-0.001026
1940.000000	345.025757	4.253036	-0.002167
1941.000000	345.214935	4.253113	-0.000835
1942.000000	345.350830	4.250481	-0.000198
1943.000000	345.467468	4.253876	-0.000423
1944.000000	345.713867	4.251663	-0.000614
1945.000000	345.787415	4.250404	-0.001060
1946.000000	345.956818	4.249146	-0.000912
1947.000000	346.121613	4.249832	-0.000771
1948.000000	346.289917	4.247391	-0.000278

1949.000000	346.460358	4.247429	-0.000195
1950.000000	346.698212	4.248955	-0.000614
1951.000000	346.817841	4.248077	-0.000931
1952.000000	346.978577	4.243996	-0.001030
1953.000000	347.148987	4.245255	-0.000542
1954.000000	347.285400	4.245216	-0.000385
1955.000000	347.544556	4.244759	-0.000458
1956.000000	347.673340	4.242317	-0.001308
1957.000000	347.848267	4.244415	-0.001217
1958.000000	347.942596	4.238846	-0.001316
1959.000000	348.221985	4.240410	-0.001316
1960.000000	348.321411	4.237740	-0.000431
1961.000000	348.459351	4.238388	-0.001316
1962.000000	348.632751	4.237701	-0.000782
1963.000000	348.858917	4.233849	-0.001251
1964.000000	349.018127	4.236099	-0.001015
1965.000000	349.301117	4.232933	-0.000355
1966.000000	349.458313	4.233086	-0.000668
1967.000000	349.517151	4.233582	-0.001431
1968.000000	349.657104	4.232437	-0.001884
1969.000000	349.863007	4.226105	-0.001373
1970.000000	350.013092	4.227402	-0.000473
1971.000000	350.138397	4.229233	0.000446
1972.000000	350.421997	4.228508	0.000397
1973.000000	350.474274	4.227783	-0.000092
1974.000000	350.638184	4.230110	-0.000713

1975.000000	350.836548	4.227974	-0.000580
1976.000000	351.025269	4.224846	-0.001038
1977.000000	351.171936	4.227516	-0.000969
1978.000000	351.467651	4.225151	-0.000866
1979.000000	351.563599	4.222977	-0.000954
1980.000000	351.713745	4.222786	-0.000614
1981.000000	351.828430	4.223930	-0.000729
1982.000000	352.011566	4.221603	-0.000660
1983.000000	352.172394	4.219925	-0.000629
1984.000000	352.337830	4.221489	-0.001007
1985.000000	352.588959	4.220840	-0.001553
1986.000000	352.715271	4.216110	-0.001202
1987.000000	352.853271	4.214851	-0.000854
1988.000000	353.017151	4.218475	-0.000885
1989.000000	353.213470	4.215385	-0.000912
1990.000000	353.422028	4.211418	-0.001148
1991.000000	353.588928	4.213821	-0.000782
1992.000000	353.744202	4.213516	-0.000965
1993.000000	353.877106	4.210426	-0.001698
1994.000000	354.068909	4.208290	-0.000935
1995.000000	354.213501	4.207947	-0.000263
1996.000000	354.406281	4.210083	0.000118
1997.000000	354.559540	4.208214	-0.000385
1998.000000	354.608246	4.208748	-0.001183
1999.000000	354.809174	4.206688	-0.000755
2000.000000	354.989258	4.204933	-0.001377

2001.000000	355.250519	4.206345	-0.001091
2002.000000	355.259644	4.202034	-0.000641
2003.000000	355.512329	4.202682	-0.001099
2004.000000	355.597534	4.203560	-0.001083
2005.000000	355.742676	4.200089	-0.001114
2006.000000	355.986206	4.197914	-0.000175
2007.000000	356.132324	4.199936	-0.000004
2008.000000	356.303314	4.202759	-0.000267
2009.000000	356.449921	4.197647	-0.001083
2010.000000	356.594025	4.197723	-0.002121
2011.000000	356.744720	4.197037	-0.000923
2012.000000	356.915649	4.192459	-0.000381
2013.000000	357.105957	4.195663	-0.001053
2014.000000	357.274414	4.196503	0.000004
2015.000000	357.450958	4.189750	-0.001053
2016.000000	357.612793	4.195435	-0.000080
2017.000000	357.650330	4.190933	0.000145
2018.000000	357.916718	4.195511	-0.001102
2019.000000	358.115051	4.190437	-0.000221
2020.000000	358.260193	4.190170	-0.000740
2021.000000	358.371796	4.192496	-0.000084
2022.000000	358.522980	4.190780	-0.000027
2023.000000	358.647278	4.189712	-0.001408
2024.000000	358.836548	4.191429	-0.001931
2025.000000	358.946167	4.185133	-0.001236
2026.000000	359.082611	4.183416	-0.001538

2027.000000	359.317505	4.187537	0.000477
2028.000000	359.470764	4.182539	-0.000217
2029.000000	359.715271	4.187957	-0.000919
2030.000000	359.865479	4.182119	-0.000847
2031.000000	360.082642	4.183150	-0.002331
2032.000000	360.343384	4.180708	-0.000652
2033.000000	360.430145	4.177008	-0.000374
2034.000000	360.626495	4.181929	-0.000069
2035.000000	360.755371	4.180670	-0.000011
2036.000000	360.920776	4.178534	-0.001682
2037.000000	361.092255	4.178648	-0.002041
2038.000000	361.204834	4.174528	-0.001205
2039.000000	361.416382	4.172468	0.000214
2040.000000	361.599121	4.175596	0.000999
2041.000000	361.856323	4.179182	-0.000706
2042.000000	361.992798	4.176168	-0.002052
2043.000000	362.147552	4.168653	-0.001770
2044.000000	362.390594	4.170599	-0.000164
2045.000000	362.667603	4.173117	0.000267
2046.000000	362.739136	4.173117	-0.000759
2047.000000	362.976105	4.168730	-0.001251
2048.000000	363.124207	4.168997	-0.000103
2049.000000	363.315460	4.168921	0.000137
2050.000000	363.456543	4.172506	-0.000942
2051.000000	363.676178	4.167662	-0.001186
2052.000000	363.854309	4.164915	-0.001553

2053.000000	364.041504	4.166784	-0.000095
2054.000000	364.210938	4.165182	-0.000149
2055.000000	364.408813	4.167051	-0.001190
2056.000000	364.458038	4.164038	-0.001236
2057.000000	364.659454	4.161406	-0.001389
2058.000000	364.869019	4.161825	-0.000744
2059.000000	365.026794	4.161215	-0.000340
2060.000000	365.213013	4.160414	-0.000874
2061.000000	365.364655	4.160414	-0.000553
2062.000000	365.498138	4.157858	-0.000435
2063.000000	365.576782	4.159727	-0.001011
2064.000000	365.743164	4.158583	-0.001312
2065.000000	365.947632	4.154997	-0.002331
2066.000000	366.064331	4.153662	-0.001682
2067.000000	366.168335	4.150534	-0.001080
2068.000000	366.457520	4.152403	-0.000237
2069.000000	366.587433	4.150229	0.000374
2070.000000	366.766510	4.152632	0.000366
2071.000000	366.851288	4.152288	0.000103
2072.000000	367.054718	4.153204	-0.001564
2073.000000	367.194244	4.150457	-0.001427
2074.000000	367.308350	4.145727	-0.001598
2075.000000	367.405762	4.148893	-0.000240
2076.000000	367.568634	4.145994	0.000771
2077.000000	367.709198	4.149122	-0.000416
2078.000000	367.901459	4.149466	-0.000549

2079.000000	368.042480	4.145079	-0.001541
2080.000000	368.237823	4.145269	-0.000820
2081.000000	368.481903	4.143515	-0.000729
2082.000000	368.659485	4.146147	-0.000587
2083.000000	368.814209	4.140997	-0.000507
2084.000000	368.960327	4.143591	-0.001011
2085.000000	369.099854	4.142256	-0.001236
2086.000000	369.245972	4.140463	-0.001686
2087.000000	369.513367	4.136381	-0.000427
2088.000000	369.542755	4.138098	-0.000355
2089.000000	369.670105	4.141302	0.000271
2090.000000	369.803558	4.136229	-0.000034
2091.000000	370.026825	4.138670	-0.001049
2092.000000	370.239746	4.139242	-0.000542
2093.000000	370.315979	4.134550	-0.002167
2094.000000	370.474060	4.135580	-0.001831
2095.000000	370.674744	4.129667	-0.000626
2096.000000	370.821289	4.132528	-0.000816
2097.000000	370.972809	4.132948	-0.000176
2098.000000	371.158936	4.129858	-0.000290
2099.000000	371.230713	4.130125	-0.000362
2100.000000	371.449951	4.132490	-0.000824
2101.000000	371.598511	4.129819	-0.001095
2102.000000	371.765625	4.125890	-0.001095
2103.000000	371.908081	4.127950	-0.000404
2104.000000	372.110809	4.127950	0.000027

2105.000000	372.229218	4.126768	-0.000500
2106.000000	372.450012	4.126615	-0.001148
2107.000000	372.649170	4.126119	-0.001087
2108.000000	372.784668	4.122533	-0.001266
2109.000000	372.916595	4.123373	-0.000919
2110.000000	373.176025	4.121656	0.000385
2111.000000	373.298462	4.121961	-0.000099
2112.000000	373.546295	4.125165	-0.000404
2113.000000	373.695313	4.121122	-0.000992
2114.000000	373.894531	4.120054	-0.001026
2115.000000	374.079651	4.119558	-0.000519
2116.000000	374.311493	4.120817	-0.000759
2117.000000	374.469055	4.118146	-0.000080
2118.000000	374.612549	4.116964	-0.001087
2119.000000	374.864929	4.121084	-0.000626
2120.000000	374.990356	4.113912	-0.001057
2121.000000	375.252319	4.116544	-0.001362
2122.000000	375.352173	4.113950	0.000168
2123.000000	375.544800	4.114255	-0.000614
2124.000000	375.700348	4.115896	0.000000
2125.000000	375.829834	4.112501	0.000160
2126.000000	375.946747	4.114828	-0.000713
2127.000000	376.209656	4.115591	-0.001488
2128.000000	376.365692	4.110784	-0.001873
2129.000000	376.571381	4.107084	-0.001755
2130.000000	376.780151	4.109716	-0.000492

2131.000000	376.875977	4.107351	-0.001038
2132.000000	377.078156	4.108190	-0.001431
2133.000000	377.267822	4.102659	-0.000458
2134.000000	377.363159	4.104909	-0.000748
2135.000000	377.557861	4.106702	0.000263
2136.000000	377.820251	4.102430	-0.001331
2137.000000	377.959229	4.105215	-0.001137
2138.000000	378.153931	4.098997	-0.000629
2139.000000	378.262817	4.102735	-0.001709
2140.000000	378.411835	4.100523	-0.000141
2141.000000	378.557373	4.095907	-0.000454
2142.000000	378.793213	4.101705	-0.000031
2143.000000	378.993896	4.099874	-0.000423
2144.000000	379.032043	4.098386	-0.001747
2145.000000	379.197113	4.095449	-0.001343
2146.000000	379.384735	4.095182	-0.000465
2147.000000	379.628571	4.094762	-0.000008
2148.000000	379.832794	4.096403	-0.000240
2149.000000	379.970825	4.094800	-0.000847
2150.000000	380.123383	4.093961	-0.001495
2151.000000	380.247375	4.091749	-0.001442
2152.000000	380.392975	4.090452	-0.001392
2153.000000	380.562134	4.089345	-0.000683
2154.000000	380.705231	4.088201	-0.000088
2155.000000	380.968262	4.089460	-0.000275
2156.000000	381.093781	4.089956	0.000008

2157.000000	381.277008	4.087095	-0.001373
2158.000000	381.411041	4.089422	-0.000820
2159.000000	381.610840	4.082860	-0.000748
2160.000000	381.726807	4.087972	-0.001198
2161.000000	381.832245	4.084081	-0.000263
2162.000000	381.941650	4.082822	-0.000996
2163.000000	382.124390	4.084119	-0.000549
2164.000000	382.352325	4.082975	-0.000515
2165.000000	382.496887	4.081258	-0.000923
2166.000000	382.628906	4.081678	0.000172
2167.000000	382.757935	4.080152	-0.000599
2168.000000	382.917572	4.084386	-0.000805
2169.000000	383.174591	4.076910	-0.000648
2170.000000	383.290039	4.079275	-0.000896
2171.000000	383.499390	4.079465	-0.000164
2172.000000	383.609375	4.078626	-0.001541
2173.000000	383.813690	4.076414	-0.001324
2174.000000	384.034027	4.073094	0.000065
2175.000000	384.083252	4.075613	0.000214
2176.000000	384.243408	4.079351	-0.000080
2177.000000	384.355347	4.074353	-0.001755
2178.000000	384.668091	4.073324	-0.002037
2179.000000	384.822693	4.069851	-0.001419
2180.000000	384.992340	4.071415	-0.001011
2181.000000	385.101318	4.068211	-0.000713
2182.000000	385.234344	4.069088	-0.000794

2183.000000	385.454224	4.067448	-0.000660
2184.000000	385.680115	4.067829	-0.001236
2185.000000	385.811646	4.065541	-0.000526
2186.000000	385.957245	4.063862	-0.000614
2187.000000	386.104309	4.066799	-0.000446
2188.000000	386.233368	4.064129	-0.001087
2189.000000	386.359833	4.063175	-0.001350
2190.000000	386.586761	4.060238	-0.000141
2191.000000	386.703217	4.061993	0.000111
2192.000000	386.855835	4.064014	0.000595
2193.000000	387.037079	4.061840	-0.000835
2194.000000	387.213745	4.063290	-0.001404
2195.000000	387.361847	4.058178	-0.001194
2196.000000	387.566162	4.058826	-0.001617
2197.000000	387.778015	4.058102	-0.001587
2198.000000	387.901001	4.055241	-0.001408
2199.000000	388.118896	4.052036	-0.001072
2200.000000	388.265472	4.054821	-0.000305
2201.000000	388.456726	4.052952	-0.000355
2202.000000	388.624908	4.053257	-0.001205
2203.000000	388.718262	4.051044	-0.000679
2204.000000	388.924072	4.049747	-0.001156
2205.000000	389.116333	4.051311	-0.001102
2206.000000	389.314697	4.047344	-0.000565
2207.000000	389.473785	4.046734	-0.000904
2208.000000	389.619904	4.049213	-0.000599

2209.000000	389.858337	4.045856	-0.001160
2210.000000	390.033997	4.044788	-0.001797
2211.000000	390.238342	4.043148	-0.001175
2212.000000	390.326782	4.041584	-0.000881
2213.000000	390.546356	4.041584	-0.000332
2214.000000	390.687439	4.041164	-0.000324
2215.000000	390.806641	4.041698	-0.000156
2216.000000	390.990448	4.039906	-0.000385
2217.000000	391.284027	4.041431	-0.001442
2218.000000	391.423676	4.039371	-0.001507
2219.000000	391.597015	4.034756	-0.001663
2220.000000	391.788269	4.035709	-0.001141
2221.000000	391.977051	4.034946	-0.001198
2222.000000	392.072449	4.033573	-0.000381
2223.000000	392.288544	4.029835	-0.000629
2224.000000	392.395325	4.036358	-0.000774
2225.000000	392.634796	4.030407	-0.000191
2226.000000	392.712280	4.029415	-0.001438
2227.000000	392.907990	4.032352	-0.000908
2228.000000	393.054047	4.028194	-0.001122
2229.000000	393.190674	4.026478	-0.001320
2230.000000	393.371033	4.026745	-0.000549
2231.000000	393.516083	4.026478	-0.000534
2232.000000	393.698364	4.025448	-0.001797
2233.000000	393.882172	4.024456	-0.001469
2234.000000	394.067474	4.018772	-0.001556

2235.000000	394.171783	4.022472	-0.001163
2236.000000	394.426147	4.018658	-0.000187
2237.000000	394.495209	4.018696	-0.001175
2238.000000	394.747070	4.019726	-0.001102
2239.000000	394.904022	4.016064	-0.001068
2240.000000	395.044128	4.014462	-0.000359
2241.000000	395.209534	4.015987	0.001205
2242.000000	395.317871	4.017971	0.000092
2243.000000	395.499695	4.020336	-0.001919
2244.000000	395.674988	4.012745	-0.002239
2245.000000	395.758453	4.009007	-0.002022
2246.000000	395.866760	4.012440	-0.000946
2247.000000	396.025757	4.010380	-0.000992
2248.000000	396.152893	4.007328	-0.001377
2249.000000	396.413239	4.006603	-0.000370
2250.000000	396.564697	4.007442	-0.000416
2251.000000	396.719727	4.008472	-0.001495
2252.000000	396.889130	4.004314	-0.001801
2253.000000	397.068451	4.000690	-0.001236
2254.000000	397.371521	4.002331	0.000221
2255.000000	397.416687	4.003284	0.000332
2256.000000	397.665558	4.004124	-0.000923
2257.000000	397.833496	4.001453	-0.001923
2258.000000	398.051056	3.998631	-0.002022
2259.000000	398.290527	3.996418	-0.001713
2260.000000	398.405762	3.996532	-0.000378

2261.000000	398.570190	3.993938	-0.000961
2262.000000	398.650635	3.997982	-0.001320
2263.000000	398.864258	3.990887	-0.000767
2264.000000	399.024750	3.991459	-0.000904
2265.000000	399.290527	3.993366	-0.000626
2266.000000	399.350586	3.992222	-0.001705
2267.000000	399.531952	3.987377	-0.002499
2268.000000	399.767426	3.985928	-0.001747
2269.000000	399.879639	3.984020	-0.000175
2270.000000	400.066467	3.985165	0.000164
2271.000000	400.225464	3.986881	0.000374
2272.000000	400.405273	3.985317	-0.000534
2273.000000	400.497681	3.985813	-0.001587
2274.000000	400.706299	3.983028	-0.002831
2275.000000	400.873230	3.980091	-0.001945
2276.000000	401.066437	3.974026	-0.000782
2277.000000	401.118134	3.980587	-0.000706
2278.000000	401.269653	3.978870	-0.000599
2279.000000	401.456421	3.974140	-0.001110
2280.000000	401.673035	3.974255	0.000015
2281.000000	401.791748	3.977345	0.000057
2282.000000	401.999420	3.977345	-0.001621
2283.000000	402.161377	3.972881	-0.002934
2284.000000	402.289551	3.968380	-0.002693
2285.000000	402.455444	3.967159	-0.001820
2286.000000	402.646729	3.966740	-0.000149

2287.000000	402.878204	3.964603	-0.000530
2288.000000	402.913940	3.968914	-0.000553
2289.000000	403.155396	3.963421	-0.000603
2290.000000	403.360077	3.964565	-0.001469
2291.000000	403.496674	3.963764	-0.000889
2292.000000	403.673035	3.961399	-0.001801
2293.000000	403.819580	3.960560	-0.002163
2294.000000	404.064972	3.957165	-0.001084
2295.000000	404.189178	3.955066	-0.000340
2296.000000	404.352600	3.958728	-0.000424
2297.000000	404.494171	3.958079	-0.001190
2298.000000	404.597046	3.953540	-0.001293
2299.000000	404.788788	3.951709	-0.000564
2300.000000	404.954712	3.955448	-0.000420
2301.000000	405.121094	3.954303	-0.001141
2302.000000	405.296448	3.950144	-0.001618
2303.000000	405.450958	3.948656	-0.002278
2304.000000	405.541840	3.950182	-0.001385
2305.000000	405.723694	3.942896	-0.001003
2306.000000	405.908478	3.946100	-0.002453
2307.000000	406.135010	3.945681	-0.001530
2308.000000	406.282593	3.936526	-0.001732
2309.000000	406.454437	3.940035	-0.001678
2310.000000	406.663574	3.940264	0.000187
2311.000000	406.821533	3.935419	-0.001400
2312.000000	407.022766	3.939768	-0.001740

2313.000000	407.192627	3.933283	-0.001347
2314.000000	407.294006	3.932635	-0.001240
2315.000000	407.529449	3.932253	0.000305
2316.000000	407.654633	3.934084	-0.000771
2317.000000	407.839966	3.934084	-0.001637
2318.000000	408.080414	3.927866	-0.001957
2319.000000	408.138977	3.927180	-0.001637
2320.000000	408.404297	3.927752	-0.000954
2321.000000	408.591553	3.925959	-0.001106
2322.000000	408.760437	3.923708	-0.001144
2323.000000	408.912964	3.923670	-0.001091
2324.000000	409.031677	3.923174	-0.000946
2325.000000	409.190186	3.920771	-0.001564
2326.000000	409.342163	3.920427	-0.001392
2327.000000	409.623322	3.917223	-0.001511
2328.000000	409.736115	3.917986	-0.001484
2329.000000	409.918396	3.914438	-0.001083
2330.000000	410.041595	3.914400	-0.001938
2331.000000	410.211914	3.913599	-0.001431
2332.000000	410.388184	3.908716	-0.001339
2333.000000	410.587250	3.910128	-0.000675
2334.000000	410.782898	3.909441	0.000057
2335.000000	410.843994	3.909861	-0.000069
2336.000000	411.018250	3.909136	-0.000870
2337.000000	411.223328	3.909937	-0.001476
2338.000000	411.344421	3.905054	-0.002403

2339.000000	411.475098	3.904520	-0.002918
2340.000000	411.637390	3.899828	-0.001266
2341.000000	411.828125	3.897959	-0.001743
2342.000000	411.953217	3.902002	-0.001415
2343.000000	412.040619	3.894716	-0.001900
2344.000000	412.298767	3.894373	-0.001884
2345.000000	412.454193	3.892275	-0.000858
2346.000000	412.764526	3.893801	-0.001583
2347.000000	412.860870	3.890711	-0.002304
2348.000000	413.014282	3.887239	-0.002453
2349.000000	413.176208	3.884035	-0.001686
2350.000000	413.323608	3.884874	-0.001019
2351.000000	413.541595	3.883463	-0.000847
2352.000000	413.700470	3.882433	-0.001205
2353.000000	413.836517	3.881021	-0.001694
2354.000000	414.060974	3.880068	-0.001598
2355.000000	414.197510	3.876176	-0.001236
2356.000000	414.324097	3.876863	-0.001545
2357.000000	414.452179	3.876443	-0.000740
2358.000000	414.748138	3.872209	-0.001209
2359.000000	414.880249	3.874803	-0.001667
2360.000000	415.119019	3.871637	-0.001198
2361.000000	415.166687	3.868394	-0.001984
2362.000000	415.351929	3.869424	-0.002117
2363.000000	415.511322	3.865991	-0.001419
2364.000000	415.714355	3.862253	-0.001789

2365.000000	415.923920	3.864885	-0.001373
2366.000000	416.085785	3.861032	-0.001156
2367.000000	416.282898	3.859735	-0.001877
2368.000000	416.378204	3.859048	-0.000973
2369.000000	416.549561	3.856493	-0.001637
2370.000000	416.696960	3.857790	-0.002087
2371.000000	416.815186	3.852182	-0.001244
2372.000000	417.001343	3.850770	-0.001297
2373.000000	417.287384	3.853784	-0.000340
2374.000000	417.368286	3.850503	-0.000568
2375.000000	417.542114	3.850618	-0.001640
2376.000000	417.648865	3.849512	-0.001034
2377.000000	417.869263	3.846078	-0.001404
2378.000000	418.005859	3.847604	-0.001434
2379.000000	418.169220	3.844553	-0.001526
2380.000000	418.391144	3.843103	-0.001789
2381.000000	418.470093	3.840700	-0.001358
2382.000000	418.665222	3.840585	-0.001465
2383.000000	418.896606	3.839021	-0.001000
2384.000000	419.005371	3.836617	-0.001049
2385.000000	419.206970	3.837686	-0.001473
2386.000000	419.306732	3.836007	-0.001457
2387.000000	419.486938	3.831963	-0.002068
2388.000000	419.593750	3.832192	-0.002232
2389.000000	419.778931	3.829254	-0.001919
2390.000000	419.911469	3.826203	-0.001930

2391.000000	420.037140	3.825363	-0.001598
2392.000000	420.261475	3.824486	-0.001629
2393.000000	420.388794	3.822121	-0.001869
2394.000000	420.578064	3.819679	-0.001453
2395.000000	420.743256	3.818421	-0.000641
2396.000000	420.928101	3.819069	-0.000202
2397.000000	421.131134	3.819222	-0.000912
2398.000000	421.283081	3.818268	-0.001450
2399.000000	421.410889	3.814263	-0.001709
2400.000000	421.615356	3.814301	-0.002007
2401.000000	421.790466	3.812660	-0.001511
2402.000000	422.035278	3.809036	-0.001118
2403.000000	422.140503	3.809342	-0.001163
2404.000000	422.312531	3.810372	-0.001644
2405.000000	422.518097	3.806175	-0.001560
2406.000000	422.665558	3.802399	-0.002216
2407.000000	422.822876	3.805527	-0.001850
2408.000000	423.053467	3.799614	-0.000957
2409.000000	423.210327	3.798317	-0.001236
2410.000000	423.349457	3.801216	-0.001213
2411.000000	423.585938	3.798546	-0.001221
2412.000000	423.758545	3.793434	-0.001358
2413.000000	423.920258	3.796105	-0.000534
2414.000000	424.089844	3.795647	-0.000057
2415.000000	424.286499	3.794770	-0.000710
2416.000000	424.509705	3.793816	-0.000816

2417.000000	424.646362	3.793473	-0.001076
2418.000000	424.834717	3.792214	-0.002525
2419.000000	424.971375	3.790192	-0.003052
2420.000000	425.192139	3.782830	-0.002010
2421.000000	425.273712	3.782906	-0.000473
2422.000000	425.475281	3.785805	0.000359
2423.000000	425.680298	3.786339	-0.001305
2424.000000	425.831726	3.782906	-0.002583
2425.000000	425.901550	3.777832	-0.002110
2426.000000	426.143433	3.777145	-0.001595
2427.000000	426.324402	3.778672	-0.001034
2428.000000	426.414307	3.774513	-0.000607
2429.000000	426.654297	3.773979	-0.001835
2430.000000	426.775726	3.776459	-0.001446
2431.000000	426.923126	3.768524	-0.001507
2432.000000	427.088867	3.770012	-0.001442
2433.000000	427.313995	3.769669	-0.001331
2434.000000	427.365173	3.768677	-0.001461
2435.000000	427.609497	3.762535	-0.001492
2436.000000	427.731476	3.766273	-0.001316
2437.000000	427.903564	3.763412	-0.000957
2438.000000	428.041138	3.761658	-0.002312
2439.000000	428.207306	3.760056	-0.001190
2440.000000	428.328308	3.756393	-0.000103
2441.000000	428.478760	3.760094	-0.000191
2442.000000	428.673431	3.761124	-0.000702

2443.000000	428.861694	3.756737	-0.001648
2444.000000	428.978271	3.754562	-0.001534
2445.000000	429.111511	3.755135	-0.000809
2446.000000	429.320435	3.754257	-0.000214
2447.000000	429.429626	3.752846	-0.001587
2448.000000	429.616394	3.754639	-0.002621
2449.000000	429.694611	3.747009	-0.002262
2450.000000	429.925171	3.744072	-0.002003
2451.000000	430.070984	3.746819	-0.000317
2452.000000	430.299438	3.744720	-0.000896
2453.000000	430.405212	3.745102	-0.001938
2454.000000	430.627808	3.740448	-0.001591
2455.000000	430.765900	3.739265	-0.001465
2456.000000	430.868256	3.739685	-0.001133
2457.000000	431.128052	3.738159	-0.001190
2458.000000	431.272583	3.735336	-0.002205
2459.000000	431.476807	3.735489	-0.001640
2460.000000	431.657593	3.729996	-0.001057
2461.000000	431.730103	3.732628	-0.001186
2462.000000	431.908966	3.731483	0.000137
2463.000000	432.136993	3.728813	-0.000721
2464.000000	432.260681	3.732590	-0.000965
2465.000000	432.427612	3.728470	-0.001133
2466.000000	432.720215	3.726830	-0.001495
2467.000000	432.893555	3.726028	-0.001248
2468.000000	433.091797	3.726333	-0.002125

2469.000000	433.204590	3.722480	-0.002049
2470.000000	433.415161	3.717978	-0.001312
2471.000000	433.545349	3.719962	-0.001503
2472.000000	433.769379	3.721030	-0.001492
2473.000000	433.959167	3.713438	-0.001236
2474.000000	434.072937	3.713782	-0.000748
2475.000000	434.235352	3.717406	0.000237
2476.000000	434.441040	3.715307	-0.000332
2477.000000	434.538361	3.713858	-0.002216
2478.000000	434.685974	3.713896	-0.001225
2479.000000	434.938812	3.707030	-0.000931
2480.000000	435.045044	3.712599	-0.000908
2481.000000	435.133057	3.709852	-0.000961
2482.000000	435.344177	3.707945	-0.002453
2483.000000	435.490173	3.704550	-0.000839
2484.000000	435.668060	3.702986	-0.000740
2485.000000	435.766449	3.708136	-0.000862
2486.000000	435.965607	3.702452	-0.001038
2487.000000	436.234375	3.700506	-0.001839
2488.000000	436.295441	3.701613	-0.001282
2489.000000	436.501099	3.699362	-0.000690
2490.000000	436.660095	3.696615	-0.000599
2491.000000	436.783295	3.699553	-0.001061
2492.000000	437.004883	3.698523	-0.002140
2493.000000	437.158813	3.693106	-0.002876
2494.000000	437.257202	3.689139	-0.002033

2495.000000	437.516052	3.689863	0.000008
2496.000000	437.663574	3.689978	0.001209
2497.000000	437.844421	3.692724	-0.000309
2498.000000	438.014832	3.693754	-0.001385
2499.000000	438.208008	3.686430	-0.001427
2500.000000	438.429565	3.686201	-0.001377
2501.000000	438.500610	3.689367	-0.000599
2502.000000	438.755005	3.685400	-0.001614
2503.000000	438.811127	3.683836	-0.001404
2504.000000	439.024200	3.680899	-0.000809
2505.000000	439.247253	3.684599	-0.001389
2506.000000	439.401245	3.680975	-0.001232
2507.000000	439.552765	3.676855	-0.001656
2508.000000	439.722687	3.678610	-0.001026
2509.000000	439.893921	3.677504	-0.001469
2510.000000	440.114258	3.675520	-0.001942
2511.000000	440.225220	3.671057	-0.001621
2512.000000	440.347839	3.672125	-0.001606
2513.000000	440.632446	3.671095	-0.001740
2514.000000	440.814484	3.667471	-0.001236
2515.000000	440.960968	3.664686	-0.000595
2516.000000	441.119568	3.669149	-0.000320
2517.000000	441.298676	3.667280	-0.000580
2518.000000	441.488464	3.664572	-0.001221
2519.000000	441.552185	3.664076	-0.000687
2520.000000	441.822266	3.664648	-0.000271

2521.000000	441.963867	3.663809	-0.001625
2522.000000	442.103027	3.663351	-0.002117
2523.000000	442.299164	3.656599	-0.001919
2524.000000	442.432983	3.657667	-0.001083
2525.000000	442.634888	3.657057	-0.000164
2526.000000	442.740997	3.657705	-0.000427
2527.000000	442.928833	3.655760	-0.000397
2528.000000	443.022736	3.656179	-0.001373
2529.000000	443.157562	3.655836	-0.001820
2530.000000	443.360931	3.650801	-0.001465
2531.000000	443.449005	3.649351	-0.000938
2532.000000	443.656830	3.652098	-0.000122
2533.000000	443.857300	3.650496	-0.000767
2534.000000	444.019318	3.649618	-0.002548
2535.000000	444.157562	3.646757	-0.002167
2536.000000	444.283569	3.641225	-0.000961
2537.000000	444.452484	3.643857	-0.000175
2538.000000	444.631531	3.646261	-0.000736
2539.000000	444.757996	3.643362	-0.001877
2540.000000	444.886993	3.637792	-0.002319
2541.000000	445.150238	3.638708	-0.001278
2542.000000	445.322998	3.636991	-0.000057
2543.000000	445.363892	3.637372	-0.000652
2544.000000	445.616394	3.638174	-0.000763
2545.000000	445.706482	3.634855	-0.001038
2546.000000	445.935150	3.634435	-0.001400

2547.000000	446.229095	3.634054	-0.001728
2548.000000	446.382843	3.631574	-0.002602
2549.000000	446.601807	3.627645	-0.002167
2550.000000	446.774109	3.624631	-0.001461
2551.000000	446.892365	3.626691	-0.000904
2552.000000	447.022736	3.624746	-0.000988
2553.000000	447.250488	3.623067	-0.000935
2554.000000	447.462646	3.621503	-0.000828
2555.000000	447.628113	3.623640	-0.000706
2556.000000	447.733734	3.620321	-0.000923
2557.000000	447.864532	3.620130	-0.001617
2558.000000	448.098663	3.618642	-0.000977
2559.000000	448.319122	3.616392	-0.001392
2560.000000	448.372101	3.617307	-0.001438
2561.000000	448.563416	3.613836	-0.001419
2562.000000	448.684021	3.612730	-0.001392
2563.000000	448.949738	3.611585	-0.001942
2564.000000	449.161896	3.611471	-0.001774
2565.000000	449.237396	3.604756	-0.001381
2566.000000	449.468018	3.607274	-0.001103
2567.000000	449.536163	3.606778	-0.001057
2568.000000	449.734222	3.604947	-0.001598
2569.000000	449.924011	3.600636	-0.001854
2570.000000	450.051544	3.602353	-0.001595
2571.000000	450.191528	3.598805	-0.000584
2572.000000	450.395935	3.597889	-0.000527

2573.000000	450.628906	3.599949	-0.000576
2574.000000	450.610199	3.599148	-0.000801
2575.000000	450.824493	3.595295	-0.001163
2576.000000	450.928589	3.596211	-0.000946
2577.000000	451.045532	3.595600	-0.000927
2578.000000	451.323242	3.594265	-0.001900
2579.000000	451.447540	3.591633	-0.002060
2580.000000	451.624451	3.588696	-0.001842
2581.000000	451.820557	3.588085	-0.001835
2582.000000	451.980743	3.586827	-0.001896
2583.000000	452.247101	3.583393	-0.001953
2584.000000	452.287354	3.581562	-0.002487
2585.000000	452.477051	3.580952	-0.001175
2586.000000	452.634308	3.575611	-0.001465
2587.000000	452.851929	3.580494	-0.001060
2588.000000	452.954132	3.574467	-0.001205
2589.000000	453.202271	3.576222	-0.001999
2590.000000	453.327148	3.571720	-0.001266
2591.000000	453.482452	3.571873	-0.001423
2592.000000	453.662781	3.570309	-0.000549
2593.000000	453.878998	3.569813	-0.001083
2594.000000	454.056396	3.570004	-0.001183
2595.000000	454.231873	3.566609	-0.001514
2596.000000	454.431824	3.565998	-0.001205
2597.000000	454.587646	3.564244	-0.001152
2598.000000	454.710480	3.565159	-0.001656

2599.000000	454.876556	3.561268	-0.002869
2600.000000	455.096222	3.559208	-0.002556
2601.000000	455.151703	3.552876	-0.001209
2602.000000	455.411163	3.556576	-0.001282
2603.000000	455.568939	3.556538	-0.000774
2604.000000	455.779724	3.550968	-0.001869
2605.000000	455.965454	3.551808	-0.002007
2606.000000	456.150269	3.549595	-0.001354
2607.000000	456.231323	3.547192	-0.002255
2608.000000	456.444092	3.546505	-0.002129
2609.000000	456.696167	3.542080	-0.001522
2610.000000	456.921753	3.541508	-0.001366
2611.000000	457.070648	3.542080	-0.001221
2612.000000	457.263275	3.539677	-0.001968
2613.000000	457.406281	3.536892	-0.002083
2614.000000	457.537964	3.534260	-0.002167
2615.000000	457.771362	3.534374	-0.001499
2616.000000	457.874054	3.530102	-0.001068
2617.000000	458.048035	3.531475	-0.001553
2618.000000	458.226929	3.530369	-0.001774
2619.000000	458.326660	3.526478	-0.002350
2620.000000	458.428833	3.523731	-0.002251
2621.000000	458.685883	3.523045	-0.001587
2622.000000	458.731079	3.520832	-0.001060
2623.000000	458.975281	3.519993	-0.000790
2624.000000	459.191986	3.519955	-0.001266

2625.000000	459.345825	3.519535	-0.002369
2626.000000	459.503540	3.514729	-0.002686
2627.000000	459.620972	3.510761	-0.001846
2628.000000	459.756622	3.510914	-0.000542
2629.000000	459.877472	3.512211	-0.000191
2630.000000	460.106812	3.511295	-0.001343
2631.000000	460.212982	3.509617	-0.002800
2632.000000	460.428589	3.505497	-0.003498
2633.000000	460.610535	3.501110	-0.002834
2634.000000	460.715698	3.498058	-0.001911
2635.000000	460.878754	3.499165	-0.001198
2636.000000	461.005737	3.496914	-0.001728
2637.000000	461.129272	3.495693	-0.002316
2638.000000	461.272400	3.491153	-0.001682
2639.000000	461.452911	3.490467	-0.002140
2640.000000	461.602325	3.491115	-0.001415
2641.000000	461.729279	3.485011	-0.002606
2642.000000	461.872894	3.486804	-0.002396
2643.000000	462.092804	3.479594	-0.001595
2644.000000	462.205688	3.481844	-0.001793
2645.000000	462.431030	3.479517	-0.000832
2646.000000	462.472412	3.477877	-0.002445
2647.000000	462.678711	3.477419	-0.002422
2648.000000	462.825684	3.470667	-0.002380
2649.000000	462.951691	3.471010	-0.002689
2650.000000	463.203308	3.469179	-0.002323

2651.000000	463.315674	3.464716	-0.001957
2652.000000	463.547791	3.462198	-0.001610
2653.000000	463.743439	3.464716	-0.000931
2654.000000	463.849976	3.461130	-0.001678
2655.000000	464.057800	3.460596	-0.002312
2656.000000	464.277710	3.455866	-0.002632
2657.000000	464.385773	3.455790	-0.002342
2658.000000	464.551270	3.450373	-0.001095
2659.000000	464.828125	3.451632	-0.001163
2660.000000	465.077179	3.452471	-0.001141
2661.000000	465.149292	3.448923	-0.003017
2662.000000	465.350220	3.446024	-0.003014
2663.000000	465.500122	3.439768	-0.003448
2664.000000	465.667023	3.441981	-0.002377
2665.000000	465.835938	3.433703	-0.001991
2666.000000	466.061707	3.437174	-0.001644
2667.000000	466.190125	3.432215	-0.000542
2668.000000	466.345337	3.434504	-0.001240
2669.000000	466.541016	3.432329	-0.001679
2670.000000	466.744873	3.430918	-0.003204
2671.000000	466.866974	3.425615	-0.003677
2672.000000	467.084076	3.421839	-0.003552
2673.000000	467.172607	3.418482	-0.002312
2674.000000	467.372589	3.416727	-0.001949
2675.000000	467.544922	3.416613	-0.001637
2676.000000	467.696716	3.413027	-0.001568

2677.000000	467.910339	3.412149	-0.002487
2678.000000	468.089935	3.411119	-0.002266
2679.000000	468.237335	3.405130	-0.002979
2680.000000	468.359497	3.405207	-0.003017
2681.000000	468.479126	3.400209	-0.001549
2682.000000	468.651978	3.398493	-0.001167
2683.000000	468.799896	3.400743	-0.001251
2684.000000	468.984741	3.399103	-0.002716
2685.000000	469.234955	3.393648	-0.002926
2686.000000	469.353638	3.388460	-0.003387
2687.000000	469.536621	3.391436	-0.002472
2688.000000	469.721527	3.383272	-0.002251
2689.000000	469.848022	3.383883	-0.002831
2690.000000	470.049500	3.380983	-0.002010
2691.000000	470.187744	3.378427	-0.002365
2692.000000	470.332275	3.375948	-0.001965
2693.000000	470.541992	3.374574	-0.001972
2694.000000	470.679199	3.373086	-0.001824
2695.000000	470.900085	3.369996	-0.002007
2696.000000	471.030029	3.369118	-0.003048
2697.000000	471.137146	3.366524	-0.003548
2698.000000	471.366302	3.359581	-0.003941
2699.000000	471.584808	3.357025	-0.002518
2700.000000	471.763855	3.354164	-0.001678
2701.000000	471.851440	3.356644	-0.001476
2702.000000	471.984314	3.351380	-0.002216

2703.000000	472.215912	3.351036	-0.003189
2704.000000	472.345825	3.345886	-0.001926
2705.000000	472.424683	3.343445	-0.002605
2706.000000	472.672394	3.345543	-0.002159
2707.000000	472.758484	3.338181	-0.002636
2708.000000	472.925873	3.337723	-0.003208
2709.000000	473.052429	3.334175	-0.002865
2710.000000	473.287384	3.331505	-0.002987
2711.000000	473.453369	3.326966	-0.003181
2712.000000	473.561951	3.326393	-0.001873
2713.000000	473.758972	3.320824	-0.001072
2714.000000	473.815918	3.325211	-0.002319
2715.000000	474.001831	3.322197	-0.002781
2716.000000	474.172150	3.314110	-0.003925
2717.000000	474.334717	3.312469	-0.002876
2718.000000	474.512299	3.310448	-0.001755
2719.000000	474.667999	3.309647	-0.001598
2720.000000	474.802277	3.306747	-0.002781
2721.000000	474.925385	3.306328	-0.003632
2722.000000	475.197449	3.298203	-0.003437
2723.000000	475.319092	3.295761	-0.003448
2724.000000	475.479675	3.294846	-0.002750
2725.000000	475.598419	3.290764	-0.002613
2726.000000	475.781372	3.286949	-0.002464
2727.000000	475.949738	3.286644	-0.002480
2728.000000	476.124939	3.284584	-0.002167

2729.000000	476.327332	3.279549	-0.002697
2730.000000	476.421753	3.279663	-0.002148
2731.000000	476.619385	3.275620	-0.001820
2732.000000	476.860687	3.275810	-0.002216
2733.000000	477.024200	3.272377	-0.002605
2734.000000	477.317627	3.270203	-0.003368
2735.000000	477.412048	3.265396	-0.003719
2736.000000	477.726410	3.262459	-0.003498
2737.000000	477.835388	3.257652	-0.002907
2738.000000	478.033447	3.256583	-0.003308
2739.000000	478.207703	3.253798	-0.002842
2740.000000	478.358490	3.247847	-0.002918
2741.000000	478.519073	3.247809	-0.002171
2742.000000	478.710327	3.244986	-0.002663
2743.000000	478.864594	3.244375	-0.002613
2744.000000	479.060181	3.236250	-0.003052
2745.000000	479.189209	3.239111	-0.002579
2746.000000	479.328857	3.232359	-0.001972
2747.000000	479.493835	3.233427	-0.002731
2748.000000	479.619324	3.229231	-0.001534
2749.000000	479.794952	3.227019	-0.002575
2750.000000	480.004333	3.227896	-0.002434
2751.000000	480.241699	3.221220	-0.002678
2752.000000	480.298096	3.219961	-0.002872
2753.000000	480.439697	3.217596	-0.002659
2754.000000	480.702667	3.215346	-0.003826

2755.000000	480.812988	3.210234	-0.003380
2756.000000	481.033630	3.204512	-0.003181
2757.000000	481.195923	3.206114	-0.002750
2758.000000	481.320740	3.201498	-0.002651
2759.000000	481.526855	3.197989	-0.003342
2760.000000	481.660278	3.195318	-0.002632
2761.000000	481.862091	3.192495	-0.002155
2762.000000	482.051392	3.191084	-0.002399
2763.000000	482.263794	3.189329	-0.002129
2764.000000	482.419952	3.184904	-0.002094
2765.000000	482.545166	3.184942	-0.002209
2766.000000	482.755157	3.182806	-0.002598
2767.000000	482.883301	3.179335	-0.002762
2768.000000	483.020599	3.174719	-0.003033
2769.000000	483.218536	3.175177	-0.003139
2770.000000	483.403564	3.169722	-0.002773
2771.000000	483.594818	3.166136	-0.003090
2772.000000	483.717621	3.165373	-0.002544
2773.000000	483.897247	3.161901	-0.002281
2774.000000	484.088501	3.159117	-0.003288
2775.000000	484.207031	3.157858	-0.003021
2776.000000	484.435822	3.150953	-0.002666
2777.000000	484.587585	3.150877	-0.002922
2778.000000	484.754730	3.149275	-0.002777
2779.000000	484.861603	3.144087	-0.003758
2780.000000	485.011902	3.140462	-0.003498

2781.000000	485.201691	3.136494	-0.002197
2782.000000	485.349121	3.135579	-0.000584
2783.000000	485.520111	3.135541	-0.001099
2784.000000	485.660278	3.138021	-0.002354
2785.000000	485.837036	3.129781	-0.003262
2786.000000	485.926147	3.126691	-0.004303
2787.000000	486.072144	3.124897	-0.003162
2788.000000	486.289398	3.118947	-0.002956
2789.000000	486.475769	3.117840	-0.002090
2790.000000	486.604919	3.115437	-0.001621
2791.000000	486.680023	3.116200	-0.001774
2792.000000	486.889526	3.111660	-0.001793
2793.000000	486.988312	3.110859	-0.002144
2794.000000	487.144867	3.109143	-0.002861
2795.000000	487.361664	3.106740	-0.003437
2796.000000	487.579834	3.099415	-0.002842
2797.000000	487.698822	3.098538	-0.002617
2798.000000	487.846710	3.099034	-0.002117
2799.000000	488.032593	3.093846	-0.002937
2800.000000	488.150146	3.091176	-0.003811
2801.000000	488.381378	3.087780	-0.003304
2802.000000	488.536011	3.083012	-0.003140
2803.000000	488.688721	3.081410	-0.002449
2804.000000	488.827423	3.078663	-0.001911
2805.000000	489.109711	3.077709	-0.002365
2806.000000	489.172791	3.075306	-0.002285

2807.000000	489.399689	3.071262	-0.002232
2808.000000	489.572601	3.070461	-0.002312
2809.000000	489.753754	3.068974	-0.001945
2810.000000	489.896332	3.064892	-0.002647
2811.000000	490.101074	3.064320	-0.002022
2812.000000	490.272064	3.059551	-0.001194
2813.000000	490.438721	3.061535	-0.001938
2814.000000	490.645294	3.060314	-0.002445
2815.000000	490.864532	3.054249	-0.003643
2816.000000	491.041290	3.050968	-0.003159
2817.000000	491.196899	3.047993	-0.002285
2818.000000	491.419922	3.047649	-0.002426
2819.000000	491.578400	3.044483	-0.002285
2820.000000	491.747925	3.040592	-0.002338
2821.000000	491.791779	3.040096	-0.001808
2822.000000	492.001312	3.038151	-0.001953
2823.000000	492.260925	3.036663	-0.001781
2824.000000	492.368408	3.032543	-0.001862
2825.000000	492.596710	3.033993	-0.002266
2826.000000	492.731079	3.030178	-0.002419
2827.000000	492.903076	3.026515	-0.002377
2828.000000	493.069702	3.024188	-0.001278
2829.000000	493.280212	3.025104	-0.001373
2830.000000	493.438721	3.024494	-0.002209
2831.000000	493.567322	3.019496	-0.002610
2832.000000	493.692993	3.015948	-0.002484

2833.000000	493.917999	3.016330	-0.002060
2834.000000	494.082764	3.013659	-0.001965
2835.000000	494.268646	3.010340	-0.001934
2836.000000	494.484009	3.009120	-0.001644
2837.000000	494.558685	3.008929	-0.001495
2838.000000	494.702179	3.006144	-0.002319
2839.000000	494.796631	3.004351	-0.003006
2840.000000	494.986359	2.999812	-0.001919
2841.000000	495.106293	2.997065	-0.001404
2842.000000	495.322113	3.000193	-0.000465
2843.000000	495.538422	2.997141	-0.000168
2844.000000	495.663605	2.997447	-0.001427
2845.000000	495.812042	2.997599	-0.001911
2846.000000	495.963745	2.992831	-0.002869
2847.000000	496.088959	2.989893	-0.002670
2848.000000	496.278748	2.986956	-0.001865
2849.000000	496.442017	2.987185	-0.001690
2850.000000	496.633789	2.984858	-0.001438
2851.000000	496.739777	2.982493	-0.001564
2852.000000	496.938232	2.982111	-0.001732
2853.000000	497.126984	2.980738	-0.001373
2854.000000	497.308685	2.977076	-0.002037
2855.000000	497.454102	2.978144	-0.001247
2856.000000	497.575470	2.973223	-0.001133
2857.000000	497.755127	2.976428	-0.001499
2858.000000	497.949341	2.972270	-0.000912

2859.000000	498.165558	2.971125	-0.000904
2860.000000	498.335571	2.971316	-0.001301
2861.000000	498.435394	2.972384	-0.001030
2862.000000	498.650665	2.965136	-0.001476
2863.000000	498.825531	2.969065	-0.001060
2864.000000	499.022064	2.965594	-0.000229
2865.000000	499.168976	2.966853	-0.001370
2866.000000	499.279785	2.965097	-0.001438
2867.000000	499.408813	2.962465	-0.001854
2868.000000	499.586548	2.960596	-0.001308
2869.000000	499.799011	2.959833	-0.001087
2870.000000	499.986908	2.959871	-0.001518
2871.000000	500.146240	2.957392	-0.001526
2872.000000	500.230469	2.954226	-0.001221
2873.000000	500.466888	2.955027	-0.000782
2874.000000	500.692719	2.954950	-0.001007
2875.000000	500.831390	2.953119	-0.001015
2876.000000	501.002777	2.950144	-0.001556
2877.000000	501.176544	2.952356	-0.000748
2878.000000	501.335480	2.947550	-0.000381
2879.000000	501.571381	2.950678	-0.000290
2880.000000	501.760620	2.949076	-0.000389
2881.000000	501.884796	2.950144	-0.001656
2882.000000	502.049927	2.945871	-0.001087
2883.000000	502.202087	2.944002	-0.001099
2884.000000	502.436066	2.946711	-0.000778

2885.000000	502.570404	2.944231	-0.000317
2886.000000	502.616150	2.941866	-0.000542
2887.000000	502.837158	2.944841	-0.000763
2888.000000	503.025879	2.943697	-0.000664
2889.000000	503.194366	2.939501	-0.001240
2890.000000	503.357605	2.941217	-0.001312
2891.000000	503.451508	2.939882	-0.000168
2892.000000	503.581482	2.936945	-0.000648
2893.000000	503.741791	2.940798	-0.000530
2894.000000	503.934845	2.937517	-0.000347
2895.000000	504.040771	2.936945	-0.000557
2896.000000	504.299347	2.937136	-0.000401
2897.000000	504.493835	2.938204	-0.000576
2898.000000	504.640717	2.934885	-0.001133
2899.000000	504.826538	2.935190	-0.001091
2900.000000	504.902130	2.932978	-0.000294
2901.000000	505.089386	2.933702	-0.001125
2902.000000	505.246857	2.934160	-0.001183
2903.000000	505.364838	2.928972	-0.001152
2904.000000	505.475586	2.929430	-0.000973
2905.000000	505.655609	2.930307	-0.000843
2906.000000	505.731689	2.928629	-0.001255
2907.000000	505.976807	2.925158	-0.000198
2908.000000	506.128448	2.925730	-0.000118
2909.000000	506.365814	2.930765	-0.000538
2910.000000	506.527527	2.925234	-0.000965

2911.000000	506.623322	2.922716	-0.001297
2912.000000	506.805359	2.924929	-0.000027
2913.000000	506.976807	2.924433	0.000107
2914.000000	507.172729	2.924242	-0.000801
2915.000000	507.338806	2.923594	-0.000809
2916.000000	507.536713	2.921343	-0.000580
2917.000000	507.690765	2.921839	-0.000187
2918.000000	507.865997	2.922220	-0.000252
2919.000000	508.042236	2.922220	-0.000561
2920.000000	508.245392	2.919893	-0.001751
2921.000000	508.416351	2.920198	-0.002014
2922.000000	508.596436	2.914476	-0.000801
2923.000000	508.757690	2.914858	-0.000172
2924.000000	508.950287	2.918558	0.001022
2925.000000	509.204926	2.917299	0.000919
2926.000000	509.270935	2.918367	0.000057
2927.000000	509.457275	2.919550	-0.000729
2928.000000	509.641144	2.917719	-0.001373
2929.000000	509.911743	2.913980	-0.000900
2930.000000	509.954590	2.914286	-0.001125
2931.000000	510.202728	2.916765	-0.000519
2932.000000	510.292267	2.910700	-0.000588
2933.000000	510.492188	2.913179	-0.000145
2934.000000	510.669922	2.913141	0.000332
2935.000000	510.819702	2.914820	-0.000305
2936.000000	510.972443	2.911539	-0.001312

2937.000000	511.225281	2.912454	-0.001133
2938.000000	511.290314	2.907762	-0.000553
2939.000000	511.573578	2.911043	-0.000065
2940.000000	511.688263	2.909479	0.000099
2941.000000	511.825043	2.911272	0.000050
2942.000000	511.963318	2.908143	0.000320
2943.000000	512.116394	2.911958	-0.000435
2944.000000	512.374634	2.910738	-0.001118
2945.000000	512.581299	2.907800	-0.002831
2946.000000	512.755615	2.904634	-0.001808
2947.000000	512.837036	2.900857	-0.000542
2948.000000	513.050354	2.905167	0.000755
2949.000000	513.121216	2.904824	0.000683
2950.000000	513.335571	2.906427	-0.001514
2951.000000	513.497437	2.903642	-0.001831
2952.000000	513.584167	2.898186	-0.001118
2953.000000	513.832214	2.899789	0.000458
2954.000000	513.958862	2.902764	0.001240
2955.000000	514.009949	2.903642	0.000404
2956.000000	514.217102	2.902459	-0.000748
2957.000000	514.455078	2.901963	-0.000977
2958.000000	514.580322	2.899865	-0.000854
2959.000000	514.764771	2.900056	-0.001041
2960.000000	514.969971	2.899140	-0.000294
2961.000000	515.114502	2.897118	0.000156
2962.000000	515.239746	2.899865	-0.000366

2963.000000	515.438660	2.900475	-0.000538
2964.000000	515.572998	2.895631	-0.001598
2965.000000	515.794189	2.896546	-0.000530
2966.000000	515.913574	2.893837	-0.000019
2967.000000	516.043213	2.898721	0.000175
2968.000000	516.254211	2.894448	0.000603
2969.000000	516.407410	2.897118	-0.000191
2970.000000	516.557129	2.897652	0.000568
2971.000000	516.859680	2.896165	-0.000721
2972.000000	516.968018	2.897767	-0.001194
2973.000000	517.049438	2.893456	-0.000938
2974.000000	517.231567	2.893037	-0.000504
2975.000000	517.382324	2.893837	0.000111
2976.000000	517.527771	2.895058	0.000008
2977.000000	517.734863	2.892998	0.000111
2978.000000	517.964233	2.893494	-0.000492
2979.000000	518.110657	2.895173	-0.001312
2980.000000	518.311951	2.891510	-0.001545
2981.000000	518.420349	2.887429	-0.001614
2982.000000	518.624573	2.889641	-0.000633
2983.000000	518.838928	2.888039	-0.000473
2984.000000	518.968994	2.888039	0.000256
2985.000000	519.109619	2.885865	0.000687
2986.000000	519.352905	2.892006	0.000660
2987.000000	519.416992	2.889489	-0.000042
2988.000000	519.593811	2.889527	-0.001144

2989.000000	519.885742	2.886895	-0.000500
2990.000000	519.910217	2.887581	-0.000565
2991.000000	520.130493	2.887963	-0.000843
2992.000000	520.269287	2.886170	-0.000061
2993.000000	520.423767	2.883385	0.000141
2994.000000	520.599243	2.889565	0.000328
2995.000000	520.702759	2.886971	-0.000202
2996.000000	520.943542	2.886017	-0.001518
2997.000000	521.127686	2.884148	-0.000935
2998.000000	521.165771	2.883385	-0.000008
2999.000000	521.401367	2.883614	0.000546
3000.000000	521.509155	2.886246	-0.000633
3001.000000	521.776184	2.885445	0.000290
3002.000000	521.960266	2.879303	0.000095
3003.000000	522.064697	2.888535	-0.000778
3004.000000	522.231628	2.885178	0.000191
3005.000000	522.450439	2.878617	-0.001827
3006.000000	522.566772	2.885216	-0.001480
3007.000000	522.720886	2.879380	-0.000706
3008.000000	522.931641	2.877396	-0.001053
3009.000000	523.130005	2.878998	0.000725
3010.000000	523.276428	2.880143	0.000622
3011.000000	523.467712	2.881630	0.000111
3012.000000	523.616882	2.879189	-0.000023
3013.000000	523.767639	2.880028	-0.000286
3014.000000	523.933105	2.880829	-0.000538

3015.000000	524.078064	2.879380	-0.000751
3016.000000	524.201538	2.876824	-0.000866
3017.000000	524.438049	2.878273	-0.001091
3018.000000	524.595947	2.877053	0.000469
3019.000000	524.744263	2.873810	0.000320
3020.000000	524.934509	2.881402	0.000694
3021.000000	525.081299	2.877701	0.000046
3022.000000	525.231140	2.878579	-0.001255
3023.000000	525.446228	2.875451	-0.000751
3024.000000	525.670837	2.876252	-0.000187
3025.000000	525.800964	2.875107	-0.000103
3026.000000	526.007019	2.877816	-0.000240
3027.000000	526.140930	2.874154	-0.000282
3028.000000	526.318420	2.875527	-0.000648
3029.000000	526.414246	2.874840	0.000061
3030.000000	526.531982	2.874230	0.000042
3031.000000	526.756653	2.875107	0.000648
3032.000000	526.939270	2.875603	0.000000
3033.000000	527.068542	2.877396	-0.000320
3034.000000	527.259277	2.873085	-0.000210
3035.000000	527.347961	2.874764	-0.000172
3036.000000	527.597412	2.875870	0.000420
3037.000000	527.802368	2.875145	-0.000561
3038.000000	527.976013	2.874993	-0.000290
3039.000000	528.090454	2.872399	-0.000546
3040.000000	528.332214	2.875794	-0.000275

3041.000000	528.440918	2.872017	0.000374
3042.000000	528.625061	2.873810	-0.000225
3043.000000	528.799561	2.875260	-0.000252
3044.000000	528.931641	2.873047	-0.000870
3045.000000	529.016968	2.871140	-0.000336
3046.000000	529.233032	2.871521	-0.000366
3047.000000	529.436218	2.874344	-0.000114
3048.000000	529.629761	2.869614	-0.000481
3049.000000	529.738464	2.871521	-0.000359
3050.000000	529.856750	2.870530	0.000034
3051.000000	530.037537	2.872094	-0.000477
3052.000000	530.228760	2.869500	-0.000130
3053.000000	530.430908	2.869652	-0.000183
3054.000000	530.616882	2.871102	0.000515
3055.000000	530.730408	2.870377	0.000389
3056.000000	530.908325	2.871712	0.000160
3057.000000	531.192932	2.871293	-0.000683
3058.000000	531.311279	2.871445	-0.000565
3059.000000	531.514404	2.867096	-0.000626
3060.000000	531.711304	2.870987	-0.000969
3061.000000	531.909180	2.868393	-0.000114
3062.000000	532.053711	2.865952	0.000027
3063.000000	532.195313	2.869042	0.000622
3064.000000	532.371826	2.870797	0.000973
3065.000000	532.506775	2.869080	-0.000221
3066.000000	532.601624	2.870797	-0.000061

3067.000000	532.816223	2.867936	-0.000568
3068.000000	532.971680	2.871064	-0.001411
3069.000000	533.132874	2.866105	-0.001244
3070.000000	533.338379	2.864655	-0.001194
3071.000000	533.501953	2.864922	0.000538
3072.000000	533.525330	2.865685	0.000801
3073.000000	533.795349	2.868279	0.000458
3074.000000	533.971252	2.866982	-0.000652
3075.000000	534.066101	2.866562	-0.000965
3076.000000	534.307495	2.863282	0.000553
3077.000000	534.457642	2.865304	0.000069
3078.000000	534.598816	2.870377	0.001118
3079.000000	534.684143	2.863358	0.000069
3080.000000	534.927307	2.869843	-0.001072
3081.000000	535.049438	2.865914	-0.001038
3082.000000	535.206299	2.863739	-0.002392
3083.000000	535.436707	2.861222	-0.000603
3084.000000	535.651672	2.860230	-0.000252
3085.000000	535.702271	2.864655	0.000198
3086.000000	535.983643	2.860764	0.000126
3087.000000	536.177734	2.861947	-0.000496
3088.000000	536.369385	2.862214	0.000637
3089.000000	536.565369	2.861451	0.000816
3090.000000	536.677979	2.864197	0.000198
3091.000000	536.833862	2.865036	-0.000286
3092.000000	536.952637	2.861413	-0.000622

3093.000000	537.132874	2.861413	-0.001186
3094.000000	537.250183	2.862900	-0.000717
3095.000000	537.456726	2.858361	0.000027
3096.000000	537.715088	2.859353	0.000755
3097.000000	537.822449	2.863320	0.001331
3098.000000	538.006531	2.864197	0.000202
3099.000000	538.175781	2.862595	-0.000751
3100.000000	538.322205	2.860726	0.000114
3101.000000	538.492004	2.861298	-0.000553
3102.000000	538.587341	2.865418	-0.000244
3103.000000	538.800537	2.857483	-0.000469
3104.000000	538.997925	2.861413	-0.000568
3105.000000	539.091858	2.860955	0.000561
3106.000000	539.279785	2.860840	0.000496
3107.000000	539.449036	2.860573	0.000668
3108.000000	539.604980	2.864083	0.000118
3109.000000	539.838623	2.862671	0.000019
3110.000000	540.028015	2.860383	-0.000526
3111.000000	540.169617	2.862519	-0.000313
3112.000000	540.338928	2.861527	0.000145
3113.000000	540.532471	2.860535	-0.000774
3114.000000	540.666504	2.862099	-0.000973
3115.000000	540.812378	2.858361	0.000427
3116.000000	540.942627	2.857750	0.000725
3117.000000	541.106628	2.864846	0.001595
3118.000000	541.277344	2.862481	0.000446

3119.000000	541.391357	2.863968	-0.001434
3120.000000	541.543457	2.860421	-0.000900
3121.000000	541.677979	2.858704	-0.001019
3122.000000	541.942627	2.860611	-0.000244
3123.000000	542.082275	2.858780	-0.000641
3124.000000	542.211121	2.859162	-0.000576
3125.000000	542.409424	2.856225	0.000504
3126.000000	542.593506	2.859009	-0.000034
3127.000000	542.753296	2.861374	-0.000175
3128.000000	542.916382	2.856415	0.000313
3129.000000	543.045654	2.856644	0.000469
3130.000000	543.188660	2.862938	0.000519
3131.000000	543.403320	2.860459	-0.000523
3132.000000	543.524902	2.857102	-0.001205
3133.000000	543.749512	2.856949	-0.000111
3134.000000	543.871094	2.858666	0.000576
3135.000000	544.152893	2.859124	-0.000053
3136.000000	544.336548	2.858895	-0.000401
3137.000000	544.453857	2.856568	-0.000729
3138.000000	544.719910	2.857941	-0.000565
3139.000000	544.852539	2.855958	0.000004
3140.000000	544.985596	2.856377	0.000156
3141.000000	545.218750	2.857369	0.000248
3142.000000	545.413757	2.858017	0.000000
3143.000000	545.582520	2.856377	0.000435
3144.000000	545.735657	2.856873	0.000832

3145.000000	545.902100	2.860116	-0.000099
3146.000000	546.041809	2.860306	0.000114
3147.000000	546.217712	2.854164	-0.000629
3148.000000	546.358948	2.860421	0.000145
3149.000000	546.477600	2.856911	0.000908
3150.000000	546.765686	2.859658	0.000034
3151.000000	546.880615	2.859086	-0.000267
3152.000000	546.999878	2.859505	-0.000320
3153.000000	547.177673	2.855652	-0.000805
3154.000000	547.352295	2.859772	-0.001171
3155.000000	547.578247	2.854928	0.000660
3156.000000	547.687988	2.854012	0.000614
3157.000000	547.842529	2.861832	0.001247
3158.000000	548.029907	2.859391	0.000046
3159.000000	548.167725	2.858475	-0.001041
3160.000000	548.370850	2.855919	-0.000088
3161.000000	548.531555	2.858361	0.000591
3162.000000	548.683167	2.859009	0.001190
3163.000000	548.742798	2.859887	0.000050
3164.000000	548.883484	2.861107	-0.000309
3165.000000	549.093750	2.857560	0.000027
3166.000000	549.268311	2.858628	-0.000393
3167.000000	549.377991	2.861260	-0.000202
3168.000000	549.647461	2.857293	-0.000725
3169.000000	549.745667	2.857216	-0.000423
3170.000000	549.915405	2.857026	0.000542

3171.000000	550.109070	2.859276	0.000446
3172.000000	550.258301	2.858971	-0.000057
3173.000000	550.409485	2.858475	-0.000595
3174.000000	550.652710	2.857140	0.000332
3175.000000	550.806213	2.857216	0.000381
3176.000000	550.901611	2.861260	0.000881
3177.000000	551.093811	2.858323	0.001095
3178.000000	551.225403	2.860993	0.000504
3179.000000	551.464294	2.862824	0.000443
3180.000000	551.578796	2.861527	-0.000538
3181.000000	551.738037	2.860268	-0.001087
3182.000000	551.954529	2.859581	-0.001083
3183.000000	552.124268	2.858361	-0.000427
3184.000000	552.252625	2.857064	0.000141
3185.000000	552.547302	2.859391	0.000980
3186.000000	552.624512	2.859772	0.000420
3187.000000	552.839600	2.861908	0.000187
3188.000000	553.032776	2.857903	0.000362
3189.000000	553.244934	2.861260	-0.000546
3190.000000	553.352295	2.861908	0.000744
3191.000000	553.645508	2.857178	-0.000633
3192.000000	553.858643	2.863663	-0.000454
3193.000000	553.930664	2.857216	0.000435
3194.000000	554.144836	2.859620	-0.000294
3195.000000	554.224915	2.861374	0.000454
3196.000000	554.408447	2.860116	-0.000526

3197.000000	554.545410	2.859238	-0.000191
3198.000000	554.663147	2.858056	0.001007
3199.000000	554.828613	2.861451	0.000401
3200.000000	554.978394	2.864045	0.000298
3201.000000	555.183472	2.858246	-0.001137
3202.000000	555.316528	2.861145	0.000011
3203.000000	555.516785	2.857216	0.001255
3204.000000	555.684143	2.864617	0.000526
3205.000000	555.864868	2.862786	0.000301
3206.000000	556.010803	2.860993	-0.000256
3207.000000	556.175781	2.860535	-0.000355
3208.000000	556.360352	2.864464	0.000057
3209.000000	556.452881	2.859276	-0.000660
3210.000000	556.672241	2.861908	-0.001678
3211.000000	556.797119	2.858513	-0.000706
3212.000000	556.944092	2.856453	-0.000271
3213.000000	557.056152	2.858475	0.000328
3214.000000	557.260254	2.860573	0.000595
3215.000000	557.448486	2.858094	0.000965
3216.000000	557.583557	2.859620	0.000416
3217.000000	557.812439	2.863778	-0.000278
3218.000000	557.899658	2.859810	-0.001083
3219.000000	558.023682	2.856606	-0.001408
3220.000000	558.286011	2.857789	0.000107
3221.000000	558.457153	2.857750	0.000885
3222.000000	558.590637	2.859772	0.000122

3223.000000	558.817627	2.860039	0.000717
3224.000000	559.001282	2.857254	-0.000080
3225.000000	559.159119	2.862595	-0.000015
3226.000000	559.302673	2.858094	0.000725
3227.000000	559.429993	2.859543	0.000248
3228.000000	559.644531	2.862404	-0.000244
3229.000000	559.818176	2.861680	-0.000568
3230.000000	559.934998	2.855805	-0.000122
3231.000000	560.098389	2.860001	0.001003
3232.000000	560.290100	2.862633	0.001068
3233.000000	560.570374	2.863282	-0.000282
3234.000000	560.718567	2.859505	0.000000
3235.000000	560.873840	2.860154	-0.000706
3236.000000	561.091797	2.864197	-0.000229
3237.000000	561.237671	2.857407	-0.001171
3238.000000	561.386780	2.859734	-0.001072
3239.000000	561.486877	2.856530	0.000435
3240.000000	561.658691	2.859276	0.000221
3241.000000	561.891724	2.859810	0.000710
3242.000000	562.056946	2.859200	0.000504
3243.000000	562.125366	2.860116	0.000549
3244.000000	562.322632	2.861641	-0.000229
3245.000000	562.514221	2.861336	-0.000565
3246.000000	562.642090	2.857445	-0.000706
3247.000000	562.852051	2.859391	-0.000553
3248.000000	562.952148	2.859086	-0.000217

3249.000000	563.150391	2.857750	-0.000534
3250.000000	563.271667	2.857178	-0.000015
3251.000000	563.405273	2.857674	0.000912
3252.000000	563.627441	2.859047	0.000515
3253.000000	563.767639	2.861374	0.000389
3254.000000	563.918701	2.857903	-0.000172
3255.000000	564.112122	2.860192	-0.000187
3256.000000	564.223999	2.858780	0.000324
3257.000000	564.409485	2.860001	-0.000305
3258.000000	564.594910	2.859620	0.000137
3259.000000	564.781311	2.858246	0.000645
3260.000000	564.911133	2.860344	0.000385
3261.000000	565.031494	2.862862	-0.000401
3262.000000	565.134277	2.859238	-0.000927
3263.000000	565.320740	2.856797	-0.000385
3264.000000	565.563721	2.858742	-0.000385
3265.000000	565.747803	2.861184	-0.000042
3266.000000	565.900330	2.855118	-0.000103
3267.000000	566.093262	2.858399	-0.000618
3268.000000	566.268311	2.859620	0.000141
3269.000000	566.390625	2.855843	-0.000793
3270.000000	566.544434	2.857102	-0.000336
3271.000000	566.727539	2.855690	0.000633
3272.000000	566.967285	2.858017	0.000980
3273.000000	567.176880	2.858552	0.000599
3274.000000	567.282471	2.860573	-0.000496

3275.000000	567.430176	2.857407	-0.001202
3276.000000	567.635986	2.856110	-0.001225
3277.000000	567.848389	2.854775	0.000248
3278.000000	567.949402	2.855767	0.000816
3279.000000	568.162170	2.858819	0.000607
3280.000000	568.282959	2.858170	0.000008
3281.000000	568.479309	2.856606	-0.000637
3282.000000	568.717590	2.856911	-0.000408
3283.000000	568.845032	2.856263	-0.000816
3284.000000	568.980957	2.856301	-0.000534
3285.000000	569.176819	2.852829	-0.000591
3286.000000	569.302856	2.855958	-0.000568
3287.000000	569.520386	2.853478	0.000008
3288.000000	569.675598	2.853135	0.000912
3289.000000	569.809143	2.854279	0.001568
3290.000000	570.014526	2.860116	0.000782
3291.000000	570.217041	2.857827	0.000065
3292.000000	570.361511	2.855271	-0.000584
3293.000000	570.512146	2.857026	0.000111
3294.000000	570.724548	2.857598	0.000248
3295.000000	570.899719	2.857216	-0.000721
3296.000000	570.987976	2.856415	-0.000687
3297.000000	571.165039	2.854012	-0.001270
3298.000000	571.365723	2.855767	-0.000908
3299.000000	571.558411	2.851189	-0.000149
3300.000000	571.695801	2.853287	0.000568

3301.000000	571.946472	2.854508	0.000832
3302.000000	572.043213	2.856949	0.000275
3303.000000	572.161804	2.853516	-0.000149
3304.000000	572.306702	2.855156	-0.000450
3305.000000	572.531006	2.854661	-0.000271
3306.000000	572.686340	2.854126	-0.000900
3307.000000	572.911560	2.852677	-0.000797
3308.000000	573.106995	2.851647	-0.000061
3309.000000	573.261292	2.851914	0.000752
3310.000000	573.350159	2.854203	0.000896
3311.000000	573.540466	2.855156	-0.000038
3312.000000	573.731689	2.854508	-0.001381
3313.000000	573.783081	2.851571	-0.001267
3314.000000	573.927612	2.849091	-0.000469
3315.000000	574.046143	2.851532	0.000813
3316.000000	574.208984	2.852181	0.001690
3317.000000	574.496582	2.854088	0.000637
3318.000000	574.604187	2.856263	-0.000309
3319.000000	574.704773	2.852677	-0.000393
3320.000000	574.881348	2.851341	-0.000359
3321.000000	575.069214	2.854584	-0.000187
3322.000000	575.244873	2.853516	-0.000340
3323.000000	575.356750	2.850655	-0.001480
3324.000000	575.531982	2.851609	-0.001801
3325.000000	575.700500	2.848137	-0.001164
3326.000000	575.809082	2.845772	0.000328

3327.000000	576.048523	2.847755	0.001232
3328.000000	576.234985	2.853440	0.001560
3329.000000	576.498840	2.850464	0.000946
3330.000000	576.576355	2.852219	-0.001019
3331.000000	576.796814	2.853096	-0.000839
3332.000000	576.967285	2.847031	-0.000660
3333.000000	577.103210	2.848862	-0.000797
3334.000000	577.253906	2.851036	0.000328
3335.000000	577.491272	2.847107	0.000309
3336.000000	577.599854	2.849549	0.000214
3337.000000	577.807678	2.851151	0.000992
3338.000000	577.951660	2.850083	-0.000225
3339.000000	578.164612	2.851799	-0.001160
3340.000000	578.370056	2.848099	-0.000828
3341.000000	578.531494	2.846344	-0.000057
3342.000000	578.662720	2.848671	-0.000381
3343.000000	578.901184	2.851227	0.000286
3344.000000	579.079163	2.843750	0.000481
3345.000000	579.207092	2.850235	-0.000278
3346.000000	579.440796	2.851570	0.000858
3347.000000	579.565430	2.845924	-0.000954
3348.000000	579.763245	2.850197	-0.000320
3349.000000	579.931396	2.846153	0.000378
3350.000000	580.071350	2.849854	-0.000252
3351.000000	580.325684	2.847985	0.000076
3352.000000	580.384583	2.848022	-0.000389

3353.000000	580.520142	2.847450	0.000793
3354.000000	580.624939	2.848175	-0.000511
3355.000000	580.775940	2.851876	-0.000896
3356.000000	580.912476	2.843254	-0.001339
3357.000000	581.082092	2.845428	-0.001221
3358.000000	581.233154	2.844704	0.000122
3359.000000	581.335510	2.845047	0.000309
3360.000000	581.569763	2.844055	-0.000134
3361.000000	581.720215	2.847298	-0.000038
3362.000000	581.849304	2.842911	-0.000179
3363.000000	581.912415	2.845429	-0.000065
3364.000000	582.146667	2.844093	0.000713
3365.000000	582.304626	2.846382	0.000290
3366.000000	582.488831	2.846001	-0.000084
3367.000000	582.719788	2.845924	-0.000187
3368.000000	582.881958	2.843903	-0.000660
3369.000000	583.058289	2.846497	-0.000294
3370.000000	583.228516	2.842415	0.000130
3371.000000	583.457092	2.845200	0.000210
3372.000000	583.659485	2.845200	0.000401
3373.000000	583.804932	2.846153	-0.000446
3374.000000	584.006836	2.843941	-0.000362
3375.000000	584.227966	2.843597	-0.001194
3376.000000	584.374756	2.844666	-0.001080
3377.000000	584.522949	2.839821	-0.000835
3378.000000	584.668396	2.840431	-0.000225

3379.000000	584.754883	2.841537	0.001072
3380.000000	584.982544	2.842682	-0.000031
3381.000000	585.109680	2.844055	-0.000526
3382.000000	585.223755	2.839020	-0.001026
3383.000000	585.448608	2.840736	-0.000805
3384.000000	585.590332	2.839211	0.001057
3385.000000	585.768005	2.839935	0.000694
3386.000000	585.936279	2.844704	0.000633
3387.000000	586.088623	2.841461	-0.000256
3388.000000	586.357971	2.841614	-0.000950
3389.000000	586.489807	2.840202	-0.000080
3390.000000	586.661316	2.840584	0.000237
3391.000000	586.853943	2.841576	-0.000008
3392.000000	586.974121	2.842110	-0.000790
3393.000000	587.245239	2.839401	-0.000332
3394.000000	587.375244	2.837723	0.000530
3395.000000	587.483276	2.842110	0.000347
3396.000000	587.735718	2.843407	0.000145
3397.000000	587.931580	2.838295	-0.000732
3398.000000	587.982544	2.840355	0.000031
3399.000000	588.232666	2.839973	0.000221
3400.000000	588.376160	2.842720	0.000206
3401.000000	588.595459	2.838219	-0.000313
3402.000000	588.748291	2.842262	-0.000904
3403.000000	588.889099	2.838638	-0.000198
3404.000000	589.004517	2.837990	-0.001369

3405.000000	589.152710	2.839363	-0.000469
3406.000000	589.305115	2.835052	-0.000221
3407.000000	589.461731	2.837761	0.000713
3408.000000	589.659058	2.837685	0.000591
3409.000000	589.714661	2.841614	0.000092
3410.000000	589.937256	2.836082	-0.001312
3411.000000	590.120667	2.839020	-0.001297
3412.000000	590.326599	2.832420	-0.000275
3413.000000	590.444580	2.836960	-0.000118
3414.000000	590.686340	2.835739	0.000652
3415.000000	590.844482	2.836769	-0.000599
3416.000000	590.975281	2.835777	-0.000504
3417.000000	591.172119	2.833946	-0.000511
3418.000000	591.278381	2.834633	0.000008
3419.000000	591.428040	2.834785	-0.000275
3420.000000	591.659424	2.835396	0.000244
3421.000000	591.768494	2.832191	0.000488
3422.000000	591.872314	2.837151	-0.000134
3423.000000	592.121155	2.836349	-0.000557
3424.000000	592.219910	2.832649	-0.001095
3425.000000	592.399292	2.831657	-0.000259
3426.000000	592.556458	2.834023	-0.000065
3427.000000	592.735901	2.834366	0.000565
3428.000000	592.933228	2.830971	-0.000210
3429.000000	593.051270	2.836006	0.000271
3430.000000	593.250000	2.832153	0.001194

3431.000000	593.431885	2.835129	0.000080
3432.000000	593.553711	2.837379	-0.000736
3433.000000	593.759521	2.833794	-0.000969
3434.000000	593.929016	2.829140	-0.001671
3435.000000	594.124512	2.834404	-0.000729
3436.000000	594.304810	2.828720	0.000610
3437.000000	594.495117	2.830360	-0.000031
3438.000000	594.717529	2.834213	0.000206
3439.000000	594.874268	2.831505	-0.000420
3440.000000	595.070679	2.829178	-0.000362
3441.000000	595.265625	2.830780	0.000298
3442.000000	595.366699	2.832764	0.000568
3443.000000	595.582397	2.831200	-0.000385
3444.000000	595.711304	2.831810	-0.001095
3445.000000	595.895935	2.829330	-0.001434
3446.000000	596.075867	2.828224	-0.000923
3447.000000	596.192505	2.825821	0.000019
3448.000000	596.388367	2.828949	0.001087
3449.000000	596.444580	2.829063	0.001003
3450.000000	596.682068	2.832039	0.000282
3451.000000	596.866211	2.829292	0.000065
3452.000000	596.995544	2.830246	-0.000504
3453.000000	597.228821	2.830284	-0.000259
3454.000000	597.352539	2.829025	0.000099
3455.000000	597.489929	2.828606	0.000340
3456.000000	597.645203	2.831581	-0.000141

3457.000000	597.758057	2.830704	-0.000690
3458.000000	597.940308	2.827271	-0.001408
3459.000000	598.085754	2.827309	-0.000202
3460.000000	598.360596	2.826241	-0.000015
3461.000000	598.450806	2.830208	-0.000393
3462.000000	598.683960	2.825745	-0.000149
3463.000000	598.811462	2.825592	-0.001221
3464.000000	598.955872	2.827805	-0.000305
3465.000000	599.078186	2.823074	-0.000042
3466.000000	599.231201	2.825478	-0.000332
3467.000000	599.346375	2.826546	0.001343
3468.000000	599.454468	2.824409	0.000835
3469.000000	599.634888	2.830322	0.000580
3470.000000	599.852051	2.827766	0.000416

[TA Ascii File]

TGA-51H

File Name : C:\TAData\TGA Data\ISMAILi\LDH-425- AC.txt

[File Information]

File Name: LDH-425- AC.tad

Sample Name: LDH-425- AC

Lot No: LDH-425- AC

Acquire Date: 25/05/21

Acquire Time: 00:36:29(-0800)

Detector Type: TGA-51H

Detector Serial No:

Operator:

Atmosphere: Air

Gas Flow: 0[ml/min]

Annotation:

Pan Name: Aluminum

Sample Weight: 9.210[mg]

[Measurements Program]

[Temperature Program]

Start Temp.

Temp. Rate Hold Temp. Hold Time

[C/min] [C] [min]

10.00 600.0 0

[Valve Program]

V1: 0

V2: 0

V1 V2 Direction Hold Temp. Hold Time

[C] [min]

- Repeat Program -

Init. Final Count

[PID Program]

P : 10

I : 10

D : 10

Temp. Direction P I D

[C]

- Repeat Program -

Init. Final Count

[Data]

Time Temp TGA

sec	C	mg
0.000000	27.851341	9.202609
1.000000	27.852139	9.203334
2.000000	27.873602	9.211345
3.000000	27.875988	9.209933
4.000000	27.802055	9.212985
5.000000	27.933220	9.211612
6.000000	27.941967	9.213977
7.000000	27.970581	9.214549
8.000000	27.916527	9.214854
9.000000	27.921297	9.213977
10.000000	27.935604	9.212604
11.000000	27.934013	9.212832
12.000000	27.955475	9.217563
13.000000	28.076305	9.212489
14.000000	28.014303	9.212070
15.000000	27.895859	9.216914
16.000000	27.853729	9.214625
17.000000	27.871216	9.217219
18.000000	27.906988	9.216723
19.000000	28.064383	9.218707
20.000000	28.160564	9.220309
21.000000	28.180437	9.213367
22.000000	28.264696	9.211612
23.000000	28.290136	9.211764
24.000000	28.375988	9.214702

25.000000	28.496019	9.215922
26.000000	28.393478	9.216952
27.000000	28.537354	9.214091
28.000000	28.479324	9.213939
29.000000	28.352139	9.215922
30.000000	28.406195	9.215846
31.000000	28.635124	9.212947
32.000000	28.680437	9.215655
33.000000	28.633537	9.212718
34.000000	28.750393	9.215541
35.000000	28.681231	9.215503
36.000000	28.782982	9.214473
37.000000	28.845779	9.213290
38.000000	28.910164	9.213710
39.000000	28.978527	9.212718
40.000000	29.070740	9.212260
41.000000	28.920498	9.211955
42.000000	28.990454	9.216113
43.000000	28.972170	9.213786
44.000000	29.084251	9.210773
45.000000	29.078690	9.211116
46.000000	29.098562	9.209704
47.000000	29.152615	9.210315
48.000000	29.073919	9.210429
49.000000	29.251976	9.210315
50.000000	29.205873	9.211917

51.000000	29.404602	9.211993
52.000000	29.398245	9.210849
53.000000	29.323524	9.215503
54.000000	29.356112	9.209476
55.000000	29.414141	9.214396
56.000000	29.522249	9.209056
57.000000	29.569153	9.212298
58.000000	29.605715	9.209819
59.000000	29.691566	9.211345
60.000000	29.829086	9.210353
61.000000	29.860882	9.209094
62.000000	29.895065	9.210315
63.000000	29.959015	9.206958
64.000000	30.014761	9.209018
65.000000	30.004324	9.209704
66.000000	30.086147	9.209323
67.000000	30.014990	9.208369
68.000000	30.135376	9.213023
69.000000	30.179222	9.210277
70.000000	30.379995	9.208674
71.000000	30.300762	9.209552
72.000000	30.403841	9.207988
73.000000	30.389221	9.210086
74.000000	30.538452	9.211421
75.000000	30.610764	9.205089
76.000000	30.575375	9.207911

77.000000	30.620760	9.207263
78.000000	30.806915	9.207110
79.000000	30.992296	9.206538
80.000000	30.958452	9.207339
81.000000	30.907686	9.209590
82.000000	31.075378	9.207873
83.000000	31.154608	9.210086
84.000000	31.287685	9.204783
85.000000	31.219225	9.206347
86.000000	31.372301	9.206309
87.000000	31.429226	9.208674
88.000000	31.607683	9.210505
89.000000	31.751532	9.207721
90.000000	31.874609	9.213634
91.000000	31.975374	9.207797
92.000000	32.217682	9.206729
93.000000	32.231529	9.206882
94.000000	32.391529	9.208636
95.000000	32.603062	9.206347
96.000000	32.692295	9.206157
97.000000	32.779221	9.205394
98.000000	32.819992	9.205737
99.000000	32.954605	9.208865
100.000000	33.114605	9.208674
101.000000	33.349991	9.205966
102.000000	33.439224	9.207721

103.000000	33.468452	9.210620
104.000000	33.673065	9.208293
105.000000	34.045376	9.205775
106.000000	34.204605	9.203219
107.000000	34.286144	9.203982
108.000000	34.449219	9.209170
109.000000	34.608452	9.205699
110.000000	34.712296	9.204974
111.000000	34.948456	9.206614
112.000000	35.080761	9.203410
113.000000	35.388451	9.204097
114.000000	35.523834	9.202418
115.000000	35.666142	9.203906
116.000000	35.887680	9.202304
117.000000	36.136913	9.200587
118.000000	36.385376	9.202838
119.000000	36.569988	9.201427
120.000000	36.678452	9.202189
121.000000	36.898453	9.202418
122.000000	37.188446	9.200702
123.000000	37.396915	9.199252
124.000000	37.649990	9.200816
125.000000	37.803066	9.203067
126.000000	38.071526	9.197040
127.000000	38.229992	9.197726
128.000000	38.506145	9.196391

129.000000	38.737679	9.198489
130.000000	38.881523	9.198642
131.000000	39.193069	9.191508
132.000000	39.441528	9.193149
133.000000	39.556911	9.193263
134.000000	39.809219	9.194369
135.000000	40.182552	9.193988
136.000000	40.379272	9.193530
137.000000	40.509674	9.189067
138.000000	40.732479	9.187121
139.000000	40.939629	9.190364
140.000000	41.084194	9.185672
141.000000	41.216087	9.186625
142.000000	41.521599	9.185443
143.000000	41.725769	9.185328
144.000000	41.951553	9.184107
145.000000	42.184040	9.180521
146.000000	42.367352	9.177469
147.000000	42.517872	9.181131
148.000000	42.632622	9.179873
149.000000	42.844254	9.173426
150.000000	43.071526	9.177393
151.000000	43.259304	9.173006
152.000000	43.365864	9.171251
153.000000	43.695965	9.173082
154.000000	43.841270	9.169573

155.000000	44.081207	9.171442
156.000000	44.312950	9.167780
157.000000	44.563324	9.164003
158.000000	44.807739	9.164690
159.000000	45.040222	9.162134
160.000000	45.267502	9.159426
161.000000	45.361385	9.158281
162.000000	45.553638	9.157785
163.000000	45.847229	9.158129
164.000000	45.887470	9.157862
165.000000	46.091644	9.152178
166.000000	46.292091	9.151377
167.000000	46.551407	9.148973
168.000000	46.695965	9.148821
169.000000	46.866604	9.145502
170.000000	46.929943	9.146303
171.000000	47.141571	9.147791
172.000000	47.259304	9.145311
173.000000	47.374054	9.139895
174.000000	47.530540	9.137568
175.000000	47.789856	9.138331
176.000000	47.980614	9.137073
177.000000	48.093132	9.142336
178.000000	48.309227	9.135890
179.000000	48.400887	9.133143
180.000000	48.657959	9.131312

181.000000	48.827858	9.127650
182.000000	48.954529	9.129672
183.000000	49.149017	9.127040
184.000000	49.182552	9.126353
185.000000	49.418762	9.122920
186.000000	49.497753	9.123454
187.000000	49.660950	9.118152
188.000000	49.776440	9.119678
189.000000	49.988003	9.117846
190.000000	50.010147	9.115939
191.000000	50.135010	9.113688
192.000000	50.292404	9.112735
193.000000	50.462082	9.113536
194.000000	50.532837	9.109874
195.000000	50.751617	9.105487
196.000000	50.892406	9.107242
197.000000	50.992771	9.108958
198.000000	51.041866	9.103236
199.000000	51.191326	9.103961
200.000000	51.348724	9.101863
201.000000	51.522736	9.096828
202.000000	51.699627	9.094119
203.000000	51.848362	9.096828
204.000000	51.979046	9.091525
205.000000	52.028149	9.090686
206.000000	52.172550	9.088931

207.000000	52.198540	9.090419
208.000000	52.389160	9.088855
209.000000	52.664970	9.086680
210.000000	52.624535	9.087291
211.000000	52.810814	9.086070
212.000000	52.922009	9.079585
213.000000	53.035362	9.079318
214.000000	53.240421	9.080005
215.000000	53.325623	9.082408
216.000000	53.451969	9.073214
217.000000	53.568218	9.073824
218.000000	53.888073	9.069932
219.000000	54.012253	9.067490
220.000000	54.065689	9.062531
221.000000	54.323448	9.066574
222.000000	54.571823	9.068138
223.000000	54.657749	9.064972
224.000000	54.846924	9.060852
225.000000	55.024536	9.058487
226.000000	55.255585	9.057343
227.000000	55.355217	9.058487
228.000000	55.514069	9.055702
229.000000	55.677246	9.055855
230.000000	55.873638	9.051315
231.000000	55.936447	9.048264
232.000000	56.113346	9.050743

233.000000	56.379044	9.045937
234.000000	56.503235	9.046547
235.000000	56.722008	9.039604
236.000000	56.919842	9.041512
237.000000	57.005039	9.044144
238.000000	57.231754	9.038918
239.000000	57.424534	9.035522
240.000000	57.657024	9.033997
241.000000	57.796371	9.031670
242.000000	58.037529	9.032509
243.000000	58.275799	9.028275
244.000000	58.514061	9.027168
245.000000	58.641857	9.024384
246.000000	58.862804	9.019577
247.000000	58.971825	9.018700
248.000000	59.218033	9.013893
249.000000	59.332836	9.015915
250.000000	59.516953	9.014198
251.000000	59.680126	9.011223
252.000000	59.812176	9.008514
253.000000	60.047581	9.003632
254.000000	60.308880	9.002831
255.000000	60.397850	8.996498
256.000000	60.617779	9.000313
257.000000	60.904613	8.995163
258.000000	61.006390	8.996002

259.000000	61.212082	8.995087
260.000000	61.357986	8.991081
261.000000	61.551586	8.989632
262.000000	61.760124	8.986389
263.000000	61.947319	8.985054
264.000000	62.192871	8.980858
265.000000	62.316711	8.983070
266.000000	62.520275	8.981430
267.000000	62.676853	8.978607
268.000000	62.842693	8.973190
269.000000	63.003544	8.975517
270.000000	63.154434	8.974335
271.000000	63.394291	8.966782
272.000000	63.641266	8.965103
273.000000	63.726677	8.962509
274.000000	63.842693	8.961403
275.000000	63.948742	8.956635
276.000000	64.113152	8.952477
277.000000	64.307457	8.950417
278.000000	64.470444	8.950760
279.000000	64.617767	8.950493
280.000000	64.743744	8.945000
281.000000	64.925957	8.944084
282.000000	65.127380	8.942749
283.000000	65.308868	8.941185
284.000000	65.463318	8.934700

285.000000	65.598557	8.934547
286.000000	65.797852	8.933784
287.000000	65.994995	8.935272
288.000000	66.085388	8.927834
289.000000	66.339485	8.923790
290.000000	66.518837	8.925163
291.000000	66.809937	8.921272
292.000000	66.949440	8.920624
293.000000	67.098206	8.913567
294.000000	67.266174	8.912079
295.000000	67.477554	8.909447
296.000000	67.585030	8.909599
297.000000	67.759415	8.905823
298.000000	67.951576	8.903267
299.000000	68.072586	8.903992
300.000000	68.288239	8.903076
301.000000	68.437706	8.894989
302.000000	68.518127	8.893311
303.000000	68.709587	8.895790
304.000000	68.967957	8.888809
305.000000	69.126671	8.888924
306.000000	69.148026	8.887817
307.000000	69.271156	8.881790
308.000000	69.573639	8.879807
309.000000	69.626312	8.880531
310.000000	69.664749	8.877975

311.000000	69.922821	8.873550
312.000000	70.138268	8.868782
313.000000	70.238403	8.873894
314.000000	70.449570	8.870003
315.000000	70.583763	8.863670
316.000000	70.683899	8.865768
317.000000	70.863739	8.861229
318.000000	71.128723	8.859741
319.000000	71.228867	8.857414
320.000000	71.491814	8.853600
321.000000	71.542213	8.853485
322.000000	71.679138	8.851540
323.000000	71.784721	8.849098
324.000000	72.078316	8.851234
325.000000	72.108971	8.839826
326.000000	72.250664	8.841927
327.000000	72.516327	8.839980
328.000000	72.566742	8.839103
329.000000	72.877365	8.835781
330.000000	72.976135	8.834560
331.000000	73.241806	8.828800
332.000000	73.337845	8.828037
333.000000	73.517014	8.826855
334.000000	73.680496	8.827961
335.000000	73.888939	8.820141
336.000000	74.004066	8.818729

337.000000	74.131447	8.820980
338.000000	74.341942	8.817509
339.000000	74.431175	8.816708
340.000000	74.643036	8.808964
341.000000	74.764969	8.806179
342.000000	74.871918	8.807323
343.000000	75.171638	8.805950
344.000000	75.269051	8.803051
345.000000	75.395752	8.797176
346.000000	75.702301	8.799809
347.000000	75.905296	8.796413
348.000000	75.968643	8.797482
349.000000	76.192078	8.793476
350.000000	76.380081	8.789814
351.000000	76.511566	8.789165
352.000000	76.820816	8.788288
353.000000	76.997925	8.784092
354.000000	77.136223	8.782413
355.000000	77.347389	8.781422
356.000000	77.446167	8.779858
357.000000	77.564697	8.774097
358.000000	77.743164	8.771961
359.000000	78.017685	8.769176
360.000000	78.138260	8.768909
361.000000	78.318787	8.770283
362.000000	78.499298	8.767384

363.000000	78.615105	8.766582
364.000000	78.869873	8.760098
365.000000	79.019043	8.757771
366.000000	79.218651	8.755405
367.000000	79.402557	8.754452
368.000000	79.486359	8.749111
369.000000	79.576950	8.746555
370.000000	79.849403	8.746212
371.000000	80.017021	8.744038
372.000000	80.099976	8.744038
373.000000	80.262573	8.741749
374.000000	80.449944	8.739803
375.000000	80.631279	8.736599
376.000000	80.792450	8.736752
377.000000	80.901253	8.735607
378.000000	81.127586	8.732670
379.000000	81.206825	8.725651
380.000000	81.376068	8.725422
381.000000	81.494263	8.724162
382.000000	81.688354	8.723933
383.000000	82.002663	8.719584
384.000000	82.183998	8.719928
385.000000	82.364655	8.716189
386.000000	82.480164	8.713214
387.000000	82.631279	8.713328
388.000000	82.814621	8.713214

389.000000	82.989906	8.705852
390.000000	83.273315	8.706996
391.000000	83.475464	8.704974
392.000000	83.550682	8.698222
393.000000	83.750153	8.700282
394.000000	83.887146	8.700053
395.000000	84.065125	8.693149
396.000000	84.339134	8.694713
397.000000	84.423073	8.690555
398.000000	84.595680	8.689868
399.000000	84.858948	8.689029
400.000000	84.934166	8.686206
401.000000	85.120193	8.682277
402.000000	85.276001	8.682658
403.000000	85.458672	8.678271
404.000000	85.656792	8.678386
405.000000	85.841484	8.675563
406.000000	86.022804	8.676059
407.000000	86.218918	8.674457
408.000000	86.398895	8.666751
409.000000	86.519798	8.669726
410.000000	86.696411	8.663699
411.000000	86.909309	8.666522
412.000000	87.055717	8.661448
413.000000	87.164520	8.657252
414.000000	87.301529	8.663013

415.000000	87.482178	8.654010
416.000000	87.676270	8.654887
417.000000	87.896545	8.650805
418.000000	88.080566	8.646991
419.000000	88.243095	8.652141
420.000000	88.341141	8.647296
421.000000	88.469421	8.644168
422.000000	88.614487	8.641421
423.000000	88.770966	8.639667
424.000000	88.933487	8.638370
425.000000	89.034225	8.634593
426.000000	89.255852	8.631198
427.000000	89.359955	8.630626
428.000000	89.650085	8.630359
429.000000	89.770958	8.629405
430.000000	89.987671	8.625743
431.000000	90.148323	8.622958
432.000000	90.331108	8.622806
433.000000	90.417854	8.621509
434.000000	90.621826	8.618953
435.000000	90.820496	8.615863
436.000000	90.866196	8.616282
437.000000	91.010574	8.609912
438.000000	91.203293	8.608653
439.000000	91.360901	8.604416
440.000000	91.603287	8.605638

441.000000	91.733734	8.604379
442.000000	91.852943	8.599075
443.000000	92.001961	8.597969
444.000000	92.227806	8.600906
445.000000	92.478119	8.597321
446.000000	92.511238	8.592972
447.000000	92.627792	8.592743
448.000000	92.799965	8.593048
449.000000	93.052307	8.592209
450.000000	93.205940	8.589691
451.000000	93.543022	8.588127
452.000000	93.582092	8.582748
453.000000	93.840363	8.581032
454.000000	93.943016	8.578629
455.000000	94.229790	8.573288
456.000000	94.387390	8.576111
457.000000	94.552956	8.576797
458.000000	94.714539	8.571915
459.000000	94.880768	8.570541
460.000000	95.096008	8.570389
461.000000	95.220512	8.567757
462.000000	95.391365	8.563560
463.000000	95.517197	8.559975
464.000000	95.740372	8.558258
465.000000	95.980774	8.560051
466.000000	96.125153	8.554939

467.000000	96.294678	8.553566
468.000000	96.371506	8.551315
469.000000	96.571503	8.550323
470.000000	96.750969	8.551506
471.000000	96.901955	8.544945
472.000000	97.022499	8.541206
473.000000	97.182098	8.538956
474.000000	97.358246	8.542274
475.000000	97.482094	8.540291
476.000000	97.598648	8.537468
477.000000	97.870163	8.533005
478.000000	98.086731	8.532738
479.000000	98.198669	8.530487
480.000000	98.330444	8.527435
481.000000	98.441032	8.528961
482.000000	98.543686	8.527817
483.000000	98.706589	8.525185
484.000000	98.880096	8.522629
485.000000	99.074821	8.518700
486.000000	99.207275	8.518509
487.000000	99.356270	8.517441
488.000000	99.613876	8.513359
489.000000	99.867508	8.515076
490.000000	100.024231	8.512405
491.000000	100.152679	8.511108
492.000000	100.452942	8.508362

493.000000	100.609528	8.506645
494.000000	100.681679	8.500275
495.000000	100.829865	8.499817
496.000000	100.981934	8.497719
497.000000	101.119820	8.493561
498.000000	101.373047	8.494133
499.000000	101.572144	8.494362
500.000000	101.680389	8.488487
501.000000	101.861450	8.492950
502.000000	102.101776	8.486885
503.000000	102.250618	8.488602
504.000000	102.398178	8.484711
505.000000	102.547653	8.484444
506.000000	102.615303	8.480247
507.000000	102.934250	8.477005
508.000000	103.035408	8.476471
509.000000	103.158478	8.472961
510.000000	103.341461	8.477234
511.000000	103.476135	8.472618
512.000000	103.719040	8.469223
513.000000	103.954865	8.466286
514.000000	104.126251	8.469681
515.000000	104.244171	8.464722
516.000000	104.527679	8.463501
517.000000	104.580513	8.463234
518.000000	104.728706	8.459114

519.000000	104.963875	8.454613
520.000000	105.077278	8.453773
521.000000	105.275742	8.454803
522.000000	105.449066	8.453697
523.000000	105.485802	8.449425
524.000000	105.648804	8.446182
525.000000	105.838242	8.448090
526.000000	106.000618	8.445839
527.000000	106.153328	8.444923
528.000000	106.222260	8.439545
529.000000	106.528961	8.437294
530.000000	106.633995	8.440155
531.000000	106.854355	8.435081
532.000000	106.971626	8.435234
533.000000	107.158470	8.433594
534.000000	107.322777	8.432869
535.000000	107.484505	8.426575
536.000000	107.585663	8.423294
537.000000	107.833092	8.422264
538.000000	108.016083	8.422417
539.000000	108.278328	8.418640
540.000000	108.445213	8.419594
541.000000	108.539925	8.418106
542.000000	108.660408	8.415245
543.000000	108.994820	8.414444
544.000000	109.196495	8.409142

545.000000	109.251266	8.407578
546.000000	109.438110	8.408722
547.000000	109.568268	8.407196
548.000000	109.842743	8.405708
549.000000	109.965240	8.402008
550.000000	110.143723	8.405251
551.000000	110.261742	8.401627
552.000000	110.367828	8.398575
553.000000	110.679184	8.400482
554.000000	110.858101	8.394875
555.000000	110.988045	8.394073
556.000000	111.123627	8.391518
557.000000	111.350250	8.387398
558.000000	111.399231	8.387779
559.000000	111.585655	8.385033
560.000000	111.743225	8.385223
561.000000	111.983650	8.386902
562.000000	112.128647	8.381104
563.000000	112.324509	8.379044
564.000000	112.455078	8.372022
565.000000	112.600098	8.380798
566.000000	112.771469	8.373737
567.000000	113.011887	8.372211
568.000000	113.129913	8.368587
569.000000	113.307564	8.366566
570.000000	113.414291	8.363743

571.000000	113.558662	8.368702
572.000000	113.831734	8.359814
573.000000	114.057098	8.359928
574.000000	114.108582	8.360958
575.000000	114.340218	8.356571
576.000000	114.455711	8.352489
577.000000	114.485214	8.353939
578.000000	114.693626	8.353672
579.000000	114.877563	8.348827
580.000000	115.130524	8.344707
581.000000	115.322006	8.344326
582.000000	115.494629	8.345852
583.000000	115.635262	8.342228
584.000000	115.885712	8.339596
585.000000	115.977997	8.339367
586.000000	116.215294	8.333988
587.000000	116.396065	8.334332
588.000000	116.584396	8.331051
589.000000	116.752647	8.329983
590.000000	116.917732	8.325672
591.000000	117.167572	8.326626
592.000000	117.239136	8.327274
593.000000	117.480194	8.324947
594.000000	117.557419	8.321781
595.000000	117.736328	8.321857
596.000000	117.866890	8.317280

597.000000	118.006882	8.313694
598.000000	118.187653	8.315601
599.000000	118.391060	8.314495
600.000000	118.596321	8.309994
601.000000	118.652832	8.308697
602.000000	118.910828	8.307858
603.000000	119.134933	8.302937
604.000000	119.326401	8.303661
605.000000	119.497772	8.299580
606.000000	119.618919	8.299122
607.000000	119.814781	8.301907
608.000000	119.988243	8.295116
609.000000	120.166527	8.295383
610.000000	120.322571	8.294964
611.000000	120.562195	8.289127
612.000000	120.692223	8.285809
613.000000	120.783875	8.286877
614.000000	120.965294	8.288479
615.000000	120.990059	8.285503
616.000000	121.178291	8.282681
617.000000	121.370872	8.283443
618.000000	121.477364	8.281841
619.000000	121.674896	8.281422
620.000000	121.885422	8.277569
621.000000	121.993774	8.273449
622.000000	122.180771	8.271618

623.000000	122.319481	8.272915
624.000000	122.555389	8.265018
625.000000	122.674278	8.267612
626.000000	122.853844	8.266468
627.000000	122.974571	8.257732
628.000000	123.264366	8.262081
629.000000	123.395020	8.260670
630.000000	123.484177	8.254832
631.000000	123.621643	8.256778
632.000000	123.766541	8.256167
633.000000	123.928146	8.251513
634.000000	124.133102	8.254146
635.000000	124.300903	8.248195
636.000000	124.474884	8.249606
637.000000	124.740524	8.244685
638.000000	124.889122	8.240947
639.000000	125.006775	8.238467
640.000000	125.276756	8.242930
641.000000	125.412964	8.239993
642.000000	125.550423	8.238887
643.000000	125.669930	8.238467
644.000000	125.809235	8.237552
645.000000	125.990059	8.235263
646.000000	126.212349	8.232249
647.000000	126.318855	8.231715
648.000000	126.432785	8.227786

649.000000	126.755394	8.228587
650.000000	126.934959	8.225421
651.000000	127.162193	8.223132
652.000000	127.360962	8.224391
653.000000	127.473640	8.218822
654.000000	127.601830	8.218249
655.000000	127.973335	8.216037
656.000000	128.068680	8.219432
657.000000	128.238342	8.212756
658.000000	128.328125	8.213557
659.000000	128.540527	8.207911
660.000000	128.647644	8.208255
661.000000	128.891602	8.205089
662.000000	129.120087	8.206386
663.000000	129.286011	8.204059
664.000000	129.520081	8.202800
665.000000	129.629059	8.200168
666.000000	129.774521	8.199939
667.000000	129.991089	8.193568
668.000000	130.136215	8.196506
669.000000	130.368546	8.191928
670.000000	130.487122	8.188876
671.000000	130.600815	8.188724
672.000000	130.831863	8.186130
673.000000	130.997528	8.186893
674.000000	131.133194	8.183459

675.000000	131.297012	8.184985
676.000000	131.445557	8.181781
677.000000	131.611816	8.181705
678.000000	131.722458	8.177699
679.000000	131.924759	8.177165
680.000000	132.039673	8.177623
681.000000	132.234070	8.173045
682.000000	132.373413	8.170489
683.000000	132.523804	8.172130
684.000000	132.595932	8.168200
685.000000	132.813538	8.167819
686.000000	132.946777	8.163318
687.000000	133.136887	8.163089
688.000000	133.385040	8.163890
689.000000	133.557434	8.160686
690.000000	133.710846	8.157596
691.000000	133.866089	8.158282
692.000000	134.065979	8.155192
693.000000	134.180283	8.153628
694.000000	134.308640	8.152141
695.000000	134.547653	8.151950
696.000000	134.612427	8.147067
697.000000	134.741394	8.152408
698.000000	134.896667	8.151111
699.000000	135.120972	8.143023
700.000000	135.262802	8.147716

701.000000	135.414368	8.141726
702.000000	135.699234	8.138634
703.000000	135.970612	8.137299
704.000000	136.145432	8.135963
705.000000	136.284790	8.140466
706.000000	136.391159	8.134743
707.000000	136.611206	8.133789
708.000000	136.851425	8.132072
709.000000	137.063538	8.129593
710.000000	137.148499	8.127342
711.000000	137.310486	8.129402
712.000000	137.571472	8.123756
713.000000	137.659485	8.124977
714.000000	137.787247	8.123032
715.000000	137.966354	8.119446
716.000000	138.142380	8.117195
717.000000	138.276855	8.121391
718.000000	138.401550	8.116203
719.000000	138.641159	8.111931
720.000000	138.793350	8.112617
721.000000	138.861206	8.111740
722.000000	139.079437	8.111130
723.000000	139.248734	8.107544
724.000000	139.504852	8.106895
725.000000	139.661942	8.106514
726.000000	139.844696	8.102928

727.000000	139.917236	8.102242
728.000000	140.114014	8.099762
729.000000	140.355408	8.099915
730.000000	140.482773	8.098541
731.000000	140.605270	8.096176
732.000000	140.700027	8.096939
733.000000	140.856323	8.093468
734.000000	140.951675	8.090073
735.000000	141.138153	8.093697
736.000000	141.342743	8.091103
737.000000	141.413956	8.088013
738.000000	141.534668	8.086258
739.000000	141.760986	8.085609
740.000000	141.915466	8.086716
741.000000	142.046432	8.079773
742.000000	142.203339	8.081985
743.000000	142.392853	8.081947
744.000000	142.564850	8.078705
745.000000	142.678894	8.081451
746.000000	142.959534	8.073135
747.000000	143.022888	8.078400
748.000000	143.179199	8.070694
749.000000	143.334290	8.072983
750.000000	143.550949	8.070923
751.000000	143.716919	8.071495
752.000000	143.950470	8.070274

753.000000	144.170135	8.066841
754.000000	144.342148	8.063408
755.000000	144.439911	8.061844
756.000000	144.665619	8.062263
757.000000	144.770035	8.063103
758.000000	144.893127	8.057800
759.000000	145.027710	8.059517
760.000000	145.272141	8.056770
761.000000	145.399475	8.052345
762.000000	145.621552	8.054024
763.000000	145.789322	8.049942
764.000000	146.005997	8.050972
765.000000	146.190674	8.051544
766.000000	146.468872	8.050591
767.000000	146.558807	8.049713
768.000000	146.678284	8.046089
769.000000	146.869598	8.048492
770.000000	147.050659	8.043228
771.000000	147.241348	8.042046
772.000000	147.389816	8.042694
773.000000	147.670471	8.039757
774.000000	147.780273	8.036705
775.000000	147.922729	8.033501
776.000000	148.077209	8.033195
777.000000	148.275146	8.034454
778.000000	148.432663	8.032166

779.000000	148.706665	8.030373
780.000000	148.853317	8.031441
781.000000	149.057907	8.028198
782.000000	149.284805	8.026443
783.000000	149.470703	8.028656
784.000000	149.625793	8.023773
785.000000	149.775452	8.022400
786.000000	149.876770	8.020912
787.000000	150.046310	8.016563
788.000000	150.077881	8.023201
789.000000	150.352371	8.016220
790.000000	150.481781	8.016830
791.000000	150.761444	8.012863
792.000000	150.852676	8.012672
793.000000	151.046463	8.011642
794.000000	151.274857	8.013016
795.000000	151.362503	8.010574
796.000000	151.584335	8.006645
797.000000	151.750702	8.008362
798.000000	151.895599	8.009697
799.000000	152.103699	8.004852
800.000000	152.299927	8.002945
801.000000	152.370850	8.005692
802.000000	152.606995	8.002106
803.000000	152.712540	7.998024
804.000000	152.928986	7.995430

805.000000	153.082245	7.996689
806.000000	153.268890	7.996002
807.000000	153.336273	7.991806
808.000000	153.509186	7.992912
809.000000	153.697037	7.992950
810.000000	153.887268	7.988029
811.000000	153.964172	7.987495
812.000000	154.057800	7.989822
813.000000	154.256958	7.990433
814.000000	154.409622	7.987457
815.000000	154.586121	7.982422
816.000000	154.810333	7.983376
817.000000	154.939728	7.983490
818.000000	155.056015	7.981316
819.000000	155.262329	7.980209
820.000000	155.437637	7.979561
821.000000	155.580750	7.977768
822.000000	155.799011	7.976089
823.000000	155.851471	7.976509
824.000000	156.109085	7.977463
825.000000	156.180038	7.971245
826.000000	156.441818	7.974678
827.000000	156.572403	7.972771
828.000000	156.689880	7.972427
829.000000	156.874741	7.967735
830.000000	157.020203	7.963272

831.000000	157.185989	7.966286
832.000000	157.310013	7.966667
833.000000	157.534241	7.964264
834.000000	157.630859	7.962814
835.000000	157.827026	7.963501
836.000000	157.929611	7.957932
837.000000	158.189575	7.958885
838.000000	158.359528	7.957817
839.000000	158.492493	7.959114
840.000000	158.707169	7.955299
841.000000	158.813309	7.957283
842.000000	158.987427	7.953812
843.000000	159.225357	7.955032
844.000000	159.391129	7.951180
845.000000	159.710175	7.954994
846.000000	159.832382	7.949005
847.000000	160.000107	7.947975
848.000000	160.019699	7.951065
849.000000	160.346619	7.945877
850.000000	160.573242	7.947021
851.000000	160.569122	7.945610
852.000000	160.818085	7.939163
853.000000	160.945816	7.941910
854.000000	161.162979	7.940422
855.000000	161.318970	7.939163
856.000000	161.463760	7.939011

857.000000	161.686249	7.936722
858.000000	161.859283	7.936264
859.000000	162.052338	7.934319
860.000000	162.194183	7.933861
861.000000	162.319550	7.929283
862.000000	162.467896	7.932182
863.000000	162.715652	7.929474
864.000000	162.919922	7.931915
865.000000	163.051758	7.928406
866.000000	163.215958	7.929398
867.000000	163.388412	7.924591
868.000000	163.562637	7.922913
869.000000	163.663300	7.924438
870.000000	163.802185	7.923294
871.000000	164.053528	7.917114
872.000000	164.153580	7.919289
873.000000	164.338989	7.919937
874.000000	164.506149	7.914978
875.000000	164.617966	7.919479
876.000000	164.870483	7.916618
877.000000	165.007599	7.911544
878.000000	165.208328	7.913604
879.000000	165.338394	7.913146
880.000000	165.381958	7.915550
881.000000	165.600296	7.907615
882.000000	165.830444	7.913185

883.000000	166.006424	7.905248
884.000000	166.120026	7.905018
885.000000	166.426086	7.907500
886.000000	166.469055	7.906774
887.000000	166.666824	7.904562
888.000000	166.796906	7.903302
889.000000	166.972290	7.901738
890.000000	167.188293	7.899067
891.000000	167.361328	7.899411
892.000000	167.532028	7.899106
893.000000	167.780396	7.899029
894.000000	167.950516	7.898190
895.000000	168.024689	7.897313
896.000000	168.287766	7.897809
897.000000	168.416687	7.893574
898.000000	168.565002	7.893612
899.000000	168.799835	7.892888
900.000000	168.915771	7.888310
901.000000	169.056458	7.885373
902.000000	169.196533	7.883770
903.000000	169.358398	7.886250
904.000000	169.616791	7.887890
905.000000	169.740402	7.886746
906.000000	169.929810	7.885945
907.000000	170.054459	7.884686
908.000000	170.243500	7.884800

909.000000	170.389389	7.883809
910.000000	170.560547	7.881520
911.000000	170.624878	7.877743
912.000000	170.883362	7.877400
913.000000	171.133789	7.878125
914.000000	171.210159	7.877705
915.000000	171.437622	7.875836
916.000000	171.631775	7.875302
917.000000	171.740906	7.876751
918.000000	171.920685	7.872059
919.000000	172.134338	7.871525
920.000000	172.275665	7.871296
921.000000	172.361801	7.871602
922.000000	172.553650	7.867253
923.000000	172.842560	7.869122
924.000000	173.001099	7.867558
925.000000	173.151016	7.868092
926.000000	173.313568	7.864125
927.000000	173.425568	7.867215
928.000000	173.593292	7.870114
929.000000	173.847748	7.869046
930.000000	173.970642	7.862599
931.000000	174.114822	7.861073
932.000000	174.234314	7.860882
933.000000	174.446823	7.862256
934.000000	174.541016	7.859051

935.000000	174.710480	7.856686
936.000000	174.812698	7.857029
937.000000	175.048767	7.857449
938.000000	175.171127	7.854702
939.000000	175.265900	7.854626
940.000000	175.474380	7.852261
941.000000	175.654175	7.853444
942.000000	175.854630	7.851346
943.000000	175.946533	7.848904
944.000000	176.098175	7.845737
945.000000	176.332520	7.846615
946.000000	176.520340	7.852414
947.000000	176.631210	7.850087
948.000000	176.752380	7.845356
949.000000	176.999390	7.848446
950.000000	177.130341	7.843754
951.000000	177.306671	7.846158
952.000000	177.578918	7.843945
953.000000	177.799500	7.845966
954.000000	177.914368	7.839100
955.000000	178.078064	7.842991
956.000000	178.235458	7.838146
957.000000	178.372131	7.844212
958.000000	178.574905	7.841236
959.000000	178.774796	7.838757
960.000000	178.912643	7.837421

961.000000	179.079803	7.834637
962.000000	179.190063	7.834064
963.000000	179.344589	7.834713
964.000000	179.473251	7.833950
965.000000	179.776520	7.835896
966.000000	179.979858	7.833416
967.000000	180.020065	7.827427
968.000000	180.183289	7.833683
969.000000	180.320648	7.827236
970.000000	180.499374	7.830059
971.000000	180.676956	7.826931
972.000000	180.902832	7.829563
973.000000	181.077530	7.823917
974.000000	181.293640	7.825634
975.000000	181.390182	7.825481
976.000000	181.590195	7.822506
977.000000	181.866623	7.820446
978.000000	181.899948	7.820332
979.000000	182.140167	7.820522
980.000000	182.353409	7.819645
981.000000	182.474670	7.817204
982.000000	182.551086	7.819683
983.000000	182.791336	7.817966
984.000000	182.948227	7.817738
985.000000	183.158569	7.816975
986.000000	183.353394	7.817966

987.000000	183.429825	7.815830
988.000000	183.543060	7.815067
989.000000	183.739029	7.812473
990.000000	183.937317	7.812969
991.000000	184.110886	7.812550
992.000000	184.254547	7.812283
993.000000	184.475235	7.811863
994.000000	184.618912	7.809383
995.000000	184.880997	7.811062
996.000000	185.051102	7.810986
997.000000	185.199356	7.811367
998.000000	185.374084	7.805874
999.000000	185.543640	7.807705
1000.000000	185.682144	7.803776
1001.000000	185.814880	7.806522
1002.000000	186.014893	7.805225
1003.000000	186.138458	7.801830
1004.000000	186.264328	7.803852
1005.000000	186.475800	7.799541
1006.000000	186.593628	7.802708
1007.000000	186.797073	7.803471
1008.000000	186.921204	7.798740
1009.000000	187.014343	7.799618
1010.000000	187.295349	7.795193
1011.000000	187.421219	7.794125
1012.000000	187.604553	7.795803

1013.000000	187.745926	7.795307
1014.000000	187.850525	7.793285
1015.000000	188.080978	7.791912
1016.000000	188.241333	7.791531
1017.000000	188.365479	7.796261
1018.000000	188.531555	7.787678
1019.000000	188.586166	7.791798
1020.000000	188.793640	7.792904
1021.000000	189.042480	7.792141
1022.000000	189.166626	7.792904
1023.000000	189.386169	7.786304
1024.000000	189.583878	7.788898
1025.000000	189.757996	7.783901
1026.000000	189.870636	7.790882
1027.000000	190.020691	7.784130
1028.000000	190.241150	7.781841
1029.000000	190.370560	7.784206
1030.000000	190.577698	7.781727
1031.000000	190.788254	7.782337
1032.000000	190.863159	7.778294
1033.000000	191.061264	7.781498
1034.000000	191.209351	7.781689
1035.000000	191.392700	7.777264
1036.000000	191.568054	7.775928
1037.000000	191.659424	7.779667
1038.000000	191.802460	7.778255

1039.000000	192.001648	7.776577
1040.000000	192.206543	7.776958
1041.000000	192.408569	7.779133
1042.000000	192.468750	7.775928
1043.000000	192.673050	7.774632
1044.000000	192.754761	7.774670
1045.000000	193.022644	7.773335
1046.000000	193.219025	7.772457
1047.000000	193.459641	7.773563
1048.000000	193.606079	7.770702
1049.000000	193.789978	7.769558
1050.000000	193.919937	7.772495
1051.000000	194.077148	7.770092
1052.000000	194.252502	7.770397
1053.000000	194.388138	7.772038
1054.000000	194.546509	7.766468
1055.000000	194.776917	7.767422
1056.000000	194.924469	7.766964
1057.000000	195.103226	7.765705
1058.000000	195.256485	7.764140
1059.000000	195.400070	7.763302
1060.000000	195.559555	7.762309
1061.000000	195.746246	7.763492
1062.000000	195.938095	7.763912
1063.000000	196.048187	7.765629
1064.000000	196.286545	7.760021

1065.000000	196.581665	7.755404
1066.000000	196.611755	7.762042
1067.000000	196.827438	7.757846
1068.000000	196.985764	7.760211
1069.000000	197.275772	7.755328
1070.000000	197.378479	7.754489
1071.000000	197.491440	7.758075
1072.000000	197.572601	7.754909
1073.000000	197.779724	7.759028
1074.000000	198.017548	7.752505
1075.000000	198.184982	7.755099
1076.000000	198.385315	7.755557
1077.000000	198.543640	7.751781
1078.000000	198.781448	7.756358
1079.000000	198.951721	7.750865
1080.000000	199.133316	7.753268
1081.000000	199.229233	7.752086
1082.000000	199.426163	7.750407
1083.000000	199.639557	7.750941
1084.000000	199.783722	7.750712
1085.000000	199.989594	7.745334
1086.000000	200.078979	7.745677
1087.000000	200.183350	7.748157
1088.000000	200.370117	7.746020
1089.000000	200.528275	7.745944
1090.000000	200.754852	7.743960

1091.000000	200.839539	7.741862
1092.000000	201.022949	7.743350
1093.000000	201.179443	7.743617
1094.000000	201.308975	7.743655
1095.000000	201.463211	7.741023
1096.000000	201.631470	7.742740
1097.000000	201.764404	7.746173
1098.000000	201.998260	7.739726
1099.000000	202.118866	7.744037
1100.000000	202.327484	7.737514
1101.000000	202.515381	7.736445
1102.000000	202.750931	7.738238
1103.000000	202.892822	7.738391
1104.000000	203.032486	7.736331
1105.000000	203.194016	7.734347
1106.000000	203.331421	7.735606
1107.000000	203.398697	7.737971
1108.000000	203.586029	7.737056
1109.000000	203.781219	7.736484
1110.000000	203.973572	7.736941
1111.000000	204.114929	7.733241
1112.000000	204.246719	7.732211
1113.000000	204.380768	7.732440
1114.000000	204.591644	7.729426
1115.000000	204.845718	7.728129
1116.000000	204.919739	7.727977

1117.000000	205.061646	7.731029
1118.000000	205.243347	7.730075
1119.000000	205.330292	7.729922
1120.000000	205.564713	7.730418
1121.000000	205.808716	7.727328
1122.000000	205.946671	7.726146
1123.000000	206.091919	7.729465
1124.000000	206.317398	7.728320
1125.000000	206.373459	7.726413
1126.000000	206.628662	7.725574
1127.000000	206.781219	7.725421
1128.000000	206.962372	7.723933
1129.000000	207.090790	7.722293
1130.000000	207.352173	7.722713
1131.000000	207.431244	7.719737
1132.000000	207.596680	7.723437
1133.000000	207.752609	7.720729
1134.000000	207.962341	7.718249
1135.000000	208.096405	7.723018
1136.000000	208.270813	7.718516
1137.000000	208.417236	7.716685
1138.000000	208.607910	7.717563
1139.000000	208.768311	7.720004
1140.000000	208.906830	7.715007
1141.000000	209.120499	7.718287
1142.000000	209.261322	7.715121

1143.000000	209.491791	7.715007
1144.000000	209.631470	7.715999
1145.000000	209.736343	7.714129
1146.000000	210.001221	7.715770
1147.000000	210.083099	7.716495
1148.000000	210.257263	7.716189
1149.000000	210.370422	7.713519
1150.000000	210.541809	7.711726
1151.000000	210.757584	7.713061
1152.000000	210.872940	7.711459
1153.000000	211.070374	7.709132
1154.000000	211.219009	7.707416
1155.000000	211.444748	7.708140
1156.000000	211.554581	7.707377
1157.000000	211.705414	7.710391
1158.000000	211.886780	7.703906
1159.000000	212.171890	7.708484
1160.000000	212.283371	7.706195
1161.000000	212.409836	7.708140
1162.000000	212.512985	7.705127
1163.000000	212.803040	7.710238
1164.000000	212.911194	7.705623
1165.000000	213.097549	7.707225
1166.000000	213.390396	7.703296
1167.000000	213.498016	7.702838
1168.000000	213.649963	7.702495

1169.000000	213.850189	7.706233
1170.000000	213.976089	7.703906
1171.000000	214.129166	7.701045
1172.000000	214.282242	7.698871
1173.000000	214.572327	7.695895
1174.000000	214.626114	7.701312
1175.000000	214.824112	7.697497
1176.000000	214.972778	7.700511
1177.000000	215.181854	7.699519
1178.000000	215.389832	7.702914
1179.000000	215.582886	7.697383
1180.000000	215.778091	7.696353
1181.000000	215.883469	7.696620
1182.000000	216.036545	7.694789
1183.000000	216.261734	7.696887
1184.000000	216.418701	7.694331
1185.000000	216.556778	7.692576
1186.000000	216.803604	7.691699
1187.000000	216.918961	7.695094
1188.000000	217.083130	7.691432
1189.000000	217.282806	7.691508
1190.000000	217.511322	7.691737
1191.000000	217.807495	7.689029
1192.000000	217.904556	7.685138
1193.000000	218.059860	7.689982
1194.000000	218.239517	7.687121

1195.000000	218.324951	7.689143
1196.000000	218.555115	7.689334
1197.000000	218.628342	7.685061
1198.000000	218.869598	7.687236
1199.000000	218.961105	7.686168
1200.000000	219.122528	7.683650
1201.000000	219.321060	7.686549
1202.000000	219.474167	7.682429
1203.000000	219.700989	7.686511
1204.000000	219.823547	7.685023
1205.000000	220.039307	7.684833
1206.000000	220.095276	7.683230
1207.000000	220.299683	7.679530
1208.000000	220.399918	7.684337
1209.000000	220.603806	7.678500
1210.000000	220.747330	7.678195
1211.000000	220.904587	7.681094
1212.000000	221.086517	7.682239
1213.000000	221.162155	7.682010
1214.000000	221.315002	7.681132
1215.000000	221.511154	7.679339
1216.000000	221.683228	7.675142
1217.000000	221.878845	7.677660
1218.000000	222.054199	7.680255
1219.000000	222.107880	7.677508
1220.000000	222.344040	7.676516

1221.000000	222.508423	7.674837
1222.000000	222.653076	7.673501
1223.000000	222.747360	7.671403
1224.000000	222.994446	7.675676
1225.000000	223.213074	7.674532
1226.000000	223.358292	7.677051
1227.000000	223.565430	7.672357
1228.000000	223.659683	7.668313
1229.000000	223.868454	7.675447
1230.000000	224.019104	7.668198
1231.000000	224.168152	7.668961
1232.000000	224.359955	7.669916
1233.000000	224.500214	7.671403
1234.000000	224.691986	7.668198
1235.000000	224.860764	7.667321
1236.000000	224.988419	7.672204
1237.000000	225.235031	7.668694
1238.000000	225.406509	7.666634
1239.000000	225.573090	7.670144
1240.000000	225.678314	7.667474
1241.000000	225.918274	7.664727
1242.000000	226.026245	7.665833
1243.000000	226.152832	7.661713
1244.000000	226.396118	7.668923
1245.000000	226.591156	7.666749
1246.000000	226.751724	7.663048

1247.000000	226.885986	7.661980
1248.000000	227.109543	7.666596
1249.000000	227.203217	7.663697
1250.000000	227.367615	7.660264
1251.000000	227.557190	7.665223
1252.000000	227.602661	7.658738
1253.000000	227.896378	7.662781
1254.000000	228.131439	7.657555
1255.000000	228.231705	7.659424
1256.000000	228.396088	7.658089
1257.000000	228.614182	7.657174
1258.000000	228.837769	7.656830
1259.000000	229.007080	7.658089
1260.000000	229.077759	7.656296
1261.000000	229.204315	7.661141
1262.000000	229.501282	7.656373
1263.000000	229.676651	7.655152
1264.000000	229.774170	7.656449
1265.000000	229.953384	7.656716
1266.000000	230.099182	7.652253
1267.000000	230.288864	7.648858
1268.000000	230.359589	7.649468
1269.000000	230.600830	7.656640
1270.000000	230.868896	7.654007
1271.000000	231.106323	7.650460
1272.000000	231.202774	7.651947

1273.000000	231.345306	7.648552
1274.000000	231.454437	7.649888
1275.000000	231.646332	7.649773
1276.000000	231.862350	7.651261
1277.000000	232.007080	7.646149
1278.000000	232.166626	7.644356
1279.000000	232.255417	7.650002
1280.000000	232.431946	7.650002
1281.000000	232.630951	7.648057
1282.000000	232.776779	7.642144
1283.000000	232.966476	7.645272
1284.000000	233.132050	7.646302
1285.000000	233.285034	7.642754
1286.000000	233.492798	7.641648
1287.000000	233.729660	7.646035
1288.000000	233.900696	7.640275
1289.000000	234.083267	7.639855
1290.000000	234.221970	7.638482
1291.000000	234.334930	7.641533
1292.000000	234.452240	7.641877
1293.000000	234.642471	7.639550
1294.000000	234.861221	7.643174
1295.000000	235.043259	7.639283
1296.000000	235.239502	7.641572
1297.000000	235.361252	7.642029
1298.000000	235.582703	7.638329

1299.000000	235.743912	7.638978
1300.000000	235.820129	7.635087
1301.000000	235.996674	7.634629
1302.000000	236.183609	7.636345
1303.000000	236.389175	7.633523
1304.000000	236.527893	7.638520
1305.000000	236.617249	7.637223
1306.000000	236.857925	7.634171
1307.000000	237.043259	7.636231
1308.000000	237.167160	7.630623
1309.000000	237.346436	7.636422
1310.000000	237.485123	7.632226
1311.000000	237.627136	7.632073
1312.000000	237.878220	7.631463
1313.000000	238.090393	7.630738
1314.000000	238.169327	7.631386
1315.000000	238.282288	7.628945
1316.000000	238.378784	7.629746
1317.000000	238.571747	7.629517
1318.000000	238.741150	7.629136
1319.000000	238.873291	7.626656
1320.000000	239.036667	7.626656
1321.000000	239.242249	7.625702
1322.000000	239.387543	7.627305
1323.000000	239.547653	7.627800
1324.000000	239.728027	7.623490

1325.000000	239.894135	7.626580
1326.000000	240.037201	7.624215
1327.000000	240.181946	7.624825
1328.000000	240.324966	7.622994
1329.000000	240.535156	7.621773
1330.000000	240.686279	7.623566
1331.000000	240.868820	7.623414
1332.000000	240.986389	7.621621
1333.000000	241.116379	7.620171
1334.000000	241.334717	7.623261
1335.000000	241.523773	7.624672
1336.000000	241.666763	7.619790
1337.000000	241.881851	7.618226
1338.000000	242.051407	7.618645
1339.000000	242.163513	7.619561
1340.000000	242.358002	7.615746
1341.000000	242.569275	7.618035
1342.000000	242.631027	7.618187
1343.000000	242.860733	7.616356
1344.000000	243.027588	7.614716
1345.000000	243.192261	7.612236
1346.000000	243.292480	7.614411
1347.000000	243.427902	7.611321
1348.000000	243.647278	7.614754
1349.000000	243.801651	7.611588
1350.000000	244.015106	7.609948

1351.000000	244.276215	7.614869
1352.000000	244.467987	7.613762
1353.000000	244.595276	7.611588
1354.000000	244.785400	7.610558
1355.000000	244.991821	7.609337
1356.000000	245.057877	7.608155
1357.000000	245.322800	7.608918
1358.000000	245.512939	7.609337
1359.000000	245.748016	7.610634
1360.000000	245.809814	7.605103
1361.000000	246.030807	7.605408
1362.000000	246.193863	7.603615
1363.000000	246.456589	7.604798
1364.000000	246.586609	7.608613
1365.000000	246.668396	7.607544
1366.000000	246.914352	7.606743
1367.000000	247.072525	7.606553
1368.000000	247.295166	7.602471
1369.000000	247.356934	7.601975
1370.000000	247.625626	7.604836
1371.000000	247.800049	7.602204
1372.000000	247.966324	7.596825
1373.000000	248.178711	7.600068
1374.000000	248.351501	7.599839
1375.000000	248.530273	7.601555
1376.000000	248.707947	7.599343

1377.000000	248.810318	7.602204
1378.000000	248.902954	7.597474
1379.000000	249.124527	7.595833
1380.000000	249.266449	7.598389
1381.000000	249.465256	7.598198
1382.000000	249.596924	7.594613
1383.000000	249.754532	7.595376
1384.000000	249.924118	7.594002
1385.000000	250.086182	7.594269
1386.000000	250.288818	7.596177
1387.000000	250.453827	7.596367
1388.000000	250.609802	7.592972
1389.000000	250.869156	7.594574
1390.000000	250.906143	7.590378
1391.000000	251.102295	7.591256
1392.000000	251.314499	7.589272
1393.000000	251.470459	7.588242
1394.000000	251.600708	7.587098
1395.000000	251.747528	7.590569
1396.000000	251.928116	7.589196
1397.000000	252.075485	7.588013
1398.000000	252.207336	7.589386
1399.000000	252.385788	7.586525
1400.000000	252.509048	7.590226
1401.000000	252.667160	7.583970
1402.000000	252.832733	7.585000

1403.000000	253.055145	7.583817
1404.000000	253.177872	7.583588
1405.000000	253.358978	7.583626
1406.000000	253.509048	7.583626
1407.000000	253.618912	7.579125
1408.000000	253.743774	7.580651
1409.000000	253.830078	7.582444
1410.000000	254.043350	7.580346
1411.000000	254.221817	7.577141
1412.000000	254.390625	7.580880
1413.000000	254.552979	7.574357
1414.000000	254.748062	7.580079
1415.000000	254.842911	7.578896
1416.000000	255.066376	7.577103
1417.000000	255.250214	7.578896
1418.000000	255.419556	7.576531
1419.000000	255.565887	7.577981
1420.000000	255.769501	7.572564
1421.000000	255.995087	7.577256
1422.000000	256.019226	7.573517
1423.000000	256.207855	7.576264
1424.000000	256.447388	7.571343
1425.000000	256.592102	7.574128
1426.000000	256.742126	7.574433
1427.000000	256.952759	7.570847
1428.000000	257.097992	7.568139

1429.000000	257.255005	7.572564
1430.000000	257.497772	7.567795
1431.000000	257.618347	7.569893
1432.000000	257.737305	7.572526
1433.000000	257.977905	7.566422
1434.000000	258.137085	7.568482
1435.000000	258.310730	7.566384
1436.000000	258.428101	7.568863
1437.000000	258.555664	7.566155
1438.000000	258.797852	7.564629
1439.000000	258.853088	7.564744
1440.000000	258.981140	7.560699
1441.000000	259.188538	7.564438
1442.000000	259.342346	7.562874
1443.000000	259.556183	7.563828
1444.000000	259.750732	7.561310
1445.000000	259.921692	7.559479
1446.000000	260.006866	7.558640
1447.000000	260.208801	7.561539
1448.000000	260.460541	7.558868
1449.000000	260.625000	7.560166
1450.000000	260.723511	7.559975
1451.000000	260.997223	7.557686
1452.000000	261.233459	7.559708
1453.000000	261.358246	7.555016
1454.000000	261.597656	7.556084

1455.000000	261.807617	7.554672
1456.000000	261.919037	7.556885
1457.000000	262.188995	7.558640
1458.000000	262.370026	7.552307
1459.000000	262.496429	7.555321
1460.000000	262.693573	7.552994
1461.000000	262.949005	7.549065
1462.000000	263.055634	7.552116
1463.000000	263.253265	7.550934
1464.000000	263.440765	7.552956
1465.000000	263.503418	7.549942
1466.000000	263.718719	7.549866
1467.000000	264.027740	7.547691
1468.000000	264.009552	7.547615
1469.000000	264.321289	7.545898
1470.000000	264.363617	7.547615
1471.000000	264.560699	7.547310
1472.000000	264.685547	7.545593
1473.000000	264.869781	7.542923
1474.000000	265.085602	7.543114
1475.000000	265.258667	7.545135
1476.000000	265.341125	7.546394
1477.000000	265.491089	7.543724
1478.000000	265.674805	7.539795
1479.000000	265.852081	7.541664
1480.000000	265.917419	7.537621

1481.000000	266.135437	7.539452
1482.000000	266.298828	7.542503
1483.000000	266.485718	7.535103
1484.000000	266.666748	7.536858
1485.000000	266.884766	7.536285
1486.000000	266.965088	7.536133
1487.000000	267.132202	7.537964
1488.000000	267.243622	7.533234
1489.000000	267.429474	7.533730
1490.000000	267.614258	7.533043
1491.000000	267.686584	7.531212
1492.000000	267.940491	7.530983
1493.000000	268.043823	7.529419
1494.000000	268.306793	7.530907
1495.000000	268.289673	7.534874
1496.000000	268.531250	7.530296
1497.000000	268.754028	7.529800
1498.000000	268.841919	7.529457
1499.000000	269.000458	7.529343
1500.000000	269.273621	7.524651
1501.000000	269.439667	7.523659
1502.000000	269.479309	7.526520
1503.000000	269.648010	7.523811
1504.000000	269.810822	7.520988
1505.000000	269.970367	7.523506
1506.000000	270.119659	7.521523

1507.000000	270.239960	7.521294
1508.000000	270.385193	7.521255
1509.000000	270.573303	7.519043
1510.000000	270.635315	7.522018
1511.000000	270.912476	7.517365
1512.000000	271.053986	7.512596
1513.000000	271.199707	7.516754
1514.000000	271.397369	7.520302
1515.000000	271.535706	7.515457
1516.000000	271.678802	7.511948
1517.000000	271.903442	7.512329
1518.000000	272.021118	7.513168
1519.000000	272.205017	7.512444
1520.000000	272.370361	7.512825
1521.000000	272.616211	7.513626
1522.000000	272.785797	7.514084
1523.000000	272.909821	7.511032
1524.000000	273.085266	7.515076
1525.000000	273.331146	7.511719
1526.000000	273.490112	7.510155
1527.000000	273.676147	7.508514
1528.000000	273.786865	7.510155
1529.000000	273.970245	7.508209
1530.000000	274.132385	7.503822
1531.000000	274.348602	7.506836
1532.000000	274.447754	7.502563

1533.000000	274.651215	7.505463
1534.000000	274.844666	7.502258
1535.000000	275.095306	7.497414
1536.000000	275.227783	7.501228
1537.000000	275.442444	7.503860
1538.000000	275.519287	7.502373
1539.000000	275.764099	7.503059
1540.000000	275.958618	7.500122
1541.000000	276.068817	7.500389
1542.000000	276.324768	7.492149
1543.000000	276.450897	7.496841
1544.000000	276.659180	7.496155
1545.000000	276.791138	7.494781
1546.000000	276.985046	7.493446
1547.000000	277.171082	7.496574
1548.000000	277.285522	7.495430
1549.000000	277.501221	7.490738
1550.000000	277.731781	7.491081
1551.000000	277.846771	7.489632
1552.000000	278.007324	7.489479
1553.000000	278.224091	7.489670
1554.000000	278.429718	7.486504
1555.000000	278.614624	7.485130
1556.000000	278.771515	7.484062
1557.000000	278.974457	7.484673
1558.000000	279.158905	7.487228

1559.000000	279.268585	7.481926
1560.000000	279.419098	7.487228
1561.000000	279.570679	7.477615
1562.000000	279.634796	7.480782
1563.000000	279.769928	7.477844
1564.000000	279.953003	7.478798
1565.000000	280.130981	7.477386
1566.000000	280.283997	7.478912
1567.000000	280.504120	7.476929
1568.000000	280.647705	7.475822
1569.000000	280.784515	7.474525
1570.000000	280.878876	7.473190
1571.000000	280.968475	7.472313
1572.000000	281.126740	7.471741
1573.000000	281.216370	7.472389
1574.000000	281.427612	7.470787
1575.000000	281.531891	7.469833
1576.000000	281.706940	7.473801
1577.000000	281.863647	7.471703
1578.000000	281.991516	7.470291
1579.000000	282.238892	7.467049
1580.000000	282.492065	7.463692
1581.000000	282.485229	7.466019
1582.000000	282.637238	7.466133
1583.000000	282.890900	7.464722
1584.000000	282.989441	7.466286

1585.000000	283.287659	7.460106
1586.000000	283.379395	7.458733
1587.000000	283.552338	7.462357
1588.000000	283.625732	7.460182
1589.000000	283.776672	7.461784
1590.000000	283.977905	7.457817
1591.000000	284.174469	7.453659
1592.000000	284.412903	7.456329
1593.000000	284.626740	7.455376
1594.000000	284.705902	7.454994
1595.000000	284.928101	7.452744
1596.000000	285.026642	7.452400
1597.000000	285.279785	7.455070
1598.000000	285.394043	7.446258
1599.000000	285.597900	7.450912
1600.000000	285.736816	7.449501
1601.000000	285.958008	7.450912
1602.000000	286.095825	7.449005
1603.000000	286.311279	7.450188
1604.000000	286.470581	7.446526
1605.000000	286.604218	7.448318
1606.000000	286.706970	7.446945
1607.000000	286.961151	7.444654
1608.000000	287.056519	7.445915
1609.000000	287.242065	7.443548
1610.000000	287.446472	7.442289

1611.000000	287.579590	7.440725
1612.000000	287.846344	7.440114
1613.000000	287.946991	7.438054
1614.000000	288.193848	7.438398
1615.000000	288.339539	7.437292
1616.000000	288.593201	7.436947
1617.000000	288.678589	7.436108
1618.000000	288.882019	7.431416
1619.000000	289.038696	7.433247
1620.000000	289.206421	7.430768
1621.000000	289.434418	7.429623
1622.000000	289.653473	7.427754
1623.000000	289.750977	7.426724
1624.000000	289.864166	7.429013
1625.000000	290.023499	7.427296
1626.000000	290.263916	7.427563
1627.000000	290.436768	7.426800
1628.000000	290.708160	7.425084
1629.000000	290.877869	7.422299
1630.000000	291.014618	7.421383
1631.000000	291.149719	7.423977
1632.000000	291.295380	7.422871
1633.000000	291.442017	7.414708
1634.000000	291.617523	7.416920
1635.000000	291.820801	7.419095
1636.000000	291.961151	7.416729

1637.000000	292.095245	7.414059
1638.000000	292.250366	7.417683
1639.000000	292.424744	7.416234
1640.000000	292.603882	7.412076
1641.000000	292.792480	7.414441
1642.000000	292.863190	7.409520
1643.000000	293.062256	7.410130
1644.000000	293.194794	7.407231
1645.000000	293.466644	7.406811
1646.000000	293.465088	7.407116
1647.000000	293.706024	7.403340
1648.000000	293.867371	7.403607
1649.000000	294.064850	7.402882
1650.000000	294.241943	7.404179
1651.000000	294.410034	7.403683
1652.000000	294.599182	7.400517
1653.000000	294.731201	7.401661
1654.000000	294.943359	7.397542
1655.000000	295.054932	7.399334
1656.000000	295.168091	7.399487
1657.000000	295.373413	7.399678
1658.000000	295.594971	7.392430
1659.000000	295.759979	7.394528
1660.000000	295.863708	7.393803
1661.000000	296.119354	7.392773
1662.000000	296.188477	7.392392

1663.000000	296.357178	7.391667
1664.000000	296.567749	7.389340
1665.000000	296.703430	7.381291
1666.000000	296.902466	7.387013
1667.000000	297.140839	7.390217
1668.000000	297.245605	7.389149
1669.000000	297.404846	7.385563
1670.000000	297.582397	7.383274
1671.000000	297.709717	7.379269
1672.000000	297.948578	7.380757
1673.000000	298.152344	7.379307
1674.000000	298.229889	7.377781
1675.000000	298.385437	7.379688
1676.000000	298.539429	7.376179
1677.000000	298.728058	7.374424
1678.000000	298.792480	7.375607
1679.000000	298.985779	7.374653
1680.000000	299.169098	7.369541
1681.000000	299.275970	7.369884
1682.000000	299.445679	7.369312
1683.000000	299.557281	7.370914
1684.000000	299.743744	7.371334
1685.000000	299.948059	7.362637
1686.000000	300.119965	7.367634
1687.000000	300.313324	7.366222
1688.000000	300.426544	7.363628

1689.000000	300.590576	7.362141
1690.000000	300.767731	7.359509
1691.000000	300.926544	7.357792
1692.000000	301.094757	7.361416
1693.000000	301.136749	7.359051
1694.000000	301.371002	7.355770
1695.000000	301.609467	7.356419
1696.000000	301.755157	7.354015
1697.000000	301.941742	7.351574
1698.000000	302.136688	7.354511
1699.000000	302.269806	7.351116
1700.000000	302.519287	7.346844
1701.000000	302.697998	7.353901
1702.000000	302.819092	7.347683
1703.000000	303.002533	7.343182
1704.000000	303.056519	7.345356
1705.000000	303.265137	7.345814
1706.000000	303.424438	7.342304
1707.000000	303.602661	7.338909
1708.000000	303.814331	7.341541
1709.000000	303.968445	7.339977
1710.000000	304.159210	7.340282
1711.000000	304.361572	7.335323
1712.000000	304.544983	7.331738
1713.000000	304.714264	7.335018
1714.000000	304.874634	7.335781

1715.000000	305.080658	7.334408
1716.000000	305.165527	7.330135
1717.000000	305.412933	7.328991
1718.000000	305.594757	7.330784
1719.000000	305.787659	7.325024
1720.000000	305.987335	7.326664
1721.000000	306.176514	7.325481
1722.000000	306.309174	7.325405
1723.000000	306.539764	7.321590
1724.000000	306.712158	7.321934
1725.000000	306.891968	7.320866
1726.000000	307.097382	7.318615
1727.000000	307.351074	7.316708
1728.000000	307.489410	7.316860
1729.000000	307.592163	7.317585
1730.000000	307.758820	7.315907
1731.000000	307.887207	7.311100
1732.000000	308.075928	7.311596
1733.000000	308.227386	7.311672
1734.000000	308.424988	7.309536
1735.000000	308.607330	7.309917
1736.000000	308.749390	7.307018
1737.000000	308.888824	7.302822
1738.000000	309.127808	7.302135
1739.000000	309.187012	7.301792
1740.000000	309.428101	7.298550

1741.000000	309.587433	7.299809
1742.000000	309.771942	7.300303
1743.000000	309.958374	7.296871
1744.000000	310.108063	7.296947
1745.000000	310.191101	7.295306
1746.000000	310.287994	7.293857
1747.000000	310.471924	7.290614
1748.000000	310.650665	7.289317
1749.000000	310.759491	7.286571
1750.000000	310.920105	7.285617
1751.000000	311.052246	7.287944
1752.000000	311.232056	7.285693
1753.000000	311.299927	7.283710
1754.000000	311.487488	7.285083
1755.000000	311.648071	7.282260
1756.000000	311.797333	7.282832
1757.000000	312.027374	7.279323
1758.000000	312.166748	7.278712
1759.000000	312.324799	7.276958
1760.000000	312.445557	7.275813
1761.000000	312.644989	7.274440
1762.000000	312.792694	7.273410
1763.000000	312.896271	7.271922
1764.000000	313.026367	7.266238
1765.000000	313.216522	7.267268
1766.000000	313.374023	7.262805

1767.000000	313.536713	7.262729
1768.000000	313.688538	7.260783
1769.000000	313.892120	7.260364
1770.000000	314.129974	7.263759
1771.000000	314.291107	7.254947
1772.000000	314.425812	7.260859
1773.000000	314.555359	7.256892
1774.000000	314.750153	7.254756
1775.000000	315.018066	7.255061
1776.000000	315.145020	7.250522
1777.000000	315.350708	7.249682
1778.000000	315.553772	7.250026
1779.000000	315.694733	7.251933
1780.000000	315.829437	7.246821
1781.000000	315.988525	7.243884
1782.000000	316.242920	7.240489
1783.000000	316.297852	7.245067
1784.000000	316.504059	7.241710
1785.000000	316.783844	7.239001
1786.000000	317.003021	7.239955
1787.000000	317.130493	7.237781
1788.000000	317.231537	7.234004
1789.000000	317.424286	7.230762
1790.000000	317.609253	7.232173
1791.000000	317.784363	7.232135
1792.000000	317.964172	7.230189

1793.000000	318.162109	7.227328
1794.000000	318.333038	7.227366
1795.000000	318.588531	7.224658
1796.000000	318.634613	7.224391
1797.000000	318.881256	7.221568
1798.000000	319.019104	7.220691
1799.000000	319.140350	7.218097
1800.000000	319.309235	7.214282
1801.000000	319.519592	7.216456
1802.000000	319.743408	7.218020
1803.000000	319.885895	7.212413
1804.000000	320.047089	7.212832
1805.000000	320.231720	7.212221
1806.000000	320.318878	7.211497
1807.000000	320.619385	7.206612
1808.000000	320.816284	7.206383
1809.000000	320.939880	7.206193
1810.000000	321.044556	7.204171
1811.000000	321.245575	7.199631
1812.000000	321.441437	7.201233
1813.000000	321.501434	7.198105
1814.000000	321.661957	7.195359
1815.000000	321.877319	7.194329
1816.000000	322.038391	7.191544
1817.000000	322.140472	7.191124
1818.000000	322.359375	7.190476

1819.000000	322.449127	7.187272
1820.000000	322.674255	7.188149
1821.000000	322.833221	7.184868
1822.000000	322.989685	7.184563
1823.000000	323.082001	7.183381
1824.000000	323.264038	7.180939
1825.000000	323.438385	7.179375
1826.000000	323.554810	7.179413
1827.000000	323.708618	7.178994
1828.000000	323.871155	7.177468
1829.000000	324.031738	7.175599
1830.000000	324.182007	7.168541
1831.000000	324.415802	7.169877
1832.000000	324.467590	7.168541
1833.000000	324.676819	7.168274
1834.000000	324.820923	7.166977
1835.000000	324.961456	7.162781
1836.000000	325.193268	7.163239
1837.000000	325.325043	7.163697
1838.000000	325.467590	7.161141
1839.000000	325.653229	7.154809
1840.000000	325.859375	7.156563
1841.000000	325.969116	7.154732
1842.000000	326.143524	7.154465
1843.000000	326.266083	7.149392
1844.000000	326.485016	7.150727

1845.000000	326.634766	7.148133
1846.000000	326.813721	7.146035
1847.000000	326.991730	7.144738
1848.000000	327.153809	7.142945
1849.000000	327.273254	7.143631
1850.000000	327.517853	7.141686
1851.000000	327.755768	7.139359
1852.000000	327.889130	7.135125
1853.000000	328.117401	7.137528
1854.000000	328.250183	7.134018
1855.000000	328.454773	7.131539
1856.000000	328.595306	7.129326
1857.000000	328.738342	7.124482
1858.000000	328.903992	7.127038
1859.000000	329.058411	7.126694
1860.000000	329.253265	7.127076
1861.000000	329.440918	7.120858
1862.000000	329.566071	7.128449
1863.000000	329.660919	7.118149
1864.000000	329.886566	7.118989
1865.000000	330.112274	7.114449
1866.000000	330.256348	7.117577
1867.000000	330.436829	7.113877
1868.000000	330.597870	7.113724
1869.000000	330.769623	7.111092
1870.000000	331.008148	7.110672

1871.000000	331.138428	7.110596
1872.000000	331.260437	7.106324
1873.000000	331.472229	7.104187
1874.000000	331.601440	7.100640
1875.000000	331.802948	7.100029
1876.000000	331.983032	7.101670
1877.000000	332.239410	7.100449
1878.000000	332.379395	7.094078
1879.000000	332.572235	7.090034
1880.000000	332.781952	7.094689
1881.000000	332.946045	7.093697
1882.000000	333.106110	7.087593
1883.000000	333.281982	7.087479
1884.000000	333.449677	7.083092
1885.000000	333.566589	7.081184
1886.000000	333.776825	7.083244
1887.000000	333.910156	7.080421
1888.000000	334.044525	7.079468
1889.000000	334.193787	7.077103
1890.000000	334.314301	7.076569
1891.000000	334.489136	7.077370
1892.000000	334.616333	7.073555
1893.000000	334.747559	7.071381
1894.000000	334.879883	7.072830
1895.000000	335.072266	7.067947
1896.000000	335.205078	7.070351

1897.000000	335.379395	7.065048
1898.000000	335.537354	7.065659
1899.000000	335.723480	7.061195
1900.000000	335.954254	7.059631
1901.000000	336.054291	7.058372
1902.000000	336.234283	7.057266
1903.000000	336.373260	7.056885
1904.000000	336.619904	7.055168
1905.000000	336.737823	7.052536
1906.000000	336.918335	7.050362
1907.000000	337.071747	7.049980
1908.000000	337.141998	7.049484
1909.000000	337.375824	7.043648
1910.000000	337.494293	7.043495
1911.000000	337.573761	7.045937
1912.000000	337.735260	7.040825
1913.000000	337.917358	7.040634
1914.000000	338.149689	7.040482
1915.000000	338.331177	7.036095
1916.000000	338.525574	7.033310
1917.000000	338.679901	7.030716
1918.000000	338.908081	7.030487
1919.000000	339.030182	7.028656
1920.000000	339.212769	7.025833
1921.000000	339.385559	7.026367
1922.000000	339.564026	7.026138

1923.000000	339.766022	7.026596
1924.000000	339.942352	7.027130
1925.000000	340.054138	7.023354
1926.000000	340.259552	7.017174
1927.000000	340.352844	7.015152
1928.000000	340.592163	7.015190
1929.000000	340.709839	7.011032
1930.000000	340.878723	7.010612
1931.000000	341.107422	7.009964
1932.000000	341.251434	7.012024
1933.000000	341.438019	7.006874
1934.000000	341.640381	7.003632
1935.000000	341.749908	7.002907
1936.000000	341.913696	7.002029
1937.000000	342.063293	7.001839
1938.000000	342.243835	6.996002
1939.000000	342.387817	6.993446
1940.000000	342.499908	6.995392
1941.000000	342.791473	6.995773
1942.000000	342.890869	6.988449
1943.000000	343.088623	6.989250
1944.000000	343.184998	6.988182
1945.000000	343.387329	6.985970
1946.000000	343.590698	6.987038
1947.000000	343.781860	6.984634
1948.000000	343.897949	6.981391

1949.000000	344.110962	6.979751
1950.000000	344.243286	6.976240
1951.000000	344.390320	6.977653
1952.000000	344.578003	6.977346
1953.000000	344.753448	6.970898
1954.000000	344.880249	6.970669
1955.000000	345.005005	6.971203
1956.000000	345.219971	6.969792
1957.000000	345.372131	6.962735
1958.000000	345.519165	6.964222
1959.000000	345.747864	6.964146
1960.000000	345.954254	6.963078
1961.000000	346.018188	6.955754
1962.000000	346.221008	6.961094
1963.000000	346.364014	6.955220
1964.000000	346.616516	6.947934
1965.000000	346.774261	6.952130
1966.000000	346.936523	6.950451
1967.000000	347.114990	6.947628
1968.000000	347.265137	6.951329
1969.000000	347.458313	6.945111
1970.000000	347.607422	6.942669
1971.000000	347.782349	6.939732
1972.000000	347.964905	6.940114
1973.000000	348.150513	6.939007
1974.000000	348.319855	6.936146

1975.000000	348.482178	6.934315
1976.000000	348.693604	6.935841
1977.000000	348.818359	6.929356
1978.000000	349.047607	6.929585
1979.000000	349.218445	6.927067
1980.000000	349.358948	6.927220
1981.000000	349.473053	6.926342
1982.000000	349.651550	6.921307
1983.000000	349.780853	6.920849
1984.000000	350.033905	6.914784
1985.000000	350.305328	6.913678
1986.000000	350.447388	6.917607
1987.000000	350.590942	6.917492
1988.000000	350.726929	6.912266
1989.000000	350.881195	6.910130
1990.000000	351.081085	6.908108
1991.000000	351.256592	6.905743
1992.000000	351.460571	6.904370
1993.000000	351.549377	6.900479
1994.000000	351.761414	6.903073
1995.000000	351.900421	6.896664
1996.000000	352.047607	6.897465
1997.000000	352.210938	6.898152
1998.000000	352.385986	6.896168
1999.000000	352.459076	6.893841
2000.000000	352.716797	6.889798

2001.000000	352.862396	6.886517
2002.000000	352.990295	6.890141
2003.000000	353.038452	6.883770
2004.000000	353.229736	6.883732
2005.000000	353.395630	6.884762
2006.000000	353.590942	6.881367
2007.000000	353.801514	6.881749
2008.000000	353.912598	6.875607
2009.000000	354.040466	6.876103
2010.000000	354.153625	6.876904
2011.000000	354.382935	6.872632
2012.000000	354.588959	6.867444
2013.000000	354.686859	6.869160
2014.000000	354.817749	6.868397
2015.000000	355.027283	6.861950
2016.000000	355.204346	6.868245
2017.000000	355.340881	6.864163
2018.000000	355.586395	6.858593
2019.000000	355.687378	6.860386
2020.000000	355.883698	6.856915
2021.000000	356.086182	6.858364
2022.000000	356.302795	6.853062
2023.000000	356.422516	6.851002
2024.000000	356.507233	6.852642
2025.000000	356.667084	6.847149
2026.000000	356.787323	6.844555

2027.000000	357.018188	6.844326
2028.000000	357.300781	6.844135
2029.000000	357.403290	6.839481
2030.000000	357.556976	6.831623
2031.000000	357.838043	6.834751
2032.000000	357.918213	6.832882
2033.000000	358.146515	6.832767
2034.000000	358.253082	6.830555
2035.000000	358.463623	6.827923
2036.000000	358.621948	6.827389
2037.000000	358.752869	6.827808
2038.000000	358.958313	6.825253
2039.000000	359.038971	6.822964
2040.000000	359.241394	6.821590
2041.000000	359.552948	6.816975
2042.000000	359.714264	6.816631
2043.000000	359.858856	6.815334
2044.000000	360.068909	6.813694
2045.000000	360.206909	6.810986
2046.000000	360.394653	6.802212
2047.000000	360.574738	6.806980
2048.000000	360.713226	6.805988
2049.000000	360.902008	6.805416
2050.000000	361.093750	6.804882
2051.000000	361.178986	6.802250
2052.000000	361.351990	6.799503

2053.000000	361.500641	6.793438
2054.000000	361.663513	6.796986
2055.000000	361.845673	6.791874
2056.000000	362.072449	6.788975
2057.000000	362.122681	6.785313
2058.000000	362.335785	6.784016
2059.000000	362.461121	6.781841
2060.000000	362.634094	6.779705
2061.000000	362.738098	6.780849
2062.000000	362.909088	6.776157
2063.000000	363.051636	6.775967
2064.000000	363.278442	6.778980
2065.000000	363.476807	6.773907
2066.000000	363.615814	6.772381
2067.000000	363.793915	6.767727
2068.000000	364.002411	6.766621
2069.000000	364.122192	6.764370
2070.000000	364.321045	6.762615
2071.000000	364.488983	6.765476
2072.000000	364.684814	6.760479
2073.000000	364.932922	6.760403
2074.000000	365.016113	6.761204
2075.000000	365.213013	6.755291
2076.000000	365.354523	6.753078
2077.000000	365.419495	6.752811
2078.000000	365.608704	6.752964

2079.000000	365.759430	6.749415
2080.000000	365.953247	6.747661
2081.000000	366.088165	6.741670
2082.000000	366.280457	6.744647
2083.000000	366.461090	6.738350
2084.000000	366.611267	6.738961
2085.000000	366.753845	6.740334
2086.000000	366.840576	6.730568
2087.000000	367.037964	6.733887
2088.000000	367.105408	6.736672
2089.000000	367.393585	6.729424
2090.000000	367.554443	6.727440
2091.000000	367.725952	6.727135
2092.000000	367.910583	6.724045
2093.000000	368.032349	6.723511
2094.000000	368.290649	6.722405
2095.000000	368.336304	6.719658
2096.000000	368.545319	6.716683
2097.000000	368.729492	6.715539
2098.000000	368.910553	6.716721
2099.000000	368.991272	6.713593
2100.000000	369.195221	6.708748
2101.000000	369.319519	6.707757
2102.000000	369.503723	6.701958
2103.000000	369.715302	6.701653
2104.000000	369.955872	6.700318

2105.000000	370.099670	6.697304
2106.000000	370.330566	6.696808
2107.000000	370.388763	6.696618
2108.000000	370.638611	6.690781
2109.000000	370.826294	6.691697
2110.000000	370.993866	6.689942
2111.000000	371.125854	6.689484
2112.000000	371.170502	6.684639
2113.000000	371.539795	6.679871
2114.000000	371.712891	6.681054
2115.000000	371.817261	6.680596
2116.000000	371.987366	6.675408
2117.000000	372.137360	6.677163
2118.000000	372.215637	6.681321
2119.000000	372.474579	6.671975
2120.000000	372.663208	6.670029
2121.000000	372.792206	6.669877
2122.000000	372.948761	6.668084
2123.000000	373.145935	6.666520
2124.000000	373.306000	6.661255
2125.000000	373.504669	6.659157
2126.000000	373.678802	6.656983
2127.000000	373.813751	6.654809
2128.000000	373.955750	6.653855
2129.000000	374.187561	6.652863
2130.000000	374.372681	6.654885

2131.000000	374.493164	6.650994
2132.000000	374.710876	6.646950
2133.000000	374.853882	6.647904
2134.000000	374.955261	6.645081
2135.000000	375.241730	6.641037
2136.000000	375.408325	6.641610
2137.000000	375.558350	6.636002
2138.000000	375.729462	6.639626
2139.000000	375.864441	6.639664
2140.000000	375.970337	6.635010
2141.000000	376.245789	6.632721
2142.000000	376.309448	6.628105
2143.000000	376.553345	6.630013
2144.000000	376.689331	6.625969
2145.000000	376.924164	6.622078
2146.000000	377.048065	6.620552
2147.000000	377.171509	6.619637
2148.000000	377.392303	6.615326
2149.000000	377.515717	6.613953
2150.000000	377.633636	6.612312
2151.000000	377.677246	6.613075
2152.000000	377.894043	6.608040
2153.000000	378.027496	6.608154
2154.000000	378.123840	6.605446
2155.000000	378.371704	6.599686
2156.000000	378.436432	6.603233

2157.000000	378.658203	6.597206
2158.000000	378.868469	6.596252
2159.000000	379.007446	6.593849
2160.000000	379.159454	6.595566
2161.000000	379.355133	6.590454
2162.000000	379.528259	6.590263
2163.000000	379.652191	6.585686
2164.000000	379.819794	6.585114
2165.000000	380.024017	6.585114
2166.000000	380.130432	6.580956
2167.000000	380.329712	6.579277
2168.000000	380.446686	6.579582
2169.000000	380.676605	6.577713
2170.000000	380.832764	6.570160
2171.000000	381.012451	6.573593
2172.000000	381.259918	6.572487
2173.000000	381.418579	6.568634
2174.000000	381.535034	6.565582
2175.000000	381.756470	6.561462
2176.000000	381.911560	6.559975
2177.000000	382.132446	6.561081
2178.000000	382.346802	6.558182
2179.000000	382.432129	6.555664
2180.000000	382.607330	6.549980
2181.000000	382.766968	6.553719
2182.000000	382.985840	6.551773

2183.000000	383.171600	6.549828
2184.000000	383.300079	6.543800
2185.000000	383.531494	6.543953
2186.000000	383.695679	6.542007
2187.000000	383.848785	6.537354
2188.000000	384.047607	6.538116
2189.000000	384.215271	6.535332
2190.000000	384.334290	6.535828
2191.000000	384.627930	6.530106
2192.000000	384.772491	6.529877
2193.000000	384.940186	6.528580
2194.000000	385.144989	6.524002
2195.000000	385.356323	6.525261
2196.000000	385.419067	6.522667
2197.000000	385.602325	6.518433
2198.000000	385.770996	6.519348
2199.000000	385.902008	6.516868
2200.000000	386.100830	6.511831
2201.000000	386.275513	6.511945
2202.000000	386.464233	6.509923
2203.000000	386.551086	6.510992
2204.000000	386.693146	6.505879
2205.000000	386.862885	6.505879
2206.000000	387.042114	6.502446
2207.000000	387.167114	6.502331
2208.000000	387.337250	6.498517

2209.000000	387.470825	6.494282
2210.000000	387.717773	6.494702
2211.000000	387.735352	6.491230
2212.000000	387.979858	6.489132
2213.000000	388.036041	6.489247
2214.000000	388.268951	6.486195
2215.000000	388.411591	6.482342
2216.000000	388.593750	6.483487
2217.000000	388.712769	6.479672
2218.000000	388.909515	6.474408
2219.000000	389.082733	6.473073
2220.000000	389.157532	6.475895
2221.000000	389.367371	6.472920
2222.000000	389.507935	6.471509
2223.000000	389.674133	6.465634
2224.000000	389.840759	6.460980
2225.000000	389.929626	6.463192
2226.000000	390.162720	6.465062
2227.000000	390.264679	6.455945
2228.000000	390.449493	6.455601
2229.000000	390.615906	6.453808
2230.000000	390.793213	6.450985
2231.000000	390.901062	6.449574
2232.000000	391.093811	6.450451
2233.000000	391.284058	6.446484
2234.000000	391.439026	6.448010

2235.000000	391.684937	6.443356
2236.000000	391.839447	6.441220
2237.000000	391.997955	6.438435
2238.000000	392.159851	6.434353
2239.000000	392.334717	6.436108
2240.000000	392.508087	6.438168
2241.000000	392.679016	6.433209
2242.000000	392.877197	6.429738
2243.000000	393.016296	6.429966
2244.000000	393.142456	6.427563
2245.000000	393.350616	6.426190
2246.000000	393.507599	6.420163
2247.000000	393.716217	6.420010
2248.000000	393.848389	6.419056
2249.000000	394.081879	6.414746
2250.000000	394.290039	6.415204
2251.000000	394.395325	6.412724
2252.000000	394.610962	6.412152
2253.000000	394.812622	6.407994
2254.000000	395.052551	6.405171
2255.000000	395.188171	6.401738
2256.000000	395.365082	6.401127
2257.000000	395.605957	6.400860
2258.000000	395.687927	6.399182
2259.000000	395.878693	6.392353
2260.000000	396.054535	6.393803

2261.000000	396.201630	6.393192
2262.000000	396.340179	6.389340
2263.000000	396.476807	6.389645
2264.000000	396.678467	6.385525
2265.000000	396.864288	6.383312
2266.000000	396.985016	6.381824
2267.000000	397.140472	6.384571
2268.000000	397.429596	6.379879
2269.000000	397.546387	6.373661
2270.000000	397.664093	6.376141
2271.000000	397.863281	6.372517
2272.000000	397.949219	6.370762
2273.000000	398.096802	6.366909
2274.000000	398.311890	6.361645
2275.000000	398.500153	6.365231
2276.000000	398.699341	6.361607
2277.000000	398.779358	6.358822
2278.000000	398.987976	6.360043
2279.000000	399.142456	6.361797
2280.000000	399.305908	6.350010
2281.000000	399.460388	6.353596
2282.000000	399.591095	6.347263
2283.000000	399.794220	6.348522
2284.000000	399.944275	6.347683
2285.000000	400.035645	6.341312
2286.000000	400.240814	6.343258

2287.000000	400.371490	6.341389
2288.000000	400.562744	6.334599
2289.000000	400.649689	6.338833
2290.000000	400.775879	6.333721
2291.000000	400.995422	6.333530
2292.000000	401.267151	6.329983
2293.000000	401.245300	6.324413
2294.000000	401.462921	6.324947
2295.000000	401.688416	6.328381
2296.000000	401.709290	6.325062
2297.000000	401.950745	6.321781
2298.000000	402.146454	6.319454
2299.000000	402.315369	6.320332
2300.000000	402.499634	6.316555
2301.000000	402.721710	6.314114
2302.000000	402.922913	6.315029
2303.000000	403.004883	6.311176
2304.000000	403.232422	6.310184
2305.000000	403.421143	6.309727
2306.000000	403.545868	6.305187
2307.000000	403.714294	6.304729
2308.000000	403.900543	6.301563
2309.000000	404.096283	6.300838
2310.000000	404.227448	6.300037
2311.000000	404.371979	6.297405
2312.000000	404.583130	6.294086

2313.000000	404.732666	6.295460
2314.000000	404.959686	6.290462
2315.000000	405.092804	6.291721
2316.000000	405.253265	6.291073
2317.000000	405.478302	6.287182
2318.000000	405.645203	6.288364
2319.000000	405.801697	6.285236
2320.000000	405.961182	6.279055
2321.000000	406.087830	6.277605
2322.000000	406.354614	6.276155
2323.000000	406.520508	6.276231
2324.000000	406.553802	6.280314
2325.000000	406.859833	6.269670
2326.000000	407.052551	6.270814
2327.000000	407.128571	6.266046
2328.000000	407.389374	6.266580
2329.000000	407.573669	6.262765
2330.000000	407.706299	6.265283
2331.000000	407.868774	6.265626
2332.000000	408.036163	6.262498
2333.000000	408.192627	6.257501
2334.000000	408.320313	6.261468
2335.000000	408.557800	6.255861
2336.000000	408.760986	6.253763
2337.000000	408.886139	6.252923
2338.000000	408.997894	6.254297

2339.000000	409.310394	6.252580
2340.000000	409.358093	6.249986
2341.000000	409.601929	6.249032
2342.000000	409.730103	6.245141
2343.000000	409.868744	6.247583
2344.000000	410.060425	6.245141
2345.000000	410.176147	6.242700
2346.000000	410.272461	6.239877
2347.000000	410.419952	6.235833
2348.000000	410.693054	6.234803
2349.000000	410.779907	6.235871
2350.000000	410.975037	6.236672
2351.000000	411.093750	6.230035
2352.000000	411.287872	6.233315
2353.000000	411.477539	6.229539
2354.000000	411.622070	6.228890
2355.000000	411.821625	6.224732
2356.000000	411.898560	6.225953
2357.000000	412.052002	6.224313
2358.000000	412.288849	6.222444
2359.000000	412.435333	6.222634
2360.000000	412.566895	6.220765
2361.000000	412.641937	6.219354
2362.000000	412.864838	6.215158
2363.000000	413.072876	6.215348
2364.000000	413.212891	6.215119

2365.000000	413.382202	6.210885
2366.000000	413.539642	6.212068
2367.000000	413.697998	6.206613
2368.000000	413.853882	6.206880
2369.000000	414.021240	6.204247
2370.000000	414.099152	6.200433
2371.000000	414.291351	6.199441
2372.000000	414.458191	6.195588
2373.000000	414.680115	6.202111
2374.000000	414.807709	6.197724
2375.000000	414.979523	6.196275
2376.000000	415.194031	6.194711
2377.000000	415.352417	6.191468
2378.000000	415.556000	6.195283
2379.000000	415.769043	6.190705
2380.000000	415.927917	6.187043
2381.000000	416.149841	6.187005
2382.000000	416.320648	6.185250
2383.000000	416.468597	6.185937
2384.000000	416.625488	6.185517
2385.000000	416.817657	6.179833
2386.000000	416.953217	6.179109
2387.000000	417.201996	6.175141
2388.000000	417.264526	6.181512
2389.000000	417.485962	6.179452
2390.000000	417.662231	6.174417

2391.000000	417.805267	6.171327
2392.000000	417.995392	6.171556
2393.000000	418.205444	6.167893
2394.000000	418.328064	6.171517
2395.000000	418.511322	6.169419
2396.000000	418.621521	6.169381
2397.000000	418.826111	6.166482
2398.000000	419.047607	6.160721
2399.000000	419.179626	6.159996
2400.000000	419.325134	6.161827
2401.000000	419.513794	6.158318
2402.000000	419.735718	6.158661
2403.000000	419.905029	6.155533
2404.000000	420.058289	6.155571
2405.000000	420.229492	6.153015
2406.000000	420.327332	6.155075
2407.000000	420.430542	6.150536
2408.000000	420.625732	6.154961
2409.000000	420.711761	6.148209
2410.000000	421.005249	6.148743
2411.000000	421.199493	6.147255
2412.000000	421.250122	6.145462
2413.000000	421.371582	6.137527
2414.000000	421.590851	6.139816
2415.000000	421.696564	6.141037
2416.000000	421.878448	6.135963

2417.000000	422.057892	6.139053
2418.000000	422.264404	6.138176
2419.000000	422.481201	6.135735
2420.000000	422.665070	6.131233
2421.000000	422.729980	6.132111
2422.000000	422.797302	6.132072
2423.000000	423.005737	6.128067
2424.000000	423.257507	6.129288
2425.000000	423.364136	6.127914
2426.000000	423.485596	6.127609
2427.000000	423.637573	6.123260
2428.000000	423.749146	6.122765
2429.000000	423.884857	6.122269
2430.000000	424.042175	6.121849
2431.000000	424.227051	6.119026
2432.000000	424.466003	6.117386
2433.000000	424.689209	6.118721
2434.000000	424.885284	6.113419
2435.000000	425.024017	6.112541
2436.000000	425.185242	6.111816
2437.000000	425.337158	6.108803
2438.000000	425.537781	6.103348
2439.000000	425.700958	6.106781
2440.000000	425.986115	6.105446
2441.000000	426.036255	6.102547
2442.000000	426.245697	6.106323

2443.000000	426.380951	6.109375
2444.000000	426.497406	6.100983
2445.000000	426.643005	6.099380
2446.000000	426.803711	6.097359
2447.000000	427.011230	6.096901
2448.000000	427.201935	6.097702
2449.000000	427.400024	6.093582
2450.000000	427.543152	6.096062
2451.000000	427.710297	6.096100
2452.000000	427.958618	6.091751
2453.000000	428.185730	6.088051
2454.000000	428.321899	6.087746
2455.000000	428.479187	6.088356
2456.000000	428.702484	6.085571
2457.000000	428.892181	6.086334
2458.000000	429.004822	6.080193
2459.000000	429.248657	6.080383
2460.000000	429.299744	6.079773
2461.000000	429.495453	6.081718
2462.000000	429.693115	6.075424
2463.000000	429.826843	6.076492
2464.000000	430.006134	6.075119
2465.000000	430.156860	6.070313
2466.000000	430.324310	6.075806
2467.000000	430.448486	6.068787
2468.000000	430.648193	6.069092

2469.000000	430.771332	6.068024
2470.000000	430.985443	6.067871
2471.000000	431.176758	6.061501
2472.000000	431.372986	6.064209
2473.000000	431.465851	6.062645
2474.000000	431.616333	6.062378
2475.000000	431.720215	6.057495
2476.000000	431.911469	6.059402
2477.000000	432.023224	6.058029
2478.000000	432.243835	6.056351
2479.000000	432.375977	6.054329
2480.000000	432.593018	6.054405
2481.000000	432.712250	6.051887
2482.000000	432.941742	6.046431
2483.000000	433.173279	6.048224
2484.000000	433.380890	6.044333
2485.000000	433.543823	6.044561
2486.000000	433.657104	6.043188
2487.000000	433.798187	6.044790
2488.000000	433.987000	6.038953
2489.000000	434.135010	6.038267
2490.000000	434.293976	6.038076
2491.000000	434.456909	6.037847
2492.000000	434.636230	6.035673
2493.000000	434.810577	6.036207
2494.000000	434.916992	6.035863

2495.000000	435.171753	6.030637
2496.000000	435.323761	6.030942
2497.000000	435.512054	6.028959
2498.000000	435.643188	6.030561
2499.000000	435.771362	6.026594
2500.000000	435.910919	6.023580
2501.000000	436.063995	6.023542
2502.000000	436.210052	6.026594
2503.000000	436.373413	6.022169
2504.000000	436.591553	6.021635
2505.000000	436.727661	6.021482
2506.000000	436.922882	6.018506
2507.000000	437.034119	6.014539
2508.000000	437.194122	6.014883
2509.000000	437.419189	6.015073
2510.000000	437.547363	6.012174
2511.000000	437.714233	6.008664
2512.000000	437.878662	6.008702
2513.000000	438.054535	6.008588
2514.000000	438.221466	6.011868
2515.000000	438.372925	6.006108
2516.000000	438.461853	6.003476
2517.000000	438.624298	6.001874
2518.000000	438.801147	6.002866
2519.000000	438.923859	5.998364
2520.000000	439.125580	5.999852

2521.000000	439.291016	5.993749
2522.000000	439.469818	5.995236
2523.000000	439.704834	5.992757
2524.000000	439.900085	5.992452
2525.000000	440.022583	5.993291
2526.000000	440.263611	5.990239
2527.000000	440.434937	5.986196
2528.000000	440.649506	5.988141
2529.000000	440.839294	5.987950
2530.000000	441.047546	5.983640
2531.000000	441.161469	5.980817
2532.000000	441.322021	5.976430
2533.000000	441.500580	5.976888
2534.000000	441.635437	5.980092
2535.000000	441.894714	5.976087
2536.000000	442.081146	5.971509
2537.000000	442.131775	5.976010
2538.000000	442.346863	5.970746
2539.000000	442.620850	5.967198
2540.000000	442.702087	5.965100
2541.000000	442.845154	5.964795
2542.000000	443.031525	5.964681
2543.000000	443.156097	5.962506
2544.000000	443.269470	5.964566
2545.000000	443.489929	5.958920
2546.000000	443.590668	5.953275

2547.000000	443.767761	5.953542
2548.000000	443.938568	5.952092
2549.000000	444.150726	5.956059
2550.000000	444.360474	5.952016
2551.000000	444.407227	5.949994
2552.000000	444.598480	5.944615
2553.000000	444.804291	5.946523
2554.000000	444.932739	5.945607
2555.000000	445.087494	5.944158
2556.000000	445.281097	5.938016
2557.000000	445.403809	5.934812
2558.000000	445.567261	5.934468
2559.000000	445.735138	5.935346
2560.000000	445.895782	5.930080
2561.000000	446.086517	5.929356
2562.000000	446.278259	5.929738
2563.000000	446.442230	5.927677
2564.000000	446.569275	5.927830
2565.000000	446.721039	5.922260
2566.000000	446.943451	5.921268
2567.000000	446.969727	5.919209
2568.000000	447.152679	5.916042
2569.000000	447.344421	5.912838
2570.000000	447.485535	5.915585
2571.000000	447.644653	5.913639
2572.000000	447.767761	5.909710

2573.000000	447.984802	5.911770
2574.000000	448.140991	5.913029
2575.000000	448.272400	5.904980
2576.000000	448.463623	5.901470
2577.000000	448.608643	5.902729
2578.000000	448.801361	5.898380
2579.000000	449.008636	5.897427
2580.000000	449.099640	5.891247
2581.000000	449.303986	5.895939
2582.000000	449.501587	5.892086
2583.000000	449.701111	5.888615
2584.000000	449.930786	5.891628
2585.000000	450.057953	5.886288
2586.000000	450.306000	5.884800
2587.000000	450.460815	5.882053
2588.000000	450.625427	5.882969
2589.000000	450.753662	5.877857
2590.000000	450.938965	5.878582
2591.000000	451.119324	5.873508
2592.000000	451.286377	5.873203
2593.000000	451.419525	5.872593
2594.000000	451.561554	5.867634
2595.000000	451.762054	5.866909
2596.000000	451.893250	5.867023
2597.000000	452.071136	5.863590
2598.000000	452.224945	5.859280

2599.000000	452.401367	5.860882
2600.000000	452.543884	5.855541
2601.000000	452.764496	5.851727
2602.000000	452.894714	5.851994
2603.000000	453.071625	5.850811
2604.000000	453.230896	5.847645
2605.000000	453.327637	5.846767
2606.000000	453.530121	5.842342
2607.000000	453.711945	5.838566
2608.000000	453.806763	5.839710
2609.000000	454.094727	5.835018
2610.000000	454.262299	5.836697
2611.000000	454.421997	5.830746
2612.000000	454.581177	5.829563
2613.000000	454.723236	5.827923
2614.000000	454.918304	5.830097
2615.000000	455.159058	5.823612
2616.000000	455.384155	5.818539
2617.000000	455.555664	5.821285
2618.000000	455.649017	5.815333
2619.000000	455.806274	5.817775
2620.000000	455.983185	5.807359
2621.000000	456.188568	5.809572
2622.000000	456.245544	5.802858
2623.000000	456.415131	5.804307
2624.000000	456.659790	5.801370

2625.000000	456.779236	5.797784
2626.000000	456.923706	5.798318
2627.000000	457.063751	5.796373
2628.000000	457.250000	5.793512
2629.000000	457.414154	5.788400
2630.000000	457.582642	5.787675
2631.000000	457.727142	5.783403
2632.000000	457.937012	5.778901
2633.000000	458.055420	5.781724
2634.000000	458.246094	5.778176
2635.000000	458.445587	5.776917
2636.000000	458.573822	5.771577
2637.000000	458.701111	5.769135
2638.000000	458.918793	5.769822
2639.000000	459.108978	5.765702
2640.000000	459.240662	5.760324
2641.000000	459.380219	5.760285
2642.000000	459.542419	5.757844
2643.000000	459.707947	5.757691
2644.000000	459.851440	5.754563
2645.000000	460.054810	5.752427
2646.000000	460.192139	5.745980
2647.000000	460.328857	5.744912
2648.000000	460.438812	5.738427
2649.000000	460.715179	5.738008
2650.000000	460.923950	5.735108

2651.000000	461.032471	5.732896
2652.000000	461.161896	5.732133
2653.000000	461.290405	5.729424
2654.000000	461.532257	5.725648
2655.000000	461.593536	5.725305
2656.000000	461.790619	5.721604
2657.000000	461.954620	5.718438
2658.000000	462.105957	5.711190
2659.000000	462.239746	5.709664
2660.000000	462.412048	5.707833
2661.000000	462.588715	5.706384
2662.000000	462.791595	5.701806
2663.000000	462.913269	5.699669
2664.000000	463.137573	5.699784
2665.000000	463.398926	5.697495
2666.000000	463.580902	5.694405
2667.000000	463.734222	5.690590
2668.000000	463.879669	5.685211
2669.000000	464.118164	5.684906
2670.000000	464.278687	5.678307
2671.000000	464.340515	5.674759
2672.000000	464.663605	5.674263
2673.000000	464.784790	5.670143
2674.000000	464.980896	5.670105
2675.000000	465.154144	5.667244
2676.000000	465.248047	5.664688

2677.000000	465.421753	5.662743
2678.000000	465.585236	5.654388
2679.000000	465.741486	5.654961
2680.000000	465.923950	5.652023
2681.000000	466.019318	5.647903
2682.000000	466.185730	5.645119
2683.000000	466.390625	5.641113
2684.000000	466.556030	5.641571
2685.000000	466.759033	5.638443
2686.000000	466.858734	5.635353
2687.000000	467.090851	5.632492
2688.000000	467.250488	5.624329
2689.000000	467.459259	5.627113
2690.000000	467.651489	5.620399
2691.000000	467.756104	5.617195
2692.000000	467.958527	5.611435
2693.000000	468.219818	5.612160
2694.000000	468.315704	5.610786
2695.000000	468.475281	5.606705
2696.000000	468.625183	5.602432
2697.000000	468.895752	5.598503
2698.000000	469.035400	5.595490
2699.000000	469.270874	5.588585
2700.000000	469.398865	5.587821
2701.000000	469.569214	5.584692
2702.000000	469.695740	5.583396

2703.000000	469.893311	5.577673
2704.000000	470.040283	5.574354
2705.000000	470.199402	5.570997
2706.000000	470.417419	5.568632
2707.000000	470.606689	5.564359
2708.000000	470.688416	5.561231
2709.000000	470.876312	5.559934
2710.000000	471.014496	5.555509
2711.000000	471.110352	5.557416
2712.000000	471.300629	5.552152
2713.000000	471.419800	5.547078
2714.000000	471.619324	5.544751
2715.000000	471.802826	5.538457
2716.000000	471.887970	5.539487
2717.000000	472.018829	5.534642
2718.000000	472.156097	5.531972
2719.000000	472.272400	5.526250
2720.000000	472.487976	5.526402
2721.000000	472.652435	5.524190
2722.000000	472.766357	5.519383
2723.000000	472.885529	5.518659
2724.000000	473.145386	5.514424
2725.000000	473.256348	5.512059
2726.000000	473.429077	5.507825
2727.000000	473.522461	5.503858
2728.000000	473.751709	5.504354

2729.000000	473.857788	5.498746
2730.000000	474.057251	5.497830
2731.000000	474.226166	5.492032
2732.000000	474.423218	5.492490
2733.000000	474.638794	5.485051
2734.000000	474.810089	5.483373
2735.000000	475.038818	5.480512
2736.000000	475.172150	5.475934
2737.000000	475.363373	5.478070
2738.000000	475.467041	5.471166
2739.000000	475.678741	5.470860
2740.000000	475.848480	5.468075
2741.000000	476.015900	5.464413
2742.000000	476.237335	5.458118
2743.000000	476.348816	5.459072
2744.000000	476.567749	5.455372
2745.000000	476.785736	5.451824
2746.000000	476.964844	5.451443
2747.000000	477.164307	5.447781
2748.000000	477.236908	5.441906
2749.000000	477.406250	5.444691
2750.000000	477.618835	5.439503
2751.000000	477.873383	5.437023
2752.000000	478.058777	5.434887
2753.000000	478.193115	5.429623
2754.000000	478.352631	5.425312

2755.000000	478.480164	5.423710
2756.000000	478.619812	5.422451
2757.000000	478.870392	5.420315
2758.000000	479.090912	5.415852
2759.000000	479.177979	5.415966
2760.000000	479.334229	5.410206
2761.000000	479.490845	5.412609
2762.000000	479.616943	5.407078
2763.000000	479.750244	5.404064
2764.000000	479.922394	5.399982
2765.000000	480.124146	5.397083
2766.000000	480.265747	5.394794
2767.000000	480.430054	5.394604
2768.000000	480.537445	5.392773
2769.000000	480.723877	5.384495
2770.000000	480.908813	5.385792
2771.000000	481.066284	5.382397
2772.000000	481.339966	5.379269
2773.000000	481.484467	5.377323
2774.000000	481.628510	5.374538
2775.000000	481.756622	5.371487
2776.000000	481.901123	5.372860
2777.000000	482.065918	5.369732
2778.000000	482.260437	5.367634
2779.000000	482.371765	5.362904
2780.000000	482.586548	5.361111

2781.000000	482.741211	5.357067
2782.000000	482.952179	5.354740
2783.000000	483.076447	5.357029
2784.000000	483.238312	5.349283
2785.000000	483.363556	5.349399
2786.000000	483.549011	5.349245
2787.000000	483.680023	5.339059
2788.000000	483.834656	5.342569
2789.000000	484.078857	5.337114
2790.000000	484.222351	5.335016
2791.000000	484.308136	5.336465
2792.000000	484.500366	5.339136
2793.000000	484.730591	5.332841
2794.000000	484.817261	5.327882
2795.000000	485.033630	5.331506
2796.000000	485.100098	5.323648
2797.000000	485.342804	5.323305
2798.000000	485.477234	5.318651
2799.000000	485.633301	5.316705
2800.000000	485.826996	5.311174
2801.000000	486.026886	5.314722
2802.000000	486.140503	5.313310
2803.000000	486.323578	5.309686
2804.000000	486.524933	5.305223
2805.000000	486.655945	5.305490
2806.000000	486.815857	5.303316

2807.000000	486.966156	5.297327
2808.000000	487.153564	5.298662
2809.000000	487.286011	5.297365
2810.000000	487.419952	5.295763
2811.000000	487.624634	5.289430
2812.000000	487.839478	5.289239
2813.000000	487.922302	5.286263
2814.000000	488.112091	5.287332
2815.000000	488.296631	5.285310
2816.000000	488.487823	5.285119
2817.000000	488.678070	5.280694
2818.000000	488.929047	5.274781
2819.000000	489.073578	5.278024
2820.000000	489.244080	5.273103
2821.000000	489.443542	5.272759
2822.000000	489.596191	5.269631
2823.000000	489.786957	5.260934
2824.000000	489.956970	5.266351
2825.000000	490.130402	5.263146
2826.000000	490.246948	5.259904
2827.000000	490.430511	5.258340
2828.000000	490.609222	5.255822
2829.000000	490.805267	5.257958
2830.000000	490.995605	5.253381
2831.000000	491.133728	5.250749
2832.000000	491.389099	5.249490

2833.000000	491.526855	5.249986
2834.000000	491.710876	5.244798
2835.000000	491.784546	5.244721
2836.000000	492.022003	5.243463
2837.000000	492.158325	5.239839
2838.000000	492.271515	5.235833
2839.000000	492.535034	5.235833
2840.000000	492.685333	5.230416
2841.000000	492.788452	5.231446
2842.000000	492.987335	5.229080
2843.000000	493.167023	5.230187
2844.000000	493.405945	5.226830
2845.000000	493.467621	5.226257
2846.000000	493.644409	5.223167
2847.000000	493.784546	5.221069
2848.000000	494.026855	5.215843
2849.000000	494.192078	5.215500
2850.000000	494.263916	5.213326
2851.000000	494.472412	5.215767
2852.000000	494.613068	5.215004
2853.000000	494.801453	5.207870
2854.000000	494.977722	5.209091
2855.000000	495.130890	5.206421
2856.000000	495.245056	5.204895
2857.000000	495.448822	5.202873
2858.000000	495.563019	5.205009

2859.000000	495.844330	5.201805
2860.000000	495.966187	5.195053
2861.000000	496.121765	5.193871
2862.000000	496.246521	5.194099
2863.000000	496.424744	5.194138
2864.000000	496.592377	5.189598
2865.000000	496.664154	5.189713
2866.000000	496.854858	5.186394
2867.000000	497.028809	5.186852
2868.000000	497.215210	5.178841
2869.000000	497.346222	5.181015
2870.000000	497.587585	5.180939
2871.000000	497.613556	5.180443
2872.000000	497.833679	5.175102
2873.000000	497.951660	5.176819
2874.000000	498.196381	5.173920
2875.000000	498.275391	5.175255
2876.000000	498.585144	5.172089
2877.000000	498.677124	5.170143
2878.000000	498.882324	5.166519
2879.000000	498.984467	5.165222
2880.000000	499.217621	5.161140
2881.000000	499.413177	5.162704
2882.000000	499.567749	5.161217
2883.000000	499.660736	5.159805
2884.000000	499.776794	5.155457

2885.000000	500.034546	5.151871
2886.000000	500.109650	5.152023
2887.000000	500.358521	5.152138
2888.000000	500.571808	5.153473
2889.000000	500.705688	5.146797
2890.000000	500.967163	5.143822
2891.000000	501.133179	5.144814
2892.000000	501.265167	5.144356
2893.000000	501.325806	5.142525
2894.000000	501.584351	5.142296
2895.000000	501.714386	5.139664
2896.000000	501.794800	5.132835
2897.000000	501.938721	5.133064
2898.000000	502.120239	5.133369
2899.000000	502.308472	5.128448
2900.000000	502.529968	5.128868
2901.000000	502.737488	5.129021
2902.000000	502.865509	5.126312
2903.000000	502.991699	5.123756
2904.000000	503.161133	5.128983
2905.000000	503.369171	5.125473
2906.000000	503.498657	5.122459
2907.000000	503.724945	5.116698
2908.000000	503.868011	5.119788
2909.000000	504.110138	5.119445
2910.000000	504.129883	5.120474

2911.000000	504.406250	5.112310
2912.000000	504.639740	5.109640
2913.000000	504.739380	5.113455
2914.000000	504.872314	5.107618
2915.000000	505.141388	5.106016
2916.000000	505.304688	5.103765
2917.000000	505.373535	5.103155
2918.000000	505.587677	5.104337
2919.000000	505.689789	5.102849
2920.000000	505.860291	5.098005
2921.000000	506.102905	5.099569
2922.000000	506.204956	5.097661
2923.000000	506.408630	5.089956
2924.000000	506.620483	5.092206
2925.000000	506.770691	5.093580
2926.000000	506.904083	5.094266
2927.000000	507.124542	5.091215
2928.000000	507.260345	5.084081
2929.000000	507.436615	5.081602
2930.000000	507.646027	5.082784
2931.000000	507.748047	5.082517
2932.000000	507.840515	5.082365
2933.000000	508.012390	5.080266
2934.000000	508.253662	5.080190
2935.000000	508.421143	5.077291
2936.000000	508.679230	5.072980

2937.000000	508.883331	5.073476
2938.000000	508.987335	5.073705
2939.000000	509.086029	5.071722
2940.000000	509.370605	5.069242
2941.000000	509.502533	5.067373
2942.000000	509.586273	5.063596
2943.000000	509.797699	5.066114
2944.000000	509.965271	5.065809
2945.000000	510.049500	5.064664
2946.000000	510.295654	5.059782
2947.000000	510.448334	5.060201
2948.000000	510.633331	5.058027
2949.000000	510.732056	5.056996
2950.000000	510.873169	5.054898
2951.000000	511.064941	5.055967
2952.000000	511.240723	5.054060
2953.000000	511.436798	5.054212
2954.000000	511.609222	5.050397
2955.000000	511.704620	5.045629
2956.000000	511.935333	5.047574
2957.000000	512.130371	5.048337
2958.000000	512.258057	5.045323
2959.000000	512.444885	5.044522
2960.000000	512.679932	5.044522
2961.000000	512.829285	5.039182
2962.000000	512.916016	5.035634

2963.000000	513.106812	5.039792
2964.000000	513.304749	5.039983
2965.000000	513.405457	5.031819
2966.000000	513.622253	5.031171
2967.000000	513.778320	5.034795
2968.000000	513.885193	5.029569
2969.000000	514.130859	5.027509
2970.000000	514.266235	5.028501
2971.000000	514.460388	5.026670
2972.000000	514.574951	5.023923
2973.000000	514.786438	5.024266
2974.000000	514.856812	5.020795
2975.000000	515.071106	5.023923
2976.000000	515.276855	5.020490
2977.000000	515.374146	5.017819
2978.000000	515.605347	5.013433
2979.000000	515.722839	5.014272
2980.000000	515.861145	5.017171
2981.000000	516.094727	5.014729
2982.000000	516.210876	5.015111
2983.000000	516.400574	5.012479
2984.000000	516.503662	5.009389
2985.000000	516.707947	5.005574
2986.000000	516.887634	5.004582
2987.000000	517.064880	5.006032
2988.000000	517.232910	5.006566

2989.000000	517.454102	5.004239
2990.000000	517.470459	5.000271
2991.000000	517.654480	4.997334
2992.000000	517.873596	4.998173
2993.000000	518.079834	4.992908
2994.000000	518.167969	4.992489
2995.000000	518.379395	4.996304
2996.000000	518.485352	4.995884
2997.000000	518.581787	4.995312
2998.000000	518.762329	4.989170
2999.000000	518.985840	4.994053
3000.000000	519.164551	4.986118
3001.000000	519.330322	4.986271
3002.000000	519.490234	4.985088
3003.000000	519.668457	4.984249
3004.000000	519.835083	4.985966
3005.000000	520.000488	4.982800
3006.000000	520.172424	4.979710
3007.000000	520.330811	4.981731
3008.000000	520.454773	4.977917
3009.000000	520.661255	4.977077
3010.000000	520.835327	4.977917
3011.000000	521.021301	4.974827
3012.000000	521.202515	4.974407
3013.000000	521.310303	4.972729
3014.000000	521.551086	4.970325

3015.000000	521.644104	4.967846
3016.000000	521.822876	4.966740
3017.000000	522.047485	4.965405
3018.000000	522.242615	4.965290
3019.000000	522.486755	4.965099
3020.000000	522.638367	4.960560
3021.000000	522.821533	4.962963
3022.000000	522.978821	4.961628
3023.000000	523.175781	4.957241
3024.000000	523.327454	4.955715
3025.000000	523.500549	4.956364
3026.000000	523.624084	4.955334
3027.000000	523.775696	4.954037
3028.000000	523.942139	4.954838
3029.000000	524.084229	4.955029
3030.000000	524.222534	4.951214
3031.000000	524.417603	4.951176
3032.000000	524.596436	4.947285
3033.000000	524.731384	4.945988
3034.000000	524.963623	4.947094
3035.000000	525.064209	4.945835
3036.000000	525.208679	4.938168
3037.000000	525.284973	4.940456
3038.000000	525.466248	4.939846
3039.000000	525.601685	4.938969
3040.000000	525.811523	4.941334

3041.000000	525.996094	4.938244
3042.000000	526.151062	4.936069
3043.000000	526.287354	4.931835
3044.000000	526.449036	4.931644
3045.000000	526.618835	4.933018
3046.000000	526.767578	4.929852
3047.000000	526.992676	4.927525
3048.000000	527.132874	4.927296
3049.000000	527.346130	4.927677
3050.000000	527.447205	4.927029
3051.000000	527.619263	4.928860
3052.000000	527.807678	4.929546
3053.000000	528.043701	4.922832
3054.000000	528.239258	4.920353
3055.000000	528.316956	4.920238
3056.000000	528.479492	4.920887
3057.000000	528.778564	4.917835
3058.000000	528.933105	4.917416
3059.000000	529.125732	4.916309
3060.000000	529.221069	4.914364
3061.000000	529.384216	4.911312
3062.000000	529.608337	4.913906
3063.000000	529.770996	4.912151
3064.000000	529.934570	4.908337
3065.000000	530.046082	4.908833
3066.000000	530.201050	4.909939

3067.000000	530.380371	4.908451
3068.000000	530.491028	4.907803
3069.000000	530.686096	4.903225
3070.000000	530.850586	4.904713
3071.000000	530.964111	4.900517
3072.000000	531.193970	4.901623
3073.000000	531.362732	4.900517
3074.000000	531.514893	4.896816
3075.000000	531.690796	4.897961
3076.000000	531.884460	4.895176
3077.000000	531.977417	4.893421
3078.000000	532.142883	4.895214
3079.000000	532.353699	4.897083
3080.000000	532.552979	4.893307
3081.000000	532.697998	4.892773
3082.000000	532.873962	4.887470
3083.000000	532.988403	4.890026
3084.000000	533.204895	4.890674
3085.000000	533.376038	4.886096
3086.000000	533.513000	4.886936
3087.000000	533.682678	4.883615
3088.000000	533.950745	4.881594
3089.000000	534.171997	4.879572
3090.000000	534.288818	4.878541
3091.000000	534.355530	4.878351
3092.000000	534.622620	4.878160

3093.000000	534.645508	4.875452
3094.000000	534.896790	4.875680
3095.000000	535.119080	4.876176
3096.000000	535.204407	4.872324
3097.000000	535.366516	4.874307
3098.000000	535.564453	4.870836
3099.000000	535.679382	4.873506
3100.000000	535.875854	4.870416
3101.000000	536.059448	4.871294
3102.000000	536.133362	4.867898
3103.000000	536.339355	4.865381
3104.000000	536.530151	4.865686
3105.000000	536.685547	4.862176
3106.000000	536.839111	4.863664
3107.000000	536.980774	4.865495
3108.000000	537.152405	4.858972
3109.000000	537.341309	4.858629
3110.000000	537.437195	4.853746
3111.000000	537.606934	4.856874
3112.000000	537.759949	4.856607
3113.000000	537.986023	4.850999
3114.000000	538.155273	4.855348
3115.000000	538.347961	4.852678
3116.000000	538.474854	4.852258
3117.000000	538.618347	4.850923
3118.000000	538.786194	4.847070

3119.000000	538.942139	4.849092
3120.000000	539.081787	4.850236
3121.000000	539.255920	4.845201
3122.000000	539.458069	4.842226
3123.000000	539.613525	4.845468
3124.000000	539.770996	4.843294
3125.000000	539.984131	4.843141
3126.000000	540.118103	4.840471
3127.000000	540.268799	4.838182
3128.000000	540.523926	4.838678
3129.000000	540.739929	4.838144
3130.000000	540.939758	4.835741
3131.000000	541.083740	4.835664
3132.000000	541.203003	4.834329
3133.000000	541.369019	4.835550
3134.000000	541.552490	4.830781
3135.000000	541.734192	4.829561
3136.000000	541.947449	4.829942
3137.000000	542.104736	4.828760
3138.000000	542.330750	4.828531
3139.000000	542.454834	4.825822
3140.000000	542.673706	4.824258
3141.000000	542.884888	4.825326
3142.000000	543.016479	4.822885
3143.000000	543.228271	4.822846
3144.000000	543.374634	4.824449

3145.000000	543.565430	4.819680
3146.000000	543.732727	4.818345
3147.000000	543.859619	4.819337
3148.000000	543.999329	4.819451
3149.000000	544.177734	4.814607
3150.000000	544.231140	4.809609
3151.000000	544.398438	4.817163
3152.000000	544.525330	4.813844
3153.000000	544.683167	4.814759
3154.000000	544.853943	4.809495
3155.000000	545.036011	4.809152
3156.000000	545.160522	4.809114
3157.000000	545.280762	4.809037
3158.000000	545.436157	4.806024
3159.000000	545.538696	4.803506
3160.000000	545.688965	4.803086
3161.000000	545.921143	4.802857
3162.000000	546.079468	4.800111
3163.000000	546.254517	4.799806
3164.000000	546.449951	4.794541
3165.000000	546.611633	4.797021
3166.000000	546.832947	4.794732
3167.000000	547.040771	4.798776
3168.000000	547.182007	4.796754
3169.000000	547.283630	4.792253
3170.000000	547.504822	4.794580

3171.000000	547.687439	4.793015
3172.000000	547.812927	4.793283
3173.000000	548.036011	4.789124
3174.000000	548.227295	4.789582
3175.000000	548.363220	4.789201
3176.000000	548.534424	4.789392
3177.000000	548.660828	4.788514
3178.000000	548.801025	4.785157
3179.000000	548.931152	4.779092
3180.000000	549.187744	4.782868
3181.000000	549.290771	4.784852
3182.000000	549.476746	4.783402
3183.000000	549.717529	4.781762
3184.000000	549.830078	4.780923
3185.000000	549.978821	4.778863
3186.000000	550.177734	4.777719
3187.000000	550.306885	4.777146
3188.000000	550.550537	4.775811
3189.000000	550.619324	4.775086
3190.000000	550.841492	4.774552
3191.000000	550.984619	4.774743
3192.000000	551.154785	4.770089
3193.000000	551.289307	4.770814
3194.000000	551.492920	4.769555
3195.000000	551.657410	4.765892
3196.000000	551.800049	4.766693

3197.000000	551.974609	4.765817
3198.000000	552.159607	4.764824
3199.000000	552.301270	4.766121
3200.000000	552.420898	4.765549
3201.000000	552.648926	4.764023
3202.000000	552.766113	4.761581
3203.000000	553.000366	4.759789
3204.000000	553.155762	4.759903
3205.000000	553.333191	4.757462
3206.000000	553.495850	4.754105
3207.000000	553.692261	4.755173
3208.000000	553.877808	4.754753
3209.000000	553.982178	4.750900
3210.000000	554.141907	4.753418
3211.000000	554.336426	4.754906
3212.000000	554.589294	4.753990
3213.000000	554.739990	4.748230
3214.000000	554.851563	4.749603
3215.000000	555.069885	4.747200
3216.000000	555.277344	4.748878
3217.000000	555.410889	4.747467
3218.000000	555.543457	4.749756
3219.000000	555.660767	4.744835
3220.000000	555.825317	4.742050
3221.000000	556.085205	4.744225
3222.000000	556.151001	4.742966

3223.000000	556.324585	4.738388
3224.000000	556.483337	4.741249
3225.000000	556.625000	4.740334
3226.000000	556.830078	4.743385
3227.000000	557.037048	4.738007
3228.000000	557.213440	4.739342
3229.000000	557.405151	4.740334
3230.000000	557.555847	4.736862
3231.000000	557.718018	4.731026
3232.000000	557.910645	4.736519
3233.000000	558.079956	4.734840
3234.000000	558.239746	4.732819
3235.000000	558.372803	4.729500
3236.000000	558.572571	4.730186
3237.000000	558.729004	4.729347
3238.000000	558.912048	4.729500
3239.000000	559.024658	4.728012
3240.000000	559.202515	4.723511
3241.000000	559.377014	4.723854
3242.000000	559.510559	4.726715
3243.000000	559.608826	4.724655
3244.000000	559.873474	4.725571
3245.000000	560.020752	4.721909
3246.000000	560.229675	4.720039
3247.000000	560.313171	4.718285
3248.000000	560.514709	4.722023

3249.000000	560.759216	4.718552
3250.000000	560.922485	4.720268
3251.000000	560.978088	4.718323
3252.000000	561.111633	4.714622
3253.000000	561.272156	4.713097
3254.000000	561.431641	4.713631
3255.000000	561.638367	4.710693
3256.000000	561.880432	4.712791
3257.000000	562.013428	4.708710
3258.000000	562.156982	4.713364
3259.000000	562.284424	4.710007
3260.000000	562.352844	4.711189
3261.000000	562.575623	4.709740
3262.000000	562.653442	4.699287
3263.000000	562.884216	4.703865
3264.000000	563.067749	4.705620
3265.000000	563.309875	4.702873
3266.000000	563.370789	4.705849
3267.000000	563.527466	4.704475
3268.000000	563.744934	4.700508
3269.000000	563.891357	4.701233
3270.000000	564.082886	4.699783
3271.000000	564.208008	4.700928
3272.000000	564.342041	4.701653
3273.000000	564.500549	4.698906
3274.000000	564.741211	4.698257

3275.000000	564.891785	4.698601
3276.000000	565.031433	4.692612
3277.000000	565.201843	4.694557
3278.000000	565.323120	4.692574
3279.000000	565.552490	4.697342
3280.000000	565.760559	4.694252
3281.000000	565.958801	4.696045
3282.000000	566.085205	4.690361
3283.000000	566.361755	4.692955
3284.000000	566.508057	4.690552
3285.000000	566.607178	4.687462
3286.000000	566.781738	4.691200
3287.000000	567.030518	4.688034
3288.000000	567.225342	4.687347
3289.000000	567.354736	4.686203
3290.000000	567.483093	4.683723
3291.000000	567.661072	4.685516
3292.000000	567.896484	4.680634
3293.000000	568.003174	4.682541
3294.000000	568.203186	4.680443
3295.000000	568.362305	4.685516
3296.000000	568.547241	4.682312
3297.000000	568.735107	4.679794
3298.000000	568.819519	4.680481
3299.000000	568.982422	4.682274
3300.000000	569.160278	4.680061

3301.000000	569.414185	4.677811
3302.000000	569.461853	4.676666
3303.000000	569.648254	4.677315
3304.000000	569.805847	4.679871
3305.000000	569.980469	4.673576
3306.000000	570.103760	4.675903
3307.000000	570.304810	4.675789
3308.000000	570.529541	4.672546
3309.000000	570.662231	4.674530
3310.000000	570.805298	4.671249
3311.000000	570.998413	4.671440
3312.000000	571.104675	4.667740
3313.000000	571.287354	4.673538
3314.000000	571.439392	4.670334
3315.000000	571.628235	4.668007
3316.000000	571.755737	4.665337
3317.000000	571.964966	4.669380
3318.000000	572.090027	4.668159
3319.000000	572.245361	4.664841
3320.000000	572.375244	4.664383
3321.000000	572.531006	4.665565
3322.000000	572.677307	4.662704
3323.000000	572.904968	4.665833
3324.000000	573.101318	4.664993
3325.000000	573.238281	4.661903
3326.000000	573.472412	4.665909

3327.000000	573.589111	4.660912
3328.000000	573.723633	4.664116
3329.000000	573.831787	4.662781
3330.000000	574.108887	4.661865
3331.000000	574.241577	4.661179
3332.000000	574.446533	4.655113
3333.000000	574.602783	4.657326
3334.000000	574.787842	4.657936
3335.000000	574.937073	4.654769
3336.000000	575.219849	4.657402
3337.000000	575.353027	4.652784
3338.000000	575.507813	4.653816
3339.000000	575.646729	4.653434
3340.000000	575.783997	4.654999
3341.000000	575.931885	4.655266
3342.000000	576.110779	4.647939
3343.000000	576.285522	4.651221
3344.000000	576.407715	4.653319
3345.000000	576.660828	4.652442
3346.000000	576.786865	4.649618
3347.000000	576.932373	4.646566
3348.000000	577.095703	4.649618
3349.000000	577.333191	4.643171
3350.000000	577.504578	4.649275
3351.000000	577.643799	4.645269
3352.000000	577.771790	4.645498

3353.000000	577.979553	4.645193
3354.000000	578.135803	4.639738
3355.000000	578.341675	4.644620
3356.000000	578.542358	4.638937
3357.000000	578.691528	4.638135
3358.000000	578.825134	4.639127
3359.000000	579.018311	4.640691
3360.000000	579.162720	4.637945
3361.000000	579.348694	4.636495
3362.000000	579.581055	4.639394
3363.000000	579.749573	4.638326
3364.000000	579.818542	4.635656
3365.000000	579.942566	4.637258
3366.000000	580.159302	4.633482
3367.000000	580.316406	4.639814
3368.000000	580.488403	4.636686
3369.000000	580.667480	4.634778
3370.000000	580.864258	4.636152
3371.000000	580.975586	4.634359
3372.000000	581.093750	4.633482
3373.000000	581.238770	4.630926
3374.000000	581.501526	4.630544
3375.000000	581.626282	4.635465
3376.000000	581.835266	4.633787
3377.000000	582.017090	4.628141
3378.000000	582.200439	4.629781

3379.000000	582.366394	4.632337
3380.000000	582.515442	4.629209
3381.000000	582.643616	4.628599
3382.000000	582.759094	4.634397
3383.000000	582.947937	4.628332
3384.000000	583.070862	4.628522
3385.000000	583.194824	4.627454
3386.000000	583.327576	4.626119
3387.000000	583.587952	4.625432
3388.000000	583.710449	4.629819
3389.000000	583.890015	4.625585
3390.000000	584.131226	4.625432
3391.000000	584.306519	4.625852
3392.000000	584.452393	4.624784
3393.000000	584.540771	4.621389
3394.000000	584.766541	4.622228
3395.000000	584.970398	4.622953
3396.000000	585.132568	4.622533
3397.000000	585.380859	4.622457
3398.000000	585.510803	4.621351
3399.000000	585.625732	4.619939
3400.000000	585.761353	4.618948
3401.000000	586.037781	4.621961
3402.000000	586.200928	4.618185
3403.000000	586.374817	4.620397
3404.000000	586.528564	4.616811

3405.000000	586.709961	4.614141
3406.000000	586.860046	4.618757
3407.000000	587.002197	4.614141
3408.000000	587.235046	4.618185
3409.000000	587.377991	4.613874
3410.000000	587.471497	4.610670
3411.000000	587.651550	4.616544
3412.000000	587.856201	4.615209
3413.000000	588.010620	4.613798
3414.000000	588.179749	4.618337
3415.000000	588.303223	4.613454
3416.000000	588.479919	4.617078
3417.000000	588.656677	4.612119
3418.000000	588.811951	4.613035
3419.000000	588.969421	4.610326
3420.000000	589.205505	4.611127
3421.000000	589.424316	4.614370
3422.000000	589.599670	4.611394
3423.000000	589.739868	4.611394
3424.000000	589.924561	4.612806
3425.000000	590.018555	4.613264
3426.000000	590.170288	4.610250
3427.000000	590.336914	4.614484
3428.000000	590.531494	4.612157
3429.000000	590.744873	4.609601
3430.000000	590.911987	4.609373

3431.000000	590.985107	4.605024
3432.000000	591.205750	4.609449
3433.000000	591.349670	4.609449
3434.000000	591.496887	4.608991
3435.000000	591.693909	4.609945
3436.000000	591.930420	4.609487
3437.000000	592.099915	4.608152
3438.000000	592.246338	4.606626
3439.000000	592.424316	4.605787
3440.000000	592.614075	4.607580
3441.000000	592.656067	4.608648
3442.000000	592.920959	4.604452
3443.000000	593.043274	4.608457
3444.000000	593.280273	4.606817
3445.000000	593.363892	4.606855
3446.000000	593.595642	4.607122
3447.000000	593.766113	4.612234
3448.000000	593.833191	4.606588
3449.000000	594.043274	4.608305
3450.000000	594.179260	4.611776
3451.000000	594.261780	4.612272
3452.000000	594.480957	4.608877
3453.000000	594.659851	4.602354
3454.000000	594.819397	4.606054
3455.000000	595.001709	4.607122
3456.000000	595.157959	4.601552

3457.000000	595.301514	4.599226
3458.000000	595.483276	4.605100
3459.000000	595.691040	4.606016
3460.000000	595.773193	4.605520
3461.000000	595.973816	4.604566
3462.000000	596.125488	4.604795
3463.000000	596.292603	4.608686
3464.000000	596.465820	4.605482
3465.000000	596.619690	4.601743
3466.000000	596.792603	4.599874
3467.000000	596.991333	4.604566
3468.000000	597.085754	4.600637
3469.000000	597.348267	4.601285
3470.000000	597.531921	4.602964
3471.000000	597.726929	4.599264
3472.000000	597.924805	4.598348
3473.000000	598.134827	4.601095
3474.000000	598.243958	4.601209
3475.000000	598.459717	4.597852
3476.000000	598.698608	4.599683
3477.000000	598.811829	4.603689
3478.000000	599.014893	4.601858
3479.000000	599.147095	4.601476
3480.000000	599.271790	4.600255
3481.000000	599.456421	4.596555
3482.000000	599.507324	4.597738

3483.000000	599.792053	4.597127
3484.000000	599.911987	4.596555

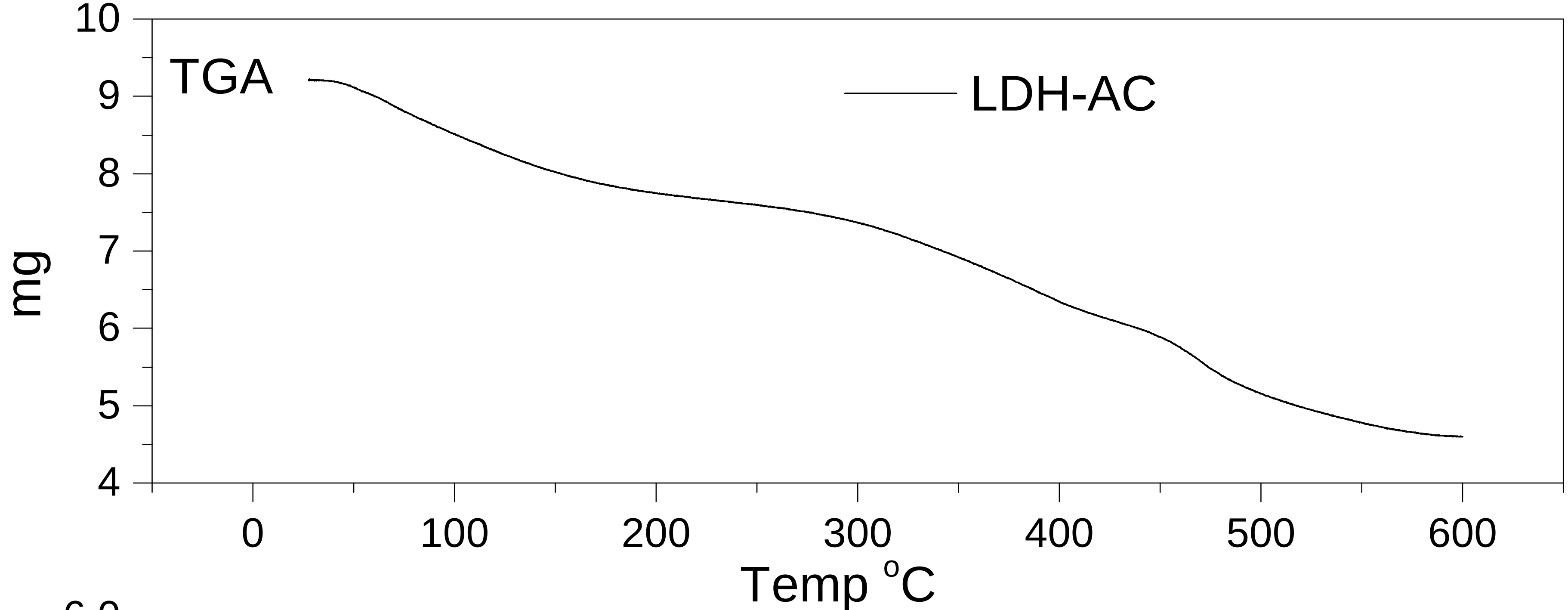
TGA
LDH-AC
mg
Temp °C

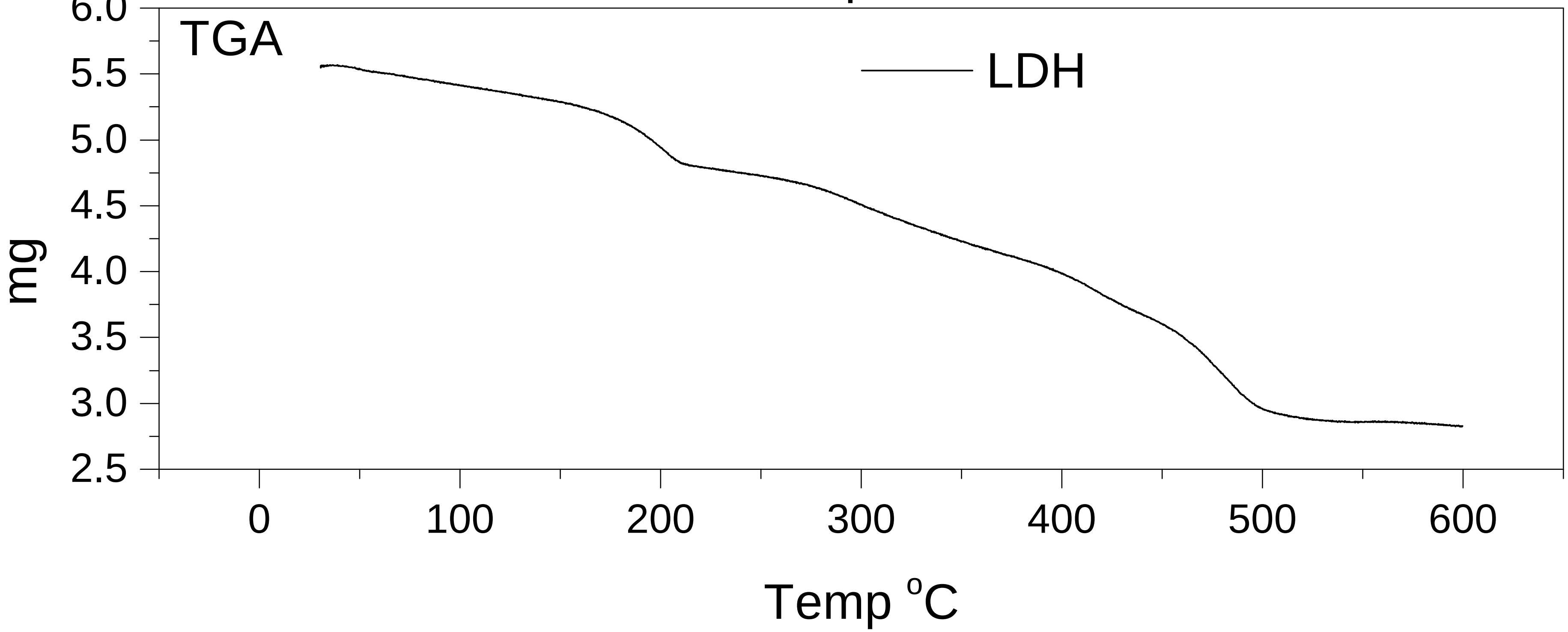
TGA
LDH
mg
Temp °C

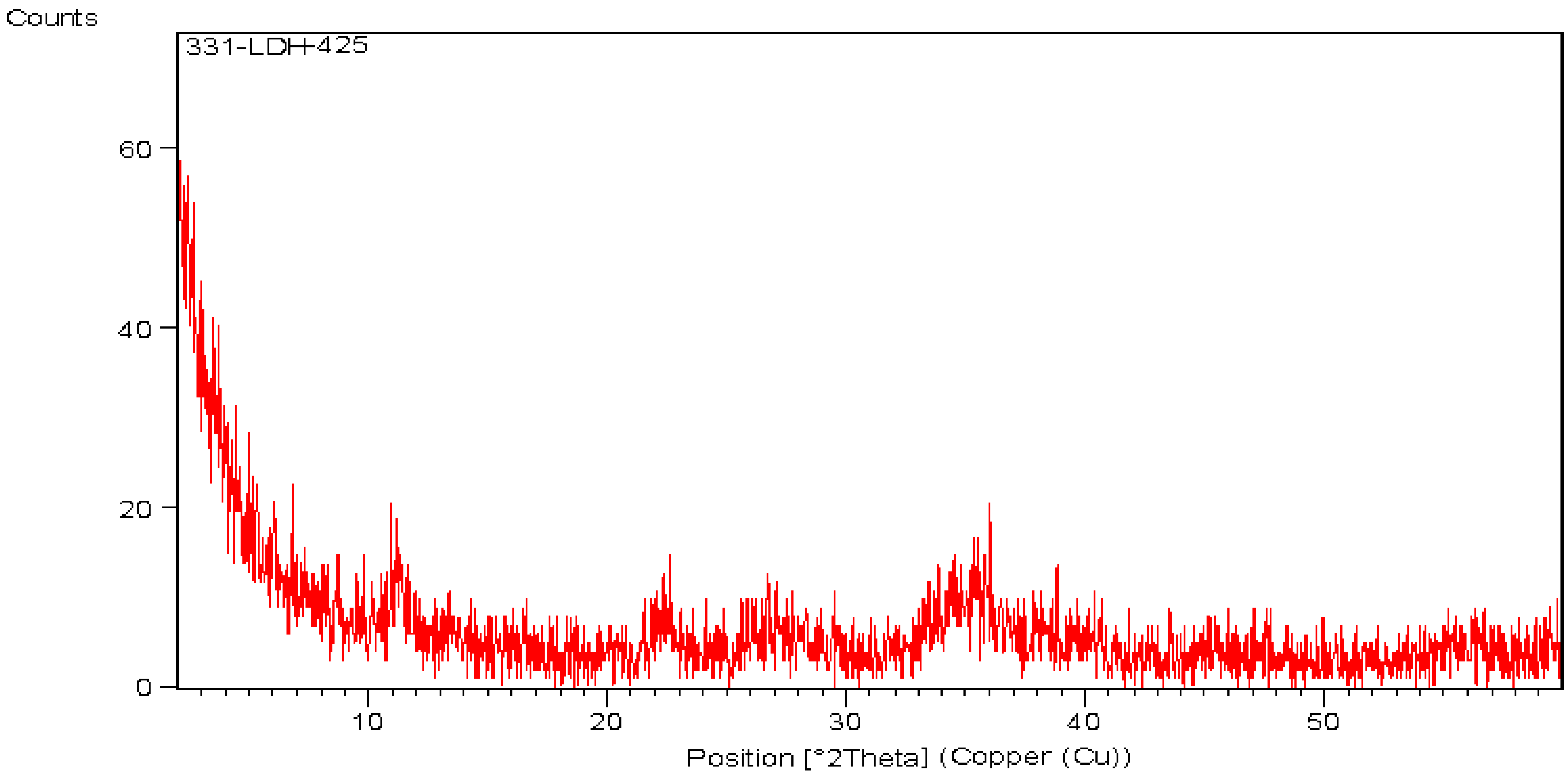
Counts
331-LDH-425
60
40
20
0
10
20
30
40
50
Position [°2Theta] (Copper (Cu))

Pos. [°2Th.] Iobs [cts]

2.025073.0000

2.055065.0000

2.085062.0000

2.115055.0000

2.145053.0000

2.175053.0000

2.205050.0000

2.235047.0000

2.265057.0000

2.295056.0000

2.325047.0000

2.355044.0000

2.385043.0000

2.415055.0000

2.445052.0000

2.475058.0000

2.505041.0000

2.535042.0000

2.565050.0000

2.595051.0000

2.625043.0000

2.655055.0000

2.685038.0000

2.715053.0000

2.745042.0000

2.775042.0000

2.805042.0000

2.835036.0000

2.865033.0000

2.895033.0000

2.925044.0000

2.955032.0000

2.985046.0000

3.015029.0000

3.045034.0000

3.075043.0000

3.105033.0000

3.135038.0000

3.165037.0000

3.195032.0000

3.225031.0000

3.255036.0000

3.285034.0000

3.315027.0000

3.345033.0000

3.375028.0000

3.405023.0000

3.435034.0000

3.465035.0000

3.495031.0000

3.525042.0000

3.555031.0000

3.585029.0000

3.615029.0000

3.645030.0000

3.675034.0000

3.705041.0000

3.735041.0000

3.765025.0000

3.795034.0000

3.825027.0000

3.855029.0000

3.885023.0000

3.915021.0000

3.945032.0000

3.975024.0000

4.005026.0000

4.035026.0000

4.065030.0000

4.095028.0000

4.125015.0000

4.155021.0000

4.185024.0000

4.215025.0000

4.245020.0000

4.275025.0000

4.305028.0000

4.335021.0000

4.365014.0000

4.395026.0000

4.425032.0000

4.455020.0000

4.485024.0000

4.515023.0000

4.545020.0000

4.575023.0000

4.605025.0000

4.635020.0000

4.665021.0000

4.695015.0000

4.725015.0000

4.755014.0000

4.785020.0000

4.815018.0000

4.845016.0000

4.875017.0000

4.905014.0000

4.935016.0000

4.965022.0000

4.995013.0000

5.025029.0000

5.055015.0000

5.085021.0000

5.115018.0000

5.145018.0000

5.175021.0000

5.205024.0000

5.235012.0000

5.265012.0000

5.295020.0000

5.325020.0000

5.355023.0000

5.385016.0000

5.415013.0000

5.445012.0000

5.475013.0000

5.505014.0000

5.535017.0000

5.565015.0000

5.595015.0000

5.625013.0000

5.655013.0000

5.685012.0000

5.715014.0000

5.745015.0000

5.775017.0000

5.805017.0000

5.83509.0000

5.865013.0000

5.895018.0000

5.925012.0000

5.955012.0000

5.985017.0000

6.015016.0000

6.045021.0000

6.075019.0000

6.105019.0000

6.135011.0000

6.165015.0000

6.19509.0000

6.225014.0000

6.255011.0000

6.285013.0000

6.315011.0000

6.345012.0000

6.375012.0000

6.405013.0000

6.435012.0000

6.46509.0000

6.495013.0000

6.525010.0000

6.555014.0000

6.585012.0000

6.615012.0000

6.64506.0000

6.675013.0000

6.70506.0000

6.73509.0000

6.765013.0000

6.795011.0000

6.825023.0000

6.855013.0000

6.88508.0000

6.915014.0000

6.945014.0000

6.975013.0000

7.005015.0000

7.03507.0000

7.065010.0000

7.095010.0000

7.12508.0000

7.155014.0000

7.18509.0000

7.215013.0000

7.24508.0000

7.275011.0000

7.305016.0000

7.335010.0000

7.365011.0000

7.395011.0000

7.42509.0000

7.455013.0000

7.48509.0000

7.515010.0000

7.54509.0000

7.575011.0000

7.605010.0000

7.63507.0000

7.665012.0000

7.695013.0000

7.725010.0000

7.75507.0000

7.785012.0000

7.815010.0000

7.84507.0000

7.875012.0000

7.905010.0000

7.93506.0000

7.965011.0000

7.995011.0000

8.025011.0000

8.055014.0000

8.08505.0000

8.11508.0000

8.145013.0000

8.175014.0000

8.205010.0000

8.23507.0000

8.26509.0000

8.295014.0000

8.32507.0000

8.35509.0000

8.38503.0000

8.41506.0000

8.44507.0000

8.47506.0000

8.50504.0000

8.53508.0000

8.56509.0000

8.595011.0000

8.625011.0000

8.65509.0000

8.685015.0000

8.715015.0000

8.74508.0000

8.77507.0000

8.805010.0000

8.83509.0000

8.86507.0000

8.895010.0000

8.92503.0000

8.95509.0000

8.98506.0000

9.01505.0000

9.04508.0000

9.07507.0000

9.10506.0000

9.13507.0000

9.16504.0000

9.19506.0000

9.22507.0000

9.25509.0000

9.28507.0000

9.31509.0000

9.34508.0000

9.37505.0000

9.40506.0000

9.43505.0000

9.46508.0000

9.495013.0000

9.52506.0000

9.55506.0000

9.585012.0000

9.61509.0000

9.64509.0000

9.67505.0000

9.70506.0000

9.73508.0000

9.765011.0000

9.79507.0000

9.825015.0000

9.85506.0000

9.88508.0000

9.91507.0000

9.94504.0000

9.97504.0000

10.0050	3.0000
10.0350	7.0000
10.0650	8.0000
10.0950	8.0000
10.1250	12.0000
10.1550	10.0000
10.1850	7.0000
10.2150	9.0000
10.2450	8.0000
10.2750	4.0000
10.3050	7.0000
10.3350	5.0000
10.3650	8.0000
10.3950	8.0000
10.4250	9.0000
10.4550	7.0000
10.4850	6.0000
10.5150	7.0000
10.5450	13.0000

10.5750	5.0000
10.6050	9.0000
10.6350	10.0000
10.6650	10.0000
10.6950	12.0000
10.7250	3.0000
10.7550	3.0000
10.7850	13.0000
10.8150	7.0000
10.8450	7.0000
10.8750	9.0000
10.9050	16.0000
10.9350	21.0000
10.9650	13.0000
10.9950	7.0000
11.0250	13.0000
11.0550	13.0000
11.0850	14.0000
11.1150	7.0000
11.1450	19.0000
11.1750	12.0000
11.2050	16.0000
11.2350	16.0000
11.2650	9.0000
11.2950	15.0000
11.3250	14.0000

11.3550	12.0000
11.3850	12.0000
11.4150	12.0000
11.4450	14.0000
11.4750	7.0000
11.5050	10.0000
11.5350	6.0000
11.5650	5.0000
11.5950	13.0000
11.6250	14.0000
11.6550	8.0000
11.6850	6.0000
11.7150	7.0000
11.7450	12.0000
11.7750	6.0000
11.8050	6.0000
11.8350	6.0000
11.8650	6.0000
11.8950	9.0000
11.9250	11.0000
11.9550	4.0000
11.9850	5.0000
12.0150	5.0000
12.0450	8.0000
12.0750	7.0000
12.1050	4.0000

12.1350	5.0000
12.1650	10.0000
12.1950	4.0000
12.2250	6.0000
12.2550	8.0000
12.2850	2.0000
12.3150	7.0000
12.3450	8.0000
12.3750	4.0000
12.4050	5.0000
12.4350	7.0000
12.4650	3.0000
12.4950	6.0000
12.5250	7.0000
12.5550	4.0000
12.5850	7.0000
12.6150	7.0000
12.6450	6.0000
12.6750	5.0000
12.7050	3.0000
12.7350	8.0000
12.7650	10.0000
12.7950	1.0000
12.8250	2.0000
12.8550	8.0000
12.8850	9.0000

12.9150	4.0000
12.9450	2.0000
12.9750	7.0000
13.0050	8.0000
13.0350	4.0000
13.0650	5.0000
13.0950	3.0000
13.1250	9.0000
13.1550	8.0000
13.1850	4.0000
13.2150	4.0000
13.2450	7.0000
13.2750	8.0000
13.3050	3.0000
13.3350	9.0000
13.3650	11.0000
13.3950	8.0000
13.4250	8.0000
13.4550	6.0000
13.4850	8.0000
13.5150	5.0000
13.5450	5.0000
13.5750	8.0000
13.6050	7.0000
13.6350	6.0000
13.6650	6.0000

13.6950	4.0000
13.7250	5.0000
13.7550	5.0000
13.7850	8.0000
13.8150	7.0000
13.8450	6.0000
13.8750	3.0000
13.9050	4.0000
13.9350	3.0000
13.9650	5.0000
13.9950	3.0000
14.0250	6.0000
14.0550	6.0000
14.0850	7.0000
14.1150	1.0000
14.1450	4.0000
14.1750	6.0000
14.2050	8.0000
14.2350	4.0000
14.2650	4.0000
14.2950	10.0000
14.3250	3.0000
14.3550	5.0000
14.3850	6.0000
14.4150	3.0000
14.4450	1.0000

14.4750	9.0000
14.5050	4.0000
14.5350	1.0000
14.5650	7.0000
14.5950	1.0000
14.6250	4.0000
14.6550	5.0000
14.6850	6.0000
14.7150	4.0000
14.7450	3.0000
14.7750	5.0000
14.8050	4.0000
14.8350	6.0000
14.8650	7.0000
14.8950	6.0000
14.9250	1.0000
14.9550	2.0000
14.9850	8.0000
15.0150	2.0000
15.0450	3.0000
15.0750	5.0000
15.1050	8.0000
15.1350	1.0000
15.1650	5.0000
15.1950	8.0000
15.2250	1.0000

15.2550	1.0000
15.2850	4.0000
15.3150	8.0000
15.3450	6.0000
15.3750	7.0000
15.4050	4.0000
15.4350	4.0000
15.4650	4.0000
15.4950	6.0000
15.5250	4.0000
15.5550	3.0000
15.5850	0.0000
15.6150	3.0000
15.6450	8.0000
15.6750	6.0000
15.7050	4.0000
15.7350	6.0000
15.7650	8.0000
15.7950	6.0000
15.8250	5.0000
15.8550	1.0000
15.8850	6.0000
15.9150	4.0000
15.9450	2.0000
15.9750	7.0000
16.0050	4.0000

16.0350	5.0000
16.0650	9.0000
16.0950	8.0000
16.1250	4.0000
16.1550	3.0000
16.1850	7.0000
16.2150	1.0000
16.2450	3.0000
16.2750	6.0000
16.3050	6.0000
16.3350	3.0000
16.3650	1.0000
16.3950	3.0000
16.4250	9.0000
16.4550	8.0000
16.4850	8.0000
16.5150	5.0000
16.5450	8.0000
16.5750	4.0000
16.6050	2.0000
16.6350	10.0000
16.6650	6.0000
16.6950	4.0000
16.7250	3.0000
16.7550	1.0000
16.7850	6.0000

16.8150	4.0000
16.8450	3.0000
16.8750	3.0000
16.9050	7.0000
16.9350	2.0000
16.9650	3.0000
16.9950	6.0000
17.0250	2.0000
17.0550	3.0000
17.0850	6.0000
17.1150	2.0000
17.1450	5.0000
17.1750	4.0000
17.2050	2.0000
17.2350	7.0000
17.2650	5.0000
17.2950	2.0000
17.3250	5.0000
17.3550	3.0000
17.3850	2.0000
17.4150	1.0000
17.4450	2.0000
17.4750	5.0000
17.5050	2.0000
17.5350	5.0000
17.5650	1.0000

17.5950	8.0000
17.6250	3.0000
17.6550	5.0000
17.6850	7.0000
17.7150	3.0000
17.7450	2.0000
17.7750	5.0000
17.8050	2.0000
17.8350	0.0000
17.8650	8.0000
17.8950	5.0000
17.9250	2.0000
17.9550	4.0000
17.9850	2.0000
18.0150	4.0000
18.0450	3.0000
18.0750	4.0000
18.1050	0.0000
18.1350	4.0000
18.1650	1.0000
18.1950	2.0000
18.2250	5.0000
18.2550	4.0000
18.2850	8.0000
18.3150	5.0000
18.3450	5.0000

18.3750	5.0000
18.4050	1.0000
18.4350	8.0000
18.4650	5.0000
18.4950	6.0000
18.5250	4.0000
18.5550	6.0000
18.5850	1.0000
18.6150	0.0000
18.6450	5.0000
18.6750	8.0000
18.7050	3.0000
18.7350	7.0000
18.7650	0.0000
18.7950	7.0000
18.8250	2.0000
18.8550	4.0000
18.8850	3.0000
18.9150	4.0000
18.9450	3.0000
18.9750	7.0000
19.0050	2.0000
19.0350	1.0000
19.0650	4.0000
19.0950	3.0000
19.1250	2.0000

19.1550	3.0000
19.1850	0.0000
19.2150	5.0000
19.2450	5.0000
19.2750	4.0000
19.3050	1.0000
19.3350	1.0000
19.3650	4.0000
19.3950	4.0000
19.4250	2.0000
19.4550	4.0000
19.4850	3.0000
19.5150	0.0000
19.5450	3.0000
19.5750	6.0000
19.6050	2.0000
19.6350	3.0000
19.6650	7.0000
19.6950	6.0000
19.7250	2.0000
19.7550	4.0000
19.7850	5.0000
19.8150	3.0000
19.8450	3.0000
19.8750	3.0000
19.9050	4.0000

19.9350	4.0000
19.9650	4.0000
19.9950	5.0000
20.0250	3.0000
20.0550	7.0000
20.0850	6.0000
20.1150	7.0000
20.1450	6.0000
20.1750	4.0000
20.2050	4.0000
20.2350	5.0000
20.2650	0.0000
20.2950	4.0000
20.3250	5.0000
20.3550	6.0000
20.3850	6.0000
20.4150	3.0000
20.4450	6.0000
20.4750	4.0000
20.5050	5.0000
20.5350	5.0000
20.5650	5.0000
20.5950	4.0000
20.6250	7.0000
20.6550	1.0000
20.6850	4.0000

20.7150	4.0000
20.7450	7.0000
20.7750	4.0000
20.8050	5.0000
20.8350	2.0000
20.8650	1.0000
20.8950	2.0000
20.9250	4.0000
20.9550	0.0000
20.9850	5.0000
21.0150	4.0000
21.0450	3.0000
21.0750	2.0000
21.1050	1.0000
21.1350	2.0000
21.1650	2.0000
21.1950	3.0000
21.2250	1.0000
21.2550	2.0000
21.2850	4.0000
21.3150	3.0000
21.3450	5.0000
21.3750	4.0000
21.4050	5.0000
21.4350	4.0000
21.4650	7.0000

21.4950	5.0000
21.5250	6.0000
21.5550	10.0000
21.5850	2.0000
21.6150	3.0000
21.6450	5.0000
21.6750	3.0000
21.7050	4.0000
21.7350	1.0000
21.7650	2.0000
21.7950	10.0000
21.8250	5.0000
21.8550	6.0000
21.8850	4.0000
21.9150	7.0000
21.9450	10.0000
21.9750	3.0000
22.0050	6.0000
22.0350	6.0000
22.0650	11.0000
22.0950	5.0000
22.1250	9.0000
22.1550	6.0000
22.1850	6.0000
22.2150	4.0000
22.2450	11.0000

22.2750	6.0000
22.3050	7.0000
22.3350	13.0000
22.3650	5.0000
22.3950	7.0000
22.4250	11.0000
22.4550	5.0000
22.4850	5.0000
22.5150	8.0000
22.5450	8.0000
22.5750	10.0000
22.6050	6.0000
22.6350	15.0000
22.6650	8.0000
22.6950	4.0000
22.7250	5.0000
22.7550	6.0000
22.7850	4.0000
22.8150	7.0000
22.8450	5.0000
22.8750	5.0000
22.9050	4.0000
22.9350	5.0000
22.9650	5.0000
22.9950	1.0000
23.0250	3.0000

23.0550	9.0000
23.0850	6.0000
23.1150	3.0000
23.1450	4.0000
23.1750	6.0000
23.2050	5.0000
23.2350	4.0000
23.2650	3.0000
23.2950	1.0000
23.3250	3.0000
23.3550	7.0000
23.3850	6.0000
23.4150	3.0000
23.4450	3.0000
23.4750	4.0000
23.5050	6.0000
23.5350	4.0000
23.5650	1.0000
23.5950	9.0000
23.6250	2.0000
23.6550	5.0000
23.6850	3.0000
23.7150	3.0000
23.7450	2.0000
23.7750	5.0000
23.8050	2.0000

23.8350	4.0000
23.8650	4.0000
23.8950	4.0000
23.9250	4.0000
23.9550	3.0000
23.9850	2.0000
24.0150	3.0000
24.0450	2.0000
24.0750	6.0000
24.1050	7.0000
24.1350	10.0000
24.1650	3.0000
24.1950	2.0000
24.2250	5.0000
24.2550	3.0000
24.2850	6.0000
24.3150	5.0000
24.3450	1.0000
24.3750	3.0000
24.4050	6.0000
24.4350	1.0000
24.4650	2.0000
24.4950	5.0000
24.5250	6.0000
24.5550	3.0000
24.5850	7.0000

24.6150	3.0000
24.6450	7.0000
24.6750	6.0000
24.7050	1.0000
24.7350	6.0000
24.7650	2.0000
24.7950	2.0000
24.8250	5.0000
24.8550	9.0000
24.8850	7.0000
24.9150	3.0000
24.9450	6.0000
24.9750	6.0000
25.0050	5.0000
25.0350	5.0000
25.0650	0.0000
25.0950	3.0000
25.1250	2.0000
25.1550	2.0000
25.1850	2.0000
25.2150	1.0000
25.2450	5.0000
25.2750	5.0000
25.3050	2.0000
25.3350	4.0000
25.3650	4.0000

25.3950	3.0000
25.4250	6.0000
25.4550	6.0000
25.4850	2.0000
25.5150	3.0000
25.5450	3.0000
25.5750	5.0000
25.6050	2.0000
25.6350	10.0000
25.6650	4.0000
25.6950	5.0000
25.7250	2.0000
25.7550	5.0000
25.7850	4.0000
25.8150	10.0000
25.8450	5.0000
25.8750	9.0000
25.9050	10.0000
25.9350	4.0000
25.9650	4.0000
25.9950	3.0000
26.0250	5.0000
26.0550	6.0000
26.0850	10.0000
26.1150	5.0000
26.1450	8.0000

26.1750	10.0000
26.2050	5.0000
26.2350	6.0000
26.2650	5.0000
26.2950	6.0000
26.3250	4.0000
26.3550	3.0000
26.3850	6.0000
26.4150	5.0000
26.4450	10.0000
26.4750	4.0000
26.5050	2.0000
26.5350	8.0000
26.5650	7.0000
26.5950	5.0000
26.6250	8.0000
26.6550	3.0000
26.6850	10.0000
26.7150	10.0000
26.7450	13.0000
26.7750	5.0000
26.8050	9.0000
26.8350	6.0000
26.8650	4.0000
26.8950	6.0000
26.9250	4.0000

26.9550	4.0000
26.9850	8.0000
27.0150	2.0000
27.0450	11.0000
27.0750	6.0000
27.1050	12.0000
27.1350	9.0000
27.1650	6.0000
27.1950	8.0000
27.2250	6.0000
27.2550	6.0000
27.2850	7.0000
27.3150	7.0000
27.3450	3.0000
27.3750	5.0000
27.4050	8.0000
27.4350	3.0000
27.4650	3.0000
27.4950	10.0000
27.5250	7.0000
27.5550	3.0000
27.5850	8.0000
27.6150	5.0000
27.6450	1.0000
27.6750	4.0000
27.7050	11.0000

27.7350	6.0000
27.7650	9.0000
27.7950	6.0000
27.8250	8.0000
27.8550	6.0000
27.8850	3.0000
27.9150	2.0000
27.9450	8.0000
27.9750	6.0000
28.0050	6.0000
28.0350	5.0000
28.0650	7.0000
28.0950	7.0000
28.1250	5.0000
28.1550	7.0000
28.1850	6.0000
28.2150	4.0000
28.2450	6.0000
28.2750	9.0000
28.3050	9.0000
28.3350	8.0000
28.3650	5.0000
28.3950	3.0000
28.4250	5.0000
28.4550	3.0000
28.4850	5.0000

28.5150	3.0000
28.5450	5.0000
28.5750	3.0000
28.6050	5.0000
28.6350	3.0000
28.6650	3.0000
28.6950	3.0000
28.7250	8.0000
28.7550	3.0000
28.7850	2.0000
28.8150	5.0000
28.8450	3.0000
28.8750	7.0000
28.9050	3.0000
28.9350	7.0000
28.9650	9.0000
28.9950	4.0000
29.0250	4.0000
29.0550	4.0000
29.0850	3.0000
29.1150	4.0000
29.1450	4.0000
29.1750	6.0000
29.2050	3.0000
29.2350	2.0000
29.2650	3.0000

29.2950	2.0000
29.3250	5.0000
29.3550	5.0000
29.3850	6.0000
29.4150	8.0000
29.4450	5.0000
29.4750	0.0000
29.5050	11.0000
29.5350	1.0000
29.5650	5.0000
29.5950	7.0000
29.6250	4.0000
29.6550	7.0000
29.6850	7.0000
29.7150	7.0000
29.7450	3.0000
29.7750	2.0000
29.8050	6.0000
29.8350	4.0000
29.8650	4.0000
29.8950	6.0000
29.9250	3.0000
29.9550	2.0000
29.9850	6.0000
30.0150	1.0000
30.0450	1.0000

30.0750	4.0000
30.1050	4.0000
30.1350	3.0000
30.1650	6.0000
30.1950	7.0000
30.2250	5.0000
30.2550	6.0000
30.2850	1.0000
30.3150	8.0000
30.3450	4.0000
30.3750	4.0000
30.4050	3.0000
30.4350	1.0000
30.4650	4.0000
30.4950	6.0000
30.5250	0.0000
30.5550	5.0000
30.5850	5.0000
30.6150	2.0000
30.6450	4.0000
30.6750	3.0000
30.7050	6.0000
30.7350	2.0000
30.7650	3.0000
30.7950	4.0000
30.8250	4.0000

30.8550	3.0000
30.8850	2.0000
30.9150	5.0000
30.9450	5.0000
30.9750	6.0000
31.0050	4.0000
31.0350	8.0000
31.0650	2.0000
31.0950	2.0000
31.1250	1.0000
31.1550	6.0000
31.1850	7.0000
31.2150	5.0000
31.2450	1.0000
31.2750	2.0000
31.3050	4.0000
31.3350	4.0000
31.3650	4.0000
31.3950	4.0000
31.4250	2.0000
31.4550	3.0000
31.4850	1.0000
31.5150	1.0000
31.5450	4.0000
31.5750	6.0000
31.6050	5.0000

31.6350	2.0000
31.6650	4.0000
31.6950	5.0000
31.7250	5.0000
31.7550	8.0000
31.7850	7.0000
31.8150	3.0000
31.8450	3.0000
31.8750	4.0000
31.9050	4.0000
31.9350	6.0000
31.9650	3.0000
31.9950	6.0000
32.0250	7.0000
32.0550	5.0000
32.0850	2.0000
32.1150	7.0000
32.1450	5.0000
32.1750	4.0000
32.2050	3.0000
32.2350	7.0000
32.2650	3.0000
32.2950	1.0000
32.3250	3.0000
32.3550	5.0000
32.3850	1.0000

32.4150	2.0000
32.4450	6.0000
32.4750	4.0000
32.5050	5.0000
32.5350	5.0000
32.5650	5.0000
32.5950	4.0000
32.6250	4.0000
32.6550	5.0000
32.6850	4.0000
32.7150	1.0000
32.7450	5.0000
32.7750	3.0000
32.8050	8.0000
32.8350	7.0000
32.8650	3.0000
32.8950	7.0000
32.9250	3.0000
32.9550	4.0000
32.9850	9.0000
33.0150	3.0000
33.0450	3.0000
33.0750	2.0000
33.1050	3.0000
33.1350	7.0000
33.1650	6.0000

33.1950	10.0000
33.2250	5.0000
33.2550	6.0000
33.2850	10.0000
33.3150	9.0000
33.3450	5.0000
33.3750	9.0000
33.4050	7.0000
33.4350	7.0000
33.4650	7.0000
33.4950	12.0000
33.5250	4.0000
33.5550	4.0000
33.5850	5.0000
33.6150	5.0000
33.6450	11.0000
33.6750	5.0000
33.7050	8.0000
33.7350	6.0000
33.7650	11.0000
33.7950	5.0000
33.8250	9.0000
33.8550	9.0000
33.8850	14.0000
33.9150	8.0000
33.9450	7.0000

33.9750	8.0000
34.0050	6.0000
34.0350	4.0000
34.0650	6.0000
34.0950	7.0000
34.1250	7.0000
34.1550	6.0000
34.1850	10.0000
34.2150	11.0000
34.2450	7.0000
34.2750	7.0000
34.3050	13.0000
34.3350	7.0000
34.3650	12.0000
34.3950	13.0000
34.4250	8.0000
34.4550	11.0000
34.4850	15.0000
34.5150	8.0000
34.5450	10.0000
34.5750	12.0000
34.6050	13.0000
34.6350	7.0000
34.6650	11.0000
34.6950	10.0000
34.7250	7.0000

34.7550	14.0000
34.7850	7.0000
34.8150	11.0000
34.8450	10.0000
34.8750	10.0000
34.9050	9.0000
34.9350	8.0000
34.9650	9.0000
34.9950	9.0000
35.0250	10.0000
35.0550	11.0000
35.0850	9.0000
35.1150	7.0000
35.1450	9.0000
35.1750	4.0000
35.2050	14.0000
35.2350	12.0000
35.2650	8.0000
35.2950	14.0000
35.3250	13.0000
35.3550	17.0000
35.3850	10.0000
35.4150	13.0000
35.4450	10.0000
35.4750	17.0000
35.5050	10.0000

35.5350	12.0000
35.5650	13.0000
35.5950	3.0000
35.6250	8.0000
35.6550	11.0000
35.6850	11.0000
35.7150	11.0000
35.7450	5.0000
35.7750	15.0000
35.8050	13.0000
35.8350	15.0000
35.8650	10.0000
35.8950	10.0000
35.9250	11.0000
35.9550	13.0000
35.9850	6.0000
36.0150	5.0000
36.0450	21.0000
36.0750	7.0000
36.1050	11.0000
36.1350	10.0000
36.1650	7.0000
36.1950	4.0000
36.2250	9.0000
36.2550	6.0000
36.2850	4.0000

36.3150	6.0000
36.3450	7.0000
36.3750	8.0000
36.4050	7.0000
36.4350	9.0000
36.4650	10.0000
36.4950	10.0000
36.5250	8.0000
36.5550	4.0000
36.5850	4.0000
36.6150	5.0000
36.6450	7.0000
36.6750	9.0000
36.7050	10.0000
36.7350	8.0000
36.7650	8.0000
36.7950	9.0000
36.8250	8.0000
36.8550	6.0000
36.8850	8.0000
36.9150	4.0000
36.9450	4.0000
36.9750	7.0000
37.0050	9.0000
37.0350	6.0000
37.0650	6.0000

37.0950	7.0000
37.1250	4.0000
37.1550	4.0000
37.1850	4.0000
37.2150	10.0000
37.2450	6.0000
37.2750	7.0000
37.3050	1.0000
37.3350	7.0000
37.3650	8.0000
37.3950	10.0000
37.4250	5.0000
37.4550	2.0000
37.4850	5.0000
37.5150	8.0000
37.5450	3.0000
37.5750	3.0000
37.6050	4.0000
37.6350	4.0000
37.6650	4.0000
37.6950	5.0000
37.7250	6.0000
37.7550	6.0000
37.7850	4.0000
37.8150	3.0000
37.8450	4.0000

37.8750	11.0000
37.9050	6.0000
37.9350	9.0000
37.9650	8.0000
37.9950	9.0000
38.0250	5.0000
38.0550	4.0000
38.0850	8.0000
38.1150	3.0000
38.1450	3.0000
38.1750	11.0000
38.2050	5.0000
38.2350	9.0000
38.2650	8.0000
38.2950	7.0000
38.3250	6.0000
38.3550	7.0000
38.3850	6.0000
38.4150	7.0000
38.4450	4.0000
38.4750	6.0000
38.5050	7.0000
38.5350	6.0000
38.5650	9.0000
38.5950	3.0000
38.6250	10.0000

38.6550	4.0000
38.6850	6.0000
38.7150	2.0000
38.7450	3.0000
38.7750	8.0000
38.8050	14.0000
38.8350	5.0000
38.8650	7.0000
38.8950	6.0000
38.9250	2.0000
38.9550	2.0000
38.9850	2.0000
39.0150	5.0000
39.0450	4.0000
39.0750	3.0000
39.1050	4.0000
39.1350	3.0000
39.1650	8.0000
39.1950	8.0000
39.2250	4.0000
39.2550	4.0000
39.2850	7.0000
39.3150	2.0000
39.3450	9.0000
39.3750	3.0000
39.4050	8.0000

39.4350	4.0000
39.4650	7.0000
39.4950	5.0000
39.5250	7.0000
39.5550	6.0000
39.5850	7.0000
39.6150	5.0000
39.6450	4.0000
39.6750	6.0000
39.7050	10.0000
39.7350	5.0000
39.7650	3.0000
39.7950	6.0000
39.8250	3.0000
39.8550	5.0000
39.8850	5.0000
39.9150	3.0000
39.9450	7.0000
39.9750	5.0000
40.0050	5.0000
40.0350	4.0000
40.0650	8.0000
40.0950	3.0000
40.1250	9.0000
40.1550	5.0000
40.1850	4.0000

40.2150	6.0000
40.2450	6.0000
40.2750	6.0000
40.3050	6.0000
40.3350	5.0000
40.3650	11.0000
40.3950	4.0000
40.4250	5.0000
40.4550	5.0000
40.4850	1.0000
40.5150	7.0000
40.5450	6.0000
40.5750	8.0000
40.6050	8.0000
40.6350	7.0000
40.6650	5.0000
40.6950	10.0000
40.7250	9.0000
40.7550	4.0000
40.7850	4.0000
40.8150	2.0000
40.8450	7.0000
40.8750	3.0000
40.9050	3.0000
40.9350	1.0000
40.9650	2.0000

40.9950	2.0000
41.0250	2.0000
41.0550	2.0000
41.0850	4.0000
41.1150	1.0000
41.1450	5.0000
41.1750	4.0000
41.2050	4.0000
41.2350	7.0000
41.2650	5.0000
41.2950	2.0000
41.3250	6.0000
41.3550	3.0000
41.3850	3.0000
41.4150	4.0000
41.4450	4.0000
41.4750	5.0000
41.5050	5.0000
41.5350	3.0000
41.5650	1.0000
41.5950	3.0000
41.6250	0.0000
41.6550	2.0000
41.6850	5.0000
41.7150	4.0000
41.7450	4.0000

41.7750	2.0000
41.8050	7.0000
41.8350	9.0000
41.8650	2.0000
41.8950	1.0000
41.9250	2.0000
41.9550	3.0000
41.9850	4.0000
42.0150	3.0000
42.0450	0.0000
42.0750	6.0000
42.1050	2.0000
42.1350	2.0000
42.1650	4.0000
42.1950	3.0000
42.2250	4.0000
42.2550	5.0000
42.2850	5.0000
42.3150	3.0000
42.3450	3.0000
42.3750	0.0000
42.4050	6.0000
42.4350	3.0000
42.4650	2.0000
42.4950	2.0000
42.5250	4.0000

42.5550	2.0000
42.5850	5.0000
42.6150	3.0000
42.6450	7.0000
42.6750	3.0000
42.7050	2.0000
42.7350	5.0000
42.7650	4.0000
42.7950	7.0000
42.8250	5.0000
42.8550	5.0000
42.8850	3.0000
42.9150	3.0000
42.9450	1.0000
42.9750	2.0000
43.0050	7.0000
43.0350	2.0000
43.0650	1.0000
43.0950	4.0000
43.1250	4.0000
43.1550	1.0000
43.1850	1.0000
43.2150	3.0000
43.2450	2.0000
43.2750	3.0000
43.3050	0.0000

43.3350	4.0000
43.3650	3.0000
43.3950	4.0000
43.4250	4.0000
43.4550	1.0000
43.4850	3.0000
43.5150	2.0000
43.5450	9.0000
43.5750	6.0000
43.6050	5.0000
43.6350	6.0000
43.6650	2.0000
43.6950	5.0000
43.7250	1.0000
43.7550	2.0000
43.7850	3.0000
43.8150	4.0000
43.8450	6.0000
43.8750	3.0000
43.9050	1.0000
43.9350	3.0000
43.9650	4.0000
43.9950	6.0000
44.0250	4.0000
44.0550	4.0000
44.0850	1.0000

44.1150	0.0000
44.1450	7.0000
44.1750	3.0000
44.2050	2.0000
44.2350	3.0000
44.2650	4.0000
44.2950	4.0000
44.3250	2.0000
44.3550	3.0000
44.3850	4.0000
44.4150	4.0000
44.4450	5.0000
44.4750	1.0000
44.5050	0.0000
44.5350	7.0000
44.5650	6.0000
44.5950	2.0000
44.6250	4.0000
44.6550	5.0000
44.6850	5.0000
44.7150	3.0000
44.7450	3.0000
44.7750	4.0000
44.8050	6.0000
44.8350	6.0000
44.8650	2.0000

44.8950	5.0000
44.9250	3.0000
44.9550	7.0000
44.9850	6.0000
45.0150	1.0000
45.0450	3.0000
45.0750	4.0000
45.1050	4.0000
45.1350	8.0000
45.1650	8.0000
45.1950	3.0000
45.2250	2.0000
45.2550	8.0000
45.2850	4.0000
45.3150	2.0000
45.3450	4.0000
45.3750	4.0000
45.4050	5.0000
45.4350	8.0000
45.4650	4.0000
45.4950	7.0000
45.5250	6.0000
45.5550	3.0000
45.5850	7.0000
45.6150	5.0000
45.6450	1.0000

45.6750	3.0000
45.7050	4.0000
45.7350	5.0000
45.7650	3.0000
45.7950	4.0000
45.8250	2.0000
45.8550	2.0000
45.8850	3.0000
45.9150	6.0000
45.9450	2.0000
45.9750	5.0000
46.0050	9.0000
46.0350	3.0000
46.0650	5.0000
46.0950	2.0000
46.1250	1.0000
46.1550	2.0000
46.1850	6.0000
46.2150	6.0000
46.2450	5.0000
46.2750	1.0000
46.3050	3.0000
46.3350	7.0000
46.3650	6.0000
46.3950	3.0000
46.4250	0.0000

46.4550	5.0000
46.4850	4.0000
46.5150	4.0000
46.5450	4.0000
46.5750	4.0000
46.6050	2.0000
46.6350	5.0000
46.6650	2.0000
46.6950	4.0000
46.7250	4.0000
46.7550	6.0000
46.7850	6.0000
46.8150	1.0000
46.8450	3.0000
46.8750	1.0000
46.9050	0.0000
46.9350	2.0000
46.9650	3.0000
46.9950	8.0000
47.0250	5.0000
47.0550	1.0000
47.0850	9.0000
47.1150	2.0000
47.1450	6.0000
47.1750	3.0000
47.2050	5.0000

47.2350	3.0000
47.2650	5.0000
47.2950	4.0000
47.3250	5.0000
47.3550	3.0000
47.3850	3.0000
47.4150	6.0000
47.4450	6.0000
47.4750	1.0000
47.5050	4.0000
47.5350	7.0000
47.5650	9.0000
47.5950	6.0000
47.6250	5.0000
47.6550	2.0000
47.6850	0.0000
47.7150	9.0000
47.7450	5.0000
47.7750	2.0000
47.8050	5.0000
47.8350	3.0000
47.8650	2.0000
47.8950	4.0000
47.9250	3.0000
47.9550	1.0000
47.9850	3.0000

48.0150	3.0000
48.0450	4.0000
48.0750	2.0000
48.1050	3.0000
48.1350	4.0000
48.1650	2.0000
48.1950	4.0000
48.2250	2.0000
48.2550	2.0000
48.2850	3.0000
48.3150	3.0000
48.3450	7.0000
48.3750	3.0000
48.4050	5.0000
48.4350	7.0000
48.4650	5.0000
48.4950	3.0000
48.5250	3.0000
48.5550	1.0000
48.5850	4.0000
48.6150	6.0000
48.6450	0.0000
48.6750	4.0000
48.7050	3.0000
48.7350	1.0000
48.7650	3.0000

48.7950	0.0000
48.8250	4.0000
48.8550	5.0000
48.8850	3.0000
48.9150	2.0000
48.9450	2.0000
48.9750	3.0000
49.0050	2.0000
49.0350	5.0000
49.0650	3.0000
49.0950	4.0000
49.1250	3.0000
49.1550	0.0000
49.1850	3.0000
49.2150	1.0000
49.2450	6.0000
49.2750	4.0000
49.3050	2.0000
49.3350	3.0000
49.3650	3.0000
49.3950	3.0000
49.4250	5.0000
49.4550	4.0000
49.4850	1.0000
49.5150	3.0000
49.5450	3.0000

49.5750	3.0000
49.6050	1.0000
49.6350	1.0000
49.6650	2.0000
49.6950	7.0000
49.7250	1.0000
49.7550	2.0000
49.7850	2.0000
49.8150	2.0000
49.8450	1.0000
49.8750	2.0000
49.9050	3.0000
49.9350	4.0000
49.9650	8.0000
49.9950	3.0000
50.0250	1.0000
50.0550	1.0000
50.0850	5.0000
50.1150	2.0000
50.1450	4.0000
50.1750	1.0000
50.2050	4.0000
50.2350	5.0000
50.2650	1.0000
50.2950	3.0000
50.3250	2.0000

50.3550	3.0000
50.3850	4.0000
50.4150	4.0000
50.4450	4.0000
50.4750	6.0000
50.5050	4.0000
50.5350	2.0000
50.5650	4.0000
50.5950	2.0000
50.6250	2.0000
50.6550	1.0000
50.6850	1.0000
50.7150	2.0000
50.7450	7.0000
50.7750	2.0000
50.8050	1.0000
50.8350	3.0000
50.8650	4.0000
50.8950	3.0000
50.9250	4.0000
50.9550	2.0000
50.9850	0.0000
51.0150	2.0000
51.0450	2.0000
51.0750	3.0000
51.1050	3.0000

51.1350	1.0000
51.1650	5.0000
51.1950	3.0000
51.2250	0.0000
51.2550	4.0000
51.2850	2.0000
51.3150	7.0000
51.3450	2.0000
51.3750	2.0000
51.4050	2.0000
51.4350	4.0000
51.4650	1.0000
51.4950	3.0000
51.5250	2.0000
51.5550	0.0000
51.5850	4.0000
51.6150	5.0000
51.6450	4.0000
51.6750	5.0000
51.7050	5.0000
51.7350	2.0000
51.7650	2.0000
51.7950	2.0000
51.8250	4.0000
51.8550	4.0000
51.8850	5.0000

51.9150	3.0000
51.9450	2.0000
51.9750	3.0000
52.0050	5.0000
52.0350	4.0000
52.0650	4.0000
52.0950	4.0000
52.1250	2.0000
52.1550	3.0000
52.1850	5.0000
52.2150	7.0000
52.2450	1.0000
52.2750	3.0000
52.3050	3.0000
52.3350	1.0000
52.3650	1.0000
52.3950	3.0000
52.4250	0.0000
52.4550	1.0000
52.4850	3.0000
52.5150	2.0000
52.5450	5.0000
52.5750	5.0000
52.6050	4.0000
52.6350	3.0000
52.6650	2.0000

52.6950	2.0000
52.7250	3.0000
52.7550	1.0000
52.7850	3.0000
52.8150	2.0000
52.8450	1.0000
52.8750	4.0000
52.9050	2.0000
52.9350	3.0000
52.9650	4.0000
52.9950	1.0000
53.0250	2.0000
53.0550	5.0000
53.0850	3.0000
53.1150	6.0000
53.1450	2.0000
53.1750	4.0000
53.2050	1.0000
53.2350	4.0000
53.2650	2.0000
53.2950	5.0000
53.3250	5.0000
53.3550	3.0000
53.3850	4.0000
53.4150	1.0000
53.4450	1.0000

53.4750	7.0000
53.5050	3.0000
53.5350	2.0000
53.5650	4.0000
53.5950	1.0000
53.6250	1.0000
53.6550	4.0000
53.6850	5.0000
53.7150	4.0000
53.7450	4.0000
53.7750	5.0000
53.8050	0.0000
53.8350	2.0000
53.8650	3.0000
53.8950	3.0000
53.9250	3.0000
53.9550	3.0000
53.9850	7.0000
54.0150	1.0000
54.0450	3.0000
54.0750	4.0000
54.1050	3.0000
54.1350	2.0000
54.1650	3.0000
54.1950	7.0000
54.2250	1.0000

54.2550	4.0000
54.2850	4.0000
54.3150	4.0000
54.3450	3.0000
54.3750	0.0000
54.4050	6.0000
54.4350	5.0000
54.4650	4.0000
54.4950	4.0000
54.5250	1.0000
54.5550	3.0000
54.5850	5.0000
54.6150	0.0000
54.6450	2.0000
54.6750	6.0000
54.7050	3.0000
54.7350	4.0000
54.7650	5.0000
54.7950	5.0000
54.8250	4.0000
54.8550	2.0000
54.8850	5.0000
54.9150	2.0000
54.9450	3.0000
54.9750	7.0000
55.0050	3.0000

55.0350	2.0000
55.0650	7.0000
55.0950	5.0000
55.1250	4.0000
55.1550	5.0000
55.1850	9.0000
55.2150	5.0000
55.2450	2.0000
55.2750	1.0000
55.3050	6.0000
55.3350	5.0000
55.3650	6.0000
55.3950	5.0000
55.4250	7.0000
55.4550	4.0000
55.4850	8.0000
55.5150	6.0000
55.5450	2.0000
55.5750	5.0000
55.6050	5.0000
55.6350	5.0000
55.6650	4.0000
55.6950	3.0000
55.7250	7.0000
55.7550	7.0000
55.7850	3.0000

55.8150	3.0000
55.8450	6.0000
55.8750	6.0000
55.9050	5.0000
55.9350	4.0000
55.9650	4.0000
55.9950	3.0000
56.0250	4.0000
56.0550	3.0000
56.0850	3.0000
56.1150	3.0000
56.1450	3.0000
56.1750	8.0000
56.2050	5.0000
56.2350	5.0000
56.2650	9.0000
56.2950	4.0000
56.3250	0.0000
56.3550	4.0000
56.3850	8.0000
56.4150	3.0000
56.4450	7.0000
56.4750	6.0000
56.5050	5.0000
56.5350	6.0000
56.5650	6.0000

56.5950	3.0000
56.6250	6.0000
56.6550	9.0000
56.6850	8.0000
56.7150	3.0000
56.7450	7.0000
56.7750	5.0000
56.8050	5.0000
56.8350	0.0000
56.8650	2.0000
56.8950	4.0000
56.9250	2.0000
56.9550	7.0000
56.9850	3.0000
57.0150	2.0000
57.0450	7.0000
57.0750	2.0000
57.1050	4.0000
57.1350	6.0000
57.1650	5.0000
57.1950	5.0000
57.2250	4.0000
57.2550	3.0000
57.2850	7.0000
57.3150	7.0000
57.3450	2.0000

57.3750	6.0000
57.4050	3.0000
57.4350	1.0000
57.4650	5.0000
57.4950	3.0000
57.5250	6.0000
57.5550	1.0000
57.5850	3.0000
57.6150	5.0000
57.6450	3.0000
57.6750	1.0000
57.7050	1.0000
57.7350	3.0000
57.7650	2.0000
57.7950	2.0000
57.8250	5.0000
57.8550	5.0000
57.8850	7.0000
57.9150	0.0000
57.9450	5.0000
57.9750	3.0000
58.0050	6.0000
58.0350	5.0000
58.0650	2.0000
58.0950	1.0000
58.1250	8.0000

58.1550	4.0000
58.1850	3.0000
58.2150	3.0000
58.2450	5.0000
58.2750	4.0000
58.3050	4.0000
58.3350	3.0000
58.3650	1.0000
58.3950	1.0000
58.4250	7.0000
58.4550	4.0000
58.4850	3.0000
58.5150	6.0000
58.5450	1.0000
58.5750	5.0000
58.6050	4.0000
58.6350	3.0000
58.6650	2.0000
58.6950	5.0000
58.7250	2.0000
58.7550	1.0000
58.7850	2.0000
58.8150	8.0000
58.8450	2.0000
58.8750	7.0000
58.9050	1.0000

58.9350	1.0000
58.9650	3.0000
58.9950	1.0000
59.0250	7.0000
59.0550	6.0000
59.0850	5.0000
59.1150	5.0000
59.1450	1.0000
59.1750	8.0000
59.2050	3.0000
59.2350	4.0000
59.2650	2.0000
59.2950	7.0000
59.3250	8.0000
59.3550	2.0000
59.3850	4.0000
59.4150	9.0000
59.4450	6.0000
59.4750	5.0000
59.5050	6.0000
59.5350	4.0000
59.5650	3.0000
59.5950	5.0000
59.6250	3.0000
59.6550	5.0000
59.6850	5.0000

59.7150	3.0000
59.7450	7.0000
59.7750	10.0000
59.8050	1.0000
59.8350	5.0000
59.8650	4.0000
59.8950	4.0000
59.9250	5.0000
59.9550	7.0000
59.9850	8.0000

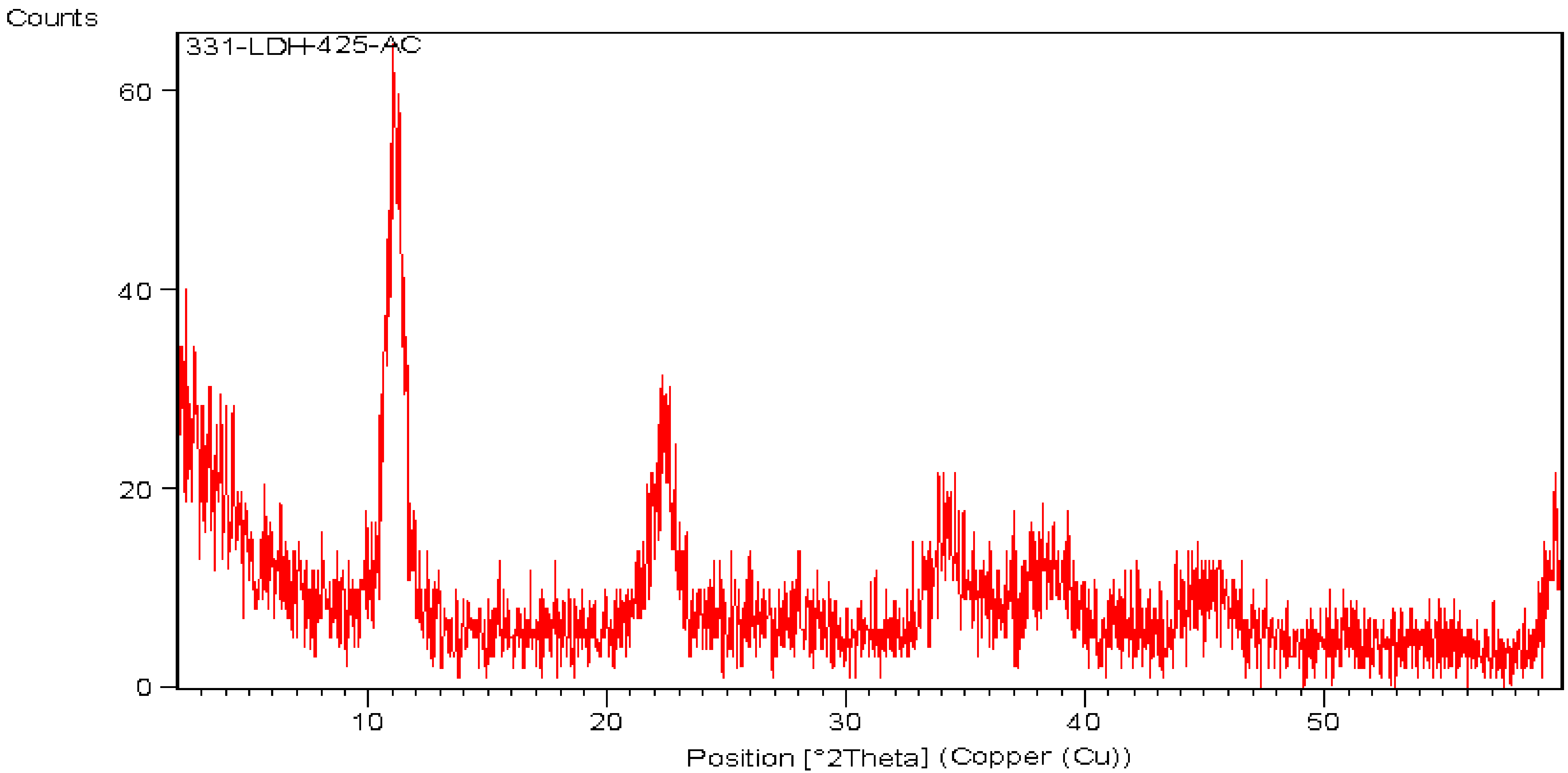
Counts
331-LDH-425-AC
60
40
20
0
10
20
30
40
50
Position [°2Theta] (Copper (Cu))

Pos. [°2Th.]	Iobs [cts]
2.0250	34.0000
2.0550	35.0000
2.0850	24.0000
2.1150	35.0000
2.1450	26.0000
2.1750	31.0000
2.2050	35.0000
2.2350	33.0000
2.2650	20.0000
2.2950	27.0000
2.3250	23.0000
2.3550	26.0000
2.3850	41.0000
2.4150	19.0000
2.4450	29.0000
2.4750	31.0000
2.5050	27.0000
2.5350	23.0000
2.5650	22.0000
2.5950	19.0000
2.6250	29.0000
2.6550	27.0000
2.6850	25.0000
2.7150	26.0000
2.7450	35.0000

2.775029.0000

2.805028.0000

2.835029.0000

2.865026.0000

2.895023.0000

2.925013.0000

2.955021.0000

2.985019.0000

3.015020.0000

3.045029.0000

3.075029.0000

3.105017.0000

3.135025.0000

3.165016.0000

3.195024.0000

3.225026.0000

3.255019.0000

3.285024.0000

3.315030.0000

3.345031.0000

3.375017.0000

3.405022.0000

3.435016.0000

3.465023.0000

3.495018.0000

3.525021.0000

3.555012.0000

3.585020.0000

3.615027.0000

3.645024.0000

3.675019.0000

3.705019.0000

3.735019.0000

3.765022.0000

3.795021.0000

3.825030.0000

3.855021.0000

3.885027.0000

3.915013.0000

3.945019.0000

3.975019.0000

4.005020.0000

4.035029.0000

4.065019.0000

4.095017.0000

4.125012.0000

4.155019.0000

4.185015.0000

4.215017.0000

4.245014.0000

4.275017.0000

4.305027.0000

4.335029.0000

4.365020.0000

4.395018.0000

4.425018.0000

4.455016.0000

4.485014.0000

4.515015.0000

4.545020.0000

4.575016.0000

4.605019.0000

4.635018.0000

4.665020.0000

4.695014.0000

4.725012.0000

4.75507.0000

4.785018.0000

4.815018.0000

4.845019.0000

4.875017.0000

4.905016.0000

4.935014.0000

4.965018.0000

4.995011.0000

5.025011.0000

5.055014.0000

5.085016.0000

5.115015.0000

5.145012.0000

5.175015.0000

5.205011.0000

5.235010.0000

5.265011.0000

5.29508.0000

5.325010.0000

5.35508.0000

5.385010.0000

5.41509.0000

5.445012.0000

5.475015.0000

5.505015.0000

5.535016.0000

5.56509.0000

5.595014.0000

5.62509.0000

5.655015.0000

5.685021.0000

5.715012.0000

5.745011.0000

5.77509.0000

5.80508.0000

5.835017.0000

5.865014.0000

5.895013.0000

5.925011.0000

5.955012.0000

5.985016.0000

6.015015.0000

6.04509.0000

6.07507.0000

6.105012.0000

6.135011.0000

6.165014.0000

6.195012.0000

6.225012.0000

6.255017.0000

6.285012.0000

6.31509.0000

6.345019.0000

6.375018.0000

6.40508.0000

6.435010.0000

6.465011.0000

6.495015.0000

6.525013.0000

6.55507.0000

6.585014.0000

6.61509.0000

6.645012.0000

6.675011.0000

6.70507.0000

6.735013.0000

6.76506.0000

6.79507.0000

6.82505.0000

6.855014.0000

6.885014.0000

6.915014.0000

6.94508.0000

6.97505.0000

7.00509.0000

7.035010.0000

7.065012.0000

7.095012.0000

7.125015.0000

7.155010.0000

7.185011.0000

7.215010.0000

7.24509.0000

7.275011.0000

7.305012.0000

7.33504.0000

7.365011.0000

7.395012.0000

7.425013.0000

7.45506.0000

7.485010.0000

7.51507.0000

7.545010.0000

7.57504.0000

7.605010.0000

7.63508.0000

7.66505.0000

7.695012.0000

7.72509.0000

7.75503.0000

7.78507.0000

7.815010.0000

7.84503.0000

7.875012.0000

7.90508.0000

7.93507.0000

7.96507.0000

7.995010.0000

8.02507.0000

8.055016.0000

8.085010.0000

8.115010.0000

8.145013.0000

8.175011.0000

8.20508.0000

8.23509.0000

8.26509.0000

8.29508.0000

8.32509.0000

8.355011.0000

8.38507.0000

8.41505.0000

8.44508.0000

8.47506.0000

8.50506.0000

8.53509.0000

8.565011.0000

8.59507.0000

8.625011.0000

8.65505.0000

8.685014.0000

8.71505.0000

8.74508.0000

8.775011.0000

8.80508.0000

8.83504.0000

8.86509.0000

8.895011.0000

8.925012.0000

8.955010.0000

8.985010.0000

9.01504.0000

9.04507.0000

9.07502.0000

9.10508.0000

9.13505.0000

9.165013.0000

9.19509.0000

9.22507.0000

9.25509.0000

9.285010.0000

9.31507.0000

9.345011.0000

9.37504.0000

9.405010.0000

9.435010.0000

9.46507.0000

9.49509.0000

9.525010.0000

9.555010.0000

9.58504.0000

9.61509.0000

9.64505.0000

9.675011.0000

9.705011.0000

9.735010.0000

9.765011.0000

9.795013.0000

9.825010.0000

9.855011.0000

9.88508.0000

9.915011.0000

9.945018.0000

9.975011.0000

10.0050	7.0000
10.0350	12.0000
10.0650	9.0000
10.0950	12.0000
10.1250	17.0000
10.1550	6.0000
10.1850	10.0000
10.2150	13.0000
10.2450	9.0000
10.2750	16.0000
10.3050	11.0000
10.3350	17.0000
10.3650	13.0000
10.3950	15.0000
10.4250	9.0000
10.4550	21.0000
10.4850	30.0000
10.5150	29.0000
10.5450	17.0000

10.5750	29.0000
10.6050	28.0000
10.6350	23.0000
10.6650	34.0000
10.6950	36.0000
10.7250	38.0000
10.7550	33.0000
10.7850	46.0000
10.8150	40.0000
10.8450	38.0000
10.8750	50.0000
10.9050	40.0000
10.9350	48.0000
10.9650	56.0000
10.9950	48.0000
11.0250	66.0000
11.0550	61.0000
11.0850	59.0000
11.1150	63.0000
11.1450	54.0000
11.1750	53.0000
11.2050	49.0000
11.2350	61.0000
11.2650	59.0000
11.2950	59.0000
11.3250	55.0000

11.3550	59.0000
11.3850	45.0000
11.4150	43.0000
11.4450	35.0000
11.4750	42.0000
11.5050	30.0000
11.5350	36.0000
11.5650	35.0000
11.5950	30.0000
11.6250	33.0000
11.6550	24.0000
11.6850	11.0000
11.7150	13.0000
11.7450	11.0000
11.7750	19.0000
11.8050	12.0000
11.8350	12.0000
11.8650	12.0000
11.8950	18.0000
11.9250	15.0000
11.9550	17.0000
11.9850	17.0000
12.0150	7.0000
12.0450	10.0000
12.0750	8.0000
12.1050	7.0000

12.1350	14.0000
12.1650	7.0000
12.1950	6.0000
12.2250	10.0000
12.2550	5.0000
12.2850	5.0000
12.3150	8.0000
12.3450	10.0000
12.3750	9.0000
12.4050	10.0000
12.4350	10.0000
12.4650	4.0000
12.4950	14.0000
12.5250	5.0000
12.5550	11.0000
12.5850	4.0000
12.6150	8.0000
12.6450	2.0000
12.6750	7.0000
12.7050	10.0000
12.7350	5.0000
12.7650	3.0000
12.7950	6.0000
12.8250	11.0000
12.8550	8.0000
12.8850	7.0000

12.9150	10.0000
12.9450	12.0000
12.9750	9.0000
13.0050	2.0000
13.0350	2.0000
13.0650	2.0000
13.0950	2.0000
13.1250	5.0000
13.1550	6.0000
13.1850	8.0000
13.2150	7.0000
13.2450	7.0000
13.2750	6.0000
13.3050	7.0000
13.3350	6.0000
13.3650	3.0000
13.3950	3.0000
13.4250	4.0000
13.4550	7.0000
13.4850	7.0000
13.5150	7.0000
13.5450	4.0000
13.5750	3.0000
13.6050	3.0000
13.6350	4.0000
13.6650	10.0000

13.6950	7.0000
13.7250	9.0000
13.7550	8.0000
13.7850	1.0000
13.8150	4.0000
13.8450	5.0000
13.8750	3.0000
13.9050	4.0000
13.9350	5.0000
13.9650	9.0000
13.9950	8.0000
14.0250	5.0000
14.0550	4.0000
14.0850	4.0000
14.1150	4.0000
14.1450	5.0000
14.1750	9.0000
14.2050	6.0000
14.2350	7.0000
14.2650	6.0000
14.2950	7.0000
14.3250	6.0000
14.3550	7.0000
14.3850	5.0000
14.4150	5.0000
14.4450	6.0000

14.4750	7.0000
14.5050	6.0000
14.5350	5.0000
14.5650	7.0000
14.5950	2.0000
14.6250	8.0000
14.6550	7.0000
14.6850	8.0000
14.7150	8.0000
14.7450	4.0000
14.7750	5.0000
14.8050	5.0000
14.8350	7.0000
14.8650	6.0000
14.8950	2.0000
14.9250	4.0000
14.9550	1.0000
14.9850	5.0000
15.0150	8.0000
15.0450	9.0000
15.0750	3.0000
15.1050	7.0000
15.1350	5.0000
15.1650	7.0000
15.1950	3.0000
15.2250	8.0000

15.2550	3.0000
15.2850	8.0000
15.3150	8.0000
15.3450	5.0000
15.3750	5.0000
15.4050	8.0000
15.4350	7.0000
15.4650	13.0000
15.4950	6.0000
15.5250	5.0000
15.5550	4.0000
15.5850	6.0000
15.6150	3.0000
15.6450	10.0000
15.6750	6.0000
15.7050	5.0000
15.7350	5.0000
15.7650	7.0000
15.7950	4.0000
15.8250	9.0000
15.8550	4.0000
15.8850	6.0000
15.9150	8.0000
15.9450	5.0000
15.9750	5.0000
16.0050	5.0000

16.0350	5.0000
16.0650	2.0000
16.0950	5.0000
16.1250	6.0000
16.1550	3.0000
16.1850	7.0000
16.2150	8.0000
16.2450	5.0000
16.2750	5.0000
16.3050	4.0000
16.3350	8.0000
16.3650	5.0000
16.3950	6.0000
16.4250	6.0000
16.4550	5.0000
16.4850	2.0000
16.5150	2.0000
16.5450	8.0000
16.5750	6.0000
16.6050	4.0000
16.6350	7.0000
16.6650	5.0000
16.6950	4.0000
16.7250	7.0000
16.7550	6.0000
16.7850	12.0000

16.8150	6.0000
16.8450	4.0000
16.8750	6.0000
16.9050	5.0000
16.9350	6.0000
16.9650	5.0000
16.9950	6.0000
17.0250	2.0000
17.0550	7.0000
17.0850	6.0000
17.1150	6.0000
17.1450	3.0000
17.1750	2.0000
17.2050	9.0000
17.2350	1.0000
17.2650	10.0000
17.2950	9.0000
17.3250	6.0000
17.3550	3.0000
17.3850	5.0000
17.4150	6.0000
17.4450	8.0000
17.4750	7.0000
17.5050	5.0000
17.5350	8.0000
17.5650	7.0000

17.5950	5.0000
17.6250	3.0000
17.6550	7.0000
17.6850	6.0000
17.7150	6.0000
17.7450	7.0000
17.7750	8.0000
17.8050	13.0000
17.8350	7.0000
17.8650	3.0000
17.8950	1.0000
17.9250	9.0000
17.9550	7.0000
17.9850	5.0000
18.0150	5.0000
18.0450	5.0000
18.0750	2.0000
18.1050	7.0000
18.1350	4.0000
18.1650	9.0000
18.1950	8.0000
18.2250	7.0000
18.2550	3.0000
18.2850	3.0000
18.3150	5.0000
18.3450	5.0000

18.3750	10.0000
18.4050	9.0000
18.4350	5.0000
18.4650	3.0000
18.4950	2.0000
18.5250	4.0000
18.5550	4.0000
18.5850	4.0000
18.6150	1.0000
18.6450	9.0000
18.6750	8.0000
18.7050	5.0000
18.7350	5.0000
18.7650	3.0000
18.7950	6.0000
18.8250	7.0000
18.8550	5.0000
18.8850	7.0000
18.9150	10.0000
18.9450	4.0000
18.9750	6.0000
19.0050	5.0000
19.0350	8.0000
19.0650	5.0000
19.0950	4.0000
19.1250	5.0000

19.1550	7.0000
19.1850	7.0000
19.2150	2.0000
19.2450	5.0000
19.2750	9.0000
19.3050	5.0000
19.3350	7.0000
19.3650	8.0000
19.3950	5.0000
19.4250	6.0000
19.4550	9.0000
19.4850	4.0000
19.5150	7.0000
19.5450	4.0000
19.5750	5.0000
19.6050	4.0000
19.6350	3.0000
19.6650	3.0000
19.6950	6.0000
19.7250	3.0000
19.7550	6.0000
19.7850	6.0000
19.8150	5.0000
19.8450	8.0000
19.8750	5.0000
19.9050	7.0000

19.9350	10.0000
19.9650	6.0000
19.9950	7.0000
20.0250	5.0000
20.0550	3.0000
20.0850	3.0000
20.1150	7.0000
20.1450	6.0000
20.1750	4.0000
20.2050	2.0000
20.2350	3.0000
20.2650	6.0000
20.2950	2.0000
20.3250	9.0000
20.3550	4.0000
20.3850	10.0000
20.4150	5.0000
20.4450	5.0000
20.4750	8.0000
20.5050	8.0000
20.5350	4.0000
20.5650	10.0000
20.5950	5.0000
20.6250	4.0000
20.6550	9.0000
20.6850	8.0000

20.7150	7.0000
20.7450	6.0000
20.7750	3.0000
20.8050	9.0000
20.8350	10.0000
20.8650	6.0000
20.8950	5.0000
20.9250	9.0000
20.9550	4.0000
20.9850	7.0000
21.0150	9.0000
21.0450	10.0000
21.0750	7.0000
21.1050	8.0000
21.1350	9.0000
21.1650	12.0000
21.1950	9.0000
21.2250	14.0000
21.2550	11.0000
21.2850	8.0000
21.3150	7.0000
21.3450	9.0000
21.3750	14.0000
21.4050	7.0000
21.4350	8.0000
21.4650	12.0000

21.4950	15.0000
21.5250	8.0000
21.5550	8.0000
21.5850	11.0000
21.6150	13.0000
21.6450	14.0000
21.6750	16.0000
21.7050	21.0000
21.7350	9.0000
21.7650	10.0000
21.7950	11.0000
21.8250	22.0000
21.8550	21.0000
21.8850	22.0000
21.9150	20.0000
21.9450	18.0000
21.9750	13.0000
22.0050	16.0000
22.0350	21.0000
22.0650	18.0000
22.0950	22.0000
22.1250	27.0000
22.1550	15.0000
22.1850	17.0000
22.2150	28.0000
22.2450	32.0000

22.2750	28.0000
22.3050	22.0000
22.3350	30.0000
22.3650	30.0000
22.3950	26.0000
22.4250	24.0000
22.4550	30.0000
22.4850	21.0000
22.5150	29.0000
22.5450	21.0000
22.5750	31.0000
22.6050	25.0000
22.6350	21.0000
22.6650	17.0000
22.6950	19.0000
22.7250	16.0000
22.7550	14.0000
22.7850	15.0000
22.8150	19.0000
22.8450	25.0000
22.8750	12.0000
22.9050	14.0000
22.9350	12.0000
22.9650	9.0000
22.9950	17.0000
23.0250	12.0000

23.0550	14.0000
23.0850	10.0000
23.1150	11.0000
23.1450	14.0000
23.1750	6.0000
23.2050	14.0000
23.2350	6.0000
23.2650	15.0000
23.2950	16.0000
23.3250	9.0000
23.3550	10.0000
23.3850	6.0000
23.4150	7.0000
23.4450	5.0000
23.4750	3.0000
23.5050	9.0000
23.5350	6.0000
23.5650	5.0000
23.5950	10.0000
23.6250	5.0000
23.6550	5.0000
23.6850	8.0000
23.7150	4.0000
23.7450	7.0000
23.7750	10.0000
23.8050	7.0000

23.8350	6.0000
23.8650	7.0000
23.8950	10.0000
23.9250	8.0000
23.9550	7.0000
23.9850	4.0000
24.0150	8.0000
24.0450	10.0000
24.0750	6.0000
24.1050	9.0000
24.1350	4.0000
24.1650	4.0000
24.1950	6.0000
24.2250	8.0000
24.2550	10.0000
24.2850	11.0000
24.3150	4.0000
24.3450	9.0000
24.3750	4.0000
24.4050	13.0000
24.4350	5.0000
24.4650	9.0000
24.4950	7.0000
24.5250	5.0000
24.5550	5.0000
24.5850	8.0000

24.6150	5.0000
24.6450	8.0000
24.6750	7.0000
24.7050	13.0000
24.7350	10.0000
24.7650	5.0000
24.7950	4.0000
24.8250	1.0000
24.8550	8.0000
24.8850	10.0000
24.9150	9.0000
24.9450	6.0000
24.9750	6.0000
25.0050	4.0000
25.0350	3.0000
25.0650	7.0000
25.0950	4.0000
25.1250	7.0000
25.1550	6.0000
25.1850	14.0000
25.2150	3.0000
25.2450	8.0000
25.2750	4.0000
25.3050	8.0000
25.3350	10.0000
25.3650	11.0000

25.3950	4.0000
25.4250	2.0000
25.4550	10.0000
25.4850	8.0000
25.5150	9.0000
25.5450	6.0000
25.5750	7.0000
25.6050	6.0000
25.6350	2.0000
25.6650	4.0000
25.6950	8.0000
25.7250	5.0000
25.7550	6.0000
25.7850	11.0000
25.8150	6.0000
25.8450	4.0000
25.8750	4.0000
25.9050	8.0000
25.9350	14.0000
25.9650	4.0000
25.9950	6.0000
26.0250	6.0000
26.0550	12.0000
26.0850	7.0000
26.1150	5.0000
26.1450	5.0000

26.1750	9.0000
26.2050	3.0000
26.2350	7.0000
26.2650	11.0000
26.2950	8.0000
26.3250	8.0000
26.3550	8.0000
26.3850	5.0000
26.4150	4.0000
26.4450	9.0000
26.4750	6.0000
26.5050	5.0000
26.5350	10.0000
26.5650	5.0000
26.5950	7.0000
26.6250	9.0000
26.6550	5.0000
26.6850	7.0000
26.7150	7.0000
26.7450	7.0000
26.7750	10.0000
26.8050	9.0000
26.8350	7.0000
26.8650	3.0000
26.8950	1.0000
26.9250	7.0000

26.9550	3.0000
26.9850	7.0000
27.0150	8.0000
27.0450	4.0000
27.0750	4.0000
27.1050	6.0000
27.1350	3.0000
27.1650	10.0000
27.1950	6.0000
27.2250	8.0000
27.2550	5.0000
27.2850	4.0000
27.3150	6.0000
27.3450	9.0000
27.3750	6.0000
27.4050	9.0000
27.4350	4.0000
27.4650	11.0000
27.4950	6.0000
27.5250	8.0000
27.5550	6.0000
27.5850	5.0000
27.6150	5.0000
27.6450	4.0000
27.6750	11.0000
27.7050	8.0000

27.7350	4.0000
27.7650	8.0000
27.7950	6.0000
27.8250	10.0000
27.8550	3.0000
27.8850	11.0000
27.9150	8.0000
27.9450	9.0000
27.9750	14.0000
28.0050	6.0000
28.0350	14.0000
28.0650	7.0000
28.0950	6.0000
28.1250	6.0000
28.1550	6.0000
28.1850	6.0000
28.2150	6.0000
28.2450	4.0000
28.2750	6.0000
28.3050	8.0000
28.3350	10.0000
28.3650	9.0000
28.3950	8.0000
28.4250	9.0000
28.4550	8.0000
28.4850	6.0000

28.5150	3.0000
28.5450	9.0000
28.5750	7.0000
28.6050	2.0000
28.6350	10.0000
28.6650	4.0000
28.6950	8.0000
28.7250	7.0000
28.7550	12.0000
28.7850	11.0000
28.8150	8.0000
28.8450	9.0000
28.8750	6.0000
28.9050	9.0000
28.9350	4.0000
28.9650	5.0000
28.9950	4.0000
29.0250	9.0000
29.0550	5.0000
29.0850	9.0000
29.1150	5.0000
29.1450	5.0000
29.1750	3.0000
29.2050	4.0000
29.2350	6.0000
29.2650	8.0000

29.2950	11.0000
29.3250	5.0000
29.3550	3.0000
29.3850	2.0000
29.4150	4.0000
29.4450	9.0000
29.4750	6.0000
29.5050	4.0000
29.5350	2.0000
29.5650	9.0000
29.5950	5.0000
29.6250	7.0000
29.6550	4.0000
29.6850	5.0000
29.7150	7.0000
29.7450	3.0000
29.7750	4.0000
29.8050	5.0000
29.8350	5.0000
29.8650	4.0000
29.8950	4.0000
29.9250	4.0000
29.9550	2.0000
29.9850	6.0000
30.0150	8.0000
30.0450	5.0000

30.0750	8.0000
30.1050	1.0000
30.1350	5.0000
30.1650	4.0000
30.1950	5.0000
30.2250	7.0000
30.2550	4.0000
30.2850	5.0000
30.3150	5.0000
30.3450	6.0000
30.3750	5.0000
30.4050	1.0000
30.4350	8.0000
30.4650	6.0000
30.4950	8.0000
30.5250	6.0000
30.5550	6.0000
30.5850	4.0000
30.6150	3.0000
30.6450	9.0000
30.6750	5.0000
30.7050	7.0000
30.7350	4.0000
30.7650	5.0000
30.7950	7.0000
30.8250	6.0000

30.8550	6.0000
30.8850	6.0000
30.9150	6.0000
30.9450	3.0000
30.9750	6.0000
31.0050	5.0000
31.0350	11.0000
31.0650	5.0000
31.0950	4.0000
31.1250	6.0000
31.1550	3.0000
31.1850	10.0000
31.2150	12.0000
31.2450	7.0000
31.2750	4.0000
31.3050	3.0000
31.3350	6.0000
31.3650	5.0000
31.3950	4.0000
31.4250	1.0000
31.4550	6.0000
31.4850	6.0000
31.5150	4.0000
31.5450	5.0000
31.5750	7.0000
31.6050	3.0000

31.6350	7.0000
31.6650	10.0000
31.6950	10.0000
31.7250	5.0000
31.7550	5.0000
31.7850	7.0000
31.8150	7.0000
31.8450	4.0000
31.8750	7.0000
31.9050	5.0000
31.9350	3.0000
31.9650	9.0000
31.9950	5.0000
32.0250	3.0000
32.0550	6.0000
32.0850	9.0000
32.1150	7.0000
32.1450	5.0000
32.1750	7.0000
32.2050	4.0000
32.2350	8.0000
32.2650	3.0000
32.2950	5.0000
32.3250	5.0000
32.3550	7.0000
32.3850	7.0000

32.4150	4.0000
32.4450	5.0000
32.4750	6.0000
32.5050	5.0000
32.5350	3.0000
32.5650	10.0000
32.5950	4.0000
32.6250	3.0000
32.6550	5.0000
32.6850	9.0000
32.7150	7.0000
32.7450	15.0000
32.7750	4.0000
32.8050	10.0000
32.8350	8.0000
32.8650	6.0000
32.8950	4.0000
32.9250	6.0000
32.9550	5.0000
32.9850	7.0000
33.0150	10.0000
33.0450	6.0000
33.0750	9.0000
33.1050	7.0000
33.1350	7.0000
33.1650	9.0000

33.1950	9.0000
33.2250	15.0000
33.2550	10.0000
33.2850	10.0000
33.3150	13.0000
33.3450	10.0000
33.3750	14.0000
33.4050	10.0000
33.4350	4.0000
33.4650	5.0000
33.4950	12.0000
33.5250	4.0000
33.5550	15.0000
33.5850	7.0000
33.6150	10.0000
33.6450	11.0000
33.6750	11.0000
33.7050	13.0000
33.7350	14.0000
33.7650	13.0000
33.7950	7.0000
33.8250	12.0000
33.8550	16.0000
33.8850	22.0000
33.9150	17.0000
33.9450	16.0000

33.9750	12.0000
34.0050	14.0000
34.0350	14.0000
34.0650	22.0000
34.0950	11.0000
34.1250	16.0000
34.1550	16.0000
34.1850	19.0000
34.2150	19.0000
34.2450	20.0000
34.2750	16.0000
34.3050	13.0000
34.3350	19.0000
34.3650	20.0000
34.3950	14.0000
34.4250	13.0000
34.4550	11.0000
34.4850	13.0000
34.5150	13.0000
34.5450	18.0000
34.5750	22.0000
34.6050	14.0000
34.6350	12.0000
34.6650	11.0000
34.6950	18.0000
34.7250	10.0000

34.7550	9.0000
34.7850	6.0000
34.8150	10.0000
34.8450	10.0000
34.8750	18.0000
34.9050	12.0000
34.9350	18.0000
34.9650	9.0000
34.9950	11.0000
35.0250	9.0000
35.0550	11.0000
35.0850	10.0000
35.1150	12.0000
35.1450	9.0000
35.1750	11.0000
35.2050	10.0000
35.2350	14.0000
35.2650	8.0000
35.2950	9.0000
35.3250	6.0000
35.3550	16.0000
35.3850	10.0000
35.4150	9.0000
35.4450	12.0000
35.4750	8.0000
35.5050	3.0000

35.5350	11.0000
35.5650	10.0000
35.5950	11.0000
35.6250	12.0000
35.6550	5.0000
35.6850	12.0000
35.7150	8.0000
35.7450	13.0000
35.7750	12.0000
35.8050	11.0000
35.8350	8.0000
35.8650	7.0000
35.8950	10.0000
35.9250	12.0000
35.9550	6.0000
35.9850	4.0000
36.0150	9.0000
36.0450	15.0000
36.0750	8.0000
36.1050	11.0000
36.1350	4.0000
36.1650	10.0000
36.1950	5.0000
36.2250	9.0000
36.2550	9.0000
36.2850	5.0000

36.3150	6.0000
36.3450	12.0000
36.3750	7.0000
36.4050	9.0000
36.4350	9.0000
36.4650	6.0000
36.4950	9.0000
36.5250	8.0000
36.5550	8.0000
36.5850	6.0000
36.6150	5.0000
36.6450	11.0000
36.6750	5.0000
36.7050	14.0000
36.7350	8.0000
36.7650	10.0000
36.7950	9.0000
36.8250	8.0000
36.8550	10.0000
36.8850	13.0000
36.9150	7.0000
36.9450	7.0000
36.9750	18.0000
37.0050	5.0000
37.0350	9.0000
37.0650	2.0000

37.0950	12.0000
37.1250	14.0000
37.1550	9.0000
37.1850	9.0000
37.2150	2.0000
37.2450	6.0000
37.2750	9.0000
37.3050	10.0000
37.3350	5.0000
37.3650	13.0000
37.3950	8.0000
37.4250	12.0000
37.4550	7.0000
37.4850	5.0000
37.5150	11.0000
37.5450	13.0000
37.5750	10.0000
37.6050	7.0000
37.6350	14.0000
37.6650	11.0000
37.6950	17.0000
37.7250	11.0000
37.7550	16.0000
37.7850	14.0000
37.8150	16.0000
37.8450	8.0000

37.8750	8.0000
37.9050	15.0000
37.9350	8.0000
37.9650	6.0000
37.9950	13.0000
38.0250	9.0000
38.0550	16.0000
38.0850	7.0000
38.1150	15.0000
38.1450	15.0000
38.1750	14.0000
38.2050	14.0000
38.2350	19.0000
38.2650	12.0000
38.2950	13.0000
38.3250	12.0000
38.3550	14.0000
38.3850	8.0000
38.4150	16.0000
38.4450	15.0000
38.4750	14.0000
38.5050	11.0000
38.5350	9.0000
38.5650	11.0000
38.5950	13.0000
38.6250	9.0000

38.6550	16.0000
38.6850	17.0000
38.7150	15.0000
38.7450	11.0000
38.7750	13.0000
38.8050	9.0000
38.8350	10.0000
38.8650	14.0000
38.8950	11.0000
38.9250	9.0000
38.9550	10.0000
38.9850	10.0000
39.0150	12.0000
39.0450	13.0000
39.0750	12.0000
39.1050	14.0000
39.1350	8.0000
39.1650	13.0000
39.1950	9.0000
39.2250	15.0000
39.2550	7.0000
39.2850	9.0000
39.3150	18.0000
39.3450	10.0000
39.3750	12.0000
39.4050	9.0000

39.4350	6.0000
39.4650	9.0000
39.4950	8.0000
39.5250	13.0000
39.5550	5.0000
39.5850	11.0000
39.6150	11.0000
39.6450	10.0000
39.6750	6.0000
39.7050	8.0000
39.7350	6.0000
39.7650	7.0000
39.7950	10.0000
39.8250	8.0000
39.8550	11.0000
39.8850	7.0000
39.9150	7.0000
39.9450	4.0000
39.9750	11.0000
40.0050	7.0000
40.0350	9.0000
40.0650	4.0000
40.0950	8.0000
40.1250	2.0000
40.1550	10.0000
40.1850	5.0000

40.2150	6.0000
40.2450	8.0000
40.2750	7.0000
40.3050	5.0000
40.3350	5.0000
40.3650	9.0000
40.3950	5.0000
40.4250	11.0000
40.4550	9.0000
40.4850	5.0000
40.5150	2.0000
40.5450	2.0000
40.5750	5.0000
40.6050	5.0000
40.6350	4.0000
40.6650	2.0000
40.6950	4.0000
40.7250	7.0000
40.7550	6.0000
40.7850	5.0000
40.8150	7.0000
40.8450	5.0000
40.8750	1.0000
40.9050	6.0000
40.9350	9.0000
40.9650	5.0000

40.9950	4.0000
41.0250	6.0000
41.0550	10.0000
41.0850	8.0000
41.1150	4.0000
41.1450	5.0000
41.1750	8.0000
41.2050	11.0000
41.2350	5.0000
41.2650	5.0000
41.2950	9.0000
41.3250	7.0000
41.3550	6.0000
41.3850	4.0000
41.4150	4.0000
41.4450	6.0000
41.4750	10.0000
41.5050	8.0000
41.5350	2.0000
41.5650	5.0000
41.5950	9.0000
41.6250	5.0000
41.6550	12.0000
41.6850	8.0000
41.7150	6.0000
41.7450	5.0000

41.7750	7.0000
41.8050	5.0000
41.8350	7.0000
41.8650	3.0000
41.8950	12.0000
41.9250	11.0000
41.9550	2.0000
41.9850	8.0000
42.0150	3.0000
42.0450	6.0000
42.0750	5.0000
42.1050	4.0000
42.1350	5.0000
42.1650	3.0000
42.1950	13.0000
42.2250	10.0000
42.2550	4.0000
42.2850	10.0000
42.3150	5.0000
42.3450	8.0000
42.3750	3.0000
42.4050	2.0000
42.4350	5.0000
42.4650	4.0000
42.4950	6.0000
42.5250	4.0000

42.5550	8.0000
42.5850	4.0000
42.6150	5.0000
42.6450	7.0000
42.6750	10.0000
42.7050	1.0000
42.7350	3.0000
42.7650	5.0000
42.7950	7.0000
42.8250	5.0000
42.8550	6.0000
42.8850	3.0000
42.9150	8.0000
42.9450	8.0000
42.9750	6.0000
43.0050	5.0000
43.0350	7.0000
43.0650	2.0000
43.0950	11.0000
43.1250	7.0000
43.1550	10.0000
43.1850	6.0000
43.2150	2.0000
43.2450	1.0000
43.2750	7.0000
43.3050	5.0000

43.3350	3.0000
43.3650	3.0000
43.3950	3.0000
43.4250	9.0000
43.4550	4.0000
43.4850	4.0000
43.5150	6.0000
43.5450	6.0000
43.5750	7.0000
43.6050	5.0000
43.6350	2.0000
43.6650	6.0000
43.6950	6.0000
43.7250	6.0000
43.7550	5.0000
43.7850	14.0000
43.8150	6.0000
43.8450	9.0000
43.8750	8.0000
43.9050	8.0000
43.9350	8.0000
43.9650	12.0000
43.9950	10.0000
44.0250	5.0000
44.0550	11.0000
44.0850	5.0000

44.1150	11.0000
44.1450	9.0000
44.1750	6.0000
44.2050	8.0000
44.2350	2.0000
44.2650	6.0000
44.2950	14.0000
44.3250	10.0000
44.3550	7.0000
44.3850	6.0000
44.4150	8.0000
44.4450	12.0000
44.4750	14.0000
44.5050	8.0000
44.5350	12.0000
44.5650	6.0000
44.5950	8.0000
44.6250	6.0000
44.6550	11.0000
44.6850	15.0000
44.7150	6.0000
44.7450	11.0000
44.7750	8.0000
44.8050	11.0000
44.8350	11.0000
44.8650	13.0000

44.8950	7.0000
44.9250	12.0000
44.9550	11.0000
44.9850	3.0000
45.0150	13.0000
45.0450	7.0000
45.0750	10.0000
45.1050	11.0000
45.1350	9.0000
45.1650	7.0000
45.1950	13.0000
45.2250	12.0000
45.2550	12.0000
45.2850	8.0000
45.3150	10.0000
45.3450	13.0000
45.3750	9.0000
45.4050	8.0000
45.4350	12.0000
45.4650	11.0000
45.4950	5.0000
45.5250	10.0000
45.5550	13.0000
45.5850	13.0000
45.6150	12.0000
45.6450	4.0000

45.6750	13.0000
45.7050	8.0000
45.7350	12.0000
45.7650	9.0000
45.7950	8.0000
45.8250	10.0000
45.8550	10.0000
45.8850	10.0000
45.9150	11.0000
45.9450	9.0000
45.9750	7.0000
46.0050	6.0000
46.0350	8.0000
46.0650	7.0000
46.0950	8.0000
46.1250	8.0000
46.1550	4.0000
46.1850	11.0000
46.2150	7.0000
46.2450	11.0000
46.2750	6.0000
46.3050	8.0000
46.3350	10.0000
46.3650	6.0000
46.3950	9.0000
46.4250	5.0000

46.4550	10.0000
46.4850	7.0000
46.5150	4.0000
46.5450	8.0000
46.5750	13.0000
46.6050	3.0000
46.6350	6.0000
46.6650	4.0000
46.6950	7.0000
46.7250	1.0000
46.7550	2.0000
46.7850	7.0000
46.8150	4.0000
46.8450	4.0000
46.8750	2.0000
46.9050	6.0000
46.9350	5.0000
46.9650	7.0000
46.9950	9.0000
47.0250	10.0000
47.0550	6.0000
47.0850	8.0000
47.1150	4.0000
47.1450	6.0000
47.1750	2.0000
47.2050	7.0000

47.2350	6.0000
47.2650	8.0000
47.2950	7.0000
47.3250	3.0000
47.3550	0.0000
47.3850	6.0000
47.4150	5.0000
47.4450	6.0000
47.4750	8.0000
47.5050	8.0000
47.5350	5.0000
47.5650	8.0000
47.5950	11.0000
47.6250	2.0000
47.6550	5.0000
47.6850	2.0000
47.7150	5.0000
47.7450	5.0000
47.7750	4.0000
47.8050	7.0000
47.8350	7.0000
47.8650	5.0000
47.8950	5.0000
47.9250	2.0000
47.9550	6.0000
47.9850	5.0000

48.0150	4.0000
48.0450	7.0000
48.0750	7.0000
48.1050	6.0000
48.1350	2.0000
48.1650	1.0000
48.1950	4.0000
48.2250	4.0000
48.2550	7.0000
48.2850	3.0000
48.3150	5.0000
48.3450	5.0000
48.3750	8.0000
48.4050	2.0000
48.4350	3.0000
48.4650	9.0000
48.4950	2.0000
48.5250	5.0000
48.5550	5.0000
48.5850	3.0000
48.6150	3.0000
48.6450	3.0000
48.6750	3.0000
48.7050	5.0000
48.7350	6.0000
48.7650	5.0000

48.7950	3.0000
48.8250	6.0000
48.8550	5.0000
48.8850	5.0000
48.9150	5.0000
48.9450	2.0000
48.9750	4.0000
49.0050	4.0000
49.0350	6.0000
49.0650	6.0000
49.0950	0.0000
49.1250	6.0000
49.1550	1.0000
49.1850	5.0000
49.2150	0.0000
49.2450	4.0000
49.2750	1.0000
49.3050	7.0000
49.3350	3.0000
49.3650	3.0000
49.3950	8.0000
49.4250	5.0000
49.4550	5.0000
49.4850	2.0000
49.5150	7.0000
49.5450	6.0000

49.5750	5.0000
49.6050	1.0000
49.6350	2.0000
49.6650	5.0000
49.6950	6.0000
49.7250	5.0000
49.7550	4.0000
49.7850	5.0000
49.8150	7.0000
49.8450	8.0000
49.8750	4.0000
49.9050	5.0000
49.9350	5.0000
49.9650	4.0000
49.9950	4.0000
50.0250	5.0000
50.0550	1.0000
50.0850	8.0000
50.1150	6.0000
50.1450	3.0000
50.1750	6.0000
50.2050	3.0000
50.2350	10.0000
50.2650	5.0000
50.2950	6.0000
50.3250	6.0000

50.3550	0.0000
50.3850	6.0000
50.4150	5.0000
50.4450	3.0000
50.4750	8.0000
50.5050	5.0000
50.5350	7.0000
50.5650	4.0000
50.5950	4.0000
50.6250	5.0000
50.6550	3.0000
50.6850	2.0000
50.7150	3.0000
50.7450	6.0000
50.7750	10.0000
50.8050	6.0000
50.8350	7.0000
50.8650	4.0000
50.8950	8.0000
50.9250	4.0000
50.9550	8.0000
50.9850	6.0000
51.0150	6.0000
51.0450	5.0000
51.0750	3.0000
51.1050	9.0000

51.1350	6.0000
51.1650	8.0000
51.1950	7.0000
51.2250	2.0000
51.2550	6.0000
51.2850	7.0000
51.3150	8.0000
51.3450	9.0000
51.3750	6.0000
51.4050	3.0000
51.4350	2.0000
51.4650	7.0000
51.4950	4.0000
51.5250	2.0000
51.5550	3.0000
51.5850	2.0000
51.6150	5.0000
51.6450	8.0000
51.6750	2.0000
51.7050	6.0000
51.7350	3.0000
51.7650	4.0000
51.7950	6.0000
51.8250	3.0000
51.8550	7.0000
51.8850	7.0000

51.9150	1.0000
51.9450	3.0000
51.9750	3.0000
52.0050	4.0000
52.0350	2.0000
52.0650	7.0000
52.0950	4.0000
52.1250	6.0000
52.1550	5.0000
52.1850	7.0000
52.2150	4.0000
52.2450	5.0000
52.2750	4.0000
52.3050	2.0000
52.3350	1.0000
52.3650	4.0000
52.3950	2.0000
52.4250	4.0000
52.4550	6.0000
52.4850	7.0000
52.5150	4.0000
52.5450	4.0000
52.5750	5.0000
52.6050	5.0000
52.6350	5.0000
52.6650	3.0000

52.6950	5.0000
52.7250	5.0000
52.7550	0.0000
52.7850	2.0000
52.8150	7.0000
52.8450	4.0000
52.8750	6.0000
52.9050	8.0000
52.9350	7.0000
52.9650	0.0000
52.9950	2.0000
53.0250	3.0000
53.0550	5.0000
53.0850	4.0000
53.1150	3.0000
53.1450	6.0000
53.1750	3.0000
53.2050	2.0000
53.2350	8.0000
53.2650	7.0000
53.2950	3.0000
53.3250	2.0000
53.3550	5.0000
53.3850	4.0000
53.4150	2.0000
53.4450	6.0000

53.4750	6.0000
53.5050	5.0000
53.5350	5.0000
53.5650	4.0000
53.5950	8.0000
53.6250	8.0000
53.6550	8.0000
53.6850	2.0000
53.7150	6.0000
53.7450	5.0000
53.7750	6.0000
53.8050	2.0000
53.8350	7.0000
53.8650	2.0000
53.8950	2.0000
53.9250	1.0000
53.9550	6.0000
53.9850	5.0000
54.0150	4.0000
54.0450	6.0000
54.0750	5.0000
54.1050	4.0000
54.1350	2.0000
54.1650	6.0000
54.1950	4.0000
54.2250	3.0000

54.2550	5.0000
54.2850	7.0000
54.3150	4.0000
54.3450	3.0000
54.3750	2.0000
54.4050	9.0000
54.4350	3.0000
54.4650	6.0000
54.4950	4.0000
54.5250	3.0000
54.5550	3.0000
54.5850	1.0000
54.6150	5.0000
54.6450	7.0000
54.6750	7.0000
54.7050	6.0000
54.7350	2.0000
54.7650	3.0000
54.7950	9.0000
54.8250	1.0000
54.8550	1.0000
54.8850	8.0000
54.9150	4.0000
54.9450	3.0000
54.9750	4.0000
55.0050	6.0000

55.0350	2.0000
55.0650	8.0000
55.0950	4.0000
55.1250	3.0000
55.1550	6.0000
55.1850	2.0000
55.2150	7.0000
55.2450	4.0000
55.2750	4.0000
55.3050	2.0000
55.3350	2.0000
55.3650	7.0000
55.3950	3.0000
55.4250	9.0000
55.4550	6.0000
55.4850	1.0000
55.5150	6.0000
55.5450	2.0000
55.5750	5.0000
55.6050	7.0000
55.6350	2.0000
55.6650	8.0000
55.6950	2.0000
55.7250	6.0000
55.7550	3.0000
55.7850	6.0000

55.8150	4.0000
55.8450	4.0000
55.8750	3.0000
55.9050	3.0000
55.9350	6.0000
55.9650	5.0000
55.9950	0.0000
56.0250	2.0000
56.0550	4.0000
56.0850	3.0000
56.1150	6.0000
56.1450	6.0000
56.1750	2.0000
56.2050	4.0000
56.2350	5.0000
56.2650	4.0000
56.2950	2.0000
56.3250	5.0000
56.3550	7.0000
56.3850	5.0000
56.4150	2.0000
56.4450	5.0000
56.4750	4.0000
56.5050	3.0000
56.5350	3.0000
56.5650	3.0000

56.5950	2.0000
56.6250	4.0000
56.6550	1.0000
56.6850	4.0000
56.7150	6.0000
56.7450	6.0000
56.7750	3.0000
56.8050	3.0000
56.8350	6.0000
56.8650	3.0000
56.8950	4.0000
56.9250	1.0000
56.9550	3.0000
56.9850	2.0000
57.0150	5.0000
57.0450	9.0000
57.0750	4.0000
57.1050	5.0000
57.1350	3.0000
57.1650	4.0000
57.1950	3.0000
57.2250	1.0000
57.2550	3.0000
57.2850	6.0000
57.3150	2.0000
57.3450	5.0000

57.3750	4.0000
57.4050	1.0000
57.4350	5.0000
57.4650	3.0000
57.4950	5.0000
57.5250	0.0000
57.5550	4.0000
57.5850	4.0000
57.6150	2.0000
57.6450	5.0000
57.6750	5.0000
57.7050	4.0000
57.7350	4.0000
57.7650	0.0000
57.7950	4.0000
57.8250	4.0000
57.8550	4.0000
57.8850	1.0000
57.9150	3.0000
57.9450	5.0000
57.9750	4.0000
58.0050	3.0000
58.0350	2.0000
58.0650	5.0000
58.0950	6.0000
58.1250	6.0000

58.1550	6.0000
58.1850	6.0000
58.2150	4.0000
58.2450	2.0000
58.2750	3.0000
58.3050	1.0000
58.3350	5.0000
58.3650	8.0000
58.3950	2.0000
58.4250	3.0000
58.4550	2.0000
58.4850	4.0000
58.5150	4.0000
58.5450	3.0000
58.5750	6.0000
58.6050	3.0000
58.6350	2.0000
58.6650	5.0000
58.6950	6.0000
58.7250	2.0000
58.7550	3.0000
58.7850	6.0000
58.8150	5.0000
58.8450	7.0000
58.8750	3.0000
58.9050	3.0000

58.9350	5.0000
58.9650	4.0000
58.9950	11.0000
59.0250	7.0000
59.0550	9.0000
59.0850	1.0000
59.1150	5.0000
59.1450	13.0000
59.1750	4.0000
59.2050	15.0000
59.2350	9.0000
59.2650	12.0000
59.2950	7.0000
59.3250	14.0000
59.3550	8.0000
59.3850	13.0000
59.4150	11.0000
59.4450	14.0000
59.4750	11.0000
59.5050	11.0000
59.5350	12.0000
59.5650	20.0000
59.5950	11.0000
59.6250	18.0000
59.6550	17.0000
59.6850	15.0000

59.7150	22.0000
59.7450	10.0000
59.7750	11.0000
59.8050	13.0000
59.8350	10.0000
59.8650	10.0000
59.8950	9.0000
59.9250	11.0000
59.9550	10.0000
59.9850	18.0000

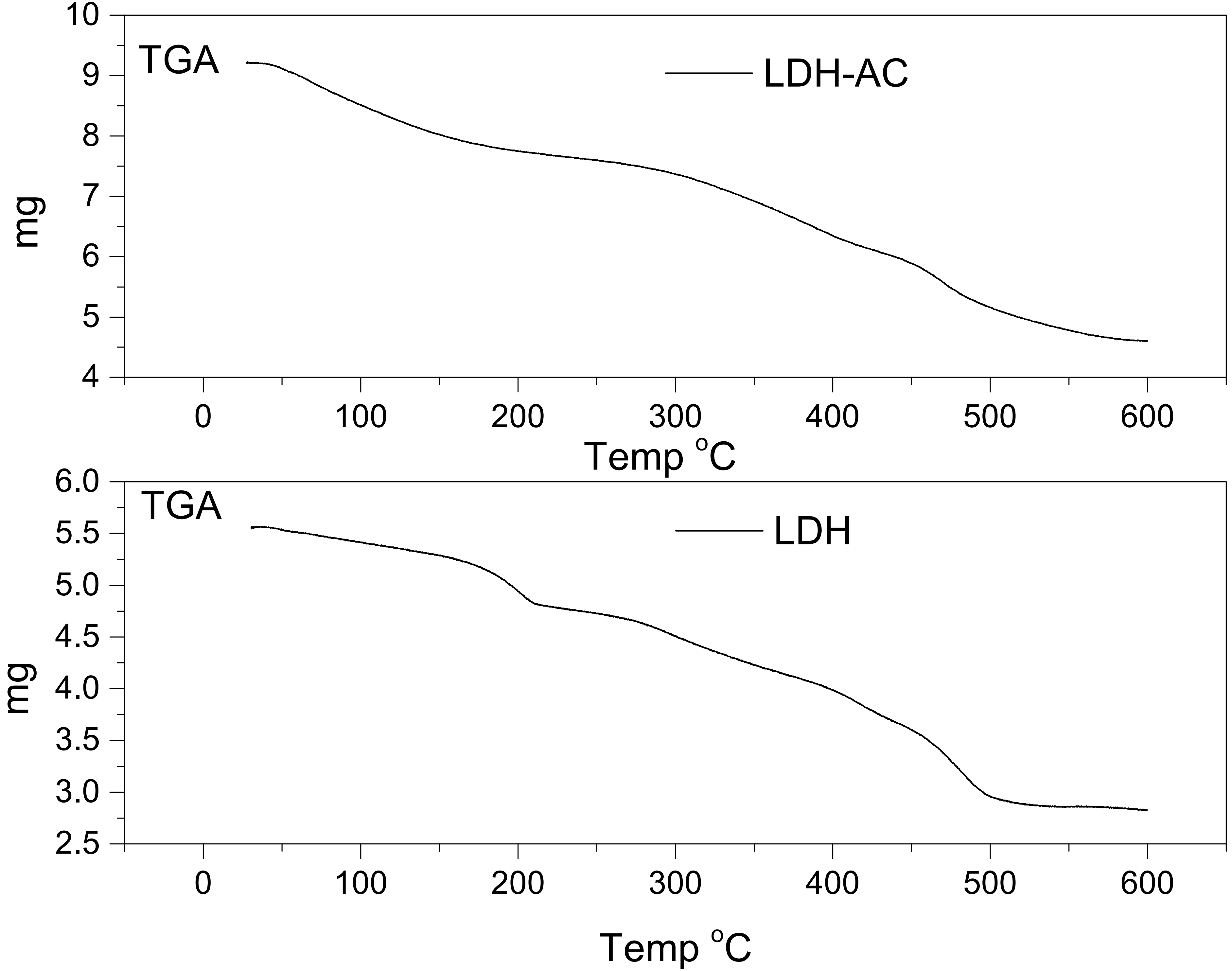
TGA
LDH-AC
mg
10
9
8
7
6
5
4
0
100
200
300
400
500
600
Temp °C
TGA
LDH
mg
6.0
5.5
5.0
4.5
4.0
3.5
3.0
2.5
0
100
200
300
400
500
600
Temp °C
